# Molecular Mechanisms of Drug Resistance in Natural *Leishmania* Populations Vary with Genetic Background

**DOI:** 10.1371/journal.pntd.0001514

**Published:** 2012-02-28

**Authors:** Saskia Decuypere, Manu Vanaerschot, Kirstyn Brunker, Hideo Imamura, Sylke Müller, Basudha Khanal, Suman Rijal, Jean-Claude Dujardin, Graham H. Coombs

**Affiliations:** 1 Department of Biomedical Sciences, Institute of Tropical Medicine, Antwerp, Belgium; 2 Strathclyde Institute of Pharmacy and Biomedical Sciences, University of Strathclyde, Glasgow, Scotland, United Kingdom; 3 Wellcome Trust Centre for Molecular Parasitology, Institute of Infection, Immunity and Inflammation, College of Medical, Veterinary and Life Sciences, University of Glasgow, Glasgow, Scotland, United Kingdom; 4 Wellcome Trust Sanger Institute, Wellcome Trust Genome Campus, Hinxton, England, United Kingdom; 5 B.P. Koirala Institute of Health Sciences, Ghopa, Dharan, Nepal; 6 Department of Biomedical Sciences, University of Antwerp, Antwerp, Belgium; McGill University, Canada

## Abstract

The evolution of drug-resistance in pathogens is a major global health threat. Elucidating the molecular basis of pathogen drug-resistance has been the focus of many studies but rarely is it known whether a drug-resistance mechanism identified is universal for the studied pathogen; it has seldom been clarified whether drug-resistance mechanisms vary with the pathogen's genotype. Nevertheless this is of critical importance in gaining an understanding of the complexity of this global threat and in underpinning epidemiological surveillance of pathogen drug resistance in the field. This study aimed to assess the molecular and phenotypic heterogeneity that emerges in natural parasite populations under drug treatment pressure. We studied lines of the protozoan parasite *Leishmania (L.) donovani* with differential susceptibility to antimonial drugs; the lines being derived from clinical isolates belonging to two distinct genetic populations that circulate in the leishmaniasis endemic region of Nepal. Parasite pathways known to be affected by antimonial drugs were characterised on five experimental levels in the lines of the two populations. Characterisation of DNA sequence, gene expression, protein expression and thiol levels revealed a number of molecular features that mark antimonial-resistant parasites in only one of the two populations studied. A final series of *in vitro* stress phenotyping experiments confirmed this heterogeneity amongst drug-resistant parasites from the two populations. These data provide evidence that the molecular changes associated with antimonial-resistance in natural *Leishmania* populations depend on the genetic background of the *Leishmania* population, which has resulted in a divergent set of resistance markers in the *Leishmania* populations. This heterogeneity of parasite adaptations provides severe challenges for the control of drug resistance in the field and the design of molecular surveillance tools for widespread applicability.

## Author Summary

Drug resistance is a serious problem that strikes at the core of infectious disease control. The mechanisms developed by pathogens to become resistant against existing drug treatments have been studied for many years but these studies have frequently scrutinized a few lines of the pathogen and rarely is it known whether the mechanisms identified occur in all pathogen populations present in endemic regions. In this study we assessed the diversity amongst drug-resistant parasites which emerged under treatment pressure in a natural parasite population. An extensive molecular and phenotypic characterisation of a collection of *Leishmania donovani* parasites isolated from leishmaniasis patients revealed that the parasites which are resistant to treatment have heterogeneous characters. The results provide evidence that how a parasite develops resistance under treatment pressure depends upon its genetic background. These findings provide key insights into the challenge that drug resistance poses for the control of infectious diseases like leishmaniasis.

## Introduction

*Leishmania*, a protozoan parasite transmitted by phlebotomine sand flies, causes a neglected infectious disease commonly referred to as leishmaniasis. The species *Leishmania (Leishmania) donovani* is the causative agent of visceral leishmaniasis (VL) or kala-azar, the most severe form of leishmaniasis which is lethal if left untreated [1], [2]. Pentavalent antimonials, such as sodium stibogluconate (SSG), have been the first-line treatment for leishmaniasis worldwide for more than 70 years. However, this therapy is challenged by emergence of resistant parasites [3]–[6], a phenomenon best documented in the Indian subcontinent with SSG-resistant *L. (L.) donovani* being reported in both India [7] and Nepal [8]. In the late 1990s it was reported that parasite resistance to antimonials consistently correlated with SSG treatment failure in the Indian province Bihar [7], [9], and appeared to cause up to 60% treatment failure in that region [5]. In contrast, in the neighbouring Nepalese VL endemic region VL patients infected with SSG-resistant parasites were found to have only a 25% risk of SSG treatment failure [8].

**Figure 1:**
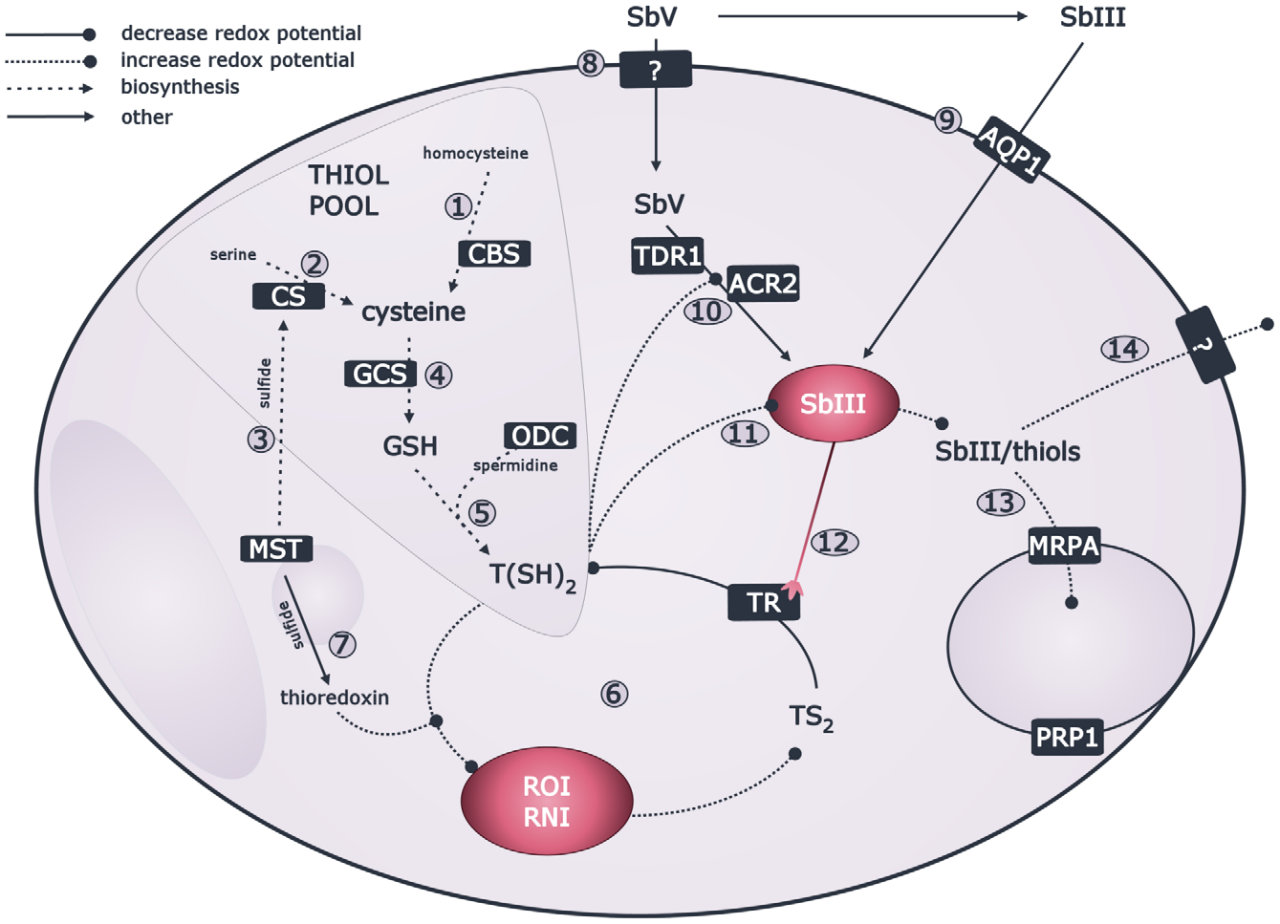
Overview of *Leishmania* pathways involved in response to SSG. The defence against oxidative and nitrosative radicals imposed by macrophages relies on antioxidants here represented in the thiol pool with 3 major thiols: (a) cysteine with 2 synthesis pathways in trypanosomatids including transsulfuration pathway (as in vertebrates) via homocysteine with a key enzyme cystathione β-synthase (CBS) (#1), and de novo synthesis from serine (as in some prokaryotes and eukaryotes) catalysed by cysteine synthase (CS) (#2); with sulfide possibly provided by mercaptopyruvate sulfurtransferase (MST) (#3) [60], [61]; (b) glutathione (GSH) synthesised by condensation of cysteine and glutamate with the key enzyme γ-glutamylcysteine synthase (GCS) (#4) [62]; and (c) trypanothione (T(SH)_2_) which is synthesised by condensation of glutathione and spermidine; with ornithine decarboxylase (ODC) as key enzyme of spermidine synthesis (#5) [63]. Maintenance of redox balance is centred around the strong reductant T(SH)_2_ and the flavo-enzyme trypanothione reductase (TR) which keeps the T(SH)_2_ pool reduced and the redox potential low (#6) [40], [63]–[65]; MST possibly also has an antioxidant role via thioredoxin (#7) [61]. The drug SbV can be taken up by the parasite via an unidentified transporter (#8) [66] and the reduced form SbIII gains access via aquaglyceroporin (AQP1) (#9) [67]. The reduction of SbV to SbIII involves thiols and can be a non-enzymatical spontaneous reduction [68]–[71], or enzymatically catalyzed by thiol dependent reductase 1 (TDR1) [72] or arsenate reductase 2 (ACR2) [73] (#10). The resulting SbIII can form conjugates with thiols and inhibit TR (#12), together leading to increase of redox potential [12]–[14], [39]. The SbIII/thiol conjugates can be sequestered by ABC transporter multidrug resistance protein A (MRPA) or possibly pentamidine resistance protein 1 (PRP1) [74], [75] into intracellular organelles (#13), or can be directly pumped out by an uncharacterised transporter (#14) [76] (boxed names = proteins of which the corresponding genes were chosen for DNA sequencing and gene expression profiling in this study, unboxed names = metabolites; used abbreviations: ROI = reactive oxygen intermediates, RNI = reactive nitrogen intermediates).

Pentavalent antimonials are considered to have a dual action mode whereby (i) the administered formulation sodium stibogluconate stimulates the infected macrophages (mΦ) to impose oxidative/nitrosative stress on the intracellular parasites [10], [11], and (ii) the reduced form of the drug, trivalent antimony (SbIII), acts directly on the parasite by perturbing its redox-balance [12]–[14]. Figure 1 shows a schematic overview of the cellular processes known to be affected or involved in the mode of action of antimonials. The adaptations that *Leishmania* requires to resist this complex drug pressure are poorly understood. It has been shown that SSG-resistant *L. (L.) donovani* clinical isolates have a polyclonal origin, which could be explained by frequently and independent events of SSG-resistance emergence throughout the endemic region [15]–[17]. It is not known how variable or invariable the associated molecular changes are at each emergence event although results from studies investigating antimonial resistance in different *L. (L.) donovani* clinical isolates have suggested a very heterogeneous situation. Many different approaches have been used to study various clinical isolates and this has led to the tentative identification of multiple putative markers of SSG-resistance including heat shock proteins [18], histones [19], surface proteins [20]–[22], and various transporters [20], [21], [23]–[25]. The up-regulation of antioxidant pathways in SSG-resistant parasites has been most frequently reported and is thought to entail possible cross-resistance to oxidative/nitrosative stress [21], [23], [26]–[29]. It is currently not known whether any of these putative markers is universal to all SSG-resistant parasites and the number of clinical isolates included in individual studies so far has been too limited to make conclusions on the homogeneous or heterogeneous character of the SSG-resistant parasite population on the Indian subcontinent [21], [23], [26]–[28].

The aim of this study was to address whether or not SSG-resistant *Leishmania* emerging in a particular endemic region evolved via similar molecular adaptations. For this purpose, we designed a comprehensive characterisation of the parasite pathways implicated in SSG/SbV/SbIII metabolism using a collection of parasite strains isolated from Nepalese VL patients. The resulting data suggest that the SSG-resistant *Leishmania* parasites in this single endemic region have heterogeneous molecular and phenotypic features related to drug resistance. Our findings highlight the necessity of molecular typing of emerging drug resistant parasite populations in order to fully understand the nature, complexity and consequences of the appearance of drug resistance.

## Methods

### Ethics statement

Written informed consent was obtained from the patients and in case of children from the parents or guardians. Ethical clearance was obtained from the institutional review boards of the Nepal Health Research Council, Kathmandu, Nepal and the Institute of Tropical Medicine, Antwerp, Belgium.

### Parasites

Parasites were isolated from bone marrow aspirates from confirmed VL patients recruited at the B.P. Koirala Institute of Health Sciences, Dharan, Nepal between 2002 and 2003 as described before [8] with a success rate of approximatively 60%. All clinical isolates were obtained at time of diagnosis, before SSG treatment, except for 3 isolates (BPK164/1, BPK177/0 and BPK181/12) which were obtained at time of adjudged treatment failure. The methods used for species typing, genotyping and *in vitro* antimonial testing of the clinical isolates were performed as described previously [8]. Briefly, all isolates were typed as *L. (L.) donovani* based on a CPB PCR-RFLP assay [30] and were further genotyped using kDNA PCR-RFLP [15], Illumina whole genome sequencing [16] and typing of 130 SNPs on the Sequenom platform [17]. Genotyping of the parasite strain collection used in this study revealed 2 major genetic clusters, designated here as population A and B. Population A is very homogenous and geographically clustered, while population B is heterogeneous compared to population A and geographically scattered [15]. Selected parasite isolates were cloned using the micro-drop method [31], to obtain homogenous working parasite populations for study, which we further refer to as clones. All clones were confirmed to be *L. donovani* by *CPB* PCR-RFLP analysis. All strains were infectious for macrophages in vitro [8] and 12 of them were previously studied for infectivity in vivo, showing that all are infective to BALB/c mice [32]. The *in vitro* susceptibility of a strain for SSG or SbIII is expressed as an activity index (A.I.), which is defined as the ratio of the ED50 of a tested strain versus the ED50 of the SSG-sensitive reference strain included in each assay. Isolates with an A.I. of 1–2 were considered sensitive to the tested drug, while isolates with an A.I. of 3 or higher were considered resistant. The details of all isolates and derived clones included in this study are given in Table 1.

**Table 1:** Geographic, clinical and biological characteristics of *L. donovani* strains.

parasite isolate code	A.I. SSG isolate^a^	derived clones	A.I. SSG clone^a^	origin (district)	SSG treatment outcome	population	DNA^c^	RNA^b^	protein^c^	thiol^c^	stress test^c^
MHOM/NP/03/BPK206/0	1	clone 10	1	Sunsari	definite cure	A	✓	✓	✓	✓	✓
		clone 14	1						✓	✓	✓
		clone 20	1						✓	✓	✓
MHOM/NP/02/BPK090/0	1	na	na	Sunsari	definite cure	A		✓			
MHOM/NP/02/BPK091/0	1	na	na	Sunsari	definite cure	A		✓			
MHOM/NP/03/BPK282/0	1	clone 4	1	Sunsari	definite cure	B	✓	✓	✓	✓	✓
		clone 9	1						✓	✓	✓
MHOM/NP/03/BPK294/0	2	clone 1	2	Siraha	definite cure	B	✓	✓			
MHOM/NP/02/BPK035/0	1	clone 1	1	Saptari	definite cure	B	✓	✓			
MHOM/NP/02/BPK043/0	1	clone 2	1	Sunsari	definite cure	B	✓	✓			
MHOM/NP/03/BPK181/0	1	na	na	Sunsari	relapse	B		✓			
MHOM/NP/03/BPK190/0	6+	clone 3	6+	Morang	non-responder	A	✓	✓	✓	✓	✓
		clone 11	6+						✓	✓	✓
		clone 19	6+						✓		✓
MHOM/NP/02/BPK025/0	6	na	na	Sunsari	definite cure	A		✓			
MHOM/NP/02/BPK087/0	6+	clone 11	5	Morang	definite cure	A	✓	✓		✓	
MHOM/NP/03/BPK279/0	6+	na	na	Morang	definite cure	A		✓			
MHOM/NP/03/BPK298/0	6+	clone 8	1	Sunsari	definite cure	A	✓	✓			
MHOM/NP/03/BPK275/0	6+	clone 12	6+	Morang	non-responder	B			✓	✓	✓
		clone 15	6+						✓		✓
		clone 17	6+						✓		✓
		clone 18	6+				✓		✓	✓	✓
MHOM/NP/02/BPK085/0	6+	clone 3	6+	Saptari	definite cure	B		✓		✓	
		clone 8	6+				✓			✓	
MHOM/NP/02/BPK164/1	6+	na	na	Dhanusa	non-responder	B		✓			
MHOM/NP/02/BPK177/0	6	na	na	Dhanusa	relapse	B		✓			
MHOM/NP/02/BPK178/0	6	clone 3	1	Sunsari	definite cure	B	✓	✓			
MHOM/NP/03/BPK181/12	6	na	na	Sunsari	relapse	B		✓			

**a** = *in vitro* antimonial susceptibility as described in Rijal *et al.* [8], A.I. or activity index is defined as the ratio of the EC50 of a particular isolate or clone versus the EC50 of reference isolate sensitive to SSG. The activity index is used to express the *in vitro* susceptibility of that tested isolate or clone, isolates or clones with an activity index between 1 and 2 are considered as sensitive to antimonials (white background), while those showing an activity index between 3 and 6 are considered to be *in vitro* resistant (grey background), **b** = experiment done with clinical isolates, **c** = experiment done with clones derived from clinical isolates.

### *In vitro* promastigote growth

Promastigotes were grown on modified Eagle's medium [33] (Invitrogen) supplemented with 20% (v/v) heat inactivated foetal calf serum (PAA laboratories GmbH) pH 7.5 at 26°C. The cultures were initiated by inoculating parasites at day 4 stationary phase into 50 mL culture medium to give a density of 5×10^5^ parasites/mL; the resulting inoculated medium was equally distributed over 10 culture flasks. Every 24 hrs for 8 consecutive days, parasite density was determined to record parasite growth. Monitoring of parasite growth and concomitant sampling at various time-points was repeated for production of DNA extracts for sequencing, mRNA extracts for gene expression profiling, protein extracts for protein profiling, metabolite extracts for thiol profiling and *in vitro* oxidative and nitrosative stress testing.

### DNA sequencing

Promastigote pellets were harvested during stationary phase of promastigote growth and washed 3 times in PBS. Three flasks of 5 mL cultures were needed for the required yield of 7–10 µg DNA. DNA isolation was performed from the dry pellets with the QIAamp DNA Mini Kit, according to manufacturer's instructions. From whole-genome sequencing data [16], the sequences of the 11 target genes and 500 bp upstream/downstream were retrieved and aligned to check for sequence polymorphism; read-depth analysis and SNP calling were done as described elsewhere [16] and the 11 targets all proved to be single-copy per haploid genome.

### RNA isolation, real-time quantitative PCR and comparative analysis

A promastigote pellet of each studied strain was harvested every 24 hrs for 8 consecutive days of *in vitro* promastigote growth. RNA was isolated, analysed and reverse transcribed as described before [34]. The resulting cDNA was 10 times diluted and used for quantitative PCR of 11 genes as described elsewhere [34]. Briefly, the mRNA levels of the 11 genes were determined at the 8 different time-points during promastigote growth in 1 quantitative experiment. The analysis of 11 genes in 144 samples could be performed as 1 integrated experiment by performing inter-plate calibrations (based on 3 samples included in each quantitative PCR-run) and centralised data-management with the qBase software package [35].

### Quantitative protein profiling by Western blot and comparative analysis

Duplicate pellets of 1×10^8^ promastigotes were harvested for each line at 4 different time-points during promastigote growth, washed in PBS and lysed by repeated aspiration in ice-cold lysis buffer (0.25 M sucrose, 0.25% triton X-100, 10 mM EDTA) containing a protease inhibitor mix (10 µM E-64, 2 mM 1,10-phenanthrolin, 4 µM pepstatin A, 1 mM phenylmethylsulfonylfluoride). Cell debris was removed by centrifugation at 12000× *g* for 10 min at 4°C. The soluble protein fraction was fractionated on a 12% SDS-PAGE and electroblotted to nitrocellulose membranes (Amersham Biosciences). Membranes were blocked for 2 hrs at room temperature in TBS containing 5% milk and 0.2% Tween 20. Immuno-blotting of proteins of interest was done overnight with the primary antibodies anti-TR (trypanothione reductase) and anti-MST (mercaptopyruvate sulfurtransferase). Protein loading per well was assessed with anti-OPB (oligopeptidase B) antibody which was shown to have a stable expression throughout *Leishmania* promastigote growth [36]. The secondary antibodies used were rabbit anti-IgG and antibodies were detected using peroxidise-linked anti-rabbit IgG and ECL reagents (Pierce). The Western blots were digitally analysed using the software package Image J 1.38× (NIH, USA). The background of all blots was subtracted automatically before measuring the intensity of specific bands. Integrated densities for each band were determined for each protein of interest and its corresponding loading control. The ratio of the band intensity of the protein of interest versus the band intensity of the corresponding loading control was used as relative protein expression level and allowed comparison with other samples. All duplicate samples for each of the 4 time-points of 1 particular line were analysed in 1 blot.

### Quantification thiol levels using HPLC and comparative analysis

Thiol levels were quantified by HPLC as described previously [37] with minor modifications. Briefly, triplicate cultures of 2.5×10^7^ promastigote were harvested at 4 different time-points during promastigote growth and were prepared independently for thiol quantification. Following cold washing steps to remove extracellular medium, each pellet was resuspended in 40 mM HEPPS in 2 mM EDTA, pH 8.0 containing 0.7 mM TCEP and incubated for 45 min at room temperature during which cellular thiols were reduced by the agent TCEP. The reduced thiols were subsequently derivatised by adding 50 µL 2 mM monobromobimane, a fluorescent thiol-specific reagent, and immediate heating for 3 min at 70°C. After briefly cooling on ice and a deproteinisation step, the resulting supernatant with the derivatised thiols was kept for HPLC analysis. Separation and quantification of the derivatised thiols was done by high pressure liquid chromatography (HPLC, Dionex). The 3 thiols cysteine (CSH), glutathione (GSH) and trypanothione (T(SH)_2_) could be confidently identified in *Leishmania* extracts through comparison with chromatograms of commercially available cysteine (Sigma), glutathione (Sigma) and trypanothione (BACHEM). These commercially available thiols were also used to prepare standard solutions containing all 3 thiols in variable concentrations but with a total thiol concentration comparable to the content of a *Leishmania* lysate. The obtained thiol mixtures were used to determine (i) the correlation between fluorescence peak area and thiol concentration and (ii) the linear range of detection. A volume of 25 µL of each prepared derivatised *Leishmania* extract was injected on the column for thiol quantification. A mobile phase consisting of 0.25% acetic acid (solvent A) and 100% acetonitrile (solvent B) was applied to separate the different thiols. Gradient elution started with 100% solvent A and 0% solvent B. After 40 min, solvent B was increased to 8% for 60 min, to 15% for the subsequent 10 min, and finally to 50% for 1 min. The system returned to the initial solvent composition with 0% solvent B for the remaining 10 min of the run. Thiols were detected as they ran off the column by a fluorescence spectrophotometer (excitation 365 nm, emission 480 nm).

### *In vitro* promastigote susceptibility test for oxidative and nitrosative stress and comparative analysis

Promastigote susceptibility for 3 different oxidative/nitrosative stresses (Table 2) was assessed using Alamar blue (AB) assays as described elsewhere [38], with minor modifications. Briefly, at 3 different time-points during promastigote growth, parasites were plated in quadruplicate in 96-well plates at an initial density of 5×10^5^ parasites/ml for 48 hrs incubation experiments. Each well with 100 µl parasite culture was topped with 100 µl drug solution prepared in 6 different dilutions (Table 2). The following drugs were used: Perdrogen hydrogen peroxide 30 WT % (Sigma-Aldrich), S-Nitroso-N-acetylpenicillamine (SNAP, Biomol International), and potassium antimonyl tartrate trihydrate (SbIII, Sigma-Aldrich). Quadruplicate untreated controls and blanks were included in each test plate. The culture plates were sealed and incubated at 26°C. AB reagent (Invitrogen) was added 24 hrs before the end of drug incubation to test cell viability, and AB fluorescence was read at the end of the drug incubation using 555 nm excitation wavelength and 585 nm emission wavelength.

**Table 2:** Conditions used for the oxidative/nitrosative stress phenotyping assays of *L. (L) donovani* clones.

Drug	nature stress	final drug concentration (range tested)
H_2_0_2_	direct oxidative	0–100–150–300–500–1000–1500 µM
SNAP (NO-donor)	direct nitrosative	0–5–20–50–150–300–500 µM
SbIII	indirect oxidative+nitrosative	0–2–7–20–70–200–700 µg/mL

AB fluorescence data from treated and non-treated cultures was used to calculate the IC50 by sigmoidal regression analysis (with variable slope) using GraphPad Prism v.5.02.

### Statistical analysis

The change of all measured variables (RNA/protein/thiol/IC50) was evaluated (i) in relation to the SSG-phenotype and (ii) in relation to time during *in vitro* growth with a repeated measures two-way ANOVA (ANOVA p-value indicated under each heatmap) followed by Bonferroni post-tests (significance indicated by an asterisk under each time point). Since experiments were repeated at different time-points during promastigote growth, the analysis was done with matching by time-points. Statistical analysis was done with GraphPad Prism v.5.02 from GraphPad Software, Inc. Heatmaps were plotted in R (www.r-project.org) using the plotting tools of the packages gplots and RColorbrewer.

## Results

**Figure 2:**
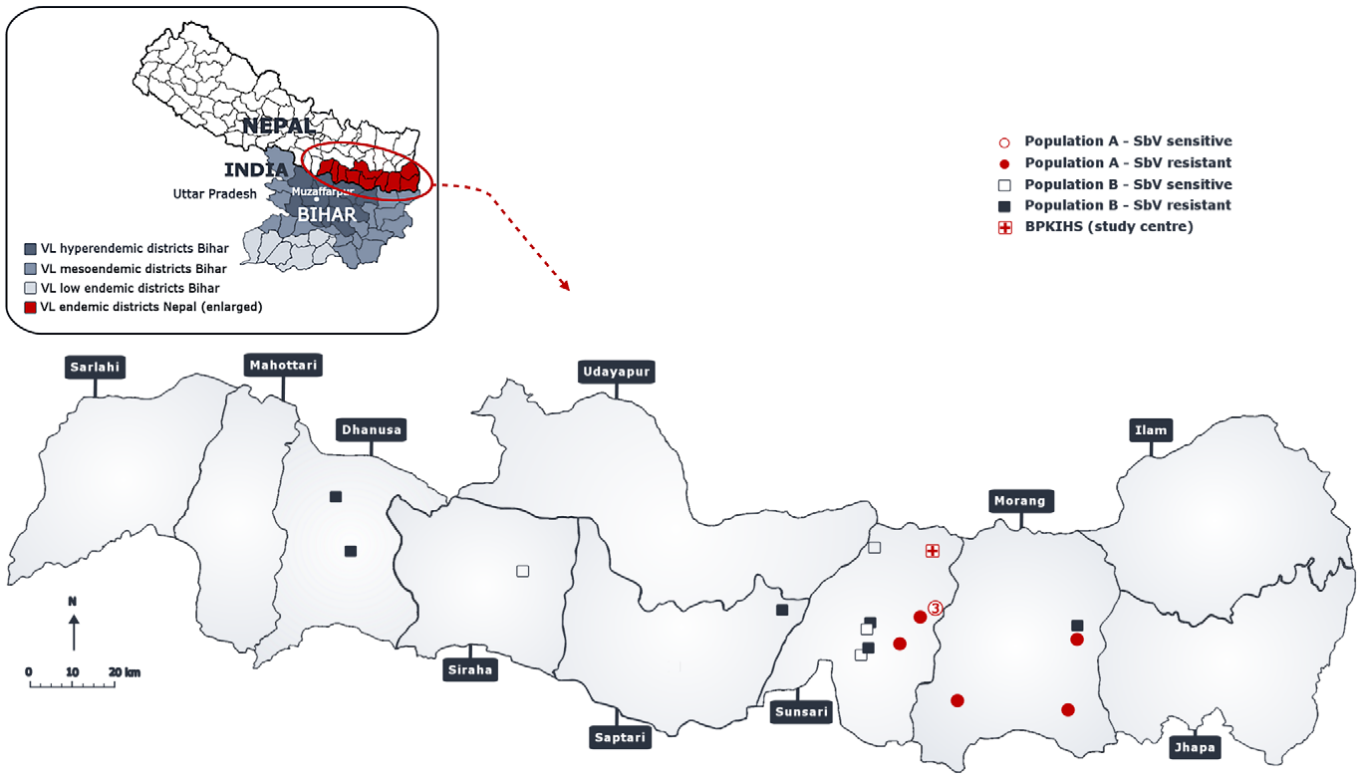
Geographic distribution of studied *L. (L.) donovani* isolates with population and *in vitro* SSG susceptibility information.

We selected 19 *L. (L.) donovani* clinical isolates with documented differential *in vivo* and *in vitro* SSG-susceptibility [8]: 8 of those isolates are *in vitro* sensitive to SSG (SSG-S) and 11 are *in vitro* resistant to SSG (SSG-R). Within this particular collection of *L. (L.) donovani* clinical isolates, kDNA fingerprinting showed there to be 2 distinct genotypic populations (here designated as population A and population B) differentiated by the highest minicircle sequence dissimilarity [15]. Population A (eight isolates) which is genetically very homogeneous and found in only 2 Eastern Nepalese districts, Sunsari and Morang (Figure 2), is thought to result from a recent clonal expansion [15]. In contrast, population B (eleven isolates) is much more heterogeneous at kDNA level than population A [15], and was found in the entire VL endemic region of Nepal (Figure 2). The higher homogeneity of population A (versus population B) was confirmed by whole genome sequencing [16] and genome-wide SNP typing [17]. PCA analysis revealed that population A clusters separately from population B (see fig. 1 in ref [16]) due to 35 homozygous SNPs - 19 coding, 11 non-synonymous and 8 synonymous. Populations A and B should be considered at present as model populations, as the parasite isolation success rate is only around 60% we cannot yet exclude the possibility that parasites with other genetic backgrounds occur in the region. Table 1 gives an overview of the geographical, clinical and biological characteristics of all parasite isolates (and derived clones) included in this study.

**Figure 3:**
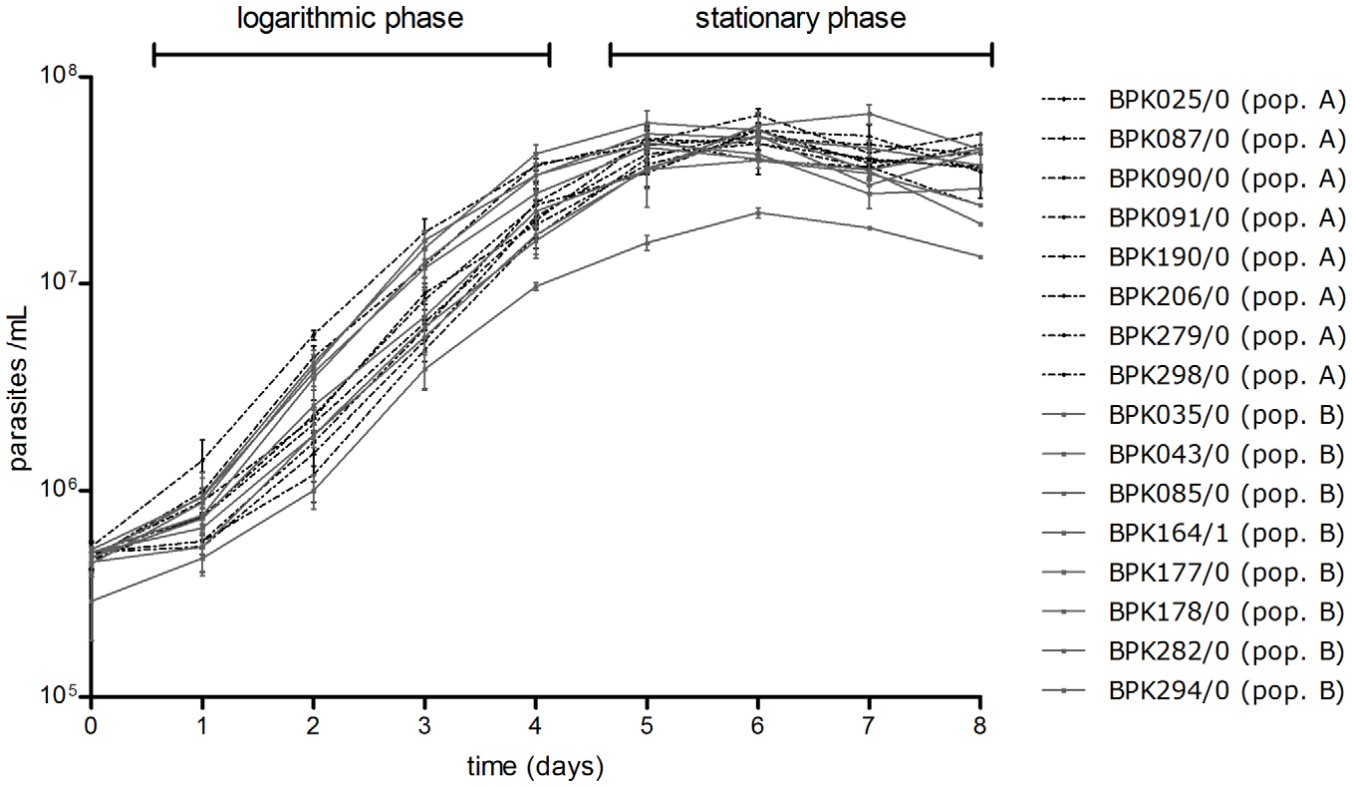
*In vitro* promastigote growth profiles of studied *L. (L.) donovani* isolates. The growth profiles of population A isolates (in black dotted curves) and population B isolates (in full black curves) largely coincide and clearly indicate that all studied isolates had a similar *in vitro* promastigote growth dynamic. The growth curve shown for each isolate represents the average growth assessed in 3 repeated experiments.

We comparatively characterised *in vitro* promastigotes of these *L. (L.) donovani* isolates (and derived clones) on the level of DNA sequence, RNA expression, protein expression, thiol content and oxidative/nitrosative stress phenotype. For the last 4 experiments, we monitored and sampled the promastigote cultures during 8 days *in vitro* growth, a process which consists of a logarithmic phase (day 1 to day 4) during which the parasites multiply, and a stationary phase (day 5 to day 8) during which metacyclogenesis (the development of the metacyclic promastigotes that are infectious to humans) occurs. The strains studied here all had a comparable *in vitro* growth dynamic (Figure 3). The purpose of this time-course analysis was two-fold: (i) to detect quantitative differences in parasite features at specific stages of growth, and (ii) to detect differential regulation of parasite features over time.

### Gene expression profiling of 11 targets

The gene expression levels of 11 target genes, selected on the basis of their reported relevance in the parasite's antioxidant response to SSG or pathways known to be affected by SSG (Figure 1), were assessed using quantitative PCR with 18 *L. (L.) donovani* clinical isolates (Table 1). The detailed results of the gene expression profiling experiments can be found in dataset S1.

**Figure 4:**
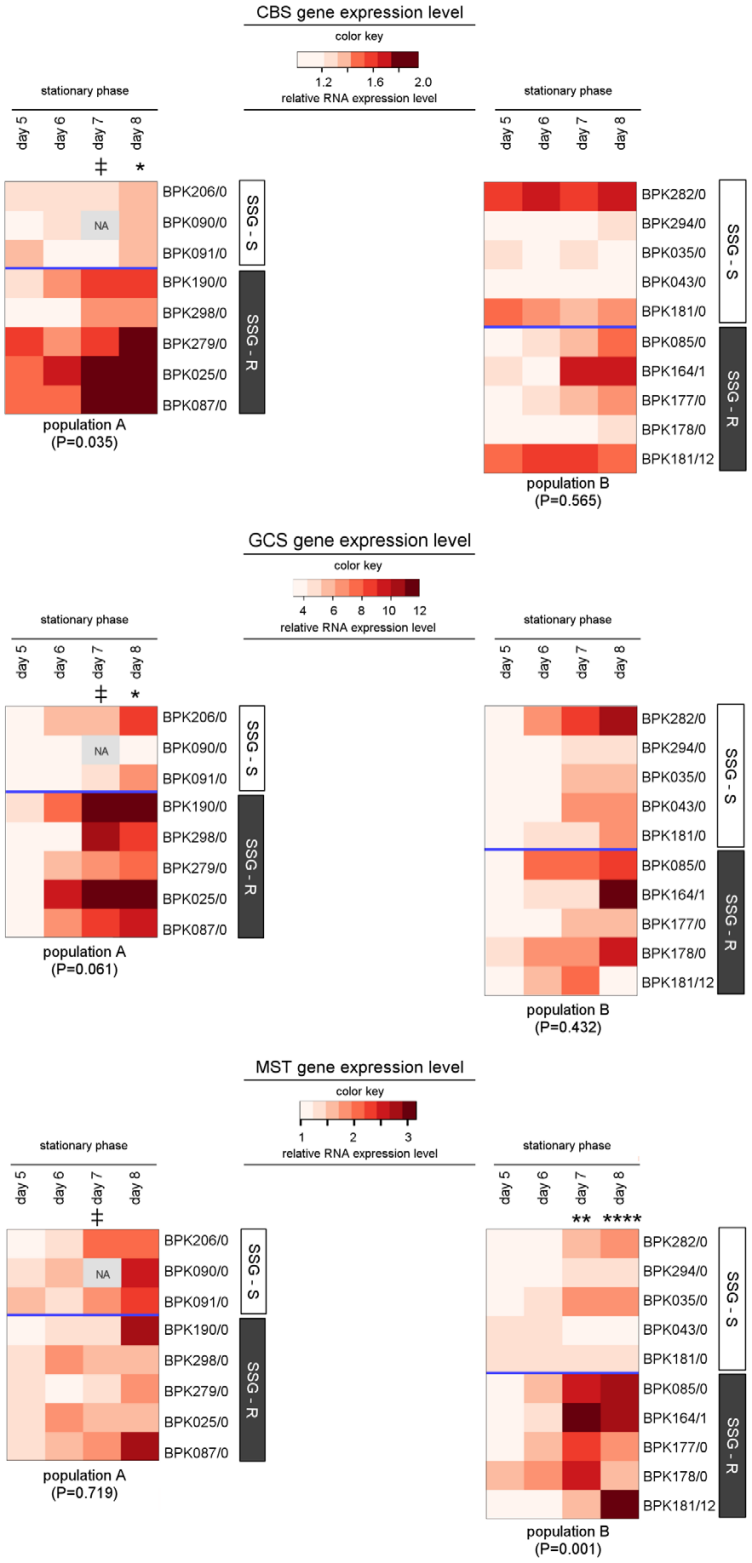
Heatmap of *CBS*, *GCS* and *MST* gene expression levels in 18 *L. (L.) donovani* isolates. The color key shown for each gene reveals the color-code used to visualise the relative gene expression values which were determined by Q-PCR, light red colors correspond to low relative gene expression levels while dark red colors correspond to high relative gene expression levels. An identical color key was used to present all results of 1 particular gene and allows comparison of gene expression levels between the 2 populations (population A in heatmap on the left, population B in heatmap on the right). The sampled isolates are presented along the Y-axis, their SSG-susceptibility status is indicated by the white (SSG-S)/grey (SSG-R) sidebars. Relative gene expression levels determined at 4 consecutive days during stationary phase of promastigote growth are visualised (data from logarithmic growth phase are not shown here); the included time-points are indicated on the X-axis. The P-value shown for each population reflects the overall similarity between the gene expression-profiles of SSG-S and SSG-R isolates within that population (repeated measures two-way ANOVA), and the asterisks further mark the specific time-points where gene expression levels were found to be significant different according to Bonferroni post-tests (* = P<0.05; ** = P<0.01, *** = P<0.001, **** = P<0.0001; ‡ = time-point excluded from statistical analysis due to missing values). The relative gene expression levels ± SD determined at all 8 time-points (logarithmic and stationary phase) of promastigote growth are given in dataset S1.

Comparison of the expression profiles of all SSG-S and all SSG-R isolates identified 3 genes (*CBS*, *GCS* and *MST*) with a significantly different expression profile in the 2 groups of isolates (Table 3). However, a more detailed analysis clarified that the expression levels of these 3 genes do not represent molecular markers of all SSG-R parasites, but rather characterise SSG-R parasites in either population A or B (Table 3 and visualised in the heatmaps of figure 4):

- In population A, the gene *CBS* has an overall significantly higher expression in SSG-R isolates compared to SSG-S isolates (P = 0.035). This difference gradually increases during stationary phase to reach 1.3-fold by day 8 (Figure 4). In population B, on the other hand, *CBS* profiles were similar for the 2 phenotypes (P = 0.565, Figure 4).
- *GCS* expression also appears to differ between SSG-S and SSG-R isolates of population A. Although we could not find an overall significant difference in population A (P = 0.061), the Bonferroni post-tests indicated that *GCS* expression increases more steeply during stationary phase in SSG-R compared to SSG-S isolates, with an average 1.5-fold difference between the 2 phenotypes by day 8. In population B, there was no difference between the SSG-S and SSG-R isolates with all showing a similar increase in *GCS* expression during stationary phase (P = 0.432).
- *MST* expression was, in contrast, significantly different between SSG-R and SSG-S isolates in population B (P = 0.001) but not in population A (P = 0.719). In population B, SSG-R isolates had a steeper increase during stationary phase compared to SSG-S isolates, reaching an average 1.8–1.9-fold difference at days 7 and 8 of culture.

**Table 3:** Relative expression levels of 11 genes in the different biological groups of *L. donovani* isolates.

gene	ALL ISOLATES differences between SSG-S and SSG-R	POPULATION A differences between SSG-S and SSG-R	POPULATION B differences between SSG-S and SSG-R
*CBS*	**yes (P = 0.050)**	**yes (P = 0.035)**	no (P = 0.565)
*GCS*	**yes (P = 0.034)**	no (P = 0.061)	no (P = 0.432)
*MST*	**yes (P = 0.037)**	no (P = 0.719)	**yes (P = 0.001)**
*TR*	no (P = 0.086)	no (P = 0.063)	no (P = 0.742)
*ACR2*	no (P = 0.188)	no (P = 0.354)	no (P = 0.251)
*AQP1*	no (P = 0.095)	no (P = 0.250)	no (P = 0.671)
*CS*	no (P = 0.815)	no (P = 0.801)	no (P = 0.923)
*ODC*	no (P = 0.124)	no (P = 0.487)	no (P = 0.138)
*TDR1*	no (P = 0.633)	no (P = 0.802)	no (P = 0.829)
*PRP1*	no (P = 0.847)	no (P = 0.619)	no (P = 0.599)
*MRPA*	no (P = 0.851)	no (P = 0.873)	no (P = 0.399)

Statistical analysis: P-values comparing the indicated groups of isolates were determined by repeated measures two-way ANOVA with matching by time-points. Data were missing from time-point ‘day 7’ and needed to be excluded from statistical analysis. The complete dataset of average relative gene expression levels (± SD) measured during 8 consecutive days *in vitro* promastigote growth can be found in dataset S1.

Thus the SSG-R isolates are marked by an enhanced gene expression during metacyclogenesis, however in each case in just 1 of the 2 populations studied. This heterogeneous profile for expression of these genes in the SSG-R isolates contrasts with the more homogenous profiles of the SSG-S isolates (Figure 4). The hypothesis arising from these experiments, that SSG-R isolates in the 2 populations have heterogeneous adaptations, instigated further molecular and biochemical comparison of the isolates. To avoid working with mixed parasite isolates for this investigation, we produced clones (derived from a single parasite picked from the isolate culture and grown as independent culture) of selected clinical isolates which were confirmed to be representative for population A and population B by whole genome sequencing and SNP typing [16], [17]. These clones (characteristics shown in Table 1) were used for the remainder of the study.

### DNA sequencing of coding and neighbouring non-coding regions of 11 genes of interest

Single nucleotide polymorphisms (SNPs) in the DNA sequence of the 11 studied genes (Figure 1) could modify performance of the encoded proteins (SNPs in coding regions) or could underlie the observed changes in gene expression (SNPs in non-coding regions), although such SNPs had not been reported previously in the context of antimonial resistance in *Leishmania*. In our study, the coding DNA sequence of the 11 genes of interest was determined by Illumina sequencing for 11 different parasite clones (5 SSG-S and 6 SSG-R, Table 1). All coding sequences were found to be 100% identical. Further screening of the neighbouring non-coding upstream and downstream sequences of all genes revealed that the upstream regions (500 bp) of the 11 genes were also completely conserved among the 11 clones. Some SNPs were found in the downstream regions (500 bp) of the genes coding for TR, MST and ACR2, but the SNP pattern did not correlate with either the genotypic diversity (populations A and B) or the SSG-susceptibility. The sequencing data are detailed in dataset S2.

### Protein expression profiling

Our protein analysis experiments focussed on MST and TR. The gene encoding MST was the one showing the most significant differential gene expression-profile between SSG-S isolates and SSG-R isolates (in pop. B) of all the genes analysed (Table 3). TR, on the other hand, did not have significantly different gene expression-profiles in the SSG-S and SSG-R isolates tested here, but has been implicated as a target of SbIII, the active form of SSG (Figure 1) [12], [14], [39]. We determined by Western blot analysis the relative protein levels within the measurable range at 4 different days during the 8-day promastigote growth for 6 clones of each population (Table 1).

**Figure 5:**
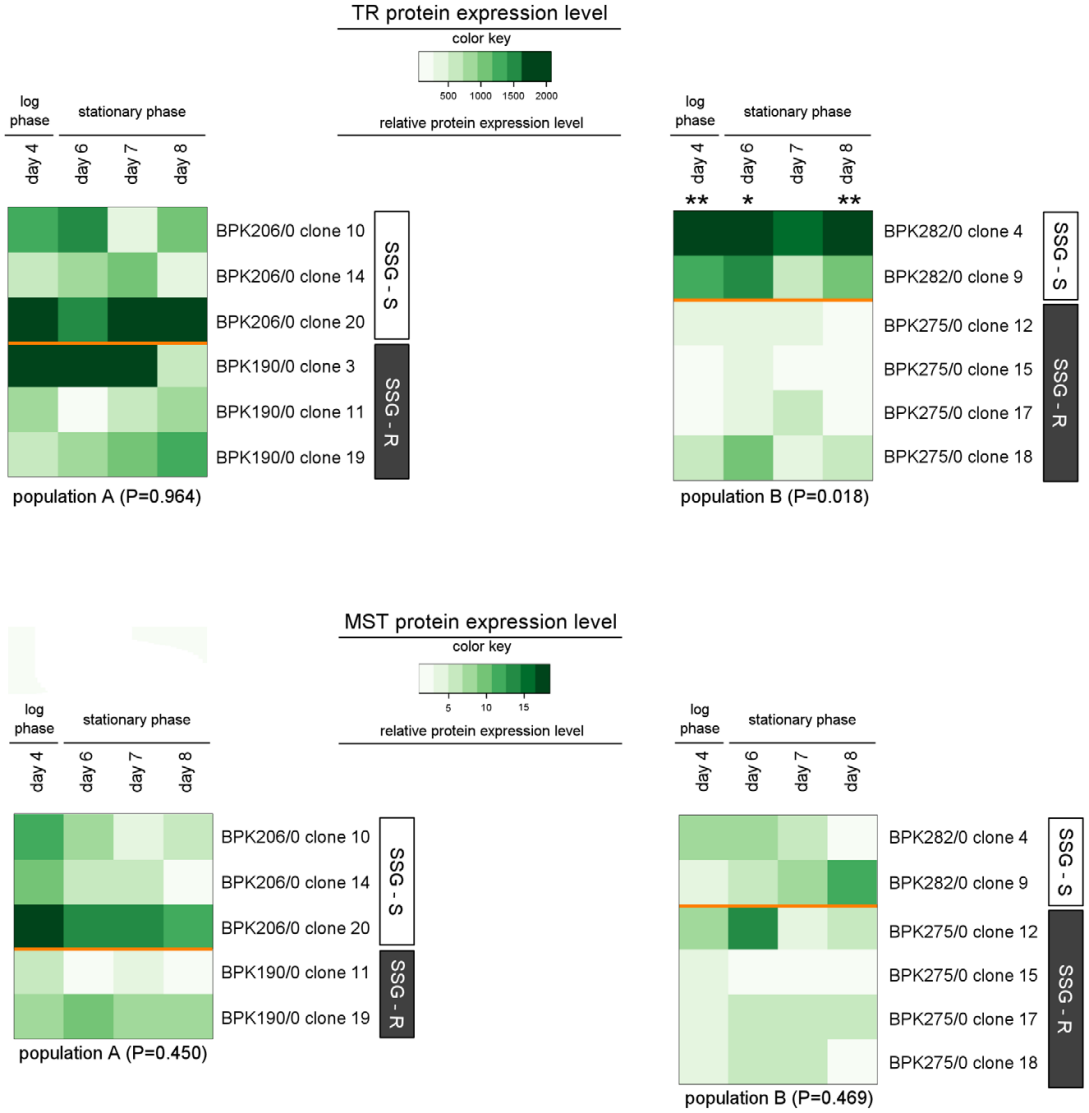
Heatmap of MST and TR protein expression levels of 12 *L. (L.) donovani* clones. The Western blots were digitally analysed by integrating the density of each protein band and its corresponding loading control (see materials and methods). The ratio of the band intensity of the protein of interest versus the band intensity of the corresponding loading control was defined as the relative protein expression level. The color key shown for each protein reveals the color-code used to visualise the relative protein expression level, light green colors correspond to low relative protein expression levels while dark green colors correspond to high relative protein expression levels. An identical color key was used for the 2 heatmaps of 1 particular protein and allows comparison of protein expression levels between the 2 populations (population A in heatmap on the left, population B in heatmap on the right). The sampled clones are presented along the Y-axis, their SSG-susceptibility status is indicated by the white (SSG-S)/grey (SSG-R) sidebars. Average protein expression levels from duplicate cultures were assessed at 4 different days during promastigote growth; the included time-points are indicated on the X-axis. The P-value shown for each population reflects the overall similarity between the protein expression-profiles of SSG-S and SSG-R clones within that population (repeated measures two-way ANOVA), and the asterisks further mark the specific time-points where protein expression levels were found to be significant different according to Bonferroni post-tests (* = P<0.05; ** = P<0.01, *** = P<0.001, **** = P<0.0001). The relative protein expression levels ± SEM determined at the 4 time-points of promastigote growth are given in dataset S3.

The digitally integrated protein expression levels of TR and MST are visualised in the heatmaps of figure 5; the full dataset is given in dataset S3. MST expression-profiles were overall similar within population A (P = 0.450) and within population B (P = 0.469). The protein profiles of TR are also comparable amongst the clones of population A (P = 0.964), but in population B the SSG-R clones were found to have an average 3.9-fold lower protein level compared to the SSG-S clones (P = 0.018, Figure 5). Bonferroni post-tests indicated that this difference was statistical significant at 3 out of 4 time-points tested.

### Thiol quantity profiling

Thiols are the central metabolites of *Leishmania*'s redox metabolism [40], and in this study were quantified at 4 different days during the 8-day promastigote growth for 6 clones of each population (Table 1). The resulting thiol quantities are in the same range as reported by other *L. (L.) donovani* studies [41]. The complete thiol quantification data are given in dataset S4.

**Figure 6:**
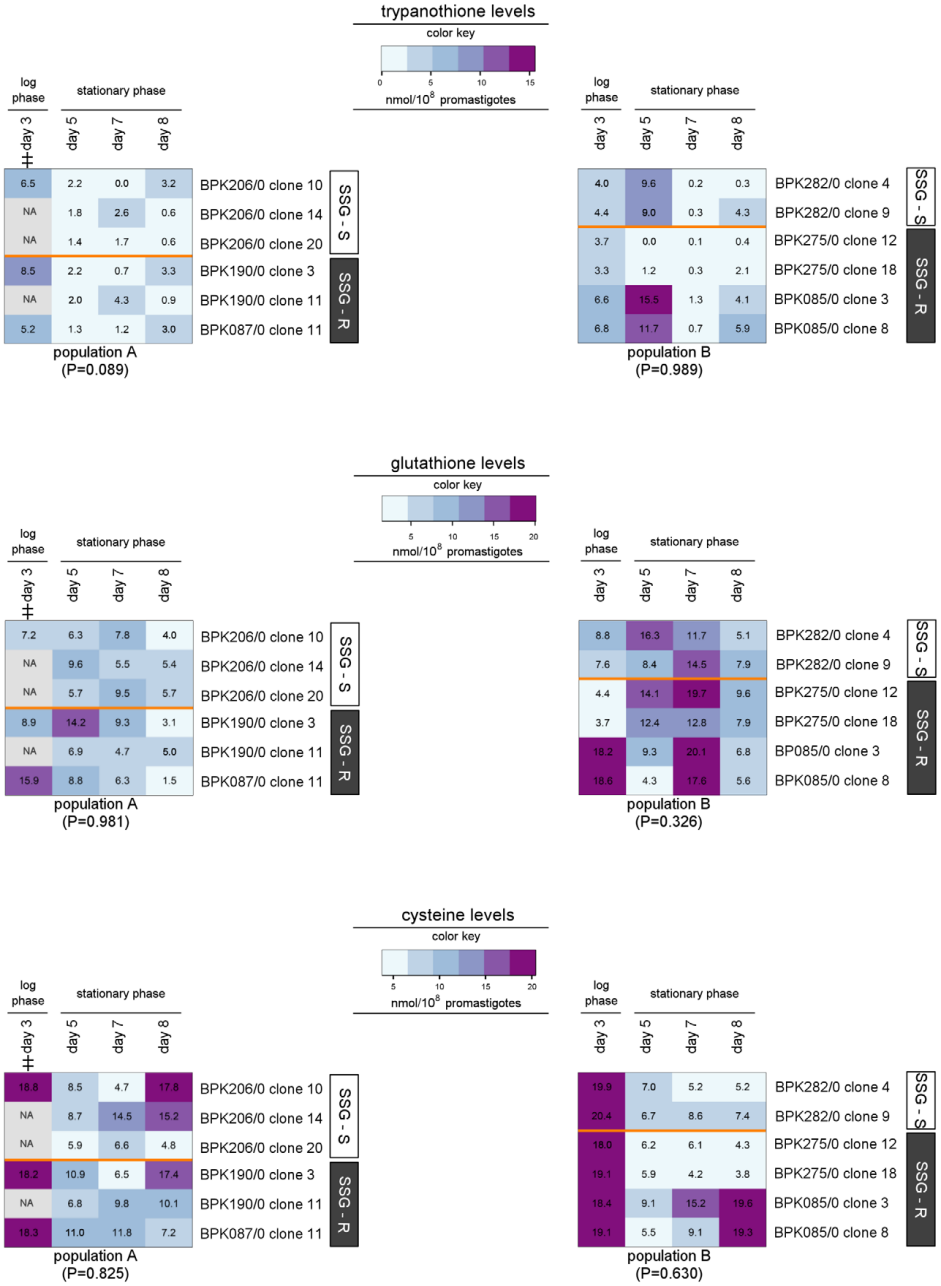
Heatmap of thiol levels of 12 *L. (L.) donovani* clones. The color key shown for each thiol reveals the color-code used to visualise the thiol quantities expressed in nmol/10^8^ parasites, light blue values correspond to low concentrations while purple values correspond to high concentrations. An identical color key was used for the 2 heatmaps per thiol and allows comparison of thiol quantities between the 2 populations (population A in heatmap on the left, population B in heatmap on the right). In addition, the actual thiol quantities are shown as labels on the heatmap. The tested clones are presented along the Y-axis, their SSG-susceptibility status is indicated by the white (SSG-S)/grey (SSG-R) sidebars. Average thiol levels from triplicate cultures were determined at 4 different days of promastigote growth; the included time-points are indicated on the X-axis. The P-value shown for each population reflects the overall similarity between the thiol levels of SSG-S and SSG-R clones within that population (repeated measures two-way ANOVA), ‡ = time-point excluded from statistical analysis due to missing values. The thiol quantities ± SEM determined at the 4 time-points of promastigote growth are given in dataset S4.

The thiol levels in the 2 populations are visualised in the heatmaps of figure 6. The trypanothione and cysteine profiles are generally comparable for all tested clones. We also did not find a correlation between glutathione levels and SSG-susceptibility of the tested clones. However there was a significant overall difference in glutathione levels between population A and B, with the clones of population B having an average 1.5-fold higher glutathione level compared to those of population A (P = 0.0004). Although the levels detected were significantly different, it is not possible with the current knowledge to know whether or not such a small variation is of any biological significance. Interestingly, differences were found between the cysteine and glutathione levels in log and stationary phase promastigotes, however as this was not central to this study it was not investigated further

### *In vitro* oxidative/nitrosative stress phenotyping

*In vitro* oxidative and nitrosative stress exposure of promastigotes was chosen as a phenotypic approach to verify if the molecular modifications leading to *L. (L.) donovani* SSG-resistance confer a modified tolerance to oxidative/nitrosative stress, which has been hypothesised from many other studies [21], [23], [26]–[29]. We exposed 6 clones of each population (Table 1) to 3 different oxidative/nitrosative stresses: (i) hydrogen peroxide (H_2_0_2_), a powerful oxidising agent; (ii) S-nitroso-N-acetylpenicillamine (SNAP), an NO donor imposing a nitrosative stress; and (iii) SbIII, a metalloid compound closely related to SSG which imposes an indirect oxidative/nitrosative stress by binding thiols and inhibiting the enzyme TR [12]–[14]. The stress susceptibility of the clones was determined on 3 consecutive days (days 5 to 7) during the stationary phase of the 8-day promastigote cultures. Table 2 summarises the conditions of the performed *in vitro* susceptibility tests.

**Figure 7:**
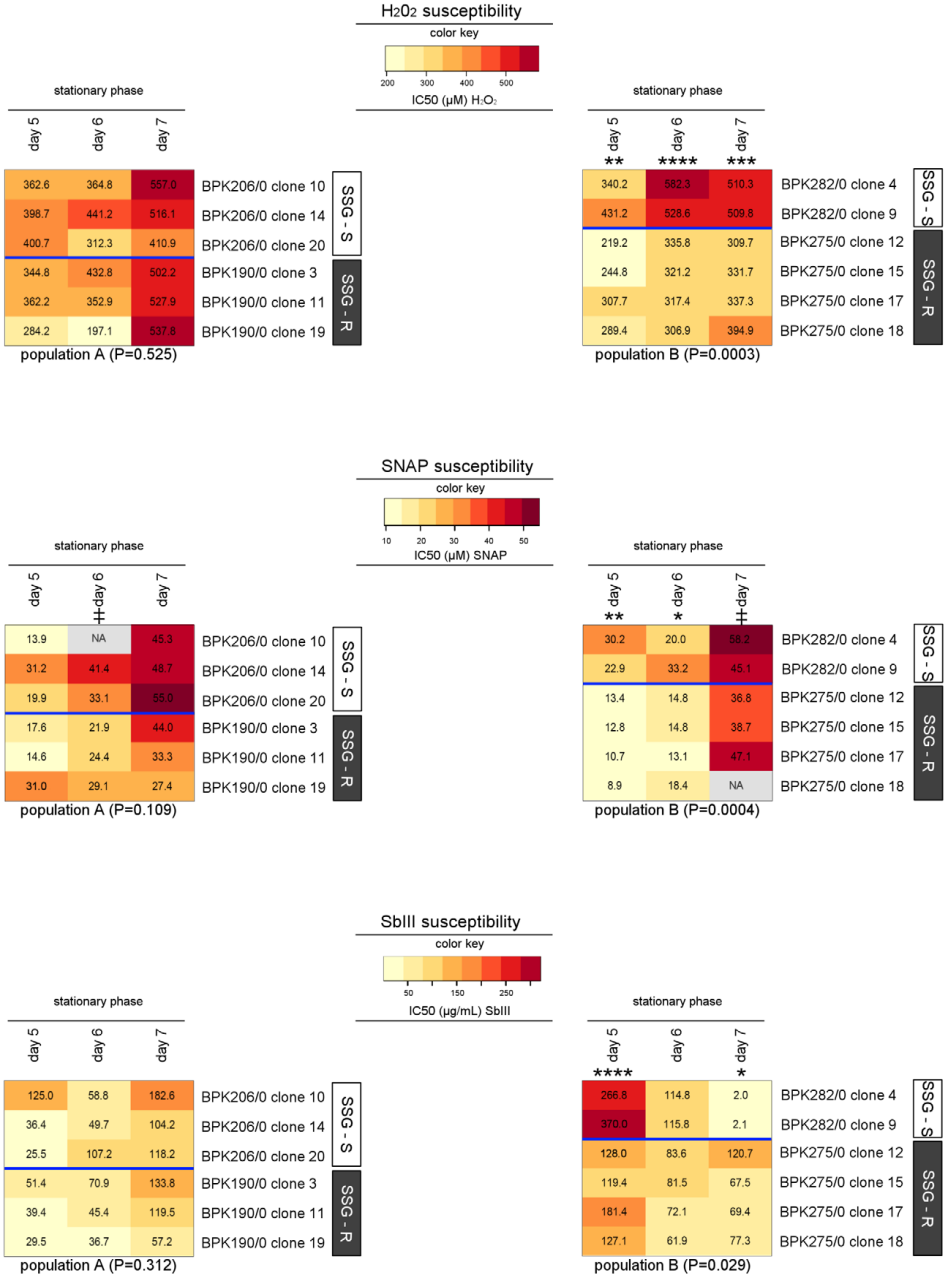
Heatmap of i*n vitro* oxidative and nitrosative stress susceptibility of 12 *L. (L.) donovani* clones. The color key shown for each stress test reveals the color-code used to visualise the determined IC50-values, light yellow colors correspond to low IC50-values and dark orange colors correspond to high IC50-values. An identical color key was used for the 2 heatmaps per stress-test and allows comparison of IC50-values between the 2 populations (population A in heatmap on the left, population B in heatmap on the right). In addition, the actual IC50-values are shown as labels on the heatmap. The tested clones are presented along the Y-axis, their SSG-susceptibility status is indicated by the white (SSG-S)/grey (SSG-R) sidebars. Stress susceptibility was assessed on 3 consecutive days during stationary phase of promastigote growth; the included time-points are indicated on the X-axis. The P-value shown for each population reflects the overall similarity between the susceptibility-profiles of SSG-S and SSG-R clones within that population (repeated measures two-way ANOVA), and the asterisks further mark the specific time-points where stress-susceptibility was found to be significant different according to Bonferroni post-tests (* = P<0.05; ** = P<0.01, *** = P<0.001, **** = P<0.0001; ‡ = time-point excluded from statistical analysis due to missing values). All IC50-values ±95% CI are given in dataset S5.

The results of the stress susceptibility tests are shown in heatmaps in figure 7 which visually highlight one common finding for all 3 stresses: all clones of population A have a comparable stress-susceptibility, while in population B the SSG-R clones have a significantly different stress-susceptibility profile compared with the SSG-S clones. The susceptibility differences between SSG-S and SSG-R in population B, however, are not of the same nature and magnitude for the 3 compounds used. The difference is most pronounced for H_2_0_2_, with the tested SSG-R clones having a stable 1.6-fold lower (on average) IC50 compared with the tested SSG-S clones (P = 0.0003). The difference in SNAP susceptibility between the 2 phenotypes (P = 0.0004) varied during growth. The SNAP-tolerance of the SSG-R clones increased more steeply during stationary phase compared with the SSG-S clones. Consequently the difference between the 2 phenotypes was less significant as stationary phase progressed, and was almost non-existent by the end of stationary phase (day 7). The SbIII-susceptibility difference between the 2 phenotypes (P = 0.029) also changed during stationary phase. At the beginning of stationary phase, the SSG-R clones had a 2.3-fold lower SbIII tolerance compared with the SSG-S clones. However, the SbIII IC50 values of the SSG-S clones decreased drastically as stationary phase progressed, while those of the SSG-R clones remained approximately stable. The result is that the SSG-R clones had an average 41-fold higher SbIII tolerance compared with SSG-S clones by day 7. This SbIII tolerance difference at the end of stationary phase (when more infectious parasite forms are present) is consistent with the difference in SSG susceptibility of the subsequent intracellular amastigote life-stage. It is also of note that the steep drop in SbIII tolerance observed during stationary phase of the SSG-S clones in population B was not seen in any of the clones of population A, thus represents a significant phenotypic difference between the 2 populations (P = 0.041).

## Discussion

The primary aim of our study was to assess and attempt to correlate the molecular and phenotypic heterogeneity in parasite populations under drug treatment pressure. For this purpose we characterised a sample of *L. (L.) donovani* isolates that belong to 2 genetically distinct populations circulating in the VL endemic region of Nepal. In order to guarantee (i) reliable comparison of data collected in for numerous parasite lines and (ii) meaningful integration of data collected with various experimental approaches, it was of critical importance to use an experimental design based on highly standardised protocols. It is extremely difficult to achieve such standards when studying intracellular amastigotes, the clinical relevant form of the parasite, which need to be generated by *in vitro* macrophage infections [34]. Axenic amastigote culture techniques have been developed to overcome in part this limitation, but can only be used with parasites that are well-adapted to *in vitro* culture which is normally not the case for clinical isolates [42], [43]. Hence we chose to work with promastigotes, the form of the parasite that resides in the insect vector, which can be relatively easily and consistently cultured *in vitro*. We also chose to perform time-course analyses based on samples taken at different days during promastigote growth, an approach which we have applied previously to increase sensitivity and robustness in experimental studies of *Leishmania* [34]. Recent studies showed a dramatic down-regulation of gene expression from stationary phase promastigote to amastigotes, highlighting the functional importance of the former stage and supporting the hypothesis of a pre-adaption for intracellular life as an amastigote [44], [45], we cannot assume that the SSG-R features identified here in stationary phase promastigotes are also present in the corresponding amastigote stage. This limitation curtails confirmation of the role of the identified putative SSG-R markers in the resistance mechanisms active in amastigotes, nevertheless, as discussed below in detail, the identified promastigote characteristics provide significant new insights regarding the nature of the SSG-resistant phenotype in natural *Leishmania* populations.

### Molecular features of SSG-R population A and SSG-R population B reveal heterogeneity among SSG-R *L. (L.) donovani*

We identified in this study a number of molecular and phenotypic features that correlate with the SSG-R phenotype. However, none of those markers were found to be common for all SSG-R lines included in this study (overview in Table 4) but were specific for either population A or B.

**Table 4:** Exploration of parasite pathways known to be affected by antimonial drugs at five experimental levels.

	population A only SSG-R vs SSG-S	population B only SSG-R vs SSG-S	population A vs population B
**DNA sequence**	no differences	no differences	no differences
**gene expression**	higher levels of *CBS* and *GCS* in SSG-R	higher levels of *MST* in SSG-R	no differences
**protein expression**	no differences	lower levels of TR in SSG-R	no differences
**thiols**	no differences	no differences	higher glutathione levels in population B
**oxidative/nitrosative stress tolerance**	no differences	lower tolerance for H_2_0_2_, SNAP and SbIII in SSG-R	differential regulation of SbIII tolerance during growth

Summary of differences between (i) SSG-S parasites versus SSG-R parasites within a specific population, and (ii) all studied parasites of population A compared with population B.

The SSG-R isolates of population A were characterised by elevated mRNA abundance of CBS and GCS in comparison to the SSG-S isolates of population A (Table 3 and Figure 4), but the underlying genetic polymorphism of these mRNA differences could not be identified as SNPs in the neighbouring untranslated regions (UTRs) could not be correlated to the differential gene expression profiles between SSG-S and SSG-R strains. This does not, however, exclude the possibility for changes in trans-regulatory elements. *CBS* and *GCS* encode key enzymes for the biosynthesis of the thiols cysteine and glutathione, respectively, which confer protection against oxidant stresses (Figure 1) [27], [40], [46], [47]. However in our sample, we did not detect a concomitant increase of thiol levels (Figure 6), nor an increased oxidative/nitrosative stress tolerance of the SSG-R clones compared to the SSG-S clones of population A (Figure 7). This is in contrast to what was reported for Indian *L. (L.) donovani*, where increased *GCS* gene expression linked to increased thiol levels in the promastigote stage have been frequently described in SSG-R clinical isolates [21], [23], [27], [29]. At this point it is unclear what other role(s) increased CBS and GCS mRNA abundance could play in SSG-resistance of population A.

In population B, the SSG-R isolates were found to have a significantly up-regulated gene expression of the sulfurtransferase MST (Table 3, Figure 4). However, we could not identify the underlying genetic polymorphism (mutations in the UTR's did not correlate with SSG-susceptibility) and protein profiling demonstrated that this mRNA difference is not translated to the level of protein (Figure 5). Hence, like the enhanced *CBS*/*GCS* gene expression in population A, it is not clear what role up-regulated *MST* gene expression plays in the SSG-resistance mechanism in population B. At the protein level, the enzyme TR was found to be present in significant lower quantities in SSG-R clones compared to SSG-S clones (Figure 5). This differential protein profile presumably results from distinct post-transcriptional regulation in the 2 phenotypes since upstream TR mRNA levels of all population B isolates were similar (Table 3). A decrease of the enzyme TR signifies a decrease of an important antimonial target in the parasite, and could thus represent the basis of the SSG-resistance mechanism of population B. It is nevertheless surprising to find decreased levels of TR as part of a successful *Leishmania* adaptation since the enzyme TR has a central role in the protection against oxidants in the host [48]–[50] (Figure 1). One would expect that the observed four-fold decrease in TR protein would interfere with the parasite's capacity to manage oxidative/nitrosative stress. The stress susceptibility tests of this study indeed revealed that in population B, the tested SSG-R clones have a significant different oxidative/nitrosative stress tolerance compared to the SSG-S clones (Figure 7). The reduced TR levels and associated modified oxidative/nitrosative stress management are a prominent tandem hallmark of antimonial-resistance in population B, but not in population A where TR protein levels and likewise responses to oxidant stresses were generally conserved.

### Genetic parasite diversity is an underlying cause for molecular heterogeneity in SSG-R parasites

The distinct profiles of SSG-R parasites in the 2 studied populations provide strong evidence that the evolved molecular mechanisms leading to the SSG-resistant phenotype differ between the 2 populations (Table 4). The key question that arises is which factors drive this divergent adaptation to antimonial treatment.

Several studies hypothesised that stress factors inherent to the natural environment of *Leishmania* (*e.g.* oxidative stress from the host cell, environmental arsenic contamination) can influence the mechanisms of adaptation to antimonial pressure [6], [51], [52]. Differential exposure to such environmental stress factors theoretically could result in differential molecular changes in SSG-R parasites. However, it seems unlikely that environmental differences have been the driving force in shaping the SSG-R heterogeneity in the studied parasite sample, since the 2 studied populations originate from the same endemic region and are likely to have been exposed to similar environmental stresses.

Based on the findings of this study, we hypothesize that differences in genetic background were a major driving force in the development of heterogeneous SSG-R phenotypes. The parasite's genetic background determines the parasite's intrinsic capacity to cope with antimonial stress, and this intrinsic capacity is likely to vary to some extent between distinct genetic populations. For instance, the distinct genetic background of the 2 populations studied here is manifested as population differences in antimonial detoxifying metabolites (glutathione) and antimonial stress response (SbIII IC50, Table 4). We postulate that the success of emerging mutations to confer antimonial-resistance to a particular parasite population can depend on the specific antimonial metabolising character of that population. For instance, a particular mutation might be successful in one population and unsuccessful in another population because the intrinsic antimonial metabolism of the 2 populations is differently affected by that same mutation. Our results imply that the mutation(s) which underlie the tandem TR/oxidant stress-feature of SSG-R in population B have a beneficial effect in combination with the population B background. As this TR-related mutation has not surfaced in population A, it appears that the same beneficial effect is not attained (or, at least, not attained as readily) in combination with the population A background. Drug-resistance studies on other pathogens including *Plasmodium falciparum* and *Mycobacterium tuberculosis* also reported that the effect of specific drug-resistance mutations can depend on the strain genetic background [53], [54]. It has been proposed that this relation between genetic background and drug-resistance mutations reflects a form of epistasis. Epistasis occurs when the phenotypic effect of a mutation changes depending on the presence or absence of other mutations in the same genome [55]. In the context of drug-resistance, epistasis is thought to become manifest when a particular drug-resistance mutation has a different fitness effect depending on the strain's genetic background [56], [57].

A picture emerges in which each genetically distinct population can (and perhaps is likely to) develop a SSG-resistant phenotype with a different molecular basis. This completely transforms current perceptions of *Leishmania* drug resistance, in which it is frequently presumed that there is a single dominant molecular feature which underlies the SSG-R phenotype encountered in the field. The multitude of clinical *L.(L.) donovani* resistance markers already reported in the literature [20]–[23], [26]–[29] appeared incoherent, and sometimes incongruent (*e.g.* SSG-R markers of Indian vs Nepalese strains), but our data suggest that those findings should be re-interpreted taking into account the genetic background of the various studied parasite samples. Further studies are needed to investigate in what way the various identified SSG-R molecular features interact with the genetic background of the respective natural populations to convey the antimonial resistant phenotype. Additional isolates with a different genetic background from those of populations A and B (if any should be found) will also be considered in future studies. Epidemiological surveillance of *Leishmania* drug resistance is currently impeded by the lack of molecular-based tools to monitor the emergence and spreading of antimonial resistant parasites [2], [58], [59]. It now seems that the putative high degree of molecular heterogeneity in SSG-resistant parasites needs to be included as a key factor when designing molecular tools to monitor SSG-resistance.

## Supporting Information

Dataset S1 **Relative gene expression levels of 11 target genes in 18** ***L. (L.) donovani*** **isolates with variable SSG susceptibility during 8 consecutive days of** ***in vitro*** **promastigote growth.** The given relative gene expression levels (± SD) are the average of 3 repeated measurements in 1 experiment using the same RNA sample. (NA = result not available).
(DOC)

Dataset S2 **DNA sequence of 11 target genes in 11** ***L. (L.) donovani*** **clones with variable** ***in vitro*** **SSG susceptibility.** The coding DNA sequences (+500 basepairs upstream and downstream) of 11 proteins reported to be involved in SSG/SbV/SbIII metabolism were determined by Illumina sequencing in 11 *L. (L.) donovani* clones with differential *in vitro* SSG susceptibility.
(DOC)

Dataset S3 **Relative levels of the proteins trypanothione reductase (TR) and mercaptopyruvate sulfurtransferase of 12** ***L. (L.) donovani*** **clones with variable SSG susceptibility during 8 consecutive days of** ***in vitro*** **promastigote growth.** The given relative protein expression levels (± SEM) are the average of 2 independent samples prepared from parallel cultures which were analysed on the same Western Blot, the listed values correspond to the normalised digitally integrated intensities of the protein bands. (NA = result not available).
(DOC)

Dataset S4 **Thiol levels of 12** ***L. (L.) donovani*** **clones with variable SSG susceptibility during 8 consecutive days of** ***in vitro*** **promastigote growth.** The thiol quantities (± SEM) are the average of 3 independent samples prepared from parallel cultures which were analysed in 1 HPLC experiment, the listed values are expressed in nmol/10^8^ parasites. (NA = result not available).
(DOC)

Dataset S5 ***In vitro*** **susceptibility to H_2_0_2_, SNAP and SbIII of 12** ***L. (L.) donovani*** **clones with variable SSG susceptibility during 8 consecutive days of** ***in vitro*** **promastigote growth.** The given IC50 values [±95 CI] were determined by sigmoidal regression analysis based on the % parasite viability following 48 hrs exposure to 6 different concentrations of the tested compound. (NA = result not available).
(DOC)
