# Supplementary material for: Molecular Mechanisms of Drug Resistance in Natural Leishmania Populations Vary with Genetic Background
Source: PLoS Negl Trop Dis. 2012 Feb 28;6(2):e1514. doi: 10.1371/journal.pntd.0001514 (PMC3289598; doi:10.1371/journal.pntd.0001514)
Supplement: Dataset S1 — Relative gene expression levels of 11 target genes in 18 L. (L.) donovani isolates with variable SSG susceptibility during 8 consecutive days of in vitro promastigote growth. The given relative gene expression levels (± SD) are the average of 3 repeated measurements in 1 experiment using the same RNA sample. (NA = result not available). (DOC) [file pntd.0001514.s001.doc]

## Dataset S1.

#### Cystathionine beta-synthase (CBS)

| POPULATION A | | | | | | | | | | | | | | | | | | | | | | | | | |
| --- | --- | --- | --- | --- | --- | --- | --- | --- | --- | --- | --- | --- | --- | --- | --- | --- | --- | --- | --- | --- | --- | --- | --- | --- | --- |
| Time  point | | SSG-sensitive strains | | | | | | | | | SSG-resistant strains | | | | | | | | | | | | | | |
|  |  | BPK090/0 | | | BPK091/0 | | | BPK206/0 | | | BPK025/0 | | | BPK087/0 | | | BPK279/0 | | | BPK298/0 | | | BPK190/0 | | |
| log phase | day 1 | 1.26 | ± | 0.14 | 1.82 | ± | 0.28 | 1.75 | ± | 0.38 | 1.95 | ± | 0.15 | 2.00 | ± | 0.19 | 1.69 | ± | 0.23 | 1.57 | ± | 0.16 | 2.01 | ± | 0.32 |
|  | day 2 | 1.47 | ± | 0.16 | 1.40 | ± | 0.22 | 1.61 | ± | 0.36 | 2.07 | ± | 0.22 | 1.81 | ± | 0.19 | 1.88 | ± | 0.20 | 1.82 | ± | 0.15 | 1.88 | ± | 0.22 |
|  | day 3 | 1.36 | ± | 0.24 | 1.48 | ± | 0.18 | 1.48 | ± | 0.13 | 1.83 | ± | 0.29 | 1.83 | ± | 0.15 | 1.70 | ± | 0.20 | 1.53 | ± | 0.12 | 1.98 | ± | 0.30 |
|  | day 4 | 1.29 | ± | 0.14 | 1.44 | ± | 0.27 | 1.43 | ± | 0.20 | 1.92 | ± | 0.25 | 1.80 | ± | 0.15 | 1.65 | ± | 0.18 | 1.19 | ± | 0.24 | 1.63 | ± | 0.31 |
| stationary phase | day 5 | 1.13 | ± | 0.14 | 1.34 | ± | 0.18 | 1.27 | ± | 0.17 | 1.49 | ± | 0.16 | 1.55 | ± | 0.15 | 1.63 | ± | 0.17 | 1.11 | ± | 0.12 | 1.26 | ± | 0.18 |
|  | day 6 | 1.25 | ± | 0.23 | 1.00 | ± | 0.11 | 1.26 | ± | 0.14 | 1.66 | ± | 0.24 | 1.54 | ± | 0.17 | 1.42 | ± | 0.20 | 1.09 | ± | 0.09 | 1.43 | ± | 0.18 |
|  | day 7 | NA | | | 1.14 | ± | 0.12 | 1.20 | ± | 0.10 | 2.10 | ± | 0.22 | 1.92 | ± | 0.16 | 1.59 | ± | 0.12 | 1.41 | ± | 0.16 | 1.62 | ± | 0.26 |
|  | day 8 | 1.30 | ± | 0.32 | 1.35 | ± | 0.18 | 1.29 | ± | 0.21 | 2.04 | ± | 0.23 | 1.88 | ± | 0.16 | 1.87 | ± | 0.26 | 1.40 | ± | 0.14 | 1.61 | ± | 0.20 |

| POPULATION B | | | | | | | | | | | | | | | | | | | | | | | | | | | | | | | |
| --- | --- | --- | --- | --- | --- | --- | --- | --- | --- | --- | --- | --- | --- | --- | --- | --- | --- | --- | --- | --- | --- | --- | --- | --- | --- | --- | --- | --- | --- | --- | --- |
| Time  point | | SSG-sensitive strains | | | | | | | | | | | | | | | SSG-resistant strains | | | | | | | | | | | | | | |
|  |  | BPK282/0 | | | BPK294/0 | | | BPK035/0 | | | BPK043/0 | | | BPK181/0 | | | BPK085/0 | | | BPK164/1 | | | BPK177/0 | | | BPK178/0 | | | BPK181/12 | | |
| log phase | day 1 | 1.62 | ± | 0.18 | 1.39 | ± | 0.21 | 1.52 | ± | 0.17 | 1.38 | ± | 0.15 | 1.51 | ± | 0.19 | 1.92 | ± | 0.25 | 1.31 | ± | 0.20 | 1.75 | ± | 0.22 | 1.38 | ± | 0.12 | 2.04 | ± | 0.32 |
|  | day 2 | 1.73 | ± | 0.22 | 1.43 | ± | 0.15 | 1.48 | ± | 0.18 | 1.52 | ± | 0.21 | 1.47 | ± | 0.18 | 1.64 | ± | 0.21 | 1.43 | ± | 0.18 | 1.71 | ± | 0.21 | 1.26 | ± | 0.15 | 1.96 | ± | 0.27 |
|  | day 3 | 1.74 | ± | 0.27 | 1.37 | ± | 0.15 | 1.24 | ± | 0.16 | 1.13 | ± | 0.24 | 1.43 | ± | 0.19 | 1.44 | ± | 0.21 | 1.28 | ± | 0.14 | 1.65 | ± | 0.21 | 1.19 | ± | 0.12 | 1.89 | ± | 0.25 |
|  | day 4 | 1.59 | ± | 0.24 | 1.51 | ± | 0.28 | 1.24 | ± | 0.13 | 1.32 | ± | 0.20 | 1.34 | ± | 0.16 | 1.58 | ± | 0.17 | 1.43 | ± | 0.16 | 1.51 | ± | 0.20 | 1.11 | ± | 0.11 | 1.83 | ± | 0.28 |
| stationary phase | day 5 | 1.58 | ± | 0.25 | 1.17 | ± | 0.14 | 1.24 | ± | 0.19 | 1.10 | ± | 0.13 | 1.47 | ± | 0.23 | 1.05 | ± | 0.18 | 1.22 | ± | 0.17 | 1.06 | ± | 0.14 | 1.03 | ± | 0.20 | 1.52 | ± | 0.23 |
|  | day 6 | 1.67 | ± | 0.19 | 1.12 | ± | 0.11 | 1.02 | ± | 0.16 | 1.01 | ± | 0.11 | 1.41 | ± | 0.17 | 1.20 | ± | 0.19 | 1.17 | ± | 0.19 | 1.21 | ± | 0.13 | 1.11 | ± | 0.15 | 1.63 | ± | 0.22 |
|  | day 7 | 1.58 | ± | 0.20 | 1.19 | ± | 0.12 | 1.20 | ± | 0.23 | 1.18 | ± | 0.15 | 1.34 | ± | 0.20 | 1.36 | ± | 0.15 | 1.71 | ± | 0.27 | 1.31 | ± | 0.20 | 1.08 | ± | 0.08 | 1.63 | ± | 0.22 |
|  | day 8 | 1.67 | ± | 0.17 | 1.24 | ± | 0.21 | 1.19 | ± | 0.20 | 1.04 | ± | 0.18 | 1.41 | ± | 0.26 | 1.52 | ± | 0.26 | 1.66 | ± | 0.32 | 1.46 | ± | 0.24 | 1.20 | ± | 0.24 | 1.55 | ± | 0.23 |

#### Gamma-glutamylcysteine synthase (GCS)

| POPULATION A | | | | | | | | | | | | | | | | | | | | | | | | | |
| --- | --- | --- | --- | --- | --- | --- | --- | --- | --- | --- | --- | --- | --- | --- | --- | --- | --- | --- | --- | --- | --- | --- | --- | --- | --- |
| Time  point | | SSG-sensitive strains | | | | | | | | | SSG-resistant strains | | | | | | | | | | | | | | |
|  |  | BPK090/0 | | | BPK091/0 | | | BPK206/0 | | | BPK025/0 | | | BPK087/0 | | | BPK279/0 | | | BPK298/0 | | | BPK190/0 | | |
| log phase | day 1 | 2.48 | ± | 0.28 | 1.59 | ± | 0.20 | 1.27 | ± | 0.18 | 2.83 | ± | 0.31 | 1.58 | ± | 0.16 | 2.70 | ± | 0.40 | 1.70 | ± | 0.24 | 1.97 | ± | 0.20 |
|  | day 2 | 3.03 | ± | 0.32 | 1.51 | ± | 0.32 | 1.48 | ± | 0.22 | 2.15 | ± | 0.24 | 2.11 | ± | 0.22 | 2.76 | ± | 0.29 | 1.44 | ± | 0.20 | 2.21 | ± | 0.23 |
|  | day 3 | 2.53 | ± | 0.31 | 1.78 | ± | 0.28 | 1.43 | ± | 0.23 | 2.44 | ± | 0.42 | 1.61 | ± | 0.31 | 2.58 | ± | 0.39 | 1.21 | ± | 0.17 | 2.23 | ± | 0.48 |
|  | day 4 | 1.73 | ± | 0.27 | 1.00 | ± | 0.23 | 1.43 | ± | 0.18 | 2.63 | ± | 0.44 | 2.24 | ± | 0.20 | 2.65 | ± | 0.28 | 1.21 | ± | 0.18 | 2.77 | ± | 0.61 |
| stationary phase | day 5 | 2.46 | ± | 0.40 | 1.80 | ± | 0.23 | 2.47 | ± | 0.36 | 3.72 | ± | 0.39 | 2.98 | ± | 0.35 | 3.20 | ± | 0.42 | 3.46 | ± | 0.43 | 5.02 | ± | 0.66 |
|  | day 6 | 3.81 | ± | 0.49 | 2.19 | ± | 0.29 | 5.24 | ± | 0.74 | 9.33 | ± | 2.20 | 6.29 | ± | 1.06 | 5.22 | ± | 0.90 | 3.50 | ± | 0.44 | 7.91 | ± | 0.73 |
|  | day 7 | NA | | | 5.06 | ± | 0.72 | 6.00 | ± | 0.75 | 11.92 | ± | 1.67 | 8.83 | ± | 0.88 | 6.63 | ± | 0.73 | 10.08 | ± | 1.36 | 15.86 | ± | 1.71 |
|  | day 8 | 3.84 | ± | 0.74 | 6.68 | ± | 0.96 | 9.04 | ± | 1.57 | 11.45 | ± | 1.15 | 9.06 | ± | 0.85 | 7.14 | ± | 0.77 | 8.48 | ± | 1.11 | 13.94 | ± | 2.69 |

| POPULATION B | | | | | | | | | | | | | | | | | | | | | | | | | | | | | | | |
| --- | --- | --- | --- | --- | --- | --- | --- | --- | --- | --- | --- | --- | --- | --- | --- | --- | --- | --- | --- | --- | --- | --- | --- | --- | --- | --- | --- | --- | --- | --- | --- |
| Time  point | | SSG-sensitive strains | | | | | | | | | | | | | | | SSG-resistant strains | | | | | | | | | | | | | | |
|  |  | BPK282/0 | | | BPK294/0 | | | BPK035/0 | | | BPK043/0 | | | BPK181/0 | | | BPK085/0 | | | BPK164/1 | | | BPK177/0 | | | BPK178/0 | | | BPK181/12 | | |
| log phase | day 1 | 1.86 | ± | 0.23 | 1.82 | ± | 0.28 | 1.38 | ± | 0.16 | 2.96 | ± | 0.39 | 2.05 | ± | 0.35 | 1.75 | ± | 0.31 | 1.66 | ± | 0.28 | 1.76 | ± | 0.22 | 1.80 | ± | 0.23 | 2.06 | ± | 0.46 |
|  | day 2 | 2.30 | ± | 0.35 | 2.34 | ± | 0.36 | 1.70 | ± | 0.27 | 3.16 | ± | 0.32 | 1.96 | ± | 0.27 | 1.82 | ± | 0.26 | 1.82 | ± | 0.54 | 1.64 | ± | 0.30 | 1.60 | ± | 0.24 | 3.00 | ± | 0.52 |
|  | day 3 | 2.30 | ± | 0.43 | 1.68 | ± | 0.23 | 1.24 | ± | 0.16 | 1.88 | ± | 0.49 | 2.39 | ± | 0.39 | 1.93 | ± | 0.24 | 1.53 | ± | 0.22 | 1.50 | ± | 0.23 | 1.66 | ± | 0.23 | 3.89 | ± | 0.76 |
|  | day 4 | 2.48 | ± | 0.38 | 1.84 | ± | 0.29 | 1.32 | ± | 0.18 | 1.50 | ± | 0.17 | 2.29 | ± | 0.30 | 1.80 | ± | 0.25 | 1.70 | ± | 0.29 | 1.50 | ± | 0.23 | 1.82 | ± | 0.29 | 2.82 | ± | 0.39 |
| stationary phase | day 5 | 2.75 | ± | 0.38 | 2.19 | ± | 0.29 | 1.69 | ± | 0.22 | 2.26 | ± | 0.37 | 3.63 | ± | 0.69 | 3.37 | ± | 0.58 | 2.09 | ± | 0.28 | 2.19 | ± | 0.34 | 4.93 | ± | 0.66 | 3.86 | ± | 0.67 |
|  | day 6 | 6.92 | ± | 0.97 | 4.20 | ± | 0.59 | 4.14 | ± | 0.89 | 3.86 | ± | 0.43 | 4.75 | ± | 0.64 | 7.68 | ± | 2.06 | 4.30 | ± | 0.83 | 3.83 | ± | 0.59 | 6.71 | ± | 1.13 | 6.09 | ± | 1.12 |
|  | day 7 | 8.41 | ± | 1.14 | 4.89 | ± | 1.01 | 5.86 | ± | 0.84 | 6.98 | ± | 0.72 | 4.27 | ± | 0.63 | 7.44 | ± | 1.20 | 4.83 | ± | 0.74 | 5.66 | ± | 0.74 | 6.85 | ± | 1.11 | 7.92 | ± | 1.04 |
|  | day 8 | 10.57 | ± | 1.28 | 4.76 | ± | 0.62 | 5.93 | ± | 0.93 | 6.59 | ± | 0.77 | 6.87 | ± | 1.47 | 8.09 | ± | 1.06 | 11.97 | ± | 1.72 | 6.07 | ± | 0.87 | 9.30 | ± | 1.28 | 2.87 | ± | 0.56 |

#### Mercapto-pyruvate sulfurtransferase (MST)

| POPULATION A | | | | | | | | | | | | | | | | | | | | | | | | | |
| --- | --- | --- | --- | --- | --- | --- | --- | --- | --- | --- | --- | --- | --- | --- | --- | --- | --- | --- | --- | --- | --- | --- | --- | --- | --- |
| Time  point | | SSG-sensitive strains | | | | | | | | | SSG-resistant strains | | | | | | | | | | | | | | |
|  |  | BPK090/0 | | | BPK091/0 | | | BPK206/0 | | | BPK025/0 | | | BPK087/0 | | | BPK279/0 | | | BPK298/0 | | | BPK190/0 | | |
| log phase | day 1 | 1.28 | ± | 0.12 | 1.37 | ± | 0.19 | 1.10 | ± | 0.15 | 1.03 | ± | 0.11 | 1.29 | ± | 0.20 | 1.02 | ± | 0.12 | 1.39 | ± | 0.14 | 1.45 | ± | 0.17 |
|  | day 2 | 1.22 | ± | 0.13 | 1.25 | ± | 0.16 | 1.09 | ± | 0.16 | 1.00 | ± | 0.10 | 1.00 | ± | 0.12 | 1.11 | ± | 0.13 | 1.17 | ± | 0.16 | 1.24 | ± | 0.15 |
|  | day 3 | 1.16 | ± | 0.17 | 1.00 | ± | 0.09 | 1.01 | ± | 0.10 | 1.01 | ± | 0.15 | 1.08 | ± | 0.19 | 1.00 | ± | 0.16 | 1.13 | ± | 0.15 | 1.35 | ± | 0.18 |
|  | day 4 | 1.00 | ± | 0.10 | 1.41 | ± | 0.17 | 1.03 | ± | 0.10 | 1.16 | ± | 0.17 | 1.10 | ± | 0.14 | 1.13 | ± | 0.12 | 1.00 | ± | 0.09 | 1.43 | ± | 0.22 |
| stationary phase | day 5 | 1.18 | ± | 0.13 | 1.47 | ± | 0.21 | 1.00 | ± | 0.14 | 1.25 | ± | 0.13 | 1.24 | ± | 0.15 | 1.33 | ± | 0.16 | 1.25 | ± | 0.11 | 1.00 | ± | 0.21 |
|  | day 6 | 1.56 | ± | 0.21 | 1.35 | ± | 0.10 | 1.20 | ± | 0.12 | 1.73 | ± | 0.27 | 1.43 | ± | 0.18 | 1.02 | ± | 0.15 | 1.83 | ± | 0.17 | 1.31 | ± | 0.19 |
|  | day 7 | NA | | | 1.83 | ± | 0.15 | 1.98 | ± | 0.27 | 1.49 | ± | 0.14 | 1.88 | ± | 0.30 | 1.24 | ± | 0.15 | 1.60 | ± | 0.17 | 1.29 | ± | 0.22 |
|  | day 8 | 2.60 | ± | 0.46 | 2.32 | ± | 0.27 | 2.05 | ± | 0.29 | 1.49 | ± | 0.16 | 2.67 | ± | 0.33 | 1.72 | ± | 0.31 | 1.43 | ± | 0.20 | 2.73 | ± | 0.40 |

| POPULATION B | | | | | | | | | | | | | | | | | | | | | | | | | | | | | | | |
| --- | --- | --- | --- | --- | --- | --- | --- | --- | --- | --- | --- | --- | --- | --- | --- | --- | --- | --- | --- | --- | --- | --- | --- | --- | --- | --- | --- | --- | --- | --- | --- |
| Time  point | | SSG-sensitive strains | | | | | | | | | | | | | | | SSG-resistant strains | | | | | | | | | | | | | | |
|  |  | BPK282/0 | | | BPK294/0 | | | BPK035/0 | | | BPK043/0 | | | BPK181/0 | | | BPK085/0 | | | BPK164/1 | | | BPK177/0 | | | BPK178/0 | | | BPK181/12 | | |
| log phase | day 1 | 1.27 | ± | 0.15 | 1.21 | ± | 0.25 | 1.37 | ± | 0.20 | 1.46 | ± | 0.17 | 1.00 | ± | 0.07 | 1.65 | ± | 0.21 | 1.73 | ± | 0.36 | 1.68 | ± | 0.20 | 1.90 | ± | 0.18 | 1.26 | ± | 0.19 |
|  | day 2 | 1.30 | ± | 0.17 | 1.09 | ± | 0.14 | 1.08 | ± | 0.11 | 1.30 | ± | 0.15 | 1.00 | ± | 0.13 | 1.15 | ± | 0.14 | 1.66 | ± | 0.21 | 1.41 | ± | 0.17 | 1.28 | ± | 0.15 | 1.39 | ± | 0.23 |
|  | day 3 | 1.24 | ± | 0.20 | 1.17 | ± | 0.14 | 1.11 | ± | 0.10 | 1.43 | ± | 0.24 | 1.21 | ± | 0.15 | 1.10 | ± | 0.17 | 1.12 | ± | 0.15 | 1.34 | ± | 0.14 | 1.19 | ± | 0.11 | 1.25 | ± | 0.20 |
|  | day 4 | 1.20 | ± | 0.19 | 1.00 | ± | 0.17 | 1.00 | ± | 0.11 | 1.23 | ± | 0.16 | 1.21 | ± | 0.13 | 1.00 | ± | 0.15 | 1.00 | ± | 0.12 | 1.11 | ± | 0.15 | 1.00 | ± | 0.15 | 1.03 | ± | 0.19 |
| stationary phase | day 5 | 1.01 | ± | 0.12 | 1.00 | ± | 0.16 | 1.10 | ± | 0.12 | 1.16 | ± | 0.12 | 1.24 | ± | 0.14 | 1.08 | ± | 0.22 | 1.11 | ± | 0.16 | 1.00 | ± | 0.08 | 1.59 | ± | 0.17 | 1.03 | ± | 0.13 |
|  | day 6 | 1.00 | ± | 0.12 | 1.07 | ± | 0.12 | 1.38 | ± | 0.19 | 1.16 | ± | 0.12 | 1.21 | ± | 0.15 | 1.59 | ± | 0.19 | 1.32 | ± | 0.32 | 1.45 | ± | 0.13 | 1.82 | ± | 0.22 | 1.00 | ± | 0.13 |
|  | day 7 | 1.59 | ± | 0.19 | 1.20 | ± | 0.22 | 1.72 | ± | 0.25 | 1.00 | ± | 0.13 | 1.17 | ± | 0.18 | 2.64 | ± | 0.49 | 3.08 | ± | 0.39 | 2.32 | ± | 0.32 | 2.41 | ± | 0.40 | 1.56 | ± | 0.21 |
|  | day 8 | 1.89 | ± | 0.24 | 1.35 | ± | 0.20 | 1.90 | ± | 0.39 | 1.15 | ± | 0.16 | 1.21 | ± | 0.18 | 2.86 | ± | 0.51 | 2.89 | ± | 0.58 | 1.84 | ± | 0.17 | 1.44 | ± | 0.21 | 5.28 | ± | 0.89 |

#### Trypanothione reductase (TR)

| POPULATION A | | | | | | | | | | | | | | | | | | | | | | | | | |
| --- | --- | --- | --- | --- | --- | --- | --- | --- | --- | --- | --- | --- | --- | --- | --- | --- | --- | --- | --- | --- | --- | --- | --- | --- | --- |
| Time  point | | SSG-sensitive strains | | | | | | | | | SSG-resistant strains | | | | | | | | | | | | | | |
|  |  | BPK090/0 | | | BPK091/0 | | | BPK206/0 | | | BPK025/0 | | | BPK087/0 | | | BPK279/0 | | | BPK298/0 | | | BPK190/0 | | |
| log phase | day 1 | 1.37 | ± | 0.14 | 1.06 | ± | 0.12 | 1.65 | ± | 0.20 | 2.73 | ± | 0.21 | 1.51 | ± | 0.17 | 1.87 | ± | 0.23 | 2.44 | ± | 0.41 | 1.99 | ± | 0.23 |
|  | day 2 | 1.47 | ± | 0.12 | 1.18 | ± | 0.21 | 2.04 | ± | 0.36 | 2.46 | ± | 0.34 | 1.74 | ± | 0.28 | 2.30 | ± | 0.21 | 2.04 | ± | 0.35 | 2.35 | ± | 0.20 |
|  | day 3 | 1.24 | ± | 0.11 | 1.25 | ± | 0.15 | 1.97 | ± | 0.24 | 2.49 | ± | 0.28 | 1.56 | ± | 0.30 | 1.87 | ± | 0.23 | 1.81 | ± | 0.25 | 2.63 | ± | 0.23 |
|  | day 4 | 1.00 | ± | 0.09 | 1.22 | ± | 0.16 | 1.79 | ± | 0.21 | 2.16 | ± | 0.19 | 1.55 | ± | 0.13 | 1.78 | ± | 0.29 | 2.11 | ± | 0.33 | 3.34 | ± | 0.43 |
| stationary phase | day 5 | 1.29 | ± | 0.11 | 1.27 | ± | 0.13 | 2.35 | ± | 0.49 | 3.46 | ± | 0.38 | 2.55 | ± | 0.20 | 2.05 | ± | 0.27 | 3.21 | ± | 0.39 | 3.55 | ± | 0.42 |
|  | day 6 | 2.16 | ± | 0.28 | 1.90 | ± | 0.18 | 3.31 | ± | 0.35 | 3.53 | ± | 0.47 | 3.07 | ± | 0.26 | 2.97 | ± | 0.39 | 3.31 | ± | 0.30 | 2.93 | ± | 0.31 |
|  | day 7 | NA | | | 2.19 | ± | 0.25 | 4.35 | ± | 0.72 | 5.18 | ± | 0.51 | 3.36 | ± | 0.44 | 3.25 | ± | 0.25 | 4.34 | ± | 0.49 | 4.49 | ± | 0.59 |
|  | day 8 | 1.88 | ± | 0.35 | 1.83 | ± | 0.22 | 4.41 | ± | 0.66 | 4.63 | ± | 0.65 | 2.49 | ± | 0.19 | 3.68 | ± | 0.38 | 3.22 | ± | 0.56 | 3.84 | ± | 0.44 |

| POPULATION B | | | | | | | | | | | | | | | | | | | | | | | | | | | | | | | |
| --- | --- | --- | --- | --- | --- | --- | --- | --- | --- | --- | --- | --- | --- | --- | --- | --- | --- | --- | --- | --- | --- | --- | --- | --- | --- | --- | --- | --- | --- | --- | --- |
| Time  point | | SSG-sensitive strains | | | | | | | | | | | | | | | SSG-resistant strains | | | | | | | | | | | | | | |
|  |  | BPK282/0 | | | BPK294/0 | | | BPK035/0 | | | BPK043/0 | | | BPK181/0 | | | BPK085/0 | | | BPK164/1 | | | BPK177/0 | | | BPK178/0 | | | BPK181/12 | | |
| log phase | day 1 | 1.64 | ± | 0.21 | 1.61 | ± | 0.28 | 1.42 | ± | 0.16 | 1.81 | ± | 0.30 | 1.11 | ± | 0.15 | 1.65 | ± | 0.18 | 1.26 | ± | 0.11 | 1.39 | ± | 0.15 | 2.27 | ± | 0.32 | 1.20 | ± | 0.20 |
|  | day 2 | 1.76 | ± | 0.25 | 1.74 | ± | 0.19 | 1.86 | ± | 0.23 | 2.62 | ± | 0.36 | 1.40 | ± | 0.13 | 2.10 | ± | 0.32 | 1.36 | ± | 0.11 | 1.50 | ± | 0.16 | 1.87 | ± | 0.23 | 1.47 | ± | 0.15 |
|  | day 3 | 2.10 | ± | 0.38 | 1.59 | ± | 0.17 | 1.66 | ± | 0.20 | 1.77 | ± | 0.42 | 1.54 | ± | 0.14 | 1.96 | ± | 0.25 | 1.67 | ± | 0.19 | 1.67 | ± | 0.16 | 2.25 | ± | 0.26 | 1.56 | ± | 0.18 |
|  | day 4 | 1.69 | ± | 0.31 | 1.26 | ± | 0.20 | 1.61 | ± | 0.19 | 1.68 | ± | 0.24 | 1.29 | ± | 0.14 | 1.81 | ± | 0.21 | 1.39 | ± | 0.23 | 1.46 | ± | 0.17 | 2.18 | ± | 0.22 | 1.37 | ± | 0.17 |
| stationary phase | day 5 | 2.16 | ± | 0.25 | 1.33 | ± | 0.20 | 1.44 | ± | 0.20 | 2.19 | ± | 0.22 | 1.53 | ± | 0.28 | 2.39 | ± | 0.43 | 1.86 | ± | 0.16 | 1.47 | ± | 0.17 | 2.69 | ± | 0.55 | 1.63 | ± | 0.18 |
|  | day 6 | 3.32 | ± | 0.41 | 1.96 | ± | 0.19 | 2.93 | ± | 0.45 | 3.15 | ± | 0.27 | 1.69 | ± | 0.18 | 3.58 | ± | 0.50 | 2.45 | ± | 0.26 | 2.03 | ± | 0.34 | 3.62 | ± | 0.62 | 1.76 | ± | 0.17 |
|  | day 7 | 2.95 | ± | 0.38 | 2.10 | ± | 0.27 | 3.25 | ± | 0.58 | 3.21 | ± | 0.35 | 1.45 | ± | 0.15 | 4.62 | ± | 0.69 | 2.92 | ± | 0.33 | 2.11 | ± | 0.37 | 3.66 | ± | 0.40 | 2.45 | ± | 0.21 |
|  | day 8 | 2.61 | ± | 0.52 | 2.11 | ± | 0.34 | 3.27 | ± | 0.43 | 3.13 | ± | 0.42 | 1.50 | ± | 0.26 | 4.72 | ± | 0.56 | 1.45 | ± | 0.20 | 1.87 | ± | 0.19 | 4.03 | ± | 0.72 | 2.15 | ± | 0.32 |

#### Arsenate reductase 2 (ACR2)

| POPULATION A | | | | | | | | | | | | | | | | | | | | | | | | | |
| --- | --- | --- | --- | --- | --- | --- | --- | --- | --- | --- | --- | --- | --- | --- | --- | --- | --- | --- | --- | --- | --- | --- | --- | --- | --- |
| Time  point | | SSG-sensitive strains | | | | | | | | | SSG-resistant strains | | | | | | | | | | | | | | |
|  |  | BPK090/0 | | | BPK091/0 | | | BPK206/0 | | | BPK025/0 | | | BPK087/0 | | | BPK279/0 | | | BPK298/0 | | | BPK190/0 | | |
| log phase | day 1 | 2.11 | ± | 0.22 | 2.15 | ± | 0.27 | 1.44 | ± | 0.16 | 1.76 | ± | 0.19 | 1.10 | ± | 0.15 | 1.74 | ± | 0.19 | 1.45 | ± | 0.17 | 1.76 | ± | 0.19 |
|  | day 2 | 2.16 | ± | 0.23 | 2.09 | ± | 0.29 | 1.43 | ± | 0.17 | 1.51 | ± | 0.20 | 1.36 | ± | 0.11 | 1.73 | ± | 0.32 | 1.60 | ± | 0.12 | 1.99 | ± | 0.27 |
|  | day 3 | 2.11 | ± | 0.33 | 1.88 | ± | 0.25 | 1.55 | ± | 0.12 | 1.85 | ± | 0.26 | 1.22 | ± | 0.20 | 1.86 | ± | 0.23 | 1.52 | ± | 0.11 | 1.93 | ± | 0.30 |
|  | day 4 | 1.90 | ± | 0.28 | 1.70 | ± | 0.30 | 1.25 | ± | 0.14 | 1.92 | ± | 0.32 | 1.35 | ± | 0.14 | 1.78 | ± | 0.29 | 1.34 | ± | 0.17 | 1.99 | ± | 0.44 |
| stationary phase | day 5 | 1.91 | ± | 0.19 | 1.70 | ± | 0.22 | 1.49 | ± | 0.24 | 2.49 | ± | 0.29 | 1.63 | ± | 0.19 | 1.70 | ± | 0.16 | 1.52 | ± | 0.12 | 2.32 | ± | 0.31 |
|  | day 6 | 2.26 | ± | 0.28 | 2.47 | ± | 0.23 | 1.67 | ± | 0.11 | 2.02 | ± | 0.20 | 1.30 | ± | 0.18 | 1.71 | ± | 0.25 | 1.69 | ± | 0.12 | 1.96 | ± | 0.22 |
|  | day 7 | NA | | | 2.12 | ± | 0.22 | 1.65 | ± | 0.13 | 1.70 | ± | 0.15 | 1.12 | ± | 0.16 | 1.47 | ± | 0.16 | 1.11 | ± | 0.19 | 1.75 | ± | 0.31 |
|  | day 8 | 2.77 | ± | 0.57 | 2.08 | ± | 0.31 | 1.43 | ± | 0.23 | 1.59 | ± | 0.18 | 1.52 | ± | 0.15 | 1.44 | ± | 0.17 | 1.23 | ± | 0.13 | 1.50 | ± | 0.22 |

| POPULATION B | | | | | | | | | | | | | | | | | | | | | | | | | | | | | | | |
| --- | --- | --- | --- | --- | --- | --- | --- | --- | --- | --- | --- | --- | --- | --- | --- | --- | --- | --- | --- | --- | --- | --- | --- | --- | --- | --- | --- | --- | --- | --- | --- |
| Time  point | | SSG-sensitive strains | | | | | | | | | | | | | | | SSG-resistant strains | | | | | | | | | | | | | | |
|  |  | BPK282/0 | | | BPK294/0 | | | BPK035/0 | | | BPK043/0 | | | BPK181/0 | | | BPK085/0 | | | BPK164/1 | | | BPK177/0 | | | BPK178/0 | | | BPK181/12 | | |
| log phase | day 1 | 1.47 | ± | 0.22 | 1.41 | ± | 0.19 | 2.03 | ± | 0.19 | 2.44 | ± | 0.30 | 1.00 | ± | 0.12 | 1.27 | ± | 0.14 | 1.44 | ± | 0.17 | 1.37 | ± | 0.16 | 1.51 | ± | 0.12 | 1.46 | ± | 0.22 |
|  | day 2 | 1.38 | ± | 0.19 | 1.43 | ± | 0.16 | 1.84 | ± | 0.28 | 2.18 | ± | 0.33 | 1.20 | ± | 0.15 | 1.25 | ± | 0.16 | 1.38 | ± | 0.14 | 1.24 | ± | 0.14 | 1.28 | ± | 0.18 | 1.40 | ± | 0.15 |
|  | day 3 | 1.31 | ± | 0.19 | 1.43 | ± | 0.22 | 1.93 | ± | 0.20 | 2.29 | ± | 0.52 | 1.06 | ± | 0.13 | 1.40 | ± | 0.19 | 1.48 | ± | 0.17 | 1.51 | ± | 0.18 | 1.53 | ± | 0.15 | 1.21 | ± | 0.25 |
|  | day 4 | 1.21 | ± | 0.19 | 1.37 | ± | 0.16 | 1.74 | ± | 0.22 | 2.12 | ± | 0.24 | 1.19 | ± | 0.14 | 1.28 | ± | 0.19 | 1.29 | ± | 0.13 | 1.28 | ± | 0.15 | 1.35 | ± | 0.14 | 1.58 | ± | 0.20 |
| stationary phase | day 5 | 1.20 | ± | 0.15 | 1.41 | ± | 0.16 | 1.63 | ± | 0.20 | 2.34 | ± | 0.43 | 1.24 | ± | 0.18 | 1.48 | ± | 0.30 | 1.43 | ± | 0.14 | 1.39 | ± | 0.14 | 1.45 | ± | 0.16 | 1.39 | ± | 0.20 |
|  | day 6 | 1.27 | ± | 0.15 | 1.57 | ± | 0.20 | 2.46 | ± | 0.27 | 2.80 | ± | 0.30 | 1.28 | ± | 0.15 | 1.40 | ± | 0.16 | 1.58 | ± | 0.28 | 1.45 | ± | 0.14 | 1.45 | ± | 0.16 | 1.49 | ± | 0.18 |
|  | day 7 | 1.45 | ± | 0.19 | 1.36 | ± | 0.20 | 2.32 | ± | 0.26 | 2.78 | ± | 0.35 | 1.17 | ± | 0.18 | 1.57 | ± | 0.21 | 1.35 | ± | 0.17 | 1.19 | ± | 0.20 | 1.32 | ± | 0.17 | 1.47 | ± | 0.22 |
|  | day 8 | 1.47 | ± | 0.19 | 1.46 | ± | 0.19 | 2.28 | ± | 0.33 | 3.02 | ± | 0.43 | 1.39 | ± | 0.18 | 1.24 | ± | 0.15 | 1.14 | ± | 0.18 | 1.46 | ± | 0.16 | 1.57 | ± | 0.19 | 1.48 | ± | 0.23 |

#### Aquaglyceroporin 1 (AQP1)

| POPULATION A | | | | | | | | | | | | | | | | | | | | | | | | | |
| --- | --- | --- | --- | --- | --- | --- | --- | --- | --- | --- | --- | --- | --- | --- | --- | --- | --- | --- | --- | --- | --- | --- | --- | --- | --- |
| Time  point | | SSG-sensitive strains | | | | | | | | | SSG-resistant strains | | | | | | | | | | | | | | |
|  |  | BPK090/0 | | | BPK091/0 | | | BPK206/0 | | | BPK025/0 | | | BPK087/0 | | | BPK279/0 | | | BPK298/0 | | | BPK190/0 | | |
| log phase | day 1 | 3.37 | ± | 0.34 | 2.75 | ± | 0.34 | 2.35 | ± | 0.36 | 2.76 | ± | 0.30 | 1.50 | ± | 0.20 | 2.13 | ± | 0.24 | 2.78 | ± | 0.29 | 2.45 | ± | 0.42 |
|  | day 2 | 2.16 | ± | 0.23 | 3.01 | ± | 0.46 | 2.77 | ± | 0.40 | 2.05 | ± | 0.30 | 1.90 | ± | 0.33 | 1.84 | ± | 0.23 | 3.45 | ± | 0.30 | 2.88 | ± | 0.48 |
|  | day 3 | 2.30 | ± | 0.34 | 2.91 | ± | 0.35 | 2.45 | ± | 0.32 | 1.49 | ± | 0.22 | 1.57 | ± | 0.27 | 1.13 | ± | 0.14 | 1.55 | ± | 0.16 | 2.22 | ± | 0.26 |
|  | day 4 | 1.64 | ± | 0.30 | 1.79 | ± | 0.26 | 1.23 | ± | 0.16 | 1.40 | ± | 0.20 | 1.64 | ± | 0.19 | 1.05 | ± | 0.13 | 1.68 | ± | 0.18 | 1.48 | ± | 0.24 |
| stationary phase | day 5 | 2.25 | ± | 0.31 | 1.38 | ± | 0.15 | 2.43 | ± | 0.33 | 1.57 | ± | 0.20 | 2.84 | ± | 0.30 | 2.13 | ± | 0.31 | 3.63 | ± | 0.40 | 4.00 | ± | 0.68 |
|  | day 6 | 3.10 | ± | 0.56 | 2.63 | ± | 0.25 | 6.00 | ± | 0.69 | 7.61 | ± | 1.15 | 4.90 | ± | 0.73 | 3.51 | ± | 0.49 | 6.00 | ± | 0.66 | 9.41 | ± | 1.33 |
|  | day 7 | NA | | | 4.44 | ± | 0.46 | 8.59 | ± | 0.88 | 13.56 | ± | 2.07 | 7.04 | ± | 0.93 | 4.23 | ± | 0.61 | 13.21 | ± | 2.54 | 28.18 | ± | 3.81 |
|  | day 8 | 6.53 | ± | 1.22 | 5.86 | ± | 0.62 | 10.14 | ± | 1.17 | 19.90 | ± | 3.57 | 10.55 | ± | 1.29 | 6.80 | ± | 0.79 | 12.70 | ± | 1.36 | 28.30 | ± | 5.70 |

| POPULATION B | | | | | | | | | | | | | | | | | | | | | | | | | | | | | | | |
| --- | --- | --- | --- | --- | --- | --- | --- | --- | --- | --- | --- | --- | --- | --- | --- | --- | --- | --- | --- | --- | --- | --- | --- | --- | --- | --- | --- | --- | --- | --- | --- |
| Time  point | | SSG-sensitive strains | | | | | | | | | | | | | | | SSG-resistant strains | | | | | | | | | | | | | | |
|  |  | BPK282/0 | | | BPK294/0 | | | BPK035/0 | | | BPK043/0 | | | BPK181/0 | | | BPK085/0 | | | BPK164/1 | | | BPK177/0 | | | BPK178/0 | | | BPK181/12 | | |
| log phase | day 1 | 2.67 | ± | 0.37 | 2.09 | ± | 0.29 | 1.24 | ± | 0.12 | 2.58 | ± | 0.35 | 1.54 | ± | 0.25 | 1.72 | ± | 0.24 | 1.09 | ± | 0.13 | 1.51 | ± | 0.22 | 2.26 | ± | 0.21 | 2.41 | ± | 0.27 |
|  | day 2 | 3.54 | ± | 0.60 | 2.52 | ± | 0.34 | 2.33 | ± | 0.25 | 2.46 | ± | 0.38 | 1.64 | ± | 0.23 | 1.96 | ± | 0.20 | 2.13 | ± | 0.17 | 1.97 | ± | 0.21 | 1.71 | ± | 0.17 | 2.45 | ± | 0.47 |
|  | day 3 | 2.32 | ± | 0.30 | 1.58 | ± | 0.15 | 1.77 | ± | 0.21 | 2.50 | ± | 0.54 | 2.00 | ± | 0.23 | 1.86 | ± | 0.17 | 1.00 | ± | 0.17 | 1.88 | ± | 0.21 | 1.59 | ± | 0.22 | 2.44 | ± | 0.34 |
|  | day 4 | 2.12 | ± | 0.37 | 1.24 | ± | 0.21 | 1.24 | ± | 0.11 | 1.09 | ± | 0.17 | 1.44 | ± | 0.20 | 1.52 | ± | 0.15 | 1.42 | ± | 0.17 | 1.28 | ± | 0.13 | 1.39 | ± | 0.14 | 2.67 | ± | 0.36 |
| stationary phase | day 5 | 1.94 | ± | 0.27 | 1.73 | ± | 0.21 | 1.88 | ± | 0.22 | 1.47 | ± | 0.21 | 1.66 | ± | 0.27 | 1.99 | ± | 0.28 | 1.34 | ± | 0.22 | 1.76 | ± | 0.21 | 3.03 | ± | 0.50 | 2.58 | ± | 0.24 |
|  | day 6 | 3.27 | ± | 0.36 | 2.32 | ± | 0.22 | 3.40 | ± | 0.51 | 2.88 | ± | 0.29 | 2.04 | ± | 0.38 | 4.53 | ± | 0.58 | 4.30 | ± | 0.57 | 2.87 | ± | 0.26 | 7.04 | ± | 0.99 | 5.10 | ± | 0.42 |
|  | day 7 | 10.35 | ± | 1.27 | 3.02 | ± | 0.50 | 8.40 | ± | 1.28 | 13.10 | ± | 2.34 | 1.81 | ± | 0.23 | 9.57 | ± | 1.39 | 6.03 | ± | 0.72 | 5.14 | ± | 0.78 | 6.58 | ± | 0.76 | 4.15 | ± | 0.46 |
|  | day 8 | 11.90 | ± | 1.64 | 5.41 | ± | 0.57 | 13.26 | ± | 1.57 | 12.93 | ± | 1.69 | 3.68 | ± | 0.51 | 9.21 | ± | 1.19 | 12.50 | ± | 1.49 | 11.35 | ± | 1.69 | 14.09 | ± | 2.10 | 8.16 | ± | 1.34 |

#### Cysteine synthase (CS)

| POPULATION A | | | | | | | | | | | | | | | | | | | | | | | | | |
| --- | --- | --- | --- | --- | --- | --- | --- | --- | --- | --- | --- | --- | --- | --- | --- | --- | --- | --- | --- | --- | --- | --- | --- | --- | --- |
| Time  point | | SSG-sensitive strains | | | | | | | | | SSG-resistant strains | | | | | | | | | | | | | | |
|  |  | BPK090/0 | | | BPK091/0 | | | BPK206/0 | | | BPK025/0 | | | BPK087/0 | | | BPK279/0 | | | BPK298/0 | | | BPK190/0 | | |
| log phase | day 1 | 1.45 | ± | 0.16 | 1.13 | ± | 0.09 | 1.78 | ± | 0.19 | 1.50 | ± | 0.13 | 1.39 | ± | 0.23 | 1.62 | ± | 0.14 | 1.47 | ± | 0.14 | 1.53 | ± | 0.14 |
|  | day 2 | 1.46 | ± | 0.13 | 1.31 | ± | 0.23 | 1.99 | ± | 0.22 | 1.56 | ± | 0.14 | 1.43 | ± | 0.13 | 1.63 | ± | 0.19 | 1.35 | ± | 0.08 | 1.65 | ± | 0.28 |
|  | day 3 | 1.70 | ± | 0.29 | 1.39 | ± | 0.17 | 2.01 | ± | 0.18 | 1.81 | ± | 0.20 | 1.57 | ± | 0.15 | 1.65 | ± | 0.16 | 1.50 | ± | 0.08 | 1.88 | ± | 0.22 |
|  | day 4 | 1.81 | ± | 0.16 | 1.61 | ± | 0.18 | 2.41 | ± | 0.25 | 1.72 | ± | 0.25 | 1.71 | ± | 0.24 | 1.71 | ± | 0.19 | 1.96 | ± | 0.16 | 1.99 | ± | 0.22 |
| stationary phase | day 5 | 1.77 | ± | 0.15 | 1.59 | ± | 0.18 | 2.35 | ± | 0.18 | 1.86 | ± | 0.25 | 1.77 | ± | 0.15 | 2.11 | ± | 0.31 | 1.65 | ± | 0.12 | 2.17 | ± | 0.33 |
|  | day 6 | 1.70 | ± | 0.29 | 1.49 | ± | 0.11 | 2.18 | ± | 0.22 | 1.68 | ± | 0.29 | 1.66 | ± | 0.22 | 1.84 | ± | 0.23 | 1.64 | ± | 0.13 | 1.79 | ± | 0.24 |
|  | day 7 | NA | | | 1.50 | ± | 0.13 | 2.07 | ± | 0.15 | 1.43 | ± | 0.16 | 1.50 | ± | 0.14 | 1.92 | ± | 0.16 | 2.00 | ± | 0.19 | 1.69 | ± | 0.32 |
|  | day 8 | 1.29 | ± | 0.26 | 1.34 | ± | 0.18 | 2.20 | ± | 0.23 | 1.50 | ± | 0.21 | 1.31 | ± | 0.13 | 1.71 | ± | 0.25 | 1.53 | ± | 0.19 | 1.51 | ± | 0.27 |

| POPULATION B | | | | | | | | | | | | | | | | | | | | | | | | | | | | | | | |
| --- | --- | --- | --- | --- | --- | --- | --- | --- | --- | --- | --- | --- | --- | --- | --- | --- | --- | --- | --- | --- | --- | --- | --- | --- | --- | --- | --- | --- | --- | --- | --- |
| Time  point | | SSG-sensitive strains | | | | | | | | | | | | | | | SSG-resistant strains | | | | | | | | | | | | | | |
|  |  | BPK282/0 | | | BPK294/0 | | | BPK035/0 | | | BPK043/0 | | | BPK181/0 | | | BPK085/0 | | | BPK164/1 | | | BPK177/0 | | | BPK178/0 | | | BPK181/12 | | |
| log phase | day 1 | 1.33 | ± | 0.12 | 1.55 | ± | 0.14 | 1.27 | ± | 0.12 | 1.64 | ± | 0.13 | 2.76 | ± | 0.18 | 1.36 | ± | 0.18 | 1.50 | ± | 0.15 | 1.20 | ± | 0.22 | 1.72 | ± | 0.23 | 1.49 | ± | 0.15 |
|  | day 2 | 1.34 | ± | 0.14 | 1.50 | ± | 0.16 | 1.27 | ± | 0.15 | 1.46 | ± | 0.20 | 2.47 | ± | 0.31 | 1.76 | ± | 0.21 | 1.70 | ± | 0.12 | 1.42 | ± | 0.15 | 2.01 | ± | 0.17 | 1.77 | ± | 0.22 |
|  | day 3 | 1.31 | ± | 0.20 | 1.72 | ± | 0.16 | 1.43 | ± | 0.13 | 1.68 | ± | 0.31 | 2.53 | ± | 0.23 | 1.76 | ± | 0.17 | 1.85 | ± | 0.23 | 1.58 | ± | 0.13 | 2.09 | ± | 0.26 | 1.92 | ± | 0.32 |
|  | day 4 | 1.45 | ± | 0.24 | 1.85 | ± | 0.18 | 1.63 | ± | 0.22 | 1.83 | ± | 0.20 | 2.67 | ± | 0.37 | 1.81 | ± | 0.17 | 1.91 | ± | 0.14 | 1.66 | ± | 0.18 | 2.44 | ± | 0.17 | 1.94 | ± | 0.19 |
| stationary phase | day 5 | 1.62 | ± | 0.14 | 2.03 | ± | 0.28 | 1.71 | ± | 0.19 | 1.80 | ± | 0.33 | 2.53 | ± | 0.28 | 2.20 | ± | 0.32 | 2.02 | ± | 0.12 | 1.84 | ± | 0.22 | 2.08 | ± | 0.30 | 2.05 | ± | 0.22 |
|  | day 6 | 1.49 | ± | 0.21 | 1.89 | ± | 0.17 | 1.55 | ± | 0.17 | 1.79 | ± | 0.14 | 2.60 | ± | 0.21 | 1.89 | ± | 0.22 | 1.92 | ± | 0.18 | 1.60 | ± | 0.14 | 1.98 | ± | 0.16 | 1.97 | ± | 0.15 |
|  | day 7 | 1.30 | ± | 0.11 | 2.12 | ± | 0.28 | 1.11 | ± | 0.13 | 1.32 | ± | 0.15 | 2.81 | ± | 0.48 | 1.54 | ± | 0.22 | 1.47 | ± | 0.19 | 1.69 | ± | 0.20 | 1.65 | ± | 0.12 | 1.69 | ± | 0.24 |
|  | day 8 | 1.35 | ± | 0.12 | 1.85 | ± | 0.28 | 1.20 | ± | 0.11 | 1.00 | ± | 0.19 | 2.65 | ± | 0.38 | 1.55 | ± | 0.21 | 1.35 | ± | 0.27 | 1.17 | ± | 0.16 | 1.56 | ± | 0.19 | 1.33 | ± | 0.19 |

#### Ornithine decarboxylase (ODC)

| POPULATION A | | | | | | | | | | | | | | | | | | | | | | | | | |
| --- | --- | --- | --- | --- | --- | --- | --- | --- | --- | --- | --- | --- | --- | --- | --- | --- | --- | --- | --- | --- | --- | --- | --- | --- | --- |
| Time  point | | SSG-sensitive strains | | | | | | | | | SSG-resistant strains | | | | | | | | | | | | | | |
|  |  | BPK090/0 | | | BPK091/0 | | | BPK206/0 | | | BPK025/0 | | | BPK087/0 | | | BPK279/0 | | | BPK298/0 | | | BPK190/0 | | |
| log phase | day 1 | 1.32 | ± | 0.35 | 3.05 | ± | 0.35 | 2.77 | ± | 0.49 | 3.27 | ± | 0.43 | 3.51 | ± | 0.50 | 1.74 | ± | 0.26 | 2.32 | ± | 0.41 | 2.30 | ± | 0.40 |
|  | day 2 | 1.25 | ± | 0.26 | 2.87 | ± | 0.47 | 2.32 | ± | 0.49 | 4.65 | ± | 0.52 | 3.41 | ± | 0.40 | 1.60 | ± | 0.17 | 2.00 | ± | 0.30 | 2.58 | ± | 0.49 |
|  | day 3 | 1.66 | ± | 0.32 | 2.12 | ± | 0.28 | 2.57 | ± | 0.42 | 5.92 | ± | 0.71 | 3.84 | ± | 0.55 | 2.16 | ± | 0.34 | 2.71 | ± | 0.40 | 3.10 | ± | 0.78 |
|  | day 4 | 2.12 | ± | 0.39 | 2.97 | ± | 0.54 | 2.43 | ± | 0.37 | 6.50 | ± | 0.76 | 3.39 | ± | 0.39 | 3.09 | ± | 0.30 | 2.30 | ± | 0.39 | 2.24 | ± | 0.51 |
| stationary phase | day 5 | 2.43 | ± | 0.43 | 2.83 | ± | 0.38 | 2.63 | ± | 0.50 | 3.36 | ± | 0.46 | 2.54 | ± | 0.41 | 3.93 | ± | 0.53 | 2.88 | ± | 0.47 | 2.38 | ± | 0.51 |
|  | day 6 | 2.05 | ± | 0.57 | 2.47 | ± | 0.27 | 2.04 | ± | 0.37 | 3.83 | ± | 0.51 | 3.31 | ± | 0.32 | 2.58 | ± | 0.41 | 2.06 | ± | 0.37 | 1.88 | ± | 0.35 |
|  | day 7 | NA | | | 3.45 | ± | 0.52 | 2.12 | ± | 0.38 | 4.22 | ± | 0.56 | 2.87 | ± | 0.74 | 2.92 | ± | 0.26 | 3.30 | ± | 0.54 | 3.12 | ± | 0.64 |
|  | day 8 | 1.63 | ± | 0.45 | 4.36 | ± | 0.61 | 3.48 | ± | 0.56 | 4.10 | ± | 1.04 | 1.96 | ± | 0.28 | 2.81 | ± | 0.42 | 3.26 | ± | 0.50 | 2.93 | ± | 0.55 |

| POPULATION B | | | | | | | | | | | | | | | | | | | | | | | | | | | | | | | |
| --- | --- | --- | --- | --- | --- | --- | --- | --- | --- | --- | --- | --- | --- | --- | --- | --- | --- | --- | --- | --- | --- | --- | --- | --- | --- | --- | --- | --- | --- | --- | --- |
| Time  point | | SSG-sensitive strains | | | | | | | | | | | | | | | SSG-resistant strains | | | | | | | | | | | | | | |
|  |  | BPK282/0 | | | BPK294/0 | | | BPK035/0 | | | BPK043/0 | | | BPK181/0 | | | BPK085/0 | | | BPK164/1 | | | BPK177/0 | | | BPK178/0 | | | BPK181/12 | | |
| log phase | day 1 | 1.00 | ± | 0.15 | 1.54 | ± | 0.17 | 2.60 | ± | 0.34 | 1.05 | ± | 0.24 | 2.17 | ± | 0.22 | 2.33 | ± | 0.41 | 1.53 | ± | 0.33 | 4.12 | ± | 0.67 | 2.23 | ± | 0.48 | 2.18 | ± | 0.25 |
|  | day 2 | 1.37 | ± | 0.20 | 2.23 | ± | 0.29 | 2.22 | ± | 0.27 | 1.56 | ± | 0.32 | 1.40 | ± | 0.18 | 2.49 | ± | 0.38 | 1.64 | ± | 0.37 | 3.22 | ± | 0.41 | 2.50 | ± | 0.39 | 1.83 | ± | 0.24 |
|  | day 3 | 1.93 | ± | 0.30 | 2.67 | ± | 0.30 | 2.09 | ± | 0.24 | 2.28 | ± | 0.43 | 2.56 | ± | 0.38 | 2.75 | ± | 0.32 | 2.25 | ± | 0.39 | 4.78 | ± | 0.57 | 3.02 | ± | 0.46 | 2.77 | ± | 0.49 |
|  | day 4 | 2.37 | ± | 0.36 | 4.04 | ± | 0.49 | 2.67 | ± | 0.36 | 4.67 | ± | 1.01 | 2.01 | ± | 0.26 | 2.75 | ± | 0.35 | 2.15 | ± | 0.21 | 3.81 | ± | 0.59 | 2.63 | ± | 0.47 | 2.14 | ± | 0.36 |
| stationary phase | day 5 | 1.87 | ± | 0.35 | 2.94 | ± | 0.44 | 2.85 | ± | 0.47 | 3.04 | ± | 0.63 | 3.03 | ± | 0.52 | 2.39 | ± | 0.37 | 1.87 | ± | 0.34 | 3.91 | ± | 0.53 | 3.01 | ± | 0.48 | 2.95 | ± | 0.33 |
|  | day 6 | 2.48 | ± | 0.29 | 3.15 | ± | 0.35 | 2.50 | ± | 0.34 | 1.44 | ± | 0.32 | 3.33 | ± | 0.36 | 2.30 | ± | 0.30 | 1.64 | ± | 0.25 | 4.01 | ± | 0.47 | 3.17 | ± | 0.58 | 2.39 | ± | 0.45 |
|  | day 7 | 2.40 | ± | 0.28 | 3.04 | ± | 0.48 | 2.16 | ± | 0.36 | 1.73 | ± | 0.35 | 2.81 | ± | 0.34 | 2.48 | ± | 0.37 | 2.94 | ± | 0.38 | 5.19 | ± | 0.59 | 2.74 | ± | 0.62 | 2.86 | ± | 0.54 |
|  | day 8 | 2.60 | ± | 0.33 | 3.16 | ± | 0.35 | 2.56 | ± | 0.36 | 2.18 | ± | 0.43 | 2.21 | ± | 0.24 | 2.59 | ± | 0.45 | 2.28 | ± | 0.43 | 4.21 | ± | 0.50 | 2.12 | ± | 0.50 | 5.35 | ± | 1.06 |

#### Thiol dependent reductase 1 (TDR1)

| POPULATION A | | | | | | | | | | | | | | | | | | | | | | | | | |
| --- | --- | --- | --- | --- | --- | --- | --- | --- | --- | --- | --- | --- | --- | --- | --- | --- | --- | --- | --- | --- | --- | --- | --- | --- | --- |
| Time  point | | SSG-sensitive strains | | | | | | | | | SSG-resistant strains | | | | | | | | | | | | | | |
|  |  | BPK090/0 | | | BPK091/0 | | | BPK206/0 | | | BPK025/0 | | | BPK087/0 | | | BPK279/0 | | | BPK298/0 | | | BPK190/0 | | |
| log phase | day 1 | 2.51 | ± | 0.24 | 2.24 | ± | 0.22 | 3.41 | ± | 0.46 | 3.68 | ± | 0.45 | 2.09 | ± | 0.26 | 3.49 | ± | 0.44 | 3.29 | ± | 0.51 | 3.05 | ± | 0.31 |
|  | day 2 | 2.83 | ± | 0.28 | 2.50 | ± | 0.54 | 3.24 | ± | 0.56 | 3.17 | ± | 0.40 | 1.91 | ± | 0.28 | 4.09 | ± | 0.45 | 2.63 | ± | 0.35 | 2.99 | ± | 0.43 |
|  | day 3 | 2.03 | ± | 0.25 | 2.59 | ± | 0.28 | 3.12 | ± | 0.43 | 3.29 | ± | 0.67 | 1.75 | ± | 0.21 | 2.71 | ± | 0.37 | 1.85 | ± | 0.23 | 2.77 | ± | 0.36 |
|  | day 4 | 2.54 | ± | 0.31 | 1.95 | ± | 0.26 | 2.65 | ± | 0.44 | 2.92 | ± | 0.32 | 1.83 | ± | 0.19 | 2.82 | ± | 0.44 | 2.20 | ± | 0.40 | 2.58 | ± | 0.48 |
| stationary phase | day 5 | 2.26 | ± | 0.22 | 2.48 | ± | 0.44 | 3.33 | ± | 0.52 | 3.15 | ± | 0.39 | 2.07 | ± | 0.26 | 3.11 | ± | 0.45 | 2.18 | ± | 0.25 | 3.04 | ± | 0.37 |
|  | day 6 | 2.84 | ± | 0.34 | 1.91 | ± | 0.26 | 2.95 | ± | 0.33 | 3.29 | ± | 0.57 | 1.57 | ± | 0.23 | 2.52 | ± | 0.36 | 2.38 | ± | 0.33 | 2.89 | ± | 0.30 |
|  | day 7 | NA | | | 2.26 | ± | 0.22 | 3.70 | ± | 0.47 | 4.83 | ± | 0.68 | 1.33 | ± | 0.24 | 2.58 | ± | 0.26 | 3.07 | ± | 0.50 | 3.68 | ± | 0.64 |
|  | day 8 | 2.28 | ± | 0.43 | 2.32 | ± | 0.25 | 4.11 | ± | 0.56 | 5.07 | ± | 0.50 | 1.93 | ± | 0.21 | 2.63 | ± | 0.49 | 3.15 | ± | 0.57 | 3.29 | ± | 0.59 |

| POPULATION B | | | | | | | | | | | | | | | | | | | | | | | | | | | | | | | |
| --- | --- | --- | --- | --- | --- | --- | --- | --- | --- | --- | --- | --- | --- | --- | --- | --- | --- | --- | --- | --- | --- | --- | --- | --- | --- | --- | --- | --- | --- | --- | --- |
| Time  point | | SSG-sensitive strains | | | | | | | | | | | | | | | SSG-resistant strains | | | | | | | | | | | | | | |
|  |  | BPK282/0 | | | BPK294/0 | | | BPK035/0 | | | BPK043/0 | | | BPK181/0 | | | BPK085/0 | | | BPK164/1 | | | BPK177/0 | | | BPK178/0 | | | BPK181/12 | | |
| log phase | day 1 | 2.65 | ± | 0.49 | 2.99 | ± | 0.31 | 2.33 | ± | 0.32 | 3.49 | ± | 0.53 | 1.40 | ± | 0.13 | 2.56 | ± | 0.32 | 1.80 | ± | 0.18 | 2.55 | ± | 0.29 | 4.34 | ± | 0.66 | 2.77 | ± | 0.32 |
|  | day 2 | 2.71 | ± | 0.37 | 2.34 | ± | 0.23 | 2.65 | ± | 0.30 | 3.33 | ± | 0.49 | 1.99 | ± | 0.20 | 2.96 | ± | 0.36 | 1.54 | ± | 0.14 | 2.83 | ± | 0.43 | 3.12 | ± | 0.44 | 2.49 | ± | 0.31 |
|  | day 3 | 3.03 | ± | 0.39 | 2.57 | ± | 0.26 | 2.42 | ± | 0.30 | 2.97 | ± | 0.63 | 2.14 | ± | 0.19 | 2.70 | ± | 0.28 | 1.40 | ± | 0.19 | 3.22 | ± | 0.46 | 2.89 | ± | 0.32 | 2.85 | ± | 0.32 |
|  | day 4 | 2.94 | ± | 0.30 | 2.05 | ± | 0.29 | 2.24 | ± | 0.27 | 3.23 | ± | 0.41 | 1.49 | ± | 0.15 | 2.41 | ± | 0.34 | 1.28 | ± | 0.12 | 2.33 | ± | 0.22 | 2.81 | ± | 0.32 | 2.73 | ± | 0.45 |
| stationary phase | day 5 | 2.65 | ± | 0.41 | 2.10 | ± | 0.24 | 2.09 | ± | 0.32 | 2.65 | ± | 0.29 | 1.58 | ± | 0.27 | 2.61 | ± | 0.38 | 1.59 | ± | 0.15 | 2.20 | ± | 0.26 | 2.88 | ± | 0.42 | 2.76 | ± | 0.27 |
|  | day 6 | 2.62 | ± | 0.38 | 2.24 | ± | 0.21 | 2.88 | ± | 0.35 | 2.70 | ± | 0.24 | 1.49 | ± | 0.20 | 2.30 | ± | 0.27 | 1.47 | ± | 0.22 | 2.25 | ± | 0.22 | 3.15 | ± | 0.38 | 2.47 | ± | 0.25 |
|  | day 7 | 2.74 | ± | 0.29 | 2.40 | ± | 0.32 | 3.42 | ± | 0.51 | 2.88 | ± | 0.50 | 1.28 | ± | 0.24 | 3.11 | ± | 0.36 | 1.21 | ± | 0.15 | 3.18 | ± | 0.35 | 2.45 | ± | 0.36 | 2.26 | ± | 0.24 |
|  | day 8 | 2.72 | ± | 0.26 | 2.64 | ± | 0.37 | 3.96 | ± | 0.52 | 3.80 | ± | 0.64 | 1.00 | ± | 0.16 | 3.36 | ± | 0.48 | 1.81 | ± | 0.31 | 4.64 | ± | 0.67 | 4.96 | ± | 0.84 | 2.20 | ± | 0.35 |

#### Pentamidine resistance protein 1 (PRP1)

| POPULATION A | | | | | | | | | | | | | | | | | | | | | | | | | |
| --- | --- | --- | --- | --- | --- | --- | --- | --- | --- | --- | --- | --- | --- | --- | --- | --- | --- | --- | --- | --- | --- | --- | --- | --- | --- |
| Time  point | | SSG-sensitive strains | | | | | | | | | SSG-resistant strains | | | | | | | | | | | | | | |
|  |  | BPK090/0 | | | BPK091/0 | | | BPK206/0 | | | BPK025/0 | | | BPK087/0 | | | BPK279/0 | | | BPK298/0 | | | BPK190/0 | | |
| log phase | day 1 | 2.08 | ± | 0.28 | 2.04 | ± | 0.26 | 2.02 | ± | 0.29 | 2.14 | ± | 0.21 | 2.42 | ± | 0.32 | 1.34 | ± | 0.19 | 1.64 | ± | 0.25 | 2.77 | ± | 0.38 |
|  | day 2 | 1.60 | ± | 0.19 | 2.48 | ± | 0.55 | 1.80 | ± | 0.37 | 2.03 | ± | 0.24 | 1.60 | ± | 0.25 | 1.11 | ± | 0.12 | 1.20 | ± | 0.10 | 2.48 | ± | 0.30 |
|  | day 3 | 1.49 | ± | 0.24 | 1.82 | ± | 0.26 | 1.86 | ± | 0.24 | 1.92 | ± | 0.21 | 1.89 | ± | 0.28 | 1.00 | ± | 0.13 | 1.53 | ± | 0.12 | 1.93 | ± | 0.23 |
|  | day 4 | 1.83 | ± | 0.28 | 2.08 | ± | 0.31 | 1.24 | ± | 0.14 | 1.71 | ± | 0.30 | 1.41 | ± | 0.21 | 1.05 | ± | 0.15 | 1.33 | ± | 0.14 | 1.82 | ± | 0.35 |
| stationary phase | day 5 | 1.79 | ± | 0.20 | 2.17 | ± | 0.33 | 1.95 | ± | 0.20 | 1.54 | ± | 0.17 | 1.12 | ± | 0.14 | 1.30 | ± | 0.19 | 1.29 | ± | 0.18 | 1.69 | ± | 0.31 |
|  | day 6 | 1.22 | ± | 0.17 | 1.80 | ± | 0.22 | 1.98 | ± | 0.17 | 1.51 | ± | 0.27 | 1.31 | ± | 0.16 | 1.44 | ± | 0.21 | 1.26 | ± | 0.24 | 1.82 | ± | 0.23 |
|  | day 7 | NA | | | 2.26 | ± | 0.28 | 2.29 | ± | 0.19 | 2.35 | ± | 0.33 | 1.07 | ± | 0.13 | 1.55 | ± | 0.15 | 1.39 | ± | 0.24 | 2.16 | ± | 0.39 |
|  | day 8 | 1.91 | ± | 0.53 | 2.67 | ± | 0.40 | 2.21 | ± | 0.21 | 2.61 | ± | 0.37 | 1.96 | ± | 0.27 | 1.18 | ± | 0.17 | 1.93 | ± | 0.27 | 3.13 | ± | 0.41 |

| POPULATION B | | | | | | | | | | | | | | | | | | | | | | | | | | | | | | | |
| --- | --- | --- | --- | --- | --- | --- | --- | --- | --- | --- | --- | --- | --- | --- | --- | --- | --- | --- | --- | --- | --- | --- | --- | --- | --- | --- | --- | --- | --- | --- | --- |
| Time  point | | SSG-sensitive strains | | | | | | | | | | | | | | | SSG-resistant strains | | | | | | | | | | | | | | |
|  |  | BPK282/0 | | | BPK294/0 | | | BPK035/0 | | | BPK043/0 | | | BPK181/0 | | | BPK085/0 | | | BPK164/1 | | | BPK177/0 | | | BPK178/0 | | | BPK181/12 | | |
| log phase | day 1 | 1.52 | ± | 0.30 | 1.70 | ± | 0.20 | 1.66 | ± | 0.19 | 1.91 | ± | 0.25 | 1.98 | ± | 0.26 | 2.11 | ± | 0.21 | 2.30 | ± | 0.27 | 2.02 | ± | 0.30 | 1.76 | ± | 0.19 | 1.95 | ± | 0.25 |
|  | day 2 | 1.26 | ± | 0.17 | 1.61 | ± | 0.22 | 1.63 | ± | 0.23 | 2.60 | ± | 0.40 | 1.45 | ± | 0.23 | 1.76 | ± | 0.18 | 1.92 | ± | 0.23 | 1.69 | ± | 0.22 | 1.90 | ± | 0.28 | 1.74 | ± | 0.27 |
|  | day 3 | 1.53 | ± | 0.18 | 1.76 | ± | 0.17 | 1.64 | ± | 0.20 | 2.97 | ± | 0.52 | 1.53 | ± | 0.21 | 2.03 | ± | 0.18 | 1.80 | ± | 0.24 | 1.41 | ± | 0.17 | 1.63 | ± | 0.16 | 1.77 | ± | 0.23 |
|  | day 4 | 1.56 | ± | 0.19 | 1.67 | ± | 0.17 | 1.59 | ± | 0.22 | 2.20 | ± | 0.45 | 1.37 | ± | 0.18 | 2.18 | ± | 0.21 | 1.68 | ± | 0.20 | 1.55 | ± | 0.20 | 1.77 | ± | 0.15 | 1.48 | ± | 0.18 |
| stationary phase | day 5 | 1.38 | ± | 0.13 | 1.62 | ± | 0.18 | 2.14 | ± | 0.31 | 2.14 | ± | 0.29 | 1.47 | ± | 0.23 | 1.88 | ± | 0.32 | 1.83 | ± | 0.21 | 1.83 | ± | 0.26 | 1.62 | ± | 0.16 | 1.73 | ± | 0.21 |
|  | day 6 | 1.68 | ± | 0.19 | 1.65 | ± | 0.24 | 1.75 | ± | 0.22 | 1.82 | ± | 0.20 | 1.41 | ± | 0.17 | 1.85 | ± | 0.26 | 1.49 | ± | 0.43 | 1.71 | ± | 0.21 | 2.01 | ± | 0.23 | 1.28 | ± | 0.16 |
|  | day 7 | 1.61 | ± | 0.16 | 1.66 | ± | 0.19 | 1.77 | ± | 0.26 | 3.06 | ± | 0.35 | 1.67 | ± | 0.24 | 1.82 | ± | 0.20 | 1.79 | ± | 0.24 | 1.92 | ± | 0.31 | 2.27 | ± | 0.37 | 1.94 | ± | 0.31 |
|  | day 8 | 1.55 | ± | 0.17 | 1.61 | ± | 0.23 | 2.29 | ± | 0.33 | 3.75 | ± | 0.73 | 1.49 | ± | 0.22 | 2.23 | ± | 0.36 | 2.23 | ± | 0.31 | 2.39 | ± | 0.38 | 2.56 | ± | 0.25 | 4.15 | ± | 0.71 |

#### Multidrug resistance protein A (MRPA)

| POPULATION A | | | | | | | | | | | | | | | | | | | | | | | | | |
| --- | --- | --- | --- | --- | --- | --- | --- | --- | --- | --- | --- | --- | --- | --- | --- | --- | --- | --- | --- | --- | --- | --- | --- | --- | --- |
| Time  point | | SSG-sensitive strains | | | | | | | | | SSG-resistant strains | | | | | | | | | | | | | | |
|  |  | BPK090/0 | | | BPK091/0 | | | BPK206/0 | | | BPK025/0 | | | BPK087/0 | | | BPK279/0 | | | BPK298/0 | | | BPK190/0 | | |
| log phase | day 1 | 1.85 | ± | 0.14 | 1.63 | ± | 0.21 | 1.59 | ± | 0.24 | 1.39 | ± | 0.14 | 2.35 | ± | 0.27 | 1.51 | ± | 0.15 | 2.15 | ± | 0.23 | 1.32 | ± | 0.13 |
|  | day 2 | 1.55 | ± | 0.12 | 1.87 | ± | 0.31 | 1.57 | ± | 0.28 | 1.47 | ± | 0.15 | 2.04 | ± | 0.28 | 1.35 | ± | 0.14 | 1.83 | ± | 0.22 | 1.16 | ± | 0.13 |
|  | day 3 | 1.48 | ± | 0.17 | 1.85 | ± | 0.17 | 1.55 | ± | 0.19 | 1.17 | ± | 0.17 | 2.05 | ± | 0.20 | 1.37 | ± | 0.14 | 2.05 | ± | 0.21 | 1.00 | ± | 0.14 |
|  | day 4 | 1.61 | ± | 0.13 | 1.81 | ± | 0.22 | 1.66 | ± | 0.16 | 1.13 | ± | 0.10 | 1.72 | ± | 0.15 | 1.44 | ± | 0.18 | 2.29 | ± | 0.26 | 1.11 | ± | 0.19 |
| stationary phase | day 5 | 1.88 | ± | 0.18 | 1.97 | ± | 0.26 | 1.61 | ± | 0.17 | 1.03 | ± | 0.12 | 1.60 | ± | 0.13 | 1.23 | ± | 0.17 | 2.57 | ± | 0.26 | 1.13 | ± | 0.13 |
|  | day 6 | 1.49 | ± | 0.21 | 1.94 | ± | 0.15 | 1.56 | ± | 0.22 | 1.27 | ± | 0.14 | 2.16 | ± | 0.23 | 1.61 | ± | 0.19 | 2.38 | ± | 0.29 | 1.42 | ± | 0.11 |
|  | day 7 | NA | | | 1.97 | ± | 0.16 | 1.75 | ± | 0.18 | 1.40 | ± | 0.13 | 2.22 | ± | 0.30 | 1.59 | ± | 0.14 | 2.30 | ± | 0.40 | 1.50 | ± | 0.32 |
|  | day 8 | 1.54 | ± | 0.32 | 1.90 | ± | 0.25 | 1.77 | ± | 0.24 | 1.48 | ± | 0.15 | 1.91 | ± | 0.17 | 1.55 | ± | 0.19 | 2.70 | ± | 0.29 | 1.96 | ± | 0.27 |

| POPULATION B | | | | | | | | | | | | | | | | | | | | | | | | | | | | | | | |
| --- | --- | --- | --- | --- | --- | --- | --- | --- | --- | --- | --- | --- | --- | --- | --- | --- | --- | --- | --- | --- | --- | --- | --- | --- | --- | --- | --- | --- | --- | --- | --- |
| Time  point | | SSG-sensitive strains | | | | | | | | | | | | | | | SSG-resistant strains | | | | | | | | | | | | | | |
|  |  | BPK282/0 | | | BPK294/0 | | | BPK035/0 | | | BPK043/0 | | | BPK181/0 | | | BPK085/0 | | | BPK164/1 | | | BPK177/0 | | | BPK178/0 | | | BPK181/12 | | |
| log phase | day 1 | 2.26 | ± | 0.32 | 2.36 | ± | 0.26 | 1.83 | ± | 0.19 | 1.30 | ± | 0.16 | 1.72 | ± | 0.14 | 2.18 | ± | 0.28 | 2.54 | ± | 0.33 | 2.50 | ± | 0.35 | 2.00 | ± | 0.24 | 1.61 | ± | 0.26 |
|  | day 2 | 2.24 | ± | 0.29 | 2.33 | ± | 0.29 | 2.06 | ± | 0.23 | 1.48 | ± | 0.16 | 1.64 | ± | 0.17 | 1.98 | ± | 0.26 | 2.12 | ± | 0.17 | 2.37 | ± | 0.34 | 2.21 | ± | 0.38 | 1.47 | ± | 0.14 |
|  | day 3 | 2.40 | ± | 0.30 | 2.13 | ± | 0.26 | 2.09 | ± | 0.18 | 1.64 | ± | 0.29 | 1.87 | ± | 0.20 | 2.01 | ± | 0.29 | 2.04 | ± | 0.17 | 1.82 | ± | 0.18 | 1.88 | ± | 0.20 | 1.63 | ± | 0.22 |
|  | day 4 | 2.56 | ± | 0.31 | 1.88 | ± | 0.27 | 2.04 | ± | 0.16 | 1.40 | ± | 0.11 | 1.68 | ± | 0.15 | 1.96 | ± | 0.25 | 2.02 | ± | 0.20 | 2.22 | ± | 0.29 | 1.97 | ± | 0.20 | 1.28 | ± | 0.11 |
| stationary phase | day 5 | 2.34 | ± | 0.31 | 2.13 | ± | 0.23 | 2.07 | ± | 0.22 | 1.54 | ± | 0.18 | 1.56 | ± | 0.16 | 2.10 | ± | 0.33 | 2.04 | ± | 0.17 | 2.65 | ± | 0.29 | 2.30 | ± | 0.41 | 1.65 | ± | 0.15 |
|  | day 6 | 2.26 | ± | 0.39 | 2.15 | ± | 0.22 | 1.84 | ± | 0.24 | 1.42 | ± | 0.17 | 1.52 | ± | 0.13 | 2.25 | ± | 0.31 | 2.02 | ± | 0.24 | 2.54 | ± | 0.23 | 2.24 | ± | 0.32 | 1.50 | ± | 0.14 |
|  | day 7 | 2.41 | ± | 0.49 | 2.09 | ± | 0.28 | 2.31 | ± | 0.38 | 1.66 | ± | 0.26 | 1.62 | ± | 0.29 | 2.18 | ± | 0.30 | 2.12 | ± | 0.24 | 2.72 | ± | 0.22 | 3.05 | ± | 0.43 | 1.78 | ± | 0.19 |
|  | day 8 | 2.17 | ± | 0.24 | 2.15 | ± | 0.31 | 2.21 | ± | 0.42 | 2.29 | ± | 0.39 | 1.38 | ± | 0.14 | 2.45 | ± | 0.35 | 2.81 | ± | 0.30 | 2.88 | ± | 0.32 | 2.45 | ± | 0.29 | 2.35 | ± | 0.31 |
