# Supplementary material for: Molecular Mechanisms of Drug Resistance in Natural Leishmania Populations Vary with Genetic Background
Source: PLoS Negl Trop Dis. 2012 Feb 28;6(2):e1514. doi: 10.1371/journal.pntd.0001514 (PMC3289598; doi:10.1371/journal.pntd.0001514)
Supplement: Dataset S2 — DNA sequence of 11 target genes in 11 L. (L.) donovani clones with variable in vitro SSG susceptibility. The coding DNA sequences (+500 basepairs upstream and downstream) of 11 proteins reported to be involved in SSG/SbV/SbIII metabolism were determined by Illumina sequencing in 11 L. (L.) donovani clones with differential in vitro SSG susceptibility. (DOC) [file pntd.0001514.s002.doc]

## Dataset S2.

### 1. CYSTATHIONINE BETA-SYNTHASE

>BPK035/0 clone 1|LinJ17_V3.0300|500 bp UPS + CDS + 500 bp DWS| cystathionine beta-synthase

GAGAGGCGGCGCGGCAGCAAGAGAGAGTGAGTGAGTGCGAAGGAATGAGGAGGAGGCAGAATGGATGGGGGCCTGGGCTAATCGAGGGGACCACCGCGTGTCTGCGTGCGAGGTACCCCCTCCCTCTCCACACATCAAAAGAAGAGAATGCTGTCATGTTGCAGCAGCTCTCCAGATTAAACCCTCCCTCTCTCCTTCTCTCTCCCGTGCCTCTACGCCATGCTCTGCCCGACGTTGCTCTTCTACCCCCACCCCGCATCATACATCCTTCTCTGTTTTTCCTTAACGTTTCCGCGACAGCTCCCTCCTTCGTTCGTCTAATCCGCAGCAGCACCTCCACCGTTGCGAGCCACTGCTGTGTCTCGTGTGGACTTACACATGTCACTGCGTCTTCATCCGTCCCCGTTACACCGTTTTTCTCCACCATCACCGCCACCGCCACCCGTTGGGATGCATCGATATGTGCCGCTTCCCCCTCCAGAACCCATTCCGTCATTTCTATGACATCTGCAACCCCCCGCGGCCAAATTCTGGCCAACGCCCTCGAGGCGGTCGGCAACACCCCTTGCATCCGCTTGAACCGCGTCCCACAGAAGCACGGCATTCAGTGCGAGGTTGTGGCCAAGTGCGAGTTCTTCAACCCTGGCGGCAGCGTCAAGGACCGCATTGGCAAGCAGATGATACTCGACGCCGAAAAGAACGGGACGCTCAAGCCGGGTTCCGTGATTGTGGAGGCCACAAGCGGCAATACTGGCATCGGTCTCTCCATGGCCGCGGCGATCCGCGGCTACCACATGGTGATTACGATGCCGAAGAAGATGTCGCACGAGAAGGAGACGACACTGCAGTCCCTTGGCGCCGAGGTGATCCGCACGGAGACGTCTCTTCCGCACGACCACCCGGACAGCCTCATCGGGGTCGCTCGCCGCCTGCGCGATGAGAAGGGCTACGTGCTGCTGGACCAGTACACGAACCTCAGCAACCCGGGTGCGCATTACGAGTTCACTGGTCAGGAGATCTACGACCAGTGCGGCGGCAAGGTCGACATGGTAGTCATCTGCGCCGGAACCGGAGGCACGATCACCGGAGTGGCCAAGAAGCTGAAGGAGCTCATCCCCGAAGTCATCGTTGTCGGTGTAGACCCGGTCGGCAGCATCATCGCCGACCCCGAACACCCCGGCGAGCCTGTCATGTACCACGTAGAGGGAATCGGCTATGACTTTGTGCCAGACGTGTGCGAGCGTAAGTACGTCGATCGCTGGGTCAAGACACGCGATCAGGAGAGTTTCGACCTCGCCCTCGAGCTGCACCGCGAGGAGGGTCTGCTGGTCGGCGGTAGCAGCGGCTCCGCGATGGCCGGCGTGGTGGAGGCAGCCAAGGACCTTCGCTCGGATCAGCGCTGCGTCGTGCTCATGGCGGACGGCATCCGCAACTACATGGGGAAGTTCGCCGATGTCAACTGGATGATCGAGCACGGCTTCCGGCAGGGTGAGGTGACCCGCCCCACCTACGACACGCTGAAGAAGGAGCTGGAGGAGGTCAAGGCGAAGCTAGCAAAGTACGAAAGCGGCGCTCAGTAGCGGGTCGATGCTGGAACATATCTTCTCAAAATCTCACGAGGGGAAGCAGAAGACACGCGTGCCAACTTTTCCTTGGGATGCCCAACACGCTTGTGGGTGCACCGACATGTTGTGCGAGGCCTCTTTCCTGCTTCTTCCGACATGCACACAGTGCACTCGTTCCTGGGAACTGCCCACCGTCGAAAGCCATATCACTTCTTGCCTCCCGGTCGGTGTCCTCCCCTACCCCGCACCAACCGCACAATTGGGACGCGCCTTTCTCACCGAAGTCCACCTTTGCCCTCTCTTTCCCTGCTTTCGCTCTTCTTCCCCATCCTTCCACCTTCAGTGCCTCTTTCCATCACTGCCTGCGTGTCTGCCAAATCGTTCGCCCCCCCCCCTTCTCCACAGTCGACAACAACAGCCATCGCCTTCTATCATCCTCTTCTTTTTCTGGAAGCATTTTTCTTGTCCCCTTTCCCACGCATTCTCTCTTTCTCTCTCTGTTCCCCGCTGATCAT

>BPK043/0 clone 2|LinJ17_V3.0300|500 bp UPS + CDS + 500 bp DWS|cystathionine beta-synthase

GAGAGGCGGCGCGGCAGCAAGAGAGAGTGAGTGAGTGCGAAGGAATGAGGAGGAGGCAGAATGGATGGGGGCCTGGGCTAATCGAGGGGACCACCGCGTGTCTGCGTGCGAGGTACCCCCTCCCTCTCCACACATCAAAAGAAGAGAATGCTGTCATGTTGCAGCAGCTCTCCAGATTAAACCCTCCCTCTCTCCTTCTCTCTCCCGTGCCTCTACGCCATGCTCTGCCCGACGTTGCTCTTCTACCCCCACCCCGCATCATACATCCTTCTCTGTTTTTCCTTAACGTTTCCGCGACAGCTCCCTCCTTCGTTCGTCTAATCCGCAGCAGCACCTCCACCGTTGCGAGCCACTGCTGTGTCTCGTGTGGACTTACACATGTCACTGCGTCTTCATCCGTCCCCGTTACACCGTTTTTCTCCACCATCACCGCCACCGCCACCCGTTGGGATGCATCGATATGTGCCGCTTCCCCCTCCAGAACCCATTCCGTCATTTCTATGACATCTGCAACCCCCCGCGGCCAAATTCTGGCCAACGCCCTCGAGGCGGTCGGCAACACCCCTTGCATCCGCTTGAACCGCGTCCCACAGAAGCACGGCATTCAGTGCGAGGTTGTGGCCAAGTGCGAGTTCTTCAACCCTGGCGGCAGCGTCAAGGACCGCATTGGCAAGCAGATGATACTCGACGCCGAAAAGAACGGGACGCTCAAGCCGGGTTCCGTGATTGTGGAGGCCACAAGCGGCAATACTGGCATCGGTCTCTCCATGGCCGCGGCGATCCGCGGCTACCACATGGTGATTACGATGCCGAAGAAGATGTCGCACGAGAAGGAGACGACACTGCAGTCCCTTGGCGCCGAGGTGATCCGCACGGAGACGTCTCTTCCGCACGACCACCCGGACAGCCTCATCGGGGTCGCTCGCCGCCTGCGCGATGAGAAGGGCTACGTGCTGCTGGACCAGTACACGAACCTCAGCAACCCGGGTGCGCATTACGAGTTCACTGGTCAGGAGATCTACGACCAGTGCGGCGGCAAGGTCGACATGGTAGTCATCTGCGCCGGAACCGGAGGCACGATCACCGGAGTGGCCAAGAAGCTGAAGGAGCTCATCCCCGAAGTCATCGTTGTCGGTGTAGACCCGGTCGGCAGCATCATCGCCGACCCCGAACACCCCGGCGAGCCTGTCATGTACCACGTAGAGGGAATCGGCTATGACTTTGTGCCAGACGTGTGCGAGCGTAAGTACGTCGATCGCTGGGTCAAGACACGCGATCAGGAGAGTTTCGACCTCGCCCTCGAGCTGCACCGCGAGGAGGGTCTGCTGGTCGGCGGTAGCAGCGGCTCCGCGATGGCCGGCGTGGTGGAGGCAGCCAAGGACCTTCGCTCGGATCAGCGCTGCGTCGTGCTCATGGCGGACGGCATCCGCAACTACATGGGGAAGTTCGCCGATGTCAACTGGATGATCGAGCACGGCTTCCGGCAGGGTGAGGTGACCCGCCCCACCTACGACACGCTGAAGAAGGAGCTGGAGGAGGTCAAGGCGAAGCTAGCAAAGTACGAAAGCGGCGCTCAGTAGCGGGTCGATGCTGGAACATATCTTCTCAAAATCTCACGAGGGGAAGCAGAAGACACGCGTGCCAACTTTTCCTTGGGATGCCCAACACGCTTGTGGGTGCACCGACATGTTGTGCGAGGCCTCTTTCCTGCTTCTTCCGACATGCACACAGTGCACTCGTTCCTGGGAACTGCCCACCGTCGAAAGCCATATCACTTCTTGCCTCCCGGTCGGTGTCCTCCCCTACCCCGCACCAACCGCACAATTGGGACGCGCCTTTCTCACCGAAGTCCACCTTTGCCCTCTCTTTCCCTGCTTTCGCTCTTCTTCCCCATCCTTCCACCTTCAGTGCCTCTTTCCATCACTGCCTGCGTGTCTGCCAAATCGTTCGCCCCCCCCCCTTCTCCACAGTCGACAACAACAGCCATCGCCTTCTATCATCCTCTTCTTTTTCTGGAAGCATTTTTCTTGTCCCCTTTCCCACGCATTCTCTCTTTCTCTCTCTGTTCCCCGCTGATCAT

>BPK085/0 clone 8|LinJ17_V3.0300|500 bp UPS + CDS + 500 bp DWS|cystathionine beta-synthase

GAGAGGCGGCGCGGCAGCAAGAGAGAGTGAGTGAGTGCGAAGGAATGAGGAGGAGGCAGAATGGATGGGGGCCTGGGCTAATCGAGGGGACCACCGCGTGTCTGCGTGCGAGGTACCCCCTCCCTCTCCACACATCAAAAGAAGAGAATGCTGTCATGTTGCAGCAGCTCTCCAGATTAAACCCTCCCTCTCTCCTTCTCTCTCCCGTGCCTCTACGCCATGCTCTGCCCGACGTTGCTCTTCTACCCCCACCCCGCATCATACATCCTTCTCTGTTTTTCCTTAACGTTTCCGCGACAGCTCCCTCCTTCGTTCGTCTAATCCGCAGCAGCACCTCCACCGTTGCGAGCCACTGCTGTGTCTCGTGTGGACTTACACATGTCACTGCGTCTTCATCCGTCCCCGTTACACCGTTTTTCTCCACCATCACCGCCACCGCCACCCGTTGGGATGCATCGATATGTGCCGCTTCCCCCTCCAGAACCCATTCCGTCATTTCTATGACATCTGCAACCCCCCGCGGCCAAATTCTGGCCAACGCCCTCGAGGCGGTCGGCAACACCCCTTGCATCCGCTTGAACCGCGTCCCACAGAAGCACGGCATTCAGTGCGAGGTTGTGGCCAAGTGCGAGTTCTTCAACCCTGGCGGCAGCGTCAAGGACCGCATTGGCAAGCAGATGATACTCGACGCCGAAAAGAACGGGACGCTCAAGCCGGGTTCCGTGATTGTGGAGGCCACAAGCGGCAATACTGGCATCGGTCTCTCCATGGCCGCGGCGATCCGCGGCTACCACATGGTGATTACGATGCCGAAGAAGATGTCGCACGAGAAGGAGACGACACTGCAGTCCCTTGGCGCCGAGGTGATCCGCACGGAGACGTCTCTTCCGCACGACCACCCGGACAGCCTCATCGGGGTCGCTCGCCGCCTGCGCGATGAGAAGGGCTACGTGCTGCTGGACCAGTACACGAACCTCAGCAACCCGGGTGCGCATTACGAGTTCACTGGTCAGGAGATCTACGACCAGTGCGGCGGCAAGGTCGACATGGTAGTCATCTGCGCCGGAACCGGAGGCACGATCACCGGAGTGGCCAAGAAGCTGAAGGAGCTCATCCCCGAAGTCATCGTTGTCGGTGTAGACCCGGTCGGCAGCATCATCGCCGACCCCGAACACCCCGGCGAGCCTGTCATGTACCACGTAGAGGGAATCGGCTATGACTTTGTGCCAGACGTGTGCGAGCGTAAGTACGTCGATCGCTGGGTCAAGACACGCGATCAGGAGAGTTTCGACCTCGCCCTCGAGCTGCACCGCGAGGAGGGTCTGCTGGTCGGCGGTAGCAGCGGCTCCGCGATGGCCGGCGTGGTGGAGGCAGCCAAGGACCTTCGCTCGGATCAGCGCTGCGTCGTGCTCATGGCGGACGGCATCCGCAACTACATGGGGAAGTTCGCCGATGTCAACTGGATGATCGAGCACGGCTTCCGGCAGGGTGAGGTGACCCGCCCCACCTACGACACGCTGAAGAAGGAGCTGGAGGAGGTCAAGGCGAAGCTAGCAAAGTACGAAAGCGGCGCTCAGTAGCGGGTCGATGCTGGAACATATCTTCTCAAAATCTCACGAGGGGAAGCAGAAGACACGCGTGCCAACTTTTCCTTGGGATGCCCAACACGCTTGTGGGTGCACCGACATGTTGTGCGAGGCCTCTTTCCTGCTTCTTCCGACATGCACACAGTGCACTCGTTCCTGGGAACTGCCCACCGTCGAAAGCCATATCACTTCTTGCCTCCCGGTCGGTGTCCTCCCCTACCCCGCACCAACCGCACAATTGGGACGCGCCTTTCTCACCGAAGTCCACCTTTGCCCTCTCTTTCCCTGCTTTCGCTCTTCTTCCCCATCCTTCCACCTTCAGTGCCTCTTTCCATCACTGCCTGCGTGTCTGCCAAATCGTTCGCCCCCCCCCCTTCTCCACAGTCGACAACAACAGCCATCGCCTTCTATCATCCTCTTCTTTTTCTGGAAGCATTTTTCTTGTCCCCTTTCCCACGCATTCTCTCTTTCTCTCTCTGTTCCCCGCTGATCAT

>BPK087/0 clone 11|LinJ17_V3.0300|500 bp UPS + CDS + 500 bp DWS|cystathionine beta-synthase

GAGAGGCGGCGCGGCAGCAAGAGAGAGTGAGTGAGTGCGAAGGAATGAGGAGGAGGCAGAATGGATGGGGGCCTGGGCTAATCGAGGGGACCACCGCGTGTCTGCGTGCGAGGTACCCCCTCCCTCTCCACACATCAAAAGAAGAGAATGCTGTCATGTTGCAGCAGCTCTCCAGATTAAACCCTCCCTCTCTCCTTCTCTCTCCCGTGCCTCTACGCCATGCTCTGCCCGACGTTGCTCTTCTACCCCCACCCCGCATCATACATCCTTCTCTGTTTTTCCTTAACGTTTCCGCGACAGCTCCCTCCTTCGTTCGTCTAATCCGCAGCAGCACCTCCACCGTTGCGAGCCACTGCTGTGTCTCGTGTGGACTTACACATGTCACTGCGTCTTCATCCGTCCCCGTTACACCGTTTTTCTCCACCATCACCGCCACCGCCACCCGTTGGGATGCATCGATATGTGCCGCTTCCCCCTCCAGAACCCATTCCGTCATTTCTATGACATCTGCAACCCCCCGCGGCCAAATTCTGGCCAACGCCCTCGAGGCGGTCGGCAACACCCCTTGCATCCGCTTGAACCGCGTCCCACAGAAGCACGGCATTCAGTGCGAGGTTGTGGCCAAGTGCGAGTTCTTCAACCCTGGCGGCAGCGTCAAGGACCGCATTGGCAAGCAGATGATACTCGACGCCGAAAAGAACGGGACGCTCAAGCCGGGTTCCGTGATTGTGGAGGCCACAAGCGGCAATACTGGCATCGGTCTCTCCATGGCCGCGGCGATCCGCGGCTACCACATGGTGATTACGATGCCGAAGAAGATGTCGCACGAGAAGGAGACGACACTGCAGTCCCTTGGCGCCGAGGTGATCCGCACGGAGACGTCTCTTCCGCACGACCACCCGGACAGCCTCATCGGGGTCGCTCGCCGCCTGCGCGATGAGAAGGGCTACGTGCTGCTGGACCAGTACACGAACCTCAGCAACCCGGGTGCGCATTACGAGTTCACTGGTCAGGAGATCTACGACCAGTGCGGCGGCAAGGTCGACATGGTAGTCATCTGCGCCGGAACCGGAGGCACGATCACCGGAGTGGCCAAGAAGCTGAAGGAGCTCATCCCCGAAGTCATCGTTGTCGGTGTAGACCCGGTCGGCAGCATCATCGCCGACCCCGAACACCCCGGCGAGCCTGTCATGTACCACGTAGAGGGAATCGGCTATGACTTTGTGCCAGACGTGTGCGAGCGTAAGTACGTCGATCGCTGGGTCAAGACACGCGATCAGGAGAGTTTCGACCTCGCCCTCGAGCTGCACCGCGAGGAGGGTCTGCTGGTCGGCGGTAGCAGCGGCTCCGCGATGGCCGGCGTGGTGGAGGCAGCCAAGGACCTTCGCTCGGATCAGCGCTGCGTCGTGCTCATGGCGGACGGCATCCGCAACTACATGGGGAAGTTCGCCGATGTCAACTGGATGATCGAGCACGGCTTCCGGCAGGGTGAGGTGACCCGCCCCACCTACGACACGCTGAAGAAGGAGCTGGAGGAGGTCAAGGCGAAGCTAGCAAAGTACGAAAGCGGCGCTCAGTAGCGGGTCGATGCTGGAACATATCTTCTCAAAATCTCACGAGGGGAAGCAGAAGACACGCGTGCCAACTTTTCCTTGGGATGCCCAACACGCTTGTGGGTGCACCGACATGTTGTGCGAGGCCTCTTTCCTGCTTCTTCCGACATGCACACAGTGCACTCGTTCCTGGGAACTGCCCACCGTCGAAAGCCATATCACTTCTTGCCTCCCGGTCGGTGTCCTCCCCTACCCCGCACCAACCGCACAATTGGGACGCGCCTTTCTCACCGAAGTCCACCTTTGCCCTCTCTTTCCCTGCTTTCGCTCTTCTTCCCCATCCTTCCACCTTCAGTGCCTCTTTCCATCACTGCCTGCGTGTCTGCCAAATCGTTCGCCCCCCCCCCTTCTCCACAGTCGACAACAACAGCCATCGCCTTCTATCATCCTCTTCTTTTTCTGGAAGCATTTTTCTTGTCCCCTTTCCCACGCATTCTCTCTTTCTCTCTCTGTTCCCCGCTGATCAT

>BPK178/0 clone 3|LinJ17_V3.0300|500 bp UPS + CDS + 500 bp DWS|cystathionine beta-synthase

GAGAGGCGGCGCGGCAGCAAGAGAGAGTGAGTGAGTGCGAAGGAATGAGGAGGAGGCAGAATGGATGGGGGCCTGGGCTAATCGAGGGGACCACCGCGTGTCTGCGTGCGAGGTACCCCCTCCCTCTCCACACATCAAAAGAAGAGAATGCTGTCATGTTGCAGCAGCTCTCCAGATTAAACCCTCCCTCTCTCCTTCTCTCTCCCGTGCCTCTACGCCATGCTCTGCCCGACGTTGCTCTTCTACCCCCACCCCGCATCATACATCCTTCTCTGTTTTTCCTTAACGTTTCCGCGACAGCTCCCTCCTTCGTTCGTCTAATCCGCAGCAGCACCTCCACCGTTGCGAGCCACTGCTGTGTCTCGTGTGGACTTACACATGTCACTGCGTCTTCATCCGTCCCCGTTACACCGTTTTTCTCCACCATCACCGCCACCGCCACCCGTTGGGATGCATCGATATGTGCCGCTTCCCCCTCCAGAACCCATTCCGTCATTTCTATGACATCTGCAACCCCCCGCGGCCAAATTCTGGCCAACGCCCTCGAGGCGGTCGGCAACACCCCTTGCATCCGCTTGAACCGCGTCCCACAGAAGCACGGCATTCAGTGCGAGGTTGTGGCCAAGTGCGAGTTCTTCAACCCTGGCGGCAGCGTCAAGGACCGCATTGGCAAGCAGATGATACTCGACGCCGAAAAGAACGGGACGCTCAAGCCGGGTTCCGTGATTGTGGAGGCCACAAGCGGCAATACTGGCATCGGTCTCTCCATGGCCGCGGCGATCCGCGGCTACCACATGGTGATTACGATGCCGAAGAAGATGTCGCACGAGAAGGAGACGACACTGCAGTCCCTTGGCGCCGAGGTGATCCGCACGGAGACGTCTCTTCCGCACGACCACCCGGACAGCCTCATCGGGGTCGCTCGCCGCCTGCGCGATGAGAAGGGCTACGTGCTGCTGGACCAGTACACGAACCTCAGCAACCCGGGTGCGCATTACGAGTTCACTGGTCAGGAGATCTACGACCAGTGCGGCGGCAAGGTCGACATGGTAGTCATCTGCGCCGGAACCGGAGGCACGATCACCGGAGTGGCCAAGAAGCTGAAGGAGCTCATCCCCGAAGTCATCGTTGTCGGTGTAGACCCGGTCGGCAGCATCATCGCCGACCCCGAACACCCCGGCGAGCCTGTCATGTACCACGTAGAGGGAATCGGCTATGACTTTGTGCCAGACGTGTGCGAGCGTAAGTACGTCGATCGCTGGGTCAAGACACGCGATCAGGAGAGTTTCGACCTCGCCCTCGAGCTGCACCGCGAGGAGGGTCTGCTGGTCGGCGGTAGCAGCGGCTCCGCGATGGCCGGCGTGGTGGAGGCAGCCAAGGACCTTCGCTCGGATCAGCGCTGCGTCGTGCTCATGGCGGACGGCATCCGCAACTACATGGGGAAGTTCGCCGATGTCAACTGGATGATCGAGCACGGCTTCCGGCAGGGTGAGGTGACCCGCCCCACCTACGACACGCTGAAGAAGGAGCTGGAGGAGGTCAAGGCGAAGCTAGCAAAGTACGAAAGCGGCGCTCAGTAGCGGGTCGATGCTGGAACATATCTTCTCAAAATCTCACGAGGGGAAGCAGAAGACACGCGTGCCAACTTTTCCTTGGGATGCCCAACACGCTTGTGGGTGCACCGACATGTTGTGCGAGGCCTCTTTCCTGCTTCTTCCGACATGCACACAGTGCACTCGTTCCTGGGAACTGCCCACCGTCGAAAGCCATATCACTTCTTGCCTCCCGGTCGGTGTCCTCCCCTACCCCGCACCAACCGCACAATTGGGACGCGCCTTTCTCACCGAAGTCCACCTTTGCCCTCTCTTTCCCTGCTTTCGCTCTTCTTCCCCATCCTTCCACCTTCAGTGCCTCTTTCCATCACTGCCTGCGTGTCTGCCAAATCGTTCGCCCCCCCCCCTTCTCCACAGTCGACAACAACAGCCATCGCCTTCTATCATCCTCTTCTTTTTCTGGAAGCATTTTTCTTGTCCCCTTTCCCACGCATTCTCTCTTTCTCTCTCTGTTCCCCGCTGATCAT

>BPK190/0 clone 3|LinJ17_V3.0300|500 bp UPS + CDS + 500 bp DWS|cystathionine beta-synthase

GAGAGGCGGCGCGGCAGCAAGAGAGAGTGAGTGAGTGCGAAGGAATGAGGAGGAGGCAGAATGGATGGGGGCCTGGGCTAATCGAGGGGACCACCGCGTGTCTGCGTGCGAGGTACCCCCTCCCTCTCCACACATCAAAAGAAGAGAATGCTGTCATGTTGCAGCAGCTCTCCAGATTAAACCCTCCCTCTCTCCTTCTCTCTCCCGTGCCTCTACGCCATGCTCTGCCCGACGTTGCTCTTCTACCCCCACCCCGCATCATACATCCTTCTCTGTTTTTCCTTAACGTTTCCGCGACAGCTCCCTCCTTCGTTCGTCTAATCCGCAGCAGCACCTCCACCGTTGCGAGCCACTGCTGTGTCTCGTGTGGACTTACACATGTCACTGCGTCTTCATCCGTCCCCGTTACACCGTTTTTCTCCACCATCACCGCCACCGCCACCCGTTGGGATGCATCGATATGTGCCGCTTCCCCCTCCAGAACCCATTCCGTCATTTCTATGACATCTGCAACCCCCCGCGGCCAAATTCTGGCCAACGCCCTCGAGGCGGTCGGCAACACCCCTTGCATCCGCTTGAACCGCGTCCCACAGAAGCACGGCATTCAGTGCGAGGTTGTGGCCAAGTGCGAGTTCTTCAACCCTGGCGGCAGCGTCAAGGACCGCATTGGCAAGCAGATGATACTCGACGCCGAAAAGAACGGGACGCTCAAGCCGGGTTCCGTGATTGTGGAGGCCACAAGCGGCAATACTGGCATCGGTCTCTCCATGGCCGCGGCGATCCGCGGCTACCACATGGTGATTACGATGCCGAAGAAGATGTCGCACGAGAAGGAGACGACACTGCAGTCCCTTGGCGCCGAGGTGATCCGCACGGAGACGTCTCTTCCGCACGACCACCCGGACAGCCTCATCGGGGTCGCTCGCCGCCTGCGCGATGAGAAGGGCTACGTGCTGCTGGACCAGTACACGAACCTCAGCAACCCGGGTGCGCATTACGAGTTCACTGGTCAGGAGATCTACGACCAGTGCGGCGGCAAGGTCGACATGGTAGTCATCTGCGCCGGAACCGGAGGCACGATCACCGGAGTGGCCAAGAAGCTGAAGGAGCTCATCCCCGAAGTCATCGTTGTCGGTGTAGACCCGGTCGGCAGCATCATCGCCGACCCCGAACACCCCGGCGAGCCTGTCATGTACCACGTAGAGGGAATCGGCTATGACTTTGTGCCAGACGTGTGCGAGCGTAAGTACGTCGATCGCTGGGTCAAGACACGCGATCAGGAGAGTTTCGACCTCGCCCTCGAGCTGCACCGCGAGGAGGGTCTGCTGGTCGGCGGTAGCAGCGGCTCCGCGATGGCCGGCGTGGTGGAGGCAGCCAAGGACCTTCGCTCGGATCAGCGCTGCGTCGTGCTCATGGCGGACGGCATCCGCAACTACATGGGGAAGTTCGCCGATGTCAACTGGATGATCGAGCACGGCTTCCGGCAGGGTGAGGTGACCCGCCCCACCTACGACACGCTGAAGAAGGAGCTGGAGGAGGTCAAGGCGAAGCTAGCAAAGTACGAAAGCGGCGCTCAGTAGCGGGTCGATGCTGGAACATATCTTCTCAAAATCTCACGAGGGGAAGCAGAAGACACGCGTGCCAACTTTTCCTTGGGATGCCCAACACGCTTGTGGGTGCACCGACATGTTGTGCGAGGCCTCTTTCCTGCTTCTTCCGACATGCACACAGTGCACTCGTTCCTGGGAACTGCCCACCGTCGAAAGCCATATCACTTCTTGCCTCCCGGTCGGTGTCCTCCCCTACCCCGCACCAACCGCACAATTGGGACGCGCCTTTCTCACCGAAGTCCACCTTTGCCCTCTCTTTCCCTGCTTTCGCTCTTCTTCCCCATCCTTCCACCTTCAGTGCCTCTTTCCATCACTGCCTGCGTGTCTGCCAAATCGTTCGCCCCCCCCCCTTCTCCACAGTCGACAACAACAGCCATCGCCTTCTATCATCCTCTTCTTTTTCTGGAAGCATTTTTCTTGTCCCCTTTCCCACGCATTCTCTCTTTCTCTCTCTGTTCCCCGCTGATCAT

>BPK206/0 clone 10|LinJ17_V3.0300|500 bp UPS + CDS + 500 bp DWS|cystathionine beta-synthase

GAGAGGCGGCGCGGCAGCAAGAGAGAGTGAGTGAGTGCGAAGGAATGAGGAGGAGGCAGAATGGATGGGGGCCTGGGCTAATCGAGGGGACCACCGCGTGTCTGCGTGCGAGGTACCCCCTCCCTCTCCACACATCAAAAGAAGAGAATGCTGTCATGTTGCAGCAGCTCTCCAGATTAAACCCTCCCTCTCTCCTTCTCTCTCCCGTGCCTCTACGCCATGCTCTGCCCGACGTTGCTCTTCTACCCCCACCCCGCATCATACATCCTTCTCTGTTTTTCCTTAACGTTTCCGCGACAGCTCCCTCCTTCGTTCGTCTAATCCGCAGCAGCACCTCCACCGTTGCGAGCCACTGCTGTGTCTCGTGTGGACTTACACATGTCACTGCGTCTTCATCCGTCCCCGTTACACCGTTTTTCTCCACCATCACCGCCACCGCCACCCGTTGGGATGCATCGATATGTGCCGCTTCCCCCTCCAGAACCCATTCCGTCATTTCTATGACATCTGCAACCCCCCGCGGCCAAATTCTGGCCAACGCCCTCGAGGCGGTCGGCAACACCCCTTGCATCCGCTTGAACCGCGTCCCACAGAAGCACGGCATTCAGTGCGAGGTTGTGGCCAAGTGCGAGTTCTTCAACCCTGGCGGCAGCGTCAAGGACCGCATTGGCAAGCAGATGATACTCGACGCCGAAAAGAACGGGACGCTCAAGCCGGGTTCCGTGATTGTGGAGGCCACAAGCGGCAATACTGGCATCGGTCTCTCCATGGCCGCGGCGATCCGCGGCTACCACATGGTGATTACGATGCCGAAGAAGATGTCGCACGAGAAGGAGACGACACTGCAGTCCCTTGGCGCCGAGGTGATCCGCACGGAGACGTCTCTTCCGCACGACCACCCGGACAGCCTCATCGGGGTCGCTCGCCGCCTGCGCGATGAGAAGGGCTACGTGCTGCTGGACCAGTACACGAACCTCAGCAACCCGGGTGCGCATTACGAGTTCACTGGTCAGGAGATCTACGACCAGTGCGGCGGCAAGGTCGACATGGTAGTCATCTGCGCCGGAACCGGAGGCACGATCACCGGAGTGGCCAAGAAGCTGAAGGAGCTCATCCCCGAAGTCATCGTTGTCGGTGTAGACCCGGTCGGCAGCATCATCGCCGACCCCGAACACCCCGGCGAGCCTGTCATGTACCACGTAGAGGGAATCGGCTATGACTTTGTGCCAGACGTGTGCGAGCGTAAGTACGTCGATCGCTGGGTCAAGACACGCGATCAGGAGAGTTTCGACCTCGCCCTCGAGCTGCACCGCGAGGAGGGTCTGCTGGTCGGCGGTAGCAGCGGCTCCGCGATGGCCGGCGTGGTGGAGGCAGCCAAGGACCTTCGCTCGGATCAGCGCTGCGTCGTGCTCATGGCGGACGGCATCCGCAACTACATGGGGAAGTTCGCCGATGTCAACTGGATGATCGAGCACGGCTTCCGGCAGGGTGAGGTGACCCGCCCCACCTACGACACGCTGAAGAAGGAGCTGGAGGAGGTCAAGGCGAAGCTAGCAAAGTACGAAAGCGGCGCTCAGTAGCGGGTCGATGCTGGAACATATCTTCTCAAAATCTCACGAGGGGAAGCAGAAGACACGCGTGCCAACTTTTCCTTGGGATGCCCAACACGCTTGTGGGTGCACCGACATGTTGTGCGAGGCCTCTTTCCTGCTTCTTCCGACATGCACACAGTGCACTCGTTCCTGGGAACTGCCCACCGTCGAAAGCCATATCACTTCTTGCCTCCCGGTCGGTGTCCTCCCCTACCCCGCACCAACCGCACAATTGGGACGCGCCTTTCTCACCGAAGTCCACCTTTGCCCTCTCTTTCCCTGCTTTCGCTCTTCTTCCCCATCCTTCCACCTTCAGTGCCTCTTTCCATCACTGCCTGCGTGTCTGCCAAATCGTTCGCCCCCCCCCCTTCTCCACAGTCGACAACAACAGCCATCGCCTTCTATCATCCTCTTCTTTTTCTGGAAGCATTTTTCTTGTCCCCTTTCCCACGCATTCTCTCTTTCTCTCTCTGTTCCCCGCTGATCAT

>BPK275/0 clone 18|LinJ17_V3.0300|500 bp UPS + CDS + 500 bp DWS|cystathionine beta-synthase

GAGAGGCGGCGCGGCAGCAAGAGAGAGTGAGTGAGTGCGAAGGAATGAGGAGGAGGCAGAATGGATGGGGGCCTGGGCTAATCGAGGGGACCACCGCGTGTCTGCGTGCGAGGTACCCCCTCCCTCTCCACACATCAAAAGAAGAGAATGCTGTCATGTTGCAGCAGCTCTCCAGATTAAACCCTCCCTCTCTCCTTCTCTCTCCCGTGCCTCTACGCCATGCTCTGCCCGACGTTGCTCTTCTACCCCCACCCCGCATCATACATCCTTCTCTGTTTTTCCTTAACGTTTCCGCGACAGCTCCCTCCTTCGTTCGTCTAATCCGCAGCAGCACCTCCACCGTTGCGAGCCACTGCTGTGTCTCGTGTGGACTTACACATGTCACTGCGTCTTCATCCGTCCCCGTTACACCGTTTTTCTCCACCATCACCGCCACCGCCACCCGTTGGGATGCATCGATATGTGCCGCTTCCCCCTCCAGAACCCATTCCGTCATTTCTATGACATCTGCAACCCCCCGCGGCCAAATTCTGGCCAACGCCCTCGAGGCGGTCGGCAACACCCCTTGCATCCGCTTGAACCGCGTCCCACAGAAGCACGGCATTCAGTGCGAGGTTGTGGCCAAGTGCGAGTTCTTCAACCCTGGCGGCAGCGTCAAGGACCGCATTGGCAAGCAGATGATACTCGACGCCGAAAAGAACGGGACGCTCAAGCCGGGTTCCGTGATTGTGGAGGCCACAAGCGGCAATACTGGCATCGGTCTCTCCATGGCCGCGGCGATCCGCGGCTACCACATGGTGATTACGATGCCGAAGAAGATGTCGCACGAGAAGGAGACGACACTGCAGTCCCTTGGCGCCGAGGTGATCCGCACGGAGACGTCTCTTCCGCACGACCACCCGGACAGCCTCATCGGGGTCGCTCGCCGCCTGCGCGATGAGAAGGGCTACGTGCTGCTGGACCAGTACACGAACCTCAGCAACCCGGGTGCGCATTACGAGTTCACTGGTCAGGAGATCTACGACCAGTGCGGCGGCAAGGTCGACATGGTAGTCATCTGCGCCGGAACCGGAGGCACGATCACCGGAGTGGCCAAGAAGCTGAAGGAGCTCATCCCCGAAGTCATCGTTGTCGGTGTAGACCCGGTCGGCAGCATCATCGCCGACCCCGAACACCCCGGCGAGCCTGTCATGTACCACGTAGAGGGAATCGGCTATGACTTTGTGCCAGACGTGTGCGAGCGTAAGTACGTCGATCGCTGGGTCAAGACACGCGATCAGGAGAGTTTCGACCTCGCCCTCGAGCTGCACCGCGAGGAGGGTCTGCTGGTCGGCGGTAGCAGCGGCTCCGCGATGGCCGGCGTGGTGGAGGCAGCCAAGGACCTTCGCTCGGATCAGCGCTGCGTCGTGCTCATGGCGGACGGCATCCGCAACTACATGGGGAAGTTCGCCGATGTCAACTGGATGATCGAGCACGGCTTCCGGCAGGGTGAGGTGACCCGCCCCACCTACGACACGCTGAAGAAGGAGCTGGAGGAGGTCAAGGCGAAGCTAGCAAAGTACGAAAGCGGCGCTCAGTAGCGGGTCGATGCTGGAACATATCTTCTCAAAATCTCACGAGGGGAAGCAGAAGACACGCGTGCCAACTTTTCCTTGGGATGCCCAACACGCTTGTGGGTGCACCGACATGTTGTGCGAGGCCTCTTTCCTGCTTCTTCCGACATGCACACAGTGCACTCGTTCCTGGGAACTGCCCACCGTCGAAAGCCATATCACTTCTTGCCTCCCGGTCGGTGTCCTCCCCTACCCCGCACCAACCGCACAATTGGGACGCGCCTTTCTCACCGAAGTCCACCTTTGCCCTCTCTTTCCCTGCTTTCGCTCTTCTTCCCCATCCTTCCACCTTCAGTGCCTCTTTCCATCACTGCCTGCGTGTCTGCCAAATCGTTCGCCCCCCCCCCTTCTCCACAGTCGACAACAACAGCCATCGCCTTCTATCATCCTCTTCTTTTTCTGGAAGCATTTTTCTTGTCCCCTTTCCCACGCATTCTCTCTTTCTCTCTCTGTTCCCCGCTGATCAT

>BPK282/0 clone 4|LinJ17_V3.0300|500 bp UPS + CDS + 500 bp DWS|cystathionine beta-synthase

GAGAGGCGGCGCGGCAGCAAGAGAGAGTGAGTGAGTGCGAAGGAATGAGGAGGAGGCAGAATGGATGGGGGCCTGGGCTAATCGAGGGGACCACCGCGTGTCTGCGTGCGAGGTACCCCCTCCCTCTCCACACATCAAAAGAAGAGAATGCTGTCATGTTGCAGCAGCTCTCCAGATTAAACCCTCCCTCTCTCCTTCTCTCTCCCGTGCCTCTACGCCATGCTCTGCCCGACGTTGCTCTTCTACCCCCACCCCGCATCATACATCCTTCTCTGTTTTTCCTTAACGTTTCCGCGACAGCTCCCTCCTTCGTTCGTCTAATCCGCAGCAGCACCTCCACCGTTGCGAGCCACTGCTGTGTCTCGTGTGGACTTACACATGTCACTGCGTCTTCATCCGTCCCCGTTACACCGTTTTTCTCCACCATCACCGCCACCGCCACCCGTTGGGATGCATCGATATGTGCCGCTTCCCCCTCCAGAACCCATTCCGTCATTTCTATGACATCTGCAACCCCCCGCGGCCAAATTCTGGCCAACGCCCTCGAGGCGGTCGGCAACACCCCTTGCATCCGCTTGAACCGCGTCCCACAGAAGCACGGCATTCAGTGCGAGGTTGTGGCCAAGTGCGAGTTCTTCAACCCTGGCGGCAGCGTCAAGGACCGCATTGGCAAGCAGATGATACTCGACGCCGAAAAGAACGGGACGCTCAAGCCGGGTTCCGTGATTGTGGAGGCCACAAGCGGCAATACTGGCATCGGTCTCTCCATGGCCGCGGCGATCCGCGGCTACCACATGGTGATTACGATGCCGAAGAAGATGTCGCACGAGAAGGAGACGACACTGCAGTCCCTTGGCGCCGAGGTGATCCGCACGGAGACGTCTCTTCCGCACGACCACCCGGACAGCCTCATCGGGGTCGCTCGCCGCCTGCGCGATGAGAAGGGCTACGTGCTGCTGGACCAGTACACGAACCTCAGCAACCCGGGTGCGCATTACGAGTTCACTGGTCAGGAGATCTACGACCAGTGCGGCGGCAAGGTCGACATGGTAGTCATCTGCGCCGGAACCGGAGGCACGATCACCGGAGTGGCCAAGAAGCTGAAGGAGCTCATCCCCGAAGTCATCGTTGTCGGTGTAGACCCGGTCGGCAGCATCATCGCCGACCCCGAACACCCCGGCGAGCCTGTCATGTACCACGTAGAGGGAATCGGCTATGACTTTGTGCCAGACGTGTGCGAGCGTAAGTACGTCGATCGCTGGGTCAAGACACGCGATCAGGAGAGTTTCGACCTCGCCCTCGAGCTGCACCGCGAGGAGGGTCTGCTGGTCGGCGGTAGCAGCGGCTCCGCGATGGCCGGCGTGGTGGAGGCAGCCAAGGACCTTCGCTCGGATCAGCGCTGCGTCGTGCTCATGGCGGACGGCATCCGCAACTACATGGGGAAGTTCGCCGATGTCAACTGGATGATCGAGCACGGCTTCCGGCAGGGTGAGGTGACCCGCCCCACCTACGACACGCTGAAGAAGGAGCTGGAGGAGGTCAAGGCGAAGCTAGCAAAGTACGAAAGCGGCGCTCAGTAGCGGGTCGATGCTGGAACATATCTTCTCAAAATCTCACGAGGGGAAGCAGAAGACACGCGTGCCAACTTTTCCTTGGGATGCCCAACACGCTTGTGGGTGCACCGACATGTTGTGCGAGGCCTCTTTCCTGCTTCTTCCGACATGCACACAGTGCACTCGTTCCTGGGAACTGCCCACCGTCGAAAGCCATATCACTTCTTGCCTCCCGGTCGGTGTCCTCCCCTACCCCGCACCAACCGCACAATTGGGACGCGCCTTTCTCACCGAAGTCCACCTTTGCCCTCTCTTTCCCTGCTTTCGCTCTTCTTCCCCATCCTTCCACCTTCAGTGCCTCTTTCCATCACTGCCTGCGTGTCTGCCAAATCGTTCGCCCCCCCCCCTTCTCCACAGTCGACAACAACAGCCATCGCCTTCTATCATCCTCTTCTTTTTCTGGAAGCATTTTTCTTGTCCCCTTTCCCACGCATTCTCTCTTTCTCTCTCTGTTCCCCGCTGATCAT

>BPK294/0 clone 1|LinJ17_V3.0300|500 bp UPS + CDS + 500 bp DWS|cystathionine beta-synthase

GAGAGGCGGCGCGGCAGCAAGAGAGAGTGAGTGAGTGCGAAGGAATGAGGAGGAGGCAGAATGGATGGGGGCCTGGGCTAATCGAGGGGACCACCGCGTGTCTGCGTGCGAGGTACCCCCTCCCTCTCCACACATCAAAAGAAGAGAATGCTGTCATGTTGCAGCAGCTCTCCAGATTAAACCCTCCCTCTCTCCTTCTCTCTCCCGTGCCTCTACGCCATGCTCTGCCCGACGTTGCTCTTCTACCCCCACCCCGCATCATACATCCTTCTCTGTTTTTCCTTAACGTTTCCGCGACAGCTCCCTCCTTCGTTCGTCTAATCCGCAGCAGCACCTCCACCGTTGCGAGCCACTGCTGTGTCTCGTGTGGACTTACACATGTCACTGCGTCTTCATCCGTCCCCGTTACACCGTTTTTCTCCACCATCACCGCCACCGCCACCCGTTGGGATGCATCGATATGTGCCGCTTCCCCCTCCAGAACCCATTCCGTCATTTCTATGACATCTGCAACCCCCCGCGGCCAAATTCTGGCCAACGCCCTCGAGGCGGTCGGCAACACCCCTTGCATCCGCTTGAACCGCGTCCCACAGAAGCACGGCATTCAGTGCGAGGTTGTGGCCAAGTGCGAGTTCTTCAACCCTGGCGGCAGCGTCAAGGACCGCATTGGCAAGCAGATGATACTCGACGCCGAAAAGAACGGGACGCTCAAGCCGGGTTCCGTGATTGTGGAGGCCACAAGCGGCAATACTGGCATCGGTCTCTCCATGGCCGCGGCGATCCGCGGCTACCACATGGTGATTACGATGCCGAAGAAGATGTCGCACGAGAAGGAGACGACACTGCAGTCCCTTGGCGCCGAGGTGATCCGCACGGAGACGTCTCTTCCGCACGACCACCCGGACAGCCTCATCGGGGTCGCTCGCCGCCTGCGCGATGAGAAGGGCTACGTGCTGCTGGACCAGTACACGAACCTCAGCAACCCGGGTGCGCATTACGAGTTCACTGGTCAGGAGATCTACGACCAGTGCGGCGGCAAGGTCGACATGGTAGTCATCTGCGCCGGAACCGGAGGCACGATCACCGGAGTGGCCAAGAAGCTGAAGGAGCTCATCCCCGAAGTCATCGTTGTCGGTGTAGACCCGGTCGGCAGCATCATCGCCGACCCCGAACACCCCGGCGAGCCTGTCATGTACCACGTAGAGGGAATCGGCTATGACTTTGTGCCAGACGTGTGCGAGCGTAAGTACGTCGATCGCTGGGTCAAGACACGCGATCAGGAGAGTTTCGACCTCGCCCTCGAGCTGCACCGCGAGGAGGGTCTGCTGGTCGGCGGTAGCAGCGGCTCCGCGATGGCCGGCGTGGTGGAGGCAGCCAAGGACCTTCGCTCGGATCAGCGCTGCGTCGTGCTCATGGCGGACGGCATCCGCAACTACATGGGGAAGTTCGCCGATGTCAACTGGATGATCGAGCACGGCTTCCGGCAGGGTGAGGTGACCCGCCCCACCTACGACACGCTGAAGAAGGAGCTGGAGGAGGTCAAGGCGAAGCTAGCAAAGTACGAAAGCGGCGCTCAGTAGCGGGTCGATGCTGGAACATATCTTCTCAAAATCTCACGAGGGGAAGCAGAAGACACGCGTGCCAACTTTTCCTTGGGATGCCCAACACGCTTGTGGGTGCACCGACATGTTGTGCGAGGCCTCTTTCCTGCTTCTTCCGACATGCACACAGTGCACTCGTTCCTGGGAACTGCCCACCGTCGAAAGCCATATCACTTCTTGCCTCCCGGTCGGTGTCCTCCCCTACCCCGCACCAACCGCACAATTGGGACGCGCCTTTCTCACCGAAGTCCACCTTTGCCCTCTCTTTCCCTGCTTTCGCTCTTCTTCCCCATCCTTCCACCTTCAGTGCCTCTTTCCATCACTGCCTGCGTGTCTGCCAAATCGTTCGCCCCCCCCCCTTCTCCACAGTCGACAACAACAGCCATCGCCTTCTATCATCCTCTTCTTTTTCTGGAAGCATTTTTCTTGTCCCCTTTCCCACGCATTCTCTCTTTCTCTCTCTGTTCCCCGCTGATCAT

>BPK298/0 clone 8|LinJ17_V3.0300|500 bp UPS + CDS + 500 bp DWS|cystathionine beta-synthase

GAGAGGCGGCGCGGCAGCAAGAGAGAGTGAGTGAGTGCGAAGGAATGAGGAGGAGGCAGAATGGATGGGGGCCTGGGCTAATCGAGGGGACCACCGCGTGTCTGCGTGCGAGGTACCCCCTCCCTCTCCACACATCAAAAGAAGAGAATGCTGTCATGTTGCAGCAGCTCTCCAGATTAAACCCTCCCTCTCTCCTTCTCTCTCCCGTGCCTCTACGCCATGCTCTGCCCGACGTTGCTCTTCTACCCCCACCCCGCATCATACATCCTTCTCTGTTTTTCCTTAACGTTTCCGCGACAGCTCCCTCCTTCGTTCGTCTAATCCGCAGCAGCACCTCCACCGTTGCGAGCCACTGCTGTGTCTCGTGTGGACTTACACATGTCACTGCGTCTTCATCCGTCCCCGTTACACCGTTTTTCTCCACCATCACCGCCACCGCCACCCGTTGGGATGCATCGATATGTGCCGCTTCCCCCTCCAGAACCCATTCCGTCATTTCTATGACATCTGCAACCCCCCGCGGCCAAATTCTGGCCAACGCCCTCGAGGCGGTCGGCAACACCCCTTGCATCCGCTTGAACCGCGTCCCACAGAAGCACGGCATTCAGTGCGAGGTTGTGGCCAAGTGCGAGTTCTTCAACCCTGGCGGCAGCGTCAAGGACCGCATTGGCAAGCAGATGATACTCGACGCCGAAAAGAACGGGACGCTCAAGCCGGGTTCCGTGATTGTGGAGGCCACAAGCGGCAATACTGGCATCGGTCTCTCCATGGCCGCGGCGATCCGCGGCTACCACATGGTGATTACGATGCCGAAGAAGATGTCGCACGAGAAGGAGACGACACTGCAGTCCCTTGGCGCCGAGGTGATCCGCACGGAGACGTCTCTTCCGCACGACCACCCGGACAGCCTCATCGGGGTCGCTCGCCGCCTGCGCGATGAGAAGGGCTACGTGCTGCTGGACCAGTACACGAACCTCAGCAACCCGGGTGCGCATTACGAGTTCACTGGTCAGGAGATCTACGACCAGTGCGGCGGCAAGGTCGACATGGTAGTCATCTGCGCCGGAACCGGAGGCACGATCACCGGAGTGGCCAAGAAGCTGAAGGAGCTCATCCCCGAAGTCATCGTTGTCGGTGTAGACCCGGTCGGCAGCATCATCGCCGACCCCGAACACCCCGGCGAGCCTGTCATGTACCACGTAGAGGGAATCGGCTATGACTTTGTGCCAGACGTGTGCGAGCGTAAGTACGTCGATCGCTGGGTCAAGACACGCGATCAGGAGAGTTTCGACCTCGCCCTCGAGCTGCACCGCGAGGAGGGTCTGCTGGTCGGCGGTAGCAGCGGCTCCGCGATGGCCGGCGTGGTGGAGGCAGCCAAGGACCTTCGCTCGGATCAGCGCTGCGTCGTGCTCATGGCGGACGGCATCCGCAACTACATGGGGAAGTTCGCCGATGTCAACTGGATGATCGAGCACGGCTTCCGGCAGGGTGAGGTGACCCGCCCCACCTACGACACGCTGAAGAAGGAGCTGGAGGAGGTCAAGGCGAAGCTAGCAAAGTACGAAAGCGGCGCTCAGTAGCGGGTCGATGCTGGAACATATCTTCTCAAAATCTCACGAGGGGAAGCAGAAGACACGCGTGCCAACTTTTCCTTGGGATGCCCAACACGCTTGTGGGTGCACCGACATGTTGTGCGAGGCCTCTTTCCTGCTTCTTCCGACATGCACACAGTGCACTCGTTCCTGGGAACTGCCCACCGTCGAAAGCCATATCACTTCTTGCCTCCCGGTCGGTGTCCTCCCCTACCCCGCACCAACCGCACAATTGGGACGCGCCTTTCTCACCGAAGTCCACCTTTGCCCTCTCTTTCCCTGCTTTCGCTCTTCTTCCCCATCCTTCCACCTTCAGTGCCTCTTTCCATCACTGCCTGCGTGTCTGCCAAATCGTTCGCCCCCCCCCCTTCTCCACAGTCGACAACAACAGCCATCGCCTTCTATCATCCTCTTCTTTTTCTGGAAGCATTTTTCTTGTCCCCTTTCCCACGCATTCTCTCTTTCTCTCTCTGTTCCCCGCTGATCAT

### 2. GAMMA-GLUTAMYLCYSTEINE SYNTHASE

>BPK035/0 clone 1|LinJ18_V3.1660|500 bp UPS + CDS + 500 bp DWS|Gamma-glutamylcysteine synthase

GCTTCGGCACTTCCCAGCTGCTTTCGTCCCTCGGCTGCCCAAGCCGCTGTGCGCCATAACACACCCGTATACATTGTCATACGCACTCCTTCTTCACCTTTACGCGGCCGCACCTTCTCGTCTAACCAACCACGGCAACTACTTTGCTTCTCTATCTTCGGCCCTTATTATTATTATCATCCGCGCAGCGCCTTCACCGCGTCTGTTTTTCGTCACCTCTTCTCCCTGCGTTTTCCCTCGCGCTTTCAAATTCCCTAAATCGTGTTTCTCTCTGACCCGCTCTCCGATACACTCCCGTCCACCCTCCCTGATTCTCTTTTTTCTTGTGTTTGCATCTCTTTGCCCGCTCTCCTACTACGCCGTTTCCCTCCCTCGCAGCCACTCGCCGCAGTCCCTCTGCGTGCCTCTCTCTCTCTATCTCGGCAGTCTTGCTACCCCTTCTTTCCTTCTGACGTCACTAGACGCGCACTCGCATCCTCGAAGGAGATCCGCTTGGCAAAATGGGGCTCTTGACGACTGGCGGCGCCCCGATACAGTGGGGCACCGATGCAAATAGAAAGGCCATTCCGCACGTCAGAGAGCACGGCATTCAGCAGTTCCTCAACGTTTTCAAGAACAAAAAGGACCTCCATGGTATGCCGTTTCTCTGGGGAGAGGAGCTGGAGCACCAGCTAATCCAGATCCACGATAACACGGTTACCCTCAGCACGGAAAGTGCGATGGTAATGAACAAGCTGAGGGCGCGTCCTGACAACTGCGCCGTGTGGAACCCCGAATATGGAAGCTTCATGATCGAAAGCACGCCAGACCACCCGTACAGTCTGTCGGTGGAGAGCCTCGACTCGGTGCAGGACAACATCGAGCGGCGGTACGACATGCTCAACAAGGAGGCACCACCCGGCGTGGTCGGCACCACCTTTGTGACTTTCCCACTCATGGGCCAGGGCAACTTTGTCCACTGCAGTGATAAGAGTTCTCCGTACTCGCAGTCGCTTTTTGTTCCTGATGCGTGCATCAACCAAACGCATCCGCGCTTCGCGAACCTGACGGCAAACATTCGCCTGCGCCGCGGTCAAAAGGTTTGCGTCCTGGTGCCTCTGTACATGGACTCCCGTACAATGCAGGACACGGTGGACCCCCAACTAAACATTGACCTGACTCCACACAACAAGGACATTTTTTACTCCATGAGAGAAAACGGCAGGAACATGACCGACGAACTCTACGCGGAGACGGACGCGTCTGCCGCTCTGCTAGTGCCCAGCAGCTCTCTCGATCCACGCGAGGACTACCCTGTCACCGAGACGCTGAAGCAGCTCTTCACCCCTGCTACGCTCTACTACTACGCACAGTACTTCACGGGACAGCGCCGCGAGCATATGCAGGAACGCTACAACGCGTGTAACTGCCCCGTAACCTTGGTCAGCCACCCGTGCATCTACATGGACTGCATGGCCTTTGGCATGGGTAACAGCGCTCTGCAAGTGACGATGCAGCTGGACAACATTCACGAGGCGCGCCACGTGTACGACCAGCTCGCCATCTTGTGCCCGGCATTTCTGGCTCTCAGCTCAGCCACGCCGTTCCAAAAGGGTCTTCTTTGCGACACCGATGTGCGCTGGCTGACTATCGCCGGCGCTGTCGACGACCGCCGCGTGGAGGAGGTGCCGCGTATTCTCAAGTCTCGCTACGACTCCATCTCCGTCTTCATCAGCGACAGAACCGAAAACCTCGAGGAATTCAACGATTCACAAATAGCGATAAACCGCTCGTACTGTGAACTTCTGAAGGACTCCGGTGTGGACGTGCGGTTGGCGAACCACATTGCACATTTGTTCATTCGAGATCCGCTTGTGATGTACGACAAGATGATCGACATCGATGACACGACGCACACGGAGCACTTTGACAACATCCAGTCCACTAACTGGCAGACAGTGCGCTTCAAGCCTCCGCCGATAGGAAACGACATTGGCTGGCGCGTTGAGTTCCGCGTGATGGATATTCAGCCAACACCGTTCGAAAACGCCGCCTTCGCCGTCTTCATTCCGCTTCTCACCAAGGCCATCATCACCTACAAGCCCTGCTTTTACACCAAGATCTCCATCGTCGACGAGAATATGGGCCGCGCACATCGCATCAACCCGTGTGGAGAACAATACATTATGCGCAAGGACATTTTCGCCGACAAGTGCACCGCCAGCGACGAGGAGACGGCGAGGATGAGCATTGACGAGATCTTCAACGGCAAGGAGGGCGGCTTCTATGGACTCATCCCCCTCGTGTGCCGCTATCTAGACGACGAGGGGAAGCGAAGTCCCCTCGTAAACTCCTACCTGAAGTTCCTGTCAATGCGCGCCTCTGGCCGCATTCCCACACCTGCGCAGTACATGCGCAAGTTTGTCACGACACACCCCGACTACAAGCACGACTCACGCCTCACCGACAGCATCGCGCGTGACCTTGTGCAGCGCATGCACGGCCTGGCTGCGAATCAGATCCACGACGATGACTACCTTCCCATGAGCTTTTTCACGGCCGATACAGTAGAGAGCACCAAGTGAGTCTTTCTTCTCATTCCCACGGACTGCAGAACGAAAGCGCCCAAGGCGTATGCTGCGTTCAGTCCCGCGCATGTGCGCTAAAGTCGACCCCGCTTTAAGCTATTTACTGAAGAACCACCCCTCCCCCAAAACAAAAAATAAAGAAAGCATTCCAGACAAAAAAAACAACAATATGGACCGTGTCTCACTTTTGCCTTCCTTCTTTTTTCCTGTGCTTTGCCTGCTGGCTTACGTCCGCGGCGGAGCAGCAACGTTGGCGCATACAGCAGCAGTTCTCTCCCTCGTTTCTTCTGCTGATTGTGCGCTTACTCTGTGCTGCTGCGCCATGCCTATTCTGATTTGTCTCGTATCCAGCGCTGTATTCCCTCTCTTTTCCCTATTGCCCTCATGNNNNNNNNNNNNNNNNNNNNNNNNNNNNNNNNNNNNNNNNNNNNNNNNNNNNNNNNNNNNNNNNNNNNNNNNNNNNNNTGCCCTCTGGTGCGGCTGCGGGCTCTCTGGCG

>BPK043/0 clone 2|LinJ18_V3.1660|500 bp UPS + CDS + 500 bp DWS|Gamma-glutamylcysteine synthase

GCTTCGGCACTTCCCAGCTGCTTTCGTCCCTCGGCTGCCCAAGCCGCTGTGCGCCATAACACACCCGTATACATTGTCATACGCACTCCTTCTTCACCTTTACGCGGCCGCACCTTCTCGTCTAACCAACCACGGCAACTACTTTGCTTCTCTATCTTCGGCCCTTATTATTATTATCATCCGCGCAGCGCCTTCACCGCGTCTGTTTTTCGTCACCTCTTCTCCCTGCGTTTTCCCTCGCGCTTTCAAATTCCCTAAATCGTGTTTCTCTCTGACCCGCTCTCCGATACACTCCCGTCCACCCTCCCTGATTCTCTTTTTTCTTGTGTTTGCATCTCTTTGCCCGCTCTCCTACTACGCCGTTTCCCTCCCTCGCAGCCACTCGCCGCAGTCCCTCTGCGTGCCTCTCTCTCTCTATCTCGGCAGTCTTGCTACCCCTTCTTTCCTTCTGACGTCACTAGACGCGCACTCGCATCCTCGAAGGAGATCCGCTTGGCAAAATGGGGCTCTTGACGACTGGCGGCGCCCCGATACAGTGGGGCACCGATGCAAATAGAAAGGCCATTCCGCACGTCAGAGAGCACGGCATTCAGCAGTTCCTCAACGTTTTCAAGAACAAAAAGGACCTCCATGGTATGCCGTTTCTCTGGGGAGAGGAGCTGGAGCACCAGCTAATCCAGATCCACGATAACACGGTTACCCTCAGCACGGAAAGTGCGATGGTAATGAACAAGCTGAGGGCGCGTCCTGACAACTGCGCCGTGTGGAACCCCGAATATGGAAGCTTCATGATCGAAAGCACGCCAGACCACCCGTACAGTCTGTCGGTGGAGAGCCTCGACTCGGTGCAGGACAACATCGAGCGGCGGTACGACATGCTCAACAAGGAGGCACCACCCGGCGTGGTCGGCACCACCTTTGTGACTTTCCCACTCATGGGCCAGGGCAACTTTGTCCACTGCAGTGATAAGAGTTCTCCGTACTCGCAGTCGCTTTTTGTTCCTGATGCGTGCATCAACCAAACGCATCCGCGCTTCGCGAACCTGACGGCAAACATTCGCCTGCGCCGCGGTCAAAAGGTTTGCGTCCTGGTGCCTCTGTACATGGACTCCCGTACAATGCAGGACACGGTGGACCCCCAACTAAACATTGACCTGACTCCACACAACAAGGACATTTTTTACTCCATGAGAGAAAACGGCAGGAACATGACCGACGAACTCTACGCGGAGACGGACGCGTCTGCCGCTCTGCTAGTGCCCAGCAGCTCTCTCGATCCACGCGAGGACTACCCTGTCACCGAGACGCTGAAGCAGCTCTTCACCCCTGCTACGCTCTACTACTACGCACAGTACTTCACGGGACAGCGCCGCGAGCATATGCAGGAACGCTACAACGCGTGTAACTGCCCCGTAACCTTGGTCAGCCACCCGTGCATCTACATGGACTGCATGGCCTTTGGCATGGGTAACAGCGCTCTGCAAGTGACGATGCAGCTGGACAACATTCACGAGGCGCGCCACGTGTACGACCAGCTCGCCATCTTGTGCCCGGCATTTCTGGCTCTCAGCTCAGCCACGCCGTTCCAAAAGGGTCTTCTTTGCGACACCGATGTGCGCTGGCTGACTATCGCCGGCGCTGTCGACGACCGCCGCGTGGAGGAGGTGCCGCGTATTCTCAAGTCTCGCTACGACTCCATCTCCGTCTTCATCAGCGACAGAACCGAAAACCTCGAGGAATTCAACGATTCACAAATAGCGATAAACCGCTCGTACTGTGAACTTCTGAAGGACTCCGGTGTGGACGTGCGGTTGGCGAACCACATTGCACATTTGTTCATTCGAGATCCGCTTGTGATGTACGACAAGATGATCGACATCGATGACACGACGCACACGGAGCACTTTGACAACATCCAGTCCACTAACTGGCAGACAGTGCGCTTCAAGCCTCCGCCGATAGGAAACGACATTGGCTGGCGCGTTGAGTTCCGCGTGATGGATATTCAGCCAACACCGTTCGAAAACGCCGCCTTCGCCGTCTTCATTCCGCTTCTCACCAAGGCCATCATCACCTACAAGCCCTGCTTTTACACCAAGATCTCCATCGTCGACGAGAATATGGGCCGCGCACATCGCATCAACCCGTGTGGAGAACAATACATTATGCGCAAGGACATTTTCGCCGACAAGTGCACCGCCAGCGACGAGGAGACGGCGAGGATGAGCATTGACGAGATCTTCAACGGCAAGGAGGGCGGCTTCTATGGACTCATCCCCCTCGTGTGCCGCTATCTAGACGACGAGGGGAAGCGAAGTCCCCTCGTAAACTCCTACCTGAAGTTCCTGTCAATGCGCGCCTCTGGCCGCATTCCCACACCTGCGCAGTACATGCGCAAGTTTGTCACGACACACCCCGACTACAAGCACGACTCACGCCTCACCGACAGCATCGCGCGTGACCTTGTGCAGCGCATGCACGGCCTGGCTGCGAATCAGATCCACGACGATGACTACCTTCCCATGAGCTTTTTCACGGCCGATACAGTAGAGAGCACCAAGTGAGTCTTTCTTCTCATTCCCACGGACTGCAGAACGAAAGCGCCCAAGGCGTATGCTGCGTTCAGTCCCGCGCATGTGCGCTAAAGTCGACCCCGCTTTAAGCTATTTACTGAAGAACCACCCCTCCCCCAAAACAAAAAATAAAGAAAGCATTCCAGACAAAAAAAACAACAATATGGACCGTGTCTCACTTTTGCCTTCCTTCTTTTTTCCTGTGCTTTGCCTGCTGGCTTACGTCCGCGGCGGAGCAGCAACGTTGGCGCATACAGCAGCAGTTCTCTCCCTCGTTTCTTCTGCTGATTGTGCGCTTACTCTGTGCTGCTGCGCCATGCCTATTCTGATTTGTCTCGTATCCAGCGCTGTATTCCCTCTCTTTTCCCTATTGCCCTCATGNNNNNNNNNNNNNNNNNNNNNNNNNNNNNNNNNNNNNNNNNNNNNNNNNNNNNNNNNNNNNNNNNNNNNNNNNNNNNNTGCCCTCTGGTGCGGCTGCGGGCTCTCTGGCG

>BPK085/0 clone 8|LinJ18_V3.1660|500 bp UPS + CDS + 500 bp DWS|Gamma-glutamylcysteine synthase

GCTTCGGCACTTCCCAGCTGCTTTCGTCCCTCGGCTGCCCAAGCCGCTGTGCGCCATAACACACCCGTATACATTGTCATACGCACTCCTTCTTCACCTTTACGCGGCCGCACCTTCTCGTCTAACCAACCACGGCAACTACTTTGCTTCTCTATCTTCGGCCCTTATTATTATTATCATCCGCGCAGCGCCTTCACCGCGTCTGTTTTTCGTCACCTCTTCTCCCTGCGTTTTCCCTCGCGCTTTCAAATTCCCTAAATCGTGTTTCTCTCTGACCCGCTCTCCGATACACTCCCGTCCACCCTCCCTGATTCTCTTTTTTCTTGTGTTTGCATCTCTTTGCCCGCTCTCCTACTACGCCGTTTCCCTCCCTCGCAGCCACTCGCCGCAGTCCCTCTGCGTGCCTCTCTCTCTCTATCTCGGCAGTCTTGCTACCCCTTCTTTCCTTCTGACGTCACTAGACGCGCACTCGCATCCTCGAAGGAGATCCGCTTGGCAAAATGGGGCTCTTGACGACTGGCGGCGCCCCGATACAGTGGGGCACCGATGCAAATAGAAAGGCCATTCCGCACGTCAGAGAGCACGGCATTCAGCAGTTCCTCAACGTTTTCAAGAACAAAAAGGACCTCCATGGTATGCCGTTTCTCTGGGGAGAGGAGCTGGAGCACCAGCTAATCCAGATCCACGATAACACGGTTACCCTCAGCACGGAAAGTGCGATGGTAATGAACAAGCTGAGGGCGCGTCCTGACAACTGCGCCGTGTGGAACCCCGAATATGGAAGCTTCATGATCGAAAGCACGCCAGACCACCCGTACAGTCTGTCGGTGGAGAGCCTCGACTCGGTGCAGGACAACATCGAGCGGCGGTACGACATGCTCAACAAGGAGGCACCACCCGGCGTGGTCGGCACCACCTTTGTGACTTTCCCACTCATGGGCCAGGGCAACTTTGTCCACTGCAGTGATAAGAGTTCTCCGTACTCGCAGTCGCTTTTTGTTCCTGATGCGTGCATCAACCAAACGCATCCGCGCTTCGCGAACCTGACGGCAAACATTCGCCTGCGCCGCGGTCAAAAGGTTTGCGTCCTGGTGCCTCTGTACATGGACTCCCGTACAATGCAGGACACGGTGGACCCCCAACTAAACATTGACCTGACTCCACACAACAAGGACATTTTTTACTCCATGAGAGAAAACGGCAGGAACATGACCGACGAACTCTACGCGGAGACGGACGCGTCTGCCGCTCTGCTAGTGCCCAGCAGCTCTCTCGATCCACGCGAGGACTACCCTGTCACCGAGACGCTGAAGCAGCTCTTCACCCCTGCTACGCTCTACTACTACGCACAGTACTTCACGGGACAGCGCCGCGAGCATATGCAGGAACGCTACAACGCGTGTAACTGCCCCGTAACCTTGGTCAGCCACCCGTGCATCTACATGGACTGCATGGCCTTTGGCATGGGTAACAGCGCTCTGCAAGTGACGATGCAGCTGGACAACATTCACGAGGCGCGCCACGTGTACGACCAGCTCGCCATCTTGTGCCCGGCATTTCTGGCTCTCAGCTCAGCCACGCCGTTCCAAAAGGGTCTTCTTTGCGACACCGATGTGCGCTGGCTGACTATCGCCGGCGCTGTCGACGACCGCCGCGTGGAGGAGGTGCCGCGTATTCTCAAGTCTCGCTACGACTCCATCTCCGTCTTCATCAGCGACAGAACCGAAAACCTCGAGGAATTCAACGATTCACAAATAGCGATAAACCGCTCGTACTGTGAACTTCTGAAGGACTCCGGTGTGGACGTGCGGTTGGCGAACCACATTGCACATTTGTTCATTCGAGATCCGCTTGTGATGTACGACAAGATGATCGACATCGATGACACGACGCACACGGAGCACTTTGACAACATCCAGTCCACTAACTGGCAGACAGTGCGCTTCAAGCCTCCGCCGATAGGAAACGACATTGGCTGGCGCGTTGAGTTCCGCGTGATGGATATTCAGCCAACACCGTTCGAAAACGCCGCCTTCGCCGTCTTCATTCCGCTTCTCACCAAGGCCATCATCACCTACAAGCCCTGCTTTTACACCAAGATCTCCATCGTCGACGAGAATATGGGCCGCGCACATCGCATCAACCCGTGTGGAGAACAATACATTATGCGCAAGGACATTTTCGCCGACAAGTGCACCGCCAGCGACGAGGAGACGGCGAGGATGAGCATTGACGAGATCTTCAACGGCAAGGAGGGCGGCTTCTATGGACTCATCCCCCTCGTGTGCCGCTATCTAGACGACGAGGGGAAGCGAAGTCCCCTCGTAAACTCCTACCTGAAGTTCCTGTCAATGCGCGCCTCTGGCCGCATTCCCACACCTGCGCAGTACATGCGCAAGTTTGTCACGACACACCCCGACTACAAGCACGACTCACGCCTCACCGACAGCATCGCGCGTGACCTTGTGCAGCGCATGCACGGCCTGGCTGCGAATCAGATCCACGACGATGACTACCTTCCCATGAGCTTTTTCACGGCCGATACAGTAGAGAGCACCAAGTGAGTCTTTCTTCTCATTCCCACGGACTGCAGAACGAAAGCGCCCAAGGCGTATGCTGCGTTCAGTCCCGCGCATGTGCGCTAAAGTCGACCCCGCTTTAAGCTATTTACTGAAGAACCACCCCTCCCCCAAAACAAAAAATAAAGAAAGCATTCCAGACAAAAAAAACAACAATATGGACCGTGTCTCACTTTTGCCTTCCTTCTTTTTTCCTGTGCTTTGCCTGCTGGCTTACGTCCGCGGCGGAGCAGCAACGTTGGCGCATACAGCAGCAGTTCTCTCCCTCGTTTCTTCTGCTGATTGTGCGCTTACTCTGTGCTGCTGCGCCATGCCTATTCTGATTTGTCTCGTATCCAGCGCTGTATTCCCTCTCTTTTCCCTATTGCCCTCATGNNNNNNNNNNNNNNNNNNNNNNNNNNNNNNNNNNNNNNNNNNNNNNNNNNNNNNNNNNNNNNNNNNNNNNNNNNNNNNTGCCCTCTGGTGCGGCTGCGGGCTCTCTGGCG

>BPK087/0 clone 11|LinJ18_V3.1660|500 bp UPS + CDS + 500 bp DWS|Gamma-glutamylcysteine synthase

GCTTCGGCACTTCCCAGCTGCTTTCGTCCCTCGGCTGCCCAAGCCGCTGTGCGCCATAACACACCCGTATACATTGTCATACGCACTCCTTCTTCACCTTTACGCGGCCGCACCTTCTCGTCTAACCAACCACGGCAACTACTTTGCTTCTCTATCTTCGGCCCTTATTATTATTATCATCCGCGCAGCGCCTTCACCGCGTCTGTTTTTCGTCACCTCTTCTCCCTGCGTTTTCCCTCGCGCTTTCAAATTCCCTAAATCGTGTTTCTCTCTGACCCGCTCTCCGATACACTCCCGTCCACCCTCCCTGATTCTCTTTTTTCTTGTGTTTGCATCTCTTTGCCCGCTCTCCTACTACGCCGTTTCCCTCCCTCGCAGCCACTCGCCGCAGTCCCTCTGCGTGCCTCTCTCTCTCTATCTCGGCAGTCTTGCTACCCCTTCTTTCCTTCTGACGTCACTAGACGCGCACTCGCATCCTCGAAGGAGATCCGCTTGGCAAAATGGGGCTCTTGACGACTGGCGGCGCCCCGATACAGTGGGGCACCGATGCAAATAGAAAGGCCATTCCGCACGTCAGAGAGCACGGCATTCAGCAGTTCCTCAACGTTTTCAAGAACAAAAAGGACCTCCATGGTATGCCGTTTCTCTGGGGAGAGGAGCTGGAGCACCAGCTAATCCAGATCCACGATAACACGGTTACCCTCAGCACGGAAAGTGCGATGGTAATGAACAAGCTGAGGGCGCGTCCTGACAACTGCGCCGTGTGGAACCCCGAATATGGAAGCTTCATGATCGAAAGCACGCCAGACCACCCGTACAGTCTGTCGGTGGAGAGCCTCGACTCGGTGCAGGACAACATCGAGCGGCGGTACGACATGCTCAACAAGGAGGCACCACCCGGCGTGGTCGGCACCACCTTTGTGACTTTCCCACTCATGGGCCAGGGCAACTTTGTCCACTGCAGTGATAAGAGTTCTCCGTACTCGCAGTCGCTTTTTGTTCCTGATGCGTGCATCAACCAAACGCATCCGCGCTTCGCGAACCTGACGGCAAACATTCGCCTGCGCCGCGGTCAAAAGGTTTGCGTCCTGGTGCCTCTGTACATGGACTCCCGTACAATGCAGGACACGGTGGACCCCCAACTAAACATTGACCTGACTCCACACAACAAGGACATTTTTTACTCCATGAGAGAAAACGGCAGGAACATGACCGACGAACTCTACGCGGAGACGGACGCGTCTGCCGCTCTGCTAGTGCCCAGCAGCTCTCTCGATCCACGCGAGGACTACCCTGTCACCGAGACGCTGAAGCAGCTCTTCACCCCTGCTACGCTCTACTACTACGCACAGTACTTCACGGGACAGCGCCGCGAGCATATGCAGGAACGCTACAACGCGTGTAACTGCCCCGTAACCTTGGTCAGCCACCCGTGCATCTACATGGACTGCATGGCCTTTGGCATGGGTAACAGCGCTCTGCAAGTGACGATGCAGCTGGACAACATTCACGAGGCGCGCCACGTGTACGACCAGCTCGCCATCTTGTGCCCGGCATTTCTGGCTCTCAGCTCAGCCACGCCGTTCCAAAAGGGTCTTCTTTGCGACACCGATGTGCGCTGGCTGACTATCGCCGGCGCTGTCGACGACCGCCGCGTGGAGGAGGTGCCGCGTATTCTCAAGTCTCGCTACGACTCCATCTCCGTCTTCATCAGCGACAGAACCGAAAACCTCGAGGAATTCAACGATTCACAAATAGCGATAAACCGCTCGTACTGTGAACTTCTGAAGGACTCCGGTGTGGACGTGCGGTTGGCGAACCACATTGCACATTTGTTCATTCGAGATCCGCTTGTGATGTACGACAAGATGATCGACATCGATGACACGACGCACACGGAGCACTTTGACAACATCCAGTCCACTAACTGGCAGACAGTGCGCTTCAAGCCTCCGCCGATAGGAAACGACATTGGCTGGCGCGTTGAGTTCCGCGTGATGGATATTCAGCCAACACCGTTCGAAAACGCCGCCTTCGCCGTCTTCATTCCGCTTCTCACCAAGGCCATCATCACCTACAAGCCCTGCTTTTACACCAAGATCTCCATCGTCGACGAGAATATGGGCCGCGCACATCGCATCAACCCGTGTGGAGAACAATACATTATGCGCAAGGACATTTTCGCCGACAAGTGCACCGCCAGCGACGAGGAGACGGCGAGGATGAGCATTGACGAGATCTTCAACGGCAAGGAGGGCGGCTTCTATGGACTCATCCCCCTCGTGTGCCGCTATCTAGACGACGAGGGGAAGCGAAGTCCCCTCGTAAACTCCTACCTGAAGTTCCTGTCAATGCGCGCCTCTGGCCGCATTCCCACACCTGCGCAGTACATGCGCAAGTTTGTCACGACACACCCCGACTACAAGCACGACTCACGCCTCACCGACAGCATCGCGCGTGACCTTGTGCAGCGCATGCACGGCCTGGCTGCGAATCAGATCCACGACGATGACTACCTTCCCATGAGCTTTTTCACGGCCGATACAGTAGAGAGCACCAAGTGAGTCTTTCTTCTCATTCCCACGGACTGCAGAACGAAAGCGCCCAAGGCGTATGCTGCGTTCAGTCCCGCGCATGTGCGCTAAAGTCGACCCCGCTTTAAGCTATTTACTGAAGAACCACCCCTCCCCCAAAACAAAAAATAAAGAAAGCATTCCAGACAAAAAAAACAACAATATGGACCGTGTCTCACTTTTGCCTTCCTTCTTTTTTCCTGTGCTTTGCCTGCTGGCTTACGTCCGCGGCGGAGCAGCAACGTTGGCGCATACAGCAGCAGTTCTCTCCCTCGTTTCTTCTGCTGATTGTGCGCTTACTCTGTGCTGCTGCGCCATGCCTATTCTGATTTGTCTCGTATCCAGCGCTGTATTCCCTCTCTTTTCCCTATTGCCCTCATGNNNNNNNNNNNNNNNNNNNNNNNNNNNNNNNNNNNNNNNNNNNNNNNNNNNNNNNNNNNNNNNNNNNNNNNNNNNNNNTGCCCTCTGGTGCGGCTGCGGGCTCTCTGGCG

>BPK178/0 clone 3|LinJ18_V3.1660|500 bp UPS + CDS + 500 bp DWS|Gamma-glutamylcysteine synthase

GCTTCGGCACTTCCCAGCTGCTTTCGTCCCTCGGCTGCCCAAGCCGCTGTGCGCCATAACACACCCGTATACATTGTCATACGCACTCCTTCTTCACCTTTACGCGGCCGCACCTTCTCGTCTAACCAACCACGGCAACTACTTTGCTTCTCTATCTTCGGCCCTTATTATTATTATCATCCGCGCAGCGCCTTCACCGCGTCTGTTTTTCGTCACCTCTTCTCCCTGCGTTTTCCCTCGCGCTTTCAAATTCCCTAAATCGTGTTTCTCTCTGACCCGCTCTCCGATACACTCCCGTCCACCCTCCCTGATTCTCTTTTTTCTTGTGTTTGCATCTCTTTGCCCGCTCTCCTACTACGCCGTTTCCCTCCCTCGCAGCCACTCGCCGCAGTCCCTCTGCGTGCCTCTCTCTCTCTATCTCGGCAGTCTTGCTACCCCTTCTTTCCTTCTGACGTCACTAGACGCGCACTCGCATCCTCGAAGGAGATCCGCTTGGCAAAATGGGGCTCTTGACGACTGGCGGCGCCCCGATACAGTGGGGCACCGATGCAAATAGAAAGGCCATTCCGCACGTCAGAGAGCACGGCATTCAGCAGTTCCTCAACGTTTTCAAGAACAAAAAGGACCTCCATGGTATGCCGTTTCTCTGGGGAGAGGAGCTGGAGCACCAGCTAATCCAGATCCACGATAACACGGTTACCCTCAGCACGGAAAGTGCGATGGTAATGAACAAGCTGAGGGCGCGTCCTGACAACTGCGCCGTGTGGAACCCCGAATATGGAAGCTTCATGATCGAAAGCACGCCAGACCACCCGTACAGTCTGTCGGTGGAGAGCCTCGACTCGGTGCAGGACAACATCGAGCGGCGGTACGACATGCTCAACAAGGAGGCACCACCCGGCGTGGTCGGCACCACCTTTGTGACTTTCCCACTCATGGGCCAGGGCAACTTTGTCCACTGCAGTGATAAGAGTTCTCCGTACTCGCAGTCGCTTTTTGTTCCTGATGCGTGCATCAACCAAACGCATCCGCGCTTCGCGAACCTGACGGCAAACATTCGCCTGCGCCGCGGTCAAAAGGTTTGCGTCCTGGTGCCTCTGTACATGGACTCCCGTACAATGCAGGACACGGTGGACCCCCAACTAAACATTGACCTGACTCCACACAACAAGGACATTTTTTACTCCATGAGAGAAAACGGCAGGAACATGACCGACGAACTCTACGCGGAGACGGACGCGTCTGCCGCTCTGCTAGTGCCCAGCAGCTCTCTCGATCCACGCGAGGACTACCCTGTCACCGAGACGCTGAAGCAGCTCTTCACCCCTGCTACGCTCTACTACTACGCACAGTACTTCACGGGACAGCGCCGCGAGCATATGCAGGAACGCTACAACGCGTGTAACTGCCCCGTAACCTTGGTCAGCCACCCGTGCATCTACATGGACTGCATGGCCTTTGGCATGGGTAACAGCGCTCTGCAAGTGACGATGCAGCTGGACAACATTCACGAGGCGCGCCACGTGTACGACCAGCTCGCCATCTTGTGCCCGGCATTTCTGGCTCTCAGCTCAGCCACGCCGTTCCAAAAGGGTCTTCTTTGCGACACCGATGTGCGCTGGCTGACTATCGCCGGCGCTGTCGACGACCGCCGCGTGGAGGAGGTGCCGCGTATTCTCAAGTCTCGCTACGACTCCATCTCCGTCTTCATCAGCGACAGAACCGAAAACCTCGAGGAATTCAACGATTCACAAATAGCGATAAACCGCTCGTACTGTGAACTTCTGAAGGACTCCGGTGTGGACGTGCGGTTGGCGAACCACATTGCACATTTGTTCATTCGAGATCCGCTTGTGATGTACGACAAGATGATCGACATCGATGACACGACGCACACGGAGCACTTTGACAACATCCAGTCCACTAACTGGCAGACAGTGCGCTTCAAGCCTCCGCCGATAGGAAACGACATTGGCTGGCGCGTTGAGTTCCGCGTGATGGATATTCAGCCAACACCGTTCGAAAACGCCGCCTTCGCCGTCTTCATTCCGCTTCTCACCAAGGCCATCATCACCTACAAGCCCTGCTTTTACACCAAGATCTCCATCGTCGACGAGAATATGGGCCGCGCACATCGCATCAACCCGTGTGGAGAACAATACATTATGCGCAAGGACATTTTCGCCGACAAGTGCACCGCCAGCGACGAGGAGACGGCGAGGATGAGCATTGACGAGATCTTCAACGGCAAGGAGGGCGGCTTCTATGGACTCATCCCCCTCGTGTGCCGCTATCTAGACGACGAGGGGAAGCGAAGTCCCCTCGTAAACTCCTACCTGAAGTTCCTGTCAATGCGCGCCTCTGGCCGCATTCCCACACCTGCGCAGTACATGCGCAAGTTTGTCACGACACACCCCGACTACAAGCACGACTCACGCCTCACCGACAGCATCGCGCGTGACCTTGTGCAGCGCATGCACGGCCTGGCTGCGAATCAGATCCACGACGATGACTACCTTCCCATGAGCTTTTTCACGGCCGATACAGTAGAGAGCACCAAGTGAGTCTTTCTTCTCATTCCCACGGACTGCAGAACGAAAGCGCCCAAGGCGTATGCTGCGTTCAGTCCCGCGCATGTGCGCTAAAGTCGACCCCGCTTTAAGCTATTTACTGAAGAACCACCCCTCCCCCAAAACAAAAAATAAAGAAAGCATTCCAGACAAAAAAAACAACAATATGGACCGTGTCTCACTTTTGCCTTCCTTCTTTTTTCCTGTGCTTTGCCTGCTGGCTTACGTCCGCGGCGGAGCAGCAACGTTGGCGCATACAGCAGCAGTTCTCTCCCTCGTTTCTTCTGCTGATTGTGCGCTTACTCTGTGCTGCTGCGCCATGCCTATTCTGATTTGTCTCGTATCCAGCGCTGTATTCCCTCTCTTTTCCCTATTGCCCTCATGNNNNNNNNNNNNNNNNNNNNNNNNNNNNNNNNNNNNNNNNNNNNNNNNNNNNNNNNNNNNNNNNNNNNNNNNNNNNNNTGCCCTCTGGTGCGGCTGCGGGCTCTCTGGCG

>BPK190/0 clone 3|LinJ18_V3.1660|500 bp UPS + CDS + 500 bp DWS|Gamma-glutamylcysteine synthase

GCTTCGGCACTTCCCAGCTGCTTTCGTCCCTCGGCTGCCCAAGCCGCTGTGCGCCATAACACACCCGTATACATTGTCATACGCACTCCTTCTTCACCTTTACGCGGCCGCACCTTCTCGTCTAACCAACCACGGCAACTACTTTGCTTCTCTATCTTCGGCCCTTATTATTATTATCATCCGCGCAGCGCCTTCACCGCGTCTGTTTTTCGTCACCTCTTCTCCCTGCGTTTTCCCTCGCGCTTTCAAATTCCCTAAATCGTGTTTCTCTCTGACCCGCTCTCCGATACACTCCCGTCCACCCTCCCTGATTCTCTTTTTTCTTGTGTTTGCATCTCTTTGCCCGCTCTCCTACTACGCCGTTTCCCTCCCTCGCAGCCACTCGCCGCAGTCCCTCTGCGTGCCTCTCTCTCTCTATCTCGGCAGTCTTGCTACCCCTTCTTTCCTTCTGACGTCACTAGACGCGCACTCGCATCCTCGAAGGAGATCCGCTTGGCAAAATGGGGCTCTTGACGACTGGCGGCGCCCCGATACAGTGGGGCACCGATGCAAATAGAAAGGCCATTCCGCACGTCAGAGAGCACGGCATTCAGCAGTTCCTCAACGTTTTCAAGAACAAAAAGGACCTCCATGGTATGCCGTTTCTCTGGGGAGAGGAGCTGGAGCACCAGCTAATCCAGATCCACGATAACACGGTTACCCTCAGCACGGAAAGTGCGATGGTAATGAACAAGCTGAGGGCGCGTCCTGACAACTGCGCCGTGTGGAACCCCGAATATGGAAGCTTCATGATCGAAAGCACGCCAGACCACCCGTACAGTCTGTCGGTGGAGAGCCTCGACTCGGTGCAGGACAACATCGAGCGGCGGTACGACATGCTCAACAAGGAGGCACCACCCGGCGTGGTCGGCACCACCTTTGTGACTTTCCCACTCATGGGCCAGGGCAACTTTGTCCACTGCAGTGATAAGAGTTCTCCGTACTCGCAGTCGCTTTTTGTTCCTGATGCGTGCATCAACCAAACGCATCCGCGCTTCGCGAACCTGACGGCAAACATTCGCCTGCGCCGCGGTCAAAAGGTTTGCGTCCTGGTGCCTCTGTACATGGACTCCCGTACAATGCAGGACACGGTGGACCCCCAACTAAACATTGACCTGACTCCACACAACAAGGACATTTTTTACTCCATGAGAGAAAACGGCAGGAACATGACCGACGAACTCTACGCGGAGACGGACGCGTCTGCCGCTCTGCTAGTGCCCAGCAGCTCTCTCGATCCACGCGAGGACTACCCTGTCACCGAGACGCTGAAGCAGCTCTTCACCCCTGCTACGCTCTACTACTACGCACAGTACTTCACGGGACAGCGCCGCGAGCATATGCAGGAACGCTACAACGCGTGTAACTGCCCCGTAACCTTGGTCAGCCACCCGTGCATCTACATGGACTGCATGGCCTTTGGCATGGGTAACAGCGCTCTGCAAGTGACGATGCAGCTGGACAACATTCACGAGGCGCGCCACGTGTACGACCAGCTCGCCATCTTGTGCCCGGCATTTCTGGCTCTCAGCTCAGCCACGCCGTTCCAAAAGGGTCTTCTTTGCGACACCGATGTGCGCTGGCTGACTATCGCCGGCGCTGTCGACGACCGCCGCGTGGAGGAGGTGCCGCGTATTCTCAAGTCTCGCTACGACTCCATCTCCGTCTTCATCAGCGACAGAACCGAAAACCTCGAGGAATTCAACGATTCACAAATAGCGATAAACCGCTCGTACTGTGAACTTCTGAAGGACTCCGGTGTGGACGTGCGGTTGGCGAACCACATTGCACATTTGTTCATTCGAGATCCGCTTGTGATGTACGACAAGATGATCGACATCGATGACACGACGCACACGGAGCACTTTGACAACATCCAGTCCACTAACTGGCAGACAGTGCGCTTCAAGCCTCCGCCGATAGGAAACGACATTGGCTGGCGCGTTGAGTTCCGCGTGATGGATATTCAGCCAACACCGTTCGAAAACGCCGCCTTCGCCGTCTTCATTCCGCTTCTCACCAAGGCCATCATCACCTACAAGCCCTGCTTTTACACCAAGATCTCCATCGTCGACGAGAATATGGGCCGCGCACATCGCATCAACCCGTGTGGAGAACAATACATTATGCGCAAGGACATTTTCGCCGACAAGTGCACCGCCAGCGACGAGGAGACGGCGAGGATGAGCATTGACGAGATCTTCAACGGCAAGGAGGGCGGCTTCTATGGACTCATCCCCCTCGTGTGCCGCTATCTAGACGACGAGGGGAAGCGAAGTCCCCTCGTAAACTCCTACCTGAAGTTCCTGTCAATGCGCGCCTCTGGCCGCATTCCCACACCTGCGCAGTACATGCGCAAGTTTGTCACGACACACCCCGACTACAAGCACGACTCACGCCTCACCGACAGCATCGCGCGTGACCTTGTGCAGCGCATGCACGGCCTGGCTGCGAATCAGATCCACGACGATGACTACCTTCCCATGAGCTTTTTCACGGCCGATACAGTAGAGAGCACCAAGTGAGTCTTTCTTCTCATTCCCACGGACTGCAGAACGAAAGCGCCCAAGGCGTATGCTGCGTTCAGTCCCGCGCATGTGCGCTAAAGTCGACCCCGCTTTAAGCTATTTACTGAAGAACCACCCCTCCCCCAAAACAAAAAATAAAGAAAGCATTCCAGACAAAAAAAACAACAATATGGACCGTGTCTCACTTTTGCCTTCCTTCTTTTTTCCTGTGCTTTGCCTGCTGGCTTACGTCCGCGGCGGAGCAGCAACGTTGGCGCATACAGCAGCAGTTCTCTCCCTCGTTTCTTCTGCTGATTGTGCGCTTACTCTGTGCTGCTGCGCCATGCCTATTCTGATTTGTCTCGTATCCAGCGCTGTATTCCCTCTCTTTTCCCTATTGCCCTCATGNNNNNNNNNNNNNNNNNNNNNNNNNNNNNNNNNNNNNNNNNNNNNNNNNNNNNNNNNNNNNNNNNNNNNNNNNNNNNNTGCCCTCTGGTGCGGCTGCGGGCTCTCTGGCG

>BPK206/0 clone 10|LinJ18_V3.1660|500 bp UPS + CDS + 500 bp DWS|Gamma-glutamylcysteine synthase

GCTTCGGCACTTCCCAGCTGCTTTCGTCCCTCGGCTGCCCAAGCCGCTGTGCGCCATAACACACCCGTATACATTGTCATACGCACTCCTTCTTCACCTTTACGCGGCCGCACCTTCTCGTCTAACCAACCACGGCAACTACTTTGCTTCTCTATCTTCGGCCCTTATTATTATTATCATCCGCGCAGCGCCTTCACCGCGTCTGTTTTTCGTCACCTCTTCTCCCTGCGTTTTCCCTCGCGCTTTCAAATTCCCTAAATCGTGTTTCTCTCTGACCCGCTCTCCGATACACTCCCGTCCACCCTCCCTGATTCTCTTTTTTCTTGTGTTTGCATCTCTTTGCCCGCTCTCCTACTACGCCGTTTCCCTCCCTCGCAGCCACTCGCCGCAGTCCCTCTGCGTGCCTCTCTCTCTCTATCTCGGCAGTCTTGCTACCCCTTCTTTCCTTCTGACGTCACTAGACGCGCACTCGCATCCTCGAAGGAGATCCGCTTGGCAAAATGGGGCTCTTGACGACTGGCGGCGCCCCGATACAGTGGGGCACCGATGCAAATAGAAAGGCCATTCCGCACGTCAGAGAGCACGGCATTCAGCAGTTCCTCAACGTTTTCAAGAACAAAAAGGACCTCCATGGTATGCCGTTTCTCTGGGGAGAGGAGCTGGAGCACCAGCTAATCCAGATCCACGATAACACGGTTACCCTCAGCACGGAAAGTGCGATGGTAATGAACAAGCTGAGGGCGCGTCCTGACAACTGCGCCGTGTGGAACCCCGAATATGGAAGCTTCATGATCGAAAGCACGCCAGACCACCCGTACAGTCTGTCGGTGGAGAGCCTCGACTCGGTGCAGGACAACATCGAGCGGCGGTACGACATGCTCAACAAGGAGGCACCACCCGGCGTGGTCGGCACCACCTTTGTGACTTTCCCACTCATGGGCCAGGGCAACTTTGTCCACTGCAGTGATAAGAGTTCTCCGTACTCGCAGTCGCTTTTTGTTCCTGATGCGTGCATCAACCAAACGCATCCGCGCTTCGCGAACCTGACGGCAAACATTCGCCTGCGCCGCGGTCAAAAGGTTTGCGTCCTGGTGCCTCTGTACATGGACTCCCGTACAATGCAGGACACGGTGGACCCCCAACTAAACATTGACCTGACTCCACACAACAAGGACATTTTTTACTCCATGAGAGAAAACGGCAGGAACATGACCGACGAACTCTACGCGGAGACGGACGCGTCTGCCGCTCTGCTAGTGCCCAGCAGCTCTCTCGATCCACGCGAGGACTACCCTGTCACCGAGACGCTGAAGCAGCTCTTCACCCCTGCTACGCTCTACTACTACGCACAGTACTTCACGGGACAGCGCCGCGAGCATATGCAGGAACGCTACAACGCGTGTAACTGCCCCGTAACCTTGGTCAGCCACCCGTGCATCTACATGGACTGCATGGCCTTTGGCATGGGTAACAGCGCTCTGCAAGTGACGATGCAGCTGGACAACATTCACGAGGCGCGCCACGTGTACGACCAGCTCGCCATCTTGTGCCCGGCATTTCTGGCTCTCAGCTCAGCCACGCCGTTCCAAAAGGGTCTTCTTTGCGACACCGATGTGCGCTGGCTGACTATCGCCGGCGCTGTCGACGACCGCCGCGTGGAGGAGGTGCCGCGTATTCTCAAGTCTCGCTACGACTCCATCTCCGTCTTCATCAGCGACAGAACCGAAAACCTCGAGGAATTCAACGATTCACAAATAGCGATAAACCGCTCGTACTGTGAACTTCTGAAGGACTCCGGTGTGGACGTGCGGTTGGCGAACCACATTGCACATTTGTTCATTCGAGATCCGCTTGTGATGTACGACAAGATGATCGACATCGATGACACGACGCACACGGAGCACTTTGACAACATCCAGTCCACTAACTGGCAGACAGTGCGCTTCAAGCCTCCGCCGATAGGAAACGACATTGGCTGGCGCGTTGAGTTCCGCGTGATGGATATTCAGCCAACACCGTTCGAAAACGCCGCCTTCGCCGTCTTCATTCCGCTTCTCACCAAGGCCATCATCACCTACAAGCCCTGCTTTTACACCAAGATCTCCATCGTCGACGAGAATATGGGCCGCGCACATCGCATCAACCCGTGTGGAGAACAATACATTATGCGCAAGGACATTTTCGCCGACAAGTGCACCGCCAGCGACGAGGAGACGGCGAGGATGAGCATTGACGAGATCTTCAACGGCAAGGAGGGCGGCTTCTATGGACTCATCCCCCTCGTGTGCCGCTATCTAGACGACGAGGGGAAGCGAAGTCCCCTCGTAAACTCCTACCTGAAGTTCCTGTCAATGCGCGCCTCTGGCCGCATTCCCACACCTGCGCAGTACATGCGCAAGTTTGTCACGACACACCCCGACTACAAGCACGACTCACGCCTCACCGACAGCATCGCGCGTGACCTTGTGCAGCGCATGCACGGCCTGGCTGCGAATCAGATCCACGACGATGACTACCTTCCCATGAGCTTTTTCACGGCCGATACAGTAGAGAGCACCAAGTGAGTCTTTCTTCTCATTCCCACGGACTGCAGAACGAAAGCGCCCAAGGCGTATGCTGCGTTCAGTCCCGCGCATGTGCGCTAAAGTCGACCCCGCTTTAAGCTATTTACTGAAGAACCACCCCTCCCCCAAAACAAAAAATAAAGAAAGCATTCCAGACAAAAAAAACAACAATATGGACCGTGTCTCACTTTTGCCTTCCTTCTTTTTTCCTGTGCTTTGCCTGCTGGCTTACGTCCGCGGCGGAGCAGCAACGTTGGCGCATACAGCAGCAGTTCTCTCCCTCGTTTCTTCTGCTGATTGTGCGCTTACTCTGTGCTGCTGCGCCATGCCTATTCTGATTTGTCTCGTATCCAGCGCTGTATTCCCTCTCTTTTCCCTATTGCCCTCATGNNNNNNNNNNNNNNNNNNNNNNNNNNNNNNNNNNNNNNNNNNNNNNNNNNNNNNNNNNNNNNNNNNNNNNNNNNNNNNTGCCCTCTGGTGCGGCTGCGGGCTCTCTGGCG

>BPK275/0 clone 18|LinJ18_V3.1660|500 bp UPS + CDS + 500 bp DWS|Gamma-glutamylcysteine synthase

GCTTCGGCACTTCCCAGCTGCTTTCGTCCCTCGGCTGCCCAAGCCGCTGTGCGCCATAACACACCCGTATACATTGTCATACGCACTCCTTCTTCACCTTTACGCGGCCGCACCTTCTCGTCTAACCAACCACGGCAACTACTTTGCTTCTCTATCTTCGGCCCTTATTATTATTATCATCCGCGCAGCGCCTTCACCGCGTCTGTTTTTCGTCACCTCTTCTCCCTGCGTTTTCCCTCGCGCTTTCAAATTCCCTAAATCGTGTTTCTCTCTGACCCGCTCTCCGATACACTCCCGTCCACCCTCCCTGATTCTCTTTTTTCTTGTGTTTGCATCTCTTTGCCCGCTCTCCTACTACGCCGTTTCCCTCCCTCGCAGCCACTCGCCGCAGTCCCTCTGCGTGCCTCTCTCTCTCTATCTCGGCAGTCTTGCTACCCCTTCTTTCCTTCTGACGTCACTAGACGCGCACTCGCATCCTCGAAGGAGATCCGCTTGGCAAAATGGGGCTCTTGACGACTGGCGGCGCCCCGATACAGTGGGGCACCGATGCAAATAGAAAGGCCATTCCGCACGTCAGAGAGCACGGCATTCAGCAGTTCCTCAACGTTTTCAAGAACAAAAAGGACCTCCATGGTATGCCGTTTCTCTGGGGAGAGGAGCTGGAGCACCAGCTAATCCAGATCCACGATAACACGGTTACCCTCAGCACGGAAAGTGCGATGGTAATGAACAAGCTGAGGGCGCGTCCTGACAACTGCGCCGTGTGGAACCCCGAATATGGAAGCTTCATGATCGAAAGCACGCCAGACCACCCGTACAGTCTGTCGGTGGAGAGCCTCGACTCGGTGCAGGACAACATCGAGCGGCGGTACGACATGCTCAACAAGGAGGCACCACCCGGCGTGGTCGGCACCACCTTTGTGACTTTCCCACTCATGGGCCAGGGCAACTTTGTCCACTGCAGTGATAAGAGTTCTCCGTACTCGCAGTCGCTTTTTGTTCCTGATGCGTGCATCAACCAAACGCATCCGCGCTTCGCGAACCTGACGGCAAACATTCGCCTGCGCCGCGGTCAAAAGGTTTGCGTCCTGGTGCCTCTGTACATGGACTCCCGTACAATGCAGGACACGGTGGACCCCCAACTAAACATTGACCTGACTCCACACAACAAGGACATTTTTTACTCCATGAGAGAAAACGGCAGGAACATGACCGACGAACTCTACGCGGAGACGGACGCGTCTGCCGCTCTGCTAGTGCCCAGCAGCTCTCTCGATCCACGCGAGGACTACCCTGTCACCGAGACGCTGAAGCAGCTCTTCACCCCTGCTACGCTCTACTACTACGCACAGTACTTCACGGGACAGCGCCGCGAGCATATGCAGGAACGCTACAACGCGTGTAACTGCCCCGTAACCTTGGTCAGCCACCCGTGCATCTACATGGACTGCATGGCCTTTGGCATGGGTAACAGCGCTCTGCAAGTGACGATGCAGCTGGACAACATTCACGAGGCGCGCCACGTGTACGACCAGCTCGCCATCTTGTGCCCGGCATTTCTGGCTCTCAGCTCAGCCACGCCGTTCCAAAAGGGTCTTCTTTGCGACACCGATGTGCGCTGGCTGACTATCGCCGGCGCTGTCGACGACCGCCGCGTGGAGGAGGTGCCGCGTATTCTCAAGTCTCGCTACGACTCCATCTCCGTCTTCATCAGCGACAGAACCGAAAACCTCGAGGAATTCAACGATTCACAAATAGCGATAAACCGCTCGTACTGTGAACTTCTGAAGGACTCCGGTGTGGACGTGCGGTTGGCGAACCACATTGCACATTTGTTCATTCGAGATCCGCTTGTGATGTACGACAAGATGATCGACATCGATGACACGACGCACACGGAGCACTTTGACAACATCCAGTCCACTAACTGGCAGACAGTGCGCTTCAAGCCTCCGCCGATAGGAAACGACATTGGCTGGCGCGTTGAGTTCCGCGTGATGGATATTCAGCCAACACCGTTCGAAAACGCCGCCTTCGCCGTCTTCATTCCGCTTCTCACCAAGGCCATCATCACCTACAAGCCCTGCTTTTACACCAAGATCTCCATCGTCGACGAGAATATGGGCCGCGCACATCGCATCAACCCGTGTGGAGAACAATACATTATGCGCAAGGACATTTTCGCCGACAAGTGCACCGCCAGCGACGAGGAGACGGCGAGGATGAGCATTGACGAGATCTTCAACGGCAAGGAGGGCGGCTTCTATGGACTCATCCCCCTCGTGTGCCGCTATCTAGACGACGAGGGGAAGCGAAGTCCCCTCGTAAACTCCTACCTGAAGTTCCTGTCAATGCGCGCCTCTGGCCGCATTCCCACACCTGCGCAGTACATGCGCAAGTTTGTCACGACACACCCCGACTACAAGCACGACTCACGCCTCACCGACAGCATCGCGCGTGACCTTGTGCAGCGCATGCACGGCCTGGCTGCGAATCAGATCCACGACGATGACTACCTTCCCATGAGCTTTTTCACGGCCGATACAGTAGAGAGCACCAAGTGAGTCTTTCTTCTCATTCCCACGGACTGCAGAACGAAAGCGCCCAAGGCGTATGCTGCGTTCAGTCCCGCGCATGTGCGCTAAAGTCGACCCCGCTTTAAGCTATTTACTGAAGAACCACCCCTCCCCCAAAACAAAAAATAAAGAAAGCATTCCAGACAAAAAAAACAACAATATGGACCGTGTCTCACTTTTGCCTTCCTTCTTTTTTCCTGTGCTTTGCCTGCTGGCTTACGTCCGCGGCGGAGCAGCAACGTTGGCGCATACAGCAGCAGTTCTCTCCCTCGTTTCTTCTGCTGATTGTGCGCTTACTCTGTGCTGCTGCGCCATGCCTATTCTGATTTGTCTCGTATCCAGCGCTGTATTCCCTCTCTTTTCCCTATTGCCCTCATGNNNNNNNNNNNNNNNNNNNNNNNNNNNNNNNNNNNNNNNNNNNNNNNNNNNNNNNNNNNNNNNNNNNNNNNNNNNNNNTGCCCTCTGGTGCGGCTGCGGGCTCTCTGGCG

>BPK282/0 clone 4|LinJ18_V3.1660|500 bp UPS + CDS + 500 bp DWS|Gamma-glutamylcysteine synthase

GCTTCGGCACTTCCCAGCTGCTTTCGTCCCTCGGCTGCCCAAGCCGCTGTGCGCCATAACACACCCGTATACATTGTCATACGCACTCCTTCTTCACCTTTACGCGGCCGCACCTTCTCGTCTAACCAACCACGGCAACTACTTTGCTTCTCTATCTTCGGCCCTTATTATTATTATCATCCGCGCAGCGCCTTCACCGCGTCTGTTTTTCGTCACCTCTTCTCCCTGCGTTTTCCCTCGCGCTTTCAAATTCCCTAAATCGTGTTTCTCTCTGACCCGCTCTCCGATACACTCCCGTCCACCCTCCCTGATTCTCTTTTTTCTTGTGTTTGCATCTCTTTGCCCGCTCTCCTACTACGCCGTTTCCCTCCCTCGCAGCCACTCGCCGCAGTCCCTCTGCGTGCCTCTCTCTCTCTATCTCGGCAGTCTTGCTACCCCTTCTTTCCTTCTGACGTCACTAGACGCGCACTCGCATCCTCGAAGGAGATCCGCTTGGCAAAATGGGGCTCTTGACGACTGGCGGCGCCCCGATACAGTGGGGCACCGATGCAAATAGAAAGGCCATTCCGCACGTCAGAGAGCACGGCATTCAGCAGTTCCTCAACGTTTTCAAGAACAAAAAGGACCTCCATGGTATGCCGTTTCTCTGGGGAGAGGAGCTGGAGCACCAGCTAATCCAGATCCACGATAACACGGTTACCCTCAGCACGGAAAGTGCGATGGTAATGAACAAGCTGAGGGCGCGTCCTGACAACTGCGCCGTGTGGAACCCCGAATATGGAAGCTTCATGATCGAAAGCACGCCAGACCACCCGTACAGTCTGTCGGTGGAGAGCCTCGACTCGGTGCAGGACAACATCGAGCGGCGGTACGACATGCTCAACAAGGAGGCACCACCCGGCGTGGTCGGCACCACCTTTGTGACTTTCCCACTCATGGGCCAGGGCAACTTTGTCCACTGCAGTGATAAGAGTTCTCCGTACTCGCAGTCGCTTTTTGTTCCTGATGCGTGCATCAACCAAACGCATCCGCGCTTCGCGAACCTGACGGCAAACATTCGCCTGCGCCGCGGTCAAAAGGTTTGCGTCCTGGTGCCTCTGTACATGGACTCCCGTACAATGCAGGACACGGTGGACCCCCAACTAAACATTGACCTGACTCCACACAACAAGGACATTTTTTACTCCATGAGAGAAAACGGCAGGAACATGACCGACGAACTCTACGCGGAGACGGACGCGTCTGCCGCTCTGCTAGTGCCCAGCAGCTCTCTCGATCCACGCGAGGACTACCCTGTCACCGAGACGCTGAAGCAGCTCTTCACCCCTGCTACGCTCTACTACTACGCACAGTACTTCACGGGACAGCGCCGCGAGCATATGCAGGAACGCTACAACGCGTGTAACTGCCCCGTAACCTTGGTCAGCCACCCGTGCATCTACATGGACTGCATGGCCTTTGGCATGGGTAACAGCGCTCTGCAAGTGACGATGCAGCTGGACAACATTCACGAGGCGCGCCACGTGTACGACCAGCTCGCCATCTTGTGCCCGGCATTTCTGGCTCTCAGCTCAGCCACGCCGTTCCAAAAGGGTCTTCTTTGCGACACCGATGTGCGCTGGCTGACTATCGCCGGCGCTGTCGACGACCGCCGCGTGGAGGAGGTGCCGCGTATTCTCAAGTCTCGCTACGACTCCATCTCCGTCTTCATCAGCGACAGAACCGAAAACCTCGAGGAATTCAACGATTCACAAATAGCGATAAACCGCTCGTACTGTGAACTTCTGAAGGACTCCGGTGTGGACGTGCGGTTGGCGAACCACATTGCACATTTGTTCATTCGAGATCCGCTTGTGATGTACGACAAGATGATCGACATCGATGACACGACGCACACGGAGCACTTTGACAACATCCAGTCCACTAACTGGCAGACAGTGCGCTTCAAGCCTCCGCCGATAGGAAACGACATTGGCTGGCGCGTTGAGTTCCGCGTGATGGATATTCAGCCAACACCGTTCGAAAACGCCGCCTTCGCCGTCTTCATTCCGCTTCTCACCAAGGCCATCATCACCTACAAGCCCTGCTTTTACACCAAGATCTCCATCGTCGACGAGAATATGGGCCGCGCACATCGCATCAACCCGTGTGGAGAACAATACATTATGCGCAAGGACATTTTCGCCGACAAGTGCACCGCCAGCGACGAGGAGACGGCGAGGATGAGCATTGACGAGATCTTCAACGGCAAGGAGGGCGGCTTCTATGGACTCATCCCCCTCGTGTGCCGCTATCTAGACGACGAGGGGAAGCGAAGTCCCCTCGTAAACTCCTACCTGAAGTTCCTGTCAATGCGCGCCTCTGGCCGCATTCCCACACCTGCGCAGTACATGCGCAAGTTTGTCACGACACACCCCGACTACAAGCACGACTCACGCCTCACCGACAGCATCGCGCGTGACCTTGTGCAGCGCATGCACGGCCTGGCTGCGAATCAGATCCACGACGATGACTACCTTCCCATGAGCTTTTTCACGGCCGATACAGTAGAGAGCACCAAGTGAGTCTTTCTTCTCATTCCCACGGACTGCAGAACGAAAGCGCCCAAGGCGTATGCTGCGTTCAGTCCCGCGCATGTGCGCTAAAGTCGACCCCGCTTTAAGCTATTTACTGAAGAACCACCCCTCCCCCAAAACAAAAAATAAAGAAAGCATTCCAGACAAAAAAAACAACAATATGGACCGTGTCTCACTTTTGCCTTCCTTCTTTTTTCCTGTGCTTTGCCTGCTGGCTTACGTCCGCGGCGGAGCAGCAACGTTGGCGCATACAGCAGCAGTTCTCTCCCTCGTTTCTTCTGCTGATTGTGCGCTTACTCTGTGCTGCTGCGCCATGCCTATTCTGATTTGTCTCGTATCCAGCGCTGTATTCCCTCTCTTTTCCCTATTGCCCTCATGNNNNNNNNNNNNNNNNNNNNNNNNNNNNNNNNNNNNNNNNNNNNNNNNNNNNNNNNNNNNNNNNNNNNNNNNNNNNNNTGCCCTCTGGTGCGGCTGCGGGCTCTCTGGCG

>BPK294/0 clone 1|LinJ18_V3.1660|500 bp UPS + CDS + 500 bp DWS|Gamma-glutamylcysteine synthase

GCTTCGGCACTTCCCAGCTGCTTTCGTCCCTCGGCTGCCCAAGCCGCTGTGCGCCATAACACACCCGTATACATTGTCATACGCACTCCTTCTTCACCTTTACGCGGCCGCACCTTCTCGTCTAACCAACCACGGCAACTACTTTGCTTCTCTATCTTCGGCCCTTATTATTATTATCATCCGCGCAGCGCCTTCACCGCGTCTGTTTTTCGTCACCTCTTCTCCCTGCGTTTTCCCTCGCGCTTTCAAATTCCCTAAATCGTGTTTCTCTCTGACCCGCTCTCCGATACACTCCCGTCCACCCTCCCTGATTCTCTTTTTTCTTGTGTTTGCATCTCTTTGCCCGCTCTCCTACTACGCCGTTTCCCTCCCTCGCAGCCACTCGCCGCAGTCCCTCTGCGTGCCTCTCTCTCTCTATCTCGGCAGTCTTGCTACCCCTTCTTTCCTTCTGACGTCACTAGACGCGCACTCGCATCCTCGAAGGAGATCCGCTTGGCAAAATGGGGCTCTTGACGACTGGCGGCGCCCCGATACAGTGGGGCACCGATGCAAATAGAAAGGCCATTCCGCACGTCAGAGAGCACGGCATTCAGCAGTTCCTCAACGTTTTCAAGAACAAAAAGGACCTCCATGGTATGCCGTTTCTCTGGGGAGAGGAGCTGGAGCACCAGCTAATCCAGATCCACGATAACACGGTTACCCTCAGCACGGAAAGTGCGATGGTAATGAACAAGCTGAGGGCGCGTCCTGACAACTGCGCCGTGTGGAACCCCGAATATGGAAGCTTCATGATCGAAAGCACGCCAGACCACCCGTACAGTCTGTCGGTGGAGAGCCTCGACTCGGTGCAGGACAACATCGAGCGGCGGTACGACATGCTCAACAAGGAGGCACCACCCGGCGTGGTCGGCACCACCTTTGTGACTTTCCCACTCATGGGCCAGGGCAACTTTGTCCACTGCAGTGATAAGAGTTCTCCGTACTCGCAGTCGCTTTTTGTTCCTGATGCGTGCATCAACCAAACGCATCCGCGCTTCGCGAACCTGACGGCAAACATTCGCCTGCGCCGCGGTCAAAAGGTTTGCGTCCTGGTGCCTCTGTACATGGACTCCCGTACAATGCAGGACACGGTGGACCCCCAACTAAACATTGACCTGACTCCACACAACAAGGACATTTTTTACTCCATGAGAGAAAACGGCAGGAACATGACCGACGAACTCTACGCGGAGACGGACGCGTCTGCCGCTCTGCTAGTGCCCAGCAGCTCTCTCGATCCACGCGAGGACTACCCTGTCACCGAGACGCTGAAGCAGCTCTTCACCCCTGCTACGCTCTACTACTACGCACAGTACTTCACGGGACAGCGCCGCGAGCATATGCAGGAACGCTACAACGCGTGTAACTGCCCCGTAACCTTGGTCAGCCACCCGTGCATCTACATGGACTGCATGGCCTTTGGCATGGGTAACAGCGCTCTGCAAGTGACGATGCAGCTGGACAACATTCACGAGGCGCGCCACGTGTACGACCAGCTCGCCATCTTGTGCCCGGCATTTCTGGCTCTCAGCTCAGCCACGCCGTTCCAAAAGGGTCTTCTTTGCGACACCGATGTGCGCTGGCTGACTATCGCCGGCGCTGTCGACGACCGCCGCGTGGAGGAGGTGCCGCGTATTCTCAAGTCTCGCTACGACTCCATCTCCGTCTTCATCAGCGACAGAACCGAAAACCTCGAGGAATTCAACGATTCACAAATAGCGATAAACCGCTCGTACTGTGAACTTCTGAAGGACTCCGGTGTGGACGTGCGGTTGGCGAACCACATTGCACATTTGTTCATTCGAGATCCGCTTGTGATGTACGACAAGATGATCGACATCGATGACACGACGCACACGGAGCACTTTGACAACATCCAGTCCACTAACTGGCAGACAGTGCGCTTCAAGCCTCCGCCGATAGGAAACGACATTGGCTGGCGCGTTGAGTTCCGCGTGATGGATATTCAGCCAACACCGTTCGAAAACGCCGCCTTCGCCGTCTTCATTCCGCTTCTCACCAAGGCCATCATCACCTACAAGCCCTGCTTTTACACCAAGATCTCCATCGTCGACGAGAATATGGGCCGCGCACATCGCATCAACCCGTGTGGAGAACAATACATTATGCGCAAGGACATTTTCGCCGACAAGTGCACCGCCAGCGACGAGGAGACGGCGAGGATGAGCATTGACGAGATCTTCAACGGCAAGGAGGGCGGCTTCTATGGACTCATCCCCCTCGTGTGCCGCTATCTAGACGACGAGGGGAAGCGAAGTCCCCTCGTAAACTCCTACCTGAAGTTCCTGTCAATGCGCGCCTCTGGCCGCATTCCCACACCTGCGCAGTACATGCGCAAGTTTGTCACGACACACCCCGACTACAAGCACGACTCACGCCTCACCGACAGCATCGCGCGTGACCTTGTGCAGCGCATGCACGGCCTGGCTGCGAATCAGATCCACGACGATGACTACCTTCCCATGAGCTTTTTCACGGCCGATACAGTAGAGAGCACCAAGTGAGTCTTTCTTCTCATTCCCACGGACTGCAGAACGAAAGCGCCCAAGGCGTATGCTGCGTTCAGTCCCGCGCATGTGCGCTAAAGTCGACCCCGCTTTAAGCTATTTACTGAAGAACCACCCCTCCCCCAAAACAAAAAATAAAGAAAGCATTCCAGACAAAAAAAACAACAATATGGACCGTGTCTCACTTTTGCCTTCCTTCTTTTTTCCTGTGCTTTGCCTGCTGGCTTACGTCCGCGGCGGAGCAGCAACGTTGGCGCATACAGCAGCAGTTCTCTCCCTCGTTTCTTCTGCTGATTGTGCGCTTACTCTGTGCTGCTGCGCCATGCCTATTCTGATTTGTCTCGTATCCAGCGCTGTATTCCCTCTCTTTTCCCTATTGCCCTCATGNNNNNNNNNNNNNNNNNNNNNNNNNNNNNNNNNNNNNNNNNNNNNNNNNNNNNNNNNNNNNNNNNNNNNNNNNNNNNNTGCCCTCTGGTGCGGCTGCGGGCTCTCTGGCG

>BPK298/0 clone 8|LinJ18_V3.1660|500 bp UPS + CDS + 500 bp DWS|Gamma-glutamylcysteine synthase

GCTTCGGCACTTCCCAGCTGCTTTCGTCCCTCGGCTGCCCAAGCCGCTGTGCGCCATAACACACCCGTATACATTGTCATACGCACTCCTTCTTCACCTTTACGCGGCCGCACCTTCTCGTCTAACCAACCACGGCAACTACTTTGCTTCTCTATCTTCGGCCCTTATTATTATTATCATCCGCGCAGCGCCTTCACCGCGTCTGTTTTTCGTCACCTCTTCTCCCTGCGTTTTCCCTCGCGCTTTCAAATTCCCTAAATCGTGTTTCTCTCTGACCCGCTCTCCGATACACTCCCGTCCACCCTCCCTGATTCTCTTTTTTCTTGTGTTTGCATCTCTTTGCCCGCTCTCCTACTACGCCGTTTCCCTCCCTCGCAGCCACTCGCCGCAGTCCCTCTGCGTGCCTCTCTCTCTCTATCTCGGCAGTCTTGCTACCCCTTCTTTCCTTCTGACGTCACTAGACGCGCACTCGCATCCTCGAAGGAGATCCGCTTGGCAAAATGGGGCTCTTGACGACTGGCGGCGCCCCGATACAGTGGGGCACCGATGCAAATAGAAAGGCCATTCCGCACGTCAGAGAGCACGGCATTCAGCAGTTCCTCAACGTTTTCAAGAACAAAAAGGACCTCCATGGTATGCCGTTTCTCTGGGGAGAGGAGCTGGAGCACCAGCTAATCCAGATCCACGATAACACGGTTACCCTCAGCACGGAAAGTGCGATGGTAATGAACAAGCTGAGGGCGCGTCCTGACAACTGCGCCGTGTGGAACCCCGAATATGGAAGCTTCATGATCGAAAGCACGCCAGACCACCCGTACAGTCTGTCGGTGGAGAGCCTCGACTCGGTGCAGGACAACATCGAGCGGCGGTACGACATGCTCAACAAGGAGGCACCACCCGGCGTGGTCGGCACCACCTTTGTGACTTTCCCACTCATGGGCCAGGGCAACTTTGTCCACTGCAGTGATAAGAGTTCTCCGTACTCGCAGTCGCTTTTTGTTCCTGATGCGTGCATCAACCAAACGCATCCGCGCTTCGCGAACCTGACGGCAAACATTCGCCTGCGCCGCGGTCAAAAGGTTTGCGTCCTGGTGCCTCTGTACATGGACTCCCGTACAATGCAGGACACGGTGGACCCCCAACTAAACATTGACCTGACTCCACACAACAAGGACATTTTTTACTCCATGAGAGAAAACGGCAGGAACATGACCGACGAACTCTACGCGGAGACGGACGCGTCTGCCGCTCTGCTAGTGCCCAGCAGCTCTCTCGATCCACGCGAGGACTACCCTGTCACCGAGACGCTGAAGCAGCTCTTCACCCCTGCTACGCTCTACTACTACGCACAGTACTTCACGGGACAGCGCCGCGAGCATATGCAGGAACGCTACAACGCGTGTAACTGCCCCGTAACCTTGGTCAGCCACCCGTGCATCTACATGGACTGCATGGCCTTTGGCATGGGTAACAGCGCTCTGCAAGTGACGATGCAGCTGGACAACATTCACGAGGCGCGCCACGTGTACGACCAGCTCGCCATCTTGTGCCCGGCATTTCTGGCTCTCAGCTCAGCCACGCCGTTCCAAAAGGGTCTTCTTTGCGACACCGATGTGCGCTGGCTGACTATCGCCGGCGCTGTCGACGACCGCCGCGTGGAGGAGGTGCCGCGTATTCTCAAGTCTCGCTACGACTCCATCTCCGTCTTCATCAGCGACAGAACCGAAAACCTCGAGGAATTCAACGATTCACAAATAGCGATAAACCGCTCGTACTGTGAACTTCTGAAGGACTCCGGTGTGGACGTGCGGTTGGCGAACCACATTGCACATTTGTTCATTCGAGATCCGCTTGTGATGTACGACAAGATGATCGACATCGATGACACGACGCACACGGAGCACTTTGACAACATCCAGTCCACTAACTGGCAGACAGTGCGCTTCAAGCCTCCGCCGATAGGAAACGACATTGGCTGGCGCGTTGAGTTCCGCGTGATGGATATTCAGCCAACACCGTTCGAAAACGCCGCCTTCGCCGTCTTCATTCCGCTTCTCACCAAGGCCATCATCACCTACAAGCCCTGCTTTTACACCAAGATCTCCATCGTCGACGAGAATATGGGCCGCGCACATCGCATCAACCCGTGTGGAGAACAATACATTATGCGCAAGGACATTTTCGCCGACAAGTGCACCGCCAGCGACGAGGAGACGGCGAGGATGAGCATTGACGAGATCTTCAACGGCAAGGAGGGCGGCTTCTATGGACTCATCCCCCTCGTGTGCCGCTATCTAGACGACGAGGGGAAGCGAAGTCCCCTCGTAAACTCCTACCTGAAGTTCCTGTCAATGCGCGCCTCTGGCCGCATTCCCACACCTGCGCAGTACATGCGCAAGTTTGTCACGACACACCCCGACTACAAGCACGACTCACGCCTCACCGACAGCATCGCGCGTGACCTTGTGCAGCGCATGCACGGCCTGGCTGCGAATCAGATCCACGACGATGACTACCTTCCCATGAGCTTTTTCACGGCCGATACAGTAGAGAGCACCAAGTGAGTCTTTCTTCTCATTCCCACGGACTGCAGAACGAAAGCGCCCAAGGCGTATGCTGCGTTCAGTCCCGCGCATGTGCGCTAAAGTCGACCCCGCTTTAAGCTATTTACTGAAGAACCACCCCTCCCCCAAAACAAAAAATAAAGAAAGCATTCCAGACAAAAAAAACAACAATATGGACCGTGTCTCACTTTTGCCTTCCTTCTTTTTTCCTGTGCTTTGCCTGCTGGCTTACGTCCGCGGCGGAGCAGCAACGTTGGCGCATACAGCAGCAGTTCTCTCCCTCGTTTCTTCTGCTGATTGTGCGCTTACTCTGTGCTGCTGCGCCATGCCTATTCTGATTTGTCTCGTATCCAGCGCTGTATTCCCTCTCTTTTCCCTATTGCCCTCATGNNNNNNNNNNNNNNNNNNNNNNNNNNNNNNNNNNNNNNNNNNNNNNNNNNNNNNNNNNNNNNNNNNNNNNNNNNNNNNTGCCCTCTGGTGCGGCTGCGGGCTCTCTGGCG

### 3. MERCAPTO-PYRUVAAT SULFURTRANSFERASE

>BPK035/0 clone 1|LinJ05_V3.0970|500 bp UPS + CDS + 500 bp DWS|Mercapto-pyruvaat sulfurtransferase

AGCGCGGCGTGGGCCCAGCAGCGCCGGACCATGGGAGATAACGCGCGGCGAGGAAGGCGAAGAGGTGTGGTGCACCAGCGGTGTATTTGTGCACGCCTCTGTGTGTGCATCCGCCCGGGTGAACTCCCCTCCTCGCTTATACCCCTACAGCGACCCCACCCCCCCCCCCAGCGCACGCAAGCACCCGGCTACTCGAAAGTGCTAGCGGGCATTGCGTGCGTCTACCCGCTGATGGGGTGACTGTAAGCACAAAAAAGCACAGAGGCACTTGTAGCGCATGACCCGTCAGCCATCGCCACCATGGCGAGCCCCGAACAACACAAACACGCATGCGACAGGCAGCGAGCACTGCCACAACCACACTTCGAGGACCACGCCCAGCCATGCGGAGGCCCAACACACACACAGGGAGGCAAGAGAAACGAACTTGCCCAACTTTGGTCCCCCTGCCACTTCCCCCACCCACCCACCCCCGTGCACGCAAGTACTTGTGCCACCGTATGTCTGCTCCCGCTGCTGCGCCGAAACACCCGGGCAAGGTGTTCCTGGACCCGTGTGAGGTAAAGGACCACCTTGCCGAGTACCGCATCGTGGACTGCCGGTACAGCTTGAAGGTAAAGGACCACGGCAGCATTGAGTACGCGAAGGAGCACGTGAAGAGCGCCATCCGCGCCGATATGGATACGAACCTCTCCAAGTTGGTGCCCACCAGCACCGCCCGGCACCCGCTACCGCCCTGTGCTGAGTTTATCGAATGGTGCATGGCGAACGGGATGGCGGGAGAGCTGCCGGTGCTCTGCTACGATGACGAGTGCGGCGCCATGGGTGGATGCCGCCTGTGGTGGATGCTGAACTCTCTTGGCGCCGAGGCGTACGTGATCAACGGCGGCTTTCAGGCCTGCAAGGCTGCGGGGCTGGAGATGGAGTCAGGCGAGCCCCCGTCGCCGCCGACGCCCCCTACGCACTGGCCCTTCAAGACGGCCTTTCAGCATCACTATCTCGTGGATGAGATCCCGCCCAACGCGATCATCACCGACGCGCGCTCCGCCGACCGCTTCGCCTCGACAGTACGGCCTTACGCCGCAGACAAGATGCCAGGCCACATCGAAGGCGCGCGTAACCTCCCCTACACGTCGCACCTCGTGATACGCGGTGACGGCAAGGTGCTGCGCAGTGAGGAGGAGATCCGCCACAACATCATGACCGTCGTGCAGGGCACGGGTGACGCGACTGATCTGTCGAGCTTCGTCTTCTCCTGCGGCAGCGGCGTCACCGCCTGCATCAATATCGCCCTGGTGCACCACCTCGGCCTGGGCCATCCGTACCTCTACTGTGGCTCCTGGTCTGAGTATAGCGGCCTCTTCCGCCTCCCCATAATGCGCAGCATCATCGACGACTACGGCATGTGCATGCAAATGCAGACCCCTAGCCTCGGCGACAACCCGAAGGCAAACCTCGACACCATGACTCTGAAGGTCGACGGCGCGCCCTGCGAGAGACCCGATGCGGAGGTGCAGAGCGCCGCAGCCCACCTCCACGCTGGCGAGGCCGCTACTGTGTACTTCAAAAGCGGCCGCGTCGTCACGATCGAGGCGCCGGCAGTGCCCAACTAAAGCACGCGCAACAACGCACGCCTCGCCACACCGGCCCCCTGTGGGCTCATGAAAGCGCGGCGCGCCGCCTGCGTTTGGTGTCTCTTCGCCCGGGCTTGCCACCCACCCCACCCCGCCAGCAGGGTCGCCCGCGTTTCGGCCGCGCCGCCTTTATTTCCCATGGTGTTTGCTTTCGGTTGGTCCTCAGCCCCCCSCCCCCKCCCCGCAAAACACAGAATAAGCCCAAGCCCACCCCCCCTGCATCTGCCTACGGCAGCGTGATCGCCACGACGCTGGCTGATCGGGCGAGGCGGCCCCACGCAACCCACCCGCCCCCTTCCCAGCCCACATGCTCATGAAAATGCGAGCGCGCTTGCGCGTCATCGCCGGCCACTCCACGCACACACTCCTCCTCCTCCCCTACACACTTCTTACACGCTACGGGGCAGAGCCGATAGTCAAGCAGGTAAGAATATTCAAAGGTCTCTCTCTAACGCCCCTCTTTCGTCTCCCTGTCTCGG

>BPK043/0 clone 2|LinJ05_V3.0970|500 bp UPS + CDS + 500 bp DWS|Mercapto-pyruvaat sulfurtransferase

AGCGCGGCGTGGGCCCAGCAGCGCCGGACCATGGGAGATAACGCGCGGCGAGGAAGGCGAAGAGGTGTGGTGCACCAGCGGTGTATTTGTGCACGCCTCTGTGTGTGCATCCGCCCGGGTGAACTCCCCTCCTCGCTTATACCCCTACAGCGACCCCACCCCCCCCCCCAGCGCACGCAAGCACCCGGCTACTCGAAAGTGCTAGCGGGCATTGCGTGCGTCTACCCGCTGATGGGGTGACTGTAAGCACAAAAAAGCACAGAGGCACTTGTAGCGCATGACCCGTCAGCCATCGCCACCATGGCGAGCCCCGAACAACACAAACACGCATGCGACAGGCAGCGAGCACTGCCACAACCACACTTCGAGGACCACGCCCAGCCATGCGGAGGCCCAACACACACACAGGGAGGCAAGAGAAACGAACTTGCCCAACTTTGGTCCCCCTGCCACTTCCCCCACCCACCCACCCCCGTGCACGCAAGTACTTGTGCCACCGTATGTCTGCTCCCGCTGCTGCGCCGAAACACCCGGGCAAGGTGTTCCTGGACCCGTGTGAGGTAAAGGACCACCTTGCCGAGTACCGCATCGTGGACTGCCGGTACAGCTTGAAGGTAAAGGACCACGGCAGCATTGAGTACGCGAAGGAGCACGTGAAGAGCGCCATCCGCGCCGATATGGATACGAACCTCTCCAAGTTGGTGCCCACCAGCACCGCCCGGCACCCGCTACCGCCCTGTGCTGAGTTTATCGAATGGTGCATGGCGAACGGGATGGCGGGAGAGCTGCCGGTGCTCTGCTACGATGACGAGTGCGGCGCCATGGGTGGATGCCGCCTGTGGTGGATGCTGAACTCTCTTGGCGCCGAGGCGTACGTGATCAACGGCGGCTTTCAGGCCTGCAAGGCTGCGGGGCTGGAGATGGAGTCAGGCGAGCCCCCGTCGCCGCCGACGCCCCCTACGCACTGGCCCTTCAAGACGGCCTTTCAGCATCACTATCTCGTGGATGAGATCCCGCCCAACGCGATCATCACCGACGCGCGCTCCGCCGACCGCTTCGCCTCGACAGTACGGCCTTACGCCGCAGACAAGATGCCAGGCCACATCGAAGGCGCGCGTAACCTCCCCTACACGTCGCACCTCGTGATACGCGGTGACGGCAAGGTGCTGCGCAGTGAGGAGGAGATCCGCCACAACATCATGACCGTCGTGCAGGGCACGGGTGACGCGACTGATCTGTCGAGCTTCGTCTTCTCCTGCGGCAGCGGCGTCACCGCCTGCATCAATATCGCCCTGGTGCACCACCTCGGCCTGGGCCATCCGTACCTCTACTGTGGCTCCTGGTCTGAGTATAGCGGCCTCTTCCGCCTCCCCATAATGCGCAGCATCATCGACGACTACGGCATGTGCATGCAAATGCAGACCCCTAGCCTCGGCGACAACCCGAAGGCAAACCTCGACACCATGACTCTGAAGGTCGACGGCGCGCCCTGCGAGAGACCCGATGCGGAGGTGCAGAGCGCCGCAGCCCACCTCCACGCTGGCGAGGCCGCTACTGTGTACTTCAAAAGCGGCCGCGTCGTCACGATCGAGGCGCCGGCAGTGCCCAACTAAAGCACGCGCAACAACGCACGCCTCGCCACACCGGCCCCCTGTGGGCTCATGAAAGCGCGGCGCGCCGCCTGCGTTTGGTGTCTCTTCGCCCGGGCTTGCCACCCACCCCACCCCGCCAGCAGGGTCGCCCGCGTTTCGGCCGCGCCGCCTTTATTTCCCATGGTGTTTGCTTTCGGTTGGTCCTCAGCCCCCCGCCCCCACCCCGCAAAACACAGAATAAGCCCAAGCCCACCCCCCCTGCATCTGCCTACGGCAGCGTGATCGCCACGACGCTGGCTGATCGGGCGAGGCGGCCCCACGCAACCCACCCGCCCCCTTCCCAGCCCACATGCTCATGAAAATGCGAGCGCGCTTGCGCGTCATCGCCGGCCACTCCACGCACACACTCCTCCTCCTCCCCTACACACTTCTTACACGCTACGGGGCAGAGCCGATAGTCAAGCAGGTAAGAATATTCAAAGGTCTCTCTCTAACGCCCCTCTTTCGTCTCCCTGTCTCGG

>BPK085/0 clone 8|LinJ05_V3.0970|500 bp UPS + CDS + 500 bp DWS|Mercapto-pyruvaat sulfurtransferase

AGCGCGGCGTGGGCCCAGCAGCGCCGGACCATGGGAGATAACGCGCGGCGAGGAAGGCGAAGAGGTGTGGTGCACCAGCGGTGTATTTGTGCACGCCTCTGTGTGTGCATCCGCCCGGGTGAACTCCCCTCCTCGCTTATACCCCTACAGCGACCCCACCCCCCCCCCCAGCGCACGCAAGCACCCGGCTACTCGAAAGTGCTAGCGGGCATTGCGTGCGTCTACCCGCTGATGGGGTGACTGTAAGCACAAAAAAGCACAGAGGCACTTGTAGCGCATGACCCGTCAGCCATCGCCACCATGGCGAGCCCCGAACAACACAAACACGCATGCGACAGGCAGCGAGCACTGCCACAACCACACTTCGAGGACCACGCCCAGCCATGCGGAGGCCCAACACACACACAGGGAGGCAAGAGAAACGAACTTGCCCAACTTTGGTCCCCCTGCCACTTCCCCCACCCACCCACCCCCGTGCACGCAAGTACTTGTGCCACCGTATGTCTGCTCCCGCTGCTGCGCCGAAACACCCGGGCAAGGTGTTCCTGGACCCGTGTGAGGTAAAGGACCACCTTGCCGAGTACCGCATCGTGGACTGCCGGTACAGCTTGAAGGTAAAGGACCACGGCAGCATTGAGTACGCGAAGGAGCACGTGAAGAGCGCCATCCGCGCCGATATGGATACGAACCTCTCCAAGTTGGTGCCCACCAGCACCGCCCGGCACCCGCTACCGCCCTGTGCTGAGTTTATCGAATGGTGCATGGCGAACGGGATGGCGGGAGAGCTGCCGGTGCTCTGCTACGATGACGAGTGCGGCGCCATGGGTGGATGCCGCCTGTGGTGGATGCTGAACTCTCTTGGCGCCGAGGCGTACGTGATCAACGGCGGCTTTCAGGCCTGCAAGGCTGCGGGGCTGGAGATGGAGTCAGGCGAGCCCCCGTCGCCGCCGACGCCCCCTACGCACTGGCCCTTCAAGACGGCCTTTCAGCATCACTATCTCGTGGATGAGATCCCGCCCAACGCGATCATCACCGACGCGCGCTCCGCCGACCGCTTCGCCTCGACAGTACGGCCTTACGCCGCAGACAAGATGCCAGGCCACATCGAAGGCGCGCGTAACCTCCCCTACACGTCGCACCTCGTGATACGCGGTGACGGCAAGGTGCTGCGCAGTGAGGAGGAGATCCGCCACAACATCATGACCGTCGTGCAGGGCACGGGTGACGCGACTGATCTGTCGAGCTTCGTCTTCTCCTGCGGCAGCGGCGTCACCGCCTGCATCAATATCGCCCTGGTGCACCACCTCGGCCTGGGCCATCCGTACCTCTACTGTGGCTCCTGGTCTGAGTATAGCGGCCTCTTCCGCCTCCCCATAATGCGCAGCATCATCGACGACTACGGCATGTGCATGCAAATGCAGACCCCTAGCCTCGGCGACAACCCGAAGGCAAACCTCGACACCATGACTCTGAAGGTCGACGGCGCGCCCTGCGAGAGACCCGATGCGGAGGTGCAGAGCGCCGCAGCCCACCTCCACGCTGGCGAGGCCGCTACTGTGTACTTCAAAAGCGGCCGCGTCGTCACGATCGAGGCGCCGGCAGTGCCCAACTAAAGCACGCGCAACAACGCACGCCTCGCCACACCGGCCCCCTGTGGGCTCATGAAAGCGCGGCGCGCCGCCTGCGTTTGGTGTCTCTTCGCCCGGGCTTGCCACCCACCCCACCCCGCCAGCAGGGTCGCCCGCGTTTCGGCCGCGCCGCCTTTATTTCCCATGGTGTTTGCTTTCGGTTGGTCCTCAGCCCCCCGCCCCCACCCCGCAAAACACAGAATAAGCCCAAGCCCACCCCCCCTGCATCTGCCTACGGCAGCGTGATCGCCACGACGCTGGCTGATCGGGCGAGGCGGCCCCACGCAACCCACCCGCCCCCTTCCCAGCCCACATGCTCATGAAAATGCGAGCGCGCTTGCGCGTCATCGCCGGCCACTCCACGCACACACTCCTCCTCCTCCCCTACACACTTCTTACACGCTACGGGGCAGAGCCGATAGTCAAGCAGGTAAGAATATTCAAAGGTCTCTCTCTAACGCCCCTCTTTCGTCTCCCTGTCTCGG

>BPK087/0 clone 11|LinJ05_V3.0970|500 bp UPS + CDS + 500 bp DWS|Mercapto-pyruvaat sulfurtransferase

AGCGCGGCGTGGGCCCAGCAGCGCCGGACCATGGGAGATAACGCGCGGCGAGGAAGGCGAAGAGGTGTGGTGCACCAGCGGTGTATTTGTGCACGCCTCTGTGTGTGCATCCGCCCGGGTGAACTCCCCTCCTCGCTTATACCCCTACAGCGACCCCACCCCCCCCCCCAGCGCACGCAAGCACCCGGCTACTCGAAAGTGCTAGCGGGCATTGCGTGCGTCTACCCGCTGATGGGGTGACTGTAAGCACAAAAAAGCACAGAGGCACTTGTAGCGCATGACCCGTCAGCCATCGCCACCATGGCGAGCCCCGAACAACACAAACACGCATGCGACAGGCAGCGAGCACTGCCACAACCACACTTCGAGGACCACGCCCAGCCATGCGGAGGCCCAACACACACACAGGGAGGCAAGAGAAACGAACTTGCCCAACTTTGGTCCCCCTGCCACTTCCCCCACCCACCCACCCCCGTGCACGCAAGTACTTGTGCCACCGTATGTCTGCTCCCGCTGCTGCGCCGAAACACCCGGGCAAGGTGTTCCTGGACCCGTGTGAGGTAAAGGACCACCTTGCCGAGTACCGCATCGTGGACTGCCGGTACAGCTTGAAGGTAAAGGACCACGGCAGCATTGAGTACGCGAAGGAGCACGTGAAGAGCGCCATCCGCGCCGATATGGATACGAACCTCTCCAAGTTGGTGCCCACCAGCACCGCCCGGCACCCGCTACCGCCCTGTGCTGAGTTTATCGAATGGTGCATGGCGAACGGGATGGCGGGAGAGCTGCCGGTGCTCTGCTACGATGACGAGTGCGGCGCCATGGGTGGATGCCGCCTGTGGTGGATGCTGAACTCTCTTGGCGCCGAGGCGTACGTGATCAACGGCGGCTTTCAGGCCTGCAAGGCTGCGGGGCTGGAGATGGAGTCAGGCGAGCCCCCGTCGCCGCCGACGCCCCCTACGCACTGGCCCTTCAAGACGGCCTTTCAGCATCACTATCTCGTGGATGAGATCCCGCCCAACGCGATCATCACCGACGCGCGCTCCGCCGACCGCTTCGCCTCGACAGTACGGCCTTACGCCGCAGACAAGATGCCAGGCCACATCGAAGGCGCGCGTAACCTCCCCTACACGTCGCACCTCGTGATACGCGGTGACGGCAAGGTGCTGCGCAGTGAGGAGGAGATCCGCCACAACATCATGACCGTCGTGCAGGGCACGGGTGACGCGACTGATCTGTCGAGCTTCGTCTTCTCCTGCGGCAGCGGCGTCACCGCCTGCATCAATATCGCCCTGGTGCACCACCTCGGCCTGGGCCATCCGTACCTCTACTGTGGCTCCTGGTCTGAGTATAGCGGCCTCTTCCGCCTCCCCATAATGCGCAGCATCATCGACGACTACGGCATGTGCATGCAAATGCAGACCCCTAGCCTCGGCGACAACCCGAAGGCAAACCTCGACACCATGACTCTGAAGGTCGACGGCGCGCCCTGCGAGAGACCCGATGCGGAGGTGCAGAGCGCCGCAGCCCACCTCCACGCTGGCGAGGCCGCTACTGTGTACTTCAAAAGCGGCCGCGTCGTCACGATCGAGGCGCCGGCAGTGCCCAACTAAAGCACGCGCAACAACGCACGCCTCGCCACACCGGCCCCCTGTGGGCTCATGAAAGCGCGGCGCGCCGCCTGCGTTTGGTGTCTCTTCGCCCGGGCTTGCCACCCACCCCACCCCGCCAGCAGGGTCGCCCGCGTTTCGGCCGCGCCGCCTTTATTTCCCATGGTGTTTGCTTTCGGTTGGTCCTCAGCCCCCCGCCCCCACCCCGCAAAACACAGAATAAGCCCAAGCCCACCCCCCCTGCATCTGCCTACGGCAGCGTGATCGCCACGACGCTGGCTGATCGGGCGAGGCGGCCCCACGCAACCCACCCGCCCCCTTCCCAGCCCACATGCTCATGAAAATGCGAGCGCGCTTGCGCGTCATCGCCGGCCACTCCACGCACACACTCCTCCTCCTCCCCTACACACTTCTTACACGCTACGGGGCAGAGCCGATAGTCAAGCAGGTAAGAATATTCAAAGGTCTCTCTCTAACGCCCCTCTTTCGTCTCCCTGTCTCGG

>BPK178/0 clone 3|LinJ05_V3.0970|500 bp UPS + CDS + 500 bp DWS|Mercapto-pyruvaat sulfurtransferase

AGCGCGGCGTGGGCCCAGCAGCGCCGGACCATGGGAGATAACGCGCGGCGAGGAAGGCGAAGAGGTGTGGTGCACCAGCGGTGTATTTGTGCACGCCTCTGTGTGTGCATCCGCCCGGGTGAACTCCCCTCCTCGCTTATACCCCTACAGCGACCCCACCCCCCCCCCCAGCGCACGCAAGCACCCGGCTACTCGAAAGTGCTAGCGGGCATTGCGTGCGTCTACCCGCTGATGGGGTGACTGTAAGCACAAAAAAGCACAGAGGCACTTGTAGCGCATGACCCGTCAGCCATCGCCACCATGGCGAGCCCCGAACAACACAAACACGCATGCGACAGGCAGCGAGCACTGCCACAACCACACTTCGAGGACCACGCCCAGCCATGCGGAGGCCCAACACACACACAGGGAGGCAAGAGAAACGAACTTGCCCAACTTTGGTCCCCCTGCCACTTCCCCCACCCACCCACCCCCGTGCACGCAAGTACTTGTGCCACCGTATGTCTGCTCCCGCTGCTGCGCCGAAACACCCGGGCAAGGTGTTCCTGGACCCGTGTGAGGTAAAGGACCACCTTGCCGAGTACCGCATCGTGGACTGCCGGTACAGCTTGAAGGTAAAGGACCACGGCAGCATTGAGTACGCGAAGGAGCACGTGAAGAGCGCCATCCGCGCCGATATGGATACGAACCTCTCCAAGTTGGTGCCCACCAGCACCGCCCGGCACCCGCTACCGCCCTGTGCTGAGTTTATCGAATGGTGCATGGCGAACGGGATGGCGGGAGAGCTGCCGGTGCTCTGCTACGATGACGAGTGCGGCGCCATGGGTGGATGCCGCCTGTGGTGGATGCTGAACTCTCTTGGCGCCGAGGCGTACGTGATCAACGGCGGCTTTCAGGCCTGCAAGGCTGCGGGGCTGGAGATGGAGTCAGGCGAGCCCCCGTCGCCGCCGACGCCCCCTACGCACTGGCCCTTCAAGACGGCCTTTCAGCATCACTATCTCGTGGATGAGATCCCGCCCAACGCGATCATCACCGACGCGCGCTCCGCCGACCGCTTCGCCTCGACAGTACGGCCTTACGCCGCAGACAAGATGCCAGGCCACATCGAAGGCGCGCGTAACCTCCCCTACACGTCGCACCTCGTGATACGCGGTGACGGCAAGGTGCTGCGCAGTGAGGAGGAGATCCGCCACAACATCATGACCGTCGTGCAGGGCACGGGTGACGCGACTGATCTGTCGAGCTTCGTCTTCTCCTGCGGCAGCGGCGTCACCGCCTGCATCAATATCGCCCTGGTGCACCACCTCGGCCTGGGCCATCCGTACCTCTACTGTGGCTCCTGGTCTGAGTATAGCGGCCTCTTCCGCCTCCCCATAATGCGCAGCATCATCGACGACTACGGCATGTGCATGCAAATGCAGACCCCTAGCCTCGGCGACAACCCGAAGGCAAACCTCGACACCATGACTCTGAAGGTCGACGGCGCGCCCTGCGAGAGACCCGATGCGGAGGTGCAGAGCGCCGCAGCCCACCTCCACGCTGGCGAGGCCGCTACTGTGTACTTCAAAAGCGGCCGCGTCGTCACGATCGAGGCGCCGGCAGTGCCCAACTAAAGCACGCGCAACAACGCACGCCTCGCCACACCGGCCCCCTGTGGGCTCATGAAAGCGCGGCGCGCCGCCTGCGTTTGGTGTCTCTTCGCCCGGGCTTGCCACCCACCCCACCCCGCCAGCAGGGTCGCCCGCGTTTCGGCCGCGCCGCCTTTATTTCCCATGGTGTTTGCTTTCGGTTGGTCCTCAGCCCCCCGCCCCCACCCCGCAAAACACAGAATAAGCCCAAGCCCACCCCCCCTGCATCTGCCTACGGCAGCGTGATCGCCACGACGCTGGCTGATCGGGCGAGGCGGCCCCACGCAACCCACCCGCCCCCTTCCCAGCCCACATGCTCATGAAAATGCGAGCGCGCTTGCGCGTCATCGCCGGCCACTCCACGCACACACTCCTCCTCCTCCCCTACACACTTCTTACACGCTACGGGGCAGAGCCGATAGTCAAGCAGGTAAGAATATTCAAAGGTCTCTCTCTAACGCCCCTCTTTCGTCTCCCTGTCTCGG

>BPK190/0 clone 3|LinJ05_V3.0970|500 bp UPS + CDS + 500 bp DWS|Mercapto-pyruvaat sulfurtransferase

AGCGCGGCGTGGGCCCAGCAGCGCCGGACCATGGGAGATAACGCGCGGCGAGGAAGGCGAAGAGGTGTGGTGCACCAGCGGTGTATTTGTGCACGCCTCTGTGTGTGCATCCGCCCGGGTGAACTCCCCTCCTCGCTTATACCCCTACAGCGACCCCACCCCCCCCCCCAGCGCACGCAAGCACCCGGCTACTCGAAAGTGCTAGCGGGCATTGCGTGCGTCTACCCGCTGATGGGGTGACTGTAAGCACAAAAAAGCACAGAGGCACTTGTAGCGCATGACCCGTCAGCCATCGCCACCATGGCGAGCCCCGAACAACACAAACACGCATGCGACAGGCAGCGAGCACTGCCACAACCACACTTCGAGGACCACGCCCAGCCATGCGGAGGCCCAACACACACACAGGGAGGCAAGAGAAACGAACTTGCCCAACTTTGGTCCCCCTGCCACTTCCCCCACCCACCCACCCCCGTGCACGCAAGTACTTGTGCCACCGTATGTCTGCTCCCGCTGCTGCGCCGAAACACCCGGGCAAGGTGTTCCTGGACCCGTGTGAGGTAAAGGACCACCTTGCCGAGTACCGCATCGTGGACTGCCGGTACAGCTTGAAGGTAAAGGACCACGGCAGCATTGAGTACGCGAAGGAGCACGTGAAGAGCGCCATCCGCGCCGATATGGATACGAACCTCTCCAAGTTGGTGCCCACCAGCACCGCCCGGCACCCGCTACCGCCCTGTGCTGAGTTTATCGAATGGTGCATGGCGAACGGGATGGCGGGAGAGCTGCCGGTGCTCTGCTACGATGACGAGTGCGGCGCCATGGGTGGATGCCGCCTGTGGTGGATGCTGAACTCTCTTGGCGCCGAGGCGTACGTGATCAACGGCGGCTTTCAGGCCTGCAAGGCTGCGGGGCTGGAGATGGAGTCAGGCGAGCCCCCGTCGCCGCCGACGCCCCCTACGCACTGGCCCTTCAAGACGGCCTTTCAGCATCACTATCTCGTGGATGAGATCCCGCCCAACGCGATCATCACCGACGCGCGCTCCGCCGACCGCTTCGCCTCGACAGTACGGCCTTACGCCGCAGACAAGATGCCAGGCCACATCGAAGGCGCGCGTAACCTCCCCTACACGTCGCACCTCGTGATACGCGGTGACGGCAAGGTGCTGCGCAGTGAGGAGGAGATCCGCCACAACATCATGACCGTCGTGCAGGGCACGGGTGACGCGACTGATCTGTCGAGCTTCGTCTTCTCCTGCGGCAGCGGCGTCACCGCCTGCATCAATATCGCCCTGGTGCACCACCTCGGCCTGGGCCATCCGTACCTCTACTGTGGCTCCTGGTCTGAGTATAGCGGCCTCTTCCGCCTCCCCATAATGCGCAGCATCATCGACGACTACGGCATGTGCATGCAAATGCAGACCCCTAGCCTCGGCGACAACCCGAAGGCAAACCTCGACACCATGACTCTGAAGGTCGACGGCGCGCCCTGCGAGAGACCCGATGCGGAGGTGCAGAGCGCCGCAGCCCACCTCCACGCTGGCGAGGCCGCTACTGTGTACTTCAAAAGCGGCCGCGTCGTCACGATCGAGGCGCCGGCAGTGCCCAACTAAAGCACGCGCAACAACGCACGCCTCGCCACACCGGCCCCCTGTGGGCTCATGAAAGCGCGGCGCGCCGCCTGCGTTTGGTGTCTCTTCGCCCGGGCTTGCCACCCACCCCACCCCGCCAGCAGGGTCGCCCGCGTTTCGGCCGCGCCGCCTTTATTTCCCATGGTGTTTGCTTTCGGTTGGTCCTCAGCCCCCCGCCCCCACCCCGCAAAACACAGAATAAGCCCAAGCCCACCCCCCCTGCATCTGCCTACGGCAGCGTGATCGCCACGACGCTGGCTGATCGGGCGAGGCGGCCCCACGCAACCCACCCGCCCCCTTCCCAGCCCACATGCTCATGAAAATGCGAGCGCGCTTGCGCGTCATCGCCGGCCACTCCACGCACACACTCCTCCTCCTCCCCTACACACTTCTTACACGCTACGGGGCAGAGCCGATAGTCAAGCAGGTAAGAATATTCAAAGGTCTCTCTCTAACGCCCCTCTTTCGTCTCCCTGTCTCGG

>BPK206/0 clone 10|LinJ05_V3.0970|500 bp UPS + CDS + 500 bp DWS|Mercapto-pyruvaat sulfurtransferase

AGCGCGGCGTGGGCCCAGCAGCGCCGGACCATGGGAGATAACGCGCGGCGAGGAAGGCGAAGAGGTGTGGTGCACCAGCGGTGTATTTGTGCACGCCTCTGTGTGTGCATCCGCCCGGGTGAACTCCCCTCCTCGCTTATACCCCTACAGCGACCCCACCCCCCCCCCCAGCGCACGCAAGCACCCGGCTACTCGAAAGTGCTAGCGGGCATTGCGTGCGTCTACCCGCTGATGGGGTGACTGTAAGCACAAAAAAGCACAGAGGCACTTGTAGCGCATGACCCGTCAGCCATCGCCACCATGGCGAGCCCCGAACAACACAAACACGCATGCGACAGGCAGCGAGCACTGCCACAACCACACTTCGAGGACCACGCCCAGCCATGCGGAGGCCCAACACACACACAGGGAGGCAAGAGAAACGAACTTGCCCAACTTTGGTCCCCCTGCCACTTCCCCCACCCACCCACCCCCGTGCACGCAAGTACTTGTGCCACCGTATGTCTGCTCCCGCTGCTGCGCCGAAACACCCGGGCAAGGTGTTCCTGGACCCGTGTGAGGTAAAGGACCACCTTGCCGAGTACCGCATCGTGGACTGCCGGTACAGCTTGAAGGTAAAGGACCACGGCAGCATTGAGTACGCGAAGGAGCACGTGAAGAGCGCCATCCGCGCCGATATGGATACGAACCTCTCCAAGTTGGTGCCCACCAGCACCGCCCGGCACCCGCTACCGCCCTGTGCTGAGTTTATCGAATGGTGCATGGCGAACGGGATGGCGGGAGAGCTGCCGGTGCTCTGCTACGATGACGAGTGCGGCGCCATGGGTGGATGCCGCCTGTGGTGGATGCTGAACTCTCTTGGCGCCGAGGCGTACGTGATCAACGGCGGCTTTCAGGCCTGCAAGGCTGCGGGGCTGGAGATGGAGTCAGGCGAGCCCCCGTCGCCGCCGACGCCCCCTACGCACTGGCCCTTCAAGACGGCCTTTCAGCATCACTATCTCGTGGATGAGATCCCGCCCAACGCGATCATCACCGACGCGCGCTCCGCCGACCGCTTCGCCTCGACAGTACGGCCTTACGCCGCAGACAAGATGCCAGGCCACATCGAAGGCGCGCGTAACCTCCCCTACACGTCGCACCTCGTGATACGCGGTGACGGCAAGGTGCTGCGCAGTGAGGAGGAGATCCGCCACAACATCATGACCGTCGTGCAGGGCACGGGTGACGCGACTGATCTGTCGAGCTTCGTCTTCTCCTGCGGCAGCGGCGTCACCGCCTGCATCAATATCGCCCTGGTGCACCACCTCGGCCTGGGCCATCCGTACCTCTACTGTGGCTCCTGGTCTGAGTATAGCGGCCTCTTCCGCCTCCCCATAATGCGCAGCATCATCGACGACTACGGCATGTGCATGCAAATGCAGACCCCTAGCCTCGGCGACAACCCGAAGGCAAACCTCGACACCATGACTCTGAAGGTCGACGGCGCGCCCTGCGAGAGACCCGATGCGGAGGTGCAGAGCGCCGCAGCCCACCTCCACGCTGGCGAGGCCGCTACTGTGTACTTCAAAAGCGGCCGCGTCGTCACGATCGAGGCGCCGGCAGTGCCCAACTAAAGCACGCGCAACAACGCACGCCTCGCCACACCGGCCCCCTGTGGGCTCATGAAAGCGCGGCGCGCCGCCTGCGTTTGGTGTCTCTTCGCCCGGGCTTGCCACCCACCCCACCCCGCCAGCAGGGTCGCCCGCGTTTCGGCCGCGCCGCCTTTATTTCCCATGGTGTTTGCTTTCGGTTGGTCCTCAGCCCCCCGCCCCCACCCCGCAAAACACAGAATAAGCCCAAGCCCACCCCCCCTGCATCTGCCTACGGCAGCGTGATCGCCACGACGCTGGCTGATCGGGCGAGGCGGCCCCACGCAACCCACCCGCCCCCTTCCCAGCCCACATGCTCATGAAAATGCGAGCGCGCTTGCGCGTCATCGCCGGCCACTCCACGCACACACTCCTCCTCCTCCCCTACACACTTCTTACACGCTACGGGGCAGAGCCGATAGTCAAGCAGGTAAGAATATTCAAAGGTCTCTCTCTAACGCCCCTCTTTCGTCTCCCTGTCTCGG

>BPK275/0 clone 18|LinJ05_V3.0970|500 bp UPS + CDS + 500 bp DWS|Mercapto-pyruvaat sulfurtransferase

AGCGCGGCGTGGGCCCAGCAGCGCCGGACCATGGGAGATAACGCGCGGCGAGGAAGGCGAAGAGGTGTGGTGCACCAGCGGTGTATTTGTGCACGCCTCTGTGTGTGCATCCGCCCGGGTGAACTCCCCTCCTCGCTTATACCCCTACAGCGACCCCACCCCCCCCCCCAGCGCACGCAAGCACCCGGCTACTCGAAAGTGCTAGCGGGCATTGCGTGCGTCTACCCGCTGATGGGGTGACTGTAAGCACAAAAAAGCACAGAGGCACTTGTAGCGCATGACCCGTCAGCCATCGCCACCATGGCGAGCCCCGAACAACACAAACACGCATGCGACAGGCAGCGAGCACTGCCACAACCACACTTCGAGGACCACGCCCAGCCATGCGGAGGCCCAACACACACACAGGGAGGCAAGAGAAACGAACTTGCCCAACTTTGGTCCCCCTGCCACTTCCCCCACCCACCCACCCCCGTGCACGCAAGTACTTGTGCCACCGTATGTCTGCTCCCGCTGCTGCGCCGAAACACCCGGGCAAGGTGTTCCTGGACCCGTGTGAGGTAAAGGACCACCTTGCCGAGTACCGCATCGTGGACTGCCGGTACAGCTTGAAGGTAAAGGACCACGGCAGCATTGAGTACGCGAAGGAGCACGTGAAGAGCGCCATCCGCGCCGATATGGATACGAACCTCTCCAAGTTGGTGCCCACCAGCACCGCCCGGCACCCGCTACCGCCCTGTGCTGAGTTTATCGAATGGTGCATGGCGAACGGGATGGCGGGAGAGCTGCCGGTGCTCTGCTACGATGACGAGTGCGGCGCCATGGGTGGATGCCGCCTGTGGTGGATGCTGAACTCTCTTGGCGCCGAGGCGTACGTGATCAACGGCGGCTTTCAGGCCTGCAAGGCTGCGGGGCTGGAGATGGAGTCAGGCGAGCCCCCGTCGCCGCCGACGCCCCCTACGCACTGGCCCTTCAAGACGGCCTTTCAGCATCACTATCTCGTGGATGAGATCCCGCCCAACGCGATCATCACCGACGCGCGCTCCGCCGACCGCTTCGCCTCGACAGTACGGCCTTACGCCGCAGACAAGATGCCAGGCCACATCGAAGGCGCGCGTAACCTCCCCTACACGTCGCACCTCGTGATACGCGGTGACGGCAAGGTGCTGCGCAGTGAGGAGGAGATCCGCCACAACATCATGACCGTCGTGCAGGGCACGGGTGACGCGACTGATCTGTCGAGCTTCGTCTTCTCCTGCGGCAGCGGCGTCACCGCCTGCATCAATATCGCCCTGGTGCACCACCTCGGCCTGGGCCATCCGTACCTCTACTGTGGCTCCTGGTCTGAGTATAGCGGCCTCTTCCGCCTCCCCATAATGCGCAGCATCATCGACGACTACGGCATGTGCATGCAAATGCAGACCCCTAGCCTCGGCGACAACCCGAAGGCAAACCTCGACACCATGACTCTGAAGGTCGACGGCGCGCCCTGCGAGAGACCCGATGCGGAGGTGCAGAGCGCCGCAGCCCACCTCCACGCTGGCGAGGCCGCTACTGTGTACTTCAAAAGCGGCCGCGTCGTCACGATCGAGGCGCCGGCAGTGCCCAACTAAAGCACGCGCAACAACGCACGCCTCGCCACACCGGCCCCCTGTGGGCTCATGAAAGCGCGGCGCGCCGCCTGCGTTTGGTGTCTCTTCGCCCGGGCTTGCCACCCACCCCACCCCGCCAGCAGGGTCGCCCGCGTTTCGGCCGCGCCGCCTTTATTTCCCATGGTGTTTGCTTTCGGTTGGTCCTCAGCCCCCCGCCCCCACCCCGCAAAACACAGAATAAGCCCAAGCCCACCCCCCCTGCATCTGCCTACGGCAGCGTGATCGCCACGACGCTGGCTGATCGGGCGAGGCGGCCCCACGCAACCCACCCGCCCCCTTCCCAGCCCACATGCTCATGAAAATGCGAGCGCGCTTGCGCGTCATCGCCGGCCACTCCACGCACACACTCCTCCTCCTCCCCTACACACTTCTTACACGCTACGGGGCAGAGCCGATAGTCAAGCAGGTAAGAATATTCAAAGGTCTCTCTCTAACGCCCCTCTTTCGTCTCCCTGTCTCGG

>BPK282/0 clone 4|LinJ05_V3.0970|500 bp UPS + CDS + 500 bp DWS|Mercapto-pyruvaat sulfurtransferase

AGCGCGGCGTGGGCCCAGCAGCGCCGGACCATGGGAGATAACGCGCGGCGAGGAAGGCGAAGAGGTGTGGTGCACCAGCGGTGTATTTGTGCACGCCTCTGTGTGTGCATCCGCCCGGGTGAACTCCCCTCCTCGCTTATACCCCTACAGCGACCCCACCCCCCCCCCCAGCGCACGCAAGCACCCGGCTACTCGAAAGTGCTAGCGGGCATTGCGTGCGTCTACCCGCTGATGGGGTGACTGTAAGCACAAAAAAGCACAGAGGCACTTGTAGCGCATGACCCGTCAGCCATCGCCACCATGGCGAGCCCCGAACAACACAAACACGCATGCGACAGGCAGCGAGCACTGCCACAACCACACTTCGAGGACCACGCCCAGCCATGCGGAGGCCCAACACACACACAGGGAGGCAAGAGAAACGAACTTGCCCAACTTTGGTCCCCCTGCCACTTCCCCCACCCACCCACCCCCGTGCACGCAAGTACTTGTGCCACCGTATGTCTGCTCCCGCTGCTGCGCCGAAACACCCGGGCAAGGTGTTCCTGGACCCGTGTGAGGTAAAGGACCACCTTGCCGAGTACCGCATCGTGGACTGCCGGTACAGCTTGAAGGTAAAGGACCACGGCAGCATTGAGTACGCGAAGGAGCACGTGAAGAGCGCCATCCGCGCCGATATGGATACGAACCTCTCCAAGTTGGTGCCCACCAGCACCGCCCGGCACCCGCTACCGCCCTGTGCTGAGTTTATCGAATGGTGCATGGCGAACGGGATGGCGGGAGAGCTGCCGGTGCTCTGCTACGATGACGAGTGCGGCGCCATGGGTGGATGCCGCCTGTGGTGGATGCTGAACTCTCTTGGCGCCGAGGCGTACGTGATCAACGGCGGCTTTCAGGCCTGCAAGGCTGCGGGGCTGGAGATGGAGTCAGGCGAGCCCCCGTCGCCGCCGACGCCCCCTACGCACTGGCCCTTCAAGACGGCCTTTCAGCATCACTATCTCGTGGATGAGATCCCGCCCAACGCGATCATCACCGACGCGCGCTCCGCCGACCGCTTCGCCTCGACAGTACGGCCTTACGCCGCAGACAAGATGCCAGGCCACATCGAAGGCGCGCGTAACCTCCCCTACACGTCGCACCTCGTGATACGCGGTGACGGCAAGGTGCTGCGCAGTGAGGAGGAGATCCGCCACAACATCATGACCGTCGTGCAGGGCACGGGTGACGCGACTGATCTGTCGAGCTTCGTCTTCTCCTGCGGCAGCGGCGTCACCGCCTGCATCAATATCGCCCTGGTGCACCACCTCGGCCTGGGCCATCCGTACCTCTACTGTGGCTCCTGGTCTGAGTATAGCGGCCTCTTCCGCCTCCCCATAATGCGCAGCATCATCGACGACTACGGCATGTGCATGCAAATGCAGACCCCTAGCCTCGGCGACAACCCGAAGGCAAACCTCGACACCATGACTCTGAAGGTCGACGGCGCGCCCTGCGAGAGACCCGATGCGGAGGTGCAGAGCGCCGCAGCCCACCTCCACGCTGGCGAGGCCGCTACTGTGTACTTCAAAAGCGGCCGCGTCGTCACGATCGAGGCGCCGGCAGTGCCCAACTAAAGCACGCGCAACAACGCACGCCTCGCCACACCGGCCCCCTGTGGGCTCATGAAAGCGCGGCGCGCCGCCTGCGTTTGGTGTCTCTTCGCCCGGGCTTGCCACCCACCCCACCCCGCCAGCAGGGTCGCCCGCGTTTCGGCCGCGCCGCCTTTATTTCCCATGGTGTTTGCTTTCGGTTGGTCCTCAGCCCCCCGCCCCCACCCCGCAAAACACAGAATAAGCCCAAGCCCACCCCCCCTGCATCTGCCTACGGCAGCGTGATCGCCACGACGCTGGCTGATCGGGCGAGGCGGCCCCACGCAACCCACCCGCCCCCTTCCCAGCCCACATGCTCATGAAAATGCGAGCGCGCTTGCGCGTCATCGCCGGCCACTCCACGCACACACTCCTCCTCCTCCCCTACACACTTCTTACACGCTACGGGGCAGAGCCGATAGTCAAGCAGGTAAGAATATTCAAAGGTCTCTCTCTAACGCCCCTCTTTCGTCTCCCTGTCTCGG

>BPK294/0 clone 1|LinJ05_V3.0970|500 bp UPS + CDS + 500 bp DWS|Mercapto-pyruvaat sulfurtransferase

AGCGCGGCGTGGGCCCAGCAGCGCCGGACCATGGGAGATAACGCGCGGCGAGGAAGGCGAAGAGGTGTGGTGCACCAGCGGTGTATTTGTGCACGCCTCTGTGTGTGCATCCGCCCGGGTGAACTCCCCTCCTCGCTTATACCCCTACAGCGACCCCACCCCCCCCCCCAGCGCACGCAAGCACCCGGCTACTCGAAAGTGCTAGCGGGCATTGCGTGCGTCTACCCGCTGATGGGGTGACTGTAAGCACAAAAAAGCACAGAGGCACTTGTAGCGCATGACCCGTCAGCCATCGCCACCATGGCGAGCCCCGAACAACACAAACACGCATGCGACAGGCAGCGAGCACTGCCACAACCACACTTCGAGGACCACGCCCAGCCATGCGGAGGCCCAACACACACACAGGGAGGCAAGAGAAACGAACTTGCCCAACTTTGGTCCCCCTGCCACTTCCCCCACCCACCCACCCCCGTGCACGCAAGTACTTGTGCCACCGTATGTCTGCTCCCGCTGCTGCGCCGAAACACCCGGGCAAGGTGTTCCTGGACCCGTGTGAGGTAAAGGACCACCTTGCCGAGTACCGCATCGTGGACTGCCGGTACAGCTTGAAGGTAAAGGACCACGGCAGCATTGAGTACGCGAAGGAGCACGTGAAGAGCGCCATCCGCGCCGATATGGATACGAACCTCTCCAAGTTGGTGCCCACCAGCACCGCCCGGCACCCGCTACCGCCCTGTGCTGAGTTTATCGAATGGTGCATGGCGAACGGGATGGCGGGAGAGCTGCCGGTGCTCTGCTACGATGACGAGTGCGGCGCCATGGGTGGATGCCGCCTGTGGTGGATGCTGAACTCTCTTGGCGCCGAGGCGTACGTGATCAACGGCGGCTTTCAGGCCTGCAAGGCTGCGGGGCTGGAGATGGAGTCAGGCGAGCCCCCGTCGCCGCCGACGCCCCCTACGCACTGGCCCTTCAAGACGGCCTTTCAGCATCACTATCTCGTGGATGAGATCCCGCCCAACGCGATCATCACCGACGCGCGCTCCGCCGACCGCTTCGCCTCGACAGTACGGCCTTACGCCGCAGACAAGATGCCAGGCCACATCGAAGGCGCGCGTAACCTCCCCTACACGTCGCACCTCGTGATACGCGGTGACGGCAAGGTGCTGCGCAGTGAGGAGGAGATCCGCCACAACATCATGACCGTCGTGCAGGGCACGGGTGACGCGACTGATCTGTCGAGCTTCGTCTTCTCCTGCGGCAGCGGCGTCACCGCCTGCATCAATATCGCCCTGGTGCACCACCTCGGCCTGGGCCATCCGTACCTCTACTGTGGCTCCTGGTCTGAGTATAGCGGCCTCTTCCGCCTCCCCATAATGCGCAGCATCATCGACGACTACGGCATGTGCATGCAAATGCAGACCCCTAGCCTCGGCGACAACCCGAAGGCAAACCTCGACACCATGACTCTGAAGGTCGACGGCGCGCCCTGCGAGAGACCCGATGCGGAGGTGCAGAGCGCCGCAGCCCACCTCCACGCTGGCGAGGCCGCTACTGTGTACTTCAAAAGCGGCCGCGTCGTCACGATCGAGGCGCCGGCAGTGCCCAACTAAAGCACGCGCAACAACGCACGCCTCGCCACACCGGCCCCCTGTGGGCTCATGAAAGCGCGGCGCGCCGCCTGCGTTTGGTGTCTCTTCGCCCGGGCTTGCCACCCACCCCACCCCGCCAGCAGGGTCGCCCGCGTTTCGGCCGCGCCGCCTTTATTTCCCATGGTGTTTGCTTTCGGTTGGTCCTCAGCCCCCCGCCCCCACCCCGCAAAACACAGAATAAGCCCAAGCCCACCCCCCCTGCATCTGCCTACGGCAGCGTGATCGCCACGACGCTGGCTGATCGGGCGAGGCGGCCCCACGCAACCCACCCGCCCCCTTCCCAGCCCACATGCTCATGAAAATGCGAGCGCGCTTGCGCGTCATCGCCGGCCACTCCACGCACACACTCCTCCTCCTCCCCTACACACTTCTTACACGCTACGGGGCAGAGCCGATAGTCAAGCAGGTAAGAATATTCAAAGGTCTCTCTCTAACGCCCCTCTTTCGTCTCCCTGTCTCGG

>BPK298/0 clone 8|LinJ05_V3.0970|500 bp UPS + CDS + 500 bp DWS|Mercapto-pyruvaat sulfurtransferase

AGCGCGGCGTGGGCCCAGCAGCGCCGGACCATGGGAGATAACGCGCGGCGAGGAAGGCGAAGAGGTGTGGTGCACCAGCGGTGTATTTGTGCACGCCTCTGTGTGTGCATCCGCCCGGGTGAACTCCCCTCCTCGCTTATACCCCTACAGCGACCCCACCCCCCCCCCCAGCGCACGCAAGCACCCGGCTACTCGAAAGTGCTAGCGGGCATTGCGTGCGTCTACCCGCTGATGGGGTGACTGTAAGCACAAAAAAGCACAGAGGCACTTGTAGCGCATGACCCGTCAGCCATCGCCACCATGGCGAGCCCCGAACAACACAAACACGCATGCGACAGGCAGCGAGCACTGCCACAACCACACTTCGAGGACCACGCCCAGCCATGCGGAGGCCCAACACACACACAGGGAGGCAAGAGAAACGAACTTGCCCAACTTTGGTCCCCCTGCCACTTCCCCCACCCACCCACCCCCGTGCACGCAAGTACTTGTGCCACCGTATGTCTGCTCCCGCTGCTGCGCCGAAACACCCGGGCAAGGTGTTCCTGGACCCGTGTGAGGTAAAGGACCACCTTGCCGAGTACCGCATCGTGGACTGCCGGTACAGCTTGAAGGTAAAGGACCACGGCAGCATTGAGTACGCGAAGGAGCACGTGAAGAGCGCCATCCGCGCCGATATGGATACGAACCTCTCCAAGTTGGTGCCCACCAGCACCGCCCGGCACCCGCTACCGCCCTGTGCTGAGTTTATCGAATGGTGCATGGCGAACGGGATGGCGGGAGAGCTGCCGGTGCTCTGCTACGATGACGAGTGCGGCGCCATGGGTGGATGCCGCCTGTGGTGGATGCTGAACTCTCTTGGCGCCGAGGCGTACGTGATCAACGGCGGCTTTCAGGCCTGCAAGGCTGCGGGGCTGGAGATGGAGTCAGGCGAGCCCCCGTCGCCGCCGACGCCCCCTACGCACTGGCCCTTCAAGACGGCCTTTCAGCATCACTATCTCGTGGATGAGATCCCGCCCAACGCGATCATCACCGACGCGCGCTCCGCCGACCGCTTCGCCTCGACAGTACGGCCTTACGCCGCAGACAAGATGCCAGGCCACATCGAAGGCGCGCGTAACCTCCCCTACACGTCGCACCTCGTGATACGCGGTGACGGCAAGGTGCTGCGCAGTGAGGAGGAGATCCGCCACAACATCATGACCGTCGTGCAGGGCACGGGTGACGCGACTGATCTGTCGAGCTTCGTCTTCTCCTGCGGCAGCGGCGTCACCGCCTGCATCAATATCGCCCTGGTGCACCACCTCGGCCTGGGCCATCCGTACCTCTACTGTGGCTCCTGGTCTGAGTATAGCGGCCTCTTCCGCCTCCCCATAATGCGCAGCATCATCGACGACTACGGCATGTGCATGCAAATGCAGACCCCTAGCCTCGGCGACAACCCGAAGGCAAACCTCGACACCATGACTCTGAAGGTCGACGGCGCGCCCTGCGAGAGACCCGATGCGGAGGTGCAGAGCGCCGCAGCCCACCTCCACGCTGGCGAGGCCGCTACTGTGTACTTCAAAAGCGGCCGCGTCGTCACGATCGAGGCGCCGGCAGTGCCCAACTAAAGCACGCGCAACAACGCACGCCTCGCCACACCGGCCCCCTGTGGGCTCATGAAAGCGCGGCGCGCCGCCTGCGTTTGGTGTCTCTTCGCCCGGGCTTGCCACCCACCCCACCCCGCCAGCAGGGTCGCCCGCGTTTCGGCCGCGCCGCCTTTATTTCCCATGGTGTTTGCTTTCGGTTGGTCCTCAGCCCCCCGCCCCCACCCCGCAAAACACAGAATAAGCCCAAGCCCACCCCCCCTGCATCTGCCTACGGCAGCGTGATCGCCACGACGCTGGCTGATCGGGCGAGGCGGCCCCACGCAACCCACCCGCCCCCTTCCCAGCCCACATGCTCATGAAAATGCGAGCGCGCTTGCGCGTCATCGCCGGCCACTCCACGCACACACTCCTCCTCCTCCCCTACACACTTCTTACACGCTACGGGGCAGAGCCGATAGTCAAGCAGGTAAGAATATTCAAAGGTCTCTCTCTAACGCCCCTCTTTCGTCTCCCTGTCTCGG

### 4. TRYPANOTHIONE REDCUTASE

>BPK035/0 clone 1|LinJ05_V3.0350|500 bp UPS + CDS + 500 bp DWS|trypanothione reductase (TR)

ACGCACACCCGCTGGCGAAGAAAAGAGTGTCGTCATCTATCTCTCTCTTTACACTCCTACACACACACACACACACNNNNNNNNNNNNNNNNNNNNNNNNNNNNNNNNNNNNNNNNNNNNNNNNNNNNNNNNNNNNNNNNNNNNNNNNNNNNNNNNNNNNNNNNNNNNNNNNNNNGTGAGTCTTGATCCATCATTGCGCCTTCTACTTCGCTATACACAAACGCGCTCAGACGTTCACGGACACCACCGCCACCGCAGCCGCAGCAGCCGAAAGCCGCACGTAGGAATGCAGGCGAGCCTTACCACGAAGATTGTCTGATCAGCCCGTCTTTATCTCTCGCTCCCATCGTTGTATATACATTGCAGCGAGTCTTGCTGCTGGCGTTCTCTGCTCCGTGTGTGCCCACTCGCGCGCCGTTGGAGTTGTTCGCCGCGAACTCGCGCAGCCTCCCCCTCCCCTCCCCCTCTCGCGAAAATTCTTCGCTTCTCTCAACGGCTGAATGTCCCGCGCGTACGACCTCGTGGTGCTTGGCGCCGGATCTGGAGGTCTGGAGGCGGGATGGAACGCGGCCGTCACGCACAAGAAGAAGGTGGCCGTCGTCGATGTGCAGGCGACGCACGGTCCGCCGCTCTTCGCTGCGCTCGGCGGCACGTGCGTGAACGTCGGCTGCGTGCCAAAGAAACTCATGGTGACAGGTGCCCAGTACATGGACCTGATCCGTGAGTCTGGCGGCTTCGGATGGGAGATGGACCGCGAATCGCTCTGCCCCAACTGGAAGACGCTCATCGCCGCGAAGAACAAGGTGGTGAACAGCATCAACGAGAGCTACAAGAGCATGTTCGCTGATACGGAGGGCCTCAGCTTTCACATGGGCTTCGGTGCCCTTCAAGACGCTCACACGGTGGTGGTGCGCAAGTCGGAAGACCCACACAGCGACGTGCTGGAGACCCTCGACACGGAGTACATCCTCATTGCCACCGGCTCCTGGCCGACGCGCCTCGGAGTCCCCGGCGACGAGTTCTGCATCACGAGCAACGAGGCCTTCTACCTCGAGGATGCCCCCAAGCGGATGCTGTGCGTCGGCGGCGGCTACATCGCCGTTGAGTTTGCCGGCATCTTCAACGGCTACAAGCCCCAGGGTGGCTATGTCGACCTGTGCTACCGCGGCGATCTTATTTTGCGCGGCTTCGATACAGAGGTGCGCAAGAGCCTGACGAAGCAGCTGGGGGCGAACGGAATAAGAGTGCGTACAAACTTGAACCCGACGAAGATCACGAAGAATGAGGACGGCTCGAATCACGTTCACTTCAACGATGGCACGGAGGAGGACTACGATCAGGTCATGCTCGCGATCGGTCGCGTGCCGCGCTCGCAGGCACTACAGCTCGGCAAGGCCGGCGTCCGAACAGGAAAGAACGGTGCCGTGCAGGTCGACGCGTATTCGAAGACATCGGTGGACAACATCTACGCCATCGGCGACGTGACGAACCGCGTGATGTTGACGCCGGTGGCCATCAACGAAGGCGCCGCCTTCGTTGAAACCGTCTTCGGTGGCAAGCCCCGCGCCACCGACCACACGAAGGTCGCGTGCGCCGTGTTCTCCATACCGCCGATCGGCACGTGCGGCATGACGGAGGAGGAGGCGGCGAAGAACTACGAAACCGTCGCCGTGTACGCGAGCTCCTTCACGCCCCTTATGCACAACATCAGCGGCAGCAAGCACAAGGAATTCATGATCCGCATCATCACGAACGAATCCAACGGCGAGGTTCTGGGTGTTCACATGCTCGGCGACAGTGCGCCTGAGATCATCCAGAGCGTCGGCATTTGCATGAAGATGGGCGCCAAGATCAGCGACTTCCACAGCACCATCGGAGTCCACCCGACGAGCGCCGAGGAGCTCTGCTCCATGCGCACTCCAGCGTACTTCTACGAGAGTGGCAAGCGCGTCGAAAAGCTCAGCAGCAACCTCTGAGAGGGAGGAGAGATGAAGAAGAACGCCTCAGCCCTTCCACCTAGCCGTGATGCACGGGTATCACAGACCAGCAGGCATGCAGAGATGGAAAGGGGAAGAGGTCAGCAGGCAGTGACGTCTGATCTCCTTCAGCGTCGGTCGCTTCGGCCGCTTCCTCTCCGACCTCCCCCCCCACCACCACTGCCACCACTCCTCCTCTCCGCATAACCTATCAGCAGTCGCGCACGCCTCATCAATACGCACGCACACCCGCCGGTGGCGGGGCGCAGGATGGAGAAGGGCAGCGGATGGAGCTTCCCTTCTCCCACGCCCTTTTCACCCCACCACGCCGTGCGGGTTTTGTGGTTTGTGCGTGTGTTTGTGCTTTCTAGTGGGGAGAAAGGCCTCTCCGCATCGTTCAACTATTTCGCTCTGCGTGTGAGCACGCGTGCGCACTTGCGCGTTCGCGTGCTCGTTCCTTTCTTGCCTTCTTTCGCCCTCCGCGTGCGTGCGTGCGTGCT

>BPK043/0 clone 2|LinJ05_V3.0350|500 bp UPS + CDS + 500 bp DWS|trypanothione reductase (TR)

ACGCACACCCGCTGGCGAAGAAAAGAGTGTCGTCATCTATCTCTCTCTTTACACTCCTACACACACACACACACACNNNNNNNNNNNNNNNNNNNNNNNNNNNNNNNNNNNNNNNNNNNNNNNNNNNNNNNNNNNNNNNNNNNNNNNNNNNNNNNNNNNNNNNNNNNNNNNNNNNGTGAGTCTTGATCCATCATTGCGCCTTCTACTTCGCTATACACAAACGCGCTCAGACGTTCACGGACACCACCGCCACCGCAGCCGCAGCAGCCGAAAGCCGCACGTAGGAATGCAGGCGAGCCTTACCACGAAGATTGTCTGATCAGCCCGTCTTTATCTCTCGCTCCCATCGTTGTATATACATTGCAGCGAGTCTTGCTGCTGGCGTTCTCTGCTCCGTGTGTGCCCACTCGCGCGCCGTTGGAGTTGTTCGCCGCGAACTCGCGCAGCCTCCCCCTCCCCTCCCCCTCTCGCGAAAATTCTTCGCTTCTCTCAACGGCTGAATGTCCCGCGCGTACGACCTCGTGGTGCTTGGCGCCGGATCTGGAGGTCTGGAGGCGGGATGGAACGCGGCCGTCACGCACAAGAAGAAGGTGGCCGTCGTCGATGTGCAGGCGACGCACGGTCCGCCGCTCTTCGCTGCGCTCGGCGGCACGTGCGTGAACGTCGGCTGCGTGCCAAAGAAACTCATGGTGACAGGTGCCCAGTACATGGACCTGATCCGTGAGTCTGGCGGCTTCGGATGGGAGATGGACCGCGAATCGCTCTGCCCCAACTGGAAGACGCTCATCGCCGCGAAGAACAAGGTGGTGAACAGCATCAACGAGAGCTACAAGAGCATGTTCGCTGATACGGAGGGCCTCAGCTTTCACATGGGCTTCGGTGCCCTTCAAGACGCTCACACGGTGGTGGTGCGCAAGTCGGAAGACCCACACAGCGACGTGCTGGAGACCCTCGACACGGAGTACATCCTCATTGCCACCGGCTCCTGGCCGACGCGCCTCGGAGTCCCCGGCGACGAGTTCTGCATCACGAGCAACGAGGCCTTCTACCTCGAGGATGCCCCCAAGCGGATGCTGTGCGTCGGCGGCGGCTACATCGCCGTTGAGTTTGCCGGCATCTTCAACGGCTACAAGCCCCAGGGTGGCTATGTCGACCTGTGCTACCGCGGCGATCTTATTTTGCGCGGCTTCGATACAGAGGTGCGCAAGAGCCTGACGAAGCAGCTGGGGGCGAACGGAATAAGAGTGCGTACAAACTTGAACCCGACGAAGATCACGAAGAATGAGGACGGCTCGAATCACGTTCACTTCAACGATGGCACGGAGGAGGACTACGATCAGGTCATGCTCGCGATCGGTCGCGTGCCGCGCTCGCAGGCACTACAGCTCGGCAAGGCCGGCGTCCGAACAGGAAAGAACGGTGCCGTGCAGGTCGACGCGTATTCGAAGACATCGGTGGACAACATCTACGCCATCGGCGACGTGACGAACCGCGTGATGTTGACGCCGGTGGCCATCAACGAAGGCGCCGCCTTCGTTGAAACCGTCTTCGGTGGCAAGCCCCGCGCCACCGACCACACGAAGGTCGCGTGCGCCGTGTTCTCCATACCGCCGATCGGCACGTGCGGCATGACGGAGGAGGAGGCGGCGAAGAACTACGAAACCGTCGCCGTGTACGCGAGCTCCTTCACGCCCCTTATGCACAACATCAGCGGCAGCAAGCACAAGGAATTCATGATCCGCATCATCACGAACGAATCCAACGGCGAGGTTCTGGGTGTTCACATGCTCGGCGACAGTGCGCCTGAGATCATCCAGAGCGTCGGCATTTGCATGAAGATGGGCGCCAAGATCAGCGACTTCCACAGCACCATCGGAGTCCACCCGACGAGCGCCGAGGAGCTCTGCTCCATGCGCACTCCAGCGTACTTCTACGAGAGTGGCAAGCGCGTCGAAAAGCTCAGCAGCAACCTCTGAGAGGGAGGAGAGATGAAGAAGAACGCCTCAGCCCTTCCACCTAGCCGTGATGCACGGGTATCACAGACCAGCAGGCATGCAGAGATGGAAAGGGGAAGAGGTCAGCAGGCAGTGACGTCTGATCTCCTTCAGCGTCGGTCGCTTCGGCCGCTTCCTCTCCGACCTCCCCCCCCACCACCACTGCCACCACTCCTCCTCTCCGCATAACCTATCAGCAGTCGCGCACGCCTCATCAATACGCACGCACACCCGCCGGTGGCGGGGCGCAGGATGGAGAAGGGCAGCGGATGGAGCTTCCCTTCTCCCACGCCCTTTTCACCCCACCACGCCGTGCGGGTTTTGTGGTTTGTGCGTGTGTTTGTGCTTTCTAGTGGGGAGAAAGGCCTCTCCGCATCGTTCAACTATTTCGCTCTGCGTGTGAGCACGCGTGCGCACTTGCGCGTTCGCGTGCTCGTTCCTTTCTTGCCTTCTTTCGCCCTCCGCGTGCGTGCGTGCGTGCT

>BPK085/0 clone 8|LinJ05_V3.0350|500 bp UPS + CDS + 500 bp DWS|trypanothione reductase (TR)

ACGCACACCCGCTGGCGAAGAAAAGAGTGTCGTCATCTATCTCTCTCTTTACACTCCTACACACACACACACACACNNNNNNNNNNNNNNNNNNNNNNNNNNNNNNNNNNNNNNNNNNNNNNNNNNNNNNNNNNNNNNNNNNNNNNNNNNNNNNNNNNNNNNNNNNNNNNNNNNNGTGAGTCTTGATCCATCATTGCGCCTTCTACTTCGCTATACACAAACGCGCTCAGACGTTCACGGACACCACCGCCACCGCAGCCGCAGCAGCCGAAAGCCGCACGTAGGAATGCAGGCGAGCCTTACCACGAAGATTGTCTGATCAGCCCGTCTTTATCTCTCGCTCCCATCGTTGTATATACATTGCAGCGAGTCTTGCTGCTGGCGTTCTCTGCTCCGTGTGTGCCCACTCGCGCGCCGTTGGAGTTGTTCGCCGCGAACTCGCGCAGCCTCCCCCTCCCCTCCCCCTCTCGCGAAAATTCTTCGCTTCTCTCAACGGCTGAATGTCCCGCGCGTACGACCTCGTGGTGCTTGGCGCCGGATCTGGAGGTCTGGAGGCGGGATGGAACGCGGCCGTCACGCACAAGAAGAAGGTGGCCGTCGTCGATGTGCAGGCGACGCACGGTCCGCCGCTCTTCGCTGCGCTCGGCGGCACGTGCGTGAACGTCGGCTGCGTGCCAAAGAAACTCATGGTGACAGGTGCCCAGTACATGGACCTGATCCGTGAGTCTGGCGGCTTCGGATGGGAGATGGACCGCGAATCGCTCTGCCCCAACTGGAAGACGCTCATCGCCGCGAAGAACAAGGTGGTGAACAGCATCAACGAGAGCTACAAGAGCATGTTCGCTGATACGGAGGGCCTCAGCTTTCACATGGGCTTCGGTGCCCTTCAAGACGCTCACACGGTGGTGGTGCGCAAGTCGGAAGACCCACACAGCGACGTGCTGGAGACCCTCGACACGGAGTACATCCTCATTGCCACCGGCTCCTGGCCGACGCGCCTCGGAGTCCCCGGCGACGAGTTCTGCATCACGAGCAACGAGGCCTTCTACCTCGAGGATGCCCCCAAGCGGATGCTGTGCGTCGGCGGCGGCTACATCGCCGTTGAGTTTGCCGGCATCTTCAACGGCTACAAGCCCCAGGGTGGCTATGTCGACCTGTGCTACCGCGGCGATCTTATTTTGCGCGGCTTCGATACAGAGGTGCGCAAGAGCCTGACGAAGCAGCTGGGGGCGAACGGAATAAGAGTGCGTACAAACTTGAACCCGACGAAGATCACGAAGAATGAGGACGGCTCGAATCACGTTCACTTCAACGATGGCACGGAGGAGGACTACGATCAGGTCATGCTCGCGATCGGTCGCGTGCCGCGCTCGCAGGCACTACAGCTCGGCAAGGCCGGCGTCCGAACAGGAAAGAACGGTGCCGTGCAGGTCGACGCGTATTCGAAGACATCGGTGGACAACATCTACGCCATCGGCGACGTGACGAACCGCGTGATGTTGACGCCGGTGGCCATCAACGAAGGCGCCGCCTTCGTTGAAACCGTCTTCGGTGGCAAGCCCCGCGCCACCGACCACACGAAGGTCGCGTGCGCCGTGTTCTCCATACCGCCGATCGGCACGTGCGGCATGACGGAGGAGGAGGCGGCGAAGAACTACGAAACCGTCGCCGTGTACGCGAGCTCCTTCACGCCCCTTATGCACAACATCAGCGGCAGCAAGCACAAGGAATTCATGATCCGCATCATCACGAACGAATCCAACGGCGAGGTTCTGGGTGTTCACATGCTCGGCGACAGTGCGCCTGAGATCATCCAGAGCGTCGGCATTTGCATGAAGATGGGCGCCAAGATCAGCGACTTCCACAGCACCATCGGAGTCCACCCGACGAGCGCCGAGGAGCTCTGCTCCATGCGCACTCCAGCGTACTTCTACGAGAGTGGCAAGCGCGTCGAAAAGCTCAGCAGCAACCTCTGAGAGGGAGGAGAGATGAAGAAGAACGCCTCAGCCCTTCCACCTAGCCGTGATGCACGGGTATCACAGACCAGCAGGCATGCAGAGATGGAAAGGGGAAGAGGTCAGCAGGCAGTGACGTCTGATCTCCTTCAGCGTCGGTCGCTTCGGCCGCTTCCTCTCCGACCYCCCCCCCCMCCACCACTGCCACCACTCCTCCTCTCCGCATAACCTATCAGCAGTCGCGCACGCCTCATCAATACGCACGCACACCCGCCGGTGGCGGGGCGCAGGATGGAGAAGGGCAGCGGATGGAGCTTCCCTTCTCCCACGCCCTTTTCACCCCACCACGCCGTGCGGGTTTTGTGGTTTGTGCGTGTGTTTGTGCTTTCTAGTGGGGAGAAAGGCCTCTCCGCATCGTTCAACTATTTCGCTCTGCGTGTGAGCACGCGTGCGCACTTGCGCGTTCGCGTGCTCGTTCCTTTCTTGCYTTCTTTCGCCCTCCGCGTGCGTGCGTGCGTGCT

>BPK087/0 clone 11|LinJ05_V3.0350|500 bp UPS + CDS + 500 bp DWS|trypanothione reductase (TR)

ACGCACACCCGCTGGCGAAGAAAAGAGTGTCGTCATCTATCTCTCTCTTTACACTCCTACACACACACACACACACNNNNNNNNNNNNNNNNNNNNNNNNNNNNNNNNNNNNNNNNNNNNNNNNNNNNNNNNNNNNNNNNNNNNNNNNNNNNNNNNNNNNNNNNNNNNNNNNNNNGTGAGTCTTGATCCATCATTGCGCCTTCTACTTCGCTATACACAAACGCGCTCAGACGTTCACGGACACCACCGCCACCGCAGCCGCAGCAGCCGAAAGCCGCACGTAGGAATGCAGGCGAGCCTTACCACGAAGATTGTCTGATCAGCCCGTCTTTATCTCTCGCTCCCATCGTTGTATATACATTGCAGCGAGTCTTGCTGCTGGCGTTCTCTGCTCCGTGTGTGCCCACTCGCGCGCCGTTGGAGTTGTTCGCCGCGAACTCGCGCAGCCTCCCCCTCCCCTCCCCCTCTCGCGAAAATTCTTCGCTTCTCTCAACGGCTGAATGTCCCGCGCGTACGACCTCGTGGTGCTTGGCGCCGGATCTGGAGGTCTGGAGGCGGGATGGAACGCGGCCGTCACGCACAAGAAGAAGGTGGCCGTCGTCGATGTGCAGGCGACGCACGGTCCGCCGCTCTTCGCTGCGCTCGGCGGCACGTGCGTGAACGTCGGCTGCGTGCCAAAGAAACTCATGGTGACAGGTGCCCAGTACATGGACCTGATCCGTGAGTCTGGCGGCTTCGGATGGGAGATGGACCGCGAATCGCTCTGCCCCAACTGGAAGACGCTCATCGCCGCGAAGAACAAGGTGGTGAACAGCATCAACGAGAGCTACAAGAGCATGTTCGCTGATACGGAGGGCCTCAGCTTTCACATGGGCTTCGGTGCCCTTCAAGACGCTCACACGGTGGTGGTGCGCAAGTCGGAAGACCCACACAGCGACGTGCTGGAGACCCTCGACACGGAGTACATCCTCATTGCCACCGGCTCCTGGCCGACGCGCCTCGGAGTCCCCGGCGACGAGTTCTGCATCACGAGCAACGAGGCCTTCTACCTCGAGGATGCCCCCAAGCGGATGCTGTGCGTCGGCGGCGGCTACATCGCCGTTGAGTTTGCCGGCATCTTCAACGGCTACAAGCCCCAGGGTGGCTATGTCGACCTGTGCTACCGCGGCGATCTTATTTTGCGCGGCTTCGATACAGAGGTGCGCAAGAGCCTGACGAAGCAGCTGGGGGCGAACGGAATAAGAGTGCGTACAAACTTGAACCCGACGAAGATCACGAAGAATGAGGACGGCTCGAATCACGTTCACTTCAACGATGGCACGGAGGAGGACTACGATCAGGTCATGCTCGCGATCGGTCGCGTGCCGCGCTCGCAGGCACTACAGCTCGGCAAGGCCGGCGTCCGAACAGGAAAGAACGGTGCCGTGCAGGTCGACGCGTATTCGAAGACATCGGTGGACAACATCTACGCCATCGGCGACGTGACGAACCGCGTGATGTTGACGCCGGTGGCCATCAACGAAGGCGCCGCCTTCGTTGAAACCGTCTTCGGTGGCAAGCCCCGCGCCACCGACCACACGAAGGTCGCGTGCGCCGTGTTCTCCATACCGCCGATCGGCACGTGCGGCATGACGGAGGAGGAGGCGGCGAAGAACTACGAAACCGTCGCCGTGTACGCGAGCTCCTTCACGCCCCTTATGCACAACATCAGCGGCAGCAAGCACAAGGAATTCATGATCCGCATCATCACGAACGAATCCAACGGCGAGGTTCTGGGTGTTCACATGCTCGGCGACAGTGCGCCTGAGATCATCCAGAGCGTCGGCATTTGCATGAAGATGGGCGCCAAGATCAGCGACTTCCACAGCACCATCGGAGTCCACCCGACGAGCGCCGAGGAGCTCTGCTCCATGCGCACTCCAGCGTACTTCTACGAGAGTGGCAAGCGCGTCGAAAAGCTCAGCAGCAACCTCTGAGAGGGAGGAGAGATGAAGAAGAACGCCTCAGCCCTTCCACCTAGCCGTGATGCACGGGTATCACAGACCAGCAGGCATGCAGAGATGGAAAGGGGAAGAGGTCAGCAGGCAGTGACGTCTGATCTCCTTCAGCGTCGGTCGCTTCGGCCGCTTCCTCTCCGACCTCCCCCCCCACCACCACTGCCACCACTCCTCCTCTCCGCATAACCTATCAGCAGTCGCGCACGCCTCATCAATACGCACGCACACCCGCCGGTGGCGGGGCGCAGGATGGAGAAGGGCAGCGGATGGAGCTTCCCTTCTCCCACGCCCTTTTCACCCCACCACGCCGTGCGGGTTTTGTGGTTTGTGCGTGTGTTTGTGCTTTCTAGTGGGGAGAAAGGCCTCTCCGCATCGTTCAACTATTTCGCTCTGCGTGTGAGCACGCGTGCGCACTTGCGCGTTCGCGTGCTCGTTCCTTTCTTGCCTTCTTTCGCCCTCCGCGTGCGTGCGTGCGTGCT

>BPK178/0 clone 3|LinJ05_V3.0350|500 bp UPS + CDS + 500 bp DWS|trypanothione reductase (TR)

ACGCACACCCGCTGGCGAAGAAAAGAGTGTCGTCATCTATCTCTCTCTTTACACTCCTACACACACACACACACACNNNNNNNNNNNNNNNNNNNNNNNNNNNNNNNNNNNNNNNNNNNNNNNNNNNNNNNNNNNNNNNNNNNNNNNNNNNNNNNNNNNNNNNNNNNNNNNNNNNGTGAGTCTTGATCCATCATTGCGCCTTCTACTTCGCTATACACAAACGCGCTCAGACGTTCACGGACACCACCGCCACCGCAGCCGCAGCAGCCGAAAGCCGCACGTAGGAATGCAGGCGAGCCTTACCACGAAGATTGTCTGATCAGCCCGTCTTTATCTCTCGCTCCCATCGTTGTATATACATTGCAGCGAGTCTTGCTGCTGGCGTTCTCTGCTCCGTGTGTGCCCACTCGCGCGCCGTTGGAGTTGTTCGCCGCGAACTCGCGCAGCCTCCCCCTCCCCTCCCCCTCTCGCGAAAATTCTTCGCTTCTCTCAACGGCTGAATGTCCCGCGCGTACGACCTCGTGGTGCTTGGCGCCGGATCTGGAGGTCTGGAGGCGGGATGGAACGCGGCCGTCACGCACAAGAAGAAGGTGGCCGTCGTCGATGTGCAGGCGACGCACGGTCCGCCGCTCTTCGCTGCGCTCGGCGGCACGTGCGTGAACGTCGGCTGCGTGCCAAAGAAACTCATGGTGACAGGTGCCCAGTACATGGACCTGATCCGTGAGTCTGGCGGCTTCGGATGGGAGATGGACCGCGAATCGCTCTGCCCCAACTGGAAGACGCTCATCGCCGCGAAGAACAAGGTGGTGAACAGCATCAACGAGAGCTACAAGAGCATGTTCGCTGATACGGAGGGCCTCAGCTTTCACATGGGCTTCGGTGCCCTTCAAGACGCTCACACGGTGGTGGTGCGCAAGTCGGAAGACCCACACAGCGACGTGCTGGAGACCCTCGACACGGAGTACATCCTCATTGCCACCGGCTCCTGGCCGACGCGCCTCGGAGTCCCCGGCGACGAGTTCTGCATCACGAGCAACGAGGCCTTCTACCTCGAGGATGCCCCCAAGCGGATGCTGTGCGTCGGCGGCGGCTACATCGCCGTTGAGTTTGCCGGCATCTTCAACGGCTACAAGCCCCAGGGTGGCTATGTCGACCTGTGCTACCGCGGCGATCTTATTTTGCGCGGCTTCGATACAGAGGTGCGCAAGAGCCTGACGAAGCAGCTGGGGGCGAACGGAATAAGAGTGCGTACAAACTTGAACCCGACGAAGATCACGAAGAATGAGGACGGCTCGAATCACGTTCACTTCAACGATGGCACGGAGGAGGACTACGATCAGGTCATGCTCGCGATCGGTCGCGTGCCGCGCTCGCAGGCACTACAGCTCGGCAAGGCCGGCGTCCGAACAGGAAAGAACGGTGCCGTGCAGGTCGACGCGTATTCGAAGACATCGGTGGACAACATCTACGCCATCGGCGACGTGACGAACCGCGTGATGTTGACGCCGGTGGCCATCAACGAAGGCGCCGCCTTCGTTGAAACCGTCTTCGGTGGCAAGCCCCGCGCCACCGACCACACGAAGGTCGCGTGCGCCGTGTTCTCCATACCGCCGATCGGCACGTGCGGCATGACGGAGGAGGAGGCGGCGAAGAACTACGAAACCGTCGCCGTGTACGCGAGCTCCTTCACGCCCCTTATGCACAACATCAGCGGCAGCAAGCACAAGGAATTCATGATCCGCATCATCACGAACGAATCCAACGGCGAGGTTCTGGGTGTTCACATGCTCGGCGACAGTGCGCCTGAGATCATCCAGAGCGTCGGCATTTGCATGAAGATGGGCGCCAAGATCAGCGACTTCCACAGCACCATCGGAGTCCACCCGACGAGCGCCGAGGAGCTCTGCTCCATGCGCACTCCAGCGTACTTCTACGAGAGTGGCAAGCGCGTCGAAAAGCTCAGCAGCAACCTCTGAGAGGGAGGAGAGATGAAGAAGAACGCCTCAGCCCTTCCACCTAGCCGTGATGCACGGGTATCACAGACCAGCAGGCATGCAGAGATGGAAAGGGGAAGAGGTCAGCAGGCAGTGACGTCTGATCTCCTTCAGCGTCGGTCGCTTCGGCCGCTTCCTCTCCGACCTCCCCCCCCMCCACCACTGCCACCACTCCTCCTCTCCGCATAACCTATCAGCAGTCGCGCACGCCTCATCAATACGCACGCACACCCGCCGGTGGCGGGGCGCAGGATGGAGAAGGGCAGCGGATGGAGCTTCCCTTCTCCCACGCCCTTTTCACCCCACCACGCCGTGCGGGTTTTGTGGTTTGTGCGTGTGTTTGTGCTTTCTAGTGGGGAGAAAGGCCTCTCCGCATCGTTCAACTATTTCGCTCTGCGTGTGAGCACGCGTGCGCACTTGCGCGTTCGCGTGCTCGTTCCTTTCTTGCCTTCTTTCGCCCTCCGCGTGCGTGCGTGCGTGCT

>BPK190/0 clone 3|LinJ05_V3.0350|500 bp UPS + CDS + 500 bp DWS|trypanothione reductase (TR)

ACGCACACCCGCTGGCGAAGAAAAGAGTGTCGTCATCTATCTCTCTCTTTACACTCCTACACACACACACACACACNNNNNNNNNNNNNNNNNNNNNNNNNNNNNNNNNNNNNNNNNNNNNNNNNNNNNNNNNNNNNNNNNNNNNNNNNNNNNNNNNNNNNNNNNNNNNNNNNNNGTGAGTCTTGATCCATCATTGCGCCTTCTACTTCGCTATACACAAACGCGCTCAGACGTTCACGGACACCACCGCCACCGCAGCCGCAGCAGCCGAAAGCCGCACGTAGGAATGCAGGCGAGCCTTACCACGAAGATTGTCTGATCAGCCCGTCTTTATCTCTCGCTCCCATCGTTGTATATACATTGCAGCGAGTCTTGCTGCTGGCGTTCTCTGCTCCGTGTGTGCCCACTCGCGCGCCGTTGGAGTTGTTCGCCGCGAACTCGCGCAGCCTCCCCCTCCCCTCCCCCTCTCGCGAAAATTCTTCGCTTCTCTCAACGGCTGAATGTCCCGCGCGTACGACCTCGTGGTGCTTGGCGCCGGATCTGGAGGTCTGGAGGCGGGATGGAACGCGGCCGTCACGCACAAGAAGAAGGTGGCCGTCGTCGATGTGCAGGCGACGCACGGTCCGCCGCTCTTCGCTGCGCTCGGCGGCACGTGCGTGAACGTCGGCTGCGTGCCAAAGAAACTCATGGTGACAGGTGCCCAGTACATGGACCTGATCCGTGAGTCTGGCGGCTTCGGATGGGAGATGGACCGCGAATCGCTCTGCCCCAACTGGAAGACGCTCATCGCCGCGAAGAACAAGGTGGTGAACAGCATCAACGAGAGCTACAAGAGCATGTTCGCTGATACGGAGGGCCTCAGCTTTCACATGGGCTTCGGTGCCCTTCAAGACGCTCACACGGTGGTGGTGCGCAAGTCGGAAGACCCACACAGCGACGTGCTGGAGACCCTCGACACGGAGTACATCCTCATTGCCACCGGCTCCTGGCCGACGCGCCTCGGAGTCCCCGGCGACGAGTTCTGCATCACGAGCAACGAGGCCTTCTACCTCGAGGATGCCCCCAAGCGGATGCTGTGCGTCGGCGGCGGCTACATCGCCGTTGAGTTTGCCGGCATCTTCAACGGCTACAAGCCCCAGGGTGGCTATGTCGACCTGTGCTACCGCGGCGATCTTATTTTGCGCGGCTTCGATACAGAGGTGCGCAAGAGCCTGACGAAGCAGCTGGGGGCGAACGGAATAAGAGTGCGTACAAACTTGAACCCGACGAAGATCACGAAGAATGAGGACGGCTCGAATCACGTTCACTTCAACGATGGCACGGAGGAGGACTACGATCAGGTCATGCTCGCGATCGGTCGCGTGCCGCGCTCGCAGGCACTACAGCTCGGCAAGGCCGGCGTCCGAACAGGAAAGAACGGTGCCGTGCAGGTCGACGCGTATTCGAAGACATCGGTGGACAACATCTACGCCATCGGCGACGTGACGAACCGCGTGATGTTGACGCCGGTGGCCATCAACGAAGGCGCCGCCTTCGTTGAAACCGTCTTCGGTGGCAAGCCCCGCGCCACCGACCACACGAAGGTCGCGTGCGCCGTGTTCTCCATACCGCCGATCGGCACGTGCGGCATGACGGAGGAGGAGGCGGCGAAGAACTACGAAACCGTCGCCGTGTACGCGAGCTCCTTCACGCCCCTTATGCACAACATCAGCGGCAGCAAGCACAAGGAATTCATGATCCGCATCATCACGAACGAATCCAACGGCGAGGTTCTGGGTGTTCACATGCTCGGCGACAGTGCGCCTGAGATCATCCAGAGCGTCGGCATTTGCATGAAGATGGGCGCCAAGATCAGCGACTTCCACAGCACCATCGGAGTCCACCCGACGAGCGCCGAGGAGCTCTGCTCCATGCGCACTCCAGCGTACTTCTACGAGAGTGGCAAGCGCGTCGAAAAGCTCAGCAGCAACCTCTGAGAGGGAGGAGAGATGAAGAAGAACGCCTCAGCCCTTCCACCTAGCCGTGATGCACGGGTATCACAGACCAGCAGGCATGCAGAGATGGAAAGGGGAAGAGGTCAGCAGGCAGTGACGTCTGATCTCCTTCAGCGTCGGTCGCTTCGGCCGCTTCCTCTCCGACCTCCCCCCCCMCCACCACTGCCACCACTCCTCCTCTCCGCATAACCTATCAGCAGTCGCGCACGCCTCATCAATACGCACGCACACCCGCCGGTGGCGGGGCGCAGGATGGAGAAGGGCAGCGGATGGAGCTTCCCTTCTCCCACGCCCTTTTCACCCCACCACGCCGTGCGGGTTTTGTGGTTTGTGCGTGTGTTTGTGCTTTCTAGTGGGGAGAAAGGCCTCTCCGCATCGTTCAACTATTTCGCTCTGCGTGTGAGCACGCGTGCGCACTTGCGCGTTCGCGTGCTCGTTCCTTTCTTGCCTTCTTTCGCCCTCCGCGTGCGTGCGTGCGTGCT

>BPK206/0 clone 10|LinJ05_V3.0350|500 bp UPS + CDS + 500 bp DWS|trypanothione reductase (TR)

ACGCACACCCGCTGGCGAAGAAAAGAGTGTCGTCATCTATCTCTCTCTTTACACTCCTACACACACACACACACACNNNNNNNNNNNNNNNNNNNNNNNNNNNNNNNNNNNNNNNNNNNNNNNNNNNNNNNNNNNNNNNNNNNNNNNNNNNNNNNNNNNNNNNNNNNNNNNNNNNGTGAGTCTTGATCCATCATTGCGCCTTCTACTTCGCTATACACAAACGCGCTCAGACGTTCACGGACACCACCGCCACCGCAGCCGCAGCAGCCGAAAGCCGCACGTAGGAATGCAGGCGAGCCTTACCACGAAGATTGTCTGATCAGCCCGTCTTTATCTCTCGCTCCCATCGTTGTATATACATTGCAGCGAGTCTTGCTGCTGGCGTTCTCTGCTCCGTGTGTGCCCACTCGCGCGCCGTTGGAGTTGTTCGCCGCGAACTCGCGCAGCCTCCCCCTCCCCTCCCCCTCTCGCGAAAATTCTTCGCTTCTCTCAACGGCTGAATGTCCCGCGCGTACGACCTCGTGGTGCTTGGCGCCGGATCTGGAGGTCTGGAGGCGGGATGGAACGCGGCCGTCACGCACAAGAAGAAGGTGGCCGTCGTCGATGTGCAGGCGACGCACGGTCCGCCGCTCTTCGCTGCGCTCGGCGGCACGTGCGTGAACGTCGGCTGCGTGCCAAAGAAACTCATGGTGACAGGTGCCCAGTACATGGACCTGATCCGTGAGTCTGGCGGCTTCGGATGGGAGATGGACCGCGAATCGCTCTGCCCCAACTGGAAGACGCTCATCGCCGCGAAGAACAAGGTGGTGAACAGCATCAACGAGAGCTACAAGAGCATGTTCGCTGATACGGAGGGCCTCAGCTTTCACATGGGCTTCGGTGCCCTTCAAGACGCTCACACGGTGGTGGTGCGCAAGTCGGAAGACCCACACAGCGACGTGCTGGAGACCCTCGACACGGAGTACATCCTCATTGCCACCGGCTCCTGGCCGACGCGCCTCGGAGTCCCCGGCGACGAGTTCTGCATCACGAGCAACGAGGCCTTCTACCTCGAGGATGCCCCCAAGCGGATGCTGTGCGTCGGCGGCGGCTACATCGCCGTTGAGTTTGCCGGCATCTTCAACGGCTACAAGCCCCAGGGTGGCTATGTCGACCTGTGCTACCGCGGCGATCTTATTTTGCGCGGCTTCGATACAGAGGTGCGCAAGAGCCTGACGAAGCAGCTGGGGGCGAACGGAATAAGAGTGCGTACAAACTTGAACCCGACGAAGATCACGAAGAATGAGGACGGCTCGAATCACGTTCACTTCAACGATGGCACGGAGGAGGACTACGATCAGGTCATGCTCGCGATCGGTCGCGTGCCGCGCTCGCAGGCACTACAGCTCGGCAAGGCCGGCGTCCGAACAGGAAAGAACGGTGCCGTGCAGGTCGACGCGTATTCGAAGACATCGGTGGACAACATCTACGCCATCGGCGACGTGACGAACCGCGTGATGTTGACGCCGGTGGCCATCAACGAAGGCGCCGCCTTCGTTGAAACCGTCTTCGGTGGCAAGCCCCGCGCCACCGACCACACGAAGGTCGCGTGCGCCGTGTTCTCCATACCGCCGATCGGCACGTGCGGCATGACGGAGGAGGAGGCGGCGAAGAACTACGAAACCGTCGCCGTGTACGCGAGCTCCTTCACGCCCCTTATGCACAACATCAGCGGCAGCAAGCACAAGGAATTCATGATCCGCATCATCACGAACGAATCCAACGGCGAGGTTCTGGGTGTTCACATGCTCGGCGACAGTGCGCCTGAGATCATCCAGAGCGTCGGCATTTGCATGAAGATGGGCGCCAAGATCAGCGACTTCCACAGCACCATCGGAGTCCACCCGACGAGCGCCGAGGAGCTCTGCTCCATGCGCACTCCAGCGTACTTCTACGAGAGTGGCAAGCGCGTCGAAAAGCTCAGCAGCAACCTCTGAGAGGGAGGAGAGATGAAGAAGAACGCCTCAGCCCTTCCACCTAGCCGTGATGCACGGGTATCACAGACCAGCAGGCATGCAGAGATGGAAAGGGGAAGAGGTCAGCAGGCAGTGACGTCTGATCTCCTTCAGCGTCGGTCGCTTCGGCCGCTTCCTCTCCGACCTCCCCCCCCACCACCACTGCCACCACTCCTCCTCTCCGCATAACCTATCAGCAGTCGCGCACGCCTCATCAATACGCACGCACACCCGCCGGTGGCGGGGCGCAGGATGGAGAAGGGCAGCGGATGGAGCTTCCCTTCTCCCACGCCCTTTTCACCCCACCACGCCGTGCGGGTTTTGTGGTTTGTGCGTGTGTTTGTGCTTTCTAGTGGGGAGAAAGGCCTCTCCGCATCGTTCAACTATTTCGCTCTGCGTGTGAGCACGCGTGCGCACTTGCGCGTTCGCGTGCTCGTTCCTTTCTTGCCTTCTTTCGCCCTCCGCGTGCGTGCGTGCGTGCT

>BPK275/0 clone 18|LinJ05_V3.0350|500 bp UPS + CDS + 500 bp DWS|trypanothione reductase (TR)

ACGCACACCCGCTGGCGAAGAAAAGAGTGTCGTCATCTATCTCTCTCTTTACACTCCTACACACACACACACACACNNNNNNNNNNNNNNNNNNNNNNNNNNNNNNNNNNNNNNNNNNNNNNNNNNNNNNNNNNNNNNNNNNNNNNNNNNNNNNNNNNNNNNNNNNNNNNNNNNNGTGAGTCTTGATCCATCATTGCGCCTTCTACTTCGCTATACACAAACGCGCTCAGACGTTCACGGACACCACCGCCACCGCAGCCGCAGCAGCCGAAAGCCGCACGTAGGAATGCAGGCGAGCCTTACCACGAAGATTGTCTGATCAGCCCGTCTTTATCTCTCGCTCCCATCGTTGTATATACATTGCAGCGAGTCTTGCTGCTGGCGTTCTCTGCTCCGTGTGTGCCCACTCGCGCGCCGTTGGAGTTGTTCGCCGCGAACTCGCGCAGCCTCCCCCTCCCCTCCCCCTCTCGCGAAAATTCTTCGCTTCTCTCAACGGCTGAATGTCCCGCGCGTACGACCTCGTGGTGCTTGGCGCCGGATCTGGAGGTCTGGAGGCGGGATGGAACGCGGCCGTCACGCACAAGAAGAAGGTGGCCGTCGTCGATGTGCAGGCGACGCACGGTCCGCCGCTCTTCGCTGCGCTCGGCGGCACGTGCGTGAACGTCGGCTGCGTGCCAAAGAAACTCATGGTGACAGGTGCCCAGTACATGGACCTGATCCGTGAGTCTGGCGGCTTCGGATGGGAGATGGACCGCGAATCGCTCTGCCCCAACTGGAAGACGCTCATCGCCGCGAAGAACAAGGTGGTGAACAGCATCAACGAGAGCTACAAGAGCATGTTCGCTGATACGGAGGGCCTCAGCTTTCACATGGGCTTCGGTGCCCTTCAAGACGCTCACACGGTGGTGGTGCGCAAGTCGGAAGACCCACACAGCGACGTGCTGGAGACCCTCGACACGGAGTACATCCTCATTGCCACCGGCTCCTGGCCGACGCGCCTCGGAGTCCCCGGCGACGAGTTCTGCATCACGAGCAACGAGGCCTTCTACCTCGAGGATGCCCCCAAGCGGATGCTGTGCGTCGGCGGCGGCTACATCGCCGTTGAGTTTGCCGGCATCTTCAACGGCTACAAGCCCCAGGGTGGCTATGTCGACCTGTGCTACCGCGGCGATCTTATTTTGCGCGGCTTCGATACAGAGGTGCGCAAGAGCCTGACGAAGCAGCTGGGGGCGAACGGAATAAGAGTGCGTACAAACTTGAACCCGACGAAGATCACGAAGAATGAGGACGGCTCGAATCACGTTCACTTCAACGATGGCACGGAGGAGGACTACGATCAGGTCATGCTCGCGATCGGTCGCGTGCCGCGCTCGCAGGCACTACAGCTCGGCAAGGCCGGCGTCCGAACAGGAAAGAACGGTGCCGTGCAGGTCGACGCGTATTCGAAGACATCGGTGGACAACATCTACGCCATCGGCGACGTGACGAACCGCGTGATGTTGACGCCGGTGGCCATCAACGAAGGCGCCGCCTTCGTTGAAACCGTCTTCGGTGGCAAGCCCCGCGCCACCGACCACACGAAGGTCGCGTGCGCCGTGTTCTCCATACCGCCGATCGGCACGTGCGGCATGACGGAGGAGGAGGCGGCGAAGAACTACGAAACCGTCGCCGTGTACGCGAGCTCCTTCACGCCCCTTATGCACAACATCAGCGGCAGCAAGCACAAGGAATTCATGATCCGCATCATCACGAACGAATCCAACGGCGAGGTTCTGGGTGTTCACATGCTCGGCGACAGTGCGCCTGAGATCATCCAGAGCGTCGGCATTTGCATGAAGATGGGCGCCAAGATCAGCGACTTCCACAGCACCATCGGAGTCCACCCGACGAGCGCCGAGGAGCTCTGCTCCATGCGCACTCCAGCGTACTTCTACGAGAGTGGCAAGCGCGTCGAAAAGCTCAGCAGCAACCTCTGAGAGGGAGGAGAGATGAAGAAGAACGCCTCAGCCCTTCCACCTAGCCGTGATGCACGGGTATCACAGACCAGCAGGCATGCAGAGATGGAAAGGGGAAGAGGTCAGCAGGCAGTGACGTCTGATCTCCTTCAGCGTCGGTCGCTTCGGCCGCTTCCTCTCCGACCTCCCCCCCCACCACCACTGCCACCACTCCTCCTCTCCGCATAACCTATCAGCAGTCGCGCACGCCTCATCAATACGCACGCACACCCGCCGGTGGCGGGGCGCAGGATGGAGAAGGGCAGCGGATGGAGCTTCCCTTCTCCCACGCCCTTTTCACCCCACCACGCCGTGCGGGTTTTGTGGTTTGTGCGTGTGTTTGTGCTTTCTAGTGGGGAGAAAGGCCTCTCCGCATCGTTCAACTATTTCGCTCTGCGTGTGAGCACGCGTGCGCACTTGCGCGTTCGCGTGCTCGTTCCTTTCTTGCCTTCTTTCGCCCTCCGCGTGCGTGCGTGCGTGCT

>BPK282/0 clone 4|LinJ05_V3.0350|500 bp UPS + CDS + 500 bp DWS|trypanothione reductase (TR)

ACGCACACCCGCTGGCGAAGAAAAGAGTGTCGTCATCTATCTCTCTCTTTACACTCCTACACACACACACACACACNNNNNNNNNNNNNNNNNNNNNNNNNNNNNNNNNNNNNNNNNNNNNNNNNNNNNNNNNNNNNNNNNNNNNNNNNNNNNNNNNNNNNNNNNNNNNNNNNNNGTGAGTCTTGATCCATCATTGCGCCTTCTACTTCGCTATACACAAACGCGCTCAGACGTTCACGGACACCACCGCCACCGCAGCCGCAGCAGCCGAAAGCCGCACGTAGGAATGCAGGCGAGCCTTACCACGAAGATTGTCTGATCAGCCCGTCTTTATCTCTCGCTCCCATCGTTGTATATACATTGCAGCGAGTCTTGCTGCTGGCGTTCTCTGCTCCGTGTGTGCCCACTCGCGCGCCGTTGGAGTTGTTCGCCGCGAACTCGCGCAGCCTCCCCCTCCCCTCCCCCTCTCGCGAAAATTCTTCGCTTCTCTCAACGGCTGAATGTCCCGCGCGTACGACCTCGTGGTGCTTGGCGCCGGATCTGGAGGTCTGGAGGCGGGATGGAACGCGGCCGTCACGCACAAGAAGAAGGTGGCCGTCGTCGATGTGCAGGCGACGCACGGTCCGCCGCTCTTCGCTGCGCTCGGCGGCACGTGCGTGAACGTCGGCTGCGTGCCAAAGAAACTCATGGTGACAGGTGCCCAGTACATGGACCTGATCCGTGAGTCTGGCGGCTTCGGATGGGAGATGGACCGCGAATCGCTCTGCCCCAACTGGAAGACGCTCATCGCCGCGAAGAACAAGGTGGTGAACAGCATCAACGAGAGCTACAAGAGCATGTTCGCTGATACGGAGGGCCTCAGCTTTCACATGGGCTTCGGTGCCCTTCAAGACGCTCACACGGTGGTGGTGCGCAAGTCGGAAGACCCACACAGCGACGTGCTGGAGACCCTCGACACGGAGTACATCCTCATTGCCACCGGCTCCTGGCCGACGCGCCTCGGAGTCCCCGGCGACGAGTTCTGCATCACGAGCAACGAGGCCTTCTACCTCGAGGATGCCCCCAAGCGGATGCTGTGCGTCGGCGGCGGCTACATCGCCGTTGAGTTTGCCGGCATCTTCAACGGCTACAAGCCCCAGGGTGGCTATGTCGACCTGTGCTACCGCGGCGATCTTATTTTGCGCGGCTTCGATACAGAGGTGCGCAAGAGCCTGACGAAGCAGCTGGGGGCGAACGGAATAAGAGTGCGTACAAACTTGAACCCGACGAAGATCACGAAGAATGAGGACGGCTCGAATCACGTTCACTTCAACGATGGCACGGAGGAGGACTACGATCAGGTCATGCTCGCGATCGGTCGCGTGCCGCGCTCGCAGGCACTACAGCTCGGCAAGGCCGGCGTCCGAACAGGAAAGAACGGTGCCGTGCAGGTCGACGCGTATTCGAAGACATCGGTGGACAACATCTACGCCATCGGCGACGTGACGAACCGCGTGATGTTGACGCCGGTGGCCATCAACGAAGGCGCCGCCTTCGTTGAAACCGTCTTCGGTGGCAAGCCCCGCGCCACCGACCACACGAAGGTCGCGTGCGCCGTGTTCTCCATACCGCCGATCGGCACGTGCGGCATGACGGAGGAGGAGGCGGCGAAGAACTACGAAACCGTCGCCGTGTACGCGAGCTCCTTCACGCCCCTTATGCACAACATCAGCGGCAGCAAGCACAAGGAATTCATGATCCGCATCATCACGAACGAATCCAACGGCGAGGTTCTGGGTGTTCACATGCTCGGCGACAGTGCGCCTGAGATCATCCAGAGCGTCGGCATTTGCATGAAGATGGGCGCCAAGATCAGCGACTTCCACAGCACCATCGGAGTCCACCCGACGAGCGCCGAGGAGCTCTGCTCCATGCGCACTCCAGCGTACTTCTACGAGAGTGGCAAGCGCGTCGAAAAGCTCAGCAGCAACCTCTGAGAGGGAGGAGAGATGAAGAAGAACGCCTCAGCCCTTCCACCTAGCCGTGATGCACGGGTATCACAGACCAGCAGGCATGCAGAGATGGAAAGGGGAAGAGGTCAGCAGGCAGTGACGTCTGATCTCCTTCAGCGTCGGTCGCTTCGGCCGCTTCCTCTCCGACCTCCCCCCCCACCACCACTGCCACCACTCCTCCTCTCCGCATAACCTATCAGCAGTCGCGCACGCCTCATCAATACGCACGCACACCCGCCGGTGGCGGGGCGCAGGATGGAGAAGGGCAGCGGATGGAGCTTCCCTTCTCCCACGCCCTTTTCACCCCACCACGCCGTGCGGGTTTTGTGGTTTGTGCGTGTGTTTGTGCTTTCTAGTGGGGAGAAAGGCCTCTCCGCATCGTTCAACTATTTCGCTCTGCGTGTGAGCACGCGTGCGCACTTGCGCGTTCGCGTGCTCGTTCCTTTCTTGCCTTCTTTCGCCCTCCGCGTGCGTGCGTGCGTGCT

>BPK294/0 clone 1|LinJ05_V3.0350|500 bp UPS + CDS + 500 bp DWS|trypanothione reductase (TR)

ACGCACACCCGCTGGCGAAGAAAAGAGTGTCGTCATCTATCTCTCTCTTTACACTCCTACACACACACACACACACNNNNNNNNNNNNNNNNNNNNNNNNNNNNNNNNNNNNNNNNNNNNNNNNNNNNNNNNNNNNNNNNNNNNNNNNNNNNNNNNNNNNNNNNNNNNNNNNNNNGTGAGTCTTGATCCATCATTGCGCCTTCTACTTCGCTATACACAAACGCGCTCAGACGTTCACGGACACCACCGCCACCGCAGCCGCAGCAGCCGAAAGCCGCACGTAGGAATGCAGGCGAGCCTTACCACGAAGATTGTCTGATCAGCCCGTCTTTATCTCTCGCTCCCATCGTTGTATATACATTGCAGCGAGTCTTGCTGCTGGCGTTCTCTGCTCCGTGTGTGCCCACTCGCGCGCCGTTGGAGTTGTTCGCCGCGAACTCGCGCAGCCTCCCCCTCCCCTCCCCCTCTCGCGAAAATTCTTCGCTTCTCTCAACGGCTGAATGTCCCGCGCGTACGACCTCGTGGTGCTTGGCGCCGGATCTGGAGGTCTGGAGGCGGGATGGAACGCGGCCGTCACGCACAAGAAGAAGGTGGCCGTCGTCGATGTGCAGGCGACGCACGGTCCGCCGCTCTTCGCTGCGCTCGGCGGCACGTGCGTGAACGTCGGCTGCGTGCCAAAGAAACTCATGGTGACAGGTGCCCAGTACATGGACCTGATCCGTGAGTCTGGCGGCTTCGGATGGGAGATGGACCGCGAATCGCTCTGCCCCAACTGGAAGACGCTCATCGCCGCGAAGAACAAGGTGGTGAACAGCATCAACGAGAGCTACAAGAGCATGTTCGCTGATACGGAGGGCCTCAGCTTTCACATGGGCTTCGGTGCCCTTCAAGACGCTCACACGGTGGTGGTGCGCAAGTCGGAAGACCCACACAGCGACGTGCTGGAGACCCTCGACACGGAGTACATCCTCATTGCCACCGGCTCCTGGCCGACGCGCCTCGGAGTCCCCGGCGACGAGTTCTGCATCACGAGCAACGAGGCCTTCTACCTCGAGGATGCCCCCAAGCGGATGCTGTGCGTCGGCGGCGGCTACATCGCCGTTGAGTTTGCCGGCATCTTCAACGGCTACAAGCCCCAGGGTGGCTATGTCGACCTGTGCTACCGCGGCGATCTTATTTTGCGCGGCTTCGATACAGAGGTGCGCAAGAGCCTGACGAAGCAGCTGGGGGCGAACGGAATAAGAGTGCGTACAAACTTGAACCCGACGAAGATCACGAAGAATGAGGACGGCTCGAATCACGTTCACTTCAACGATGGCACGGAGGAGGACTACGATCAGGTCATGCTCGCGATCGGTCGCGTGCCGCGCTCGCAGGCACTACAGCTCGGCAAGGCCGGCGTCCGAACAGGAAAGAACGGTGCCGTGCAGGTCGACGCGTATTCGAAGACATCGGTGGACAACATCTACGCCATCGGCGACGTGACGAACCGCGTGATGTTGACGCCGGTGGCCATCAACGAAGGCGCCGCCTTCGTTGAAACCGTCTTCGGTGGCAAGCCCCGCGCCACCGACCACACGAAGGTCGCGTGCGCCGTGTTCTCCATACCGCCGATCGGCACGTGCGGCATGACGGAGGAGGAGGCGGCGAAGAACTACGAAACCGTCGCCGTGTACGCGAGCTCCTTCACGCCCCTTATGCACAACATCAGCGGCAGCAAGCACAAGGAATTCATGATCCGCATCATCACGAACGAATCCAACGGCGAGGTTCTGGGTGTTCACATGCTCGGCGACAGTGCGCCTGAGATCATCCAGAGCGTCGGCATTTGCATGAAGATGGGCGCCAAGATCAGCGACTTCCACAGCACCATCGGAGTCCACCCGACGAGCGCCGAGGAGCTCTGCTCCATGCGCACTCCAGCGTACTTCTACGAGAGTGGCAAGCGCGTCGAAAAGCTCAGCAGCAACCTCTGAGAGGGAGGAGAGATGAAGAAGAACGCCTCAGCCCTTCCACCTAGCCGTGATGCACGGGTATCACAGACCAGCAGGCATGCAGAGATGGAAAGGGGAAGAGGTCAGCAGGCAGTGACGTCTGATCTCCTTCAGCGTCGGTCGCTTCGGCCGCTTCCTCTCCGACCTCCCCCCCCMCCACCACTGCCACCACTCCTCCTCTCCGCATAACCTATCAGCAGTCGCGCACGCCTCATCAATACGCACGCACACCCGCCGGTGGCGGGGCGCAGGATGGAGAAGGGCAGCGGATGGAGCTTCCCTTCTCCCACGCCCTTTTCACCCCACCACGCCGTGCGGGTTTTGTGGTTTGTGCGTGTGTTTGTGCTTTCTAGTGGGGAGAAAGGCCTCTCCGCATCGTTCAACTATTTCGCTCTGCGTGTGAGCACGCGTGCGCACTTGCGCGTTCGCGTGCTCGTTCCTTTCTTGCCTTCTTTCGCCCTCCGCGTGCGTGCGTGCGTGCT

>BPK298/0 clone 8|LinJ05_V3.0350|500 bp UPS + CDS + 500 bp DWS|trypanothione reductase (TR)

ACGCACACCCGCTGGCGAAGAAAAGAGTGTCGTCATCTATCTCTCTCTTTACACTCCTACACACACACACACACACNNNNNNNNNNNNNNNNNNNNNNNNNNNNNNNNNNNNNNNNNNNNNNNNNNNNNNNNNNNNNNNNNNNNNNNNNNNNNNNNNNNNNNNNNNNNNNNNNNNGTGAGTCTTGATCCATCATTGCGCCTTCTACTTCGCTATACACAAACGCGCTCAGACGTTCACGGACACCACCGCCACCGCAGCCGCAGCAGCCGAAAGCCGCACGTAGGAATGCAGGCGAGCCTTACCACGAAGATTGTCTGATCAGCCCGTCTTTATCTCTCGCTCCCATCGTTGTATATACATTGCAGCGAGTCTTGCTGCTGGCGTTCTCTGCTCCGTGTGTGCCCACTCGCGCGCCGTTGGAGTTGTTCGCCGCGAACTCGCGCAGCCTCCCCCTCCCCTCCCCCTCTCGCGAAAATTCTTCGCTTCTCTCAACGGCTGAATGTCCCGCGCGTACGACCTCGTGGTGCTTGGCGCCGGATCTGGAGGTCTGGAGGCGGGATGGAACGCGGCCGTCACGCACAAGAAGAAGGTGGCCGTCGTCGATGTGCAGGCGACGCACGGTCCGCCGCTCTTCGCTGCGCTCGGCGGCACGTGCGTGAACGTCGGCTGCGTGCCAAAGAAACTCATGGTGACAGGTGCCCAGTACATGGACCTGATCCGTGAGTCTGGCGGCTTCGGATGGGAGATGGACCGCGAATCGCTCTGCCCCAACTGGAAGACGCTCATCGCCGCGAAGAACAAGGTGGTGAACAGCATCAACGAGAGCTACAAGAGCATGTTCGCTGATACGGAGGGCCTCAGCTTTCACATGGGCTTCGGTGCCCTTCAAGACGCTCACACGGTGGTGGTGCGCAAGTCGGAAGACCCACACAGCGACGTGCTGGAGACCCTCGACACGGAGTACATCCTCATTGCCACCGGCTCCTGGCCGACGCGCCTCGGAGTCCCCGGCGACGAGTTCTGCATCACGAGCAACGAGGCCTTCTACCTCGAGGATGCCCCCAAGCGGATGCTGTGCGTCGGCGGCGGCTACATCGCCGTTGAGTTTGCCGGCATCTTCAACGGCTACAAGCCCCAGGGTGGCTATGTCGACCTGTGCTACCGCGGCGATCTTATTTTGCGCGGCTTCGATACAGAGGTGCGCAAGAGCCTGACGAAGCAGCTGGGGGCGAACGGAATAAGAGTGCGTACAAACTTGAACCCGACGAAGATCACGAAGAATGAGGACGGCTCGAATCACGTTCACTTCAACGATGGCACGGAGGAGGACTACGATCAGGTCATGCTCGCGATCGGTCGCGTGCCGCGCTCGCAGGCACTACAGCTCGGCAAGGCCGGCGTCCGAACAGGAAAGAACGGTGCCGTGCAGGTCGACGCGTATTCGAAGACATCGGTGGACAACATCTACGCCATCGGCGACGTGACGAACCGCGTGATGTTGACGCCGGTGGCCATCAACGAAGGCGCCGCCTTCGTTGAAACCGTCTTCGGTGGCAAGCCCCGCGCCACCGACCACACGAAGGTCGCGTGCGCCGTGTTCTCCATACCGCCGATCGGCACGTGCGGCATGACGGAGGAGGAGGCGGCGAAGAACTACGAAACCGTCGCCGTGTACGCGAGCTCCTTCACGCCCCTTATGCACAACATCAGCGGCAGCAAGCACAAGGAATTCATGATCCGCATCATCACGAACGAATCCAACGGCGAGGTTCTGGGTGTTCACATGCTCGGCGACAGTGCGCCTGAGATCATCCAGAGCGTCGGCATTTGCATGAAGATGGGCGCCAAGATCAGCGACTTCCACAGCACCATCGGAGTCCACCCGACGAGCGCCGAGGAGCTCTGCTCCATGCGCACTCCAGCGTACTTCTACGAGAGTGGCAAGCGCGTCGAAAAGCTCAGCAGCAACCTCTGAGAGGGAGGAGAGATGAAGAAGAACGCCTCAGCCCTTCCACCTAGCCGTGATGCACGGGTATCACAGACCAGCAGGCATGCAGAGATGGAAAGGGGAAGAGGTCAGCAGGCAGTGACGTCTGATCTCCTTCAGCGTCGGTCGCTTCGGCCGCTTCCTCTCCGACCTCCCCCCCCACCACCACTGCCACCACTCCTCCTCTCCGCATAACCTATCAGCAGTCGCGCACGCCTCATCAATACGCACGCACACCCGCCGGTGGCGGGGCGCAGGATGGAGAAGGGCAGCGGATGGAGCTTCCCTTCTCCCACGCCCTTTTCACCCCACCACGCCGTGCGGGTTTTGTGGTTTGTGCGTGTGTTTGTGCTTTCTAGTGGGGAGAAAGGCCTCTCCGCATCGTTCAACTATTTCGCTCTGCGTGTGAGCACGCGTGCGCACTTGCGCGTTCGCGTGCTCGTTCCTTTCTTGCCTTCTTTCGCCCTCCGCGTGCGTGCGTGCGTGCT

### 5. ARSENATE REDUCTASE 2

>BPK035/0 clone 1|LinJ32_V3.2880|500 bp UPS + CDS + 500 bp DWS|arsenate reductase 2

AGTTGTAAAGAGCGACGGCACTGGCTGTGCGTGCAGCGCAGCGGTAGCTGACAACTGCCGTGCGTGTGGTGAAGGTGCAGAGGTCGAAGGAGGCGATGCGGCGTCGCCGTCGGCTTCTCCCACGTGTATTGTCGCTTCTACACGTTTTGTGACGGGCGCTGCCTGTGTGTGCGTGCGTGCGTGTGCGCTCGTCAGCTGCGTTGCAAAGACAGCGGCACCCAGGAGCAAGAGCGCGTCTTTGATGGCCGGAGGTGCACTTCAAGCTCATCTCCCTTGCTTCGTTTTTCGACCCCGCGCATGCAGAGACGCGCACACGACGAAGCCAAAAGGAAAAGACAGATAAACGGCGTCGAGGCAGCCTTCCAGGCCTGCTTTCATCGGCGAAGGACAAGGTAGCAGCGATGCTGCGTTGCACCTCTTCCTCTCTCTTCGGCGCTTGAGCCCTTGCTCATTTCCGCAGAAGAGGCAGCAGTAGCAGCCGCCATCATCCCTTCCGCGCAATGGCGAACTACACGTACATGAAGCCCGAAGAGCTGGTGGAGGTGCTCGACAATCCTGACTCACTCACCAAAGCCGCGGTGATAGACTGCAGGGATAGTGATCGAGACTGCGGCTTCATCGTGAACTCGATAAGCATGCCGACGATTAGCTGCACGGAAGAGATGTACGAAAAGCTCGCCAAGACGCTCTTCGAAGAGAAGAAAGAGATCGCTGTCTTTCACTGTGCCCAGTCGCTCATACGGGCCCCCAAGGGAGCCAACCGCTTCGTTCTGGCACAGAAGAAGCTCGGCTACGTGCTCCCTGCCGTGTACGTGCTGCGTGGTGGGTGGGAGGCGTTCTACCATATGTATGGCGACGTGCGGCCGGACCTCATTTATGTGTGAGCCATGAAGCAGCAAATGAGGCGGTGGTGGGCAAGACAGTGCTGCCGCTCCAGTGGCGCGCCAGCGCACCGCGACGGCCCATCCTCTGCCGAAAGTAGCGCGCACAGACGCAAAAAGGGGTGAGTGCGTGGCTGTTCTGTTCCCTTGCATGTGCGCTGCTCTAACTTTTACGTTGTGGGCCGCACGGAAAGGATGCACAGAAGCCCTTCCTCATGCGACAGGATTGCCATTTTGGCGCGCGTGCTTGCTTGCCACCTCACTGCCTTCTTTTTGGGTTTCTTTGTGGCTCTTACGGTGCCGCTTTTCGAACATGGTGACTGTCGTCGTGCACTCTAGTTTGCTTTGCGGCGACTTTCTACGTCCCTCGGTGGCGGCCGCGACGTACTCCTGACAAAGGAAGCAATCTGCACACTGTGTCTAAGCGCCGTGCGTGAGTGAGTGCCATTCACCGGCGCCGCTTTATGCGCTAATCTTCTTTATACCTCACTTGGCTGCGCCGC

>BPK043/0 clone 2|LinJ32_V3.2880|500 bp UPS + CDS + 500 bp DWS|arsenate reductase 2

AGTTGTAAAGAGCGACGGCACTGGCTGTGCGTGCAGCGCAGCGGTAGCTGACAACTGCCGTGCGTGTGGTGAAGGTGCAGAGGTCGAAGGAGGCGATGCGGCGTCGCCGTCGGCTTCTCCCACGTGTATTGTCGCTTCTACACGTTTTGTGACGGGCGCTGCCTGTGTGTGCGTGCGTGCGTGTGCGCTCGTCAGCTGCGTTGCAAAGACAGCGGCACCCAGGAGCAAGAGCGCGTCTTTGATGGCCGGAGGTGCACTTCAAGCTCATCTCCCTTGCTTCGTTTTTCGACCCCGCGCATGCAGAGACGCGCACACGACGAAGCCAAAAGGAAAAGACAGATAAACGGCGTCGAGGCAGCCTTCCAGGCCTGCTTTCATCGGCGAAGGACAAGGTAGCAGCGATGCTGCGTTGCACCTCTTCCTCTCTCTTCGGCGCTTGAGCCCTTGCTCATTTCCGCAGAAGAGGCAGCAGTAGCAGCCGCCATCATCCCTTCCGCGCAATGGCGAACTACACGTACATGAAGCCCGAAGAGCTGGTGGAGGTGCTCGACAATCCTGACTCACTCACCAAAGCCGCGGTGATAGACTGCAGGGATAGTGATCGAGACTGCGGCTTCATCGTGAACTCGATAAGCATGCCGACGATTAGCTGCACGGAAGAGATGTACGAAAAGCTCGCCAAGACGCTCTTCGAAGAGAAGAAAGAGATCGCTGTCTTTCACTGTGCCCAGTCGCTCATACGGGCCCCCAAGGGAGCCAACCGCTTCGTTCTGGCACAGAAGAAGCTCGGCTACGTGCTCCCTGCCGTGTACGTGCTGCGTGGTGGGTGGGAGGCGTTCTACCATATGTATGGCGACGTGCGGCCGGACCTCATTTATGTGTGAGCCATGAAGCAGCAAATGAGGCGGTGGTGGGCAAGACAGTGCTGCCGCTCCAGTGGCGCGCCAGCGCACCGCGACGGCCCATCCTCTGCCGAAAGTAGCGCGCACAGACGCAAAAAGGGGTGAGTGCGTGGCTGTTCTGTTCCCTTGCATGTGCGCTGCTCTAACTTTTACGTTGTGGGCCGCACGGAAAGGATGCACAGAAGCCCTTCCTCATGCGACAGGATTGCCATTTTGGCGCGCGTGCTTGCTTGCCACCTCACTGCCTTCTTTTTGGGTTTCTTTGTGGCTCTTACGGTGCCGCTTTTCGAACATGGTGACTGTCGTCGTGCACTCTAGTTTGCTTTGCGGCGACTTTCTACGTCCCTCGGTGGCGGCCGCGACGTACTCCTGACAAAGGAAGCAATCTGCACACTGTGTCTAAGCGCCGTGCGTGAGTGAGTGCCATTCACCGGCGCCGCTTTATGCGCTAATCTTCTTTATACCTCACTTGGCTGCGCCGC

>BPK085/0 clone 8|LinJ32_V3.2880|500 bp UPS + CDS + 500 bp DWS|arsenate reductase 2

AGTTGTAAAGAGCGACGGCACTGGCTGTGCGTGCAGCGCAGCGGTAGCTGACAACTGCCGTGCGTGTGGTGAAGGTGCAGAGGTCGAAGGAGGCGATGCGGCGTCGCCGTCGGCTTCTCCCACGTGTATTGTCGCTTCTACACGTTTTGTGACGGGCGCTGCCTGTGTGTGCGTGCGTGCGTGTGCGCTCGTCAGCTGCGTTGCAAAGACAGCGGCACCCAGGAGCAAGAGCGCGTCTTTGATGGCCGGAGGTGCACTTCAAGCTCATCTCCCTTGCTTCGTTTTTCGACCCCGCGCATGCAGAGACGCGCACACGACGAAGCCAAAAGGAAAAGACAGATAAACGGCGTCGAGGCAGCCTTCCAGGCCTGCTTTCATCGGCGAAGGACAAGGTAGCAGCGATGCTGCGTTGCACCTCTTCCTCTCTCTTCGGCGCTTGAGCCCTTGCTCATTTCCGCAGAAGAGGCAGCAGTAGCAGCCGCCATCATCCCTTCCGCGCAATGGCGAACTACACGTACATGAAGCCCGAAGAGCTGGTGGAGGTGCTCGACAATCCTGACTCACTCACCAAAGCCGCGGTGATAGACTGCAGGGATAGTGATCGAGACTGCGGCTTCATCGTGAACTCGATAAGCATGCCGACGATTAGCTGCACGGAAGAGATGTACGAAAAGCTCGCCAAGACGCTCTTCGAAGAGAAGAAAGAGATCGCTGTCTTTCACTGTGCCCAGTCGCTCATACGGGCCCCCAAGGGAGCCAACCGCTTCGTTCTGGCACAGAAGAAGCTCGGCTACGTGCTCCCTGCCGTGTACGTGCTGCGTGGTGGGTGGGAGGCGTTCTACCATATGTATGGCGACGTGCGGCCGGACCTCATTTATGTGTGAGCCATGAAGCAGCAAATGAGGCGGTGGMGGGCAAGACAGTGCTGCCGCTCCAGTGGCGCGCCAGCGCACCGCGACGGCCCATCCTCTGCCGAAAGTAGCGCGCACAGACGCAAAAAGGGGTGAGTGCGTGGCTGTTCTGTTCCCTTGCATGTGCGCTGCTCTAACTTTTACGTTGTGGGCCGCACGGAAAGGATGCACAGAAGCCCTTCCTCATGCGACAGGATTGCCATTTTGGCGCGCGTGCTTGCTTGCCACCTCACTGCCTTCTTTTTGGGTTTCTTTGTGGCTCTTACGGTGCCGCTTTTCGAACATGGTGACTGTCGTCGTGCACTCTAGTTTGCTTTGCGGCGACTTTCTACGTCCCTCGGTGGCGGCCGCGACGTACTCCTGACAAAGGAAGCAATCTGCACACTGTGTCTAAGCGCCGTGCGTGAGTGAGTGCCATTCACCGGCGCCGCTTTATGCGCTAATCTTCTTTATACCTCACTTGGCTGCGCCGC

>BPK087/0 clone 11|LinJ32_V3.2880|500 bp UPS + CDS + 500 bp DWS|arsenate reductase 2

AGTTGTAAAGAGCGACGGCACTGGCTGTGCGTGCAGCGCAGCGGTAGCTGACAACTGCCGTGCGTGTGGTGAAGGTGCAGAGGTCGAAGGAGGCGATGCGGCGTCGCCGTCGGCTTCTCCCACGTGTATTGTCGCTTCTACACGTTTTGTGACGGGCGCTGCCTGTGTGTGCGTGCGTGCGTGTGCGCTCGTCAGCTGCGTTGCAAAGACAGCGGCACCCAGGAGCAAGAGCGCGTCTTTGATGGCCGGAGGTGCACTTCAAGCTCATCTCCCTTGCTTCGTTTTTCGACCCCGCGCATGCAGAGACGCGCACACGACGAAGCCAAAAGGAAAAGACAGATAAACGGCGTCGAGGCAGCCTTCCAGGCCTGCTTTCATCGGCGAAGGACAAGGTAGCAGCGATGCTGCGTTGCACCTCTTCCTCTCTCTTCGGCGCTTGAGCCCTTGCTCATTTCCGCAGAAGAGGCAGCAGTAGCAGCCGCCATCATCCCTTCCGCGCAATGGCGAACTACACGTACATGAAGCCCGAAGAGCTGGTGGAGGTGCTCGACAATCCTGACTCACTCACCAAAGCCGCGGTGATAGACTGCAGGGATAGTGATCGAGACTGCGGCTTCATCGTGAACTCGATAAGCATGCCGACGATTAGCTGCACGGAAGAGATGTACGAAAAGCTCGCCAAGACGCTCTTCGAAGAGAAGAAAGAGATCGCTGTCTTTCACTGTGCCCAGTCGCTCATACGGGCCCCCAAGGGAGCCAACCGCTTCGTTCTGGCACAGAAGAAGCTCGGCTACGTGCTCCCTGCCGTGTACGTGCTGCGTGGTGGGTGGGAGGCGTTCTACCATATGTATGGCGACGTGCGGCCGGACCTCATTTATGTGTGAGCCATGAAGCAGCAAATGAGGCGGTGGTGGGSAAGACAGTGCTGCCGCTCCAGTGGCGCGCCAGCGCACCGCGACGGCCCATCCTCTGCCGAAAGTAGCGCGCACAGACGCAAAAAGGGGTGAGTGCGTGGCTGTTCTGTTCCCTTGCATGTGCGCTGCTCTAACTTTTACGTTGTGGGCCGCACGGAAAGGATGCACAGAAGCCCTTCCTCATGCGACAGGATTGCCATTTTGGCGCGCGTGCTTGCTTGCCACCTCACTGCCTTCTTTTTGGGTTTCTTTGTGGCTCTTACGGTGCCGCTTTTCGAACATGGTGACTGTCGTCGTGCACTCTAGTTTGCTTTGCGGCGACTTTCTACGTCCCTCGGTGGCGGCCGCGACGTACTCCTGACAAAGGAAGCAATCTGCACACTGTGTCTAAGCGCCGTGCGTGAGTGAGTGCCATTCACCGGCGCCGCTTTATGCGCTAATCTTCTTTATACCTCACTTGGCTGCGCCGC

>BPK178/0 clone 3|LinJ32_V3.2880|500 bp UPS + CDS + 500 bp DWS|arsenate reductase 2

AGTTGTAAAGAGCGACGGCACTGGCTGTGCGTGCAGCGCAGCGGTAGCTGACAACTGCCGTGCGTGTGGTGAAGGTGCAGAGGTCGAAGGAGGCGATGCGGCGTCGCCGTCGGCTTCTCCCACGTGTATTGTCGCTTCTACACGTTTTGTGACGGGCGCTGCCTGTGTGTGCGTGCGTGCGTGTGCGCTCGTCAGCTGCGTTGCAAAGACAGCGGCACCCAGGAGCAAGAGCGCGTCTTTGATGGCCGGAGGTGCACTTCAAGCTCATCTCCCTTGCTTCGTTTTTCGACCCCGCGCATGCAGAGACGCGCACACGACGAAGCCAAAAGGAAAAGACAGATAAACGGCGTCGAGGCAGCCTTCCAGGCCTGCTTTCATCGGCGAAGGACAAGGTAGCAGCGATGCTGCGTTGCACCTCTTCCTCTCTCTTCGGCGCTTGAGCCCTTGCTCATTTCCGCAGAAGAGGCAGCAGTAGCAGCCGCCATCATCCCTTCCGCGCAATGGCGAACTACACGTACATGAAGCCCGAAGAGCTGGTGGAGGTGCTCGACAATCCTGACTCACTCACCAAAGCCGCGGTGATAGACTGCAGGGATAGTGATCGAGACTGCGGCTTCATCGTGAACTCGATAAGCATGCCGACGATTAGCTGCACGGAAGAGATGTACGAAAAGCTCGCCAAGACGCTCTTCGAAGAGAAGAAAGAGATCGCTGTCTTTCACTGTGCCCAGTCGCTCATACGGGCCCCCAAGGGAGCCAACCGCTTCGTTCTGGCACAGAAGAAGCTCGGCTACGTGCTCCCTGCCGTGTACGTGCTGCGTGGTGGGTGGGAGGCGTTCTACCATATGTATGGCGACGTGCGGCCGGACCTCATTTATGTGTGAGCCATGAAGCAGCAAATGAGGCGGTGGTGGGCAAGACAGTGCTGCCGCTCCAGTGGCGCGCCAGCGCACCGCGACGGCCCATCCTCTGCCGAAAGTAGCGCGCACAGACGCAAAAAGGGGTGAGTGCGTGGCTGTTCTGTTCCCTTGCATGTGCGCTGCTCTAACTTTTACGTTGTGGGCCGCACGGAAAGGATGCACAGAAGCCCTTCCTCATGCGACAGGATTGCCATTTTGGCGCGCGTGCTTGCTTGCCACCTCACTGCCTTCTTTTTGGGTTTCTTTGTGGCTCTTACGGTGCCGCTTTTCGAACATGGTGACTGTCGTCGTGCACTCTAGTTTGCTTTGCGGCGACTTTCTACGTCCCTCGGTGGCGGCCGCGACGTACTCCTGACAAAGGAAGCAATCTGCACACTGTGTCTAAGCGCCGTGCGTGAGTGAGTGCCATTCACCGGCGCCGCTTTATGCGCTAATCTTCTTTATACCTCACTTGGCTGCGCCGC

>BPK190/0 clone 3|LinJ32_V3.2880|500 bp UPS + CDS + 500 bp DWS|arsenate reductase 2

AGTTGTAAAGAGCGACGGCACTGGCTGTGCGTGCAGCGCAGCGGTAGCTGACAACTGCCGTGCGTGTGGTGAAGGTGCAGAGGTCGAAGGAGGCGATGCGGCGTCGCCGTCGGCTTCTCCCACGTGTATTGTCGCTTCTACACGTTTTGTGACGGGCGCTGCCTGTGTGTGCGTGCGTGCGTGTGCGCTCGTCAGCTGCGTTGCAAAGACAGCGGCACCCAGGAGCAAGAGCGCGTCTTTGATGGCCGGAGGTGCACTTCAAGCTCATCTCCCTTGCTTCGTTTTTCGACCCCGCGCATGCAGAGACGCGCACACGACGAAGCCAAAAGGAAAAGACAGATAAACGGCGTCGAGGCAGCCTTCCAGGCCTGCTTTCATCGGCGAAGGACAAGGTAGCAGCGATGCTGCGTTGCACCTCTTCCTCTCTCTTCGGCGCTTGAGCCCTTGCTCATTTCCGCAGAAGAGGCAGCAGTAGCAGCCGCCATCATCCCTTCCGCGCAATGGCGAACTACACGTACATGAAGCCCGAAGAGCTGGTGGAGGTGCTCGACAATCCTGACTCACTCACCAAAGCCGCGGTGATAGACTGCAGGGATAGTGATCGAGACTGCGGCTTCATCGTGAACTCGATAAGCATGCCGACGATTAGCTGCACGGAAGAGATGTACGAAAAGCTCGCCAAGACGCTCTTCGAAGAGAAGAAAGAGATCGCTGTCTTTCACTGTGCCCAGTCGCTCATACGGGCCCCCAAGGGAGCCAACCGCTTCGTTCTGGCACAGAAGAAGCTCGGCTACGTGCTCCCTGCCGTGTACGTGCTGCGTGGTGGGTGGGAGGCGTTCTACCATATGTATGGCGACGTGCGGCCGGACCTCATTTATGTGTGAGCCATGAAGCAGCAAATGAGGCGGTGGMGGGSAAGACAGTGCTGCCGCTCCAGTGGCGCGCCAGCGCACCGCGACGGCCCATCCTCTGCCGAAAGTAGCGCGCACAGACGCAAAAAGGGGTGAGTGCGTGGCTGTTCTGTTCCCTTGCATGTGCGCTGCTCTAACTTTTACGTTGTGGGCCGCACGGAAAGGATGCACAGAAGCCCTTCCTCATGCGACAGGATTGCCATTTTGGCGCGCGTGCTTGCTTGCCACCTCACTGCCTTCTTTTTGGGTTTCTTTGTGGCTCTTACGGTGCCGCTTTTCGAACATGGTGACTGTCGTCGTGCACTCTAGTTTGCTTTGCGGCGACTTTCTACGTCCCTCGGTGGCGGCCGCGACGTACTCCTGACAAAGGAAGCAATCTGCACACTGTGTCTAAGCGCCGTGCGTGAGTGAGTGCCATTCACCGGCGCCGCTTTATGCGCTAATCTTCTTTATACCTCACTTGGCTGCGCCGC

>BPK206/0 clone 10|LinJ32_V3.2880|500 bp UPS + CDS + 500 bp DWS|arsenate reductase 2

AGTTGTAAAGAGCGACGGCACTGGCTGTGCGTGCAGCGCAGCGGTAGCTGACAACTGCCGTGCGTGTGGTGAAGGTGCAGAGGTCGAAGGAGGCGATGCGGCGTCGCCGTCGGCTTCTCCCACGTGTATTGTCGCTTCTACACGTTTTGTGACGGGCGCTGCCTGTGTGTGCGTGCGTGCGTGTGCGCTCGTCAGCTGCGTTGCAAAGACAGCGGCACCCAGGAGCAAGAGCGCGTCTTTGATGGCCGGAGGTGCACTTCAAGCTCATCTCCCTTGCTTCGTTTTTCGACCCCGCGCATGCAGAGACGCGCACACGACGAAGCCAAAAGGAAAAGACAGATAAACGGCGTCGAGGCAGCCTTCCAGGCCTGCTTTCATCGGCGAAGGACAAGGTAGCAGCGATGCTGCGTTGCACCTCTTCCTCTCTCTTCGGCGCTTGAGCCCTTGCTCATTTCCGCAGAAGAGGCAGCAGTAGCAGCCGCCATCATCCCTTCCGCGCAATGGCGAACTACACGTACATGAAGCCCGAAGAGCTGGTGGAGGTGCTCGACAATCCTGACTCACTCACCAAAGCCGCGGTGATAGACTGCAGGGATAGTGATCGAGACTGCGGCTTCATCGTGAACTCGATAAGCATGCCGACGATTAGCTGCACGGAAGAGATGTACGAAAAGCTCGCCAAGACGCTCTTCGAAGAGAAGAAAGAGATCGCTGTCTTTCACTGTGCCCAGTCGCTCATACGGGCCCCCAAGGGAGCCAACCGCTTCGTTCTGGCACAGAAGAAGCTCGGCTACGTGCTCCCTGCCGTGTACGTGCTGCGTGGTGGGTGGGAGGCGTTCTACCATATGTATGGCGACGTGCGGCCGGACCTCATTTATGTGTGAGCCATGAAGCAGCAAATGAGGCGGTGGMGGGCAAGACAGTGCTGCCGCTCCAGTGGCGCGCCAGCGCACCGCGACGGCCCATCCTCTGCCGAAAGTAGCGCGCACAGACGCAAAAAGGGGTGAGTGCGTGGCTGTTCTGTTCCCTTGCATGTGCGCTGCTCTAACTTTTACGTTGTGGGCCGCACGGAAAGGATGCACAGAAGCCCTTCCTCATGCGACAGGATTGCCATTTTGGCGCGCGTGCTTGCTTGCCACCTCACTGCCTTCTTTTTGGGTTTCTTTGTGGCTCTTACGGTGCCGCTTTTCGAACATGGTGACTGTCGTCGTGCACTCTAGTTTGCTTTGCGGCGACTTTCTACGTCCCTCGGTGGCGGCCGCGACGTACTCCTGACAAAGGAAGCAATCTGCACACTGTGTCTAAGCGCCGTGCGTGAGTGAGTGCCATTCACCGGCGCCGCTTTATGCGCTAATCTTCTTTATACCTCACTTGGCTGCGCCGC

>BPK275/0 clone 18|LinJ32_V3.2880|500 bp UPS + CDS + 500 bp DWS|arsenate reductase 2

AGTTGTAAAGAGCGACGGCACTGGCTGTGCGTGCAGCGCAGCGGTAGCTGACAACTGCCGTGCGTGTGGTGAAGGTGCAGAGGTCGAAGGAGGCGATGCGGCGTCGCCGTCGGCTTCTCCCACGTGTATTGTCGCTTCTACACGTTTTGTGACGGGCGCTGCCTGTGTGTGCGTGCGTGCGTGTGCGCTCGTCAGCTGCGTTGCAAAGACAGCGGCACCCAGGAGCAAGAGCGCGTCTTTGATGGCCGGAGGTGCACTTCAAGCTCATCTCCCTTGCTTCGTTTTTCGACCCCGCGCATGCAGAGACGCGCACACGACGAAGCCAAAAGGAAAAGACAGATAAACGGCGTCGAGGCAGCCTTCCAGGCCTGCTTTCATCGGCGAAGGACAAGGTAGCAGCGATGCTGCGTTGCACCTCTTCCTCTCTCTTCGGCGCTTGAGCCCTTGCTCATTTCCGCAGAAGAGGCAGCAGTAGCAGCCGCCATCATCCCTTCCGCGCAATGGCGAACTACACGTACATGAAGCCCGAAGAGCTGGTGGAGGTGCTCGACAATCCTGACTCACTCACCAAAGCCGCGGTGATAGACTGCAGGGATAGTGATCGAGACTGCGGCTTCATCGTGAACTCGATAAGCATGCCGACGATTAGCTGCACGGAAGAGATGTACGAAAAGCTCGCCAAGACGCTCTTCGAAGAGAAGAAAGAGATCGCTGTCTTTCACTGTGCCCAGTCGCTCATACGGGCCCCCAAGGGAGCCAACCGCTTCGTTCTGGCACAGAAGAAGCTCGGCTACGTGCTCCCTGCCGTGTACGTGCTGCGTGGTGGGTGGGAGGCGTTCTACCATATGTATGGCGACGTGCGGCCGGACCTCATTTATGTGTGAGCCATGAAGCAGCAAATGAGGCGGTGGTGGGCAAGACAGTGCTGCCGCTCCAGTGGCGCGCCAGCGCACCGCGACGGCCCATCCTCTGCCGAAAGTAGCGCGCACAGACGCAAAAAGGGGTGAGTGCGTGGCTGTTCTGTTCCCTTGCATGTGCGCTGCTCTAACTTTTACGTTGTGGGCCGCACGGAAAGGATGCACAGAAGCCCTTCCTCATGCGACAGGATTGCCATTTTGGCGCGCGTGCTTGCTTGCCACCTCACTGCCTTCTTTTTGGGTTTCTTTGTGGCTCTTACGGTGCCGCTTTTCGAACATGGTGACTGTCGTCGTGCACTCTAGTTTGCTTTGCGGCGACTTTCTACGTCCCTCGGTGGCGGCCGCGACGTACTCCTGACAAAGGAAGCAATCTGCACACTGTGTCTAAGCGCCGTGCGTGAGTGAGTGCCATTCACCGGCGCCGCTTTATGCGCTAATCTTCTTTATACCTCACTTGGCTGCGCCGC

>BPK282/0 clone 4|LinJ32_V3.2880|500 bp UPS + CDS + 500 bp DWS|arsenate reductase 2

AGTTGTAAAGAGCGACGGCACTGGCTGTGCGTGCAGCGCAGCGGTAGCTGACAACTGCCGTGCGTGTGGTGAAGGTGCAGAGGTCGAAGGAGGCGATGCGGCGTCGCCGTCGGCTTCTCCCACGTGTATTGTCGCTTCTACACGTTTTGTGACGGGCGCTGCCTGTGTGTGCGTGCGTGCGTGTGCGCTCGTCAGCTGCGTTGCAAAGACAGCGGCACCCAGGAGCAAGAGCGCGTCTTTGATGGCCGGAGGTGCACTTCAAGCTCATCTCCCTTGCTTCGTTTTTCGACCCCGCGCATGCAGAGACGCGCACACGACGAAGCCAAAAGGAAAAGACAGATAAACGGCGTCGAGGCAGCCTTCCAGGCCTGCTTTCATCGGCGAAGGACAAGGTAGCAGCGATGCTGCGTTGCACCTCTTCCTCTCTCTTCGGCGCTTGAGCCCTTGCTCATTTCCGCAGAAGAGGCAGCAGTAGCAGCCGCCATCATCCCTTCCGCGCAATGGCGAACTACACGTACATGAAGCCCGAAGAGCTGGTGGAGGTGCTCGACAATCCTGACTCACTCACCAAAGCCGCGGTGATAGACTGCAGGGATAGTGATCGAGACTGCGGCTTCATCGTGAACTCGATAAGCATGCCGACGATTAGCTGCACGGAAGAGATGTACGAAAAGCTCGCCAAGACGCTCTTCGAAGAGAAGAAAGAGATCGCTGTCTTTCACTGTGCCCAGTCGCTCATACGGGCCCCCAAGGGAGCCAACCGCTTCGTTCTGGCACAGAAGAAGCTCGGCTACGTGCTCCCTGCCGTGTACGTGCTGCGTGGTGGGTGGGAGGCGTTCTACCATATGTATGGCGACGTGCGGCCGGACCTCATTTATGTGTGAGCCATGAAGCAGCAAATGAGGCGGTGGTGGGCAAGACAGTGCTGCCGCTCCAGTGGCGCGCCAGCGCACCGCGACGGCCCATCCTCTGCCGAAAGTAGCGCGCACAGACGCAAAAAGGGGTGAGTGCGTGGCTGTTCTGTTCCCTTGCATGTGCGCTGCTCTAACTTTTACGTTGTGGGCCGCACGGAAAGGATGCACAGAAGCCCTTCCTCATGCGACAGGATTGCCATTTTGGCGCGCGTGCTTGCTTGCCACCTCACTGCCTTCTTTTTGGGTTTCTTTGTGGCTCTTACGGTGCCGCTTTTCGAACATGGTGACTGTCGTCGTGCACTCTAGTTTGCTTTGCGGCGACTTTCTACGTCCCTCGGTGGCGGCCGCGACGTACTCCTGACAAAGGAAGCAATCTGCACACTGTGTCTAAGCGCCGTGCGTGAGTGAGTGCCATTCACCGGCGCCGCTTTATGCGCTAATCTTCTTTATACCTCACTTGGCTGCGCCGC

>BPK294/0 clone 1|LinJ32_V3.2880|500 bp UPS + CDS + 500 bp DWS|arsenate reductase 2

AGTTGTAAAGAGCGACGGCACTGGCTGTGCGTGCAGCGCAGCGGTAGCTGACAACTGCCGTGCGTGTGGTGAAGGTGCAGAGGTCGAAGGAGGCGATGCGGCGTCGCCGTCGGCTTCTCCCACGTGTATTGTCGCTTCTACACGTTTTGTGACGGGCGCTGCCTGTGTGTGCGTGCGTGCGTGTGCGCTCGTCAGCTGCGTTGCAAAGACAGCGGCACCCAGGAGCAAGAGCGCGTCTTTGATGGCCGGAGGTGCACTTCAAGCTCATCTCCCTTGCTTCGTTTTTCGACCCCGCGCATGCAGAGACGCGCACACGACGAAGCCAAAAGGAAAAGACAGATAAACGGCGTCGAGGCAGCCTTCCAGGCCTGCTTTCATCGGCGAAGGACAAGGTAGCAGCGATGCTGCGTTGCACCTCTTCCTCTCTCTTCGGCGCTTGAGCCCTTGCTCATTTCCGCAGAAGAGGCAGCAGTAGCAGCCGCCATCATCCCTTCCGCGCAATGGCGAACTACACGTACATGAAGCCCGAAGAGCTGGTGGAGGTGCTCGACAATCCTGACTCACTCACCAAAGCCGCGGTGATAGACTGCAGGGATAGTGATCGAGACTGCGGCTTCATCGTGAACTCGATAAGCATGCCGACGATTAGCTGCACGGAAGAGATGTACGAAAAGCTCGCCAAGACGCTCTTCGAAGAGAAGAAAGAGATCGCTGTCTTTCACTGTGCCCAGTCGCTCATACGGGCCCCCAAGGGAGCCAACCGCTTCGTTCTGGCACAGAAGAAGCTCGGCTACGTGCTCCCTGCCGTGTACGTGCTGCGTGGTGGGTGGGAGGCGTTCTACCATATGTATGGCGACGTGCGGCCGGACCTCATTTATGTGTGAGCCATGAAGCAGCAAATGAGGCGGTGGMGGGCAAGACAGTGCTGCCGCTCCAGTGGCGCGCCAGCGCACCGCGACGGCCCATCCTCTGCCGAAAGTAGCGCGCACAGACGCAAAAAGGGGTGAGTGCGTGGCTGTTCTGTTCCCTTGCATGTGCGCTGCTCTAACTTTTACGTTGTGGGCCGCACGGAAAGGATGCACAGAAGCCCTTCCTCATGCGACAGGATTGCCATTTTGGCGCGCGTGCTTGCTTGCCACCTCACTGCCTTCTTTTTGGGTTTCTTTGTGGCTCTTACGGTGCCGCTTTTCGAACATGGTGACTGTCGTCGTGCACTCTAGTTTGCTTTGCGGCGACTTTCTACGTCCCTCGGTGGCGGCCGCGACGTACTCCTGACAAAGGAAGCAATCTGCACACTGTGTCTAAGCGCCGTGCGTGAGTGAGTGCCATTCACCGGCGCCGCTTTATGCGCTAATCTTCTTTATACCTCACTTGGCTGCGCCGC

>BPK298/0 clone 8|LinJ32_V3.2880|500 bp UPS + CDS + 500 bp DWS|arsenate reductase 2

AGTTGTAAAGAGCGACGGCACTGGCTGTGCGTGCAGCGCAGCGGTAGCTGACAACTGCCGTGCGTGTGGTGAAGGTGCAGAGGTCGAAGGAGGCGATGCGGCGTCGCCGTCGGCTTCTCCCACGTGTATTGTCGCTTCTACACGTTTTGTGACGGGCGCTGCCTGTGTGTGCGTGCGTGCGTGTGCGCTCGTCAGCTGCGTTGCAAAGACAGCGGCACCCAGGAGCAAGAGCGCGTCTTTGATGGCCGGAGGTGCACTTCAAGCTCATCTCCCTTGCTTCGTTTTTCGACCCCGCGCATGCAGAGACGCGCACACGACGAAGCCAAAAGGAAAAGACAGATAAACGGCGTCGAGGCAGCCTTCCAGGCCTGCTTTCATCGGCGAAGGACAAGGTAGCAGCGATGCTGCGTTGCACCTCTTCCTCTCTCTTCGGCGCTTGAGCCCTTGCTCATTTCCGCAGAAGAGGCAGCAGTAGCAGCCGCCATCATCCCTTCCGCGCAATGGCGAACTACACGTACATGAAGCCCGAAGAGCTGGTGGAGGTGCTCGACAATCCTGACTCACTCACCAAAGCCGCGGTGATAGACTGCAGGGATAGTGATCGAGACTGCGGCTTCATCGTGAACTCGATAAGCATGCCGACGATTAGCTGCACGGAAGAGATGTACGAAAAGCTCGCCAAGACGCTCTTCGAAGAGAAGAAAGAGATCGCTGTCTTTCACTGTGCCCAGTCGCTCATACGGGCCCCCAAGGGAGCCAACCGCTTCGTTCTGGCACAGAAGAAGCTCGGCTACGTGCTCCCTGCCGTGTACGTGCTGCGTGGTGGGTGGGAGGCGTTCTACCATATGTATGGCGACGTGCGGCCGGACCTCATTTATGTGTGAGCCATGAAGCAGCAAATGAGGCGGTGGTGGGCAAGACAGTGCTGCCGCTCCAGTGGCGCGCCAGCGCACCGCGACGGCCCATCCTCTGCCGAAAGTAGCGCGCACAGACGCAAAAAGGGGTGAGTGCGTGGCTGTTCTGTTCCCTTGCATGTGCGCTGCTCTAACTTTTACGTTGTGGGCCGCACGGAAAGGATGCACAGAAGCCCTTCCTCATGCGACAGGATTGCCATTTTGGCGCGCGTGCTTGCTTGCCACCTCACTGCCTTCTTTTTGGGTTTCTTTGTGGCTCTTACGGTGCCGCTTTTCGAACATGGTGACTGTCGTCGTGCACTCTAGTTTGCTTTGCGGCGACTTTCTACGTCCCTCGGTGGCGGCCGCGACGTACTCCTGACAAAGGAAGCAATCTGCACACTGTGTCTAAGCGCCGTGCGTGAGTGAGTGCCATTCACCGGCGCCGCTTTATGCGCTAATCTTCTTTATACCTCACTTGGCTGCGCCGC

### 6. AQUAGLYCEROPORIN 1

>BPK035/0 clone 1|LinJ31_V3.0030|500 bp UPS + CDS + 500 bp DWS|aquaglyceroporin 1

GACGTGCTTCGCTACCCCTGCTCTCTATGCTGGGTGTACAAACACCACAGCTCGTGCTGACTGACCATTTACTCTTTCCGATTTATTATTATTATTATTTTTCTCTGCTTTCCCTCTGCCTTTATGACGCGAGCCACCTCACCGTTGTATCAGGGTTCCGTGCCCACTCTGCGGGAACGTCGAAAGCCTGCACCCTGCCATCGCCTCGGCGGACAGGCTTGGGCTGACACTGTACCGGAGACACTCGCGGCTACGATGCTGTTTCTATCATCTCGCTGCCAGTCGTTTCGACGACTCAGCAAGCATGCGCTTCCAATCATTGCCCTTCTGCTTTTGCGGCGCTTCGGTAATACCGAGCGTGATTCTGGACCTGGCCTGCGCTGTATGCAGCGGAATCACGGTTTGCTGCACCACACGAAAGGGTGCTAGTCATCCCGTCTCGATACTGCCGTCGTGCAGCAGCCGTAGGTGACCTGCTATTTTAAGGGTCAGTTTGTTGAATGAACTCTCCTACAAGCACACCTCCCGCGTGCTACGATGCCGAGGTTCAGCTGTACATGGACAAGGAGGACCCGGAAGGGGTCCCCATTCAAAACCAAATGCACGAGGAAGAGCAGGGACAGCTCGAGGGTAAAAGGAACTTCACGTCGCAGAACAGGTGGCCCCTGTACAAATACCGATGGTGGCTACGCGAGTATGTTGCCGAGTTCTTCGGCACGTTTTTCCTCGTCACCTTTGGAACCGGCGTCATTGCTACCACTGTCTTTCACGCCGGTAACGCTGCAAGCTACCAGTCCAACTCCAGCTACATGGCCATCACCTTTGGCTGGGGGTTTGGGCTCACTATCGGCCTTTTCCTGAGCATGGCCGTGTCGGGTGGTCACCTGAACCCAGCTGTGACGCTGGCGAACTGTGTCTTTGGTGCCTTTCCCTGGATTAAGCTACCCGGCTATTTTCTCGCCCAATTTCTCGGAGGCTTGGTTGGTGCCGCCAACACCTACGGGCTCTTCAAATCGCACTTTGACGACGCCCAAAAGGCCTTGCTTCCGAACGAGACGATGGCGTCCAAGTACAGCGGAATCTTCGCCACATACCCTAATGTTGCAAACACCTACGCAGTGTGGAGTGAGGTGTTCAATACCATGGCGCTCATGATGGGCATTCTCGCCATCACGGATCCTCGCATGACTCCCGCCGTCAACTACAAGCCGGTGGCTATTGGACTACTGTTGTTTGTCATTGGCATCACGTCAGGCATCAACTCTTCCTATGGCCTCAACCCCGCACGCGACTTGTCACCTCGCATACTCTCGGCCATGCTCTGGGGCTCAGAGCCTTTCACGTTGTACAGCTACTACTTTTGGATACCTCTAGTCGCGCCGTTTGTTGGCGCCCTTCTCGGCATGTTCTTGTATGTCTTTTTCATCATTCCACCCAACTTCTAGCTTCCGCTGCATGGCGCCGGCGGCTTCGCCACGGGCCGGCACGCAACACCCAAGCCCGACACCCCCACCGGCGAGCACGCGCCATCGCAACAAGCAGGATAGCCCGCACGCTCATAACCCCCAAGGAACGCCTTTGGCCACCGCGGTCGCGCTCGCAACAAACGCCAGCGTCGAGACAACCGGTGCAACGCCTCCCTGCCCACCACTTCTGCGAGGTCATCTGAGAATACACTTCCCCATCCAGCGGGCCGAACGCATAAAGCGCTCGTTGCCCTTCCCACTTCATCATCTCGATATTCTTCACCACCGCGGCAACCTCCTGCCGCTAGCCTGTGCCCACGGATGCCTCTCCTTGACCGAAACTCTTCCCTTGATTATTCTCTCTTTCTTGCCCCGAAACAGGCCTCTTTCTACTCTTCTTCGCGGACTTCATCAACAGCACGCGCACTTCCCCACTTAACACTAAATTCGCGACACACACCCCTTTTTCAGTACATCCC

>BPK043/0 clone 2|LinJ31_V3.0030|500 bp UPS + CDS + 500 bp DWS|aquaglyceroporin 1

GACGTGCTTCGCTACCCCTGCTCTCTATGCTGGGTGTACAAACACCACAGCTCGTGCTGACTGACCATTTACTCTTTCCGATTTATTATTATTATTATTTTTCTCTGCTTTCCCTCTGCCTTTATGACGCGAGCCACCTCACCGTTGTATCAGGGTTCCGTGCCCACTCTGCGGGAACGTCGAAAGCCTGCACCCTGCCATCGCCTCGGCGGACAGGCTTGGGCTGACACTGTACCGGAGACACTCGCGGCTACGATGCTGTTTCTATCATCTCGCTGCCAGTCGTTTCGACGACTCAGCAAGCATGCGCTTCCAATCATTGCCCTTCTGCTTTTGCGGCGCTTCGGTAATACCGAGCGTGATTCTGGACCTGGCCTGCGCTGTATGCAGCGGAATCACGGTTTGCTGCACCACACGAAAGGGTGCTAGTCATCCCGTCTCGATACTGCCGTCGTGCAGCAGCCGTAGGTGACCTGCTATTTTAAGGGTCAGTTTGTTGAATGAACTCTCCTACAAGCACACCTCCCGCGTGCTACGATGCCGAGGTTCAGCTGTACATGGACAAGGAGGACCCGGAAGGGGTCCCCATTCAAAACCAAATGCACGAGGAAGAGCAGGGACAGCTCGAGGGTAAAAGGAACTTCACGTCGCAGAACAGGTGGCCCCTGTACAAATACCGATGGTGGCTACGCGAGTATGTTGCCGAGTTCTTCGGCACGTTTTTCCTCGTCACCTTTGGAACCGGCGTCATTGCTACCACTGTCTTTCACGCCGGTAACGCTGCAAGCTACCAGTCCAACTCCAGCTACATGGCCATCACCTTTGGCTGGGGGTTTGGGCTCACTATCGGCCTTTTCCTGAGCATGGCCGTGTCGGGTGGTCACCTGAACCCAGCTGTGACGCTGGCGAACTGTGTCTTTGGTGCCTTTCCCTGGATTAAGCTACCCGGCTATTTTCTCGCCCAATTTCTCGGAGGCTTGGTTGGTGCCGCCAACACCTACGGGCTCTTCAAATCGCACTTTGACGACGCCCAAAAGGCCTTGCTTCCGAACGAGACGATGGCGTCCAAGTACAGCGGAATCTTCGCCACATACCCTAATGTTGCAAACACCTACGCAGTGTGGAGTGAGGTGTTCAATACCATGGCGCTCATGATGGGCATTCTCGCCATCACGGATCCTCGCATGACTCCCGCCGTCAACTACAAGCCGGTGGCTATTGGACTACTGTTGTTTGTCATTGGCATCACGTCAGGCATCAACTCTTCCTATGGCCTCAACCCCGCACGCGACTTGTCACCTCGCATACTCTCGGCCATGCTCTGGGGCTCAGAGCCTTTCACGTTGTACAGCTACTACTTTTGGATACCTCTAGTCGCGCCGTTTGTTGGCGCCCTTCTCGGCATGTTCTTGTATGTCTTTTTCATCATTCCACCCAACTTCTAGCTTCCGCTGCATGGCGCCGGCGGCTTCGCCACGGGCCGGCACGCAACACCCAAGCCCGACACCCCCACCGGCGAGCACGCGCCATCGCAACAAGCAGGATAGCCCGCACGCTCATAACCCCCAAGGAACGCCTTTGGCCACCGCGGTCGCGCTCGCAACAAACGCCAGCGTCGAGACAACCGGTGCAACGCCTCCCTGCCCACCACTTCTGCGAGGTCATCTGAGAATACACTTCCCCATCCAGCGGGCCGAACGCATAAAGCGCTCGTTGCCCTTCCCACTTCATCATCTCGATATTCTTCACCACCGCGGCAACCTCCTGCCGCTAGCCTGTGCCCACGGATGCCTCTCCTTGACCGAAACTCTTCCCTTGATTATTCTCTCTTTCTTGCCCCGAAACAGGCCTCTTTCTACTCTTCTTCGCGGACTTCATCAACAGCACGCGCACTTCCCCACTTAACACTAAATTCGCGACACACACCCCTTTTTCAGTACATCCC

>BPK085/0 clone 8|LinJ31_V3.0030|500 bp UPS + CDS + 500 bp DWS|aquaglyceroporin 1

GACGTGCTTCGCTACCCCTGCTCTCTATGCTGGGTGTACAAACACCACAGCTCGTGCTGACTGACCATTTACTCTTTCCGATTTATTATTATTATTATTTTTCTCTGCTTTCCCTCTGCCTTTATGACGCGAGCCACCTCACCGTTGTATCAGGGTTCCGTGCCCACTCTGCGGGAACGTCGAAAGCCTGCACCCTGCCATCGCCTCGGCGGACAGGCTTGGGCTGACACTGTACCGGAGACACTCGCGGCTACGATGCTGTTTCTATCATCTCGCTGCCAGTCGTTTCGACGACTCAGCAAGCATGCGCTTCCAATCATTGCCCTTCTGCTTTTGCGGCGCTTCGGTAATACCGAGCGTGATTCTGGACCTGGCCTGCGCTGTATGCAGCGGAATCACGGTTTGCTGCACCACACGAAAGGGTGCTAGTCATCCCGTCTCGATACTGCCGTCGTGCAGCAGCCGTAGGTGACCTGCTATTTTAAGGGTCAGTTTGTTGAATGAACTCTCCTACAAGCACACCTCCCGCGTGCTACGATGCCGAGGTTCAGCTGTACATGGACAAGGAGGACCCGGAAGGGGTCCCCATTCAAAACCAAATGCACGAGGAAGAGCAGGGACAGCTCGAGGGTAAAAGGAACTTCACGTCGCAGAACAGGTGGCCCCTGTACAAATACCGATGGTGGCTACGCGAGTATGTTGCCGAGTTCTTCGGCACGTTTTTCCTCGTCACCTTTGGAACCGGCGTCATTGCTACCACTGTCTTTCACGCCGGTAACGCTGCAAGCTACCAGTCCAACTCCAGCTACATGGCCATCACCTTTGGCTGGGGGTTTGGGCTCACTATCGGCCTTTTCCTGAGCATGGCCGTGTCGGGTGGTCACCTGAACCCAGCTGTGACGCTGGCGAACTGTGTCTTTGGTGCCTTTCCCTGGATTAAGCTACCCGGCTATTTTCTCGCCCAATTTCTCGGAGGCTTGGTTGGTGCCGCCAACACCTACGGGCTCTTCAAATCGCACTTTGACGACGCCCAAAAGGCCTTGCTTCCGAACGAGACGATGGCGTCCAAGTACAGCGGAATCTTCGCCACATACCCTAATGTTGCAAACACCTACGCAGTGTGGAGTGAGGTGTTCAATACCATGGCGCTCATGATGGGCATTCTCGCCATCACGGATCCTCGCATGACTCCCGCCGTCAACTACAAGCCGGTGGCTATTGGACTACTGTTGTTTGTCATTGGCATCACGTCAGGCATCAACTCTTCCTATGGCCTCAACCCCGCACGCGACTTGTCACCTCGCATACTCTCGGCCATGCTCTGGGGCTCAGAGCCTTTCACGTTGTACAGCTACTACTTTTGGATACCTCTAGTCGCGCCGTTTGTTGGCGCCCTTCTCGGCATGTTCTTGTATGTCTTTTTCATCATTCCACCCAACTTCTAGCTTCCGCTGCATGGCGCCGGCGGCTTCGCCACGGGCCGGCACGCAACACCCAAGCCCGACACCCCCACCGGCGAGCACGCGCCATCGCAACAAGCAGGATAGCCCGCACGCTCATAACCCCCAAGGAACGCCTTTGGCCACCGCGGTCGCGCTCGCAACAAACGCCAGCGTCGAGACAACCGGTGCAACGCCTCCCTGCCCACCACTTCTGCGAGGTCATCTGAGAATACACTTCCCCATCCAGCGGGCCGAACGCATAAAGCGCTCGTTGCCCTTCCCACTTCATCATCTCGATATTCTTCACCACCGCGGCAACCTCCTGCCGCTAGCCTGTGCCCACGGATGCCTCTCCTTGACCGAAACTCTTCCCTTGATTATTCTCTCTTTCTTGCCCCGAAACAGGCCTCTTTCTACTCTTCTTCGCGGACTTCATCAACAGCACGCGCACTTCCCCACTTAACACTAAATTCGCGACACACACCCCTTTTTCAGTACATCCC

>BPK087/0 clone 11|LinJ31_V3.0030|500 bp UPS + CDS + 500 bp DWS|aquaglyceroporin 1

GACGTGCTTCGCTACCCCTGCTCTCTATGCTGGGTGTACAAACACCACAGCTCGTGCTGACTGACCATTTACTCTTTCCGATTTATTATTATTATTATTTTTCTCTGCTTTCCCTCTGCCTTTATGACGCGAGCCACCTCACCGTTGTATCAGGGTTCCGTGCCCACTCTGCGGGAACGTCGAAAGCCTGCACCCTGCCATCGCCTCGGCGGACAGGCTTGGGCTGACACTGTACCGGAGACACTCGCGGCTACGATGCTGTTTCTATCATCTCGCTGCCAGTCGTTTCGACGACTCAGCAAGCATGCGCTTCCAATCATTGCCCTTCTGCTTTTGCGGCGCTTCGGTAATACCGAGCGTGATTCTGGACCTGGCCTGCGCTGTATGCAGCGGAATCACGGTTTGCTGCACCACACGAAAGGGTGCTAGTCATCCCGTCTCGATACTGCCGTCGTGCAGCAGCCGTAGGTGACCTGCTATTTTAAGGGTCAGTTTGTTGAATGAACTCTCCTACAAGCACACCTCCCGCGTGCTACGATGCCGAGGTTCAGCTGTACATGGACAAGGAGGACCCGGAAGGGGTCCCCATTCAAAACCAAATGCACGAGGAAGAGCAGGGACAGCTCGAGGGTAAAAGGAACTTCACGTCGCAGAACAGGTGGCCCCTGTACAAATACCGATGGTGGCTACGCGAGTATGTTGCCGAGTTCTTCGGCACGTTTTTCCTCGTCACCTTTGGAACCGGCGTCATTGCTACCACTGTCTTTCACGCCGGTAACGCTGCAAGCTACCAGTCCAACTCCAGCTACATGGCCATCACCTTTGGCTGGGGGTTTGGGCTCACTATCGGCCTTTTCCTGAGCATGGCCGTGTCGGGTGGTCACCTGAACCCAGCTGTGACGCTGGCGAACTGTGTCTTTGGTGCCTTTCCCTGGATTAAGCTACCCGGCTATTTTCTCGCCCAATTTCTCGGAGGCTTGGTTGGTGCCGCCAACACCTACGGGCTCTTCAAATCGCACTTTGACGACGCCCAAAAGGCCTTGCTTCCGAACGAGACGATGGCGTCCAAGTACAGCGGAATCTTCGCCACATACCCTAATGTTGCAAACACCTACGCAGTGTGGAGTGAGGTGTTCAATACCATGGCGCTCATGATGGGCATTCTCGCCATCACGGATCCTCGCATGACTCCCGCCGTCAACTACAAGCCGGTGGCTATTGGACTACTGTTGTTTGTCATTGGCATCACGTCAGGCATCAACTCTTCCTATGGCCTCAACCCCGCACGCGACTTGTCACCTCGCATACTCTCGGCCATGCTCTGGGGCTCAGAGCCTTTCACGTTGTACAGCTACTACTTTTGGATACCTCTAGTCGCGCCGTTTGTTGGCGCCCTTCTCGGCATGTTCTTGTATGTCTTTTTCATCATTCCACCCAACTTCTAGCTTCCGCTGCATGGCGCCGGCGGCTTCGCCACGGGCCGGCACGCAACACCCAAGCCCGACACCCCCACCGGCGAGCACGCGCCATCGCAACAAGCAGGATAGCCCGCACGCTCATAACCCCCAAGGAACGCCTTTGGCCACCGCGGTCGCGCTCGCAACAAACGCCAGCGTCGAGACAACCGGTGCAACGCCTCCCTGCCCACCACTTCTGCGAGGTCATCTGAGAATACACTTCCCCATCCAGCGGGCCGAACGCATAAAGCGCTCGTTGCCCTTCCCACTTCATCATCTCGATATTCTTCACCACCGCGGCAACCTCCTGCCGCTAGCCTGTGCCCACGGATGCCTCTCCTTGACCGAAACTCTTCCCTTGATTATTCTCTCTTTCTTGCCCCGAAACAGGCCTCTTTCTACTCTTCTTCGCGGACTTCATCAACAGCACGCGCACTTCCCCACTTAACACTAAATTCGCGACACACACCCCTTTTTCAGTACATCCC

>BPK178/0 clone 3|LinJ31_V3.0030|500 bp UPS + CDS + 500 bp DWS|aquaglyceroporin 1

GACGTGCTTCGCTACCCCTGCTCTCTATGCTGGGTGTACAAACACCACAGCTCGTGCTGACTGACCATTTACTCTTTCCGATTTATTATTATTATTATTTTTCTCTGCTTTCCCTCTGCCTTTATGACGCGAGCCACCTCACCGTTGTATCAGGGTTCCGTGCCCACTCTGCGGGAACGTCGAAAGCCTGCACCCTGCCATCGCCTCGGCGGACAGGCTTGGGCTGACACTGTACCGGAGACACTCGCGGCTACGATGCTGTTTCTATCATCTCGCTGCCAGTCGTTTCGACGACTCAGCAAGCATGCGCTTCCAATCATTGCCCTTCTGCTTTTGCGGCGCTTCGGTAATACCGAGCGTGATTCTGGACCTGGCCTGCGCTGTATGCAGCGGAATCACGGTTTGCTGCACCACACGAAAGGGTGCTAGTCATCCCGTCTCGATACTGCCGTCGTGCAGCAGCCGTAGGTGACCTGCTATTTTAAGGGTCAGTTTGTTGAATGAACTCTCCTACAAGCACACCTCCCGCGTGCTACGATGCCGAGGTTCAGCTGTACATGGACAAGGAGGACCCGGAAGGGGTCCCCATTCAAAACCAAATGCACGAGGAAGAGCAGGGACAGCTCGAGGGTAAAAGGAACTTCACGTCGCAGAACAGGTGGCCCCTGTACAAATACCGATGGTGGCTACGCGAGTATGTTGCCGAGTTCTTCGGCACGTTTTTCCTCGTCACCTTTGGAACCGGCGTCATTGCTACCACTGTCTTTCACGCCGGTAACGCTGCAAGCTACCAGTCCAACTCCAGCTACATGGCCATCACCTTTGGCTGGGGGTTTGGGCTCACTATCGGCCTTTTCCTGAGCATGGCCGTGTCGGGTGGTCACCTGAACCCAGCTGTGACGCTGGCGAACTGTGTCTTTGGTGCCTTTCCCTGGATTAAGCTACCCGGCTATTTTCTCGCCCAATTTCTCGGAGGCTTGGTTGGTGCCGCCAACACCTACGGGCTCTTCAAATCGCACTTTGACGACGCCCAAAAGGCCTTGCTTCCGAACGAGACGATGGCGTCCAAGTACAGCGGAATCTTCGCCACATACCCTAATGTTGCAAACACCTACGCAGTGTGGAGTGAGGTGTTCAATACCATGGCGCTCATGATGGGCATTCTCGCCATCACGGATCCTCGCATGACTCCCGCCGTCAACTACAAGCCGGTGGCTATTGGACTACTGTTGTTTGTCATTGGCATCACGTCAGGCATCAACTCTTCCTATGGCCTCAACCCCGCACGCGACTTGTCACCTCGCATACTCTCGGCCATGCTCTGGGGCTCAGAGCCTTTCACGTTGTACAGCTACTACTTTTGGATACCTCTAGTCGCGCCGTTTGTTGGCGCCCTTCTCGGCATGTTCTTGTATGTCTTTTTCATCATTCCACCCAACTTCTAGCTTCCGCTGCATGGCGCCGGCGGCTTCGCCACGGGCCGGCACGCAACACCCAAGCCCGACACCCCCACCGGCGAGCACGCGCCATCGCAACAAGCAGGATAGCCCGCACGCTCATAACCCCCAAGGAACGCCTTTGGCCACCGCGGTCGCGCTCGCAACAAACGCCAGCGTCGAGACAACCGGTGCAACGCCTCCCTGCCCACCACTTCTGCGAGGTCATCTGAGAATACACTTCCCCATCCAGCGGGCCGAACGCATAAAGCGCTCGTTGCCCTTCCCACTTCATCATCTCGATATTCTTCACCACCGCGGCAACCTCCTGCCGCTAGCCTGTGCCCACGGATGCCTCTCCTTGACCGAAACTCTTCCCTTGATTATTCTCTCTTTCTTGCCCCGAAACAGGCCTCTTTCTACTCTTCTTCGCGGACTTCATCAACAGCACGCGCACTTCCCCACTTAACACTAAATTCGCGACACACACCCCTTTTTCAGTACATCCC

>BPK190/0 clone 3|LinJ31_V3.0030|500 bp UPS + CDS + 500 bp DWS|aquaglyceroporin 1

GACGTGCTTCGCTACCCCTGCTCTCTATGCTGGGTGTACAAACACCACAGCTCGTGCTGACTGACCATTTACTCTTTCCGATTTATTATTATTATTATTTTTCTCTGCTTTCCCTCTGCCTTTATGACGCGAGCCACCTCACCGTTGTATCAGGGTTCCGTGCCCACTCTGCGGGAACGTCGAAAGCCTGCACCCTGCCATCGCCTCGGCGGACAGGCTTGGGCTGACACTGTACCGGAGACACTCGCGGCTACGATGCTGTTTCTATCATCTCGCTGCCAGTCGTTTCGACGACTCAGCAAGCATGCGCTTCCAATCATTGCCCTTCTGCTTTTGCGGCGCTTCGGTAATACCGAGCGTGATTCTGGACCTGGCCTGCGCTGTATGCAGCGGAATCACGGTTTGCTGCACCACACGAAAGGGTGCTAGTCATCCCGTCTCGATACTGCCGTCGTGCAGCAGCCGTAGGTGACCTGCTATTTTAAGGGTCAGTTTGTTGAATGAACTCTCCTACAAGCACACCTCCCGCGTGCTACGATGCCGAGGTTCAGCTGTACATGGACAAGGAGGACCCGGAAGGGGTCCCCATTCAAAACCAAATGCACGAGGAAGAGCAGGGACAGCTCGAGGGTAAAAGGAACTTCACGTCGCAGAACAGGTGGCCCCTGTACAAATACCGATGGTGGCTACGCGAGTATGTTGCCGAGTTCTTCGGCACGTTTTTCCTCGTCACCTTTGGAACCGGCGTCATTGCTACCACTGTCTTTCACGCCGGTAACGCTGCAAGCTACCAGTCCAACTCCAGCTACATGGCCATCACCTTTGGCTGGGGGTTTGGGCTCACTATCGGCCTTTTCCTGAGCATGGCCGTGTCGGGTGGTCACCTGAACCCAGCTGTGACGCTGGCGAACTGTGTCTTTGGTGCCTTTCCCTGGATTAAGCTACCCGGCTATTTTCTCGCCCAATTTCTCGGAGGCTTGGTTGGTGCCGCCAACACCTACGGGCTCTTCAAATCGCACTTTGACGACGCCCAAAAGGCCTTGCTTCCGAACGAGACGATGGCGTCCAAGTACAGCGGAATCTTCGCCACATACCCTAATGTTGCAAACACCTACGCAGTGTGGAGTGAGGTGTTCAATACCATGGCGCTCATGATGGGCATTCTCGCCATCACGGATCCTCGCATGACTCCCGCCGTCAACTACAAGCCGGTGGCTATTGGACTACTGTTGTTTGTCATTGGCATCACGTCAGGCATCAACTCTTCCTATGGCCTCAACCCCGCACGCGACTTGTCACCTCGCATACTCTCGGCCATGCTCTGGGGCTCAGAGCCTTTCACGTTGTACAGCTACTACTTTTGGATACCTCTAGTCGCGCCGTTTGTTGGCGCCCTTCTCGGCATGTTCTTGTATGTCTTTTTCATCATTCCACCCAACTTCTAGCTTCCGCTGCATGGCGCCGGCGGCTTCGCCACGGGCCGGCACGCAACACCCAAGCCCGACACCCCCACCGGCGAGCACGCGCCATCGCAACAAGCAGGATAGCCCGCACGCTCATAACCCCCAAGGAACGCCTTTGGCCACCGCGGTCGCGCTCGCAACAAACGCCAGCGTCGAGACAACCGGTGCAACGCCTCCCTGCCCACCACTTCTGCGAGGTCATCTGAGAATACACTTCCCCATCCAGCGGGCCGAACGCATAAAGCGCTCGTTGCCCTTCCCACTTCATCATCTCGATATTCTTCACCACCGCGGCAACCTCCTGCCGCTAGCCTGTGCCCACGGATGCCTCTCCTTGACCGAAACTCTTCCCTTGATTATTCTCTCTTTCTTGCCCCGAAACAGGCCTCTTTCTACTCTTCTTCGCGGACTTCATCAACAGCACGCGCACTTCCCCACTTAACACTAAATTCGCGACACACACCCCTTTTTCAGTACATCCC

>BPK206/0 clone 10|LinJ31_V3.0030|500 bp UPS + CDS + 500 bp DWS|aquaglyceroporin 1

GACGTGCTTCGCTACCCCTGCTCTCTATGCTGGGTGTACAAACACCACAGCTCGTGCTGACTGACCATTTACTCTTTCCGATTTATTATTATTATTATTTTTCTCTGCTTTCCCTCTGCCTTTATGACGCGAGCCACCTCACCGTTGTATCAGGGTTCCGTGCCCACTCTGCGGGAACGTCGAAAGCCTGCACCCTGCCATCGCCTCGGCGGACAGGCTTGGGCTGACACTGTACCGGAGACACTCGCGGCTACGATGCTGTTTCTATCATCTCGCTGCCAGTCGTTTCGACGACTCAGCAAGCATGCGCTTCCAATCATTGCCCTTCTGCTTTTGCGGCGCTTCGGTAATACCGAGCGTGATTCTGGACCTGGCCTGCGCTGTATGCAGCGGAATCACGGTTTGCTGCACCACACGAAAGGGTGCTAGTCATCCCGTCTCGATACTGCCGTCGTGCAGCAGCCGTAGGTGACCTGCTATTTTAAGGGTCAGTTTGTTGAATGAACTCTCCTACAAGCACACCTCCCGCGTGCTACGATGCCGAGGTTCAGCTGTACATGGACAAGGAGGACCCGGAAGGGGTCCCCATTCAAAACCAAATGCACGAGGAAGAGCAGGGACAGCTCGAGGGTAAAAGGAACTTCACGTCGCAGAACAGGTGGCCCCTGTACAAATACCGATGGTGGCTACGCGAGTATGTTGCCGAGTTCTTCGGCACGTTTTTCCTCGTCACCTTTGGAACCGGCGTCATTGCTACCACTGTCTTTCACGCCGGTAACGCTGCAAGCTACCAGTCCAACTCCAGCTACATGGCCATCACCTTTGGCTGGGGGTTTGGGCTCACTATCGGCCTTTTCCTGAGCATGGCCGTGTCGGGTGGTCACCTGAACCCAGCTGTGACGCTGGCGAACTGTGTCTTTGGTGCCTTTCCCTGGATTAAGCTACCCGGCTATTTTCTCGCCCAATTTCTCGGAGGCTTGGTTGGTGCCGCCAACACCTACGGGCTCTTCAAATCGCACTTTGACGACGCCCAAAAGGCCTTGCTTCCGAACGAGACGATGGCGTCCAAGTACAGCGGAATCTTCGCCACATACCCTAATGTTGCAAACACCTACGCAGTGTGGAGTGAGGTGTTCAATACCATGGCGCTCATGATGGGCATTCTCGCCATCACGGATCCTCGCATGACTCCCGCCGTCAACTACAAGCCGGTGGCTATTGGACTACTGTTGTTTGTCATTGGCATCACGTCAGGCATCAACTCTTCCTATGGCCTCAACCCCGCACGCGACTTGTCACCTCGCATACTCTCGGCCATGCTCTGGGGCTCAGAGCCTTTCACGTTGTACAGCTACTACTTTTGGATACCTCTAGTCGCGCCGTTTGTTGGCGCCCTTCTCGGCATGTTCTTGTATGTCTTTTTCATCATTCCACCCAACTTCTAGCTTCCGCTGCATGGCGCCGGCGGCTTCGCCACGGGCCGGCACGCAACACCCAAGCCCGACACCCCCACCGGCGAGCACGCGCCATCGCAACAAGCAGGATAGCCCGCACGCTCATAACCCCCAAGGAACGCCTTTGGCCACCGCGGTCGCGCTCGCAACAAACGCCAGCGTCGAGACAACCGGTGCAACGCCTCCCTGCCCACCACTTCTGCGAGGTCATCTGAGAATACACTTCCCCATCCAGCGGGCCGAACGCATAAAGCGCTCGTTGCCCTTCCCACTTCATCATCTCGATATTCTTCACCACCGCGGCAACCTCCTGCCGCTAGCCTGTGCCCACGGATGCCTCTCCTTGACCGAAACTCTTCCCTTGATTATTCTCTCTTTCTTGCCCCGAAACAGGCCTCTTTCTACTCTTCTTCGCGGACTTCATCAACAGCACGCGCACTTCCCCACTTAACACTAAATTCGCGACACACACCCCTTTTTCAGTACATCCC

>BPK275/0 clone 18|LinJ31_V3.0030|500 bp UPS + CDS + 500 bp DWS|aquaglyceroporin 1

GACGTGCTTCGCTACCCCTGCTCTCTATGCTGGGTGTACAAACACCACAGCTCGTGCTGACTGACCATTTACTCTTTCCGATTTATTATTATTATTATTTTTCTCTGCTTTCCCTCTGCCTTTATGACGCGAGCCACCTCACCGTTGTATCAGGGTTCCGTGCCCACTCTGCGGGAACGTCGAAAGCCTGCACCCTGCCATCGCCTCGGCGGACAGGCTTGGGCTGACACTGTACCGGAGACACTCGCGGCTACGATGCTGTTTCTATCATCTCGCTGCCAGTCGTTTCGACGACTCAGCAAGCATGCGCTTCCAATCATTGCCCTTCTGCTTTTGCGGCGCTTCGGTAATACCGAGCGTGATTCTGGACCTGGCCTGCGCTGTATGCAGCGGAATCACGGTTTGCTGCACCACACGAAAGGGTGCTAGTCATCCCGTCTCGATACTGCCGTCGTGCAGCAGCCGTAGGTGACCTGCTATTTTAAGGGTCAGTTTGTTGAATGAACTCTCCTACAAGCACACCTCCCGCGTGCTACGATGCCGAGGTTCAGCTGTACATGGACAAGGAGGACCCGGAAGGGGTCCCCATTCAAAACCAAATGCACGAGGAAGAGCAGGGACAGCTCGAGGGTAAAAGGAACTTCACGTCGCAGAACAGGTGGCCCCTGTACAAATACCGATGGTGGCTACGCGAGTATGTTGCCGAGTTCTTCGGCACGTTTTTCCTCGTCACCTTTGGAACCGGCGTCATTGCTACCACTGTCTTTCACGCCGGTAACGCTGCAAGCTACCAGTCCAACTCCAGCTACATGGCCATCACCTTTGGCTGGGGGTTTGGGCTCACTATCGGCCTTTTCCTGAGCATGGCCGTGTCGGGTGGTCACCTGAACCCAGCTGTGACGCTGGCGAACTGTGTCTTTGGTGCCTTTCCCTGGATTAAGCTACCCGGCTATTTTCTCGCCCAATTTCTCGGAGGCTTGGTTGGTGCCGCCAACACCTACGGGCTCTTCAAATCGCACTTTGACGACGCCCAAAAGGCCTTGCTTCCGAACGAGACGATGGCGTCCAAGTACAGCGGAATCTTCGCCACATACCCTAATGTTGCAAACACCTACGCAGTGTGGAGTGAGGTGTTCAATACCATGGCGCTCATGATGGGCATTCTCGCCATCACGGATCCTCGCATGACTCCCGCCGTCAACTACAAGCCGGTGGCTATTGGACTACTGTTGTTTGTCATTGGCATCACGTCAGGCATCAACTCTTCCTATGGCCTCAACCCCGCACGCGACTTGTCACCTCGCATACTCTCGGCCATGCTCTGGGGCTCAGAGCCTTTCACGTTGTACAGCTACTACTTTTGGATACCTCTAGTCGCGCCGTTTGTTGGCGCCCTTCTCGGCATGTTCTTGTATGTCTTTTTCATCATTCCACCCAACTTCTAGCTTCCGCTGCATGGCGCCGGCGGCTTCGCCACGGGCCGGCACGCAACACCCAAGCCCGACACCCCCACCGGCGAGCACGCGCCATCGCAACAAGCAGGATAGCCCGCACGCTCATAACCCCCAAGGAACGCCTTTGGCCACCGCGGTCGCGCTCGCAACAAACGCCAGCGTCGAGACAACCGGTGCAACGCCTCCCTGCCCACCACTTCTGCGAGGTCATCTGAGAATACACTTCCCCATCCAGCGGGCCGAACGCATAAAGCGCTCGTTGCCCTTCCCACTTCATCATCTCGATATTCTTCACCACCGCGGCAACCTCCTGCCGCTAGCCTGTGCCCACGGATGCCTCTCCTTGACCGAAACTCTTCCCTTGATTATTCTCTCTTTCTTGCCCCGAAACAGGCCTCTTTCTACTCTTCTTCGCGGACTTCATCAACAGCACGCGCACTTCCCCACTTAACACTAAATTCGCGACACACACCCCTTTTTCAGTACATCCC

>BPK282/0 clone 4|LinJ31_V3.0030|500 bp UPS + CDS + 500 bp DWS|aquaglyceroporin 1

GACGTGCTTCGCTACCCCTGCTCTCTATGCTGGGTGTACAAACACCACAGCTCGTGCTGACTGACCATTTACTCTTTCCGATTTATTATTATTATTATTTTTCTCTGCTTTCCCTCTGCCTTTATGACGCGAGCCACCTCACCGTTGTATCAGGGTTCCGTGCCCACTCTGCGGGAACGTCGAAAGCCTGCACCCTGCCATCGCCTCGGCGGACAGGCTTGGGCTGACACTGTACCGGAGACACTCGCGGCTACGATGCTGTTTCTATCATCTCGCTGCCAGTCGTTTCGACGACTCAGCAAGCATGCGCTTCCAATCATTGCCCTTCTGCTTTTGCGGCGCTTCGGTAATACCGAGCGTGATTCTGGACCTGGCCTGCGCTGTATGCAGCGGAATCACGGTTTGCTGCACCACACGAAAGGGTGCTAGTCATCCCGTCTCGATACTGCCGTCGTGCAGCAGCCGTAGGTGACCTGCTATTTTAAGGGTCAGTTTGTTGAATGAACTCTCCTACAAGCACACCTCCCGCGTGCTACGATGCCGAGGTTCAGCTGTACATGGACAAGGAGGACCCGGAAGGGGTCCCCATTCAAAACCAAATGCACGAGGAAGAGCAGGGACAGCTCGAGGGTAAAAGGAACTTCACGTCGCAGAACAGGTGGCCCCTGTACAAATACCGATGGTGGCTACGCGAGTATGTTGCCGAGTTCTTCGGCACGTTTTTCCTCGTCACCTTTGGAACCGGCGTCATTGCTACCACTGTCTTTCACGCCGGTAACGCTGCAAGCTACCAGTCCAACTCCAGCTACATGGCCATCACCTTTGGCTGGGGGTTTGGGCTCACTATCGGCCTTTTCCTGAGCATGGCCGTGTCGGGTGGTCACCTGAACCCAGCTGTGACGCTGGCGAACTGTGTCTTTGGTGCCTTTCCCTGGATTAAGCTACCCGGCTATTTTCTCGCCCAATTTCTCGGAGGCTTGGTTGGTGCCGCCAACACCTACGGGCTCTTCAAATCGCACTTTGACGACGCCCAAAAGGCCTTGCTTCCGAACGAGACGATGGCGTCCAAGTACAGCGGAATCTTCGCCACATACCCTAATGTTGCAAACACCTACGCAGTGTGGAGTGAGGTGTTCAATACCATGGCGCTCATGATGGGCATTCTCGCCATCACGGATCCTCGCATGACTCCCGCCGTCAACTACAAGCCGGTGGCTATTGGACTACTGTTGTTTGTCATTGGCATCACGTCAGGCATCAACTCTTCCTATGGCCTCAACCCCGCACGCGACTTGTCACCTCGCATACTCTCGGCCATGCTCTGGGGCTCAGAGCCTTTCACGTTGTACAGCTACTACTTTTGGATACCTCTAGTCGCGCCGTTTGTTGGCGCCCTTCTCGGCATGTTCTTGTATGTCTTTTTCATCATTCCACCCAACTTCTAGCTTCCGCTGCATGGCGCCGGCGGCTTCGCCACGGGCCGGCACGCAACACCCAAGCCCGACACCCCCACCGGCGAGCACGCGCCATCGCAACAAGCAGGATAGCCCGCACGCTCATAACCCCCAAGGAACGCCTTTGGCCACCGCGGTCGCGCTCGCAACAAACGCCAGCGTCGAGACAACCGGTGCAACGCCTCCCTGCCCACCACTTCTGCGAGGTCATCTGAGAATACACTTCCCCATCCAGCGGGCCGAACGCATAAAGCGCTCGTTGCCCTTCCCACTTCATCATCTCGATATTCTTCACCACCGCGGCAACCTCCTGCCGCTAGCCTGTGCCCACGGATGCCTCTCCTTGACCGAAACTCTTCCCTTGATTATTCTCTCTTTCTTGCCCCGAAACAGGCCTCTTTCTACTCTTCTTCGCGGACTTCATCAACAGCACGCGCACTTCCCCACTTAACACTAAATTCGCGACACACACCCCTTTTTCAGTACATCCC

>BPK294/0 clone 1|LinJ31_V3.0030|500 bp UPS + CDS + 500 bp DWS|aquaglyceroporin 1

GACGTGCTTCGCTACCCCTGCTCTCTATGCTGGGTGTACAAACACCACAGCTCGTGCTGACTGACCATTTACTCTTTCCGATTTATTATTATTATTATTTTTCTCTGCTTTCCCTCTGCCTTTATGACGCGAGCCACCTCACCGTTGTATCAGGGTTCCGTGCCCACTCTGCGGGAACGTCGAAAGCCTGCACCCTGCCATCGCCTCGGCGGACAGGCTTGGGCTGACACTGTACCGGAGACACTCGCGGCTACGATGCTGTTTCTATCATCTCGCTGCCAGTCGTTTCGACGACTCAGCAAGCATGCGCTTCCAATCATTGCCCTTCTGCTTTTGCGGCGCTTCGGTAATACCGAGCGTGATTCTGGACCTGGCCTGCGCTGTATGCAGCGGAATCACGGTTTGCTGCACCACACGAAAGGGTGCTAGTCATCCCGTCTCGATACTGCCGTCGTGCAGCAGCCGTAGGTGACCTGCTATTTTAAGGGTCAGTTTGTTGAATGAACTCTCCTACAAGCACACCTCCCGCGTGCTACGATGCCGAGGTTCAGCTGTACATGGACAAGGAGGACCCGGAAGGGGTCCCCATTCAAAACCAAATGCACGAGGAAGAGCAGGGACAGCTCGAGGGTAAAAGGAACTTCACGTCGCAGAACAGGTGGCCCCTGTACAAATACCGATGGTGGCTACGCGAGTATGTTGCCGAGTTCTTCGGCACGTTTTTCCTCGTCACCTTTGGAACCGGCGTCATTGCTACCACTGTCTTTCACGCCGGTAACGCTGCAAGCTACCAGTCCAACTCCAGCTACATGGCCATCACCTTTGGCTGGGGGTTTGGGCTCACTATCGGCCTTTTCCTGAGCATGGCCGTGTCGGGTGGTCACCTGAACCCAGCTGTGACGCTGGCGAACTGTGTCTTTGGTGCCTTTCCCTGGATTAAGCTACCCGGCTATTTTCTCGCCCAATTTCTCGGAGGCTTGGTTGGTGCCGCCAACACCTACGGGCTCTTCAAATCGCACTTTGACGACGCCCAAAAGGCCTTGCTTCCGAACGAGACGATGGCGTCCAAGTACAGCGGAATCTTCGCCACATACCCTAATGTTGCAAACACCTACGCAGTGTGGAGTGAGGTGTTCAATACCATGGCGCTCATGATGGGCATTCTCGCCATCACGGATCCTCGCATGACTCCCGCCGTCAACTACAAGCCGGTGGCTATTGGACTACTGTTGTTTGTCATTGGCATCACGTCAGGCATCAACTCTTCCTATGGCCTCAACCCCGCACGCGACTTGTCACCTCGCATACTCTCGGCCATGCTCTGGGGCTCAGAGCCTTTCACGTTGTACAGCTACTACTTTTGGATACCTCTAGTCGCGCCGTTTGTTGGCGCCCTTCTCGGCATGTTCTTGTATGTCTTTTTCATCATTCCACCCAACTTCTAGCTTCCGCTGCATGGCGCCGGCGGCTTCGCCACGGGCCGGCACGCAACACCCAAGCCCGACACCCCCACCGGCGAGCACGCGCCATCGCAACAAGCAGGATAGCCCGCACGCTCATAACCCCCAAGGAACGCCTTTGGCCACCGCGGTCGCGCTCGCAACAAACGCCAGCGTCGAGACAACCGGTGCAACGCCTCCCTGCCCACCACTTCTGCGAGGTCATCTGAGAATACACTTCCCCATCCAGCGGGCCGAACGCATAAAGCGCTCGTTGCCCTTCCCACTTCATCATCTCGATATTCTTCACCACCGCGGCAACCTCCTGCCGCTAGCCTGTGCCCACGGATGCCTCTCCTTGACCGAAACTCTTCCCTTGATTATTCTCTCTTTCTTGCCCCGAAACAGGCCTCTTTCTACTCTTCTTCGCGGACTTCATCAACAGCACGCGCACTTCCCCACTTAACACTAAATTCGCGACACACACCCCTTTTTCAGTACATCCC

>BPK298/0 clone 8|LinJ31_V3.0030|500 bp UPS + CDS + 500 bp DWS|aquaglyceroporin 1

GACGTGCTTCGCTACCCCTGCTCTCTATGCTGGGTGTACAAACACCACAGCTCGTGCTGACTGACCATTTACTCTTTCCGATTTATTATTATTATTATTTTTCTCTGCTTTCCCTCTGCCTTTATGACGCGAGCCACCTCACCGTTGTATCAGGGTTCCGTGCCCACTCTGCGGGAACGTCGAAAGCCTGCACCCTGCCATCGCCTCGGCGGACAGGCTTGGGCTGACACTGTACCGGAGACACTCGCGGCTACGATGCTGTTTCTATCATCTCGCTGCCAGTCGTTTCGACGACTCAGCAAGCATGCGCTTCCAATCATTGCCCTTCTGCTTTTGCGGCGCTTCGGTAATACCGAGCGTGATTCTGGACCTGGCCTGCGCTGTATGCAGCGGAATCACGGTTTGCTGCACCACACGAAAGGGTGCTAGTCATCCCGTCTCGATACTGCCGTCGTGCAGCAGCCGTAGGTGACCTGCTATTTTAAGGGTCAGTTTGTTGAATGAACTCTCCTACAAGCACACCTCCCGCGTGCTACGATGCCGAGGTTCAGCTGTACATGGACAAGGAGGACCCGGAAGGGGTCCCCATTCAAAACCAAATGCACGAGGAAGAGCAGGGACAGCTCGAGGGTAAAAGGAACTTCACGTCGCAGAACAGGTGGCCCCTGTACAAATACCGATGGTGGCTACGCGAGTATGTTGCCGAGTTCTTCGGCACGTTTTTCCTCGTCACCTTTGGAACCGGCGTCATTGCTACCACTGTCTTTCACGCCGGTAACGCTGCAAGCTACCAGTCCAACTCCAGCTACATGGCCATCACCTTTGGCTGGGGGTTTGGGCTCACTATCGGCCTTTTCCTGAGCATGGCCGTGTCGGGTGGTCACCTGAACCCAGCTGTGACGCTGGCGAACTGTGTCTTTGGTGCCTTTCCCTGGATTAAGCTACCCGGCTATTTTCTCGCCCAATTTCTCGGAGGCTTGGTTGGTGCCGCCAACACCTACGGGCTCTTCAAATCGCACTTTGACGACGCCCAAAAGGCCTTGCTTCCGAACGAGACGATGGCGTCCAAGTACAGCGGAATCTTCGCCACATACCCTAATGTTGCAAACACCTACGCAGTGTGGAGTGAGGTGTTCAATACCATGGCGCTCATGATGGGCATTCTCGCCATCACGGATCCTCGCATGACTCCCGCCGTCAACTACAAGCCGGTGGCTATTGGACTACTGTTGTTTGTCATTGGCATCACGTCAGGCATCAACTCTTCCTATGGCCTCAACCCCGCACGCGACTTGTCACCTCGCATACTCTCGGCCATGCTCTGGGGCTCAGAGCCTTTCACGTTGTACAGCTACTACTTTTGGATACCTCTAGTCGCGCCGTTTGTTGGCGCCCTTCTCGGCATGTTCTTGTATGTCTTTTTCATCATTCCACCCAACTTCTAGCTTCCGCTGCATGGCGCCGGCGGCTTCGCCACGGGCCGGCACGCAACACCCAAGCCCGACACCCCCACCGGCGAGCACGCGCCATCGCAACAAGCAGGATAGCCCGCACGCTCATAACCCCCAAGGAACGCCTTTGGCCACCGCGGTCGCGCTCGCAACAAACGCCAGCGTCGAGACAACCGGTGCAACGCCTCCCTGCCCACCACTTCTGCGAGGTCATCTGAGAATACACTTCCCCATCCAGCGGGCCGAACGCATAAAGCGCTCGTTGCCCTTCCCACTTCATCATCTCGATATTCTTCACCACCGCGGCAACCTCCTGCCGCTAGCCTGTGCCCACGGATGCCTCTCCTTGACCGAAACTCTTCCCTTGATTATTCTCTCTTTCTTGCCCCGAAACAGGCCTCTTTCTACTCTTCTTCGCGGACTTCATCAACAGCACGCGCACTTCCCCACTTAACACTAAATTCGCGACACACACCCCTTTTTCAGTACATCCC

### 7. CYSTEINE SYNTHASE

>BPK035/0 clone 1|LinJ36_V3.3750|500 bp UPS + CDS + 500 bp DWS|cysteine synthase

GTCGATGCCTCGGAGCTGCAGGACTGAGCTACCATCGGCAGCGCCGTCACTGCAGCCTTCGTGTGGCGCGAATGTGGAGAAGGAAAGGGCCAACGCTGGAGATCCACGCCTGCATAAGCAGGAGGGATGGAGACAGAAGATACGGTGGCAGTGGGAATACGGTAGCGGAGCGCAACGCATTTCTTGATGCCTTTCAAGCCGCACACCGCCGCTGCCTTCTTTGCAGCTCCAGTATCTGTTTGAAGCGCACGTGCAGCAAGCAGAGTGGTGGGAGCGGTGACCATCGCCGGTGATCAGGAGGAGGATACCTCGCTTTCTCCTTCATTCTCTTGTCCGCCTCTTCCTTTTTTTTTTCGGTCGTGCTCCTCTGCTACCACCTCAGTCTTACTGCTTAGCGTGTGTGACGGCCACCATTTCGCGAAGGGTATATTTTATGTGTGCTCGTGGTGGTGCAATGAGGAGGTCTTGCCATCCCTCTTCTCTTCCTCTTGCTTTCGTGAATGGCGGCACCGTTCGACAAGTCAAAAAATGTGGCGCAGTCTATCGATCAGCTGATTGGCCAGACGCCGGCGTTGTACCTAAACAAGCTGAACAATACCAAGGCAAAGGTTGTGCTCAAGATGGAGTGCGAAAACCCGATGGCGTCCGTGAAGGATCGCCTTGGCTTCGCCATCTACGATAAAGCGGAGAAGGAGGGCAAGCTGATCCCTGGCAAGTCTATCGTGGTCGAGTCGTCCAGTGGAAACACGGGCGTGTCGCTAGCGCACTTGGGTGCGATTCGTGGCTACAAGGTCATCATTACGATGCCCGAATCCATGTCTCTCGAGCGCCGCTGCCTGCTGCGTATCTTCGGCGCCGAGGTAATCCTCACCCCTGCCGCTCTTGGCATGAAGGGTGCCGTGGCCATGGCCAAGAAAATCGTCGCCGCTAACCCCAACGCCGTCTTGGCGGATCAGTTCGCAACCAAGTACAACGCCCTCATACACGAGGAAACCACGGGGCCTGAGATTTGGGAGCAGACGAACCACAATGTCGACTGCTTCATAGCCGGCGTTGGAACAGGTGGCACGCTGACAGGTGTGGCGCGGGCGCTAAAGAAGATGGGCAGTCATGCCCGCATCGTTGCCGTGGAGCCGACGGAGTCACCTGTGCTGTCGGGTGGAAAACCAGGCCCGCACAAAATTCAGGGCATCGGCCCGGGCTTTGTGCCAGACGTGCTCGACCGCAGTCTCATCGACGAGGTGCTTTGCGTGGCAGGTGATGATGCCATTGAGACGGCACTGAAGCTCACGCGCAGCGACGGGGTCTTCTGCGGCTTCTCTGGTGGCGCCAACGTTTACGCGGCGCTGAAGATCGCGGAGCGTCCGGAGATGGAGGGCAAAACAATTGTGACGGTCATTCCGTCCTTCGGTGAGCGCTACCTCTCCACCACGCTGTACAGGAGCGTTCGGGATGAGGTGTCGTCCCTGCCCGTAGGTGCCGATTTCTCTTCTCCCTCTCCCCCCCGTCAGCTTTACACTGGCGGATGGGTCGCGTGAGAGGTAGGAGAGGCGGATGCCACGCTGCATCGAGGCTGTTCCTCTACAATGCCGCCCACACAATCTCTTTCCAATCATCCTTGAAGTGTGTCCAGCTTCTCCCTTGCCTGCGCATCAAGCCCTGCCGTCACCGTGGCCATGAACCGCTTTCCTCATGTCCTCGAGGTTCCCTGACTGACTTTATCCTGTTTTGTGTCTCCCCTTTGTCGTCTCCGCGCATGGCGCTTTCTTCTCCGCACTCCCCCCCCCCTTTAAGTGATCCCATCACGCATGGAACATTCGAATCAACCACACGCGTTCGCATACAACCACCTTTCTCTGCTCCTCTATTCACGTGATAGCTGTATACCCCCTCTCCCCGACACACACACACACACACACGCACGCGCGCACACGGACACAGACACAGGGTCGCTCGAAGAAGAGGAAAACAAAGGA

>BPK043/0 clone 2|LinJ36_V3.3750|500 bp UPS + CDS + 500 bp DWS|cysteine synthase

GTCGATGCCTCGGAGCTGCAGGACTGAGCTACCATCGGCAGCGCCGTCACTGCAGCCTTCGTGTGGCGCGAATGTGGAGAAGGAAAGGGCCAACGCTGGAGATCCACGCCTGCATAAGCAGGAGGGATGGAGACAGAAGATACGGTGGCAGTGGGAATACGGTAGCGGAGCGCAACGCATTTCTTGATGCCTTTCAAGCCGCACACCGCCGCTGCCTTCTTTGCAGCTCCAGTATCTGTTTGAAGCGCACGTGCAGCAAGCAGAGTGGTGGGAGCGGTGACCATCGCCGGTGATCAGGAGGAGGATACCTCGCTTTCTCCTTCATTCTCTTGTCCGCCTCTTCCTTTTTTTTTTCGGTCGTGCTCCTCTGCTACCACCTCAGTCTTACTGCTTAGCGTGTGTGACGGCCACCATTTCGCGAAGGGTATATTTTATGTGTGCTCGTGGTGGTGCAATGAGGAGGTCTTGCCATCCCTCTTCTCTTCCTCTTGCTTTCGTGAATGGCGGCACCGTTCGACAAGTCAAAAAATGTGGCGCAGTCTATCGATCAGCTGATTGGCCAGACGCCGGCGTTGTACCTAAACAAGCTGAACAATACCAAGGCAAAGGTTGTGCTCAAGATGGAGTGCGAAAACCCGATGGCGTCCGTGAAGGATCGCCTTGGCTTCGCCATCTACGATAAAGCGGAGAAGGAGGGCAAGCTGATCCCTGGCAAGTCTATCGTGGTCGAGTCGTCCAGTGGAAACACGGGCGTGTCGCTAGCGCACTTGGGTGCGATTCGTGGCTACAAGGTCATCATTACGATGCCCGAATCCATGTCTCTCGAGCGCCGCTGCCTGCTGCGTATCTTCGGCGCCGAGGTAATCCTCACCCCTGCCGCTCTTGGCATGAAGGGTGCCGTGGCCATGGCCAAGAAAATCGTCGCCGCTAACCCCAACGCCGTCTTGGCGGATCAGTTCGCAACCAAGTACAACGCCCTCATACACGAGGAAACCACGGGGCCTGAGATTTGGGAGCAGACGAACCACAATGTCGACTGCTTCATAGCCGGCGTTGGAACAGGTGGCACGCTGACAGGTGTGGCGCGGGCGCTAAAGAAGATGGGCAGTCATGCCCGCATCGTTGCCGTGGAGCCGACGGAGTCACCTGTGCTGTCGGGTGGAAAACCAGGCCCGCACAAAATTCAGGGCATCGGCCCGGGCTTTGTGCCAGACGTGCTCGACCGCAGTCTCATCGACGAGGTGCTTTGCGTGGCAGGTGATGATGCCATTGAGACGGCACTGAAGCTCACGCGCAGCGACGGGGTCTTCTGCGGCTTCTCTGGTGGCGCCAACGTTTACGCGGCGCTGAAGATCGCGGAGCGTCCGGAGATGGAGGGCAAAACAATTGTGACGGTCATTCCGTCCTTCGGTGAGCGCTACCTCTCCACCACGCTGTACAGGAGCGTTCGGGATGAGGTGTCGTCCCTGCCCGTAGGTGCCGATTTCTCTTCTCCCTCTCCCCCCCGTCAGCTTTACACTGGCGGATGGGTCGCGTGAGAGGTAGGAGAGGCGGATGCCACGCTGCATCGAGGCTGTTCCTCTACAATGCCGCCCACACAATCTCTTTCCAATCATCCTTGAAGTGTGTCCAGCTTCTCCCTTGCCTGCGCATCAAGCCCTGCCGTCACCGTGGCCATGAACCGCTTTCCTCATGTCCTCGAGGTTCCCTGACTGACTTTATCCTGTTTTGTGTCTCCCCTTTGTCGTCTCCGCGCATGGCGCTTTCTTCTCCGCACTCCCCCCCCCCTTTAAGTGATCCCATCACGCATGGAACATTCGAATCAACCACACGCGTTCGCATACAACCACCTTTCTCTGCTCCTCTATTCACGTGATAGCTGTATACCCCCTCTCCCCGACACACACACACACACACACGCACGCGCGCACACGGACACAGACACAGGGTCGCTCGAAGAAGAGGAAAACAAAGGA

>BPK085/0 clone 8|LinJ36_V3.3750|500 bp UPS + CDS + 500 bp DWS|cysteine synthase

GTCGATGCCTCGGAGCTGCAGGACTGAGCTACCATCGGCAGCGCCGTCACTGCAGCCTTCGTGTGGCGCGAATGTGGAGAAGGAAAGGGCCAACGCTGGAGATCCACGCCTGCATAAGCAGGAGGGATGGAGACAGAAGATACGGTGGCAGTGGGAATACGGTAGCGGAGCGCAACGCATTTCTTGATGCCTTTCAAGCCGCACACCGCCGCTGCCTTCTTTGCAGCTCCAGTATCTGTTTGAAGCGCACGTGCAGCAAGCAGAGTGGTGGGAGCGGTGACCATCGCCGGTGATCAGGAGGAGGATACCTCGCTTTCTCCTTCATTCTCTTGTCCGCCTCTTCCTTTTTTTTTTCGGTCGTGCTCCTCTGCTACCACCTCAGTCTTACTGCTTAGCGTGTGTGACGGCCACCATTTCGCGAAGGGTATATTTTATGTGTGCTCGTGGTGGTGCAATGAGGAGGTCTTGCCATCCCTCTTCTCTTCCTCTTGCTTTCGTGAATGGCGGCACCGTTCGACAAGTCAAAAAATGTGGCGCAGTCTATCGATCAGCTGATTGGCCAGACGCCGGCGTTGTACCTAAACAAGCTGAACAATACCAAGGCAAAGGTTGTGCTCAAGATGGAGTGCGAAAACCCGATGGCGTCCGTGAAGGATCGCCTTGGCTTCGCCATCTACGATAAAGCGGAGAAGGAGGGCAAGCTGATCCCTGGCAAGTCTATCGTGGTCGAGTCGTCCAGTGGAAACACGGGCGTGTCGCTAGCGCACTTGGGTGCGATTCGTGGCTACAAGGTCATCATTACGATGCCCGAATCCATGTCTCTCGAGCGCCGCTGCCTGCTGCGTATCTTCGGCGCCGAGGTAATCCTCACCCCTGCCGCTCTTGGCATGAAGGGTGCCGTGGCCATGGCCAAGAAAATCGTCGCCGCTAACCCCAACGCCGTCTTGGCGGATCAGTTCGCAACCAAGTACAACGCCCTCATACACGAGGAAACCACGGGGCCTGAGATTTGGGAGCAGACGAACCACAATGTCGACTGCTTCATAGCCGGCGTTGGAACAGGTGGCACGCTGACAGGTGTGGCGCGGGCGCTAAAGAAGATGGGCAGTCATGCCCGCATCGTTGCCGTGGAGCCGACGGAGTCACCTGTGCTGTCGGGTGGAAAACCAGGCCCGCACAAAATTCAGGGCATCGGCCCGGGCTTTGTGCCAGACGTGCTCGACCGCAGTCTCATCGACGAGGTGCTTTGCGTGGCAGGTGATGATGCCATTGAGACGGCACTGAAGCTCACGCGCAGCGACGGGGTCTTCTGCGGCTTCTCTGGTGGCGCCAACGTTTACGCGGCGCTGAAGATCGCGGAGCGTCCGGAGATGGAGGGCAAAACAATTGTGACGGTCATTCCGTCCTTCGGTGAGCGCTACCTCTCCACCACGCTGTACAGGAGCGTTCGGGATGAGGTGTCGTCCCTGCCCGTAGGTGCCGATTTCTCTTCTCCCTCTCCCCCCCGTCAGCTTTACACTGGCGGATGGGTCGCGTGAGAGGTAGGAGAGGCGGATGCCACGCTGCATCGAGGCTGTTCCTCTACAATGCCGCCCACACAATCTCTTTCCAATCATCCTTGAAGTGTGTCCAGCTTCTCCCTTGCCTGCGCATCAAGCCCTGCCGTCACCGTGGCCATGAACCGCTTTCCTCATGTCCTCGAGGTTCCCTGACTGACTTTATCCTGTTTTGTGTCTCCCCTTTGTCGTCTCCGCGCATGGCGCTTTCTTCTCCGCACTCCCCCCCCCCTTTAAGTGATCCCATCACGCATGGAACATTCGAATCAACCACACGCGTTCGCATACAACCACCTTTCTCTGCTCCTCTATTCACGTGATAGCTGTATACCCCCTCTCCCCGACACACACACACACACACACGCACGCGCGCACACGGACACAGACACAGGGTCGCTCGAAGAAGAGGAAAACAAAGGA

>BPK087/0 clone 11|LinJ36_V3.3750|500 bp UPS + CDS + 500 bp DWS|cysteine synthase

GTCGATGCCTCGGAGCTGCAGGACTGAGCTACCATCGGCAGCGCCGTCACTGCAGCCTTCGTGTGGCGCGAATGTGGAGAAGGAAAGGGCCAACGCTGGAGATCCACGCCTGCATAAGCAGGAGGGATGGAGACAGAAGATACGGTGGCAGTGGGAATACGGTAGCGGAGCGCAACGCATTTCTTGATGCCTTTCAAGCCGCACACCGCCGCTGCCTTCTTTGCAGCTCCAGTATCTGTTTGAAGCGCACGTGCAGCAAGCAGAGTGGTGGGAGCGGTGACCATCGCCGGTGATCAGGAGGAGGATACCTCGCTTTCTCCTTCATTCTCTTGTCCGCCTCTTCCTTTTTTTTTTCGGTCGTGCTCCTCTGCTACCACCTCAGTCTTACTGCTTAGCGTGTGTGACGGCCACCATTTCGCGAAGGGTATATTTTATGTGTGCTCGTGGTGGTGCAATGAGGAGGTCTTGCCATCCCTCTTCTCTTCCTCTTGCTTTCGTGAATGGCGGCACCGTTCGACAAGTCAAAAAATGTGGCGCAGTCTATCGATCAGCTGATTGGCCAGACGCCGGCGTTGTACCTAAACAAGCTGAACAATACCAAGGCAAAGGTTGTGCTCAAGATGGAGTGCGAAAACCCGATGGCGTCCGTGAAGGATCGCCTTGGCTTCGCCATCTACGATAAAGCGGAGAAGGAGGGCAAGCTGATCCCTGGCAAGTCTATCGTGGTCGAGTCGTCCAGTGGAAACACGGGCGTGTCGCTAGCGCACTTGGGTGCGATTCGTGGCTACAAGGTCATCATTACGATGCCCGAATCCATGTCTCTCGAGCGCCGCTGCCTGCTGCGTATCTTCGGCGCCGAGGTAATCCTCACCCCTGCCGCTCTTGGCATGAAGGGTGCCGTGGCCATGGCCAAGAAAATCGTCGCCGCTAACCCCAACGCCGTCTTGGCGGATCAGTTCGCAACCAAGTACAACGCCCTCATACACGAGGAAACCACGGGGCCTGAGATTTGGGAGCAGACGAACCACAATGTCGACTGCTTCATAGCCGGCGTTGGAACAGGTGGCACGCTGACAGGTGTGGCGCGGGCGCTAAAGAAGATGGGCAGTCATGCCCGCATCGTTGCCGTGGAGCCGACGGAGTCACCTGTGCTGTCGGGTGGAAAACCAGGCCCGCACAAAATTCAGGGCATCGGCCCGGGCTTTGTGCCAGACGTGCTCGACCGCAGTCTCATCGACGAGGTGCTTTGCGTGGCAGGTGATGATGCCATTGAGACGGCACTGAAGCTCACGCGCAGCGACGGGGTCTTCTGCGGCTTCTCTGGTGGCGCCAACGTTTACGCGGCGCTGAAGATCGCGGAGCGTCCGGAGATGGAGGGCAAAACAATTGTGACGGTCATTCCGTCCTTCGGTGAGCGCTACCTCTCCACCACGCTGTACAGGAGCGTTCGGGATGAGGTGTCGTCCCTGCCCGTAGGTGCCGATTTCTCTTCTCCCTCTCCCCCCCGTCAGCTTTACACTGGCGGATGGGTCGCGTGAGAGGTAGGAGAGGCGGATGCCACGCTGCATCGAGGCTGTTCCTCTACAATGCCGCCCACACAATCTCTTTCCAATCATCCTTGAAGTGTGTCCAGCTTCTCCCTTGCCTGCGCATCAAGCCCTGCCGTCACCGTGGCCATGAACCGCTTTCCTCATGTCCTCGAGGTTCCCTGACTGACTTTATCCTGTTTTGTGTCTCCCCTTTGTCGTCTCCGCGCATGGCGCTTTCTTCTCCGCACTCCCCCCCCCCTTTAAGTGATCCCATCACGCATGGAACATTCGAATCAACCACACGCGTTCGCATACAACCACCTTTCTCTGCTCCTCTATTCACGTGATAGCTGTATACCCCCTCTCCCCGACACACACACACACACACACGCACGCGCGCACACGGACACAGACACAGGGTCGCTCGAAGAAGAGGAAAACAAAGGA

>BPK178/0 clone 3|LinJ36_V3.3750|500 bp UPS + CDS + 500 bp DWS|cysteine synthase

GTCGATGCCTCGGAGCTGCAGGACTGAGCTACCATCGGCAGCGCCGTCACTGCAGCCTTCGTGTGGCGCGAATGTGGAGAAGGAAAGGGCCAACGCTGGAGATCCACGCCTGCATAAGCAGGAGGGATGGAGACAGAAGATACGGTGGCAGTGGGAATACGGTAGCGGAGCGCAACGCATTTCTTGATGCCTTTCAAGCCGCACACCGCCGCTGCCTTCTTTGCAGCTCCAGTATCTGTTTGAAGCGCACGTGCAGCAAGCAGAGTGGTGGGAGCGGTGACCATCGCCGGTGATCAGGAGGAGGATACCTCGCTTTCTCCTTCATTCTCTTGTCCGCCTCTTCCTTTTTTTTTTCGGTCGTGCTCCTCTGCTACCACCTCAGTCTTACTGCTTAGCGTGTGTGACGGCCACCATTTCGCGAAGGGTATATTTTATGTGTGCTCGTGGTGGTGCAATGAGGAGGTCTTGCCATCCCTCTTCTCTTCCTCTTGCTTTCGTGAATGGCGGCACCGTTCGACAAGTCAAAAAATGTGGCGCAGTCTATCGATCAGCTGATTGGCCAGACGCCGGCGTTGTACCTAAACAAGCTGAACAATACCAAGGCAAAGGTTGTGCTCAAGATGGAGTGCGAAAACCCGATGGCGTCCGTGAAGGATCGCCTTGGCTTCGCCATCTACGATAAAGCGGAGAAGGAGGGCAAGCTGATCCCTGGCAAGTCTATCGTGGTCGAGTCGTCCAGTGGAAACACGGGCGTGTCGCTAGCGCACTTGGGTGCGATTCGTGGCTACAAGGTCATCATTACGATGCCCGAATCCATGTCTCTCGAGCGCCGCTGCCTGCTGCGTATCTTCGGCGCCGAGGTAATCCTCACCCCTGCCGCTCTTGGCATGAAGGGTGCCGTGGCCATGGCCAAGAAAATCGTCGCCGCTAACCCCAACGCCGTCTTGGCGGATCAGTTCGCAACCAAGTACAACGCCCTCATACACGAGGAAACCACGGGGCCTGAGATTTGGGAGCAGACGAACCACAATGTCGACTGCTTCATAGCCGGCGTTGGAACAGGTGGCACGCTGACAGGTGTGGCGCGGGCGCTAAAGAAGATGGGCAGTCATGCCCGCATCGTTGCCGTGGAGCCGACGGAGTCACCTGTGCTGTCGGGTGGAAAACCAGGCCCGCACAAAATTCAGGGCATCGGCCCGGGCTTTGTGCCAGACGTGCTCGACCGCAGTCTCATCGACGAGGTGCTTTGCGTGGCAGGTGATGATGCCATTGAGACGGCACTGAAGCTCACGCGCAGCGACGGGGTCTTCTGCGGCTTCTCTGGTGGCGCCAACGTTTACGCGGCGCTGAAGATCGCGGAGCGTCCGGAGATGGAGGGCAAAACAATTGTGACGGTCATTCCGTCCTTCGGTGAGCGCTACCTCTCCACCACGCTGTACAGGAGCGTTCGGGATGAGGTGTCGTCCCTGCCCGTAGGTGCCGATTTCTCTTCTCCCTCTCCCCCCCGTCAGCTTTACACTGGCGGATGGGTCGCGTGAGAGGTAGGAGAGGCGGATGCCACGCTGCATCGAGGCTGTTCCTCTACAATGCCGCCCACACAATCTCTTTCCAATCATCCTTGAAGTGTGTCCAGCTTCTCCCTTGCCTGCGCATCAAGCCCTGCCGTCACCGTGGCCATGAACCGCTTTCCTCATGTCCTCGAGGTTCCCTGACTGACTTTATCCTGTTTTGTGTCTCCCCTTTGTCGTCTCCGCGCATGGCGCTTTCTTCTCCGCACTCCCCCCCCCCTTTAAGTGATCCCATCACGCATGGAACATTCGAATCAACCACACGCGTTCGCATACAACCACCTTTCTCTGCTCCTCTATTCACGTGATAGCTGTATACCCCCTCTCCCCGACACACACACACACACACACGCACGCGCGCACACGGACACAGACACAGGGTCGCTCGAAGAAGAGGAAAACAAAGGA

>BPK190/0 clone 3|LinJ36_V3.3750|500 bp UPS + CDS + 500 bp DWS|cysteine synthase

GTCGATGCCTCGGAGCTGCAGGACTGAGCTACCATCGGCAGCGCCGTCACTGCAGCCTTCGTGTGGCGCGAATGTGGAGAAGGAAAGGGCCAACGCTGGAGATCCACGCCTGCATAAGCAGGAGGGATGGAGACAGAAGATACGGTGGCAGTGGGAATACGGTAGCGGAGCGCAACGCATTTCTTGATGCCTTTCAAGCCGCACACCGCCGCTGCCTTCTTTGCAGCTCCAGTATCTGTTTGAAGCGCACGTGCAGCAAGCAGAGTGGTGGGAGCGGTGACCATCGCCGGTGATCAGGAGGAGGATACCTCGCTTTCTCCTTCATTCTCTTGTCCGCCTCTTCCTTTTTTTTTTCGGTCGTGCTCCTCTGCTACCACCTCAGTCTTACTGCTTAGCGTGTGTGACGGCCACCATTTCGCGAAGGGTATATTTTATGTGTGCTCGTGGTGGTGCAATGAGGAGGTCTTGCCATCCCTCTTCTCTTCCTCTTGCTTTCGTGAATGGCGGCACCGTTCGACAAGTCAAAAAATGTGGCGCAGTCTATCGATCAGCTGATTGGCCAGACGCCGGCGTTGTACCTAAACAAGCTGAACAATACCAAGGCAAAGGTTGTGCTCAAGATGGAGTGCGAAAACCCGATGGCGTCCGTGAAGGATCGCCTTGGCTTCGCCATCTACGATAAAGCGGAGAAGGAGGGCAAGCTGATCCCTGGCAAGTCTATCGTGGTCGAGTCGTCCAGTGGAAACACGGGCGTGTCGCTAGCGCACTTGGGTGCGATTCGTGGCTACAAGGTCATCATTACGATGCCCGAATCCATGTCTCTCGAGCGCCGCTGCCTGCTGCGTATCTTCGGCGCCGAGGTAATCCTCACCCCTGCCGCTCTTGGCATGAAGGGTGCCGTGGCCATGGCCAAGAAAATCGTCGCCGCTAACCCCAACGCCGTCTTGGCGGATCAGTTCGCAACCAAGTACAACGCCCTCATACACGAGGAAACCACGGGGCCTGAGATTTGGGAGCAGACGAACCACAATGTCGACTGCTTCATAGCCGGCGTTGGAACAGGTGGCACGCTGACAGGTGTGGCGCGGGCGCTAAAGAAGATGGGCAGTCATGCCCGCATCGTTGCCGTGGAGCCGACGGAGTCACCTGTGCTGTCGGGTGGAAAACCAGGCCCGCACAAAATTCAGGGCATCGGCCCGGGCTTTGTGCCAGACGTGCTCGACCGCAGTCTCATCGACGAGGTGCTTTGCGTGGCAGGTGATGATGCCATTGAGACGGCACTGAAGCTCACGCGCAGCGACGGGGTCTTCTGCGGCTTCTCTGGTGGCGCCAACGTTTACGCGGCGCTGAAGATCGCGGAGCGTCCGGAGATGGAGGGCAAAACAATTGTGACGGTCATTCCGTCCTTCGGTGAGCGCTACCTCTCCACCACGCTGTACAGGAGCGTTCGGGATGAGGTGTCGTCCCTGCCCGTAGGTGCCGATTTCTCTTCTCCCTCTCCCCCCCGTCAGCTTTACACTGGCGGATGGGTCGCGTGAGAGGTAGGAGAGGCGGATGCCACGCTGCATCGAGGCTGTTCCTCTACAATGCCGCCCACACAATCTCTTTCCAATCATCCTTGAAGTGTGTCCAGCTTCTCCCTTGCCTGCGCATCAAGCCCTGCCGTCACCGTGGCCATGAACCGCTTTCCTCATGTCCTCGAGGTTCCCTGACTGACTTTATCCTGTTTTGTGTCTCCCCTTTGTCGTCTCCGCGCATGGCGCTTTCTTCTCCGCACTCCCCCCCCCCTTTAAGTGATCCCATCACGCATGGAACATTCGAATCAACCACACGCGTTCGCATACAACCACCTTTCTCTGCTCCTCTATTCACGTGATAGCTGTATACCCCCTCTCCCCGACACACACACACACACACACGCACGCGCGCACACGGACACAGACACAGGGTCGCTCGAAGAAGAGGAAAACAAAGGA

>BPK206/0 clone 10|LinJ36_V3.3750|500 bp UPS + CDS + 500 bp DWS|cysteine synthase

GTCGATGCCTCGGAGCTGCAGGACTGAGCTACCATCGGCAGCGCCGTCACTGCAGCCTTCGTGTGGCGCGAATGTGGAGAAGGAAAGGGCCAACGCTGGAGATCCACGCCTGCATAAGCAGGAGGGATGGAGACAGAAGATACGGTGGCAGTGGGAATACGGTAGCGGAGCGCAACGCATTTCTTGATGCCTTTCAAGCCGCACACCGCCGCTGCCTTCTTTGCAGCTCCAGTATCTGTTTGAAGCGCACGTGCAGCAAGCAGAGTGGTGGGAGCGGTGACCATCGCCGGTGATCAGGAGGAGGATACCTCGCTTTCTCCTTCATTCTCTTGTCCGCCTCTTCCTTTTTTTTTTCGGTCGTGCTCCTCTGCTACCACCTCAGTCTTACTGCTTAGCGTGTGTGACGGCCACCATTTCGCGAAGGGTATATTTTATGTGTGCTCGTGGTGGTGCAATGAGGAGGTCTTGCCATCCCTCTTCTCTTCCTCTTGCTTTCGTGAATGGCGGCACCGTTCGACAAGTCAAAAAATGTGGCGCAGTCTATCGATCAGCTGATTGGCCAGACGCCGGCGTTGTACCTAAACAAGCTGAACAATACCAAGGCAAAGGTTGTGCTCAAGATGGAGTGCGAAAACCCGATGGCGTCCGTGAAGGATCGCCTTGGCTTCGCCATCTACGATAAAGCGGAGAAGGAGGGCAAGCTGATCCCTGGCAAGTCTATCGTGGTCGAGTCGTCCAGTGGAAACACGGGCGTGTCGCTAGCGCACTTGGGTGCGATTCGTGGCTACAAGGTCATCATTACGATGCCCGAATCCATGTCTCTCGAGCGCCGCTGCCTGCTGCGTATCTTCGGCGCCGAGGTAATCCTCACCCCTGCCGCTCTTGGCATGAAGGGTGCCGTGGCCATGGCCAAGAAAATCGTCGCCGCTAACCCCAACGCCGTCTTGGCGGATCAGTTCGCAACCAAGTACAACGCCCTCATACACGAGGAAACCACGGGGCCTGAGATTTGGGAGCAGACGAACCACAATGTCGACTGCTTCATAGCCGGCGTTGGAACAGGTGGCACGCTGACAGGTGTGGCGCGGGCGCTAAAGAAGATGGGCAGTCATGCCCGCATCGTTGCCGTGGAGCCGACGGAGTCACCTGTGCTGTCGGGTGGAAAACCAGGCCCGCACAAAATTCAGGGCATCGGCCCGGGCTTTGTGCCAGACGTGCTCGACCGCAGTCTCATCGACGAGGTGCTTTGCGTGGCAGGTGATGATGCCATTGAGACGGCACTGAAGCTCACGCGCAGCGACGGGGTCTTCTGCGGCTTCTCTGGTGGCGCCAACGTTTACGCGGCGCTGAAGATCGCGGAGCGTCCGGAGATGGAGGGCAAAACAATTGTGACGGTCATTCCGTCCTTCGGTGAGCGCTACCTCTCCACCACGCTGTACAGGAGCGTTCGGGATGAGGTGTCGTCCCTGCCCGTAGGTGCCGATTTCTCTTCTCCCTCTCCCCCCCGTCAGCTTTACACTGGCGGATGGGTCGCGTGAGAGGTAGGAGAGGCGGATGCCACGCTGCATCGAGGCTGTTCCTCTACAATGCCGCCCACACAATCTCTTTCCAATCATCCTTGAAGTGTGTCCAGCTTCTCCCTTGCCTGCGCATCAAGCCCTGCCGTCACCGTGGCCATGAACCGCTTTCCTCATGTCCTCGAGGTTCCCTGACTGACTTTATCCTGTTTTGTGTCTCCCCTTTGTCGTCTCCGCGCATGGCGCTTTCTTCTCCGCACTCCCCCCCCCCTTTAAGTGATCCCATCACGCATGGAACATTCGAATCAACCACACGCGTTCGCATACAACCACCTTTCTCTGCTCCTCTATTCACGTGATAGCTGTATACCCCCTCTCCCCGACACACACACACACACACACGCACGCGCGCACACGGACACAGACACAGGGTCGCTCGAAGAAGAGGAAAACAAAGGA

>BPK275/0 clone 18|LinJ36_V3.3750|500 bp UPS + CDS + 500 bp DWS|cysteine synthase

GTCGATGCCTCGGAGCTGCAGGACTGAGCTACCATCGGCAGCGCCGTCACTGCAGCCTTCGTGTGGCGCGAATGTGGAGAAGGAAAGGGCCAACGCTGGAGATCCACGCCTGCATAAGCAGGAGGGATGGAGACAGAAGATACGGTGGCAGTGGGAATACGGTAGCGGAGCGCAACGCATTTCTTGATGCCTTTCAAGCCGCACACCGCCGCTGCCTTCTTTGCAGCTCCAGTATCTGTTTGAAGCGCACGTGCAGCAAGCAGAGTGGTGGGAGCGGTGACCATCGCCGGTGATCAGGAGGAGGATACCTCGCTTTCTCCTTCATTCTCTTGTCCGCCTCTTCCTTTTTTTTTTCGGTCGTGCTCCTCTGCTACCACCTCAGTCTTACTGCTTAGCGTGTGTGACGGCCACCATTTCGCGAAGGGTATATTTTATGTGTGCTCGTGGTGGTGCAATGAGGAGGTCTTGCCATCCCTCTTCTCTTCCTCTTGCTTTCGTGAATGGCGGCACCGTTCGACAAGTCAAAAAATGTGGCGCAGTCTATCGATCAGCTGATTGGCCAGACGCCGGCGTTGTACCTAAACAAGCTGAACAATACCAAGGCAAAGGTTGTGCTCAAGATGGAGTGCGAAAACCCGATGGCGTCCGTGAAGGATCGCCTTGGCTTCGCCATCTACGATAAAGCGGAGAAGGAGGGCAAGCTGATCCCTGGCAAGTCTATCGTGGTCGAGTCGTCCAGTGGAAACACGGGCGTGTCGCTAGCGCACTTGGGTGCGATTCGTGGCTACAAGGTCATCATTACGATGCCCGAATCCATGTCTCTCGAGCGCCGCTGCCTGCTGCGTATCTTCGGCGCCGAGGTAATCCTCACCCCTGCCGCTCTTGGCATGAAGGGTGCCGTGGCCATGGCCAAGAAAATCGTCGCCGCTAACCCCAACGCCGTCTTGGCGGATCAGTTCGCAACCAAGTACAACGCCCTCATACACGAGGAAACCACGGGGCCTGAGATTTGGGAGCAGACGAACCACAATGTCGACTGCTTCATAGCCGGCGTTGGAACAGGTGGCACGCTGACAGGTGTGGCGCGGGCGCTAAAGAAGATGGGCAGTCATGCCCGCATCGTTGCCGTGGAGCCGACGGAGTCACCTGTGCTGTCGGGTGGAAAACCAGGCCCGCACAAAATTCAGGGCATCGGCCCGGGCTTTGTGCCAGACGTGCTCGACCGCAGTCTCATCGACGAGGTGCTTTGCGTGGCAGGTGATGATGCCATTGAGACGGCACTGAAGCTCACGCGCAGCGACGGGGTCTTCTGCGGCTTCTCTGGTGGCGCCAACGTTTACGCGGCGCTGAAGATCGCGGAGCGTCCGGAGATGGAGGGCAAAACAATTGTGACGGTCATTCCGTCCTTCGGTGAGCGCTACCTCTCCACCACGCTGTACAGGAGCGTTCGGGATGAGGTGTCGTCCCTGCCCGTAGGTGCCGATTTCTCTTCTCCCTCTCCCCCCCGTCAGCTTTACACTGGCGGATGGGTCGCGTGAGAGGTAGGAGAGGCGGATGCCACGCTGCATCGAGGCTGTTCCTCTACAATGCCGCCCACACAATCTCTTTCCAATCATCCTTGAAGTGTGTCCAGCTTCTCCCTTGCCTGCGCATCAAGCCCTGCCGTCACCGTGGCCATGAACCGCTTTCCTCATGTCCTCGAGGTTCCCTGACTGACTTTATCCTGTTTTGTGTCTCCCCTTTGTCGTCTCCGCGCATGGCGCTTTCTTCTCCGCACTCCCCCCCCCCTTTAAGTGATCCCATCACGCATGGAACATTCGAATCAACCACACGCGTTCGCATACAACCACCTTTCTCTGCTCCTCTATTCACGTGATAGCTGTATACCCCCTCTCCCCGACACACACACACACACACACGCACGCGCGCACACGGACACAGACACAGGGTCGCTCGAAGAAGAGGAAAACAAAGGA

>BPK282/0 clone 4|LinJ36_V3.3750|500 bp UPS + CDS + 500 bp DWS|cysteine synthase

GTCGATGCCTCGGAGCTGCAGGACTGAGCTACCATCGGCAGCGCCGTCACTGCAGCCTTCGTGTGGCGCGAATGTGGAGAAGGAAAGGGCCAACGCTGGAGATCCACGCCTGCATAAGCAGGAGGGATGGAGACAGAAGATACGGTGGCAGTGGGAATACGGTAGCGGAGCGCAACGCATTTCTTGATGCCTTTCAAGCCGCACACCGCCGCTGCCTTCTTTGCAGCTCCAGTATCTGTTTGAAGCGCACGTGCAGCAAGCAGAGTGGTGGGAGCGGTGACCATCGCCGGTGATCAGGAGGAGGATACCTCGCTTTCTCCTTCATTCTCTTGTCCGCCTCTTCCTTTTTTTTTTCGGTCGTGCTCCTCTGCTACCACCTCAGTCTTACTGCTTAGCGTGTGTGACGGCCACCATTTCGCGAAGGGTATATTTTATGTGTGCTCGTGGTGGTGCAATGAGGAGGTCTTGCCATCCCTCTTCTCTTCCTCTTGCTTTCGTGAATGGCGGCACCGTTCGACAAGTCAAAAAATGTGGCGCAGTCTATCGATCAGCTGATTGGCCAGACGCCGGCGTTGTACCTAAACAAGCTGAACAATACCAAGGCAAAGGTTGTGCTCAAGATGGAGTGCGAAAACCCGATGGCGTCCGTGAAGGATCGCCTTGGCTTCGCCATCTACGATAAAGCGGAGAAGGAGGGCAAGCTGATCCCTGGCAAGTCTATCGTGGTCGAGTCGTCCAGTGGAAACACGGGCGTGTCGCTAGCGCACTTGGGTGCGATTCGTGGCTACAAGGTCATCATTACGATGCCCGAATCCATGTCTCTCGAGCGCCGCTGCCTGCTGCGTATCTTCGGCGCCGAGGTAATCCTCACCCCTGCCGCTCTTGGCATGAAGGGTGCCGTGGCCATGGCCAAGAAAATCGTCGCCGCTAACCCCAACGCCGTCTTGGCGGATCAGTTCGCAACCAAGTACAACGCCCTCATACACGAGGAAACCACGGGGCCTGAGATTTGGGAGCAGACGAACCACAATGTCGACTGCTTCATAGCCGGCGTTGGAACAGGTGGCACGCTGACAGGTGTGGCGCGGGCGCTAAAGAAGATGGGCAGTCATGCCCGCATCGTTGCCGTGGAGCCGACGGAGTCACCTGTGCTGTCGGGTGGAAAACCAGGCCCGCACAAAATTCAGGGCATCGGCCCGGGCTTTGTGCCAGACGTGCTCGACCGCAGTCTCATCGACGAGGTGCTTTGCGTGGCAGGTGATGATGCCATTGAGACGGCACTGAAGCTCACGCGCAGCGACGGGGTCTTCTGCGGCTTCTCTGGTGGCGCCAACGTTTACGCGGCGCTGAAGATCGCGGAGCGTCCGGAGATGGAGGGCAAAACAATTGTGACGGTCATTCCGTCCTTCGGTGAGCGCTACCTCTCCACCACGCTGTACAGGAGCGTTCGGGATGAGGTGTCGTCCCTGCCCGTAGGTGCCGATTTCTCTTCTCCCTCTCCCCCCCGTCAGCTTTACACTGGCGGATGGGTCGCGTGAGAGGTAGGAGAGGCGGATGCCACGCTGCATCGAGGCTGTTCCTCTACAATGCCGCCCACACAATCTCTTTCCAATCATCCTTGAAGTGTGTCCAGCTTCTCCCTTGCCTGCGCATCAAGCCCTGCCGTCACCGTGGCCATGAACCGCTTTCCTCATGTCCTCGAGGTTCCCTGACTGACTTTATCCTGTTTTGTGTCTCCCCTTTGTCGTCTCCGCGCATGGCGCTTTCTTCTCCGCACTCCCCCCCCCCTTTAAGTGATCCCATCACGCATGGAACATTCGAATCAACCACACGCGTTCGCATACAACCACCTTTCTCTGCTCCTCTATTCACGTGATAGCTGTATACCCCCTCTCCCCGACACACACACACACACACACGCACGCGCGCACACGGACACAGACACAGGGTCGCTCGAAGAAGAGGAAAACAAAGGA

>BPK294/0 clone 1|LinJ36_V3.3750|500 bp UPS + CDS + 500 bp DWS|cysteine synthase

GTCGATGCCTCGGAGCTGCAGGACTGAGCTACCATCGGCAGCGCCGTCACTGCAGCCTTCGTGTGGCGCGAATGTGGAGAAGGAAAGGGCCAACGCTGGAGATCCACGCCTGCATAAGCAGGAGGGATGGAGACAGAAGATACGGTGGCAGTGGGAATACGGTAGCGGAGCGCAACGCATTTCTTGATGCCTTTCAAGCCGCACACCGCCGCTGCCTTCTTTGCAGCTCCAGTATCTGTTTGAAGCGCACGTGCAGCAAGCAGAGTGGTGGGAGCGGTGACCATCGCCGGTGATCAGGAGGAGGATACCTCGCTTTCTCCTTCATTCTCTTGTCCGCCTCTTCCTTTTTTTTTTCGGTCGTGCTCCTCTGCTACCACCTCAGTCTTACTGCTTAGCGTGTGTGACGGCCACCATTTCGCGAAGGGTATATTTTATGTGTGCTCGTGGTGGTGCAATGAGGAGGTCTTGCCATCCCTCTTCTCTTCCTCTTGCTTTCGTGAATGGCGGCACCGTTCGACAAGTCAAAAAATGTGGCGCAGTCTATCGATCAGCTGATTGGCCAGACGCCGGCGTTGTACCTAAACAAGCTGAACAATACCAAGGCAAAGGTTGTGCTCAAGATGGAGTGCGAAAACCCGATGGCGTCCGTGAAGGATCGCCTTGGCTTCGCCATCTACGATAAAGCGGAGAAGGAGGGCAAGCTGATCCCTGGCAAGTCTATCGTGGTCGAGTCGTCCAGTGGAAACACGGGCGTGTCGCTAGCGCACTTGGGTGCGATTCGTGGCTACAAGGTCATCATTACGATGCCCGAATCCATGTCTCTCGAGCGCCGCTGCCTGCTGCGTATCTTCGGCGCCGAGGTAATCCTCACCCCTGCCGCTCTTGGCATGAAGGGTGCCGTGGCCATGGCCAAGAAAATCGTCGCCGCTAACCCCAACGCCGTCTTGGCGGATCAGTTCGCAACCAAGTACAACGCCCTCATACACGAGGAAACCACGGGGCCTGAGATTTGGGAGCAGACGAACCACAATGTCGACTGCTTCATAGCCGGCGTTGGAACAGGTGGCACGCTGACAGGTGTGGCGCGGGCGCTAAAGAAGATGGGCAGTCATGCCCGCATCGTTGCCGTGGAGCCGACGGAGTCACCTGTGCTGTCGGGTGGAAAACCAGGCCCGCACAAAATTCAGGGCATCGGCCCGGGCTTTGTGCCAGACGTGCTCGACCGCAGTCTCATCGACGAGGTGCTTTGCGTGGCAGGTGATGATGCCATTGAGACGGCACTGAAGCTCACGCGCAGCGACGGGGTCTTCTGCGGCTTCTCTGGTGGCGCCAACGTTTACGCGGCGCTGAAGATCGCGGAGCGTCCGGAGATGGAGGGCAAAACAATTGTGACGGTCATTCCGTCCTTCGGTGAGCGCTACCTCTCCACCACGCTGTACAGGAGCGTTCGGGATGAGGTGTCGTCCCTGCCCGTAGGTGCCGATTTCTCTTCTCCCTCTCCCCCCCGTCAGCTTTACACTGGCGGATGGGTCGCGTGAGAGGTAGGAGAGGCGGATGCCACGCTGCATCGAGGCTGTTCCTCTACAATGCCGCCCACACAATCTCTTTCCAATCATCCTTGAAGTGTGTCCAGCTTCTCCCTTGCCTGCGCATCAAGCCCTGCCGTCACCGTGGCCATGAACCGCTTTCCTCATGTCCTCGAGGTTCCCTGACTGACTTTATCCTGTTTTGTGTCTCCCCTTTGTCGTCTCCGCGCATGGCGCTTTCTTCTCCGCACTCCCCCCCCCCTTTAAGTGATCCCATCACGCATGGAACATTCGAATCAACCACACGCGTTCGCATACAACCACCTTTCTCTGCTCCTCTATTCACGTGATAGCTGTATACCCCCTCTCCCCGACACACACACACACACACACGCACGCGCGCACACGGACACAGACACAGGGTCGCTCGAAGAAGAGGAAAACAAAGGA

>BPK298/0 clone 8|LinJ36_V3.3750|500 bp UPS + CDS + 500 bp DWS|cysteine synthase

GTCGATGCCTCGGAGCTGCAGGACTGAGCTACCATCGGCAGCGCCGTCACTGCAGCCTTCGTGTGGCGCGAATGTGGAGAAGGAAAGGGCCAACGCTGGAGATCCACGCCTGCATAAGCAGGAGGGATGGAGACAGAAGATACGGTGGCAGTGGGAATACGGTAGCGGAGCGCAACGCATTTCTTGATGCCTTTCAAGCCGCACACCGCCGCTGCCTTCTTTGCAGCTCCAGTATCTGTTTGAAGCGCACGTGCAGCAAGCAGAGTGGTGGGAGCGGTGACCATCGCCGGTGATCAGGAGGAGGATACCTCGCTTTCTCCTTCATTCTCTTGTCCGCCTCTTCCTTTTTTTTTTCGGTCGTGCTCCTCTGCTACCACCTCAGTCTTACTGCTTAGCGTGTGTGACGGCCACCATTTCGCGAAGGGTATATTTTATGTGTGCTCGTGGTGGTGCAATGAGGAGGTCTTGCCATCCCTCTTCTCTTCCTCTTGCTTTCGTGAATGGCGGCACCGTTCGACAAGTCAAAAAATGTGGCGCAGTCTATCGATCAGCTGATTGGCCAGACGCCGGCGTTGTACCTAAACAAGCTGAACAATACCAAGGCAAAGGTTGTGCTCAAGATGGAGTGCGAAAACCCGATGGCGTCCGTGAAGGATCGCCTTGGCTTCGCCATCTACGATAAAGCGGAGAAGGAGGGCAAGCTGATCCCTGGCAAGTCTATCGTGGTCGAGTCGTCCAGTGGAAACACGGGCGTGTCGCTAGCGCACTTGGGTGCGATTCGTGGCTACAAGGTCATCATTACGATGCCCGAATCCATGTCTCTCGAGCGCCGCTGCCTGCTGCGTATCTTCGGCGCCGAGGTAATCCTCACCCCTGCCGCTCTTGGCATGAAGGGTGCCGTGGCCATGGCCAAGAAAATCGTCGCCGCTAACCCCAACGCCGTCTTGGCGGATCAGTTCGCAACCAAGTACAACGCCCTCATACACGAGGAAACCACGGGGCCTGAGATTTGGGAGCAGACGAACCACAATGTCGACTGCTTCATAGCCGGCGTTGGAACAGGTGGCACGCTGACAGGTGTGGCGCGGGCGCTAAAGAAGATGGGCAGTCATGCCCGCATCGTTGCCGTGGAGCCGACGGAGTCACCTGTGCTGTCGGGTGGAAAACCAGGCCCGCACAAAATTCAGGGCATCGGCCCGGGCTTTGTGCCAGACGTGCTCGACCGCAGTCTCATCGACGAGGTGCTTTGCGTGGCAGGTGATGATGCCATTGAGACGGCACTGAAGCTCACGCGCAGCGACGGGGTCTTCTGCGGCTTCTCTGGTGGCGCCAACGTTTACGCGGCGCTGAAGATCGCGGAGCGTCCGGAGATGGAGGGCAAAACAATTGTGACGGTCATTCCGTCCTTCGGTGAGCGCTACCTCTCCACCACGCTGTACAGGAGCGTTCGGGATGAGGTGTCGTCCCTGCCCGTAGGTGCCGATTTCTCTTCTCCCTCTCCCCCCCGTCAGCTTTACACTGGCGGATGGGTCGCGTGAGAGGTAGGAGAGGCGGATGCCACGCTGCATCGAGGCTGTTCCTCTACAATGCCGCCCACACAATCTCTTTCCAATCATCCTTGAAGTGTGTCCAGCTTCTCCCTTGCCTGCGCATCAAGCCCTGCCGTCACCGTGGCCATGAACCGCTTTCCTCATGTCCTCGAGGTTCCCTGACTGACTTTATCCTGTTTTGTGTCTCCCCTTTGTCGTCTCCGCGCATGGCGCTTTCTTCTCCGCACTCCCCCCCCCCTTTAAGTGATCCCATCACGCATGGAACATTCGAATCAACCACACGCGTTCGCATACAACCACCTTTCTCTGCTCCTCTATTCACGTGATAGCTGTATACCCCCTCTCCCCGACACACACACACACACACACGCACGCGCGCACACGGACACAGACACAGGGTCGCTCGAAGAAGAGGAAAACAAAGGA

### 8. ORNITHINE DECARBOXYLASE

>BPK035/0 clone 1|LinJ.12.0100|500 bp UPS + CDS + 500 bp DWS|ornithine decarboxylase

GCTCCTTCTTGTCGCGGCTGTGTGGGTTCGCTCATTCTCGGGGTGTTGTCCCCCTTCCCTCGTGGCATCGTCATCGTCTTCTCCACCGCCACCGTCCACGCTGCACACGCACACTCCACAGCGTACTTCACCTTCCTAGCTGTCTCCCTCACTCCCTCTCTGTAACCCTAACTGTCGTTGCGGCTACCGCTCTGCGTCCTTTCCGGCAACTTCTCCTGCGGTTCACTGCCAGCGTTGCTCCTACCTGTGGTGCCCCGTTCCTCTGGCCCCTCACACACACACACACTCACCTGCACTCTCTCAGTCTCCTTTGTCAACCGCTGGAGCTCAGCGCATTCGCTTTTTCGGCCGCCGCTTCGCCTCCATCTCGCGCCGCCGCACCTCCTCGCACCTCCCTCCTCCCTCGCCCCGCGCGCGCCGGCCTCTCTCTGCATTCCGCACAGACCTGCCAAGGCGCTCTCCCGTGTGTGTCGTCGCCCGCCGTCCTTCCCAAGTGCTCAATGGGTGATCATGACGTCGCTCTCTGCCACGTCAGCCGCTACAACCATGCGAATTACTGGGCCTTTGTGCCGCTGCCAACGGTCAGCGACGACACGGGATGCGACAGTCTGCATCATGACAGCGCTTCCAAAAGGATAAGGATGGCGCCGCCGGCGTCCGCGTCGAAGGCGGGCGCGGCCGAGGAACGCCTGCACCCCTACGAAAGGCGTCTATTGGATCAGTACCAGATCCACCTCCAACCCGCCAACCGCAACCCGCTGAGCCGAGCCGATTCCGCCGCCGGTCGAGAGGAAACGGCGCAGACCCCCGCGCAGGTGCAGATGGTGTCTGGTGTTGCTGTTGCGGACTCGACTTCAGATCAGCACGCCTCTGTTGCATCCAGTCAGGACCTCGTCGACCTCTTCTTCCTGAGGGCAGCCAGGCCGTCGACGGTCTGTGCTTCTCGCCCTACCCCATCTACGGTTGGCGCACGGCCGAGGAGCGCCGGGCTGCCGTTTGTGAGGTTTTCAAGACGTACAACGTGGTCACGCGCCTGCCCGCCTCGCCGGCTGCCCTGGCCGCCGCGCAGCGCCGCTACAGCCGCCACCGGCACTCCGCCATCGCCCCCATCAACAAGAGTGCGATCGAGACGCGTGAGCAGTACTGGCGCCGCCTGTCGAACCTGTACACCCAGAAGGGTGTAAAAGATGCTGCTTCTGCCGCCGACGCTGCGGCTACGACGGCGACGAACGGTGCCGTCCCCGCTGCCCCCGCCTACGAGCCCGAGGATCCATTCTACATCATCGATCTCGGCCGTGTTGTGGAGCAGATGGCGCGCTGGCGTCACGAGCTGCCGATGGTGCGCCCTTACTTTGCCGTGAAAAGCAACCCGCAACCGGCTGTTTTGGAGGTGCTCAGCGCCCTCGGCGCCGGTTTCGACTGCGCGTCCAAGGAAGAAATACATATGGTGCTAGGTCACCAGCTCGTGGCGTCGCCGGACGACATCATCTTCGCCAACCCGTGCAAGCAGCTCGGCGACCTGCGTGAGGCGCAGGCGTGCGGCGTGACCTACGTGACGGTGGACAACCCGCTGGAGATGGAAAAGATCAGTCGCCTGATGCCCTCCGCCCACGCGATTATCCGCATCAAGACGAACGACAGCAAGGCCCAGTGCTCCTTTTCCACCAAGTTCGGTGCCCCCCTTGAAGATGTCGAGGGCCTACTGGAGGCGGCCCGCCAGTTCAACGTCACCGTGTGCGGCGTCAGCTTTCACGTCGGCAGCGGCAACGACGACCAGTCTGCGTACGTGTCCGCGGTACGCGATGCCTACCAAGTCTTTCAGCAGGCGGTCCAGTACGGCTTCAAGTGCACCATCCTCGACATCGGCGGCGGCTTCCCGGGCACGGAGGTCGTCGAGGGCAGCGGCAATACCTCTTTCGAGGCCATTGCCCGTACTATTCGACCGGTGCTGGCGGAGCTCTTCGGCGGCGGTGATGTCACCATCATCAGTGAGCCCGGCCGCTACTTCACGGCTGCCTCGCACGCGCTTCTCATGAACGTATTCGCCTCCCGCACGCTGCGACTGTCCGATGTCGAGGTGAGTCGCCAAGCCTTCCAGTCCGTCGTGTCGATGGACGAGCCCGAAGAGTACCAGTACTACGTCAACGACGGCCTCTACCACAGCTTCAACTGCATCCTCTTCGATCACGCCCACCCAACGCTTCTCCTGCTAAACGACGGCGACGGCGCTGACGGGGTGGAGAGTGGCACGGAGGCGGCAGCGGTGTGCAGCGAGGAGGAGGGCGAGACGTCGCTGAGCGGTCCCCTGGCGAACGATCCACTGTTCATGTCCGCCTGGGACCGCCGTCGCAGCTTCGCGCGCCGCCCGCTGCGCATCACAACCATCTTCGGCCCCACCTGCGATTCGATGGACTGCATCCTGAAGAAGCAGCCCTTCCCGGAGATGAAGCTGGGCGACTGGCTTCTGGTGCCAGACATGGGCAGCTACACCACCGCCGCCGCCGGCTTCTTCAACGGTTTCGCGACGCGCCGCCTTGAGTGGGTGAGCTCTGTGGACTTGTGCGCGAGGCCGAGGCCTGTGTATACGCGTGAGGGCAACACGCTGAGGTGTGTGAGCGAGTGAGAGAGGGACAGAAAGGAGACCCGGTGCTGTAGGGTGTGCTCGTCCATCCCACAAGAGGATGGACGGTCTCTTCGCTCCGGCCACCTCACCACCTCCTTTAGCCGCCCCCTCCTCCGCCCCCTCTCCCCCTATCGTCCCCTTTTCTGTGGTGGTTGCACTTGTATTCGTGTTGGATGCACGGCGACGCTGCTCGCTTGTGTTTGTGCGCCTGCGCCTGCGCGCGTGTTTCGCTTTTCGTTCACCCCCTGTGGTCCCTTCGTGAGCCTCCCCATCCCCGCTCTTCTTCGCCGCTGGCTTCGTTGTTGCTCTTCTCTGACGCCTACGCAACGCAAGCCCCCCCCCTCTCCCTCACCCTTGTGCCCCACAAACTCCCACTGACAGTGCTTCCACTTACTAAGTCGGCCCAACCCCAGCGAAGCCCCCTCGCCGCCGCTGCTGCCGCCGACCCCTTCTCTCTCTTGCCTCGTGTCTCTCCTGCTCCTTTTCTCTTGTCGTGTACA

>BPK043/0 clone 2|LinJ.12.0100|500 bp UPS + CDS + 500 bp DWS|ornithine decarboxylase

GCTCCTTCTTGTCGCGGCTGTGTGGGTTCGCTCATTCTCGGGGTGTTGTCCCCCTTCCCTCGTGGCATCGTCATCGTCTTCTCCACCGCCACCGTCCACGCTGCACACGCACACTCCACAGCGTACTTCACCTTCCTAGCTGTCTCCCTCACTCCCTCTCTGTAACCCTAACTGTCGTTGCGGCTACCGCTCTGCGTCCTTTCCGGCAACTTCTCCTGCGGTTCACTGCCAGCGTTGCTCCTACCTGTGGTGCCCCGTTCCTCTGGCCCCTCACACACACACACACTCACCTGCACTCTCTCAGTCTCCTTTGTCAACCGCTGGAGCTCAGCGCATTCGCTTTTTCGGCCGCCGCTTCGCCTCCATCTCGCGCCGCCGCACCTCCTCGCACCTCCCTCCTCCCTCGCCCCGCGCGCGCCGGCCTCTCTCTGCATTCCGCACAGACCTGCCAAGGCGCTCTCCCGTGTGTGTCGTCGCCCGCCGTCCTTCCCAAGTGCTCAATGGGTGATCATGACGTCGCTCTCTGCCACGTCAGCCGCTACAACCATGCGAATTACTGGGCCTTTGTGCCGCTGCCAACGGTCAGCGACGACACGGGATGCGACAGTCTGCATCATGACAGCGCTTCCAAAAGGATAAGGATGGCGCCGCCGGCGTCCGCGTCGAAGGCGGGCGCGGCCGAGGAACGCCTGCACCCCTACGAAAGGCGTCTATTGGATCAGTACCAGATCCACCTCCAACCCGCCAACCGCAACCCGCTGAGCCGAGCCGATTCCGCCGCCGGTCGAGAGGAAACGGCGCAGACCCCCGCGCAGGTGCAGATGGTGTCTGGTGTTGCTGTTGCGGACTCGACTTCAGATCAGCACGCCTCTGTTGCATCCAGTCAGGACCTCGTCGACCTCTTCTTCCTGAGGGCAGCCAGGCCGTCGACGGTCTGTGCTTCTCGCCCTACCCCATCTACGGTTGGCGCACGGCCGAGGAGCGCCGGGCTGCCGTTTGTGAGGTTTTCAAGACGTACAACGTGGTCACGCGCCTGCCCGCCTCGCCGGCTGCCCTGGCCGCCGCGCAGCGCCGCTACAGCCGCCACCGGCACTCCGCCATCGCCCCCATCAACAAGAGTGCGATCGAGACGCGTGAGCAGTACTGGCGCCGCCTGTCGAACCTGTACACCCAGAAGGGTGTAAAAGATGCTGCTTCTGCCGCCGACGCTGCGGCTACGACGGCGACGAACGGTGCCGTCCCCGCTGCCCCCGCCTACGAGCCCGAGGATCCATTCTACATCATCGATCTCGGCCGTGTTGTGGAGCAGATGGCGCGCTGGCGTCACGAGCTGCCGATGGTGCGCCCTTACTTTGCCGTGAAAAGCAACCCGCAACCGGCTGTTTTGGAGGTGCTCAGCGCCCTCGGCGCCGGTTTCGACTGCGCGTCCAAGGAAGAAATACATATGGTGCTAGGTCACCAGCTCGTGGCGTCGCCGGACGACATCATCTTCGCCAACCCGTGCAAGCAGCTCGGCGACCTGCGTGAGGCGCAGGCGTGCGGCGTGACCTACGTGACGGTGGACAACCCGCTGGAGATGGAAAAGATCAGTCGCCTGATGCCCTCCGCCCACGCGATTATCCGCATCAAGACGAACGACAGCAAGGCCCAGTGCTCCTTTTCCACCAAGTTCGGTGCCCCCCTTGAAGATGTCGAGGGCCTACTGGAGGCGGCCCGCCAGTTCAACGTCACCGTGTGCGGCGTCAGCTTTCACGTCGGCAGCGGCAACGACGACCAGTCTGCGTACGTGTCCGCGGTACGCGATGCCTACCAAGTCTTTCAGCAGGCGGTCCAGTACGGCTTCAAGTGCACCATCCTCGACATCGGCGGCGGCTTCCCGGGCACGGAGGTCGTCGAGGGCAGCGGCAATACCTCTTTCGAGGCCATTGCCCGTACTATTCGACCGGTGCTGGCGGAGCTCTTCGGCGGCGGTGATGTCACCATCATCAGTGAGCCCGGCCGCTACTTCACGGCTGCCTCGCACGCGCTTCTCATGAACGTATTCGCCTCCCGCACGCTGCGACTGTCCGATGTCGAGGTGAGTCGCCAAGCCTTCCAGTCCGTCGTGTCGATGGACGAGCCCGAAGAGTACCAGTACTACGTCAACGACGGCCTCTACCACAGCTTCAACTGCATCCTCTTCGATCACGCCCACCCAACGCTTCTCCTGCTAAACGACGGCGACGGCGCTGACGGGGTGGAGAGTGGCACGGAGGCGGCAGCGGTGTGCAGCGAGGAGGAGGGCGAGACGTCGCTGAGCGGTCCCCTGGCGAACGATCCACTGTTCATGTCCGCCTGGGACCGCCGTCGCAGCTTCGCGCGCCGCCCGCTGCGCATCACAACCATCTTCGGCCCCACCTGCGATTCGATGGACTGCATCCTGAAGAAGCAGCCCTTCCCGGAGATGAAGCTGGGCGACTGGCTTCTGGTGCCAGACATGGGCAGCTACACCACCGCCGCCGCCGGCTTCTTCAACGGTTTCGCGACGCGCCGCCTTGAGTGGGTGAGCTCTGTGGACTTGTGCGCGAGGCCGAGGCCTGTGTATACGCGTGAGGGCAACACGCTGAGGTGTGTGAGCGAGTGAGAGAGGGACAGAAAGGAGACCCGGTGCTGTAGGGTGTGCTCGTCCATCCCACAAGAGGATGGACGGTCTCTTCGCTCCGGCCACCTCACCACCTCCTTTAGCCGCCCCCTCCTCCGCCCCCTCTCCCCCTATCGTCCCCTTTTCTGTGGTGGTTGCACTTGTATTCGTGTTGGATGCACGGCGACGCTGCTCGCTTGTGTTTGTGCGCCTGCGCCTGCGCGCGTGTTTCGCTTTTCGTTCACCCCCTGTGGTCCCTTCGTGAGCCTCCCCATCCCCGCTCTTCTTCGCCGCTGGCTTCGTTGTTGCTCTTCTCTGACGCCTACGCAACGCAAGCCCCCCCCCTCTCCCTCACCCTTGTGCCCCACAAACTCCCACTGACAGTGCTTCCACTTACTAAGTCGGCCCAACCCCAGCGAAGCCCCCTCGCCGCCGCTGCTGCCGCCGACCCCTTCTCTCTCTTGCCTCGTGTCTCTCCTGCTCCTTTTCTCTTGTCGTGTACA

>BPK085/0 clone 8|LinJ.12.0100|500 bp UPS + CDS + 500 bp DWS|ornithine decarboxylase

GCTCCTTCTTGTCGCGGCTGTGTGGGTTCGCTCATTCTCGGGGTGTTGTCCCCCTTCCCTCGTGGCATCGTCATCGTCTTCTCCACCGCCACCGTCCACGCTGCACACGCACACTCCACAGCGTACTTCACCTTCCTAGCTGTCTCCCTCACTCCCTCTCTGTAACCCTAACTGTCGTTGCGGCTACCGCTCTGCGTCCTTTCCGGCAACTTCTCCTGCGGTTCACTGCCAGCGTTGCTCCTACCTGTGGTGCCCCGTTCCTCTGGCCCCTCACACACACACACACTCACCTGCACTCTCTCAGTCTCCTTTGTCAACCGCTGGAGCTCAGCGCATTCGCTTTTTCGGCCGCCGCTTCGCCTCCATCTCGCGCCGCCGCACCTCCTCGCACCTCCCTCCTCCCTCGCCCCGCGCGCGCCGGCCTCTCTCTGCATTCCGCACAGACCTGCCAAGGCGCTCTCCCGTGTGTGTCGTCGCCCGCCGTCCTTCCCAAGTGCTCAATGGGTGATCATGACGTCGCTCTCTGCCACGTCAGCCGCTACAACCATGCGAATTACTGGGCCTTTGTGCCGCTGCCAACGGTCAGCGACGACACGGGATGCGACAGTCTGCATCATGACAGCGCTTCCAAAAGGATAAGGATGGCGCCGCCGGCGTCCGCGTCGAAGGCGGGCGCGGCCGAGGAACGCCTGCACCCCTACGAAAGGCGTCTATTGGATCAGTACCAGATCCACCTCCAACCCGCCAACCGCAACCCGCTGAGCCGAGCCGATTCCGCCGCCGGTCGAGAGGAAACGGCGCAGACCCCCGCGCAGGTGCAGATGGTGTCTGGTGTTGCTGTTGCGGACTCGACTTCAGATCAGCACGCCTCTGTTGCATCCAGTCAGGACCTCGTCGACCTCTTCTTCCTGAGGGCAGCCAGGCCGTCGACGGTCTGTGCTTCTCGCCCTACCCCATCTACGGTTGGCGCACGGCCGAGGAGCGCCGGGCTGCCGTTTGTGAGGTTTTCAAGACGTACAACGTGGTCACGCGCCTGCCCGCCTCGCCGGCTGCCCTGGCCGCCGCGCAGCGCCGCTACAGCCGCCACCGGCACTCCGCCATCGCCCCCATCAACAAGAGTGCGATCGAGACGCGTGAGCAGTACTGGCGCCGCCTGTCGAACCTGTACACCCAGAAGGGTGTAAAAGATGCTGCTTCTGCCGCCGACGCTGCGGCTACGACGGCGACGAACGGTGCCGTCCCCGCTGCCCCCGCCTACGAGCCCGAGGATCCATTCTACATCATCGATCTCGGCCGTGTTGTGGAGCAGATGGCGCGCTGGCGTCACGAGCTGCCGATGGTGCGCCCTTACTTTGCCGTGAAAAGCAACCCGCAACCGGCTGTTTTGGAGGTGCTCAGCGCCCTCGGCGCCGGTTTCGACTGCGCGTCCAAGGAAGAAATACATATGGTGCTAGGTCACCAGCTCGTGGCGTCGCCGGACGACATCATCTTCGCCAACCCGTGCAAGCAGCTCGGCGACCTGCGTGAGGCGCAGGCGTGCGGCGTGACCTACGTGACGGTGGACAACCCGCTGGAGATGGAAAAGATCAGTCGCCTGATGCCCTCCGCCCACGCGATTATCCGCATCAAGACGAACGACAGCAAGGCCCAGTGCTCCTTTTCCACCAAGTTCGGTGCCCCCCTTGAAGATGTCGAGGGCCTACTGGAGGCGGCCCGCCAGTTCAACGTCACCGTGTGCGGCGTCAGCTTTCACGTCGGCAGCGGCAACGACGACCAGTCTGCGTACGTGTCCGCGGTACGCGATGCCTACCAAGTCTTTCAGCAGGCGGTCCAGTACGGCTTCAAGTGCACCATCCTCGACATCGGCGGCGGCTTCCCGGGCACGGAGGTCGTCGAGGGCAGCGGCAATACCTCTTTCGAGGCCATTGCCCGTACTATTCGACCGGTGCTGGCGGAGCTCTTCGGCGGCGGTGATGTCACCATCATCAGTGAGCCCGGCCGCTACTTCACGGCTGCCTCGCACGCGCTTCTCATGAACGTATTCGCCTCCCGCACGCTGCGACTGTCCGATGTCGAGGTGAGTCGCCAAGCCTTCCAGTCCGTCGTGTCGATGGACGAGCCCGAAGAGTACCAGTACTACGTCAACGACGGCCTCTACCACAGCTTCAACTGCATCCTCTTCGATCACGCCCACCCAACGCTTCTCCTGCTAAACGACGGCGACGGCGCTGACGGGGTGGAGAGTGGCACGGAGGCGGCAGCGGTGTGCAGCGAGGAGGAGGGCGAGACGTCGCTGAGCGGTCCCCTGGCGAACGATCCACTGTTCATGTCCGCCTGGGACCGCCGTCGCAGCTTCGCGCGCCGCCCGCTGCGCATCACAACCATCTTCGGCCCCACCTGCGATTCGATGGACTGCATCCTGAAGAAGCAGCCCTTCCCGGAGATGAAGCTGGGCGACTGGCTTCTGGTGCCAGACATGGGCAGCTACACCACCGCCGCCGCCGGCTTCTTCAACGGTTTCGCGACGCGCCGCCTTGAGTGGGTGAGCTCTGTGGACTTGTGCGCGAGGCCGAGGCCTGTGTATACGCGTGAGGGCAACACGCTGAGGTGTGTGAGCGAGTGAGAGAGGGACAGAAAGGAGACCCGGTGCTGTAGGGTGTGCTCGTCCATCCCACAAGAGGATGGACGGTCTCTTCGCTCCGGCCACCTCACCACCTCCTTTAGCCGCCCCCTCCTCCGCCCCCTCTCCCCCTATCGTCCCCTTTTCTGTGGTGGTTGCACTTGTATTCGTGTTGGATGCACGGCGACGCTGCTCGCTTGTGTTTGTGCGCCTGCGCCTGCGCGCGTGTTTCGCTTTTCGTTCACCCCCTGTGGTCCCTTCGTGAGCCTCCCCATCCCCGCTCTTCTTCGCCGCTGGCTTCGTTGTTGCTCTTCTCTGACGCCTACGCAACGCAAGCCCCCCCCCTCTCCCTCACCCTTGTGCCCCACAAACTCCCACTGACAGTGCTTCCACTTACTAAGTCGGCCCAACCCCAGCGAAGCCCCCTCGCCGCCGCTGCTGCCGCCGACCCCTTCTCTCTCTTGCCTCGTGTCTCTCCTGCTCCTTTTCTCTTGTCGTGTACA

>BPK087/0 clone 11|LinJ.12.0100|500 bp UPS + CDS + 500 bp DWS|ornithine decarboxylase

GCTCCTTCTTGTCGCGGCTGTGTGGGTTCGCTCATTCTCGGGGTGTTGTCCCCCTTCCCTCGTGGCATCGTCATCGTCTTCTCCACCGCCACCGTCCACGCTGCACACGCACACTCCACAGCGTACTTCACCTTCCTAGCTGTCTCCCTCACTCCCTCTCTGTAACCCTAACTGTCGTTGCGGCTACCGCTCTGCGTCCTTTCCGGCAACTTCTCCTGCGGTTCACTGCCAGCGTTGCTCCTACCTGTGGTGCCCCGTTCCTCTGGCCCCTCACACACACACACACTCACCTGCACTCTCTCAGTCTCCTTTGTCAACCGCTGGAGCTCAGCGCATTCGCTTTTTCGGCCGCCGCTTCGCCTCCATCTCGCGCCGCCGCACCTCCTCGCACCTCCCTCCTCCCTCGCCCCGCGCGCGCCGGCCTCTCTCTGCATTCCGCACAGACCTGCCAAGGCGCTCTCCCGTGTGTGTCGTCGCCCGCCGTCCTTCCCAAGTGCTCAATGGGTGATCATGACGTCGCTCTCTGCCACGTCAGCCGCTACAACCATGCGAATTACTGGGCCTTTGTGCCGCTGCCAACGGTCAGCGACGACACGGGATGCGACAGTCTGCATCATGACAGCGCTTCCAAAAGGATAAGGATGGCGCCGCCGGCGTCCGCGTCGAAGGCGGGCGCGGCCGAGGAACGCCTGCACCCCTACGAAAGGCGTCTATTGGATCAGTACCAGATCCACCTCCAACCCGCCAACCGCAACCCGCTGAGCCGAGCCGATTCCGCCGCCGGTCGAGAGGAAACGGCGCAGACCCCCGCGCAGGTGCAGATGGTGTCTGGTGTTGCTGTTGCGGACTCGACTTCAGATCAGCACGCCTCTGTTGCATCCAGTCAGGACCTCGTCGACCTCTTCTTCCTGAGGGCAGCCAGGCCGTCGACGGTCTGTGCTTCTCGCCCTACCCCATCTACGGTTGGCGCACGGCCGAGGAGCGCCGGGCTGCCGTTTGTGAGGTTTTCAAGACGTACAACGTGGTCACGCGCCTGCCCGCCTCGCCGGCTGCCCTGGCCGCCGCGCAGCGCCGCTACAGCCGCCACCGGCACTCCGCCATCGCCCCCATCAACAAGAGTGCGATCGAGACGCGTGAGCAGTACTGGCGCCGCCTGTCGAACCTGTACACCCAGAAGGGTGTAAAAGATGCTGCTTCTGCCGCCGACGCTGCGGCTACGACGGCGACGAACGGTGCCGTCCCCGCTGCCCCCGCCTACGAGCCCGAGGATCCATTCTACATCATCGATCTCGGCCGTGTTGTGGAGCAGATGGCGCGCTGGCGTCACGAGCTGCCGATGGTGCGCCCTTACTTTGCCGTGAAAAGCAACCCGCAACCGGCTGTTTTGGAGGTGCTCAGCGCCCTCGGCGCCGGTTTCGACTGCGCGTCCAAGGAAGAAATACATATGGTGCTAGGTCACCAGCTCGTGGCGTCGCCGGACGACATCATCTTCGCCAACCCGTGCAAGCAGCTCGGCGACCTGCGTGAGGCGCAGGCGTGCGGCGTGACCTACGTGACGGTGGACAACCCGCTGGAGATGGAAAAGATCAGTCGCCTGATGCCCTCCGCCCACGCGATTATCCGCATCAAGACGAACGACAGCAAGGCCCAGTGCTCCTTTTCCACCAAGTTCGGTGCCCCCCTTGAAGATGTCGAGGGCCTACTGGAGGCGGCCCGCCAGTTCAACGTCACCGTGTGCGGCGTCAGCTTTCACGTCGGCAGCGGCAACGACGACCAGTCTGCGTACGTGTCCGCGGTACGCGATGCCTACCAAGTCTTTCAGCAGGCGGTCCAGTACGGCTTCAAGTGCACCATCCTCGACATCGGCGGCGGCTTCCCGGGCACGGAGGTCGTCGAGGGCAGCGGCAATACCTCTTTCGAGGCCATTGCCCGTACTATTCGACCGGTGCTGGCGGAGCTCTTCGGCGGCGGTGATGTCACCATCATCAGTGAGCCCGGCCGCTACTTCACGGCTGCCTCGCACGCGCTTCTCATGAACGTATTCGCCTCCCGCACGCTGCGACTGTCCGATGTCGAGGTGAGTCGCCAAGCCTTCCAGTCCGTCGTGTCGATGGACGAGCCCGAAGAGTACCAGTACTACGTCAACGACGGCCTCTACCACAGCTTCAACTGCATCCTCTTCGATCACGCCCACCCAACGCTTCTCCTGCTAAACGACGGCGACGGCGCTGACGGGGTGGAGAGTGGCACGGAGGCGGCAGCGGTGTGCAGCGAGGAGGAGGGCGAGACGTCGCTGAGCGGTCCCCTGGCGAACGATCCACTGTTCATGTCCGCCTGGGACCGCCGTCGCAGCTTCGCGCGCCGCCCGCTGCGCATCACAACCATCTTCGGCCCCACCTGCGATTCGATGGACTGCATCCTGAAGAAGCAGCCCTTCCCGGAGATGAAGCTGGGCGACTGGCTTCTGGTGCCAGACATGGGCAGCTACACCACCGCCGCCGCCGGCTTCTTCAACGGTTTCGCGACGCGCCGCCTTGAGTGGGTGAGCTCTGTGGACTTGTGCGCGAGGCCGAGGCCTGTGTATACGCGTGAGGGCAACACGCTGAGGTGTGTGAGCGAGTGAGAGAGGGACAGAAAGGAGACCCGGTGCTGTAGGGTGTGCTCGTCCATCCCACAAGAGGATGGACGGTCTCTTCGCTCCGGCCACCTCACCACCTCCTTTAGCCGCCCCCTCCTCCGCCCCCTCTCCCCCTATCGTCCCCTTTTCTGTGGTGGTTGCACTTGTATTCGTGTTGGATGCACGGCGACGCTGCTCGCTTGTGTTTGTGCGCCTGCGCCTGCGCGCGTGTTTCGCTTTTCGTTCACCCCCTGTGGTCCCTTCGTGAGCCTCCCCATCCCCGCTCTTCTTCGCCGCTGGCTTCGTTGTTGCTCTTCTCTGACGCCTACGCAACGCAAGCCCCCCCCCTCTCCCTCACCCTTGTGCCCCACAAACTCCCACTGACAGTGCTTCCACTTACTAAGTCGGCCCAACCCCAGCGAAGCCCCCTCGCCGCCGCTGCTGCCGCCGACCCCTTCTCTCTCTTGCCTCGTGTCTCTCCTGCTCCTTTTCTCTTGTCGTGTACA

>BPK178/0 clone 3|LinJ.12.0100|500 bp UPS + CDS + 500 bp DWS|ornithine decarboxylase

GCTCCTTCTTGTCGCGGCTGTGTGGGTTCGCTCATTCTCGGGGTGTTGTCCCCCTTCCCTCGTGGCATCGTCATCGTCTTCTCCACCGCCACCGTCCACGCTGCACACGCACACTCCACAGCGTACTTCACCTTCCTAGCTGTCTCCCTCACTCCCTCTCTGTAACCCTAACTGTCGTTGCGGCTACCGCTCTGCGTCCTTTCCGGCAACTTCTCCTGCGGTTCACTGCCAGCGTTGCTCCTACCTGTGGTGCCCCGTTCCTCTGGCCCCTCACACACACACACACTCACCTGCACTCTCTCAGTCTCCTTTGTCAACCGCTGGAGCTCAGCGCATTCGCTTTTTCGGCCGCCGCTTCGCCTCCATCTCGCGCCGCCGCACCTCCTCGCACCTCCCTCCTCCCTCGCCCCGCGCGCGCCGGCCTCTCTCTGCATTCCGCACAGACCTGCCAAGGCGCTCTCCCGTGTGTGTCGTCGCCCGCCGTCCTTCCCAAGTGCTCAATGGGTGATCATGACGTCGCTCTCTGCCACGTCAGCCGCTACAACCATGCGAATTACTGGGCCTTTGTGCCGCTGCCAACGGTCAGCGACGACACGGGATGCGACAGTCTGCATCATGACAGCGCTTCCAAAAGGATAAGGATGGCGCCGCCGGCGTCCGCGTCGAAGGCGGGCGCGGCCGAGGAACGCCTGCACCCCTACGAAAGGCGTCTATTGGATCAGTACCAGATCCACCTCCAACCCGCCAACCGCAACCCGCTGAGCCGAGCCGATTCCGCCGCCGGTCGAGAGGAAACGGCGCAGACCCCCGCGCAGGTGCAGATGGTGTCTGGTGTTGCTGTTGCGGACTCGACTTCAGATCAGCACGCCTCTGTTGCATCCAGTCAGGACCTCGTCGACCTCTTCTTCCTGAGGGCAGCCAGGCCGTCGACGGTCTGTGCTTCTCGCCCTACCCCATCTACGGTTGGCGCACGGCCGAGGAGCGCCGGGCTGCCGTTTGTGAGGTTTTCAAGACGTACAACGTGGTCACGCGCCTGCCCGCCTCGCCGGCTGCCCTGGCCGCCGCGCAGCGCCGCTACAGCCGCCACCGGCACTCCGCCATCGCCCCCATCAACAAGAGTGCGATCGAGACGCGTGAGCAGTACTGGCGCCGCCTGTCGAACCTGTACACCCAGAAGGGTGTAAAAGATGCTGCTTCTGCCGCCGACGCTGCGGCTACGACGGCGACGAACGGTGCCGTCCCCGCTGCCCCCGCCTACGAGCCCGAGGATCCATTCTACATCATCGATCTCGGCCGTGTTGTGGAGCAGATGGCGCGCTGGCGTCACGAGCTGCCGATGGTGCGCCCTTACTTTGCCGTGAAAAGCAACCCGCAACCGGCTGTTTTGGAGGTGCTCAGCGCCCTCGGCGCCGGTTTCGACTGCGCGTCCAAGGAAGAAATACATATGGTGCTAGGTCACCAGCTCGTGGCGTCGCCGGACGACATCATCTTCGCCAACCCGTGCAAGCAGCTCGGCGACCTGCGTGAGGCGCAGGCGTGCGGCGTGACCTACGTGACGGTGGACAACCCGCTGGAGATGGAAAAGATCAGTCGCCTGATGCCCTCCGCCCACGCGATTATCCGCATCAAGACGAACGACAGCAAGGCCCAGTGCTCCTTTTCCACCAAGTTCGGTGCCCCCCTTGAAGATGTCGAGGGCCTACTGGAGGCGGCCCGCCAGTTCAACGTCACCGTGTGCGGCGTCAGCTTTCACGTCGGCAGCGGCAACGACGACCAGTCTGCGTACGTGTCCGCGGTACGCGATGCCTACCAAGTCTTTCAGCAGGCGGTCCAGTACGGCTTCAAGTGCACCATCCTCGACATCGGCGGCGGCTTCCCGGGCACGGAGGTCGTCGAGGGCAGCGGCAATACCTCTTTCGAGGCCATTGCCCGTACTATTCGACCGGTGCTGGCGGAGCTCTTCGGCGGCGGTGATGTCACCATCATCAGTGAGCCCGGCCGCTACTTCACGGCTGCCTCGCACGCGCTTCTCATGAACGTATTCGCCTCCCGCACGCTGCGACTGTCCGATGTCGAGGTGAGTCGCCAAGCCTTCCAGTCCGTCGTGTCGATGGACGAGCCCGAAGAGTACCAGTACTACGTCAACGACGGCCTCTACCACAGCTTCAACTGCATCCTCTTCGATCACGCCCACCCAACGCTTCTCCTGCTAAACGACGGCGACGGCGCTGACGGGGTGGAGAGTGGCACGGAGGCGGCAGCGGTGTGCAGCGAGGAGGAGGGCGAGACGTCGCTGAGCGGTCCCCTGGCGAACGATCCACTGTTCATGTCCGCCTGGGACCGCCGTCGCAGCTTCGCGCGCCGCCCGCTGCGCATCACAACCATCTTCGGCCCCACCTGCGATTCGATGGACTGCATCCTGAAGAAGCAGCCCTTCCCGGAGATGAAGCTGGGCGACTGGCTTCTGGTGCCAGACATGGGCAGCTACACCACCGCCGCCGCCGGCTTCTTCAACGGTTTCGCGACGCGCCGCCTTGAGTGGGTGAGCTCTGTGGACTTGTGCGCGAGGCCGAGGCCTGTGTATACGCGTGAGGGCAACACGCTGAGGTGTGTGAGCGAGTGAGAGAGGGACAGAAAGGAGACCCGGTGCTGTAGGGTGTGCTCGTCCATCCCACAAGAGGATGGACGGTCTCTTCGCTCCGGCCACCTCACCACCTCCTTTAGCCGCCCCCTCCTCCGCCCCCTCTCCCCCTATCGTCCCCTTTTCTGTGGTGGTTGCACTTGTATTCGTGTTGGATGCACGGCGACGCTGCTCGCTTGTGTTTGTGCGCCTGCGCCTGCGCGCGTGTTTCGCTTTTCGTTCACCCCCTGTGGTCCCTTCGTGAGCCTCCCCATCCCCGCTCTTCTTCGCCGCTGGCTTCGTTGTTGCTCTTCTCTGACGCCTACGCAACGCAAGCCCCCCCCCTCTCCCTCACCCTTGTGCCCCACAAACTCCCACTGACAGTGCTTCCACTTACTAAGTCGGCCCAACCCCAGCGAAGCCCCCTCGCCGCCGCTGCTGCCGCCGACCCCTTCTCTCTCTTGCCTCGTGTCTCTCCTGCTCCTTTTCTCTTGTCGTGTACA

>BPK190/0 clone 3|LinJ.12.0100|500 bp UPS + CDS + 500 bp DWS|ornithine decarboxylase

GCTCCTTCTTGTCGCGGCTGTGTGGGTTCGCTCATTCTCGGGGTGTTGTCCCCCTTCCCTCGTGGCATCGTCATCGTCTTCTCCACCGCCACCGTCCACGCTGCACACGCACACTCCACAGCGTACTTCACCTTCCTAGCTGTCTCCCTCACTCCCTCTCTGTAACCCTAACTGTCGTTGCGGCTACCGCTCTGCGTCCTTTCCGGCAACTTCTCCTGCGGTTCACTGCCAGCGTTGCTCCTACCTGTGGTGCCCCGTTCCTCTGGCCCCTCACACACACACACACTCACCTGCACTCTCTCAGTCTCCTTTGTCAACCGCTGGAGCTCAGCGCATTCGCTTTTTCGGCCGCCGCTTCGCCTCCATCTCGCGCCGCCGCACCTCCTCGCACCTCCCTCCTCCCTCGCCCCGCGCGCGCCGGCCTCTCTCTGCATTCCGCACAGACCTGCCAAGGCGCTCTCCCGTGTGTGTCGTCGCCCGCCGTCCTTCCCAAGTGCTCAATGGGTGATCATGACGTCGCTCTCTGCCACGTCAGCCGCTACAACCATGCGAATTACTGGGCCTTTGTGCCGCTGCCAACGGTCAGCGACGACACGGGATGCGACAGTCTGCATCATGACAGCGCTTCCAAAAGGATAAGGATGGCGCCGCCGGCGTCCGCGTCGAAGGCGGGCGCGGCCGAGGAACGCCTGCACCCCTACGAAAGGCGTCTATTGGATCAGTACCAGATCCACCTCCAACCCGCCAACCGCAACCCGCTGAGCCGAGCCGATTCCGCCGCCGGTCGAGAGGAAACGGCGCAGACCCCCGCGCAGGTGCAGATGGTGTCTGGTGTTGCTGTTGCGGACTCGACTTCAGATCAGCACGCCTCTGTTGCATCCAGTCAGGACCTCGTCGACCTCTTCTTCCTGAGGGCAGCCAGGCCGTCGACGGTCTGTGCTTCTCGCCCTACCCCATCTACGGTTGGCGCACGGCCGAGGAGCGCCGGGCTGCCGTTTGTGAGGTTTTCAAGACGTACAACGTGGTCACGCGCCTGCCCGCCTCGCCGGCTGCCCTGGCCGCCGCGCAGCGCCGCTACAGCCGCCACCGGCACTCCGCCATCGCCCCCATCAACAAGAGTGCGATCGAGACGCGTGAGCAGTACTGGCGCCGCCTGTCGAACCTGTACACCCAGAAGGGTGTAAAAGATGCTGCTTCTGCCGCCGACGCTGCGGCTACGACGGCGACGAACGGTGCCGTCCCCGCTGCCCCCGCCTACGAGCCCGAGGATCCATTCTACATCATCGATCTCGGCCGTGTTGTGGAGCAGATGGCGCGCTGGCGTCACGAGCTGCCGATGGTGCGCCCTTACTTTGCCGTGAAAAGCAACCCGCAACCGGCTGTTTTGGAGGTGCTCAGCGCCCTCGGCGCCGGTTTCGACTGCGCGTCCAAGGAAGAAATACATATGGTGCTAGGTCACCAGCTCGTGGCGTCGCCGGACGACATCATCTTCGCCAACCCGTGCAAGCAGCTCGGCGACCTGCGTGAGGCGCAGGCGTGCGGCGTGACCTACGTGACGGTGGACAACCCGCTGGAGATGGAAAAGATCAGTCGCCTGATGCCCTCCGCCCACGCGATTATCCGCATCAAGACGAACGACAGCAAGGCCCAGTGCTCCTTTTCCACCAAGTTCGGTGCCCCCCTTGAAGATGTCGAGGGCCTACTGGAGGCGGCCCGCCAGTTCAACGTCACCGTGTGCGGCGTCAGCTTTCACGTCGGCAGCGGCAACGACGACCAGTCTGCGTACGTGTCCGCGGTACGCGATGCCTACCAAGTCTTTCAGCAGGCGGTCCAGTACGGCTTCAAGTGCACCATCCTCGACATCGGCGGCGGCTTCCCGGGCACGGAGGTCGTCGAGGGCAGCGGCAATACCTCTTTCGAGGCCATTGCCCGTACTATTCGACCGGTGCTGGCGGAGCTCTTCGGCGGCGGTGATGTCACCATCATCAGTGAGCCCGGCCGCTACTTCACGGCTGCCTCGCACGCGCTTCTCATGAACGTATTCGCCTCCCGCACGCTGCGACTGTCCGATGTCGAGGTGAGTCGCCAAGCCTTCCAGTCCGTCGTGTCGATGGACGAGCCCGAAGAGTACCAGTACTACGTCAACGACGGCCTCTACCACAGCTTCAACTGCATCCTCTTCGATCACGCCCACCCAACGCTTCTCCTGCTAAACGACGGCGACGGCGCTGACGGGGTGGAGAGTGGCACGGAGGCGGCAGCGGTGTGCAGCGAGGAGGAGGGCGAGACGTCGCTGAGCGGTCCCCTGGCGAACGATCCACTGTTCATGTCCGCCTGGGACCGCCGTCGCAGCTTCGCGCGCCGCCCGCTGCGCATCACAACCATCTTCGGCCCCACCTGCGATTCGATGGACTGCATCCTGAAGAAGCAGCCCTTCCCGGAGATGAAGCTGGGCGACTGGCTTCTGGTGCCAGACATGGGCAGCTACACCACCGCCGCCGCCGGCTTCTTCAACGGTTTCGCGACGCGCCGCCTTGAGTGGGTGAGCTCTGTGGACTTGTGCGCGAGGCCGAGGCCTGTGTATACGCGTGAGGGCAACACGCTGAGGTGTGTGAGCGAGTGAGAGAGGGACAGAAAGGAGACCCGGTGCTGTAGGGTGTGCTCGTCCATCCCACAAGAGGATGGACGGTCTCTTCGCTCCGGCCACCTCACCACCTCCTTTAGCCGCCCCCTCCTCCGCCCCCTCTCCCCCTATCGTCCCCTTTTCTGTGGTGGTTGCACTTGTATTCGTGTTGGATGCACGGCGACGCTGCTCGCTTGTGTTTGTGCGCCTGCGCCTGCGCGCGTGTTTCGCTTTTCGTTCACCCCCTGTGGTCCCTTCGTGAGCCTCCCCATCCCCGCTCTTCTTCGCCGCTGGCTTCGTTGTTGCTCTTCTCTGACGCCTACGCAACGCAAGCCCCCCCCCTCTCCCTCACCCTTGTGCCCCACAAACTCCCACTGACAGTGCTTCCACTTACTAAGTCGGCCCAACCCCAGCGAAGCCCCCTCGCCGCCGCTGCTGCCGCCGACCCCTTCTCTCTCTTGCCTCGTGTCTCTCCTGCTCCTTTTCTCTTGTCGTGTACA

>BPK206/0 clone 10|LinJ.12.0100|500 bp UPS + CDS + 500 bp DWS|ornithine decarboxylase

GCTCCTTCTTGTCGCGGCTGTGTGGGTTCGCTCATTCTCGGGGTGTTGTCCCCCTTCCCTCGTGGCATCGTCATCGTCTTCTCCACCGCCACCGTCCACGCTGCACACGCACACTCCACAGCGTACTTCACCTTCCTAGCTGTCTCCCTCACTCCCTCTCTGTAACCCTAACTGTCGTTGCGGCTACCGCTCTGCGTCCTTTCCGGCAACTTCTCCTGCGGTTCACTGCCAGCGTTGCTCCTACCTGTGGTGCCCCGTTCCTCTGGCCCCTCACACACACACACACTCACCTGCACTCTCTCAGTCTCCTTTGTCAACCGCTGGAGCTCAGCGCATTCGCTTTTTCGGCCGCCGCTTCGCCTCCATCTCGCGCCGCCGCACCTCCTCGCACCTCCCTCCTCCCTCGCCCCGCGCGCGCCGGCCTCTCTCTGCATTCCGCACAGACCTGCCAAGGCGCTCTCCCGTGTGTGTCGTCGCCCGCCGTCCTTCCCAAGTGCTCAATGGGTGATCATGACGTCGCTCTCTGCCACGTCAGCCGCTACAACCATGCGAATTACTGGGCCTTTGTGCCGCTGCCAACGGTCAGCGACGACACGGGATGCGACAGTCTGCATCATGACAGCGCTTCCAAAAGGATAAGGATGGCGCCGCCGGCGTCCGCGTCGAAGGCGGGCGCGGCCGAGGAACGCCTGCACCCCTACGAAAGGCGTCTATTGGATCAGTACCAGATCCACCTCCAACCCGCCAACCGCAACCCGCTGAGCCGAGCCGATTCCGCCGCCGGTCGAGAGGAAACGGCGCAGACCCCCGCGCAGGTGCAGATGGTGTCTGGTGTTGCTGTTGCGGACTCGACTTCAGATCAGCACGCCTCTGTTGCATCCAGTCAGGACCTCGTCGACCTCTTCTTCCTGAGGGCAGCCAGGCCGTCGACGGTCTGTGCTTCTCGCCCTACCCCATCTACGGTTGGCGCACGGCCGAGGAGCGCCGGGCTGCCGTTTGTGAGGTTTTCAAGACGTACAACGTGGTCACGCGCCTGCCCGCCTCGCCGGCTGCCCTGGCCGCCGCGCAGCGCCGCTACAGCCGCCACCGGCACTCCGCCATCGCCCCCATCAACAAGAGTGCGATCGAGACGCGTGAGCAGTACTGGCGCCGCCTGTCGAACCTGTACACCCAGAAGGGTGTAAAAGATGCTGCTTCTGCCGCCGACGCTGCGGCTACGACGGCGACGAACGGTGCCGTCCCCGCTGCCCCCGCCTACGAGCCCGAGGATCCATTCTACATCATCGATCTCGGCCGTGTTGTGGAGCAGATGGCGCGCTGGCGTCACGAGCTGCCGATGGTGCGCCCTTACTTTGCCGTGAAAAGCAACCCGCAACCGGCTGTTTTGGAGGTGCTCAGCGCCCTCGGCGCCGGTTTCGACTGCGCGTCCAAGGAAGAAATACATATGGTGCTAGGTCACCAGCTCGTGGCGTCGCCGGACGACATCATCTTCGCCAACCCGTGCAAGCAGCTCGGCGACCTGCGTGAGGCGCAGGCGTGCGGCGTGACCTACGTGACGGTGGACAACCCGCTGGAGATGGAAAAGATCAGTCGCCTGATGCCCTCCGCCCACGCGATTATCCGCATCAAGACGAACGACAGCAAGGCCCAGTGCTCCTTTTCCACCAAGTTCGGTGCCCCCCTTGAAGATGTCGAGGGCCTACTGGAGGCGGCCCGCCAGTTCAACGTCACCGTGTGCGGCGTCAGCTTTCACGTCGGCAGCGGCAACGACGACCAGTCTGCGTACGTGTCCGCGGTACGCGATGCCTACCAAGTCTTTCAGCAGGCGGTCCAGTACGGCTTCAAGTGCACCATCCTCGACATCGGCGGCGGCTTCCCGGGCACGGAGGTCGTCGAGGGCAGCGGCAATACCTCTTTCGAGGCCATTGCCCGTACTATTCGACCGGTGCTGGCGGAGCTCTTCGGCGGCGGTGATGTCACCATCATCAGTGAGCCCGGCCGCTACTTCACGGCTGCCTCGCACGCGCTTCTCATGAACGTATTCGCCTCCCGCACGCTGCGACTGTCCGATGTCGAGGTGAGTCGCCAAGCCTTCCAGTCCGTCGTGTCGATGGACGAGCCCGAAGAGTACCAGTACTACGTCAACGACGGCCTCTACCACAGCTTCAACTGCATCCTCTTCGATCACGCCCACCCAACGCTTCTCCTGCTAAACGACGGCGACGGCGCTGACGGGGTGGAGAGTGGCACGGAGGCGGCAGCGGTGTGCAGCGAGGAGGAGGGCGAGACGTCGCTGAGCGGTCCCCTGGCGAACGATCCACTGTTCATGTCCGCCTGGGACCGCCGTCGCAGCTTCGCGCGCCGCCCGCTGCGCATCACAACCATCTTCGGCCCCACCTGCGATTCGATGGACTGCATCCTGAAGAAGCAGCCCTTCCCGGAGATGAAGCTGGGCGACTGGCTTCTGGTGCCAGACATGGGCAGCTACACCACCGCCGCCGCCGGCTTCTTCAACGGTTTCGCGACGCGCCGCCTTGAGTGGGTGAGCTCTGTGGACTTGTGCGCGAGGCCGAGGCCTGTGTATACGCGTGAGGGCAACACGCTGAGGTGTGTGAGCGAGTGAGAGAGGGACAGAAAGGAGACCCGGTGCTGTAGGGTGTGCTCGTCCATCCCACAAGAGGATGGACGGTCTCTTCGCTCCGGCCACCTCACCACCTCCTTTAGCCGCCCCCTCCTCCGCCCCCTCTCCCCCTATCGTCCCCTTTTCTGTGGTGGTTGCACTTGTATTCGTGTTGGATGCACGGCGACGCTGCTCGCTTGTGTTTGTGCGCCTGCGCCTGCGCGCGTGTTTCGCTTTTCGTTCACCCCCTGTGGTCCCTTCGTGAGCCTCCCCATCCCCGCTCTTCTTCGCCGCTGGCTTCGTTGTTGCTCTTCTCTGACGCCTACGCAACGCAAGCCCCCCCCCTCTCCCTCACCCTTGTGCCCCACAAACTCCCACTGACAGTGCTTCCACTTACTAAGTCGGCCCAACCCCAGCGAAGCCCCCTCGCCGCCGCTGCTGCCGCCGACCCCTTCTCTCTCTTGCCTCGTGTCTCTCCTGCTCCTTTTCTCTTGTCGTGTACA

>BPK275/0 clone 18|LinJ.12.0100|500 bp UPS + CDS + 500 bp DWS|ornithine decarboxylase

GCTCCTTCTTGTCGCGGCTGTGTGGGTTCGCTCATTCTCGGGGTGTTGTCCCCCTTCCCTCGTGGCATCGTCATCGTCTTCTCCACCGCCACCGTCCACGCTGCACACGCACACTCCACAGCGTACTTCACCTTCCTAGCTGTCTCCCTCACTCCCTCTCTGTAACCCTAACTGTCGTTGCGGCTACCGCTCTGCGTCCTTTCCGGCAACTTCTCCTGCGGTTCACTGCCAGCGTTGCTCCTACCTGTGGTGCCCCGTTCCTCTGGCCCCTCACACACACACACACTCACCTGCACTCTCTCAGTCTCCTTTGTCAACCGCTGGAGCTCAGCGCATTCGCTTTTTCGGCCGCCGCTTCGCCTCCATCTCGCGCCGCCGCACCTCCTCGCACCTCCCTCCTCCCTCGCCCCGCGCGCGCCGGCCTCTCTCTGCATTCCGCACAGACCTGCCAAGGCGCTCTCCCGTGTGTGTCGTCGCCCGCCGTCCTTCCCAAGTGCTCAATGGGTGATCATGACGTCGCTCTCTGCCACGTCAGCCGCTACAACCATGCGAATTACTGGGCCTTTGTGCCGCTGCCAACGGTCAGCGACGACACGGGATGCGACAGTCTGCATCATGACAGCGCTTCCAAAAGGATAAGGATGGCGCCGCCGGCGTCCGCGTCGAAGGCGGGCGCGGCCGAGGAACGCCTGCACCCCTACGAAAGGCGTCTATTGGATCAGTACCAGATCCACCTCCAACCCGCCAACCGCAACCCGCTGAGCCGAGCCGATTCCGCCGCCGGTCGAGAGGAAACGGCGCAGACCCCCGCGCAGGTGCAGATGGTGTCTGGTGTTGCTGTTGCGGACTCGACTTCAGATCAGCACGCCTCTGTTGCATCCAGTCAGGACCTCGTCGACCTCTTCTTCCTGAGGGCAGCCAGGCCGTCGACGGTCTGTGCTTCTCGCCCTACCCCATCTACGGTTGGCGCACGGCCGAGGAGCGCCGGGCTGCCGTTTGTGAGGTTTTCAAGACGTACAACGTGGTCACGCGCCTGCCCGCCTCGCCGGCTGCCCTGGCCGCCGCGCAGCGCCGCTACAGCCGCCACCGGCACTCCGCCATCGCCCCCATCAACAAGAGTGCGATCGAGACGCGTGAGCAGTACTGGCGCCGCCTGTCGAACCTGTACACCCAGAAGGGTGTAAAAGATGCTGCTTCTGCCGCCGACGCTGCGGCTACGACGGCGACGAACGGTGCCGTCCCCGCTGCCCCCGCCTACGAGCCCGAGGATCCATTCTACATCATCGATCTCGGCCGTGTTGTGGAGCAGATGGCGCGCTGGCGTCACGAGCTGCCGATGGTGCGCCCTTACTTTGCCGTGAAAAGCAACCCGCAACCGGCTGTTTTGGAGGTGCTCAGCGCCCTCGGCGCCGGTTTCGACTGCGCGTCCAAGGAAGAAATACATATGGTGCTAGGTCACCAGCTCGTGGCGTCGCCGGACGACATCATCTTCGCCAACCCGTGCAAGCAGCTCGGCGACCTGCGTGAGGCGCAGGCGTGCGGCGTGACCTACGTGACGGTGGACAACCCGCTGGAGATGGAAAAGATCAGTCGCCTGATGCCCTCCGCCCACGCGATTATCCGCATCAAGACGAACGACAGCAAGGCCCAGTGCTCCTTTTCCACCAAGTTCGGTGCCCCCCTTGAAGATGTCGAGGGCCTACTGGAGGCGGCCCGCCAGTTCAACGTCACCGTGTGCGGCGTCAGCTTTCACGTCGGCAGCGGCAACGACGACCAGTCTGCGTACGTGTCCGCGGTACGCGATGCCTACCAAGTCTTTCAGCAGGCGGTCCAGTACGGCTTCAAGTGCACCATCCTCGACATCGGCGGCGGCTTCCCGGGCACGGAGGTCGTCGAGGGCAGCGGCAATACCTCTTTCGAGGCCATTGCCCGTACTATTCGACCGGTGCTGGCGGAGCTCTTCGGCGGCGGTGATGTCACCATCATCAGTGAGCCCGGCCGCTACTTCACGGCTGCCTCGCACGCGCTTCTCATGAACGTATTCGCCTCCCGCACGCTGCGACTGTCCGATGTCGAGGTGAGTCGCCAAGCCTTCCAGTCCGTCGTGTCGATGGACGAGCCCGAAGAGTACCAGTACTACGTCAACGACGGCCTCTACCACAGCTTCAACTGCATCCTCTTCGATCACGCCCACCCAACGCTTCTCCTGCTAAACGACGGCGACGGCGCTGACGGGGTGGAGAGTGGCACGGAGGCGGCAGCGGTGTGCAGCGAGGAGGAGGGCGAGACGTCGCTGAGCGGTCCCCTGGCGAACGATCCACTGTTCATGTCCGCCTGGGACCGCCGTCGCAGCTTCGCGCGCCGCCCGCTGCGCATCACAACCATCTTCGGCCCCACCTGCGATTCGATGGACTGCATCCTGAAGAAGCAGCCCTTCCCGGAGATGAAGCTGGGCGACTGGCTTCTGGTGCCAGACATGGGCAGCTACACCACCGCCGCCGCCGGCTTCTTCAACGGTTTCGCGACGCGCCGCCTTGAGTGGGTGAGCTCTGTGGACTTGTGCGCGAGGCCGAGGCCTGTGTATACGCGTGAGGGCAACACGCTGAGGTGTGTGAGCGAGTGAGAGAGGGACAGAAAGGAGACCCGGTGCTGTAGGGTGTGCTCGTCCATCCCACAAGAGGATGGACGGTCTCTTCGCTCCGGCCACCTCACCACCTCCTTTAGCCGCCCCCTCCTCCGCCCCCTCTCCCCCTATCGTCCCCTTTTCTGTGGTGGTTGCACTTGTATTCGTGTTGGATGCACGGCGACGCTGCTCGCTTGTGTTTGTGCGCCTGCGCCTGCGCGCGTGTTTCGCTTTTCGTTCACCCCCTGTGGTCCCTTCGTGAGCCTCCCCATCCCCGCTCTTCTTCGCCGCTGGCTTCGTTGTTGCTCTTCTCTGACGCCTACGCAACGCAAGCCCCCCCCCTCTCCCTCACCCTTGTGCCCCACAAACTCCCACTGACAGTGCTTCCACTTACTAAGTCGGCCCAACCCCAGCGAAGCCCCCTCGCCGCCGCTGCTGCCGCCGACCCCTTCTCTCTCTTGCCTCGTGTCTCTCCTGCTCCTTTTCTCTTGTCGTGTACA

>BPK282/0 clone 4|LinJ.12.0100|500 bp UPS + CDS + 500 bp DWS|ornithine decarboxylase

GCTCCTTCTTGTCGCGGCTGTGTGGGTTCGCTCATTCTCGGGGTGTTGTCCCCCTTCCCTCGTGGCATCGTCATCGTCTTCTCCACCGCCACCGTCCACGCTGCACACGCACACTCCACAGCGTACTTCACCTTCCTAGCTGTCTCCCTCACTCCCTCTCTGTAACCCTAACTGTCGTTGCGGCTACCGCTCTGCGTCCTTTCCGGCAACTTCTCCTGCGGTTCACTGCCAGCGTTGCTCCTACCTGTGGTGCCCCGTTCCTCTGGCCCCTCACACACACACACACTCACCTGCACTCTCTCAGTCTCCTTTGTCAACCGCTGGAGCTCAGCGCATTCGCTTTTTCGGCCGCCGCTTCGCCTCCATCTCGCGCCGCCGCACCTCCTCGCACCTCCCTCCTCCCTCGCCCCGCGCGCGCCGGCCTCTCTCTGCATTCCGCACAGACCTGCCAAGGCGCTCTCCCGTGTGTGTCGTCGCCCGCCGTCCTTCCCAAGTGCTCAATGGGTGATCATGACGTCGCTCTCTGCCACGTCAGCCGCTACAACCATGCGAATTACTGGGCCTTTGTGCCGCTGCCAACGGTCAGCGACGACACGGGATGCGACAGTCTGCATCATGACAGCGCTTCCAAAAGGATAAGGATGGCGCCGCCGGCGTCCGCGTCGAAGGCGGGCGCGGCCGAGGAACGCCTGCACCCCTACGAAAGGCGTCTATTGGATCAGTACCAGATCCACCTCCAACCCGCCAACCGCAACCCGCTGAGCCGAGCCGATTCCGCCGCCGGTCGAGAGGAAACGGCGCAGACCCCCGCGCAGGTGCAGATGGTGTCTGGTGTTGCTGTTGCGGACTCGACTTCAGATCAGCACGCCTCTGTTGCATCCAGTCAGGACCTCGTCGACCTCTTCTTCCTGAGGGCAGCCAGGCCGTCGACGGTCTGTGCTTCTCGCCCTACCCCATCTACGGTTGGCGCACGGCCGAGGAGCGCCGGGCTGCCGTTTGTGAGGTTTTCAAGACGTACAACGTGGTCACGCGCCTGCCCGCCTCGCCGGCTGCCCTGGCCGCCGCGCAGCGCCGCTACAGCCGCCACCGGCACTCCGCCATCGCCCCCATCAACAAGAGTGCGATCGAGACGCGTGAGCAGTACTGGCGCCGCCTGTCGAACCTGTACACCCAGAAGGGTGTAAAAGATGCTGCTTCTGCCGCCGACGCTGCGGCTACGACGGCGACGAACGGTGCCGTCCCCGCTGCCCCCGCCTACGAGCCCGAGGATCCATTCTACATCATCGATCTCGGCCGTGTTGTGGAGCAGATGGCGCGCTGGCGTCACGAGCTGCCGATGGTGCGCCCTTACTTTGCCGTGAAAAGCAACCCGCAACCGGCTGTTTTGGAGGTGCTCAGCGCCCTCGGCGCCGGTTTCGACTGCGCGTCCAAGGAAGAAATACATATGGTGCTAGGTCACCAGCTCGTGGCGTCGCCGGACGACATCATCTTCGCCAACCCGTGCAAGCAGCTCGGCGACCTGCGTGAGGCGCAGGCGTGCGGCGTGACCTACGTGACGGTGGACAACCCGCTGGAGATGGAAAAGATCAGTCGCCTGATGCCCTCCGCCCACGCGATTATCCGCATCAAGACGAACGACAGCAAGGCCCAGTGCTCCTTTTCCACCAAGTTCGGTGCCCCCCTTGAAGATGTCGAGGGCCTACTGGAGGCGGCCCGCCAGTTCAACGTCACCGTGTGCGGCGTCAGCTTTCACGTCGGCAGCGGCAACGACGACCAGTCTGCGTACGTGTCCGCGGTACGCGATGCCTACCAAGTCTTTCAGCAGGCGGTCCAGTACGGCTTCAAGTGCACCATCCTCGACATCGGCGGCGGCTTCCCGGGCACGGAGGTCGTCGAGGGCAGCGGCAATACCTCTTTCGAGGCCATTGCCCGTACTATTCGACCGGTGCTGGCGGAGCTCTTCGGCGGCGGTGATGTCACCATCATCAGTGAGCCCGGCCGCTACTTCACGGCTGCCTCGCACGCGCTTCTCATGAACGTATTCGCCTCCCGCACGCTGCGACTGTCCGATGTCGAGGTGAGTCGCCAAGCCTTCCAGTCCGTCGTGTCGATGGACGAGCCCGAAGAGTACCAGTACTACGTCAACGACGGCCTCTACCACAGCTTCAACTGCATCCTCTTCGATCACGCCCACCCAACGCTTCTCCTGCTAAACGACGGCGACGGCGCTGACGGGGTGGAGAGTGGCACGGAGGCGGCAGCGGTGTGCAGCGAGGAGGAGGGCGAGACGTCGCTGAGCGGTCCCCTGGCGAACGATCCACTGTTCATGTCCGCCTGGGACCGCCGTCGCAGCTTCGCGCGCCGCCCGCTGCGCATCACAACCATCTTCGGCCCCACCTGCGATTCGATGGACTGCATCCTGAAGAAGCAGCCCTTCCCGGAGATGAAGCTGGGCGACTGGCTTCTGGTGCCAGACATGGGCAGCTACACCACCGCCGCCGCCGGCTTCTTCAACGGTTTCGCGACGCGCCGCCTTGAGTGGGTGAGCTCTGTGGACTTGTGCGCGAGGCCGAGGCCTGTGTATACGCGTGAGGGCAACACGCTGAGGTGTGTGAGCGAGTGAGAGAGGGACAGAAAGGAGACCCGGTGCTGTAGGGTGTGCTCGTCCATCCCACAAGAGGATGGACGGTCTCTTCGCTCCGGCCACCTCACCACCTCCTTTAGCCGCCCCCTCCTCCGCCCCCTCTCCCCCTATCGTCCCCTTTTCTGTGGTGGTTGCACTTGTATTCGTGTTGGATGCACGGCGACGCTGCTCGCTTGTGTTTGTGCGCCTGCGCCTGCGCGCGTGTTTCGCTTTTCGTTCACCCCCTGTGGTCCCTTCGTGAGCCTCCCCATCCCCGCTCTTCTTCGCCGCTGGCTTCGTTGTTGCTCTTCTCTGACGCCTACGCAACGCAAGCCCCCCCCCTCTCCCTCACCCTTGTGCCCCACAAACTCCCACTGACAGTGCTTCCACTTACTAAGTCGGCCCAACCCCAGCGAAGCCCCCTCGCCGCCGCTGCTGCCGCCGACCCCTTCTCTCTCTTGCCTCGTGTCTCTCCTGCTCCTTTTCTCTTGTCGTGTACA

>BPK294/0 clone 1|LinJ.12.0100|500 bp UPS + CDS + 500 bp DWS|ornithine decarboxylase

GCTCCTTCTTGTCGCGGCTGTGTGGGTTCGCTCATTCTCGGGGTGTTGTCCCCCTTCCCTCGTGGCATCGTCATCGTCTTCTCCACCGCCACCGTCCACGCTGCACACGCACACTCCACAGCGTACTTCACCTTCCTAGCTGTCTCCCTCACTCCCTCTCTGTAACCCTAACTGTCGTTGCGGCTACCGCTCTGCGTCCTTTCCGGCAACTTCTCCTGCGGTTCACTGCCAGCGTTGCTCCTACCTGTGGTGCCCCGTTCCTCTGGCCCCTCACACACACACACACTCACCTGCACTCTCTCAGTCTCCTTTGTCAACCGCTGGAGCTCAGCGCATTCGCTTTTTCGGCCGCCGCTTCGCCTCCATCTCGCGCCGCCGCACCTCCTCGCACCTCCCTCCTCCCTCGCCCCGCGCGCGCCGGCCTCTCTCTGCATTCCGCACAGACCTGCCAAGGCGCTCTCCCGTGTGTGTCGTCGCCCGCCGTCCTTCCCAAGTGCTCAATGGGTGATCATGACGTCGCTCTCTGCCACGTCAGCCGCTACAACCATGCGAATTACTGGGCCTTTGTGCCGCTGCCAACGGTCAGCGACGACACGGGATGCGACAGTCTGCATCATGACAGCGCTTCCAAAAGGATAAGGATGGCGCCGCCGGCGTCCGCGTCGAAGGCGGGCGCGGCCGAGGAACGCCTGCACCCCTACGAAAGGCGTCTATTGGATCAGTACCAGATCCACCTCCAACCCGCCAACCGCAACCCGCTGAGCCGAGCCGATTCCGCCGCCGGTCGAGAGGAAACGGCGCAGACCCCCGCGCAGGTGCAGATGGTGTCTGGTGTTGCTGTTGCGGACTCGACTTCAGATCAGCACGCCTCTGTTGCATCCAGTCAGGACCTCGTCGACCTCTTCTTCCTGAGGGCAGCCAGGCCGTCGACGGTCTGTGCTTCTCGCCCTACCCCATCTACGGTTGGCGCACGGCCGAGGAGCGCCGGGCTGCCGTTTGTGAGGTTTTCAAGACGTACAACGTGGTCACGCGCCTGCCCGCCTCGCCGGCTGCCCTGGCCGCCGCGCAGCGCCGCTACAGCCGCCACCGGCACTCCGCCATCGCCCCCATCAACAAGAGTGCGATCGAGACGCGTGAGCAGTACTGGCGCCGCCTGTCGAACCTGTACACCCAGAAGGGTGTAAAAGATGCTGCTTCTGCCGCCGACGCTGCGGCTACGACGGCGACGAACGGTGCCGTCCCCGCTGCCCCCGCCTACGAGCCCGAGGATCCATTCTACATCATCGATCTCGGCCGTGTTGTGGAGCAGATGGCGCGCTGGCGTCACGAGCTGCCGATGGTGCGCCCTTACTTTGCCGTGAAAAGCAACCCGCAACCGGCTGTTTTGGAGGTGCTCAGCGCCCTCGGCGCCGGTTTCGACTGCGCGTCCAAGGAAGAAATACATATGGTGCTAGGTCACCAGCTCGTGGCGTCGCCGGACGACATCATCTTCGCCAACCCGTGCAAGCAGCTCGGCGACCTGCGTGAGGCGCAGGCGTGCGGCGTGACCTACGTGACGGTGGACAACCCGCTGGAGATGGAAAAGATCAGTCGCCTGATGCCCTCCGCCCACGCGATTATCCGCATCAAGACGAACGACAGCAAGGCCCAGTGCTCCTTTTCCACCAAGTTCGGTGCCCCCCTTGAAGATGTCGAGGGCCTACTGGAGGCGGCCCGCCAGTTCAACGTCACCGTGTGCGGCGTCAGCTTTCACGTCGGCAGCGGCAACGACGACCAGTCTGCGTACGTGTCCGCGGTACGCGATGCCTACCAAGTCTTTCAGCAGGCGGTCCAGTACGGCTTCAAGTGCACCATCCTCGACATCGGCGGCGGCTTCCCGGGCACGGAGGTCGTCGAGGGCAGCGGCAATACCTCTTTCGAGGCCATTGCCCGTACTATTCGACCGGTGCTGGCGGAGCTCTTCGGCGGCGGTGATGTCACCATCATCAGTGAGCCCGGCCGCTACTTCACGGCTGCCTCGCACGCGCTTCTCATGAACGTATTCGCCTCCCGCACGCTGCGACTGTCCGATGTCGAGGTGAGTCGCCAAGCCTTCCAGTCCGTCGTGTCGATGGACGAGCCCGAAGAGTACCAGTACTACGTCAACGACGGCCTCTACCACAGCTTCAACTGCATCCTCTTCGATCACGCCCACCCAACGCTTCTCCTGCTAAACGACGGCGACGGCGCTGACGGGGTGGAGAGTGGCACGGAGGCGGCAGCGGTGTGCAGCGAGGAGGAGGGCGAGACGTCGCTGAGCGGTCCCCTGGCGAACGATCCACTGTTCATGTCCGCCTGGGACCGCCGTCGCAGCTTCGCGCGCCGCCCGCTGCGCATCACAACCATCTTCGGCCCCACCTGCGATTCGATGGACTGCATCCTGAAGAAGCAGCCCTTCCCGGAGATGAAGCTGGGCGACTGGCTTCTGGTGCCAGACATGGGCAGCTACACCACCGCCGCCGCCGGCTTCTTCAACGGTTTCGCGACGCGCCGCCTTGAGTGGGTGAGCTCTGTGGACTTGTGCGCGAGGCCGAGGCCTGTGTATACGCGTGAGGGCAACACGCTGAGGTGTGTGAGCGAGTGAGAGAGGGACAGAAAGGAGACCCGGTGCTGTAGGGTGTGCTCGTCCATCCCACAAGAGGATGGACGGTCTCTTCGCTCCGGCCACCTCACCACCTCCTTTAGCCGCCCCCTCCTCCGCCCCCTCTCCCCCTATCGTCCCCTTTTCTGTGGTGGTTGCACTTGTATTCGTGTTGGATGCACGGCGACGCTGCTCGCTTGTGTTTGTGCGCCTGCGCCTGCGCGCGTGTTTCGCTTTTCGTTCACCCCCTGTGGTCCCTTCGTGAGCCTCCCCATCCCCGCTCTTCTTCGCCGCTGGCTTCGTTGTTGCTCTTCTCTGACGCCTACGCAACGCAAGCCCCCCCCCTCTCCCTCACCCTTGTGCCCCACAAACTCCCACTGACAGTGCTTCCACTTACTAAGTCGGCCCAACCCCAGCGAAGCCCCCTCGCCGCCGCTGCTGCCGCCGACCCCTTCTCTCTCTTGCCTCGTGTCTCTCCTGCTCCTTTTCTCTTGTCGTGTACA

>BPK298/0 clone 8|LinJ.12.0100|500 bp UPS + CDS + 500 bp DWS|ornithine decarboxylase

GCTCCTTCTTGTCGCGGCTGTGTGGGTTCGCTCATTCTCGGGGTGTTGTCCCCCTTCCCTCGTGGCATCGTCATCGTCTTCTCCACCGCCACCGTCCACGCTGCACACGCACACTCCACAGCGTACTTCACCTTCCTAGCTGTCTCCCTCACTCCCTCTCTGTAACCCTAACTGTCGTTGCGGCTACCGCTCTGCGTCCTTTCCGGCAACTTCTCCTGCGGTTCACTGCCAGCGTTGCTCCTACCTGTGGTGCCCCGTTCCTCTGGCCCCTCACACACACACACACTCACCTGCACTCTCTCAGTCTCCTTTGTCAACCGCTGGAGCTCAGCGCATTCGCTTTTTCGGCCGCCGCTTCGCCTCCATCTCGCGCCGCCGCACCTCCTCGCACCTCCCTCCTCCCTCGCCCCGCGCGCGCCGGCCTCTCTCTGCATTCCGCACAGACCTGCCAAGGCGCTCTCCCGTGTGTGTCGTCGCCCGCCGTCCTTCCCAAGTGCTCAATGGGTGATCATGACGTCGCTCTCTGCCACGTCAGCCGCTACAACCATGCGAATTACTGGGCCTTTGTGCCGCTGCCAACGGTCAGCGACGACACGGGATGCGACAGTCTGCATCATGACAGCGCTTCCAAAAGGATAAGGATGGCGCCGCCGGCGTCCGCGTCGAAGGCGGGCGCGGCCGAGGAACGCCTGCACCCCTACGAAAGGCGTCTATTGGATCAGTACCAGATCCACCTCCAACCCGCCAACCGCAACCCGCTGAGCCGAGCCGATTCCGCCGCCGGTCGAGAGGAAACGGCGCAGACCCCCGCGCAGGTGCAGATGGTGTCTGGTGTTGCTGTTGCGGACTCGACTTCAGATCAGCACGCCTCTGTTGCATCCAGTCAGGACCTCGTCGACCTCTTCTTCCTGAGGGCAGCCAGGCCGTCGACGGTCTGTGCTTCTCGCCCTACCCCATCTACGGTTGGCGCACGGCCGAGGAGCGCCGGGCTGCCGTTTGTGAGGTTTTCAAGACGTACAACGTGGTCACGCGCCTGCCCGCCTCGCCGGCTGCCCTGGCCGCCGCGCAGCGCCGCTACAGCCGCCACCGGCACTCCGCCATCGCCCCCATCAACAAGAGTGCGATCGAGACGCGTGAGCAGTACTGGCGCCGCCTGTCGAACCTGTACACCCAGAAGGGTGTAAAAGATGCTGCTTCTGCCGCCGACGCTGCGGCTACGACGGCGACGAACGGTGCCGTCCCCGCTGCCCCCGCCTACGAGCCCGAGGATCCATTCTACATCATCGATCTCGGCCGTGTTGTGGAGCAGATGGCGCGCTGGCGTCACGAGCTGCCGATGGTGCGCCCTTACTTTGCCGTGAAAAGCAACCCGCAACCGGCTGTTTTGGAGGTGCTCAGCGCCCTCGGCGCCGGTTTCGACTGCGCGTCCAAGGAAGAAATACATATGGTGCTAGGTCACCAGCTCGTGGCGTCGCCGGACGACATCATCTTCGCCAACCCGTGCAAGCAGCTCGGCGACCTGCGTGAGGCGCAGGCGTGCGGCGTGACCTACGTGACGGTGGACAACCCGCTGGAGATGGAAAAGATCAGTCGCCTGATGCCCTCCGCCCACGCGATTATCCGCATCAAGACGAACGACAGCAAGGCCCAGTGCTCCTTTTCCACCAAGTTCGGTGCCCCCCTTGAAGATGTCGAGGGCCTACTGGAGGCGGCCCGCCAGTTCAACGTCACCGTGTGCGGCGTCAGCTTTCACGTCGGCAGCGGCAACGACGACCAGTCTGCGTACGTGTCCGCGGTACGCGATGCCTACCAAGTCTTTCAGCAGGCGGTCCAGTACGGCTTCAAGTGCACCATCCTCGACATCGGCGGCGGCTTCCCGGGCACGGAGGTCGTCGAGGGCAGCGGCAATACCTCTTTCGAGGCCATTGCCCGTACTATTCGACCGGTGCTGGCGGAGCTCTTCGGCGGCGGTGATGTCACCATCATCAGTGAGCCCGGCCGCTACTTCACGGCTGCCTCGCACGCGCTTCTCATGAACGTATTCGCCTCCCGCACGCTGCGACTGTCCGATGTCGAGGTGAGTCGCCAAGCCTTCCAGTCCGTCGTGTCGATGGACGAGCCCGAAGAGTACCAGTACTACGTCAACGACGGCCTCTACCACAGCTTCAACTGCATCCTCTTCGATCACGCCCACCCAACGCTTCTCCTGCTAAACGACGGCGACGGCGCTGACGGGGTGGAGAGTGGCACGGAGGCGGCAGCGGTGTGCAGCGAGGAGGAGGGCGAGACGTCGCTGAGCGGTCCCCTGGCGAACGATCCACTGTTCATGTCCGCCTGGGACCGCCGTCGCAGCTTCGCGCGCCGCCCGCTGCGCATCACAACCATCTTCGGCCCCACCTGCGATTCGATGGACTGCATCCTGAAGAAGCAGCCCTTCCCGGAGATGAAGCTGGGCGACTGGCTTCTGGTGCCAGACATGGGCAGCTACACCACCGCCGCCGCCGGCTTCTTCAACGGTTTCGCGACGCGCCGCCTTGAGTGGGTGAGCTCTGTGGACTTGTGCGCGAGGCCGAGGCCTGTGTATACGCGTGAGGGCAACACGCTGAGGTGTGTGAGCGAGTGAGAGAGGGACAGAAAGGAGACCCGGTGCTGTAGGGTGTGCTCGTCCATCCCACAAGAGGATGGACGGTCTCTTCGCTCCGGCCACCTCACCACCTCCTTTAGCCGCCCCCTCCTCCGCCCCCTCTCCCCCTATCGTCCCCTTTTCTGTGGTGGTTGCACTTGTATTCGTGTTGGATGCACGGCGACGCTGCTCGCTTGTGTTTGTGCGCCTGCGCCTGCGCGCGTGTTTCGCTTTTCGTTCACCCCCTGTGGTCCCTTCGTGAGCCTCCCCATCCCCGCTCTTCTTCGCCGCTGGCTTCGTTGTTGCTCTTCTCTGACGCCTACGCAACGCAAGCCCCCCCCCTCTCCCTCACCCTTGTGCCCCACAAACTCCCACTGACAGTGCTTCCACTTACTAAGTCGGCCCAACCCCAGCGAAGCCCCCTCGCCGCCGCTGCTGCCGCCGACCCCTTCTCTCTCTTGCCTCGTGTCTCTCCTGCTCCTTTTCTCTTGTCGTGTACA

### 9. THIOL DEPENDENT REDUCTASE 1

>BPK035/0 clone 1|LinJ33_V3.0260|500 bp UPS + CDS + 500 bp DWS|thiol dependent reductase 1

AAGGCTCGTCGAGGGGATCGACGTGCCGACCATGCGAGGCTATTTGTTTTTGAGCTGCGCATCTGATCATGCATGTCTCTGTGCCGATCTCTGTGATTATCTCTGTGAGCGGTGTGCTCTACCAAAGCGGATTTCGGCTGCGAGGCTGCCATCGGCTCCAAAATCGCACGTTAGTCGTAGTTTGTCTTCCTAAAACTGAAGCGCATGCACACCAACGCGAGTGAGGTTGGGCGGAGATGGGGAAGGTACCACCTGAGGGCGAGTAGCGGAGAAGCGTGTCGTCTTGGGCGCCGTTCTGTGTCTCGTAGCGACTTGTATGCTCATGTGATGCGAGGTTTTGTTTTCTTTTTTGAACTGTTATCGTGTTCACTGCCGGTTCGCGGGCCTCATGTTCGGCAACGTTCTAGTTTTTTTCGCTGCTGTGGGACGTGGGCGACTGCGGCAAAGCATGTCGTCGTGCTGAGCTTTTTTCGCGTATGCTCAACTTTCCGCGAGTGAGTATGGCCGCCCGCGCGCTAAAGCTGTACGTGTCGGCAACGTGCCCGTTCTGCCACCGCGTGGAGATCGTCGCACGGGAGAAGCAGGTCTCATACGATCGCGTTGCTGTTGGGCTGCGCGAGGAGATGCCGCAATGGTACAAGCAGATTAACCCGCGTGAGACAGTGCCGACGCTGGAAGTCGGCAATGCGGATAAGCGGTTTATGTTCGAGTCGATGCTGATCGCGCAGTACCTGGACAACAGTGGCGCGCCCGCGGGCGCGCTGATGGGTTCCTCGGCGGCGCAACGGCACCAGATTGAGTTCTTCCTCGCACAGGTCGGCGATTTCATTGGCGCTGCGCACGGGCTGCTCCGCGATCCGCTGAGTGGTGAAAAGCGCAAGGCCATGGATGACAACGCGGCGTACGTGGACGGGCTGCTCGCGGCGAACCAGACGACGGGGCCATACTACTGCGACGGCGAGTTCACGATGGCGGACGTTGCGCTTGTGCCGTTCCTGGTGCGGCTGAAACCTGCTCTGATGTACTACGCCGGGTACGACGTGTTCTGCAAGGCGCCACGGATGAAGGCGCTGTGGGCCGCTGCTGCGCAACGCGCGTCTGTGCGTGAGACTTCGCCGACGGCGGCGCAGTGCATCGAGAACTACCGCCACCTGGTGCCGGAGAGCGCGCCAATGATGGGCGCCAATGGTGGGCACGTCCTGTACAGCAATCTTTTCTGTCCTTTTGTGGACCGCGCACGCCTCGCGTCTGAGCTGCGCAAGTTCCAGATGCACATAGTGGAGGTGCCGCTGCATCCACAGCCGGAGTGGTACAAATATATCAATCCCCGCGATACGGTGCCTGCGCTGTTTACACCGAGCGGCGAGGCTGTACACGAGTCGCAGCTGATTGTTCAGTACATCGACTGCGTGGCGACGAAGGGTAGTGCGCTGGTGCCGCGTGGGGACGCGGAGAAGGAGTACGAGGTGGGTTTCTTCGTGGAAAACGCTGGGTATTTCGTTGGAGGACTGATGTCGTGGATCATCCGTGGTGGCGAGGATGCGAAGGCTGAGCTTCAGTGGGCTGCTGGCGAGCTCGAGCAGCAGCTGGCGAAGCACCCGTTTGGTGAGGGCCCCTTCTTCGGCGGCAAGCGGATGAACGCTGGCGATGTGGCTATTCTACCTTTCCTGGTCCGCGCAAAGGCGTTTATGCCGGAGTTTAGTGGTGGGTACGATCTCTTTGCCCACTTTCCGCTGCTGAATGGGCTGGCAGAGGCTGGCATGGCGACACCGGAGGCGAAGTCGGTGTTTCGTACACTGGAGGAGTACAAGGAGCACATCCGTAAGCGCCAACGGAGGGCCCAGAGCGGGTAAGCGGCGCGTGTAGCATACCTATTCCACTCCCTGCTTTATGGACTTGCAGCGCAGTGATCCCATCGGCTCTCTGGGTGCTGCATTGCGTCCCATTTCGCCCATCTGCGCCACCCCGTTCGCGCGCGGCGCGCCTGTGCCTCCGGCGGTGCGAGGCGAAGTGAGCCCACGCGTACGCGTCTCCAATCGTCTCATCTACTCCCCACCCCGCATCTCCCCCCCCCTCCATCATCTCCCGCCTTTTTCTCGCTCGCTTGGTGCTGTCCACGTGAGTGGATGTGTTTGCTGCCTGTCAGCCGAGCACCACAGCAAGCAGGTCCCCTGCAGCCCTTTCGCTTCCGCTCCCCAGCCCATCTTTTTTCTCTGTTTGTTTTTCTGTTCTCCCCTACGGTCTCTCACCATCTCTCCCGTTAGAAGTGCGCCCGCTGCGTGTTGCTGCTGACGCTCCCCACTCATCTTGCACCGTCGATCCACAACTAAGCCACTGCGTGCCTGGCGTCGAG

>BPK043/0 clone 2|LinJ33_V3.0260|500 bp UPS + CDS + 500 bp DWS|thiol dependent reductase 1

AAGGCTCGTCGAGGGGATCGACGTGCCGACCATGCGAGGCTATTTGTTTTTGAGCTGCGCATCTGATCATGCATGTCTCTGTGCCGATCTCTGTGATTATCTCTGTGAGCGGTGTGCTCTACCAAAGCGGATTTCGGCTGCGAGGCTGCCATCGGCTCCAAAATCGCACGTTAGTCGTAGTTTGTCTTCCTAAAACTGAAGCGCATGCACACCAACGCGAGTGAGGTTGGGCGGAGATGGGGAAGGTACCACCTGAGGGCGAGTAGCGGAGAAGCGTGTCGTCTTGGGCGCCGTTCTGTGTCTCGTAGCGACTTGTATGCTCATGTGATGCGAGGTTTTGTTTTCTTTTTTGAACTGTTATCGTGTTCACTGCCGGTTCGCGGGCCTCATGTTCGGCAACGTTCTAGTTTTTTTCGCTGCTGTGGGACGTGGGCGACTGCGGCAAAGCATGTCGTCGTGCTGAGCTTTTTTCGCGTATGCTCAACTTTCCGCGAGTGAGTATGGCCGCCCGCGCGCTAAAGCTGTACGTGTCGGCAACGTGCCCGTTCTGCCACCGCGTGGAGATCGTCGCACGGGAGAAGCAGGTCTCATACGATCGCGTTGCTGTTGGGCTGCGCGAGGAGATGCCGCAATGGTACAAGCAGATTAACCCGCGTGAGACAGTGCCGACGCTGGAAGTCGGCAATGCGGATAAGCGGTTTATGTTCGAGTCGATGCTGATCGCGCAGTACCTGGACAACAGTGGCGCGCCCGCGGGCGCGCTGATGGGTTCCTCGGCGGCGCAACGGCACCAGATTGAGTTCTTCCTCGCACAGGTCGGCGATTTCATTGGCGCTGCGCACGGGCTGCTCCGCGATCCGCTGAGTGGTGAAAAGCGCAAGGCCATGGATGACAACGCGGCGTACGTGGACGGGCTGCTCGCGGCGAACCAGACGACGGGGCCATACTACTGCGACGGCGAGTTCACGATGGCGGACGTTGCGCTTGTGCCGTTCCTGGTGCGGCTGAAACCTGCTCTGATGTACTACGCCGGGTACGACGTGTTCTGCAAGGCGCCACGGATGAAGGCGCTGTGGGCCGCTGCTGCGCAACGCGCGTCTGTGCGTGAGACTTCGCCGACGGCGGCGCAGTGCATCGAGAACTACCGCCACCTGGTGCCGGAGAGCGCGCCAATGATGGGCGCCAATGGTGGGCACGTCCTGTACAGCAATCTTTTCTGTCCTTTTGTGGACCGCGCACGCCTCGCGTCTGAGCTGCGCAAGTTCCAGATGCACATAGTGGAGGTGCCGCTGCATCCACAGCCGGAGTGGTACAAATATATCAATCCCCGCGATACGGTGCCTGCGCTGTTTACACCGAGCGGCGAGGCTGTACACGAGTCGCAGCTGATTGTTCAGTACATCGACTGCGTGGCGACGAAGGGTAGTGCGCTGGTGCCGCGTGGGGACGCGGAGAAGGAGTACGAGGTGGGTTTCTTCGTGGAAAACGCTGGGTATTTCGTTGGAGGACTGATGTCGTGGATCATCCGTGGTGGCGAGGATGCGAAGGCTGAGCTTCAGTGGGCTGCTGGCGAGCTCGAGCAGCAGCTGGCGAAGCACCCGTTTGGTGAGGGCCCCTTCTTCGGCGGCAAGCGGATGAACGCTGGCGATGTGGCTATTCTACCTTTCCTGGTCCGCGCAAAGGCGTTTATGCCGGAGTTTAGTGGTGGGTACGATCTCTTTGCCCACTTTCCGCTGCTGAATGGGCTGGCAGAGGCTGGCATGGCGACACCGGAGGCGAAGTCGGTGTTTCGTACACTGGAGGAGTACAAGGAGCACATCCGTAAGCGCCAACGGAGGGCCCAGAGCGGGTAAGCGGCGCGTGTAGCATACCTATTCCACTCCCTGCTTTATGGACTTGCAGCGCAGTGATCCCATCGGCTCTCTGGGTGCTGCATTGCGTCCCATTTCGCCCATCTGCGCCACCCCGTTCGCGCGCGGCGCGCCTGTGCCTCCGGCGGTGCGAGGCGAAGTGAGCCCACGCGTACGCGTCTCCAATCGTCTCATCTACTCCCCACCCCGCATCTCCCCCCCCCTCCATCATCTCCCGCCTTTTTCTCGCTCGCTTGGTGCTGTCCACGTGAGTGGATGTGTTTGCTGCCTGTCAGCCGAGCACCACAGCAAGCAGGTCCCCTGCAGCCCTTTCGCTTCCGCTCCCCAGCCCATCTTTTTTCTCTGTTTGTTTTTCTGTTCTCCCCTACGGTCTCTCACCATCTCTCCCGTTAGAAGTGCGCCCGCTGCGTGTTGCTGCTGACGCTCCCCACTCATCTTGCACCGTCGATCCACAACTAAGCCACTGCGTGCCTGGCGTCGAG

>BPK085/0 clone 8|LinJ33_V3.0260|500 bp UPS + CDS + 500 bp DWS|thiol dependent reductase 1

AAGGCTCGTCGAGGGGATCGACGTGCCGACCATGCGAGGCTATTTGTTTTTGAGCTGCGCATCTGATCATGCATGTCTCTGTGCCGATCTCTGTGATTATCTCTGTGAGCGGTGTGCTCTACCAAAGCGGATTTCGGCTGCGAGGCTGCCATCGGCTCCAAAATCGCACGTTAGTCGTAGTTTGTCTTCCTAAAACTGAAGCGCATGCACACCAACGCGAGTGAGGTTGGGCGGAGATGGGGAAGGTACCACCTGAGGGCGAGTAGCGGAGAAGCGTGTCGTCTTGGGCGCCGTTCTGTGTCTCGTAGCGACTTGTATGCTCATGTGATGCGAGGTTTTGTTTTCTTTTTTGAACTGTTATCGTGTTCACTGCCGGTTCGCGGGCCTCATGTTCGGCAACGTTCTAGTTTTTTTCGCTGCTGTGGGACGTGGGCGACTGCGGCAAAGCATGTCGTCGTGCTGAGCTTTTTTCGCGTATGCTCAACTTTCCGCGAGTGAGTATGGCCGCCCGCGCGCTAAAGCTGTACGTGTCGGCAACGTGCCCGTTCTGCCACCGCGTGGAGATCGTCGCACGGGAGAAGCAGGTCTCATACGATCGCGTTGCTGTTGGGCTGCGCGAGGAGATGCCGCAATGGTACAAGCAGATTAACCCGCGTGAGACAGTGCCGACGCTGGAAGTCGGCAATGCGGATAAGCGGTTTATGTTCGAGTCGATGCTGATCGCGCAGTACCTGGACAACAGTGGCGCGCCCGCGGGCGCGCTGATGGGTTCCTCGGCGGCGCAACGGCACCAGATTGAGTTCTTCCTCGCACAGGTCGGCGATTTCATTGGCGCTGCGCACGGGCTGCTCCGCGATCCGCTGAGTGGTGAAAAGCGCAAGGCCATGGATGACAACGCGGCGTACGTGGACGGGCTGCTCGCGGCGAACCAGACGACGGGGCCATACTACTGCGACGGCGAGTTCACGATGGCGGACGTTGCGCTTGTGCCGTTCCTGGTGCGGCTGAAACCTGCTCTGATGTACTACGCCGGGTACGACGTGTTCTGCAAGGCGCCACGGATGAAGGCGCTGTGGGCCGCTGCTGCGCAACGCGCGTCTGTGCGTGAGACTTCGCCGACGGCGGCGCAGTGCATCGAGAACTACCGCCACCTGGTGCCGGAGAGCGCGCCAATGATGGGCGCCAATGGTGGGCACGTCCTGTACAGCAATCTTTTCTGTCCTTTTGTGGACCGCGCACGCCTCGCGTCTGAGCTGCGCAAGTTCCAGATGCACATAGTGGAGGTGCCGCTGCATCCACAGCCGGAGTGGTACAAATATATCAATCCCCGCGATACGGTGCCTGCGCTGTTTACACCGAGCGGCGAGGCTGTACACGAGTCGCAGCTGATTGTTCAGTACATCGACTGCGTGGCGACGAAGGGTAGTGCGCTGGTGCCGCGTGGGGACGCGGAGAAGGAGTACGAGGTGGGTTTCTTCGTGGAAAACGCTGGGTATTTCGTTGGAGGACTGATGTCGTGGATCATCCGTGGTGGCGAGGATGCGAAGGCTGAGCTTCAGTGGGCTGCTGGCGAGCTCGAGCAGCAGCTGGCGAAGCACCCGTTTGGTGAGGGCCCCTTCTTCGGCGGCAAGCGGATGAACGCTGGCGATGTGGCTATTCTACCTTTCCTGGTCCGCGCAAAGGCGTTTATGCCGGAGTTTAGTGGTGGGTACGATCTCTTTGCCCACTTTCCGCTGCTGAATGGGCTGGCAGAGGCTGGCATGGCGACACCGGAGGCGAAGTCGGTGTTTCGTACACTGGAGGAGTACAAGGAGCACATCCGTAAGCGCCAACGGAGGGCCCAGAGCGGGTAAGCGGCGCGTGTAGCATACCTATTCCACTCCCTGCTTTATGGACTTGCAGCGCAGTGATCCCATCGGCTCTCTGGGTGCTGCATTGCGTCCCATTTCGCCCATCTGCGCCACCCCGTTCGCGCGCGGCGCGCCTGTGCCTCCGGCGGTGCGAGGCGAAGTGAGCCCACGCGTACGCGTCTCCAATCGTCTCATCTACTCCCCACCCCGCATCTCCCCCCCCCTCCATCATCTCCCGCCTTTTTCTCGCTCGCTTGGTGCTGTCCACGTGAGTGGATGTGTTTGCTGCCTGTCAGCCGAGCACCACAGCAAGCAGGTCCCCTGCAGCCCTTTCGCTTCCGCTCCCCAGCCCATCTTTTTTCTCTGTTTGTTTTTCTGTTCTCCCCTACGGTCTCTCACCATCTCTCCCGTTAGAAGTGCGCCCGCTGCGTGTTGCTGCTGACGCTCCCCACTCATCTTGCACCGTCGATCCACAACTAAGCCACTGCGTGCCTGGCGTCGAG

>BPK087/0 clone 11|LinJ33_V3.0260|500 bp UPS + CDS + 500 bp DWS|thiol dependent reductase 1

AAGGCTCGTCGAGGGGATCGACGTGCCGACCATGCGAGGCTATTTGTTTTTGAGCTGCGCATCTGATCATGCATGTCTCTGTGCCGATCTCTGTGATTATCTCTGTGAGCGGTGTGCTCTACCAAAGCGGATTTCGGCTGCGAGGCTGCCATCGGCTCCAAAATCGCACGTTAGTCGTAGTTTGTCTTCCTAAAACTGAAGCGCATGCACACCAACGCGAGTGAGGTTGGGCGGAGATGGGGAAGGTACCACCTGAGGGCGAGTAGCGGAGAAGCGTGTCGTCTTGGGCGCCGTTCTGTGTCTCGTAGCGACTTGTATGCTCATGTGATGCGAGGTTTTGTTTTCTTTTTTGAACTGTTATCGTGTTCACTGCCGGTTCGCGGGCCTCATGTTCGGCAACGTTCTAGTTTTTTTCGCTGCTGTGGGACGTGGGCGACTGCGGCAAAGCATGTCGTCGTGCTGAGCTTTTTTCGCGTATGCTCAACTTTCCGCGAGTGAGTATGGCCGCCCGCGCGCTAAAGCTGTACGTGTCGGCAACGTGCCCGTTCTGCCACCGCGTGGAGATCGTCGCACGGGAGAAGCAGGTCTCATACGATCGCGTTGCTGTTGGGCTGCGCGAGGAGATGCCGCAATGGTACAAGCAGATTAACCCGCGTGAGACAGTGCCGACGCTGGAAGTCGGCAATGCGGATAAGCGGTTTATGTTCGAGTCGATGCTGATCGCGCAGTACCTGGACAACAGTGGCGCGCCCGCGGGCGCGCTGATGGGTTCCTCGGCGGCGCAACGGCACCAGATTGAGTTCTTCCTCGCACAGGTCGGCGATTTCATTGGCGCTGCGCACGGGCTGCTCCGCGATCCGCTGAGTGGTGAAAAGCGCAAGGCCATGGATGACAACGCGGCGTACGTGGACGGGCTGCTCGCGGCGAACCAGACGACGGGGCCATACTACTGCGACGGCGAGTTCACGATGGCGGACGTTGCGCTTGTGCCGTTCCTGGTGCGGCTGAAACCTGCTCTGATGTACTACGCCGGGTACGACGTGTTCTGCAAGGCGCCACGGATGAAGGCGCTGTGGGCCGCTGCTGCGCAACGCGCGTCTGTGCGTGAGACTTCGCCGACGGCGGCGCAGTGCATCGAGAACTACCGCCACCTGGTGCCGGAGAGCGCGCCAATGATGGGCGCCAATGGTGGGCACGTCCTGTACAGCAATCTTTTCTGTCCTTTTGTGGACCGCGCACGCCTCGCGTCTGAGCTGCGCAAGTTCCAGATGCACATAGTGGAGGTGCCGCTGCATCCACAGCCGGAGTGGTACAAATATATCAATCCCCGCGATACGGTGCCTGCGCTGTTTACACCGAGCGGCGAGGCTGTACACGAGTCGCAGCTGATTGTTCAGTACATCGACTGCGTGGCGACGAAGGGTAGTGCGCTGGTGCCGCGTGGGGACGCGGAGAAGGAGTACGAGGTGGGTTTCTTCGTGGAAAACGCTGGGTATTTCGTTGGAGGACTGATGTCGTGGATCATCCGTGGTGGCGAGGATGCGAAGGCTGAGCTTCAGTGGGCTGCTGGCGAGCTCGAGCAGCAGCTGGCGAAGCACCCGTTTGGTGAGGGCCCCTTCTTCGGCGGCAAGCGGATGAACGCTGGCGATGTGGCTATTCTACCTTTCCTGGTCCGCGCAAAGGCGTTTATGCCGGAGTTTAGTGGTGGGTACGATCTCTTTGCCCACTTTCCGCTGCTGAATGGGCTGGCAGAGGCTGGCATGGCGACACCGGAGGCGAAGTCGGTGTTTCGTACACTGGAGGAGTACAAGGAGCACATCCGTAAGCGCCAACGGAGGGCCCAGAGCGGGTAAGCGGCGCGTGTAGCATACCTATTCCACTCCCTGCTTTATGGACTTGCAGCGCAGTGATCCCATCGGCTCTCTGGGTGCTGCATTGCGTCCCATTTCGCCCATCTGCGCCACCCCGTTCGCGCGCGGCGCGCCTGTGCCTCCGGCGGTGCGAGGCGAAGTGAGCCCACGCGTACGCGTCTCCAATCGTCTCATCTACTCCCCACCCCGCATCTCCCCCCCCCTCCATCATCTCCCGCCTTTTTCTCGCTCGCTTGGTGCTGTCCACGTGAGTGGATGTGTTTGCTGCCTGTCAGCCGAGCACCACAGCAAGCAGGTCCCCTGCAGCCCTTTCGCTTCCGCTCCCCAGCCCATCTTTTTTCTCTGTTTGTTTTTCTGTTCTCCCCTACGGTCTCTCACCATCTCTCCCGTTAGAAGTGCGCCCGCTGCGTGTTGCTGCTGACGCTCCCCACTCATCTTGCACCGTCGATCCACAACTAAGCCACTGCGTGCCTGGCGTCGAG

>BPK178/0 clone 3|LinJ33_V3.0260|500 bp UPS + CDS + 500 bp DWS|thiol dependent reductase 1

AAGGCTCGTCGAGGGGATCGACGTGCCGACCATGCGAGGCTATTTGTTTTTGAGCTGCGCATCTGATCATGCATGTCTCTGTGCCGATCTCTGTGATTATCTCTGTGAGCGGTGTGCTCTACCAAAGCGGATTTCGGCTGCGAGGCTGCCATCGGCTCCAAAATCGCACGTTAGTCGTAGTTTGTCTTCCTAAAACTGAAGCGCATGCACACCAACGCGAGTGAGGTTGGGCGGAGATGGGGAAGGTACCACCTGAGGGCGAGTAGCGGAGAAGCGTGTCGTCTTGGGCGCCGTTCTGTGTCTCGTAGCGACTTGTATGCTCATGTGATGCGAGGTTTTGTTTTCTTTTTTGAACTGTTATCGTGTTCACTGCCGGTTCGCGGGCCTCATGTTCGGCAACGTTCTAGTTTTTTTCGCTGCTGTGGGACGTGGGCGACTGCGGCAAAGCATGTCGTCGTGCTGAGCTTTTTTCGCGTATGCTCAACTTTCCGCGAGTGAGTATGGCCGCCCGCGCGCTAAAGCTGTACGTGTCGGCAACGTGCCCGTTCTGCCACCGCGTGGAGATCGTCGCACGGGAGAAGCAGGTCTCATACGATCGCGTTGCTGTTGGGCTGCGCGAGGAGATGCCGCAATGGTACAAGCAGATTAACCCGCGTGAGACAGTGCCGACGCTGGAAGTCGGCAATGCGGATAAGCGGTTTATGTTCGAGTCGATGCTGATCGCGCAGTACCTGGACAACAGTGGCGCGCCCGCGGGCGCGCTGATGGGTTCCTCGGCGGCGCAACGGCACCAGATTGAGTTCTTCCTCGCACAGGTCGGCGATTTCATTGGCGCTGCGCACGGGCTGCTCCGCGATCCGCTGAGTGGTGAAAAGCGCAAGGCCATGGATGACAACGCGGCGTACGTGGACGGGCTGCTCGCGGCGAACCAGACGACGGGGCCATACTACTGCGACGGCGAGTTCACGATGGCGGACGTTGCGCTTGTGCCGTTCCTGGTGCGGCTGAAACCTGCTCTGATGTACTACGCCGGGTACGACGTGTTCTGCAAGGCGCCACGGATGAAGGCGCTGTGGGCCGCTGCTGCGCAACGCGCGTCTGTGCGTGAGACTTCGCCGACGGCGGCGCAGTGCATCGAGAACTACCGCCACCTGGTGCCGGAGAGCGCGCCAATGATGGGCGCCAATGGTGGGCACGTCCTGTACAGCAATCTTTTCTGTCCTTTTGTGGACCGCGCACGCCTCGCGTCTGAGCTGCGCAAGTTCCAGATGCACATAGTGGAGGTGCCGCTGCATCCACAGCCGGAGTGGTACAAATATATCAATCCCCGCGATACGGTGCCTGCGCTGTTTACACCGAGCGGCGAGGCTGTACACGAGTCGCAGCTGATTGTTCAGTACATCGACTGCGTGGCGACGAAGGGTAGTGCGCTGGTGCCGCGTGGGGACGCGGAGAAGGAGTACGAGGTGGGTTTCTTCGTGGAAAACGCTGGGTATTTCGTTGGAGGACTGATGTCGTGGATCATCCGTGGTGGCGAGGATGCGAAGGCTGAGCTTCAGTGGGCTGCTGGCGAGCTCGAGCAGCAGCTGGCGAAGCACCCGTTTGGTGAGGGCCCCTTCTTCGGCGGCAAGCGGATGAACGCTGGCGATGTGGCTATTCTACCTTTCCTGGTCCGCGCAAAGGCGTTTATGCCGGAGTTTAGTGGTGGGTACGATCTCTTTGCCCACTTTCCGCTGCTGAATGGGCTGGCAGAGGCTGGCATGGCGACACCGGAGGCGAAGTCGGTGTTTCGTACACTGGAGGAGTACAAGGAGCACATCCGTAAGCGCCAACGGAGGGCCCAGAGCGGGTAAGCGGCGCGTGTAGCATACCTATTCCACTCCCTGCTTTATGGACTTGCAGCGCAGTGATCCCATCGGCTCTCTGGGTGCTGCATTGCGTCCCATTTCGCCCATCTGCGCCACCCCGTTCGCGCGCGGCGCGCCTGTGCCTCCGGCGGTGCGAGGCGAAGTGAGCCCACGCGTACGCGTCTCCAATCGTCTCATCTACTCCCCACCCCGCATCTCCCCCCCCCTCCATCATCTCCCGCCTTTTTCTCGCTCGCTTGGTGCTGTCCACGTGAGTGGATGTGTTTGCTGCCTGTCAGCCGAGCACCACAGCAAGCAGGTCCCCTGCAGCCCTTTCGCTTCCGCTCCCCAGCCCATCTTTTTTCTCTGTTTGTTTTTCTGTTCTCCCCTACGGTCTCTCACCATCTCTCCCGTTAGAAGTGCGCCCGCTGCGTGTTGCTGCTGACGCTCCCCACTCATCTTGCACCGTCGATCCACAACTAAGCCACTGCGTGCCTGGCGTCGAG

>BPK190/0 clone 3|LinJ33_V3.0260|500 bp UPS + CDS + 500 bp DWS|thiol dependent reductase 1

AAGGCTCGTCGAGGGGATCGACGTGCCGACCATGCGAGGCTATTTGTTTTTGAGCTGCGCATCTGATCATGCATGTCTCTGTGCCGATCTCTGTGATTATCTCTGTGAGCGGTGTGCTCTACCAAAGCGGATTTCGGCTGCGAGGCTGCCATCGGCTCCAAAATCGCACGTTAGTCGTAGTTTGTCTTCCTAAAACTGAAGCGCATGCACACCAACGCGAGTGAGGTTGGGCGGAGATGGGGAAGGTACCACCTGAGGGCGAGTAGCGGAGAAGCGTGTCGTCTTGGGCGCCGTTCTGTGTCTCGTAGCGACTTGTATGCTCATGTGATGCGAGGTTTTGTTTTCTTTTTTGAACTGTTATCGTGTTCACTGCCGGTTCGCGGGCCTCATGTTCGGCAACGTTCTAGTTTTTTTCGCTGCTGTGGGACGTGGGCGACTGCGGCAAAGCATGTCGTCGTGCTGAGCTTTTTTCGCGTATGCTCAACTTTCCGCGAGTGAGTATGGCCGCCCGCGCGCTAAAGCTGTACGTGTCGGCAACGTGCCCGTTCTGCCACCGCGTGGAGATCGTCGCACGGGAGAAGCAGGTCTCATACGATCGCGTTGCTGTTGGGCTGCGCGAGGAGATGCCGCAATGGTACAAGCAGATTAACCCGCGTGAGACAGTGCCGACGCTGGAAGTCGGCAATGCGGATAAGCGGTTTATGTTCGAGTCGATGCTGATCGCGCAGTACCTGGACAACAGTGGCGCGCCCGCGGGCGCGCTGATGGGTTCCTCGGCGGCGCAACGGCACCAGATTGAGTTCTTCCTCGCACAGGTCGGCGATTTCATTGGCGCTGCGCACGGGCTGCTCCGCGATCCGCTGAGTGGTGAAAAGCGCAAGGCCATGGATGACAACGCGGCGTACGTGGACGGGCTGCTCGCGGCGAACCAGACGACGGGGCCATACTACTGCGACGGCGAGTTCACGATGGCGGACGTTGCGCTTGTGCCGTTCCTGGTGCGGCTGAAACCTGCTCTGATGTACTACGCCGGGTACGACGTGTTCTGCAAGGCGCCACGGATGAAGGCGCTGTGGGCCGCTGCTGCGCAACGCGCGTCTGTGCGTGAGACTTCGCCGACGGCGGCGCAGTGCATCGAGAACTACCGCCACCTGGTGCCGGAGAGCGCGCCAATGATGGGCGCCAATGGTGGGCACGTCCTGTACAGCAATCTTTTCTGTCCTTTTGTGGACCGCGCACGCCTCGCGTCTGAGCTGCGCAAGTTCCAGATGCACATAGTGGAGGTGCCGCTGCATCCACAGCCGGAGTGGTACAAATATATCAATCCCCGCGATACGGTGCCTGCGCTGTTTACACCGAGCGGCGAGGCTGTACACGAGTCGCAGCTGATTGTTCAGTACATCGACTGCGTGGCGACGAAGGGTAGTGCGCTGGTGCCGCGTGGGGACGCGGAGAAGGAGTACGAGGTGGGTTTCTTCGTGGAAAACGCTGGGTATTTCGTTGGAGGACTGATGTCGTGGATCATCCGTGGTGGCGAGGATGCGAAGGCTGAGCTTCAGTGGGCTGCTGGCGAGCTCGAGCAGCAGCTGGCGAAGCACCCGTTTGGTGAGGGCCCCTTCTTCGGCGGCAAGCGGATGAACGCTGGCGATGTGGCTATTCTACCTTTCCTGGTCCGCGCAAAGGCGTTTATGCCGGAGTTTAGTGGTGGGTACGATCTCTTTGCCCACTTTCCGCTGCTGAATGGGCTGGCAGAGGCTGGCATGGCGACACCGGAGGCGAAGTCGGTGTTTCGTACACTGGAGGAGTACAAGGAGCACATCCGTAAGCGCCAACGGAGGGCCCAGAGCGGGTAAGCGGCGCGTGTAGCATACCTATTCCACTCCCTGCTTTATGGACTTGCAGCGCAGTGATCCCATCGGCTCTCTGGGTGCTGCATTGCGTCCCATTTCGCCCATCTGCGCCACCCCGTTCGCGCGCGGCGCGCCTGTGCCTCCGGCGGTGCGAGGCGAAGTGAGCCCACGCGTACGCGTCTCCAATCGTCTCATCTACTCCCCACCCCGCATCTCCCCCCCCCTCCATCATCTCCCGCCTTTTTCTCGCTCGCTTGGTGCTGTCCACGTGAGTGGATGTGTTTGCTGCCTGTCAGCCGAGCACCACAGCAAGCAGGTCCCCTGCAGCCCTTTCGCTTCCGCTCCCCAGCCCATCTTTTTTCTCTGTTTGTTTTTCTGTTCTCCCCTACGGTCTCTCACCATCTCTCCCGTTAGAAGTGCGCCCGCTGCGTGTTGCTGCTGACGCTCCCCACTCATCTTGCACCGTCGATCCACAACTAAGCCACTGCGTGCCTGGCGTCGAG

>BPK206/0 clone 10|LinJ33_V3.0260|500 bp UPS + CDS + 500 bp DWS|thiol dependent reductase 1

AAGGCTCGTCGAGGGGATCGACGTGCCGACCATGCGAGGCTATTTGTTTTTGAGCTGCGCATCTGATCATGCATGTCTCTGTGCCGATCTCTGTGATTATCTCTGTGAGCGGTGTGCTCTACCAAAGCGGATTTCGGCTGCGAGGCTGCCATCGGCTCCAAAATCGCACGTTAGTCGTAGTTTGTCTTCCTAAAACTGAAGCGCATGCACACCAACGCGAGTGAGGTTGGGCGGAGATGGGGAAGGTACCACCTGAGGGCGAGTAGCGGAGAAGCGTGTCGTCTTGGGCGCCGTTCTGTGTCTCGTAGCGACTTGTATGCTCATGTGATGCGAGGTTTTGTTTTCTTTTTTGAACTGTTATCGTGTTCACTGCCGGTTCGCGGGCCTCATGTTCGGCAACGTTCTAGTTTTTTTCGCTGCTGTGGGACGTGGGCGACTGCGGCAAAGCATGTCGTCGTGCTGAGCTTTTTTCGCGTATGCTCAACTTTCCGCGAGTGAGTATGGCCGCCCGCGCGCTAAAGCTGTACGTGTCGGCAACGTGCCCGTTCTGCCACCGCGTGGAGATCGTCGCACGGGAGAAGCAGGTCTCATACGATCGCGTTGCTGTTGGGCTGCGCGAGGAGATGCCGCAATGGTACAAGCAGATTAACCCGCGTGAGACAGTGCCGACGCTGGAAGTCGGCAATGCGGATAAGCGGTTTATGTTCGAGTCGATGCTGATCGCGCAGTACCTGGACAACAGTGGCGCGCCCGCGGGCGCGCTGATGGGTTCCTCGGCGGCGCAACGGCACCAGATTGAGTTCTTCCTCGCACAGGTCGGCGATTTCATTGGCGCTGCGCACGGGCTGCTCCGCGATCCGCTGAGTGGTGAAAAGCGCAAGGCCATGGATGACAACGCGGCGTACGTGGACGGGCTGCTCGCGGCGAACCAGACGACGGGGCCATACTACTGCGACGGCGAGTTCACGATGGCGGACGTTGCGCTTGTGCCGTTCCTGGTGCGGCTGAAACCTGCTCTGATGTACTACGCCGGGTACGACGTGTTCTGCAAGGCGCCACGGATGAAGGCGCTGTGGGCCGCTGCTGCGCAACGCGCGTCTGTGCGTGAGACTTCGCCGACGGCGGCGCAGTGCATCGAGAACTACCGCCACCTGGTGCCGGAGAGCGCGCCAATGATGGGCGCCAATGGTGGGCACGTCCTGTACAGCAATCTTTTCTGTCCTTTTGTGGACCGCGCACGCCTCGCGTCTGAGCTGCGCAAGTTCCAGATGCACATAGTGGAGGTGCCGCTGCATCCACAGCCGGAGTGGTACAAATATATCAATCCCCGCGATACGGTGCCTGCGCTGTTTACACCGAGCGGCGAGGCTGTACACGAGTCGCAGCTGATTGTTCAGTACATCGACTGCGTGGCGACGAAGGGTAGTGCGCTGGTGCCGCGTGGGGACGCGGAGAAGGAGTACGAGGTGGGTTTCTTCGTGGAAAACGCTGGGTATTTCGTTGGAGGACTGATGTCGTGGATCATCCGTGGTGGCGAGGATGCGAAGGCTGAGCTTCAGTGGGCTGCTGGCGAGCTCGAGCAGCAGCTGGCGAAGCACCCGTTTGGTGAGGGCCCCTTCTTCGGCGGCAAGCGGATGAACGCTGGCGATGTGGCTATTCTACCTTTCCTGGTCCGCGCAAAGGCGTTTATGCCGGAGTTTAGTGGTGGGTACGATCTCTTTGCCCACTTTCCGCTGCTGAATGGGCTGGCAGAGGCTGGCATGGCGACACCGGAGGCGAAGTCGGTGTTTCGTACACTGGAGGAGTACAAGGAGCACATCCGTAAGCGCCAACGGAGGGCCCAGAGCGGGTAAGCGGCGCGTGTAGCATACCTATTCCACTCCCTGCTTTATGGACTTGCAGCGCAGTGATCCCATCGGCTCTCTGGGTGCTGCATTGCGTCCCATTTCGCCCATCTGCGCCACCCCGTTCGCGCGCGGCGCGCCTGTGCCTCCGGCGGTGCGAGGCGAAGTGAGCCCACGCGTACGCGTCTCCAATCGTCTCATCTACTCCCCACCCCGCATCTCCCCCCCCCTCCATCATCTCCCGCCTTTTTCTCGCTCGCTTGGTGCTGTCCACGTGAGTGGATGTGTTTGCTGCCTGTCAGCCGAGCACCACAGCAAGCAGGTCCCCTGCAGCCCTTTCGCTTCCGCTCCCCAGCCCATCTTTTTTCTCTGTTTGTTTTTCTGTTCTCCCCTACGGTCTCTCACCATCTCTCCCGTTAGAAGTGCGCCCGCTGCGTGTTGCTGCTGACGCTCCCCACTCATCTTGCACCGTCGATCCACAACTAAGCCACTGCGTGCCTGGCGTCGAG

>BPK275/0 clone 18|LinJ33_V3.0260|500 bp UPS + CDS + 500 bp DWS|thiol dependent reductase 1

AAGGCTCGTCGAGGGGATCGACGTGCCGACCATGCGAGGCTATTTGTTTTTGAGCTGCGCATCTGATCATGCATGTCTCTGTGCCGATCTCTGTGATTATCTCTGTGAGCGGTGTGCTCTACCAAAGCGGATTTCGGCTGCGAGGCTGCCATCGGCTCCAAAATCGCACGTTAGTCGTAGTTTGTCTTCCTAAAACTGAAGCGCATGCACACCAACGCGAGTGAGGTTGGGCGGAGATGGGGAAGGTACCACCTGAGGGCGAGTAGCGGAGAAGCGTGTCGTCTTGGGCGCCGTTCTGTGTCTCGTAGCGACTTGTATGCTCATGTGATGCGAGGTTTTGTTTTCTTTTTTGAACTGTTATCGTGTTCACTGCCGGTTCGCGGGCCTCATGTTCGGCAACGTTCTAGTTTTTTTCGCTGCTGTGGGACGTGGGCGACTGCGGCAAAGCATGTCGTCGTGCTGAGCTTTTTTCGCGTATGCTCAACTTTCCGCGAGTGAGTATGGCCGCCCGCGCGCTAAAGCTGTACGTGTCGGCAACGTGCCCGTTCTGCCACCGCGTGGAGATCGTCGCACGGGAGAAGCAGGTCTCATACGATCGCGTTGCTGTTGGGCTGCGCGAGGAGATGCCGCAATGGTACAAGCAGATTAACCCGCGTGAGACAGTGCCGACGCTGGAAGTCGGCAATGCGGATAAGCGGTTTATGTTCGAGTCGATGCTGATCGCGCAGTACCTGGACAACAGTGGCGCGCCCGCGGGCGCGCTGATGGGTTCCTCGGCGGCGCAACGGCACCAGATTGAGTTCTTCCTCGCACAGGTCGGCGATTTCATTGGCGCTGCGCACGGGCTGCTCCGCGATCCGCTGAGTGGTGAAAAGCGCAAGGCCATGGATGACAACGCGGCGTACGTGGACGGGCTGCTCGCGGCGAACCAGACGACGGGGCCATACTACTGCGACGGCGAGTTCACGATGGCGGACGTTGCGCTTGTGCCGTTCCTGGTGCGGCTGAAACCTGCTCTGATGTACTACGCCGGGTACGACGTGTTCTGCAAGGCGCCACGGATGAAGGCGCTGTGGGCCGCTGCTGCGCAACGCGCGTCTGTGCGTGAGACTTCGCCGACGGCGGCGCAGTGCATCGAGAACTACCGCCACCTGGTGCCGGAGAGCGCGCCAATGATGGGCGCCAATGGTGGGCACGTCCTGTACAGCAATCTTTTCTGTCCTTTTGTGGACCGCGCACGCCTCGCGTCTGAGCTGCGCAAGTTCCAGATGCACATAGTGGAGGTGCCGCTGCATCCACAGCCGGAGTGGTACAAATATATCAATCCCCGCGATACGGTGCCTGCGCTGTTTACACCGAGCGGCGAGGCTGTACACGAGTCGCAGCTGATTGTTCAGTACATCGACTGCGTGGCGACGAAGGGTAGTGCGCTGGTGCCGCGTGGGGACGCGGAGAAGGAGTACGAGGTGGGTTTCTTCGTGGAAAACGCTGGGTATTTCGTTGGAGGACTGATGTCGTGGATCATCCGTGGTGGCGAGGATGCGAAGGCTGAGCTTCAGTGGGCTGCTGGCGAGCTCGAGCAGCAGCTGGCGAAGCACCCGTTTGGTGAGGGCCCCTTCTTCGGCGGCAAGCGGATGAACGCTGGCGATGTGGCTATTCTACCTTTCCTGGTCCGCGCAAAGGCGTTTATGCCGGAGTTTAGTGGTGGGTACGATCTCTTTGCCCACTTTCCGCTGCTGAATGGGCTGGCAGAGGCTGGCATGGCGACACCGGAGGCGAAGTCGGTGTTTCGTACACTGGAGGAGTACAAGGAGCACATCCGTAAGCGCCAACGGAGGGCCCAGAGCGGGTAAGCGGCGCGTGTAGCATACCTATTCCACTCCCTGCTTTATGGACTTGCAGCGCAGTGATCCCATCGGCTCTCTGGGTGCTGCATTGCGTCCCATTTCGCCCATCTGCGCCACCCCGTTCGCGCGCGGCGCGCCTGTGCCTCCGGCGGTGCGAGGCGAAGTGAGCCCACGCGTACGCGTCTCCAATCGTCTCATCTACTCCCCACCCCGCATCTCCCCCCCCCTCCATCATCTCCCGCCTTTTTCTCGCTCGCTTGGTGCTGTCCACGTGAGTGGATGTGTTTGCTGCCTGTCAGCCGAGCACCACAGCAAGCAGGTCCCCTGCAGCCCTTTCGCTTCCGCTCCCCAGCCCATCTTTTTTCTCTGTTTGTTTTTCTGTTCTCCCCTACGGTCTCTCACCATCTCTCCCGTTAGAAGTGCGCCCGCTGCGTGTTGCTGCTGACGCTCCCCACTCATCTTGCACCGTCGATCCACAACTAAGCCACTGCGTGCCTGGCGTCGAG

>BPK282/0 clone 4|LinJ33_V3.0260|500 bp UPS + CDS + 500 bp DWS|thiol dependent reductase 1

AAGGCTCGTCGAGGGGATCGACGTGCCGACCATGCGAGGCTATTTGTTTTTGAGCTGCGCATCTGATCATGCATGTCTCTGTGCCGATCTCTGTGATTATCTCTGTGAGCGGTGTGCTCTACCAAAGCGGATTTCGGCTGCGAGGCTGCCATCGGCTCCAAAATCGCACGTTAGTCGTAGTTTGTCTTCCTAAAACTGAAGCGCATGCACACCAACGCGAGTGAGGTTGGGCGGAGATGGGGAAGGTACCACCTGAGGGCGAGTAGCGGAGAAGCGTGTCGTCTTGGGCGCCGTTCTGTGTCTCGTAGCGACTTGTATGCTCATGTGATGCGAGGTTTTGTTTTCTTTTTTGAACTGTTATCGTGTTCACTGCCGGTTCGCGGGCCTCATGTTCGGCAACGTTCTAGTTTTTTTCGCTGCTGTGGGACGTGGGCGACTGCGGCAAAGCATGTCGTCGTGCTGAGCTTTTTTCGCGTATGCTCAACTTTCCGCGAGTGAGTATGGCCGCCCGCGCGCTAAAGCTGTACGTGTCGGCAACGTGCCCGTTCTGCCACCGCGTGGAGATCGTCGCACGGGAGAAGCAGGTCTCATACGATCGCGTTGCTGTTGGGCTGCGCGAGGAGATGCCGCAATGGTACAAGCAGATTAACCCGCGTGAGACAGTGCCGACGCTGGAAGTCGGCAATGCGGATAAGCGGTTTATGTTCGAGTCGATGCTGATCGCGCAGTACCTGGACAACAGTGGCGCGCCCGCGGGCGCGCTGATGGGTTCCTCGGCGGCGCAACGGCACCAGATTGAGTTCTTCCTCGCACAGGTCGGCGATTTCATTGGCGCTGCGCACGGGCTGCTCCGCGATCCGCTGAGTGGTGAAAAGCGCAAGGCCATGGATGACAACGCGGCGTACGTGGACGGGCTGCTCGCGGCGAACCAGACGACGGGGCCATACTACTGCGACGGCGAGTTCACGATGGCGGACGTTGCGCTTGTGCCGTTCCTGGTGCGGCTGAAACCTGCTCTGATGTACTACGCCGGGTACGACGTGTTCTGCAAGGCGCCACGGATGAAGGCGCTGTGGGCCGCTGCTGCGCAACGCGCGTCTGTGCGTGAGACTTCGCCGACGGCGGCGCAGTGCATCGAGAACTACCGCCACCTGGTGCCGGAGAGCGCGCCAATGATGGGCGCCAATGGTGGGCACGTCCTGTACAGCAATCTTTTCTGTCCTTTTGTGGACCGCGCACGCCTCGCGTCTGAGCTGCGCAAGTTCCAGATGCACATAGTGGAGGTGCCGCTGCATCCACAGCCGGAGTGGTACAAATATATCAATCCCCGCGATACGGTGCCTGCGCTGTTTACACCGAGCGGCGAGGCTGTACACGAGTCGCAGCTGATTGTTCAGTACATCGACTGCGTGGCGACGAAGGGTAGTGCGCTGGTGCCGCGTGGGGACGCGGAGAAGGAGTACGAGGTGGGTTTCTTCGTGGAAAACGCTGGGTATTTCGTTGGAGGACTGATGTCGTGGATCATCCGTGGTGGCGAGGATGCGAAGGCTGAGCTTCAGTGGGCTGCTGGCGAGCTCGAGCAGCAGCTGGCGAAGCACCCGTTTGGTGAGGGCCCCTTCTTCGGCGGCAAGCGGATGAACGCTGGCGATGTGGCTATTCTACCTTTCCTGGTCCGCGCAAAGGCGTTTATGCCGGAGTTTAGTGGTGGGTACGATCTCTTTGCCCACTTTCCGCTGCTGAATGGGCTGGCAGAGGCTGGCATGGCGACACCGGAGGCGAAGTCGGTGTTTCGTACACTGGAGGAGTACAAGGAGCACATCCGTAAGCGCCAACGGAGGGCCCAGAGCGGGTAAGCGGCGCGTGTAGCATACCTATTCCACTCCCTGCTTTATGGACTTGCAGCGCAGTGATCCCATCGGCTCTCTGGGTGCTGCATTGCGTCCCATTTCGCCCATCTGCGCCACCCCGTTCGCGCGCGGCGCGCCTGTGCCTCCGGCGGTGCGAGGCGAAGTGAGCCCACGCGTACGCGTCTCCAATCGTCTCATCTACTCCCCACCCCGCATCTCCCCCCCCCTCCATCATCTCCCGCCTTTTTCTCGCTCGCTTGGTGCTGTCCACGTGAGTGGATGTGTTTGCTGCCTGTCAGCCGAGCACCACAGCAAGCAGGTCCCCTGCAGCCCTTTCGCTTCCGCTCCCCAGCCCATCTTTTTTCTCTGTTTGTTTTTCTGTTCTCCCCTACGGTCTCTCACCATCTCTCCCGTTAGAAGTGCGCCCGCTGCGTGTTGCTGCTGACGCTCCCCACTCATCTTGCACCGTCGATCCACAACTAAGCCACTGCGTGCCTGGCGTCGAG

>BPK294/0 clone 1|LinJ33_V3.0260|500 bp UPS + CDS + 500 bp DWS|thiol dependent reductase 1

AAGGCTCGTCGAGGGGATCGACGTGCCGACCATGCGAGGCTATTTGTTTTTGAGCTGCGCATCTGATCATGCATGTCTCTGTGCCGATCTCTGTGATTATCTCTGTGAGCGGTGTGCTCTACCAAAGCGGATTTCGGCTGCGAGGCTGCCATCGGCTCCAAAATCGCACGTTAGTCGTAGTTTGTCTTCCTAAAACTGAAGCGCATGCACACCAACGCGAGTGAGGTTGGGCGGAGATGGGGAAGGTACCACCTGAGGGCGAGTAGCGGAGAAGCGTGTCGTCTTGGGCGCCGTTCTGTGTCTCGTAGCGACTTGTATGCTCATGTGATGCGAGGTTTTGTTTTCTTTTTTGAACTGTTATCGTGTTCACTGCCGGTTCGCGGGCCTCATGTTCGGCAACGTTCTAGTTTTTTTCGCTGCTGTGGGACGTGGGCGACTGCGGCAAAGCATGTCGTCGTGCTGAGCTTTTTTCGCGTATGCTCAACTTTCCGCGAGTGAGTATGGCCGCCCGCGCGCTAAAGCTGTACGTGTCGGCAACGTGCCCGTTCTGCCACCGCGTGGAGATCGTCGCACGGGAGAAGCAGGTCTCATACGATCGCGTTGCTGTTGGGCTGCGCGAGGAGATGCCGCAATGGTACAAGCAGATTAACCCGCGTGAGACAGTGCCGACGCTGGAAGTCGGCAATGCGGATAAGCGGTTTATGTTCGAGTCGATGCTGATCGCGCAGTACCTGGACAACAGTGGCGCGCCCGCGGGCGCGCTGATGGGTTCCTCGGCGGCGCAACGGCACCAGATTGAGTTCTTCCTCGCACAGGTCGGCGATTTCATTGGCGCTGCGCACGGGCTGCTCCGCGATCCGCTGAGTGGTGAAAAGCGCAAGGCCATGGATGACAACGCGGCGTACGTGGACGGGCTGCTCGCGGCGAACCAGACGACGGGGCCATACTACTGCGACGGCGAGTTCACGATGGCGGACGTTGCGCTTGTGCCGTTCCTGGTGCGGCTGAAACCTGCTCTGATGTACTACGCCGGGTACGACGTGTTCTGCAAGGCGCCACGGATGAAGGCGCTGTGGGCCGCTGCTGCGCAACGCGCGTCTGTGCGTGAGACTTCGCCGACGGCGGCGCAGTGCATCGAGAACTACCGCCACCTGGTGCCGGAGAGCGCGCCAATGATGGGCGCCAATGGTGGGCACGTCCTGTACAGCAATCTTTTCTGTCCTTTTGTGGACCGCGCACGCCTCGCGTCTGAGCTGCGCAAGTTCCAGATGCACATAGTGGAGGTGCCGCTGCATCCACAGCCGGAGTGGTACAAATATATCAATCCCCGCGATACGGTGCCTGCGCTGTTTACACCGAGCGGCGAGGCTGTACACGAGTCGCAGCTGATTGTTCAGTACATCGACTGCGTGGCGACGAAGGGTAGTGCGCTGGTGCCGCGTGGGGACGCGGAGAAGGAGTACGAGGTGGGTTTCTTCGTGGAAAACGCTGGGTATTTCGTTGGAGGACTGATGTCGTGGATCATCCGTGGTGGCGAGGATGCGAAGGCTGAGCTTCAGTGGGCTGCTGGCGAGCTCGAGCAGCAGCTGGCGAAGCACCCGTTTGGTGAGGGCCCCTTCTTCGGCGGCAAGCGGATGAACGCTGGCGATGTGGCTATTCTACCTTTCCTGGTCCGCGCAAAGGCGTTTATGCCGGAGTTTAGTGGTGGGTACGATCTCTTTGCCCACTTTCCGCTGCTGAATGGGCTGGCAGAGGCTGGCATGGCGACACCGGAGGCGAAGTCGGTGTTTCGTACACTGGAGGAGTACAAGGAGCACATCCGTAAGCGCCAACGGAGGGCCCAGAGCGGGTAAGCGGCGCGTGTAGCATACCTATTCCACTCCCTGCTTTATGGACTTGCAGCGCAGTGATCCCATCGGCTCTCTGGGTGCTGCATTGCGTCCCATTTCGCCCATCTGCGCCACCCCGTTCGCGCGCGGCGCGCCTGTGCCTCCGGCGGTGCGAGGCGAAGTGAGCCCACGCGTACGCGTCTCCAATCGTCTCATCTACTCCCCACCCCGCATCTCCCCCCCCCTCCATCATCTCCCGCCTTTTTCTCGCTCGCTTGGTGCTGTCCACGTGAGTGGATGTGTTTGCTGCCTGTCAGCCGAGCACCACAGCAAGCAGGTCCCCTGCAGCCCTTTCGCTTCCGCTCCCCAGCCCATCTTTTTTCTCTGTTTGTTTTTCTGTTCTCCCCTACGGTCTCTCACCATCTCTCCCGTTAGAAGTGCGCCCGCTGCGTGTTGCTGCTGACGCTCCCCACTCATCTTGCACCGTCGATCCACAACTAAGCCACTGCGTGCCTGGCGTCGAG

>BPK298/0 clone 8|LinJ33_V3.0260|500 bp UPS + CDS + 500 bp DWS|thiol dependent reductase 1

AAGGCTCGTCGAGGGGATCGACGTGCCGACCATGCGAGGCTATTTGTTTTTGAGCTGCGCATCTGATCATGCATGTCTCTGTGCCGATCTCTGTGATTATCTCTGTGAGCGGTGTGCTCTACCAAAGCGGATTTCGGCTGCGAGGCTGCCATCGGCTCCAAAATCGCACGTTAGTCGTAGTTTGTCTTCCTAAAACTGAAGCGCATGCACACCAACGCGAGTGAGGTTGGGCGGAGATGGGGAAGGTACCACCTGAGGGCGAGTAGCGGAGAAGCGTGTCGTCTTGGGCGCCGTTCTGTGTCTCGTAGCGACTTGTATGCTCATGTGATGCGAGGTTTTGTTTTCTTTTTTGAACTGTTATCGTGTTCACTGCCGGTTCGCGGGCCTCATGTTCGGCAACGTTCTAGTTTTTTTCGCTGCTGTGGGACGTGGGCGACTGCGGCAAAGCATGTCGTCGTGCTGAGCTTTTTTCGCGTATGCTCAACTTTCCGCGAGTGAGTATGGCCGCCCGCGCGCTAAAGCTGTACGTGTCGGCAACGTGCCCGTTCTGCCACCGCGTGGAGATCGTCGCACGGGAGAAGCAGGTCTCATACGATCGCGTTGCTGTTGGGCTGCGCGAGGAGATGCCGCAATGGTACAAGCAGATTAACCCGCGTGAGACAGTGCCGACGCTGGAAGTCGGCAATGCGGATAAGCGGTTTATGTTCGAGTCGATGCTGATCGCGCAGTACCTGGACAACAGTGGCGCGCCCGCGGGCGCGCTGATGGGTTCCTCGGCGGCGCAACGGCACCAGATTGAGTTCTTCCTCGCACAGGTCGGCGATTTCATTGGCGCTGCGCACGGGCTGCTCCGCGATCCGCTGAGTGGTGAAAAGCGCAAGGCCATGGATGACAACGCGGCGTACGTGGACGGGCTGCTCGCGGCGAACCAGACGACGGGGCCATACTACTGCGACGGCGAGTTCACGATGGCGGACGTTGCGCTTGTGCCGTTCCTGGTGCGGCTGAAACCTGCTCTGATGTACTACGCCGGGTACGACGTGTTCTGCAAGGCGCCACGGATGAAGGCGCTGTGGGCCGCTGCTGCGCAACGCGCGTCTGTGCGTGAGACTTCGCCGACGGCGGCGCAGTGCATCGAGAACTACCGCCACCTGGTGCCGGAGAGCGCGCCAATGATGGGCGCCAATGGTGGGCACGTCCTGTACAGCAATCTTTTCTGTCCTTTTGTGGACCGCGCACGCCTCGCGTCTGAGCTGCGCAAGTTCCAGATGCACATAGTGGAGGTGCCGCTGCATCCACAGCCGGAGTGGTACAAATATATCAATCCCCGCGATACGGTGCCTGCGCTGTTTACACCGAGCGGCGAGGCTGTACACGAGTCGCAGCTGATTGTTCAGTACATCGACTGCGTGGCGACGAAGGGTAGTGCGCTGGTGCCGCGTGGGGACGCGGAGAAGGAGTACGAGGTGGGTTTCTTCGTGGAAAACGCTGGGTATTTCGTTGGAGGACTGATGTCGTGGATCATCCGTGGTGGCGAGGATGCGAAGGCTGAGCTTCAGTGGGCTGCTGGCGAGCTCGAGCAGCAGCTGGCGAAGCACCCGTTTGGTGAGGGCCCCTTCTTCGGCGGCAAGCGGATGAACGCTGGCGATGTGGCTATTCTACCTTTCCTGGTCCGCGCAAAGGCGTTTATGCCGGAGTTTAGTGGTGGGTACGATCTCTTTGCCCACTTTCCGCTGCTGAATGGGCTGGCAGAGGCTGGCATGGCGACACCGGAGGCGAAGTCGGTGTTTCGTACACTGGAGGAGTACAAGGAGCACATCCGTAAGCGCCAACGGAGGGCCCAGAGCGGGTAAGCGGCGCGTGTAGCATACCTATTCCACTCCCTGCTTTATGGACTTGCAGCGCAGTGATCCCATCGGCTCTCTGGGTGCTGCATTGCGTCCCATTTCGCCCATCTGCGCCACCCCGTTCGCGCGCGGCGCGCCTGTGCCTCCGGCGGTGCGAGGCGAAGTGAGCCCACGCGTACGCGTCTCCAATCGTCTCATCTACTCCCCACCCCGCATCTCCCCCCCCCTCCATCATCTCCCGCCTTTTTCTCGCTCGCTTGGTGCTGTCCACGTGAGTGGATGTGTTTGCTGCCTGTCAGCCGAGCACCACAGCAAGCAGGTCCCCTGCAGCCCTTTCGCTTCCGCTCCCCAGCCCATCTTTTTTCTCTGTTTGTTTTTCTGTTCTCCCCTACGGTCTCTCACCATCTCTCCCGTTAGAAGTGCGCCCGCTGCGTGTTGCTGCTGACGCTCCCCACTCATCTTGCACCGTCGATCCACAACTAAGCCACTGCGTGCCTGGCGTCGAG

### 10. PENTAMIDINE RESISTANCE PROTEIN 1

>BPK035/0 clone 1|LinJ31_V3.1460|500 bp UPS + CDS + 500 bp DWS|31|642945|5421|-|LinJ31.1810|LinJ31_V3.1460|Pentamidine resistance protein 1

ACTGCGCATTTCTGCAAGTCTTCATTTCAGCGAGGCAAACCTCCCGACCGGTAGTGTGTCCCTTTTTTCCCGCTGCTCTTATTGCAATGCCGTGTTGTTCGATTGCCTCTTGACGGAGGGGTGGAGAAGCTTGAAAGGAGAGAGACGATGCGGCGCACGAGGACTCGCTAGCGATCCGATCGGATGAAGTGATTGCCATGCTGCTTTTTTTTTTGGCCGCGTCGAGAGGTGCTGCGAGACGGGGGTGACATCGCGGCAAACATGTTCATCGGTTCCACGTTGTTTCACTCGCTGAAGGCAATGACTGGCATCAAGGCTTGCCGTTGATGACGTCATGTGAGAGAGCAAGGACTCTTCGGATTCTCTACCTTTCGCTCAAGCAGGCTTGCTTGCCATGCGTGCTGGTGGCTTCTCATGTCAGTGCCAGGTTTTACATCTGGTGCGTGTGACACGTGTGCGTGCATTTCTTCAGCTTGACTTTTTCTCATACCTCTTCGTCTATGAGCAGTCAACGACCGGAGATGCCAGAAGGAGCGGCGAGTGATAGCGTCTACTCGTTCGGCGAGGTTAACCGACGGTTGTGGCTTCTGCGTTCTAATTCGTTACCGAAGGGTAGCGCTTGTGATGATGCGTTGGTGGACTGGGCTGCGATACGCGGGTTGCTGGAGGAGGCTCCGGAAGAGCGGAGCGGCGTTTTATCGCGTTACCTGCTGAGGTGGCTGAACCCATATGTTATGCTTGCGTGGAGGGAGCGGCTGGAGGAGGCGTACATGCCTCCACCACAGCGGGCGCACCGCGCCGTGTGCTGTGGCGCTCTGCTAAGCCGCGCGTTTCGCGAGGAGGAGGTTCGCGGGGGCCGCGACGCGTGGCGTGCGCGCGTTGAAGCTGCGCTTCCAGTGCAGTGTGGAGCCTGCGATGACCCCTCGGGGGAAAGTGAAGTGATTGGCGGCGATTATAACGGGCGATGGGGTGGCGCGCGCGTGGCGGCTATCAACGAGCTGTCACGTGTGAGGTTCCGTGGTGAGGAGGGTGTCCATGGACGGCTGCGGTGGGTGGGGTACGTTCGATCGAGCGACACGCCGCACACGCTTTTGTGCGGTGTGGAGTGGGACGCTGACAGCGCGTTGCCGCCGTATCGTGCGCGTGTGGCTGGTGATGTGGCTGAGGAAGTAGTGCACGACGGGTGCGTGCAGGGCGAGCGTCTGTTTTACCAGGTTCAGGATGGGCGGGCGCGGTGCACGTGCGAGTACGTGCAGGATCTGGTGCCGGTTTCGACGATTGGAATCGCGGATACTGGGTGCCCGCGTATGCCTCACGCTCCGTCGCTGCTTTGGGCGCTACTCCGCACGTTTCGCTCCGATCTAATGGCGATACTGCCACCTTCGATTGCCGGTATGATATGTGAAGTGTCGACACCGTGGTTGCTGCAGCAGTTTGTGCTGTTCCTGCAGTCGAGCAAGGAGCGGGCGGGAGCGAGAAAAGGGCTGCTCTTCTTCTCGATTCTTGTGATCGTGAAGTTGGTGCAACCAGCGTTGATGAACAAGGAGATGCACCGCAGTCGGCGTGTTTCGAGCCTTTTCCGCACCTCGACACTCGCCCTGATTTTCGAAAAGTGTCTGACGATATCGCCGGATGCGCTGTCGAGACCGGACATGAACACTGGTCGTGTGCTAGCGATGGCGAGCTCTGACGTTGAGAACATCAAGGAATTTCCCACGCGGGTGATGTTCCTGTGGATGGCGCCGACTATGCTGACGCTGTACGTTGCCTACCTCTTCGTTCTCGTGGGGCCGAGTGCCCTGGCTGCTGTCCTGGTTTTTGCCGCTTTACTGCCTGTCCAAGGGGGGTTAACGAATGCTATGGGCGGCGCACAAGAGAACCTGTCGAGCTGCACGGATCAGCGCTTGCGTCGCACGAATGAACTGCTCTCCGGCATTCGTGTCGTGAAGATGATGGGATGGGAGTCGAAGTTTGTCTCGGCGATCGAAGATAACGCACGGGCCGATGAGTTGCGATTCCGACGAAGACTGCAGATGCGTCAAGTCGGGCTGTGGGCATGCGTGTTCTCTACTCCGACGTTCATGATCGCCGCTGTTCTCACTACCTACACCCTCAGCGGACACAAGCTTGACGCATCCGTGGTGTTCCCTCTCATCGCAGTTGTCAGTGCTATAACCTTCCCTGTAATGATGCTTCCGGAGGCCTTTACGTCGCTGGCGAAGTTCATTGTGTCGACGGGGCGCGTCACGCAGTTCTTGGAGTGCGACGACTCGCACATCATCGTCGAGCACGCGGAGCGCATGCTCAACAGCACAGGGAGTGGCGGCGCTGCTTCCGGGGACGCACCGCTGCCGAGTGCCGCTTCGGCCGACCTTGCAAGGGTGCTTGTGTCAGTGCCCGCCGCGCTTCCCGTGTACGAGCCGCGACACGTGGGGCTGCGCCGCGTGGCGCAGAGGATACTGTGCGCGGTGCTGCGCCGCCGTGTGCCGGTGGAGCTTCAGTGGCGGAGGGCGGCTGCTTCCCCTGCCATGGGTGAAGGCGGTAGGAAGCCTGCGCGCGTTGCTGTGGGGAACGATGATGGCGGTGTGGCTGTTGCGGGTGGTGAGAACGGGCGCGGCAGTGATAGTGGCGTGGCTCTGTACGCGATGGTGGACAGGGCTCTCCTGCGCGATGTGCGCGTGTCGTTCCCGCGCGCGCAGTTGACGGTTGTTGTTGGCGCGACGGGGAGCGGGAAGTCTGTGCTGCTGGCGACGCTTTTGGGTGCGTTCCGGTTCGAGGGGCATGTGAGCGTAGCGAAGTCGGTAGCTTACGTGCCGCAGCAGCCGTGGATCATGCAGGAAACGTTAGAGGCGAACATCACGTTCTTCGAGGGCGATCGGCGCGGCGCTGCTGGTGGCGCGGCGGTCGATAGCAATAGCGGCTCTGCGCAGGCGTTCTCGCCTCTGAGGTGCGCTGAGCAAGCTGCTGGCGCTGCTGCTGTGCCACGGTGCTTCAGCTGCGGCCGTGACGCGGCGAGCGAGCGTCTTGCGCGCGCTGTGCGGAGCTGCCAGCTGAATGCTGATGTGGCACTGATGGCGCGCGGGCTGGGGACAGAGATCGGCGAGCGTGGCATCAACCTGAGCGGTGGGCAGAAGGCGCGCGTGAGCCTTGCGCGCGCCGTGTATGCGGACCGCGACGTGTACCTGCTGGACGATCCGCTGTCGGCGCTGGACGCGCACGTTGGGCGGCGCGTGATGGACGAGGTTGTGCTGCGCGCGTTGTCGGGCAAGACGCGCGTGCTAGCGACGCACCAGCTGCAGGTGCTGCCGCACGCGGACCAGGTCGTGGTGATGCGCGAGGGCTGCGCCGTGTTTGCGGGCAGCTACGCTGCTTACGCTGCGTCGGAGTGGAAGGCGTACGTGGAGAGCGAGGAGGCCGCCGCCGCGAGCAAGAAGAGCGGGTGTGGCGGTGGTGATACTGTGGCGGAGTGCACCGAGGCCAGTTCTGCGTGGTCGTCTGCGGTGAACGTGGAGTCGTGTGTGGGCACGCGTGAGGAGCGTGACCACGCTCGGTGTGGCGGTGCCGATGGCCGTGCCAGCAAAGACGGCGGTGAGTGGGGGGAGAACGATGGCAGCCCTTTGCCGAGAATGCACTGGCAGGGCTCATGGGCCAGCGAGAACTCAGAGGCGGAAGGCTGCAACAATAGGGACTCTACGGTATGTAAAGGTGTTAGGCTCGCAAGCAGGTCTAAGGCCGCGCCGCGCACGGCGAAGGGCTCATCTGGGCTGGCTACGGGTGAGGATGCAGCGGACGGCGGCGACCTGATGACTGCGGAGGAGAAGGAGACAGGGCACACACCGTGGAGTGTGTACCGTGCCTACTTTGAGGCGGGCGGGGGCGTCCCGGTCGCAATTCAAATTGTGCTGCGCTACTTCGTTAGCGAAGCGCTCTCGACAGGCTCCAGCGTTTGGCTGACGCTGTGGTCAGTGAACTACTTTGGTTCTTCTCTCTCTACCAACGAACAGCTTGGCGTGTATCTTGGACTCGTCTTTGTGGTTGCTATCACAGTGTCTGCCAACGACCTGTTTATTTTTCAGTTCGCTCGCCGCGCTGCCTGCCGCTTGCACGCCATACTCTTGTACACGGTGAGCTCAGCCACGCTGACGTTTTTTGACCGCACACCACTGGGTCGCATTGTCAACCGGTTCAGCAGGGACGTGCACGTCCTTGACGATGAGCTGCCGGCGAACGTGATTCCCTTTTTGGGCATTACGGGATATGTCATGACGTCTTTAGCAGTGACGCTATACACTTCACCGCTTAGTGTGGTCGTGGTGCTGCTAGCGGTTTACGCTTTTGTGCGCCTGCTCAAGTTCTACGCGACAGTGGTGCGTGAGGTGCGACGGCGCAGCAGTGTGGGGCAGTCTCCGTTGCTCTCGCTGCTGGAGGAGGTGGTGCACGGGCGTGCGACGATCGCTGCATACGACAAGTCGCACGTGCTATTCGCGGAGGCGCTCCTGCGGCTGGACCTTGTGTACAGCTGCACCTACGTGGAGAAGGTTATGACGTTGTGGCTCGCTATCCGAATCGAGTACATTGCGTCATTGGCCGTTATTGCTGTGGGGCTGATCGGCGTTGTGGAGAAGCTGGTAGAGGCGTCGCCGGCGCTGCCGGAGGCGCGCGTGGGCCTGATCTCGCTGAGCCTCACCATGTGCCTCGATCTCAGCTGGTCGCTATCTGCACTCCTAGACCTTGCTGCCGCTGTCGAGGCGAGCATGAACAGTGTGCAGCGCGTTTGCCACTACATTGATCACGTGCCGCAGGAAGCGGCGCTCCTGGAGCCGCGGGACGCGTATGTGGCGCGCGCTGTGGCAGCGAGTGGCGGCAGCCGCAGGGGAGGAGGCGAGAGCTCCAGAAGTGCGAGCGACATCGTTGTGGCTGCCGGCGGTGATGACGACGAGGCCGCGCCTGCGCGACAGGGTGTTGCGGCAAGGAGCGGCGCGTTTGGCGCGCTGAGGCTGGAGCACGTGGACCTGCGGTACCGGCCAGGGCTGCCGCTGGTGCTGCGCGATGTCTGCTTTGCGATCGCCCCTGGGCAGAAGGTGGGCGTTGTGGGGCGGACGGGGAGCGGGAAGTCGACGCTGCTGCTGGCCTTCCTGCGGCTGGTGGAGGTAAGCGGCGGTCGCATGCTCGTCTGCGGGCGCGACGCGCGGACGTACACGCTTCCCGCTCTGCGGCGGCTGTTTTCCATGATCCCGCAGGATCCCGTATTGTTCGATGGCACGGTGCGCAGCAACGTGGATCCGTTCGGCGACGCGACGGACGAGGAGGTGCGTGCCGCGCTGGTGTCTGTGGGGTTTGTGGGGGTGGGCGGAAGCTCAGCTACCTTTCCTACAGCTTCTGGACTGCCGAGCGCTGCATCTGACAGCATGCCTGCGCTGGACACTGTGGTGCAGGGGGGCGGGTCGAACTTCAGCGTTGGGCAGCGCCAGCTGCTGTGCTTAGCGCGCGCGCTGCTGAAGAAGGGGAGTGCATTCATCCTGATGGACGAGGCGACGGCGAACGTGGACGCACAGCTGGACCAGACGGTGCAGCGTATTGTGGCGGAGCAGTTTGGCGCGTACACGGTGGTGACGATCGCGCACCGTCTGCACACGGTGGCGGCGTACGACGTAGTGCTTGTGATGGCGCGCGGGCGTGTGGTGGAGATGGGGCAGCCGCGGGCGCTGCTGGAGCGTCGCGACTCCGTGTTCTACGGTATGGTAGCGCAGAGCGCAGCGGTGGCTGATTCCCGTGAGGGCTTGTCGCGGGAGGATGCCGAGAGTGCGGCCAGTGGTGCGCGTTCGCGTGGCGTCAGTGACGGGGGCGCTGAGCTGGGGGCGGCTGGGCGCGAACACCGCGTGGAGGCCGCGGTCGCGTCGCTCCTGCGTCAGTGCAAGTGACCGGGATCGCACTCACGCGTCGCTGTATGCCTGCGTCTCTCGCTCTCTCTGGTGAGGGCTCGCAGCACCTCACTTGCCGCTTCGCTCGATCTATTCTTTCTCTTTTTGTGCTGCACTGGGCCGCACCACCACCGCATTGGTGACCGCACACCGCTCCCCACCGAGGCTTCCGCGGCTGCTGCACGTGCGTGGACGGCATTCGCGCAGCTCAGCTGCCGGCATTGGCTAGATTGACCGTCTTTTTTGCACAGCCACCCGTTGCAGCGGCGCGACACGGCTGGACCAGTTTTGTGTGCCCTTTGGCTGCTTAGCCCGCTGCGATAGGCGTGCCGGCCGGCAGCTGTCGTCTCCCCTGTCACCGGAGTGCTCGCTCTCCCCGCACCACATGCATTGCGAGCCGCCAGAGCTCTACACGATCATCTTAGTCTTTTGGCACACTTGCTGCGCCCTTTCGGCGCACGCTGCATGTTGCCCATCCGAGCCTGGGCATCTTGGAGATT

>BPK043/0 clone 2|LinJ31_V3.1460|500 bp UPS + CDS + 500 bp DWS|31|642945|5421|-|LinJ31.1810|LinJ31_V3.1460|Pentamidine resistance protein 1

ACTGCGCATTTCTGCAAGTCTTCATTTCAGCGAGGCAAACCTCCCGACCGGTAGTGTGTCCCTTTTTTCCCGCTGCTCTTATTGCAATGCCGTGTTGTTCGATTGCCTCTTGACGGAGGGGTGGAGAAGCTTGAAAGGAGAGAGACGATGCGGCGCACGAGGACTCGCTAGCGATCCGATCGGATGAAGTGATTGCCATGCTGCTTTTTTTTTTGGCCGCGTCGAGAGGTGCTGCGAGACGGGGGTGACATCGCGGCAAACATGTTCATCGGTTCCACGTTGTTTCACTCGCTGAAGGCAATGACTGGCATCAAGGCTTGCCGTTGATGACGTCATGTGAGAGAGCAAGGACTCTTCGGATTCTCTACCTTTCGCTCAAGCAGGCTTGCTTGCCATGCGTGCTGGTGGCTTCTCATGTCAGTGCCAGGTTTTACATCTGGTGCGTGTGACACGTGTGCGTGCATTTCTTCAGCTTGACTTTTTCTCATACCTCTTCGTCTATGAGCAGTCAACGACCGGAGATGCCAGAAGGAGCGGCGAGTGATAGCGTCTACTCGTTCGGCGAGGTTAACCGACGGTTGTGGCTTCTGCGTTCTAATTCGTTACCGAAGGGTAGCGCTTGTGATGATGCGTTGGTGGACTGGGCTGCGATACGCGGGTTGCTGGAGGAGGCTCCGGAAGAGCGGAGCGGCGTTTTATCGCGTTACCTGCTGAGGTGGCTGAACCCATATGTTATGCTTGCGTGGAGGGAGCGGCTGGAGGAGGCGTACATGCCTCCACCACAGCGGGCGCACCGCGCCGTGTGCTGTGGCGCTCTGCTAAGCCGCGCGTTTCGCGAGGAGGAGGTTCGCGGGGGCCGCGACGCGTGGCGTGCGCGCGTTGAAGCTGCGCTTCCAGTGCAGTGTGGAGCCTGCGATGACCCCTCGGGGGAAAGTGAAGTGATTGGCGGCGATTATAACGGGCGATGGGGTGGCGCGCGCGTGGCGGCTATCAACGAGCTGTCACGTGTGAGGTTCCGTGGTGAGGAGGGTGTCCATGGACGGCTGCGGTGGGTGGGGTACGTTCGATCGAGCGACACGCCGCACACGCTTTTGTGCGGTGTGGAGTGGGACGCTGACAGCGCGTTGCCGCCGTATCGTGCGCGTGTGGCTGGTGATGTGGCTGAGGAAGTAGTGCACGACGGGTGCGTGCAGGGCGAGCGTCTGTTTTACCAGGTTCAGGATGGGCGGGCGCGGTGCACGTGCGAGTACGTGCAGGATCTGGTGCCGGTTTCGACGATTGGAATCGCGGATACTGGGTGCCCGCGTATGCCTCACGCTCCGTCGCTGCTTTGGGCGCTACTCCGCACGTTTCGCTCCGATCTAATGGCGATACTGCCACCTTCGATTGCCGGTATGATATGTGAAGTGTCGACACCGTGGTTGCTGCAGCAGTTTGTGCTGTTCCTGCAGTCGAGCAAGGAGCGGGCGGGAGCGAGAAAAGGGCTGCTCTTCTTCTCGATTCTTGTGATCGTGAAGTTGGTGCAACCAGCGTTGATGAACAAGGAGATGCACCGCAGTCGGCGTGTTTCGAGCCTTTTCCGCACCTCGACACTCGCCCTGATTTTCGAAAAGTGTCTGACGATATCGCCGGATGCGCTGTCGAGACCGGACATGAACACTGGTCGTGTGCTAGCGATGGCGAGCTCTGACGTTGAGAACATCAAGGAATTTCCCACGCGGGTGATGTTCCTGTGGATGGCGCCGACTATGCTGACGCTGTACGTTGCCTACCTCTTCGTTCTCGTGGGGCCGAGTGCCCTGGCTGCTGTCCTGGTTTTTGCCGCTTTACTGCCTGTCCAAGGGGGGTTAACGAATGCTATGGGCGGCGCACAAGAGAACCTGTCGAGCTGCACGGATCAGCGCTTGCGTCGCACGAATGAACTGCTCTCCGGCATTCGTGTCGTGAAGATGATGGGATGGGAGTCGAAGTTTGTCTCGGCGATCGAAGATAACGCACGGGCCGATGAGTTGCGATTCCGACGAAGACTGCAGATGCGTCAAGTCGGGCTGTGGGCATGCGTGTTCTCTACTCCGACGTTCATGATCGCCGCTGTTCTCACTACCTACACCCTCAGCGGACACAAGCTTGACGCATCCGTGGTGTTCCCTCTCATCGCAGTTGTCAGTGCTATAACCTTCCCTGTAATGATGCTTCCGGAGGCCTTTACGTCGCTGGCGAAGTTCATTGTGTCGACGGGGCGCGTCACGCAGTTCTTGGAGTGCGACGACTCGCACATCATCGTCGAGCACGCGGAGCGCATGCTCAACAGCACAGGGAGTGGCGGCGCTGCTTCCGGGGACGCACCGCTGCCGAGTGCCGCTTCGGCCGACCTTGCAAGGGTGCTTGTGTCAGTGCCCGCCGCGCTTCCCGTGTACGAGCCGCGACACGTGGGGCTGCGCCGCGTGGCGCAGAGGATACTGTGCGCGGTGCTGCGCCGCCGTGTGCCGGTGGAGCTTCAGTGGCGGAGGGCGGCTGCTTCCCCTGCCATGGGTGAAGGCGGTAGGAAGCCTGCGCGCGTTGCTGTGGGGAACGATGATGGCGGTGTGGCTGTTGCGGGTGGTGAGAACGGGCGCGGCAGTGATAGTGGCGTGGCTCTGTACGCGATGGTGGACAGGGCTCTCCTGCGCGATGTGCGCGTGTCGTTCCCGCGCGCGCAGTTGACGGTTGTTGTTGGCGCGACGGGGAGCGGGAAGTCTGTGCTGCTGGCGACGCTTTTGGGTGCGTTCCGGTTCGAGGGGCATGTGAGCGTAGCGAAGTCGGTAGCTTACGTGCCGCAGCAGCCGTGGATCATGCAGGAAACGTTAGAGGCGAACATCACGTTCTTCGAGGGCGATCGGCGCGGCGCTGCTGGTGGCGCGGCGGTCGATAGCAATAGCGGCTCTGCGCAGGCGTTCTCGCCTCTGAGGTGCGCTGAGCAAGCTGCTGGCGCTGCTGCTGTGCCACGGTGCTTCAGCTGCGGCCGTGACGCGGCGAGCGAGCGTCTTGCGCGCGCTGTGCGGAGCTGCCAGCTGAATGCTGATGTGGCACTGATGGCGCGCGGGCTGGGGACAGAGATCGGCGAGCGTGGCATCAACCTGAGCGGTGGGCAGAAGGCGCGCGTGAGCCTTGCGCGCGCCGTGTATGCGGACCGCGACGTGTACCTGCTGGACGATCCGCTGTCGGCGCTGGACGCGCACGTTGGGCGGCGCGTGATGGACGAGGTTGTGCTGCGCGCGTTGTCGGGCAAGACGCGCGTGCTAGCGACGCACCAGCTGCAGGTGCTGCCGCACGCGGACCAGGTCGTGGTGATGCGCGAGGGCTGCGCCGTGTTTGCGGGCAGCTACGCTGCTTACGCTGCGTCGGAGTGGAAGGCGTACGTGGAGAGCGAGGAGGCCGCCGCCGCGAGCAAGAAGAGCGGGTGTGGCGGTGGTGATACTGTGGCGGAGTGCACCGAGGCCAGTTCTGCGTGGTCGTCTGCGGTGAACGTGGAGTCGTGTGTGGGCACGCGTGAGGAGCGTGACCACGCTCGGTGTGGCGGTGCCGATGGCCGTGCCAGCAAAGACGGCGGTGAGTGGGGGGAGAACGATGGCAGCCCTTTGCCGAGAATGCACTGGCAGGGCTCATGGGCCAGCGAGAACTCAGAGGCGGAAGGCTGCAACAATAGGGACTCTACGGTATGTAAAGGTGTTAGGCTCGCAAGCAGGTCTAAGGCCGCGCCGCGCACGGCGAAGGGCTCATCTGGGCTGGCTACGGGTGAGGATGCAGCGGACGGCGGCGACCTGATGACTGCGGAGGAGAAGGAGACAGGGCACACACCGTGGAGTGTGTACCGTGCCTACTTTGAGGCGGGCGGGGGCGTCCCGGTCGCAATTCAAATTGTGCTGCGCTACTTCGTTAGCGAAGCGCTCTCGACAGGCTCCAGCGTTTGGCTGACGCTGTGGTCAGTGAACTACTTTGGTTCTTCTCTCTCTACCAACGAACAGCTTGGCGTGTATCTTGGACTCGTCTTTGTGGTTGCTATCACAGTGTCTGCCAACGACCTGTTTATTTTTCAGTTCGCTCGCCGCGCTGCCTGCCGCTTGCACGCCATACTCTTGTACACGGTGAGCTCAGCCACGCTGACGTTTTTTGACCGCACACCACTGGGTCGCATTGTCAACCGGTTCAGCAGGGACGTGCACGTCCTTGACGATGAGCTGCCGGCGAACGTGATTCCCTTTTTGGGCATTACGGGATATGTCATGACGTCTTTAGCAGTGACGCTATACACTTCACCGCTTAGTGTGGTCGTGGTGCTGCTAGCGGTTTACGCTTTTGTGCGCCTGCTCAAGTTCTACGCGACAGTGGTGCGTGAGGTGCGACGGCGCAGCAGTGTGGGGCAGTCTCCGTTGCTCTCGCTGCTGGAGGAGGTGGTGCACGGGCGTGCGACGATCGCTGCATACGACAAGTCGCACGTGCTATTCGCGGAGGCGCTCCTGCGGCTGGACCTTGTGTACAGCTGCACCTACGTGGAGAAGGTTATGACGTTGTGGCTCGCTATCCGAATCGAGTACATTGCGTCATTGGCCGTTATTGCTGTGGGGCTGATCGGCGTTGTGGAGAAGCTGGTAGAGGCGTCGCCGGCGCTGCCGGAGGCGCGCGTGGGCCTGATCTCGCTGAGCCTCACCATGTGCCTCGATCTCAGCTGGTCGCTATCTGCACTCCTAGACCTTGCTGCCGCTGTCGAGGCGAGCATGAACAGTGTGCAGCGCGTTTGCCACTACATTGATCACGTGCCGCAGGAAGCGGCGCTCCTGGAGCCGCGGGACGCGTATGTGGCGCGCGCTGTGGCAGCGAGTGGCGGCAGCCGCAGGGGAGGAGGCGAGAGCTCCAGAAGTGCGAGCGACATCGTTGTGGCTGCCGGCGGTGATGACGACGAGGCCGCGCCTGCGCGACAGGGTGTTGCGGCAAGGAGCGGCGCGTTTGGCGCGCTGAGGCTGGAGCACGTGGACCTGCGGTACCGGCCAGGGCTGCCGCTGGTGCTGCGCGATGTCTGCTTTGCGATCGCCCCTGGGCAGAAGGTGGGCGTTGTGGGGCGGACGGGGAGCGGGAAGTCGACGCTGCTGCTGGCCTTCCTGCGGCTGGTGGAGGTAAGCGGCGGTCGCATGCTCGTCTGCGGGCGCGACGCGCGGACGTACACGCTTCCCGCTCTGCGGCGGCTGTTTTCCATGATCCCGCAGGATCCCGTATTGTTCGATGGCACGGTGCGCAGCAACGTGGATCCGTTCGGCGACGCGACGGACGAGGAGGTGCGTGCCGCGCTGGTGTCTGTGGGGTTTGTGGGGGTGGGCGGAAGCTCAGCTACCTTTCCTACAGCTTCTGGACTGCCGAGCGCTGCATCTGACAGCATGCCTGCGCTGGACACTGTGGTGCAGGGGGGCGGGTCGAACTTCAGCGTTGGGCAGCGCCAGCTGCTGTGCTTAGCGCGCGCGCTGCTGAAGAAGGGGAGTGCATTCATCCTGATGGACGAGGCGACGGCGAACGTGGACGCACAGCTGGACCAGACGGTGCAGCGTATTGTGGCGGAGCAGTTTGGCGCGTACACGGTGGTGACGATCGCGCACCGTCTGCACACGGTGGCGGCGTACGACGTAGTGCTTGTGATGGCGCGCGGGCGTGTGGTGGAGATGGGGCAGCCGCGGGCGCTGCTGGAGCGTCGCGACTCCGTGTTCTACGGTATGGTAGCGCAGAGCGCAGCGGTGGCTGATTCCCGTGAGGGCTTGTCGCGGGAGGATGCCGAGAGTGCGGCCAGTGGTGCGCGTTCGCGTGGCGTCAGTGACGGGGGCGCTGAGCTGGGGGCGGCTGGGCGCGAACACCGCGTGGAGGCCGCGGTCGCGTCGCTCCTGCGTCAGTGCAAGTGACCGGGATCGCACTCACGCGTCGCTGTATGCCTGCGTCTCTCGCTCTCTCTGGTGAGGGCTCGCAGCACCTCACTTGCCGCTTCGCTCGATCTATTCTTTCTCTTTTTGTGCTGCACTGGGCCGCACCACCACCGCATTGGTGACCGCACACCGCTCCCCACCGAGGCTTCCGCGGCTGCTGCACGTGCGTGGACGGCATTCGCGCAGCTCAGCTGCCGGCATTGGCTAGATTGACCGTCTTTTTTGCACAGCCACCCGTTGCAGCGGCGCGACACGGCTGGACCAGTTTTGTGTGCCCTTTGGCTGCTTAGCCCGCTGCGATAGGCGTGCCGGCCGGCAGCTGTCGTCTCCCCTGTCACCGGAGTGCTCGCTCTCCCCGCACCACATGCATTGCGAGCCGCCAGAGCTCTACACGATCATCTTAGTCTTTTGGCACACTTGCTGCGCCCTTTCGGCGCACGCTGCATGTTGCCCATCCGAGCCTGGGCATCTTGGAGATT

>BPK085/0 clone 8|LinJ31_V3.1460|500 bp UPS + CDS + 500 bp DWS|Pentamidine resistance protein 1

ACTGCGCATTTCTGCAAGTCTTCATTTCAGCGAGGCAAACCTCCCGACCGGTAGTGTGTCCCTTTTTTCCCGCTGCTCTTATTGCAATGCCGTGTTGTTCGATTGCCTCTTGACGGAGGGGTGGAGAAGCTTGAAAGGAGAGAGACGATGCGGCGCACGAGGACTCGCTAGCGATCCGATCGGATGAAGTGATTGCCATGCTGCTTTTTTTTTTGGCCGCGTCGAGAGGTGCTGCGAGACGGGGGTGACATCGCGGCAAACATGTTCATCGGTTCCACGTTGTTTCACTCGCTGAAGGCAATGACTGGCATCAAGGCTTGCCGTTGATGACGTCATGTGAGAGAGCAAGGACTCTTCGGATTCTCTACCTTTCGCTCAAGCAGGCTTGCTTGCCATGCGTGCTGGTGGCTTCTCATGTCAGTGCCAGGTTTTACATCTGGTGCGTGTGACACGTGTGCGTGCATTTCTTCAGCTTGACTTTTTCTCATACCTCTTCGTCTATGAGCAGTCAACGACCGGAGATGCCAGAAGGAGCGGCGAGTGATAGCGTCTACTCGTTCGGCGAGGTTAACCGACGGTTGTGGCTTCTGCGTTCTAATTCGTTACCGAAGGGTAGCGCTTGTGATGATGCGTTGGTGGACTGGGCTGCGATACGCGGGTTGCTGGAGGAGGCTCCGGAAGAGCGGAGCGGCGTTTTATCGCGTTACCTGCTGAGGTGGCTGAACCCATATGTTATGCTTGCGTGGAGGGAGCGGCTGGAGGAGGCGTACATGCCTCCACCACAGCGGGCGCACCGCGCCGTGTGCTGTGGCGCTCTGCTAAGCCGCGCGTTTCGCGAGGAGGAGGTTCGCGGGGGCCGCGACGCGTGGCGTGCGCGCGTTGAAGCTGCGCTTCCAGTGCAGTGTGGAGCCTGCGATGACCCCTCGGGGGAAAGTGAAGTGATTGGCGGCGATTATAACGGGCGATGGGGTGGCGCGCGCGTGGCGGCTATCAACGAGCTGTCACGTGTGAGGTTCCGTGGTGAGGAGGGTGTCCATGGACGGCTGCGGTGGGTGGGGTACGTTCGATCGAGCGACACGCCGCACACGCTTTTGTGCGGTGTGGAGTGGGACGCTGACAGCGCGTTGCCGCCGTATCGTGCGCGTGTGGCTGGTGATGTGGCTGAGGAAGTAGTGCACGACGGGTGCGTGCAGGGCGAGCGTCTGTTTTACCAGGTTCAGGATGGGCGGGCGCGGTGCACGTGCGAGTACGTGCAGGATCTGGTGCCGGTTTCGACGATTGGAATCGCGGATACTGGGTGCCCGCGTATGCCTCACGCTCCGTCGCTGCTTTGGGCGCTACTCCGCACGTTTCGCTCCGATCTAATGGCGATACTGCCACCTTCGATTGCCGGTATGATATGTGAAGTGTCGACACCGTGGTTGCTGCAGCAGTTTGTGCTGTTCCTGCAGTCGAGCAAGGAGCGGGCGGGAGCGAGAAAAGGGCTGCTCTTCTTCTCGATTCTTGTGATCGTGAAGTTGGTGCAACCAGCGTTGATGAACAAGGAGATGCACCGCAGTCGGCGTGTTTCGAGCCTTTTCCGCACCTCGACACTCGCCCTGATTTTCGAAAAGTGTCTGACGATATCGCCGGATGCGCTGTCGAGACCGGACATGAACACTGGTCGTGTGCTAGCGATGGCGAGCTCTGACGTTGAGAACATCAAGGAATTTCCCACGCGGGTGATGTTCCTGTGGATGGCGCCGACTATGCTGACGCTGTACGTTGCCTACCTCTTCGTTCTCGTGGGGCCGAGTGCCCTGGCTGCTGTCCTGGTTTTTGCCGCTTTACTGCCTGTCCAAGGGGGGTTAACGAATGCTATGGGCGGCGCACAAGAGAACCTGTCGAGCTGCACGGATCAGCGCTTGCGTCGCACGAATGAACTGCTCTCCGGCATTCGTGTCGTGAAGATGATGGGATGGGAGTCGAAGTTTGTCTCGGCGATCGAAGATAACGCACGGGCCGATGAGTTGCGATTCCGACGAAGACTGCAGATGCGTCAAGTCGGGCTGTGGGCATGCGTGTTCTCTACTCCGACGTTCATGATCGCCGCTGTTCTCACTACCTACACCCTCAGCGGACACAAGCTTGACGCATCCGTGGTGTTCCCTCTCATCGCAGTTGTCAGTGCTATAACCTTCCCTGTAATGATGCTTCCGGAGGCCTTTACGTCGCTGGCGAAGTTCATTGTGTCGACGGGGCGCGTCACGCAGTTCTTGGAGTGCGACGACTCGCACATCATCGTCGAGCACGCGGAGCGCATGCTCAACAGCACAGGGAGTGGCGGCGCTGCTTCCGGGGACGCACCGCTGCCGAGTGCCGCTTCGGCCGACCTTGCAAGGGTGCTTGTGTCAGTGCCCGCCGCGCTTCCCGTGTACGAGCCGCGACACGTGGGGCTGCGCCGCGTGGCGCAGAGGATACTGTGCGCGGTGCTGCGCCGCCGTGTGCCGGTGGAGCTTCAGTGGCGGAGGGCGGCTGCTTCCCCTGCCATGGGTGAAGGCGGTAGGAAGCCTGCGCGCGTTGCTGTGGGGAACGATGATGGCGGTGTGGCTGTTGCGGGTGGTGAGAACGGGCGCGGCAGTGATAGTGGCGTGGCTCTGTACGCGATGGTGGACAGGGCTCTCCTGCGCGATGTGCGCGTGTCGTTCCCGCGCGCGCAGTTGACGGTTGTTGTTGGCGCGACGGGGAGCGGGAAGTCTGTGCTGCTGGCGACGCTTTTGGGTGCGTTCCGGTTCGAGGGGCATGTGAGCGTAGCGAAGTCGGTAGCTTACGTGCCGCAGCAGCCGTGGATCATGCAGGAAACGTTAGAGGCGAACATCACGTTCTTCGAGGGCGATCGGCGCGGCGCTGCTGGTGGCGCGGCGGTCGATAGCAATAGCGGCTCTGCGCAGGCGTTCTCGCCTCTGAGGTGCGCTGAGCAAGCTGCTGGCGCTGCTGCTGTGCCACGGTGCTTCAGCTGCGGCCGTGACGCGGCGAGCGAGCGTCTTGCGCGCGCTGTGCGGAGCTGCCAGCTGAATGCTGATGTGGCACTGATGGCGCGCGGGCTGGGGACAGAGATCGGCGAGCGTGGCATCAACCTGAGCGGTGGGCAGAAGGCGCGCGTGAGCCTTGCGCGCGCCGTGTATGCGGACCGCGACGTGTACCTGCTGGACGATCCGCTGTCGGCGCTGGACGCGCACGTTGGGCGGCGCGTGATGGACGAGGTTGTGCTGCGCGCGTTGTCGGGCAAGACGCGCGTGCTAGCGACGCACCAGCTGCAGGTGCTGCCGCACGCGGACCAGGTCGTGGTGATGCGCGAGGGCTGCGCCGTGTTTGCGGGCAGCTACGCTGCTTACGCTGCGTCGGAGTGGAAGGCGTACGTGGAGAGCGAGGAGGCCGCCGCCGCGAGCAAGAAGAGCGGGTGTGGCGGTGGTGATACTGTGGCGGAGTGCACCGAGGCCAGTTCTGCGTGGTCGTCTGCGGTGAACGTGGAGTCGTGTGTGGGCACGCGTGAGGAGCGTGACCACGCTCGGTGTGGCGGTGCCGATGGCCGTGCCAGCAAAGACGGCGGTGAGTGGGGGGAGAACGATGGCAGCCCTTTGCCGAGAATGCACTGGCAGGGCTCATGGGCCAGCGAGAACTCAGAGGCGGAAGGCTGCAACAATAGGGACTCTACGGTATGTAAAGGTGTTAGGCTCGCAAGCAGGTCTAAGGCCGCGCCGCGCACGGCGAAGGGCTCATCTGGGCTGGCTACGGGTGAGGATGCAGCGGACGGCGGCGACCTGATGACTGCGGAGGAGAAGGAGACAGGGCACACACCGTGGAGTGTGTACCGTGCCTACTTTGAGGCGGGCGGGGGCGTCCCGGTCGCAATTCAAATTGTGCTGCGCTACTTCGTTAGCGAAGCGCTCTCGACAGGCTCCAGCGTTTGGCTGACGCTGTGGTCAGTGAACTACTTTGGTTCTTCTCTCTCTACCAACGAACAGCTTGGCGTGTATCTTGGACTCGTCTTTGTGGTTGCTATCACAGTGTCTGCCAACGACCTGTTTATTTTTCAGTTCGCTCGCCGCGCTGCCTGCCGCTTGCACGCCATACTCTTGTACACGGTGAGCTCAGCCACGCTGACGTTTTTTGACCGCACACCACTGGGTCGCATTGTCAACCGGTTCAGCAGGGACGTGCACGTCCTTGACGATGAGCTGCCGGCGAACGTGATTCCCTTTTTGGGCATTACGGGATATGTCATGACGTCTTTAGCAGTGACGCTATACACTTCACCGCTTAGTGTGGTCGTGGTGCTGCTAGCGGTTTACGCTTTTGTGCGCCTGCTCAAGTTCTACGCGACAGTGGTGCGTGAGGTGCGACGGCGCAGCAGTGTGGGGCAGTCTCCGTTGCTCTCGCTGCTGGAGGAGGTGGTGCACGGGCGTGCGACGATCGCTGCATACGACAAGTCGCACGTGCTATTCGCGGAGGCGCTCCTGCGGCTGGACCTTGTGTACAGCTGCACCTACGTGGAGAAGGTTATGACGTTGTGGCTCGCTATCCGAATCGAGTACATTGCGTCATTGGCCGTTATTGCTGTGGGGCTGATCGGCGTTGTGGAGAAGCTGGTAGAGGCGTCGCCGGCGCTGCCGGAGGCGCGCGTGGGCCTGATCTCGCTGAGCCTCACCATGTGCCTCGATCTCAGCTGGTCGCTATCTGCACTCCTAGACCTTGCTGCCGCTGTCGAGGCGAGCATGAACAGTGTGCAGCGCGTTTGCCACTACATTGATCACGTGCCGCAGGAAGCGGCGCTCCTGGAGCCGCGGGACGCGTATGTGGCGCGCGCTGTGGCAGCGAGTGGCGGCAGCCGCAGGGGAGGAGGCGAGAGCTCCAGAAGTGCGAGCGACATCGTTGTGGCTGCCGGCGGTGATGACGACGAGGCCGCGCCTGCGCGACAGGGTGTTGCGGCAAGGAGCGGCGCGTTTGGCGCGCTGAGGCTGGAGCACGTGGACCTGCGGTACCGGCCAGGGCTGCCGCTGGTGCTGCGCGATGTCTGCTTTGCGATCGCCCCTGGGCAGAAGGTGGGCGTTGTGGGGCGGACGGGGAGCGGGAAGTCGACGCTGCTGCTGGCCTTCCTGCGGCTGGTGGAGGTAAGCGGCGGTCGCATGCTCGTCTGCGGGCGCGACGCGCGGACGTACACGCTTCCCGCTCTGCGGCGGCTGTTTTCCATGATCCCGCAGGATCCCGTATTGTTCGATGGCACGGTGCGCAGCAACGTGGATCCGTTCGGCGACGCGACGGACGAGGAGGTGCGTGCCGCGCTGGTGTCTGTGGGGTTTGTGGGGGTGGGCGGAAGCTCAGCTACCTTTCCTACAGCTTCTGGACTGCCGAGCGCTGCATCTGACAGCATGCCTGCGCTGGACACTGTGGTGCAGGGGGGCGGGTCGAACTTCAGCGTTGGGCAGCGCCAGCTGCTGTGCTTAGCGCGCGCGCTGCTGAAGAAGGGGAGTGCATTCATCCTGATGGACGAGGCGACGGCGAACGTGGACGCACAGCTGGACCAGACGGTGCAGCGTATTGTGGCGGAGCAGTTTGGCGCGTACACGGTGGTGACGATCGCGCACCGTCTGCACACGGTGGCGGCGTACGACGTAGTGCTTGTGATGGCGCGCGGGCGTGTGGTGGAGATGGGGCAGCCGCGGGCGCTGCTGGAGCGTCGCGACTCCGTGTTCTACGGTATGGTAGCGCAGAGCGCAGCGGTGGCTGATTCCCGTGAGGGCTTGTCGCGGGAGGATGCCGAGAGTGCGGCCAGTGGTGCGCGTTCGCGTGGCGTCAGTGACGGGGGCGCTGAGCTGGGGGCGGCTGGGCGCGAACACCGCGTGGAGGCCGCGGTCGCGTCGCTCCTGCGTCAGTGCAAGTGACCGGGATCGCACTCACGCGTCGCTGTATGCCTGCGTCTCTCGCTCTCTCTGGTGAGGGCTCGCAGCACCTCACTTGCCGCTTCGCTCGATCTATTCTTTCTCTTTTTGTGCTGCACTGGGCCGCACCACCACCGCATTGGTGACCGCACACCGCTCCCCACCGAGGCTTCCGCGGCTGCTGCACGTGCGTGGACGGCATTCGCGCAGCTCAGCTGCCGGCATTGGCTAGATTGACCGTCTTTTTTGCACAGCCACCCGTTGCAGCGGCGCGACACGGCTGGACCAGTTTTGTGTGCCCTTTGGCTGCTTAGCCCGCTGCGATAGGCGTGCCGGCCGGCAGCTGTCGTCTCCCCTGTCACCGGAGTGCTCGCTCTCCCCGCACCACATGCATTGCGAGCCGCCAGAGCTCTACACGATCATCTTAGTCTTTTGGCACACTTGCTGCGCCCTTTCGGCGCACGCTGCATGTTGCCCATCCGAGCCTGGGCATCTTGGAGATT

>BPK087/0 clone 11|LinJ31_V3.1460|500 bp UPS + CDS + 500 bp DWS|Pentamidine resistance protein 1

ACTGCGCATTTCTGCAAGTCTTCATTTCAGCGAGGCAAACCTCCCGACCGGTAGTGTGTCCCTTTTTTCCCGCTGCTCTTATTGCAATGCCGTGTTGTTCGATTGCCTCTTGACGGAGGGGTGGAGAAGCTTGAAAGGAGAGAGACGATGCGGCGCACGAGGACTCGCTAGCGATCCGATCGGATGAAGTGATTGCCATGCTGCTTTTTTTTTTGGCCGCGTCGAGAGGTGCTGCGAGACGGGGGTGACATCGCGGCAAACATGTTCATCGGTTCCACGTTGTTTCACTCGCTGAAGGCAATGACTGGCATCAAGGCTTGCCGTTGATGACGTCATGTGAGAGAGCAAGGACTCTTCGGATTCTCTACCTTTCGCTCAAGCAGGCTTGCTTGCCATGCGTGCTGGTGGCTTCTCATGTCAGTGCCAGGTTTTACATCTGGTGCGTGTGACACGTGTGCGTGCATTTCTTCAGCTTGACTTTTTCTCATACCTCTTCGTCTATGAGCAGTCAACGACCGGAGATGCCAGAAGGAGCGGCGAGTGATAGCGTCTACTCGTTCGGCGAGGTTAACCGACGGTTGTGGCTTCTGCGTTCTAATTCGTTACCGAAGGGTAGCGCTTGTGATGATGCGTTGGTGGACTGGGCTGCGATACGCGGGTTGCTGGAGGAGGCTCCGGAAGAGCGGAGCGGCGTTTTATCGCGTTACCTGCTGAGGTGGCTGAACCCATATGTTATGCTTGCGTGGAGGGAGCGGCTGGAGGAGGCGTACATGCCTCCACCACAGCGGGCGCACCGCGCCGTGTGCTGTGGCGCTCTGCTAAGCCGCGCGTTTCGCGAGGAGGAGGTTCGCGGGGGCCGCGACGCGTGGCGTGCGCGCGTTGAAGCTGCGCTTCCAGTGCAGTGTGGAGCCTGCGATGACCCCTCGGGGGAAAGTGAAGTGATTGGCGGCGATTATAACGGGCGATGGGGTGGCGCGCGCGTGGCGGCTATCAACGAGCTGTCACGTGTGAGGTTCCGTGGTGAGGAGGGTGTCCATGGACGGCTGCGGTGGGTGGGGTACGTTCGATCGAGCGACACGCCGCACACGCTTTTGTGCGGTGTGGAGTGGGACGCTGACAGCGCGTTGCCGCCGTATCGTGCGCGTGTGGCTGGTGATGTGGCTGAGGAAGTAGTGCACGACGGGTGCGTGCAGGGCGAGCGTCTGTTTTACCAGGTTCAGGATGGGCGGGCGCGGTGCACGTGCGAGTACGTGCAGGATCTGGTGCCGGTTTCGACGATTGGAATCGCGGATACTGGGTGCCCGCGTATGCCTCACGCTCCGTCGCTGCTTTGGGCGCTACTCCGCACGTTTCGCTCCGATCTAATGGCGATACTGCCACCTTCGATTGCCGGTATGATATGTGAAGTGTCGACACCGTGGTTGCTGCAGCAGTTTGTGCTGTTCCTGCAGTCGAGCAAGGAGCGGGCGGGAGCGAGAAAAGGGCTGCTCTTCTTCTCGATTCTTGTGATCGTGAAGTTGGTGCAACCAGCGTTGATGAACAAGGAGATGCACCGCAGTCGGCGTGTTTCGAGCCTTTTCCGCACCTCGACACTCGCCCTGATTTTCGAAAAGTGTCTGACGATATCGCCGGATGCGCTGTCGAGACCGGACATGAACACTGGTCGTGTGCTAGCGATGGCGAGCTCTGACGTTGAGAACATCAAGGAATTTCCCACGCGGGTGATGTTCCTGTGGATGGCGCCGACTATGCTGACGCTGTACGTTGCCTACCTCTTCGTTCTCGTGGGGCCGAGTGCCCTGGCTGCTGTCCTGGTTTTTGCCGCTTTACTGCCTGTCCAAGGGGGGTTAACGAATGCTATGGGCGGCGCACAAGAGAACCTGTCGAGCTGCACGGATCAGCGCTTGCGTCGCACGAATGAACTGCTCTCCGGCATTCGTGTCGTGAAGATGATGGGATGGGAGTCGAAGTTTGTCTCGGCGATCGAAGATAACGCACGGGCCGATGAGTTGCGATTCCGACGAAGACTGCAGATGCGTCAAGTCGGGCTGTGGGCATGCGTGTTCTCTACTCCGACGTTCATGATCGCCGCTGTTCTCACTACCTACACCCTCAGCGGACACAAGCTTGACGCATCCGTGGTGTTCCCTCTCATCGCAGTTGTCAGTGCTATAACCTTCCCTGTAATGATGCTTCCGGAGGCCTTTACGTCGCTGGCGAAGTTCATTGTGTCGACGGGGCGCGTCACGCAGTTCTTGGAGTGCGACGACTCGCACATCATCGTCGAGCACGCGGAGCGCATGCTCAACAGCACAGGGAGTGGCGGCGCTGCTTCCGGGGACGCACCGCTGCCGAGTGCCGCTTCGGCCGACCTTGCAAGGGTGCTTGTGTCAGTGCCCGCCGCGCTTCCCGTGTACGAGCCGCGACACGTGGGGCTGCGCCGCGTGGCGCAGAGGATACTGTGCGCGGTGCTGCGCCGCCGTGTGCCGGTGGAGCTTCAGTGGCGGAGGGCGGCTGCTTCCCCTGCCATGGGTGAAGGCGGTAGGAAGCCTGCGCGCGTTGCTGTGGGGAACGATGATGGCGGTGTGGCTGTTGCGGGTGGTGAGAACGGGCGCGGCAGTGATAGTGGCGTGGCTCTGTACGCGATGGTGGACAGGGCTCTCCTGCGCGATGTGCGCGTGTCGTTCCCGCGCGCGCAGTTGACGGTTGTTGTTGGCGCGACGGGGAGCGGGAAGTCTGTGCTGCTGGCGACGCTTTTGGGTGCGTTCCGGTTCGAGGGGCATGTGAGCGTAGCGAAGTCGGTAGCTTACGTGCCGCAGCAGCCGTGGATCATGCAGGAAACGTTAGAGGCGAACATCACGTTCTTCGAGGGCGATCGGCGCGGCGCTGCTGGTGGCGCGGCGGTCGATAGCAATAGCGGCTCTGCGCAGGCGTTCTCGCCTCTGAGGTGCGCTGAGCAAGCTGCTGGCGCTGCTGCTGTGCCACGGTGCTTCAGCTGCGGCCGTGACGCGGCGAGCGAGCGTCTTGCGCGCGCTGTGCGGAGCTGCCAGCTGAATGCTGATGTGGCACTGATGGCGCGCGGGCTGGGGACAGAGATCGGCGAGCGTGGCATCAACCTGAGCGGTGGGCAGAAGGCGCGCGTGAGCCTTGCGCGCGCCGTGTATGCGGACCGCGACGTGTACCTGCTGGACGATCCGCTGTCGGCGCTGGACGCGCACGTTGGGCGGCGCGTGATGGACGAGGTTGTGCTGCGCGCGTTGTCGGGCAAGACGCGCGTGCTAGCGACGCACCAGCTGCAGGTGCTGCCGCACGCGGACCAGGTCGTGGTGATGCGCGAGGGCTGCGCCGTGTTTGCGGGCAGCTACGCTGCTTACGCTGCGTCGGAGTGGAAGGCGTACGTGGAGAGCGAGGAGGCCGCCGCCGCGAGCAAGAAGAGCGGGTGTGGCGGTGGTGATACTGTGGCGGAGTGCACCGAGGCCAGTTCTGCGTGGTCGTCTGCGGTGAACGTGGAGTCGTGTGTGGGCACGCGTGAGGAGCGTGACCACGCTCGGTGTGGCGGTGCCGATGGCCGTGCCAGCAAAGACGGCGGTGAGTGGGGGGAGAACGATGGCAGCCCTTTGCCGAGAATGCACTGGCAGGGCTCATGGGCCAGCGAGAACTCAGAGGCGGAAGGCTGCAACAATAGGGACTCTACGGTATGTAAAGGTGTTAGGCTCGCAAGCAGGTCTAAGGCCGCGCCGCGCACGGCGAAGGGCTCATCTGGGCTGGCTACGGGTGAGGATGCAGCGGACGGCGGCGACCTGATGACTGCGGAGGAGAAGGAGACAGGGCACACACCGTGGAGTGTGTACCGTGCCTACTTTGAGGCGGGCGGGGGCGTCCCGGTCGCAATTCAAATTGTGCTGCGCTACTTCGTTAGCGAAGCGCTCTCGACAGGCTCCAGCGTTTGGCTGACGCTGTGGTCAGTGAACTACTTTGGTTCTTCTCTCTCTACCAACGAACAGCTTGGCGTGTATCTTGGACTCGTCTTTGTGGTTGCTATCACAGTGTCTGCCAACGACCTGTTTATTTTTCAGTTCGCTCGCCGCGCTGCCTGCCGCTTGCACGCCATACTCTTGTACACGGTGAGCTCAGCCACGCTGACGTTTTTTGACCGCACACCACTGGGTCGCATTGTCAACCGGTTCAGCAGGGACGTGCACGTCCTTGACGATGAGCTGCCGGCGAACGTGATTCCCTTTTTGGGCATTACGGGATATGTCATGACGTCTTTAGCAGTGACGCTATACACTTCACCGCTTAGTGTGGTCGTGGTGCTGCTAGCGGTTTACGCTTTTGTGCGCCTGCTCAAGTTCTACGCGACAGTGGTGCGTGAGGTGCGACGGCGCAGCAGTGTGGGGCAGTCTCCGTTGCTCTCGCTGCTGGAGGAGGTGGTGCACGGGCGTGCGACGATCGCTGCATACGACAAGTCGCACGTGCTATTCGCGGAGGCGCTCCTGCGGCTGGACCTTGTGTACAGCTGCACCTACGTGGAGAAGGTTATGACGTTGTGGCTCGCTATCCGAATCGAGTACATTGCGTCATTGGCCGTTATTGCTGTGGGGCTGATCGGCGTTGTGGAGAAGCTGGTAGAGGCGTCGCCGGCGCTGCCGGAGGCGCGCGTGGGCCTGATCTCGCTGAGCCTCACCATGTGCCTCGATCTCAGCTGGTCGCTATCTGCACTCCTAGACCTTGCTGCCGCTGTCGAGGCGAGCATGAACAGTGTGCAGCGCGTTTGCCACTACATTGATCACGTGCCGCAGGAAGCGGCGCTCCTGGAGCCGCGGGACGCGTATGTGGCGCGCGCTGTGGCAGCGAGTGGCGGCAGCCGCAGGGGAGGAGGCGAGAGCTCCAGAAGTGCGAGCGACATCGTTGTGGCTGCCGGCGGTGATGACGACGAGGCCGCGCCTGCGCGACAGGGTGTTGCGGCAAGGAGCGGCGCGTTTGGCGCGCTGAGGCTGGAGCACGTGGACCTGCGGTACCGGCCAGGGCTGCCGCTGGTGCTGCGCGATGTCTGCTTTGCGATCGCCCCTGGGCAGAAGGTGGGCGTTGTGGGGCGGACGGGGAGCGGGAAGTCGACGCTGCTGCTGGCCTTCCTGCGGCTGGTGGAGGTAAGCGGCGGTCGCATGCTCGTCTGCGGGCGCGACGCGCGGACGTACACGCTTCCCGCTCTGCGGCGGCTGTTTTCCATGATCCCGCAGGATCCCGTATTGTTCGATGGCACGGTGCGCAGCAACGTGGATCCGTTCGGCGACGCGACGGACGAGGAGGTGCGTGCCGCGCTGGTGTCTGTGGGGTTTGTGGGGGTGGGCGGAAGCTCAGCTACCTTTCCTACAGCTTCTGGACTGCCGAGCGCTGCATCTGACAGCATGCCTGCGCTGGACACTGTGGTGCAGGGGGGCGGGTCGAACTTCAGCGTTGGGCAGCGCCAGCTGCTGTGCTTAGCGCGCGCGCTGCTGAAGAAGGGGAGTGCATTCATCCTGATGGACGAGGCGACGGCGAACGTGGACGCACAGCTGGACCAGACGGTGCAGCGTATTGTGGCGGAGCAGTTTGGCGCGTACACGGTGGTGACGATCGCGCACCGTCTGCACACGGTGGCGGCGTACGACGTAGTGCTTGTGATGGCGCGCGGGCGTGTGGTGGAGATGGGGCAGCCGCGGGCGCTGCTGGAGCGTCGCGACTCCGTGTTCTACGGTATGGTAGCGCAGAGCGCAGCGGTGGCTGATTCCCGTGAGGGCTTGTCGCGGGAGGATGCCGAGAGTGCGGCCAGTGGTGCGCGTTCGCGTGGCGTCAGTGACGGGGGCGCTGAGCTGGGGGCGGCTGGGCGCGAACACCGCGTGGAGGCCGCGGTCGCGTCGCTCCTGCGTCAGTGCAAGTGACCGGGATCGCACTCACGCGTCGCTGTATGCCTGCGTCTCTCGCTCTCTCTGGTGAGGGCTCGCAGCACCTCACTTGCCGCTTCGCTCGATCTATTCTTTCTCTTTTTGTGCTGCACTGGGCCGCACCACCACCGCATTGGTGACCGCACACCGCTCCCCACCGAGGCTTCCGCGGCTGCTGCACGTGCGTGGACGGCATTCGCGCAGCTCAGCTGCCGGCATTGGCTAGATTGACCGTCTTTTTTGCACAGCCACCCGTTGCAGCGGCGCGACACGGCTGGACCAGTTTTGTGTGCCCTTTGGCTGCTTAGCCCGCTGCGATAGGCGTGCCGGCCGGCAGCTGTCGTCTCCCCTGTCACCGGAGTGCTCGCTCTCCCCGCACCACATGCATTGCGAGCCGCCAGAGCTCTACACGATCATCTTAGTCTTTTGGCACACTTGCTGCGCCCTTTCGGCGCACGCTGCATGTTGCCCATCCGAGCCTGGGCATCTTGGAGATT

>BPK178/0 clone 3|LinJ31_V3.1460|500 bp UPS + CDS + 500 bp DWS|Pentamidine resistance protein 1

ACTGCGCATTTCTGCAAGTCTTCATTTCAGCGAGGCAAACCTCCCGACCGGTAGTGTGTCCCTTTTTTCCCGCTGCTCTTATTGCAATGCCGTGTTGTTCGATTGCCTCTTGACGGAGGGGTGGAGAAGCTTGAAAGGAGAGAGACGATGCGGCGCACGAGGACTCGCTAGCGATCCGATCGGATGAAGTGATTGCCATGCTGCTTTTTTTTTTGGCCGCGTCGAGAGGTGCTGCGAGACGGGGGTGACATCGCGGCAAACATGTTCATCGGTTCCACGTTGTTTCACTCGCTGAAGGCAATGACTGGCATCAAGGCTTGCCGTTGATGACGTCATGTGAGAGAGCAAGGACTCTTCGGATTCTCTACCTTTCGCTCAAGCAGGCTTGCTTGCCATGCGTGCTGGTGGCTTCTCATGTCAGTGCCAGGTTTTACATCTGGTGCGTGTGACACGTGTGCGTGCATTTCTTCAGCTTGACTTTTTCTCATACCTCTTCGTCTATGAGCAGTCAACGACCGGAGATGCCAGAAGGAGCGGCGAGTGATAGCGTCTACTCGTTCGGCGAGGTTAACCGACGGTTGTGGCTTCTGCGTTCTAATTCGTTACCGAAGGGTAGCGCTTGTGATGATGCGTTGGTGGACTGGGCTGCGATACGCGGGTTGCTGGAGGAGGCTCCGGAAGAGCGGAGCGGCGTTTTATCGCGTTACCTGCTGAGGTGGCTGAACCCATATGTTATGCTTGCGTGGAGGGAGCGGCTGGAGGAGGCGTACATGCCTCCACCACAGCGGGCGCACCGCGCCGTGTGCTGTGGCGCTCTGCTAAGCCGCGCGTTTCGCGAGGAGGAGGTTCGCGGGGGCCGCGACGCGTGGCGTGCGCGCGTTGAAGCTGCGCTTCCAGTGCAGTGTGGAGCCTGCGATGACCCCTCGGGGGAAAGTGAAGTGATTGGCGGCGATTATAACGGGCGATGGGGTGGCGCGCGCGTGGCGGCTATCAACGAGCTGTCACGTGTGAGGTTCCGTGGTGAGGAGGGTGTCCATGGACGGCTGCGGTGGGTGGGGTACGTTCGATCGAGCGACACGCCGCACACGCTTTTGTGCGGTGTGGAGTGGGACGCTGACAGCGCGTTGCCGCCGTATCGTGCGCGTGTGGCTGGTGATGTGGCTGAGGAAGTAGTGCACGACGGGTGCGTGCAGGGCGAGCGTCTGTTTTACCAGGTTCAGGATGGGCGGGCGCGGTGCACGTGCGAGTACGTGCAGGATCTGGTGCCGGTTTCGACGATTGGAATCGCGGATACTGGGTGCCCGCGTATGCCTCACGCTCCGTCGCTGCTTTGGGCGCTACTCCGCACGTTTCGCTCCGATCTAATGGCGATACTGCCACCTTCGATTGCCGGTATGATATGTGAAGTGTCGACACCGTGGTTGCTGCAGCAGTTTGTGCTGTTCCTGCAGTCGAGCAAGGAGCGGGCGGGAGCGAGAAAAGGGCTGCTCTTCTTCTCGATTCTTGTGATCGTGAAGTTGGTGCAACCAGCGTTGATGAACAAGGAGATGCACCGCAGTCGGCGTGTTTCGAGCCTTTTCCGCACCTCGACACTCGCCCTGATTTTCGAAAAGTGTCTGACGATATCGCCGGATGCGCTGTCGAGACCGGACATGAACACTGGTCGTGTGCTAGCGATGGCGAGCTCTGACGTTGAGAACATCAAGGAATTTCCCACGCGGGTGATGTTCCTGTGGATGGCGCCGACTATGCTGACGCTGTACGTTGCCTACCTCTTCGTTCTCGTGGGGCCGAGTGCCCTGGCTGCTGTCCTGGTTTTTGCCGCTTTACTGCCTGTCCAAGGGGGGTTAACGAATGCTATGGGCGGCGCACAAGAGAACCTGTCGAGCTGCACGGATCAGCGCTTGCGTCGCACGAATGAACTGCTCTCCGGCATTCGTGTCGTGAAGATGATGGGATGGGAGTCGAAGTTTGTCTCGGCGATCGAAGATAACGCACGGGCCGATGAGTTGCGATTCCGACGAAGACTGCAGATGCGTCAAGTCGGGCTGTGGGCATGCGTGTTCTCTACTCCGACGTTCATGATCGCCGCTGTTCTCACTACCTACACCCTCAGCGGACACAAGCTTGACGCATCCGTGGTGTTCCCTCTCATCGCAGTTGTCAGTGCTATAACCTTCCCTGTAATGATGCTTCCGGAGGCCTTTACGTCGCTGGCGAAGTTCATTGTGTCGACGGGGCGCGTCACGCAGTTCTTGGAGTGCGACGACTCGCACATCATCGTCGAGCACGCGGAGCGCATGCTCAACAGCACAGGGAGTGGCGGCGCTGCTTCCGGGGACGCACCGCTGCCGAGTGCCGCTTCGGCCGACCTTGCAAGGGTGCTTGTGTCAGTGCCCGCCGCGCTTCCCGTGTACGAGCCGCGACACGTGGGGCTGCGCCGCGTGGCGCAGAGGATACTGTGCGCGGTGCTGCGCCGCCGTGTGCCGGTGGAGCTTCAGTGGCGGAGGGCGGCTGCTTCCCCTGCCATGGGTGAAGGCGGTAGGAAGCCTGCGCGCGTTGCTGTGGGGAACGATGATGGCGGTGTGGCTGTTGCGGGTGGTGAGAACGGGCGCGGCAGTGATAGTGGCGTGGCTCTGTACGCGATGGTGGACAGGGCTCTCCTGCGCGATGTGCGCGTGTCGTTCCCGCGCGCGCAGTTGACGGTTGTTGTTGGCGCGACGGGGAGCGGGAAGTCTGTGCTGCTGGCGACGCTTTTGGGTGCGTTCCGGTTCGAGGGGCATGTGAGCGTAGCGAAGTCGGTAGCTTACGTGCCGCAGCAGCCGTGGATCATGCAGGAAACGTTAGAGGCGAACATCACGTTCTTCGAGGGCGATCGGCGCGGCGCTGCTGGTGGCGCGGCGGTCGATAGCAATAGCGGCTCTGCGCAGGCGTTCTCGCCTCTGAGGTGCGCTGAGCAAGCTGCTGGCGCTGCTGCTGTGCCACGGTGCTTCAGCTGCGGCCGTGACGCGGCGAGCGAGCGTCTTGCGCGCGCTGTGCGGAGCTGCCAGCTGAATGCTGATGTGGCACTGATGGCGCGCGGGCTGGGGACAGAGATCGGCGAGCGTGGCATCAACCTGAGCGGTGGGCAGAAGGCGCGCGTGAGCCTTGCGCGCGCCGTGTATGCGGACCGCGACGTGTACCTGCTGGACGATCCGCTGTCGGCGCTGGACGCGCACGTTGGGCGGCGCGTGATGGACGAGGTTGTGCTGCGCGCGTTGTCGGGCAAGACGCGCGTGCTAGCGACGCACCAGCTGCAGGTGCTGCCGCACGCGGACCAGGTCGTGGTGATGCGCGAGGGCTGCGCCGTGTTTGCGGGCAGCTACGCTGCTTACGCTGCGTCGGAGTGGAAGGCGTACGTGGAGAGCGAGGAGGCCGCCGCCGCGAGCAAGAAGAGCGGGTGTGGCGGTGGTGATACTGTGGCGGAGTGCACCGAGGCCAGTTCTGCGTGGTCGTCTGCGGTGAACGTGGAGTCGTGTGTGGGCACGCGTGAGGAGCGTGACCACGCTCGGTGTGGCGGTGCCGATGGCCGTGCCAGCAAAGACGGCGGTGAGTGGGGGGAGAACGATGGCAGCCCTTTGCCGAGAATGCACTGGCAGGGCTCATGGGCCAGCGAGAACTCAGAGGCGGAAGGCTGCAACAATAGGGACTCTACGGTATGTAAAGGTGTTAGGCTCGCAAGCAGGTCTAAGGCCGCGCCGCGCACGGCGAAGGGCTCATCTGGGCTGGCTACGGGTGAGGATGCAGCGGACGGCGGCGACCTGATGACTGCGGAGGAGAAGGAGACAGGGCACACACCGTGGAGTGTGTACCGTGCCTACTTTGAGGCGGGCGGGGGCGTCCCGGTCGCAATTCAAATTGTGCTGCGCTACTTCGTTAGCGAAGCGCTCTCGACAGGCTCCAGCGTTTGGCTGACGCTGTGGTCAGTGAACTACTTTGGTTCTTCTCTCTCTACCAACGAACAGCTTGGCGTGTATCTTGGACTCGTCTTTGTGGTTGCTATCACAGTGTCTGCCAACGACCTGTTTATTTTTCAGTTCGCTCGCCGCGCTGCCTGCCGCTTGCACGCCATACTCTTGTACACGGTGAGCTCAGCCACGCTGACGTTTTTTGACCGCACACCACTGGGTCGCATTGTCAACCGGTTCAGCAGGGACGTGCACGTCCTTGACGATGAGCTGCCGGCGAACGTGATTCCCTTTTTGGGCATTACGGGATATGTCATGACGTCTTTAGCAGTGACGCTATACACTTCACCGCTTAGTGTGGTCGTGGTGCTGCTAGCGGTTTACGCTTTTGTGCGCCTGCTCAAGTTCTACGCGACAGTGGTGCGTGAGGTGCGACGGCGCAGCAGTGTGGGGCAGTCTCCGTTGCTCTCGCTGCTGGAGGAGGTGGTGCACGGGCGTGCGACGATCGCTGCATACGACAAGTCGCACGTGCTATTCGCGGAGGCGCTCCTGCGGCTGGACCTTGTGTACAGCTGCACCTACGTGGAGAAGGTTATGACGTTGTGGCTCGCTATCCGAATCGAGTACATTGCGTCATTGGCCGTTATTGCTGTGGGGCTGATCGGCGTTGTGGAGAAGCTGGTAGAGGCGTCGCCGGCGCTGCCGGAGGCGCGCGTGGGCCTGATCTCGCTGAGCCTCACCATGTGCCTCGATCTCAGCTGGTCGCTATCTGCACTCCTAGACCTTGCTGCCGCTGTCGAGGCGAGCATGAACAGTGTGCAGCGCGTTTGCCACTACATTGATCACGTGCCGCAGGAAGCGGCGCTCCTGGAGCCGCGGGACGCGTATGTGGCGCGCGCTGTGGCAGCGAGTGGCGGCAGCCGCAGGGGAGGAGGCGAGAGCTCCAGAAGTGCGAGCGACATCGTTGTGGCTGCCGGCGGTGATGACGACGAGGCCGCGCCTGCGCGACAGGGTGTTGCGGCAAGGAGCGGCGCGTTTGGCGCGCTGAGGCTGGAGCACGTGGACCTGCGGTACCGGCCAGGGCTGCCGCTGGTGCTGCGCGATGTCTGCTTTGCGATCGCCCCTGGGCAGAAGGTGGGCGTTGTGGGGCGGACGGGGAGCGGGAAGTCGACGCTGCTGCTGGCCTTCCTGCGGCTGGTGGAGGTAAGCGGCGGTCGCATGCTCGTCTGCGGGCGCGACGCGCGGACGTACACGCTTCCCGCTCTGCGGCGGCTGTTTTCCATGATCCCGCAGGATCCCGTATTGTTCGATGGCACGGTGCGCAGCAACGTGGATCCGTTCGGCGACGCGACGGACGAGGAGGTGCGTGCCGCGCTGGTGTCTGTGGGGTTTGTGGGGGTGGGCGGAAGCTCAGCTACCTTTCCTACAGCTTCTGGACTGCCGAGCGCTGCATCTGACAGCATGCCTGCGCTGGACACTGTGGTGCAGGGGGGCGGGTCGAACTTCAGCGTTGGGCAGCGCCAGCTGCTGTGCTTAGCGCGCGCGCTGCTGAAGAAGGGGAGTGCATTCATCCTGATGGACGAGGCGACGGCGAACGTGGACGCACAGCTGGACCAGACGGTGCAGCGTATTGTGGCGGAGCAGTTTGGCGCGTACACGGTGGTGACGATCGCGCACCGTCTGCACACGGTGGCGGCGTACGACGTAGTGCTTGTGATGGCGCGCGGGCGTGTGGTGGAGATGGGGCAGCCGCGGGCGCTGCTGGAGCGTCGCGACTCCGTGTTCTACGGTATGGTAGCGCAGAGCGCAGCGGTGGCTGATTCCCGTGAGGGCTTGTCGCGGGAGGATGCCGAGAGTGCGGCCAGTGGTGCGCGTTCGCGTGGCGTCAGTGACGGGGGCGCTGAGCTGGGGGCGGCTGGGCGCGAACACCGCGTGGAGGCCGCGGTCGCGTCGCTCCTGCGTCAGTGCAAGTGACCGGGATCGCACTCACGCGTCGCTGTATGCCTGCGTCTCTCGCTCTCTCTGGTGAGGGCTCGCAGCACCTCACTTGCCGCTTCGCTCGATCTATTCTTTCTCTTTTTGTGCTGCACTGGGCCGCACCACCACCGCATTGGTGACCGCACACCGCTCCCCACCGAGGCTTCCGCGGCTGCTGCACGTGCGTGGACGGCATTCGCGCAGCTCAGCTGCCGGCATTGGCTAGATTGACCGTCTTTTTTGCACAGCCACCCGTTGCAGCGGCGCGACACGGCTGGACCAGTTTTGTGTGCCCTTTGGCTGCTTAGCCCGCTGCGATAGGCGTGCCGGCCGGCAGCTGTCGTCTCCCCTGTCACCGGAGTGCTCGCTCTCCCCGCACCACATGCATTGCGAGCCGCCAGAGCTCTACACGATCATCTTAGTCTTTTGGCACACTTGCTGCGCCCTTTCGGCGCACGCTGCATGTTGCCCATCCGAGCCTGGGCATCTTGGAGATT

>BPK190/0 clone 3|LinJ31_V3.1460|500 bp UPS + CDS + 500 bp DWS|Pentamidine resistance protein 1

ACTGCGCATTTCTGCAAGTCTTCATTTCAGCGAGGCAAACCTCCCGACCGGTAGTGTGTCCCTTTTTTCCCGCTGCTCTTATTGCAATGCCGTGTTGTTCGATTGCCTCTTGACGGAGGGGTGGAGAAGCTTGAAAGGAGAGAGACGATGCGGCGCACGAGGACTCGCTAGCGATCCGATCGGATGAAGTGATTGCCATGCTGCTTTTTTTTTTGGCCGCGTCGAGAGGTGCTGCGAGACGGGGGTGACATCGCGGCAAACATGTTCATCGGTTCCACGTTGTTTCACTCGCTGAAGGCAATGACTGGCATCAAGGCTTGCCGTTGATGACGTCATGTGAGAGAGCAAGGACTCTTCGGATTCTCTACCTTTCGCTCAAGCAGGCTTGCTTGCCATGCGTGCTGGTGGCTTCTCATGTCAGTGCCAGGTTTTACATCTGGTGCGTGTGACACGTGTGCGTGCATTTCTTCAGCTTGACTTTTTCTCATACCTCTTCGTCTATGAGCAGTCAACGACCGGAGATGCCAGAAGGAGCGGCGAGTGATAGCGTCTACTCGTTCGGCGAGGTTAACCGACGGTTGTGGCTTCTGCGTTCTAATTCGTTACCGAAGGGTAGCGCTTGTGATGATGCGTTGGTGGACTGGGCTGCGATACGCGGGTTGCTGGAGGAGGCTCCGGAAGAGCGGAGCGGCGTTTTATCGCGTTACCTGCTGAGGTGGCTGAACCCATATGTTATGCTTGCGTGGAGGGAGCGGCTGGAGGAGGCGTACATGCCTCCACCACAGCGGGCGCACCGCGCCGTGTGCTGTGGCGCTCTGCTAAGCCGCGCGTTTCGCGAGGAGGAGGTTCGCGGGGGCCGCGACGCGTGGCGTGCGCGCGTTGAAGCTGCGCTTCCAGTGCAGTGTGGAGCCTGCGATGACCCCTCGGGGGAAAGTGAAGTGATTGGCGGCGATTATAACGGGCGATGGGGTGGCGCGCGCGTGGCGGCTATCAACGAGCTGTCACGTGTGAGGTTCCGTGGTGAGGAGGGTGTCCATGGACGGCTGCGGTGGGTGGGGTACGTTCGATCGAGCGACACGCCGCACACGCTTTTGTGCGGTGTGGAGTGGGACGCTGACAGCGCGTTGCCGCCGTATCGTGCGCGTGTGGCTGGTGATGTGGCTGAGGAAGTAGTGCACGACGGGTGCGTGCAGGGCGAGCGTCTGTTTTACCAGGTTCAGGATGGGCGGGCGCGGTGCACGTGCGAGTACGTGCAGGATCTGGTGCCGGTTTCGACGATTGGAATCGCGGATACTGGGTGCCCGCGTATGCCTCACGCTCCGTCGCTGCTTTGGGCGCTACTCCGCACGTTTCGCTCCGATCTAATGGCGATACTGCCACCTTCGATTGCCGGTATGATATGTGAAGTGTCGACACCGTGGTTGCTGCAGCAGTTTGTGCTGTTCCTGCAGTCGAGCAAGGAGCGGGCGGGAGCGAGAAAAGGGCTGCTCTTCTTCTCGATTCTTGTGATCGTGAAGTTGGTGCAACCAGCGTTGATGAACAAGGAGATGCACCGCAGTCGGCGTGTTTCGAGCCTTTTCCGCACCTCGACACTCGCCCTGATTTTCGAAAAGTGTCTGACGATATCGCCGGATGCGCTGTCGAGACCGGACATGAACACTGGTCGTGTGCTAGCGATGGCGAGCTCTGACGTTGAGAACATCAAGGAATTTCCCACGCGGGTGATGTTCCTGTGGATGGCGCCGACTATGCTGACGCTGTACGTTGCCTACCTCTTCGTTCTCGTGGGGCCGAGTGCCCTGGCTGCTGTCCTGGTTTTTGCCGCTTTACTGCCTGTCCAAGGGGGGTTAACGAATGCTATGGGCGGCGCACAAGAGAACCTGTCGAGCTGCACGGATCAGCGCTTGCGTCGCACGAATGAACTGCTCTCCGGCATTCGTGTCGTGAAGATGATGGGATGGGAGTCGAAGTTTGTCTCGGCGATCGAAGATAACGCACGGGCCGATGAGTTGCGATTCCGACGAAGACTGCAGATGCGTCAAGTCGGGCTGTGGGCATGCGTGTTCTCTACTCCGACGTTCATGATCGCCGCTGTTCTCACTACCTACACCCTCAGCGGACACAAGCTTGACGCATCCGTGGTGTTCCCTCTCATCGCAGTTGTCAGTGCTATAACCTTCCCTGTAATGATGCTTCCGGAGGCCTTTACGTCGCTGGCGAAGTTCATTGTGTCGACGGGGCGCGTCACGCAGTTCTTGGAGTGCGACGACTCGCACATCATCGTCGAGCACGCGGAGCGCATGCTCAACAGCACAGGGAGTGGCGGCGCTGCTTCCGGGGACGCACCGCTGCCGAGTGCCGCTTCGGCCGACCTTGCAAGGGTGCTTGTGTCAGTGCCCGCCGCGCTTCCCGTGTACGAGCCGCGACACGTGGGGCTGCGCCGCGTGGCGCAGAGGATACTGTGCGCGGTGCTGCGCCGCCGTGTGCCGGTGGAGCTTCAGTGGCGGAGGGCGGCTGCTTCCCCTGCCATGGGTGAAGGCGGTAGGAAGCCTGCGCGCGTTGCTGTGGGGAACGATGATGGCGGTGTGGCTGTTGCGGGTGGTGAGAACGGGCGCGGCAGTGATAGTGGCGTGGCTCTGTACGCGATGGTGGACAGGGCTCTCCTGCGCGATGTGCGCGTGTCGTTCCCGCGCGCGCAGTTGACGGTTGTTGTTGGCGCGACGGGGAGCGGGAAGTCTGTGCTGCTGGCGACGCTTTTGGGTGCGTTCCGGTTCGAGGGGCATGTGAGCGTAGCGAAGTCGGTAGCTTACGTGCCGCAGCAGCCGTGGATCATGCAGGAAACGTTAGAGGCGAACATCACGTTCTTCGAGGGCGATCGGCGCGGCGCTGCTGGTGGCGCGGCGGTCGATAGCAATAGCGGCTCTGCGCAGGCGTTCTCGCCTCTGAGGTGCGCTGAGCAAGCTGCTGGCGCTGCTGCTGTGCCACGGTGCTTCAGCTGCGGCCGTGACGCGGCGAGCGAGCGTCTTGCGCGCGCTGTGCGGAGCTGCCAGCTGAATGCTGATGTGGCACTGATGGCGCGCGGGCTGGGGACAGAGATCGGCGAGCGTGGCATCAACCTGAGCGGTGGGCAGAAGGCGCGCGTGAGCCTTGCGCGCGCCGTGTATGCGGACCGCGACGTGTACCTGCTGGACGATCCGCTGTCGGCGCTGGACGCGCACGTTGGGCGGCGCGTGATGGACGAGGTTGTGCTGCGCGCGTTGTCGGGCAAGACGCGCGTGCTAGCGACGCACCAGCTGCAGGTGCTGCCGCACGCGGACCAGGTCGTGGTGATGCGCGAGGGCTGCGCCGTGTTTGCGGGCAGCTACGCTGCTTACGCTGCGTCGGAGTGGAAGGCGTACGTGGAGAGCGAGGAGGCCGCCGCCGCGAGCAAGAAGAGCGGGTGTGGCGGTGGTGATACTGTGGCGGAGTGCACCGAGGCCAGTTCTGCGTGGTCGTCTGCGGTGAACGTGGAGTCGTGTGTGGGCACGCGTGAGGAGCGTGACCACGCTCGGTGTGGCGGTGCCGATGGCCGTGCCAGCAAAGACGGCGGTGAGTGGGGGGAGAACGATGGCAGCCCTTTGCCGAGAATGCACTGGCAGGGCTCATGGGCCAGCGAGAACTCAGAGGCGGAAGGCTGCAACAATAGGGACTCTACGGTATGTAAAGGTGTTAGGCTCGCAAGCAGGTCTAAGGCCGCGCCGCGCACGGCGAAGGGCTCATCTGGGCTGGCTACGGGTGAGGATGCAGCGGACGGCGGCGACCTGATGACTGCGGAGGAGAAGGAGACAGGGCACACACCGTGGAGTGTGTACCGTGCCTACTTTGAGGCGGGCGGGGGCGTCCCGGTCGCAATTCAAATTGTGCTGCGCTACTTCGTTAGCGAAGCGCTCTCGACAGGCTCCAGCGTTTGGCTGACGCTGTGGTCAGTGAACTACTTTGGTTCTTCTCTCTCTACCAACGAACAGCTTGGCGTGTATCTTGGACTCGTCTTTGTGGTTGCTATCACAGTGTCTGCCAACGACCTGTTTATTTTTCAGTTCGCTCGCCGCGCTGCCTGCCGCTTGCACGCCATACTCTTGTACACGGTGAGCTCAGCCACGCTGACGTTTTTTGACCGCACACCACTGGGTCGCATTGTCAACCGGTTCAGCAGGGACGTGCACGTCCTTGACGATGAGCTGCCGGCGAACGTGATTCCCTTTTTGGGCATTACGGGATATGTCATGACGTCTTTAGCAGTGACGCTATACACTTCACCGCTTAGTGTGGTCGTGGTGCTGCTAGCGGTTTACGCTTTTGTGCGCCTGCTCAAGTTCTACGCGACAGTGGTGCGTGAGGTGCGACGGCGCAGCAGTGTGGGGCAGTCTCCGTTGCTCTCGCTGCTGGAGGAGGTGGTGCACGGGCGTGCGACGATCGCTGCATACGACAAGTCGCACGTGCTATTCGCGGAGGCGCTCCTGCGGCTGGACCTTGTGTACAGCTGCACCTACGTGGAGAAGGTTATGACGTTGTGGCTCGCTATCCGAATCGAGTACATTGCGTCATTGGCCGTTATTGCTGTGGGGCTGATCGGCGTTGTGGAGAAGCTGGTAGAGGCGTCGCCGGCGCTGCCGGAGGCGCGCGTGGGCCTGATCTCGCTGAGCCTCACCATGTGCCTCGATCTCAGCTGGTCGCTATCTGCACTCCTAGACCTTGCTGCCGCTGTCGAGGCGAGCATGAACAGTGTGCAGCGCGTTTGCCACTACATTGATCACGTGCCGCAGGAAGCGGCGCTCCTGGAGCCGCGGGACGCGTATGTGGCGCGCGCTGTGGCAGCGAGTGGCGGCAGCCGCAGGGGAGGAGGCGAGAGCTCCAGAAGTGCGAGCGACATCGTTGTGGCTGCCGGCGGTGATGACGACGAGGCCGCGCCTGCGCGACAGGGTGTTGCGGCAAGGAGCGGCGCGTTTGGCGCGCTGAGGCTGGAGCACGTGGACCTGCGGTACCGGCCAGGGCTGCCGCTGGTGCTGCGCGATGTCTGCTTTGCGATCGCCCCTGGGCAGAAGGTGGGCGTTGTGGGGCGGACGGGGAGCGGGAAGTCGACGCTGCTGCTGGCCTTCCTGCGGCTGGTGGAGGTAAGCGGCGGTCGCATGCTCGTCTGCGGGCGCGACGCGCGGACGTACACGCTTCCCGCTCTGCGGCGGCTGTTTTCCATGATCCCGCAGGATCCCGTATTGTTCGATGGCACGGTGCGCAGCAACGTGGATCCGTTCGGCGACGCGACGGACGAGGAGGTGCGTGCCGCGCTGGTGTCTGTGGGGTTTGTGGGGGTGGGCGGAAGCTCAGCTACCTTTCCTACAGCTTCTGGACTGCCGAGCGCTGCATCTGACAGCATGCCTGCGCTGGACACTGTGGTGCAGGGGGGCGGGTCGAACTTCAGCGTTGGGCAGCGCCAGCTGCTGTGCTTAGCGCGCGCGCTGCTGAAGAAGGGGAGTGCATTCATCCTGATGGACGAGGCGACGGCGAACGTGGACGCACAGCTGGACCAGACGGTGCAGCGTATTGTGGCGGAGCAGTTTGGCGCGTACACGGTGGTGACGATCGCGCACCGTCTGCACACGGTGGCGGCGTACGACGTAGTGCTTGTGATGGCGCGCGGGCGTGTGGTGGAGATGGGGCAGCCGCGGGCGCTGCTGGAGCGTCGCGACTCCGTGTTCTACGGTATGGTAGCGCAGAGCGCAGCGGTGGCTGATTCCCGTGAGGGCTTGTCGCGGGAGGATGCCGAGAGTGCGGCCAGTGGTGCGCGTTCGCGTGGCGTCAGTGACGGGGGCGCTGAGCTGGGGGCGGCTGGGCGCGAACACCGCGTGGAGGCCGCGGTCGCGTCGCTCCTGCGTCAGTGCAAGTGACCGGGATCGCACTCACGCGTCGCTGTATGCCTGCGTCTCTCGCTCTCTCTGGTGAGGGCTCGCAGCACCTCACTTGCCGCTTCGCTCGATCTATTCTTTCTCTTTTTGTGCTGCACTGGGCCGCACCACCACCGCATTGGTGACCGCACACCGCTCCCCACCGAGGCTTCCGCGGCTGCTGCACGTGCGTGGACGGCATTCGCGCAGCTCAGCTGCCGGCATTGGCTAGATTGACCGTCTTTTTTGCACAGCCACCCGTTGCAGCGGCGCGACACGGCTGGACCAGTTTTGTGTGCCCTTTGGCTGCTTAGCCCGCTGCGATAGGCGTGCCGGCCGGCAGCTGTCGTCTCCCCTGTCACCGGAGTGCTCGCTCTCCCCGCACCACATGCATTGCGAGCCGCCAGAGCTCTACACGATCATCTTAGTCTTTTGGCACACTTGCTGCGCCCTTTCGGCGCACGCTGCATGTTGCCCATCCGAGCCTGGGCATCTTGGAGATT

>BPK206/0 clone 10|LinJ31_V3.1460|500 bp UPS + CDS + 500 bp DWS|Pentamidine resistance protein 1

ACTGCGCATTTCTGCAAGTCTTCATTTCAGCGAGGCAAACCTCCCGACCGGTAGTGTGTCCCTTTTTTCCCGCTGCTCTTATTGCAATGCCGTGTTGTTCGATTGCCTCTTGACGGAGGGGTGGAGAAGCTTGAAAGGAGAGAGACGATGCGGCGCACGAGGACTCGCTAGCGATCCGATCGGATGAAGTGATTGCCATGCTGCTTTTTTTTTTGGCCGCGTCGAGAGGTGCTGCGAGACGGGGGTGACATCGCGGCAAACATGTTCATCGGTTCCACGTTGTTTCACTCGCTGAAGGCAATGACTGGCATCAAGGCTTGCCGTTGATGACGTCATGTGAGAGAGCAAGGACTCTTCGGATTCTCTACCTTTCGCTCAAGCAGGCTTGCTTGCCATGCGTGCTGGTGGCTTCTCATGTCAGTGCCAGGTTTTACATCTGGTGCGTGTGACACGTGTGCGTGCATTTCTTCAGCTTGACTTTTTCTCATACCTCTTCGTCTATGAGCAGTCAACGACCGGAGATGCCAGAAGGAGCGGCGAGTGATAGCGTCTACTCGTTCGGCGAGGTTAACCGACGGTTGTGGCTTCTGCGTTCTAATTCGTTACCGAAGGGTAGCGCTTGTGATGATGCGTTGGTGGACTGGGCTGCGATACGCGGGTTGCTGGAGGAGGCTCCGGAAGAGCGGAGCGGCGTTTTATCGCGTTACCTGCTGAGGTGGCTGAACCCATATGTTATGCTTGCGTGGAGGGAGCGGCTGGAGGAGGCGTACATGCCTCCACCACAGCGGGCGCACCGCGCCGTGTGCTGTGGCGCTCTGCTAAGCCGCGCGTTTCGCGAGGAGGAGGTTCGCGGGGGCCGCGACGCGTGGCGTGCGCGCGTTGAAGCTGCGCTTCCAGTGCAGTGTGGAGCCTGCGATGACCCCTCGGGGGAAAGTGAAGTGATTGGCGGCGATTATAACGGGCGATGGGGTGGCGCGCGCGTGGCGGCTATCAACGAGCTGTCACGTGTGAGGTTCCGTGGTGAGGAGGGTGTCCATGGACGGCTGCGGTGGGTGGGGTACGTTCGATCGAGCGACACGCCGCACACGCTTTTGTGCGGTGTGGAGTGGGACGCTGACAGCGCGTTGCCGCCGTATCGTGCGCGTGTGGCTGGTGATGTGGCTGAGGAAGTAGTGCACGACGGGTGCGTGCAGGGCGAGCGTCTGTTTTACCAGGTTCAGGATGGGCGGGCGCGGTGCACGTGCGAGTACGTGCAGGATCTGGTGCCGGTTTCGACGATTGGAATCGCGGATACTGGGTGCCCGCGTATGCCTCACGCTCCGTCGCTGCTTTGGGCGCTACTCCGCACGTTTCGCTCCGATCTAATGGCGATACTGCCACCTTCGATTGCCGGTATGATATGTGAAGTGTCGACACCGTGGTTGCTGCAGCAGTTTGTGCTGTTCCTGCAGTCGAGCAAGGAGCGGGCGGGAGCGAGAAAAGGGCTGCTCTTCTTCTCGATTCTTGTGATCGTGAAGTTGGTGCAACCAGCGTTGATGAACAAGGAGATGCACCGCAGTCGGCGTGTTTCGAGCCTTTTCCGCACCTCGACACTCGCCCTGATTTTCGAAAAGTGTCTGACGATATCGCCGGATGCGCTGTCGAGACCGGACATGAACACTGGTCGTGTGCTAGCGATGGCGAGCTCTGACGTTGAGAACATCAAGGAATTTCCCACGCGGGTGATGTTCCTGTGGATGGCGCCGACTATGCTGACGCTGTACGTTGCCTACCTCTTCGTTCTCGTGGGGCCGAGTGCCCTGGCTGCTGTCCTGGTTTTTGCCGCTTTACTGCCTGTCCAAGGGGGGTTAACGAATGCTATGGGCGGCGCACAAGAGAACCTGTCGAGCTGCACGGATCAGCGCTTGCGTCGCACGAATGAACTGCTCTCCGGCATTCGTGTCGTGAAGATGATGGGATGGGAGTCGAAGTTTGTCTCGGCGATCGAAGATAACGCACGGGCCGATGAGTTGCGATTCCGACGAAGACTGCAGATGCGTCAAGTCGGGCTGTGGGCATGCGTGTTCTCTACTCCGACGTTCATGATCGCCGCTGTTCTCACTACCTACACCCTCAGCGGACACAAGCTTGACGCATCCGTGGTGTTCCCTCTCATCGCAGTTGTCAGTGCTATAACCTTCCCTGTAATGATGCTTCCGGAGGCCTTTACGTCGCTGGCGAAGTTCATTGTGTCGACGGGGCGCGTCACGCAGTTCTTGGAGTGCGACGACTCGCACATCATCGTCGAGCACGCGGAGCGCATGCTCAACAGCACAGGGAGTGGCGGCGCTGCTTCCGGGGACGCACCGCTGCCGAGTGCCGCTTCGGCCGACCTTGCAAGGGTGCTTGTGTCAGTGCCCGCCGCGCTTCCCGTGTACGAGCCGCGACACGTGGGGCTGCGCCGCGTGGCGCAGAGGATACTGTGCGCGGTGCTGCGCCGCCGTGTGCCGGTGGAGCTTCAGTGGCGGAGGGCGGCTGCTTCCCCTGCCATGGGTGAAGGCGGTAGGAAGCCTGCGCGCGTTGCTGTGGGGAACGATGATGGCGGTGTGGCTGTTGCGGGTGGTGAGAACGGGCGCGGCAGTGATAGTGGCGTGGCTCTGTACGCGATGGTGGACAGGGCTCTCCTGCGCGATGTGCGCGTGTCGTTCCCGCGCGCGCAGTTGACGGTTGTTGTTGGCGCGACGGGGAGCGGGAAGTCTGTGCTGCTGGCGACGCTTTTGGGTGCGTTCCGGTTCGAGGGGCATGTGAGCGTAGCGAAGTCGGTAGCTTACGTGCCGCAGCAGCCGTGGATCATGCAGGAAACGTTAGAGGCGAACATCACGTTCTTCGAGGGCGATCGGCGCGGCGCTGCTGGTGGCGCGGCGGTCGATAGCAATAGCGGCTCTGCGCAGGCGTTCTCGCCTCTGAGGTGCGCTGAGCAAGCTGCTGGCGCTGCTGCTGTGCCACGGTGCTTCAGCTGCGGCCGTGACGCGGCGAGCGAGCGTCTTGCGCGCGCTGTGCGGAGCTGCCAGCTGAATGCTGATGTGGCACTGATGGCGCGCGGGCTGGGGACAGAGATCGGCGAGCGTGGCATCAACCTGAGCGGTGGGCAGAAGGCGCGCGTGAGCCTTGCGCGCGCCGTGTATGCGGACCGCGACGTGTACCTGCTGGACGATCCGCTGTCGGCGCTGGACGCGCACGTTGGGCGGCGCGTGATGGACGAGGTTGTGCTGCGCGCGTTGTCGGGCAAGACGCGCGTGCTAGCGACGCACCAGCTGCAGGTGCTGCCGCACGCGGACCAGGTCGTGGTGATGCGCGAGGGCTGCGCCGTGTTTGCGGGCAGCTACGCTGCTTACGCTGCGTCGGAGTGGAAGGCGTACGTGGAGAGCGAGGAGGCCGCCGCCGCGAGCAAGAAGAGCGGGTGTGGCGGTGGTGATACTGTGGCGGAGTGCACCGAGGCCAGTTCTGCGTGGTCGTCTGCGGTGAACGTGGAGTCGTGTGTGGGCACGCGTGAGGAGCGTGACCACGCTCGGTGTGGCGGTGCCGATGGCCGTGCCAGCAAAGACGGCGGTGAGTGGGGGGAGAACGATGGCAGCCCTTTGCCGAGAATGCACTGGCAGGGCTCATGGGCCAGCGAGAACTCAGAGGCGGAAGGCTGCAACAATAGGGACTCTACGGTATGTAAAGGTGTTAGGCTCGCAAGCAGGTCTAAGGCCGCGCCGCGCACGGCGAAGGGCTCATCTGGGCTGGCTACGGGTGAGGATGCAGCGGACGGCGGCGACCTGATGACTGCGGAGGAGAAGGAGACAGGGCACACACCGTGGAGTGTGTACCGTGCCTACTTTGAGGCGGGCGGGGGCGTCCCGGTCGCAATTCAAATTGTGCTGCGCTACTTCGTTAGCGAAGCGCTCTCGACAGGCTCCAGCGTTTGGCTGACGCTGTGGTCAGTGAACTACTTTGGTTCTTCTCTCTCTACCAACGAACAGCTTGGCGTGTATCTTGGACTCGTCTTTGTGGTTGCTATCACAGTGTCTGCCAACGACCTGTTTATTTTTCAGTTCGCTCGCCGCGCTGCCTGCCGCTTGCACGCCATACTCTTGTACACGGTGAGCTCAGCCACGCTGACGTTTTTTGACCGCACACCACTGGGTCGCATTGTCAACCGGTTCAGCAGGGACGTGCACGTCCTTGACGATGAGCTGCCGGCGAACGTGATTCCCTTTTTGGGCATTACGGGATATGTCATGACGTCTTTAGCAGTGACGCTATACACTTCACCGCTTAGTGTGGTCGTGGTGCTGCTAGCGGTTTACGCTTTTGTGCGCCTGCTCAAGTTCTACGCGACAGTGGTGCGTGAGGTGCGACGGCGCAGCAGTGTGGGGCAGTCTCCGTTGCTCTCGCTGCTGGAGGAGGTGGTGCACGGGCGTGCGACGATCGCTGCATACGACAAGTCGCACGTGCTATTCGCGGAGGCGCTCCTGCGGCTGGACCTTGTGTACAGCTGCACCTACGTGGAGAAGGTTATGACGTTGTGGCTCGCTATCCGAATCGAGTACATTGCGTCATTGGCCGTTATTGCTGTGGGGCTGATCGGCGTTGTGGAGAAGCTGGTAGAGGCGTCGCCGGCGCTGCCGGAGGCGCGCGTGGGCCTGATCTCGCTGAGCCTCACCATGTGCCTCGATCTCAGCTGGTCGCTATCTGCACTCCTAGACCTTGCTGCCGCTGTCGAGGCGAGCATGAACAGTGTGCAGCGCGTTTGCCACTACATTGATCACGTGCCGCAGGAAGCGGCGCTCCTGGAGCCGCGGGACGCGTATGTGGCGCGCGCTGTGGCAGCGAGTGGCGGCAGCCGCAGGGGAGGAGGCGAGAGCTCCAGAAGTGCGAGCGACATCGTTGTGGCTGCCGGCGGTGATGACGACGAGGCCGCGCCTGCGCGACAGGGTGTTGCGGCAAGGAGCGGCGCGTTTGGCGCGCTGAGGCTGGAGCACGTGGACCTGCGGTACCGGCCAGGGCTGCCGCTGGTGCTGCGCGATGTCTGCTTTGCGATCGCCCCTGGGCAGAAGGTGGGCGTTGTGGGGCGGACGGGGAGCGGGAAGTCGACGCTGCTGCTGGCCTTCCTGCGGCTGGTGGAGGTAAGCGGCGGTCGCATGCTCGTCTGCGGGCGCGACGCGCGGACGTACACGCTTCCCGCTCTGCGGCGGCTGTTTTCCATGATCCCGCAGGATCCCGTATTGTTCGATGGCACGGTGCGCAGCAACGTGGATCCGTTCGGCGACGCGACGGACGAGGAGGTGCGTGCCGCGCTGGTGTCTGTGGGGTTTGTGGGGGTGGGCGGAAGCTCAGCTACCTTTCCTACAGCTTCTGGACTGCCGAGCGCTGCATCTGACAGCATGCCTGCGCTGGACACTGTGGTGCAGGGGGGCGGGTCGAACTTCAGCGTTGGGCAGCGCCAGCTGCTGTGCTTAGCGCGCGCGCTGCTGAAGAAGGGGAGTGCATTCATCCTGATGGACGAGGCGACGGCGAACGTGGACGCACAGCTGGACCAGACGGTGCAGCGTATTGTGGCGGAGCAGTTTGGCGCGTACACGGTGGTGACGATCGCGCACCGTCTGCACACGGTGGCGGCGTACGACGTAGTGCTTGTGATGGCGCGCGGGCGTGTGGTGGAGATGGGGCAGCCGCGGGCGCTGCTGGAGCGTCGCGACTCCGTGTTCTACGGTATGGTAGCGCAGAGCGCAGCGGTGGCTGATTCCCGTGAGGGCTTGTCGCGGGAGGATGCCGAGAGTGCGGCCAGTGGTGCGCGTTCGCGTGGCGTCAGTGACGGGGGCGCTGAGCTGGGGGCGGCTGGGCGCGAACACCGCGTGGAGGCCGCGGTCGCGTCGCTCCTGCGTCAGTGCAAGTGACCGGGATCGCACTCACGCGTCGCTGTATGCCTGCGTCTCTCGCTCTCTCTGGTGAGGGCTCGCAGCACCTCACTTGCCGCTTCGCTCGATCTATTCTTTCTCTTTTTGTGCTGCACTGGGCCGCACCACCACCGCATTGGTGACCGCACACCGCTCCCCACCGAGGCTTCCGCGGCTGCTGCACGTGCGTGGACGGCATTCGCGCAGCTCAGCTGCCGGCATTGGCTAGATTGACCGTCTTTTTTGCACAGCCACCCGTTGCAGCGGCGCGACACGGCTGGACCAGTTTTGTGTGCCCTTTGGCTGCTTAGCCCGCTGCGATAGGCGTGCCGGCCGGCAGCTGTCGTCTCCCCTGTCACCGGAGTGCTCGCTCTCCCCGCACCACATGCATTGCGAGCCGCCAGAGCTCTACACGATCATCTTAGTCTTTTGGCACACTTGCTGCGCCCTTTCGGCGCACGCTGCATGTTGCCCATCCGAGCCTGGGCATCTTGGAGATT

>BPK275/0 clone 18|LinJ31_V3.1460|500 bp UPS + CDS + 500 bp DWS|Pentamidine resistance protein 1

ACTGCGCATTTCTGCAAGTCTTCATTTCAGCGAGGCAAACCTCCCGACCGGTAGTGTGTCCCTTTTTTCCCGCTGCTCTTATTGCAATGCCGTGTTGTTCGATTGCCTCTTGACGGAGGGGTGGAGAAGCTTGAAAGGAGAGAGACGATGCGGCGCACGAGGACTCGCTAGCGATCCGATCGGATGAAGTGATTGCCATGCTGCTTTTTTTTTTGGCCGCGTCGAGAGGTGCTGCGAGACGGGGGTGACATCGCGGCAAACATGTTCATCGGTTCCACGTTGTTTCACTCGCTGAAGGCAATGACTGGCATCAAGGCTTGCCGTTGATGACGTCATGTGAGAGAGCAAGGACTCTTCGGATTCTCTACCTTTCGCTCAAGCAGGCTTGCTTGCCATGCGTGCTGGTGGCTTCTCATGTCAGTGCCAGGTTTTACATCTGGTGCGTGTGACACGTGTGCGTGCATTTCTTCAGCTTGACTTTTTCTCATACCTCTTCGTCTATGAGCAGTCAACGACCGGAGATGCCAGAAGGAGCGGCGAGTGATAGCGTCTACTCGTTCGGCGAGGTTAACCGACGGTTGTGGCTTCTGCGTTCTAATTCGTTACCGAAGGGTAGCGCTTGTGATGATGCGTTGGTGGACTGGGCTGCGATACGCGGGTTGCTGGAGGAGGCTCCGGAAGAGCGGAGCGGCGTTTTATCGCGTTACCTGCTGAGGTGGCTGAACCCATATGTTATGCTTGCGTGGAGGGAGCGGCTGGAGGAGGCGTACATGCCTCCACCACAGCGGGCGCACCGCGCCGTGTGCTGTGGCGCTCTGCTAAGCCGCGCGTTTCGCGAGGAGGAGGTTCGCGGGGGCCGCGACGCGTGGCGTGCGCGCGTTGAAGCTGCGCTTCCAGTGCAGTGTGGAGCCTGCGATGACCCCTCGGGGGAAAGTGAAGTGATTGGCGGCGATTATAACGGGCGATGGGGTGGCGCGCGCGTGGCGGCTATCAACGAGCTGTCACGTGTGAGGTTCCGTGGTGAGGAGGGTGTCCATGGACGGCTGCGGTGGGTGGGGTACGTTCGATCGAGCGACACGCCGCACACGCTTTTGTGCGGTGTGGAGTGGGACGCTGACAGCGCGTTGCCGCCGTATCGTGCGCGTGTGGCTGGTGATGTGGCTGAGGAAGTAGTGCACGACGGGTGCGTGCAGGGCGAGCGTCTGTTTTACCAGGTTCAGGATGGGCGGGCGCGGTGCACGTGCGAGTACGTGCAGGATCTGGTGCCGGTTTCGACGATTGGAATCGCGGATACTGGGTGCCCGCGTATGCCTCACGCTCCGTCGCTGCTTTGGGCGCTACTCCGCACGTTTCGCTCCGATCTAATGGCGATACTGCCACCTTCGATTGCCGGTATGATATGTGAAGTGTCGACACCGTGGTTGCTGCAGCAGTTTGTGCTGTTCCTGCAGTCGAGCAAGGAGCGGGCGGGAGCGAGAAAAGGGCTGCTCTTCTTCTCGATTCTTGTGATCGTGAAGTTGGTGCAACCAGCGTTGATGAACAAGGAGATGCACCGCAGTCGGCGTGTTTCGAGCCTTTTCCGCACCTCGACACTCGCCCTGATTTTCGAAAAGTGTCTGACGATATCGCCGGATGCGCTGTCGAGACCGGACATGAACACTGGTCGTGTGCTAGCGATGGCGAGCTCTGACGTTGAGAACATCAAGGAATTTCCCACGCGGGTGATGTTCCTGTGGATGGCGCCGACTATGCTGACGCTGTACGTTGCCTACCTCTTCGTTCTCGTGGGGCCGAGTGCCCTGGCTGCTGTCCTGGTTTTTGCCGCTTTACTGCCTGTCCAAGGGGGGTTAACGAATGCTATGGGCGGCGCACAAGAGAACCTGTCGAGCTGCACGGATCAGCGCTTGCGTCGCACGAATGAACTGCTCTCCGGCATTCGTGTCGTGAAGATGATGGGATGGGAGTCGAAGTTTGTCTCGGCGATCGAAGATAACGCACGGGCCGATGAGTTGCGATTCCGACGAAGACTGCAGATGCGTCAAGTCGGGCTGTGGGCATGCGTGTTCTCTACTCCGACGTTCATGATCGCCGCTGTTCTCACTACCTACACCCTCAGCGGACACAAGCTTGACGCATCCGTGGTGTTCCCTCTCATCGCAGTTGTCAGTGCTATAACCTTCCCTGTAATGATGCTTCCGGAGGCCTTTACGTCGCTGGCGAAGTTCATTGTGTCGACGGGGCGCGTCACGCAGTTCTTGGAGTGCGACGACTCGCACATCATCGTCGAGCACGCGGAGCGCATGCTCAACAGCACAGGGAGTGGCGGCGCTGCTTCCGGGGACGCACCGCTGCCGAGTGCCGCTTCGGCCGACCTTGCAAGGGTGCTTGTGTCAGTGCCCGCCGCGCTTCCCGTGTACGAGCCGCGACACGTGGGGCTGCGCCGCGTGGCGCAGAGGATACTGTGCGCGGTGCTGCGCCGCCGTGTGCCGGTGGAGCTTCAGTGGCGGAGGGCGGCTGCTTCCCCTGCCATGGGTGAAGGCGGTAGGAAGCCTGCGCGCGTTGCTGTGGGGAACGATGATGGCGGTGTGGCTGTTGCGGGTGGTGAGAACGGGCGCGGCAGTGATAGTGGCGTGGCTCTGTACGCGATGGTGGACAGGGCTCTCCTGCGCGATGTGCGCGTGTCGTTCCCGCGCGCGCAGTTGACGGTTGTTGTTGGCGCGACGGGGAGCGGGAAGTCTGTGCTGCTGGCGACGCTTTTGGGTGCGTTCCGGTTCGAGGGGCATGTGAGCGTAGCGAAGTCGGTAGCTTACGTGCCGCAGCAGCCGTGGATCATGCAGGAAACGTTAGAGGCGAACATCACGTTCTTCGAGGGCGATCGGCGCGGCGCTGCTGGTGGCGCGGCGGTCGATAGCAATAGCGGCTCTGCGCAGGCGTTCTCGCCTCTGAGGTGCGCTGAGCAAGCTGCTGGCGCTGCTGCTGTGCCACGGTGCTTCAGCTGCGGCCGTGACGCGGCGAGCGAGCGTCTTGCGCGCGCTGTGCGGAGCTGCCAGCTGAATGCTGATGTGGCACTGATGGCGCGCGGGCTGGGGACAGAGATCGGCGAGCGTGGCATCAACCTGAGCGGTGGGCAGAAGGCGCGCGTGAGCCTTGCGCGCGCCGTGTATGCGGACCGCGACGTGTACCTGCTGGACGATCCGCTGTCGGCGCTGGACGCGCACGTTGGGCGGCGCGTGATGGACGAGGTTGTGCTGCGCGCGTTGTCGGGCAAGACGCGCGTGCTAGCGACGCACCAGCTGCAGGTGCTGCCGCACGCGGACCAGGTCGTGGTGATGCGCGAGGGCTGCGCCGTGTTTGCGGGCAGCTACGCTGCTTACGCTGCGTCGGAGTGGAAGGCGTACGTGGAGAGCGAGGAGGCCGCCGCCGCGAGCAAGAAGAGCGGGTGTGGCGGTGGTGATACTGTGGCGGAGTGCACCGAGGCCAGTTCTGCGTGGTCGTCTGCGGTGAACGTGGAGTCGTGTGTGGGCACGCGTGAGGAGCGTGACCACGCTCGGTGTGGCGGTGCCGATGGCCGTGCCAGCAAAGACGGCGGTGAGTGGGGGGAGAACGATGGCAGCCCTTTGCCGAGAATGCACTGGCAGGGCTCATGGGCCAGCGAGAACTCAGAGGCGGAAGGCTGCAACAATAGGGACTCTACGGTATGTAAAGGTGTTAGGCTCGCAAGCAGGTCTAAGGCCGCGCCGCGCACGGCGAAGGGCTCATCTGGGCTGGCTACGGGTGAGGATGCAGCGGACGGCGGCGACCTGATGACTGCGGAGGAGAAGGAGACAGGGCACACACCGTGGAGTGTGTACCGTGCCTACTTTGAGGCGGGCGGGGGCGTCCCGGTCGCAATTCAAATTGTGCTGCGCTACTTCGTTAGCGAAGCGCTCTCGACAGGCTCCAGCGTTTGGCTGACGCTGTGGTCAGTGAACTACTTTGGTTCTTCTCTCTCTACCAACGAACAGCTTGGCGTGTATCTTGGACTCGTCTTTGTGGTTGCTATCACAGTGTCTGCCAACGACCTGTTTATTTTTCAGTTCGCTCGCCGCGCTGCCTGCCGCTTGCACGCCATACTCTTGTACACGGTGAGCTCAGCCACGCTGACGTTTTTTGACCGCACACCACTGGGTCGCATTGTCAACCGGTTCAGCAGGGACGTGCACGTCCTTGACGATGAGCTGCCGGCGAACGTGATTCCCTTTTTGGGCATTACGGGATATGTCATGACGTCTTTAGCAGTGACGCTATACACTTCACCGCTTAGTGTGGTCGTGGTGCTGCTAGCGGTTTACGCTTTTGTGCGCCTGCTCAAGTTCTACGCGACAGTGGTGCGTGAGGTGCGACGGCGCAGCAGTGTGGGGCAGTCTCCGTTGCTCTCGCTGCTGGAGGAGGTGGTGCACGGGCGTGCGACGATCGCTGCATACGACAAGTCGCACGTGCTATTCGCGGAGGCGCTCCTGCGGCTGGACCTTGTGTACAGCTGCACCTACGTGGAGAAGGTTATGACGTTGTGGCTCGCTATCCGAATCGAGTACATTGCGTCATTGGCCGTTATTGCTGTGGGGCTGATCGGCGTTGTGGAGAAGCTGGTAGAGGCGTCGCCGGCGCTGCCGGAGGCGCGCGTGGGCCTGATCTCGCTGAGCCTCACCATGTGCCTCGATCTCAGCTGGTCGCTATCTGCACTCCTAGACCTTGCTGCCGCTGTCGAGGCGAGCATGAACAGTGTGCAGCGCGTTTGCCACTACATTGATCACGTGCCGCAGGAAGCGGCGCTCCTGGAGCCGCGGGACGCGTATGTGGCGCGCGCTGTGGCAGCGAGTGGCGGCAGCCGCAGGGGAGGAGGCGAGAGCTCCAGAAGTGCGAGCGACATCGTTGTGGCTGCCGGCGGTGATGACGACGAGGCCGCGCCTGCGCGACAGGGTGTTGCGGCAAGGAGCGGCGCGTTTGGCGCGCTGAGGCTGGAGCACGTGGACCTGCGGTACCGGCCAGGGCTGCCGCTGGTGCTGCGCGATGTCTGCTTTGCGATCGCCCCTGGGCAGAAGGTGGGCGTTGTGGGGCGGACGGGGAGCGGGAAGTCGACGCTGCTGCTGGCCTTCCTGCGGCTGGTGGAGGTAAGCGGCGGTCGCATGCTCGTCTGCGGGCGCGACGCGCGGACGTACACGCTTCCCGCTCTGCGGCGGCTGTTTTCCATGATCCCGCAGGATCCCGTATTGTTCGATGGCACGGTGCGCAGCAACGTGGATCCGTTCGGCGACGCGACGGACGAGGAGGTGCGTGCCGCGCTGGTGTCTGTGGGGTTTGTGGGGGTGGGCGGAAGCTCAGCTACCTTTCCTACAGCTTCTGGACTGCCGAGCGCTGCATCTGACAGCATGCCTGCGCTGGACACTGTGGTGCAGGGGGGCGGGTCGAACTTCAGCGTTGGGCAGCGCCAGCTGCTGTGCTTAGCGCGCGCGCTGCTGAAGAAGGGGAGTGCATTCATCCTGATGGACGAGGCGACGGCGAACGTGGACGCACAGCTGGACCAGACGGTGCAGCGTATTGTGGCGGAGCAGTTTGGCGCGTACACGGTGGTGACGATCGCGCACCGTCTGCACACGGTGGCGGCGTACGACGTAGTGCTTGTGATGGCGCGCGGGCGTGTGGTGGAGATGGGGCAGCCGCGGGCGCTGCTGGAGCGTCGCGACTCCGTGTTCTACGGTATGGTAGCGCAGAGCGCAGCGGTGGCTGATTCCCGTGAGGGCTTGTCGCGGGAGGATGCCGAGAGTGCGGCCAGTGGTGCGCGTTCGCGTGGCGTCAGTGACGGGGGCGCTGAGCTGGGGGCGGCTGGGCGCGAACACCGCGTGGAGGCCGCGGTCGCGTCGCTCCTGCGTCAGTGCAAGTGACCGGGATCGCACTCACGCGTCGCTGTATGCCTGCGTCTCTCGCTCTCTCTGGTGAGGGCTCGCAGCACCTCACTTGCCGCTTCGCTCGATCTATTCTTTCTCTTTTTGTGCTGCACTGGGCCGCACCACCACCGCATTGGTGACCGCACACCGCTCCCCACCGAGGCTTCCGCGGCTGCTGCACGTGCGTGGACGGCATTCGCGCAGCTCAGCTGCCGGCATTGGCTAGATTGACCGTCTTTTTTGCACAGCCACCCGTTGCAGCGGCGCGACACGGCTGGACCAGTTTTGTGTGCCCTTTGGCTGCTTAGCCCGCTGCGATAGGCGTGCCGGCCGGCAGCTGTCGTCTCCCCTGTCACCGGAGTGCTCGCTCTCCCCGCACCACATGCATTGCGAGCCGCCAGAGCTCTACACGATCATCTTAGTCTTTTGGCACACTTGCTGCGCCCTTTCGGCGCACGCTGCATGTTGCCCATCCGAGCCTGGGCATCTTGGAGATT

>BPK282/0 clone 4|LinJ31_V3.1460|500 bp UPS + CDS + 500 bp DWS|Pentamidine resistance protein 1

ACTGCGCATTTCTGCAAGTCTTCATTTCAGCGAGGCAAACCTCCCGACCGGTAGTGTGTCCCTTTTTTCCCGCTGCTCTTATTGCAATGCCGTGTTGTTCGATTGCCTCTTGACGGAGGGGTGGAGAAGCTTGAAAGGAGAGAGACGATGCGGCGCACGAGGACTCGCTAGCGATCCGATCGGATGAAGTGATTGCCATGCTGCTTTTTTTTTTGGCCGCGTCGAGAGGTGCTGCGAGACGGGGGTGACATCGCGGCAAACATGTTCATCGGTTCCACGTTGTTTCACTCGCTGAAGGCAATGACTGGCATCAAGGCTTGCCGTTGATGACGTCATGTGAGAGAGCAAGGACTCTTCGGATTCTCTACCTTTCGCTCAAGCAGGCTTGCTTGCCATGCGTGCTGGTGGCTTCTCATGTCAGTGCCAGGTTTTACATCTGGTGCGTGTGACACGTGTGCGTGCATTTCTTCAGCTTGACTTTTTCTCATACCTCTTCGTCTATGAGCAGTCAACGACCGGAGATGCCAGAAGGAGCGGCGAGTGATAGCGTCTACTCGTTCGGCGAGGTTAACCGACGGTTGTGGCTTCTGCGTTCTAATTCGTTACCGAAGGGTAGCGCTTGTGATGATGCGTTGGTGGACTGGGCTGCGATACGCGGGTTGCTGGAGGAGGCTCCGGAAGAGCGGAGCGGCGTTTTATCGCGTTACCTGCTGAGGTGGCTGAACCCATATGTTATGCTTGCGTGGAGGGAGCGGCTGGAGGAGGCGTACATGCCTCCACCACAGCGGGCGCACCGCGCCGTGTGCTGTGGCGCTCTGCTAAGCCGCGCGTTTCGCGAGGAGGAGGTTCGCGGGGGCCGCGACGCGTGGCGTGCGCGCGTTGAAGCTGCGCTTCCAGTGCAGTGTGGAGCCTGCGATGACCCCTCGGGGGAAAGTGAAGTGATTGGCGGCGATTATAACGGGCGATGGGGTGGCGCGCGCGTGGCGGCTATCAACGAGCTGTCACGTGTGAGGTTCCGTGGTGAGGAGGGTGTCCATGGACGGCTGCGGTGGGTGGGGTACGTTCGATCGAGCGACACGCCGCACACGCTTTTGTGCGGTGTGGAGTGGGACGCTGACAGCGCGTTGCCGCCGTATCGTGCGCGTGTGGCTGGTGATGTGGCTGAGGAAGTAGTGCACGACGGGTGCGTGCAGGGCGAGCGTCTGTTTTACCAGGTTCAGGATGGGCGGGCGCGGTGCACGTGCGAGTACGTGCAGGATCTGGTGCCGGTTTCGACGATTGGAATCGCGGATACTGGGTGCCCGCGTATGCCTCACGCTCCGTCGCTGCTTTGGGCGCTACTCCGCACGTTTCGCTCCGATCTAATGGCGATACTGCCACCTTCGATTGCCGGTATGATATGTGAAGTGTCGACACCGTGGTTGCTGCAGCAGTTTGTGCTGTTCCTGCAGTCGAGCAAGGAGCGGGCGGGAGCGAGAAAAGGGCTGCTCTTCTTCTCGATTCTTGTGATCGTGAAGTTGGTGCAACCAGCGTTGATGAACAAGGAGATGCACCGCAGTCGGCGTGTTTCGAGCCTTTTCCGCACCTCGACACTCGCCCTGATTTTCGAAAAGTGTCTGACGATATCGCCGGATGCGCTGTCGAGACCGGACATGAACACTGGTCGTGTGCTAGCGATGGCGAGCTCTGACGTTGAGAACATCAAGGAATTTCCCACGCGGGTGATGTTCCTGTGGATGGCGCCGACTATGCTGACGCTGTACGTTGCCTACCTCTTCGTTCTCGTGGGGCCGAGTGCCCTGGCTGCTGTCCTGGTTTTTGCCGCTTTACTGCCTGTCCAAGGGGGGTTAACGAATGCTATGGGCGGCGCACAAGAGAACCTGTCGAGCTGCACGGATCAGCGCTTGCGTCGCACGAATGAACTGCTCTCCGGCATTCGTGTCGTGAAGATGATGGGATGGGAGTCGAAGTTTGTCTCGGCGATCGAAGATAACGCACGGGCCGATGAGTTGCGATTCCGACGAAGACTGCAGATGCGTCAAGTCGGGCTGTGGGCATGCGTGTTCTCTACTCCGACGTTCATGATCGCCGCTGTTCTCACTACCTACACCCTCAGCGGACACAAGCTTGACGCATCCGTGGTGTTCCCTCTCATCGCAGTTGTCAGTGCTATAACCTTCCCTGTAATGATGCTTCCGGAGGCCTTTACGTCGCTGGCGAAGTTCATTGTGTCGACGGGGCGCGTCACGCAGTTCTTGGAGTGCGACGACTCGCACATCATCGTCGAGCACGCGGAGCGCATGCTCAACAGCACAGGGAGTGGCGGCGCTGCTTCCGGGGACGCACCGCTGCCGAGTGCCGCTTCGGCCGACCTTGCAAGGGTGCTTGTGTCAGTGCCCGCCGCGCTTCCCGTGTACGAGCCGCGACACGTGGGGCTGCGCCGCGTGGCGCAGAGGATACTGTGCGCGGTGCTGCGCCGCCGTGTGCCGGTGGAGCTTCAGTGGCGGAGGGCGGCTGCTTCCCCTGCCATGGGTGAAGGCGGTAGGAAGCCTGCGCGCGTTGCTGTGGGGAACGATGATGGCGGTGTGGCTGTTGCGGGTGGTGAGAACGGGCGCGGCAGTGATAGTGGCGTGGCTCTGTACGCGATGGTGGACAGGGCTCTCCTGCGCGATGTGCGCGTGTCGTTCCCGCGCGCGCAGTTGACGGTTGTTGTTGGCGCGACGGGGAGCGGGAAGTCTGTGCTGCTGGCGACGCTTTTGGGTGCGTTCCGGTTCGAGGGGCATGTGAGCGTAGCGAAGTCGGTAGCTTACGTGCCGCAGCAGCCGTGGATCATGCAGGAAACGTTAGAGGCGAACATCACGTTCTTCGAGGGCGATCGGCGCGGCGCTGCTGGTGGCGCGGCGGTCGATAGCAATAGCGGCTCTGCGCAGGCGTTCTCGCCTCTGAGGTGCGCTGAGCAAGCTGCTGGCGCTGCTGCTGTGCCACGGTGCTTCAGCTGCGGCCGTGACGCGGCGAGCGAGCGTCTTGCGCGCGCTGTGCGGAGCTGCCAGCTGAATGCTGATGTGGCACTGATGGCGCGCGGGCTGGGGACAGAGATCGGCGAGCGTGGCATCAACCTGAGCGGTGGGCAGAAGGCGCGCGTGAGCCTTGCGCGCGCCGTGTATGCGGACCGCGACGTGTACCTGCTGGACGATCCGCTGTCGGCGCTGGACGCGCACGTTGGGCGGCGCGTGATGGACGAGGTTGTGCTGCGCGCGTTGTCGGGCAAGACGCGCGTGCTAGCGACGCACCAGCTGCAGGTGCTGCCGCACGCGGACCAGGTCGTGGTGATGCGCGAGGGCTGCGCCGTGTTTGCGGGCAGCTACGCTGCTTACGCTGCGTCGGAGTGGAAGGCGTACGTGGAGAGCGAGGAGGCCGCCGCCGCGAGCAAGAAGAGCGGGTGTGGCGGTGGTGATACTGTGGCGGAGTGCACCGAGGCCAGTTCTGCGTGGTCGTCTGCGGTGAACGTGGAGTCGTGTGTGGGCACGCGTGAGGAGCGTGACCACGCTCGGTGTGGCGGTGCCGATGGCCGTGCCAGCAAAGACGGCGGTGAGTGGGGGGAGAACGATGGCAGCCCTTTGCCGAGAATGCACTGGCAGGGCTCATGGGCCAGCGAGAACTCAGAGGCGGAAGGCTGCAACAATAGGGACTCTACGGTATGTAAAGGTGTTAGGCTCGCAAGCAGGTCTAAGGCCGCGCCGCGCACGGCGAAGGGCTCATCTGGGCTGGCTACGGGTGAGGATGCAGCGGACGGCGGCGACCTGATGACTGCGGAGGAGAAGGAGACAGGGCACACACCGTGGAGTGTGTACCGTGCCTACTTTGAGGCGGGCGGGGGCGTCCCGGTCGCAATTCAAATTGTGCTGCGCTACTTCGTTAGCGAAGCGCTCTCGACAGGCTCCAGCGTTTGGCTGACGCTGTGGTCAGTGAACTACTTTGGTTCTTCTCTCTCTACCAACGAACAGCTTGGCGTGTATCTTGGACTCGTCTTTGTGGTTGCTATCACAGTGTCTGCCAACGACCTGTTTATTTTTCAGTTCGCTCGCCGCGCTGCCTGCCGCTTGCACGCCATACTCTTGTACACGGTGAGCTCAGCCACGCTGACGTTTTTTGACCGCACACCACTGGGTCGCATTGTCAACCGGTTCAGCAGGGACGTGCACGTCCTTGACGATGAGCTGCCGGCGAACGTGATTCCCTTTTTGGGCATTACGGGATATGTCATGACGTCTTTAGCAGTGACGCTATACACTTCACCGCTTAGTGTGGTCGTGGTGCTGCTAGCGGTTTACGCTTTTGTGCGCCTGCTCAAGTTCTACGCGACAGTGGTGCGTGAGGTGCGACGGCGCAGCAGTGTGGGGCAGTCTCCGTTGCTCTCGCTGCTGGAGGAGGTGGTGCACGGGCGTGCGACGATCGCTGCATACGACAAGTCGCACGTGCTATTCGCGGAGGCGCTCCTGCGGCTGGACCTTGTGTACAGCTGCACCTACGTGGAGAAGGTTATGACGTTGTGGCTCGCTATCCGAATCGAGTACATTGCGTCATTGGCCGTTATTGCTGTGGGGCTGATCGGCGTTGTGGAGAAGCTGGTAGAGGCGTCGCCGGCGCTGCCGGAGGCGCGCGTGGGCCTGATCTCGCTGAGCCTCACCATGTGCCTCGATCTCAGCTGGTCGCTATCTGCACTCCTAGACCTTGCTGCCGCTGTCGAGGCGAGCATGAACAGTGTGCAGCGCGTTTGCCACTACATTGATCACGTGCCGCAGGAAGCGGCGCTCCTGGAGCCGCGGGACGCGTATGTGGCGCGCGCTGTGGCAGCGAGTGGCGGCAGCCGCAGGGGAGGAGGCGAGAGCTCCAGAAGTGCGAGCGACATCGTTGTGGCTGCCGGCGGTGATGACGACGAGGCCGCGCCTGCGCGACAGGGTGTTGCGGCAAGGAGCGGCGCGTTTGGCGCGCTGAGGCTGGAGCACGTGGACCTGCGGTACCGGCCAGGGCTGCCGCTGGTGCTGCGCGATGTCTGCTTTGCGATCGCCCCTGGGCAGAAGGTGGGCGTTGTGGGGCGGACGGGGAGCGGGAAGTCGACGCTGCTGCTGGCCTTCCTGCGGCTGGTGGAGGTAAGCGGCGGTCGCATGCTCGTCTGCGGGCGCGACGCGCGGACGTACACGCTTCCCGCTCTGCGGCGGCTGTTTTCCATGATCCCGCAGGATCCCGTATTGTTCGATGGCACGGTGCGCAGCAACGTGGATCCGTTCGGCGACGCGACGGACGAGGAGGTGCGTGCCGCGCTGGTGTCTGTGGGGTTTGTGGGGGTGGGCGGAAGCTCAGCTACCTTTCCTACAGCTTCTGGACTGCCGAGCGCTGCATCTGACAGCATGCCTGCGCTGGACACTGTGGTGCAGGGGGGCGGGTCGAACTTCAGCGTTGGGCAGCGCCAGCTGCTGTGCTTAGCGCGCGCGCTGCTGAAGAAGGGGAGTGCATTCATCCTGATGGACGAGGCGACGGCGAACGTGGACGCACAGCTGGACCAGACGGTGCAGCGTATTGTGGCGGAGCAGTTTGGCGCGTACACGGTGGTGACGATCGCGCACCGTCTGCACACGGTGGCGGCGTACGACGTAGTGCTTGTGATGGCGCGCGGGCGTGTGGTGGAGATGGGGCAGCCGCGGGCGCTGCTGGAGCGTCGCGACTCCGTGTTCTACGGTATGGTAGCGCAGAGCGCAGCGGTGGCTGATTCCCGTGAGGGCTTGTCGCGGGAGGATGCCGAGAGTGCGGCCAGTGGTGCGCGTTCGCGTGGCGTCAGTGACGGGGGCGCTGAGCTGGGGGCGGCTGGGCGCGAACACCGCGTGGAGGCCGCGGTCGCGTCGCTCCTGCGTCAGTGCAAGTGACCGGGATCGCACTCACGCGTCGCTGTATGCCTGCGTCTCTCGCTCTCTCTGGTGAGGGCTCGCAGCACCTCACTTGCCGCTTCGCTCGATCTATTCTTTCTCTTTTTGTGCTGCACTGGGCCGCACCACCACCGCATTGGTGACCGCACACCGCTCCCCACCGAGGCTTCCGCGGCTGCTGCACGTGCGTGGACGGCATTCGCGCAGCTCAGCTGCCGGCATTGGCTAGATTGACCGTCTTTTTTGCACAGCCACCCGTTGCAGCGGCGCGACACGGCTGGACCAGTTTTGTGTGCCCTTTGGCTGCTTAGCCCGCTGCGATAGGCGTGCCGGCCGGCAGCTGTCGTCTCCCCTGTCACCGGAGTGCTCGCTCTCCCCGCACCACATGCATTGCGAGCCGCCAGAGCTCTACACGATCATCTTAGTCTTTTGGCACACTTGCTGCGCCCTTTCGGCGCACGCTGCATGTTGCCCATCCGAGCCTGGGCATCTTGGAGATT

>BPK294/0 clone 1|LinJ31_V3.1460|500 bp UPS + CDS + 500 bp DWS|Pentamidine resistance protein 1

ACTGCGCATTTCTGCAAGTCTTCATTTCAGCGAGGCAAACCTCCCGACCGGTAGTGTGTCCCTTTTTTCCCGCTGCTCTTATTGCAATGCCGTGTTGTTCGATTGCCTCTTGACGGAGGGGTGGAGAAGCTTGAAAGGAGAGAGACGATGCGGCGCACGAGGACTCGCTAGCGATCCGATCGGATGAAGTGATTGCCATGCTGCTTTTTTTTTTGGCCGCGTCGAGAGGTGCTGCGAGACGGGGGTGACATCGCGGCAAACATGTTCATCGGTTCCACGTTGTTTCACTCGCTGAAGGCAATGACTGGCATCAAGGCTTGCCGTTGATGACGTCATGTGAGAGAGCAAGGACTCTTCGGATTCTCTACCTTTCGCTCAAGCAGGCTTGCTTGCCATGCGTGCTGGTGGCTTCTCATGTCAGTGCCAGGTTTTACATCTGGTGCGTGTGACACGTGTGCGTGCATTTCTTCAGCTTGACTTTTTCTCATACCTCTTCGTCTATGAGCAGTCAACGACCGGAGATGCCAGAAGGAGCGGCGAGTGATAGCGTCTACTCGTTCGGCGAGGTTAACCGACGGTTGTGGCTTCTGCGTTCTAATTCGTTACCGAAGGGTAGCGCTTGTGATGATGCGTTGGTGGACTGGGCTGCGATACGCGGGTTGCTGGAGGAGGCTCCGGAAGAGCGGAGCGGCGTTTTATCGCGTTACCTGCTGAGGTGGCTGAACCCATATGTTATGCTTGCGTGGAGGGAGCGGCTGGAGGAGGCGTACATGCCTCCACCACAGCGGGCGCACCGCGCCGTGTGCTGTGGCGCTCTGCTAAGCCGCGCGTTTCGCGAGGAGGAGGTTCGCGGGGGCCGCGACGCGTGGCGTGCGCGCGTTGAAGCTGCGCTTCCAGTGCAGTGTGGAGCCTGCGATGACCCCTCGGGGGAAAGTGAAGTGATTGGCGGCGATTATAACGGGCGATGGGGTGGCGCGCGCGTGGCGGCTATCAACGAGCTGTCACGTGTGAGGTTCCGTGGTGAGGAGGGTGTCCATGGACGGCTGCGGTGGGTGGGGTACGTTCGATCGAGCGACACGCCGCACACGCTTTTGTGCGGTGTGGAGTGGGACGCTGACAGCGCGTTGCCGCCGTATCGTGCGCGTGTGGCTGGTGATGTGGCTGAGGAAGTAGTGCACGACGGGTGCGTGCAGGGCGAGCGTCTGTTTTACCAGGTTCAGGATGGGCGGGCGCGGTGCACGTGCGAGTACGTGCAGGATCTGGTGCCGGTTTCGACGATTGGAATCGCGGATACTGGGTGCCCGCGTATGCCTCACGCTCCGTCGCTGCTTTGGGCGCTACTCCGCACGTTTCGCTCCGATCTAATGGCGATACTGCCACCTTCGATTGCCGGTATGATATGTGAAGTGTCGACACCGTGGTTGCTGCAGCAGTTTGTGCTGTTCCTGCAGTCGAGCAAGGAGCGGGCGGGAGCGAGAAAAGGGCTGCTCTTCTTCTCGATTCTTGTGATCGTGAAGTTGGTGCAACCAGCGTTGATGAACAAGGAGATGCACCGCAGTCGGCGTGTTTCGAGCCTTTTCCGCACCTCGACACTCGCCCTGATTTTCGAAAAGTGTCTGACGATATCGCCGGATGCGCTGTCGAGACCGGACATGAACACTGGTCGTGTGCTAGCGATGGCGAGCTCTGACGTTGAGAACATCAAGGAATTTCCCACGCGGGTGATGTTCCTGTGGATGGCGCCGACTATGCTGACGCTGTACGTTGCCTACCTCTTCGTTCTCGTGGGGCCGAGTGCCCTGGCTGCTGTCCTGGTTTTTGCCGCTTTACTGCCTGTCCAAGGGGGGTTAACGAATGCTATGGGCGGCGCACAAGAGAACCTGTCGAGCTGCACGGATCAGCGCTTGCGTCGCACGAATGAACTGCTCTCCGGCATTCGTGTCGTGAAGATGATGGGATGGGAGTCGAAGTTTGTCTCGGCGATCGAAGATAACGCACGGGCCGATGAGTTGCGATTCCGACGAAGACTGCAGATGCGTCAAGTCGGGCTGTGGGCATGCGTGTTCTCTACTCCGACGTTCATGATCGCCGCTGTTCTCACTACCTACACCCTCAGCGGACACAAGCTTGACGCATCCGTGGTGTTCCCTCTCATCGCAGTTGTCAGTGCTATAACCTTCCCTGTAATGATGCTTCCGGAGGCCTTTACGTCGCTGGCGAAGTTCATTGTGTCGACGGGGCGCGTCACGCAGTTCTTGGAGTGCGACGACTCGCACATCATCGTCGAGCACGCGGAGCGCATGCTCAACAGCACAGGGAGTGGCGGCGCTGCTTCCGGGGACGCACCGCTGCCGAGTGCCGCTTCGGCCGACCTTGCAAGGGTGCTTGTGTCAGTGCCCGCCGCGCTTCCCGTGTACGAGCCGCGACACGTGGGGCTGCGCCGCGTGGCGCAGAGGATACTGTGCGCGGTGCTGCGCCGCCGTGTGCCGGTGGAGCTTCAGTGGCGGAGGGCGGCTGCTTCCCCTGCCATGGGTGAAGGCGGTAGGAAGCCTGCGCGCGTTGCTGTGGGGAACGATGATGGCGGTGTGGCTGTTGCGGGTGGTGAGAACGGGCGCGGCAGTGATAGTGGCGTGGCTCTGTACGCGATGGTGGACAGGGCTCTCCTGCGCGATGTGCGCGTGTCGTTCCCGCGCGCGCAGTTGACGGTTGTTGTTGGCGCGACGGGGAGCGGGAAGTCTGTGCTGCTGGCGACGCTTTTGGGTGCGTTCCGGTTCGAGGGGCATGTGAGCGTAGCGAAGTCGGTAGCTTACGTGCCGCAGCAGCCGTGGATCATGCAGGAAACGTTAGAGGCGAACATCACGTTCTTCGAGGGCGATCGGCGCGGCGCTGCTGGTGGCGCGGCGGTCGATAGCAATAGCGGCTCTGCGCAGGCGTTCTCGCCTCTGAGGTGCGCTGAGCAAGCTGCTGGCGCTGCTGCTGTGCCACGGTGCTTCAGCTGCGGCCGTGACGCGGCGAGCGAGCGTCTTGCGCGCGCTGTGCGGAGCTGCCAGCTGAATGCTGATGTGGCACTGATGGCGCGCGGGCTGGGGACAGAGATCGGCGAGCGTGGCATCAACCTGAGCGGTGGGCAGAAGGCGCGCGTGAGCCTTGCGCGCGCCGTGTATGCGGACCGCGACGTGTACCTGCTGGACGATCCGCTGTCGGCGCTGGACGCGCACGTTGGGCGGCGCGTGATGGACGAGGTTGTGCTGCGCGCGTTGTCGGGCAAGACGCGCGTGCTAGCGACGCACCAGCTGCAGGTGCTGCCGCACGCGGACCAGGTCGTGGTGATGCGCGAGGGCTGCGCCGTGTTTGCGGGCAGCTACGCTGCTTACGCTGCGTCGGAGTGGAAGGCGTACGTGGAGAGCGAGGAGGCCGCCGCCGCGAGCAAGAAGAGCGGGTGTGGCGGTGGTGATACTGTGGCGGAGTGCACCGAGGCCAGTTCTGCGTGGTCGTCTGCGGTGAACGTGGAGTCGTGTGTGGGCACGCGTGAGGAGCGTGACCACGCTCGGTGTGGCGGTGCCGATGGCCGTGCCAGCAAAGACGGCGGTGAGTGGGGGGAGAACGATGGCAGCCCTTTGCCGAGAATGCACTGGCAGGGCTCATGGGCCAGCGAGAACTCAGAGGCGGAAGGCTGCAACAATAGGGACTCTACGGTATGTAAAGGTGTTAGGCTCGCAAGCAGGTCTAAGGCCGCGCCGCGCACGGCGAAGGGCTCATCTGGGCTGGCTACGGGTGAGGATGCAGCGGACGGCGGCGACCTGATGACTGCGGAGGAGAAGGAGACAGGGCACACACCGTGGAGTGTGTACCGTGCCTACTTTGAGGCGGGCGGGGGCGTCCCGGTCGCAATTCAAATTGTGCTGCGCTACTTCGTTAGCGAAGCGCTCTCGACAGGCTCCAGCGTTTGGCTGACGCTGTGGTCAGTGAACTACTTTGGTTCTTCTCTCTCTACCAACGAACAGCTTGGCGTGTATCTTGGACTCGTCTTTGTGGTTGCTATCACAGTGTCTGCCAACGACCTGTTTATTTTTCAGTTCGCTCGCCGCGCTGCCTGCCGCTTGCACGCCATACTCTTGTACACGGTGAGCTCAGCCACGCTGACGTTTTTTGACCGCACACCACTGGGTCGCATTGTCAACCGGTTCAGCAGGGACGTGCACGTCCTTGACGATGAGCTGCCGGCGAACGTGATTCCCTTTTTGGGCATTACGGGATATGTCATGACGTCTTTAGCAGTGACGCTATACACTTCACCGCTTAGTGTGGTCGTGGTGCTGCTAGCGGTTTACGCTTTTGTGCGCCTGCTCAAGTTCTACGCGACAGTGGTGCGTGAGGTGCGACGGCGCAGCAGTGTGGGGCAGTCTCCGTTGCTCTCGCTGCTGGAGGAGGTGGTGCACGGGCGTGCGACGATCGCTGCATACGACAAGTCGCACGTGCTATTCGCGGAGGCGCTCCTGCGGCTGGACCTTGTGTACAGCTGCACCTACGTGGAGAAGGTTATGACGTTGTGGCTCGCTATCCGAATCGAGTACATTGCGTCATTGGCCGTTATTGCTGTGGGGCTGATCGGCGTTGTGGAGAAGCTGGTAGAGGCGTCGCCGGCGCTGCCGGAGGCGCGCGTGGGCCTGATCTCGCTGAGCCTCACCATGTGCCTCGATCTCAGCTGGTCGCTATCTGCACTCCTAGACCTTGCTGCCGCTGTCGAGGCGAGCATGAACAGTGTGCAGCGCGTTTGCCACTACATTGATCACGTGCCGCAGGAAGCGGCGCTCCTGGAGCCGCGGGACGCGTATGTGGCGCGCGCTGTGGCAGCGAGTGGCGGCAGCCGCAGGGGAGGAGGCGAGAGCTCCAGAAGTGCGAGCGACATCGTTGTGGCTGCCGGCGGTGATGACGACGAGGCCGCGCCTGCGCGACAGGGTGTTGCGGCAAGGAGCGGCGCGTTTGGCGCGCTGAGGCTGGAGCACGTGGACCTGCGGTACCGGCCAGGGCTGCCGCTGGTGCTGCGCGATGTCTGCTTTGCGATCGCCCCTGGGCAGAAGGTGGGCGTTGTGGGGCGGACGGGGAGCGGGAAGTCGACGCTGCTGCTGGCCTTCCTGCGGCTGGTGGAGGTAAGCGGCGGTCGCATGCTCGTCTGCGGGCGCGACGCGCGGACGTACACGCTTCCCGCTCTGCGGCGGCTGTTTTCCATGATCCCGCAGGATCCCGTATTGTTCGATGGCACGGTGCGCAGCAACGTGGATCCGTTCGGCGACGCGACGGACGAGGAGGTGCGTGCCGCGCTGGTGTCTGTGGGGTTTGTGGGGGTGGGCGGAAGCTCAGCTACCTTTCCTACAGCTTCTGGACTGCCGAGCGCTGCATCTGACAGCATGCCTGCGCTGGACACTGTGGTGCAGGGGGGCGGGTCGAACTTCAGCGTTGGGCAGCGCCAGCTGCTGTGCTTAGCGCGCGCGCTGCTGAAGAAGGGGAGTGCATTCATCCTGATGGACGAGGCGACGGCGAACGTGGACGCACAGCTGGACCAGACGGTGCAGCGTATTGTGGCGGAGCAGTTTGGCGCGTACACGGTGGTGACGATCGCGCACCGTCTGCACACGGTGGCGGCGTACGACGTAGTGCTTGTGATGGCGCGCGGGCGTGTGGTGGAGATGGGGCAGCCGCGGGCGCTGCTGGAGCGTCGCGACTCCGTGTTCTACGGTATGGTAGCGCAGAGCGCAGCGGTGGCTGATTCCCGTGAGGGCTTGTCGCGGGAGGATGCCGAGAGTGCGGCCAGTGGTGCGCGTTCGCGTGGCGTCAGTGACGGGGGCGCTGAGCTGGGGGCGGCTGGGCGCGAACACCGCGTGGAGGCCGCGGTCGCGTCGCTCCTGCGTCAGTGCAAGTGACCGGGATCGCACTCACGCGTCGCTGTATGCCTGCGTCTCTCGCTCTCTCTGGTGAGGGCTCGCAGCACCTCACTTGCCGCTTCGCTCGATCTATTCTTTCTCTTTTTGTGCTGCACTGGGCCGCACCACCACCGCATTGGTGACCGCACACCGCTCCCCACCGAGGCTTCCGCGGCTGCTGCACGTGCGTGGACGGCATTCGCGCAGCTCAGCTGCCGGCATTGGCTAGATTGACCGTCTTTTTTGCACAGCCACCCGTTGCAGCGGCGCGACACGGCTGGACCAGTTTTGTGTGCCCTTTGGCTGCTTAGCCCGCTGCGATAGGCGTGCCGGCCGGCAGCTGTCGTCTCCCCTGTCACCGGAGTGCTCGCTCTCCCCGCACCACATGCATTGCGAGCCGCCAGAGCTCTACACGATCATCTTAGTCTTTTGGCACACTTGCTGCGCCCTTTCGGCGCACGCTGCATGTTGCCCATCCGAGCCTGGGCATCTTGGAGATT

>BPK298/0 clone 8|LinJ31_V3.1460|500 bp UPS + CDS + 500 bp DWS|Pentamidine resistance protein 1

ACTGCGCATTTCTGCAAGTCTTCATTTCAGCGAGGCAAACCTCCCGACCGGTAGTGTGTCCCTTTTTTCCCGCTGCTCTTATTGCAATGCCGTGTTGTTCGATTGCCTCTTGACGGAGGGGTGGAGAAGCTTGAAAGGAGAGAGACGATGCGGCGCACGAGGACTCGCTAGCGATCCGATCGGATGAAGTGATTGCCATGCTGCTTTTTTTTTTGGCCGCGTCGAGAGGTGCTGCGAGACGGGGGTGACATCGCGGCAAACATGTTCATCGGTTCCACGTTGTTTCACTCGCTGAAGGCAATGACTGGCATCAAGGCTTGCCGTTGATGACGTCATGTGAGAGAGCAAGGACTCTTCGGATTCTCTACCTTTCGCTCAAGCAGGCTTGCTTGCCATGCGTGCTGGTGGCTTCTCATGTCAGTGCCAGGTTTTACATCTGGTGCGTGTGACACGTGTGCGTGCATTTCTTCAGCTTGACTTTTTCTCATACCTCTTCGTCTATGAGCAGTCAACGACCGGAGATGCCAGAAGGAGCGGCGAGTGATAGCGTCTACTCGTTCGGCGAGGTTAACCGACGGTTGTGGCTTCTGCGTTCTAATTCGTTACCGAAGGGTAGCGCTTGTGATGATGCGTTGGTGGACTGGGCTGCGATACGCGGGTTGCTGGAGGAGGCTCCGGAAGAGCGGAGCGGCGTTTTATCGCGTTACCTGCTGAGGTGGCTGAACCCATATGTTATGCTTGCGTGGAGGGAGCGGCTGGAGGAGGCGTACATGCCTCCACCACAGCGGGCGCACCGCGCCGTGTGCTGTGGCGCTCTGCTAAGCCGCGCGTTTCGCGAGGAGGAGGTTCGCGGGGGCCGCGACGCGTGGCGTGCGCGCGTTGAAGCTGCGCTTCCAGTGCAGTGTGGAGCCTGCGATGACCCCTCGGGGGAAAGTGAAGTGATTGGCGGCGATTATAACGGGCGATGGGGTGGCGCGCGCGTGGCGGCTATCAACGAGCTGTCACGTGTGAGGTTCCGTGGTGAGGAGGGTGTCCATGGACGGCTGCGGTGGGTGGGGTACGTTCGATCGAGCGACACGCCGCACACGCTTTTGTGCGGTGTGGAGTGGGACGCTGACAGCGCGTTGCCGCCGTATCGTGCGCGTGTGGCTGGTGATGTGGCTGAGGAAGTAGTGCACGACGGGTGCGTGCAGGGCGAGCGTCTGTTTTACCAGGTTCAGGATGGGCGGGCGCGGTGCACGTGCGAGTACGTGCAGGATCTGGTGCCGGTTTCGACGATTGGAATCGCGGATACTGGGTGCCCGCGTATGCCTCACGCTCCGTCGCTGCTTTGGGCGCTACTCCGCACGTTTCGCTCCGATCTAATGGCGATACTGCCACCTTCGATTGCCGGTATGATATGTGAAGTGTCGACACCGTGGTTGCTGCAGCAGTTTGTGCTGTTCCTGCAGTCGAGCAAGGAGCGGGCGGGAGCGAGAAAAGGGCTGCTCTTCTTCTCGATTCTTGTGATCGTGAAGTTGGTGCAACCAGCGTTGATGAACAAGGAGATGCACCGCAGTCGGCGTGTTTCGAGCCTTTTCCGCACCTCGACACTCGCCCTGATTTTCGAAAAGTGTCTGACGATATCGCCGGATGCGCTGTCGAGACCGGACATGAACACTGGTCGTGTGCTAGCGATGGCGAGCTCTGACGTTGAGAACATCAAGGAATTTCCCACGCGGGTGATGTTCCTGTGGATGGCGCCGACTATGCTGACGCTGTACGTTGCCTACCTCTTCGTTCTCGTGGGGCCGAGTGCCCTGGCTGCTGTCCTGGTTTTTGCCGCTTTACTGCCTGTCCAAGGGGGGTTAACGAATGCTATGGGCGGCGCACAAGAGAACCTGTCGAGCTGCACGGATCAGCGCTTGCGTCGCACGAATGAACTGCTCTCCGGCATTCGTGTCGTGAAGATGATGGGATGGGAGTCGAAGTTTGTCTCGGCGATCGAAGATAACGCACGGGCCGATGAGTTGCGATTCCGACGAAGACTGCAGATGCGTCAAGTCGGGCTGTGGGCATGCGTGTTCTCTACTCCGACGTTCATGATCGCCGCTGTTCTCACTACCTACACCCTCAGCGGACACAAGCTTGACGCATCCGTGGTGTTCCCTCTCATCGCAGTTGTCAGTGCTATAACCTTCCCTGTAATGATGCTTCCGGAGGCCTTTACGTCGCTGGCGAAGTTCATTGTGTCGACGGGGCGCGTCACGCAGTTCTTGGAGTGCGACGACTCGCACATCATCGTCGAGCACGCGGAGCGCATGCTCAACAGCACAGGGAGTGGCGGCGCTGCTTCCGGGGACGCACCGCTGCCGAGTGCCGCTTCGGCCGACCTTGCAAGGGTGCTTGTGTCAGTGCCCGCCGCGCTTCCCGTGTACGAGCCGCGACACGTGGGGCTGCGCCGCGTGGCGCAGAGGATACTGTGCGCGGTGCTGCGCCGCCGTGTGCCGGTGGAGCTTCAGTGGCGGAGGGCGGCTGCTTCCCCTGCCATGGGTGAAGGCGGTAGGAAGCCTGCGCGCGTTGCTGTGGGGAACGATGATGGCGGTGTGGCTGTTGCGGGTGGTGAGAACGGGCGCGGCAGTGATAGTGGCGTGGCTCTGTACGCGATGGTGGACAGGGCTCTCCTGCGCGATGTGCGCGTGTCGTTCCCGCGCGCGCAGTTGACGGTTGTTGTTGGCGCGACGGGGAGCGGGAAGTCTGTGCTGCTGGCGACGCTTTTGGGTGCGTTCCGGTTCGAGGGGCATGTGAGCGTAGCGAAGTCGGTAGCTTACGTGCCGCAGCAGCCGTGGATCATGCAGGAAACGTTAGAGGCGAACATCACGTTCTTCGAGGGCGATCGGCGCGGCGCTGCTGGTGGCGCGGCGGTCGATAGCAATAGCGGCTCTGCGCAGGCGTTCTCGCCTCTGAGGTGCGCTGAGCAAGCTGCTGGCGCTGCTGCTGTGCCACGGTGCTTCAGCTGCGGCCGTGACGCGGCGAGCGAGCGTCTTGCGCGCGCTGTGCGGAGCTGCCAGCTGAATGCTGATGTGGCACTGATGGCGCGCGGGCTGGGGACAGAGATCGGCGAGCGTGGCATCAACCTGAGCGGTGGGCAGAAGGCGCGCGTGAGCCTTGCGCGCGCCGTGTATGCGGACCGCGACGTGTACCTGCTGGACGATCCGCTGTCGGCGCTGGACGCGCACGTTGGGCGGCGCGTGATGGACGAGGTTGTGCTGCGCGCGTTGTCGGGCAAGACGCGCGTGCTAGCGACGCACCAGCTGCAGGTGCTGCCGCACGCGGACCAGGTCGTGGTGATGCGCGAGGGCTGCGCCGTGTTTGCGGGCAGCTACGCTGCTTACGCTGCGTCGGAGTGGAAGGCGTACGTGGAGAGCGAGGAGGCCGCCGCCGCGAGCAAGAAGAGCGGGTGTGGCGGTGGTGATACTGTGGCGGAGTGCACCGAGGCCAGTTCTGCGTGGTCGTCTGCGGTGAACGTGGAGTCGTGTGTGGGCACGCGTGAGGAGCGTGACCACGCTCGGTGTGGCGGTGCCGATGGCCGTGCCAGCAAAGACGGCGGTGAGTGGGGGGAGAACGATGGCAGCCCTTTGCCGAGAATGCACTGGCAGGGCTCATGGGCCAGCGAGAACTCAGAGGCGGAAGGCTGCAACAATAGGGACTCTACGGTATGTAAAGGTGTTAGGCTCGCAAGCAGGTCTAAGGCCGCGCCGCGCACGGCGAAGGGCTCATCTGGGCTGGCTACGGGTGAGGATGCAGCGGACGGCGGCGACCTGATGACTGCGGAGGAGAAGGAGACAGGGCACACACCGTGGAGTGTGTACCGTGCCTACTTTGAGGCGGGCGGGGGCGTCCCGGTCGCAATTCAAATTGTGCTGCGCTACTTCGTTAGCGAAGCGCTCTCGACAGGCTCCAGCGTTTGGCTGACGCTGTGGTCAGTGAACTACTTTGGTTCTTCTCTCTCTACCAACGAACAGCTTGGCGTGTATCTTGGACTCGTCTTTGTGGTTGCTATCACAGTGTCTGCCAACGACCTGTTTATTTTTCAGTTCGCTCGCCGCGCTGCCTGCCGCTTGCACGCCATACTCTTGTACACGGTGAGCTCAGCCACGCTGACGTTTTTTGACCGCACACCACTGGGTCGCATTGTCAACCGGTTCAGCAGGGACGTGCACGTCCTTGACGATGAGCTGCCGGCGAACGTGATTCCCTTTTTGGGCATTACGGGATATGTCATGACGTCTTTAGCAGTGACGCTATACACTTCACCGCTTAGTGTGGTCGTGGTGCTGCTAGCGGTTTACGCTTTTGTGCGCCTGCTCAAGTTCTACGCGACAGTGGTGCGTGAGGTGCGACGGCGCAGCAGTGTGGGGCAGTCTCCGTTGCTCTCGCTGCTGGAGGAGGTGGTGCACGGGCGTGCGACGATCGCTGCATACGACAAGTCGCACGTGCTATTCGCGGAGGCGCTCCTGCGGCTGGACCTTGTGTACAGCTGCACCTACGTGGAGAAGGTTATGACGTTGTGGCTCGCTATCCGAATCGAGTACATTGCGTCATTGGCCGTTATTGCTGTGGGGCTGATCGGCGTTGTGGAGAAGCTGGTAGAGGCGTCGCCGGCGCTGCCGGAGGCGCGCGTGGGCCTGATCTCGCTGAGCCTCACCATGTGCCTCGATCTCAGCTGGTCGCTATCTGCACTCCTAGACCTTGCTGCCGCTGTCGAGGCGAGCATGAACAGTGTGCAGCGCGTTTGCCACTACATTGATCACGTGCCGCAGGAAGCGGCGCTCCTGGAGCCGCGGGACGCGTATGTGGCGCGCGCTGTGGCAGCGAGTGGCGGCAGCCGCAGGGGAGGAGGCGAGAGCTCCAGAAGTGCGAGCGACATCGTTGTGGCTGCCGGCGGTGATGACGACGAGGCCGCGCCTGCGCGACAGGGTGTTGCGGCAAGGAGCGGCGCGTTTGGCGCGCTGAGGCTGGAGCACGTGGACCTGCGGTACCGGCCAGGGCTGCCGCTGGTGCTGCGCGATGTCTGCTTTGCGATCGCCCCTGGGCAGAAGGTGGGCGTTGTGGGGCGGACGGGGAGCGGGAAGTCGACGCTGCTGCTGGCCTTCCTGCGGCTGGTGGAGGTAAGCGGCGGTCGCATGCTCGTCTGCGGGCGCGACGCGCGGACGTACACGCTTCCCGCTCTGCGGCGGCTGTTTTCCATGATCCCGCAGGATCCCGTATTGTTCGATGGCACGGTGCGCAGCAACGTGGATCCGTTCGGCGACGCGACGGACGAGGAGGTGCGTGCCGCGCTGGTGTCTGTGGGGTTTGTGGGGGTGGGCGGAAGCTCAGCTACCTTTCCTACAGCTTCTGGACTGCCGAGCGCTGCATCTGACAGCATGCCTGCGCTGGACACTGTGGTGCAGGGGGGCGGGTCGAACTTCAGCGTTGGGCAGCGCCAGCTGCTGTGCTTAGCGCGCGCGCTGCTGAAGAAGGGGAGTGCATTCATCCTGATGGACGAGGCGACGGCGAACGTGGACGCACAGCTGGACCAGACGGTGCAGCGTATTGTGGCGGAGCAGTTTGGCGCGTACACGGTGGTGACGATCGCGCACCGTCTGCACACGGTGGCGGCGTACGACGTAGTGCTTGTGATGGCGCGCGGGCGTGTGGTGGAGATGGGGCAGCCGCGGGCGCTGCTGGAGCGTCGCGACTCCGTGTTCTACGGTATGGTAGCGCAGAGCGCAGCGGTGGCTGATTCCCGTGAGGGCTTGTCGCGGGAGGATGCCGAGAGTGCGGCCAGTGGTGCGCGTTCGCGTGGCGTCAGTGACGGGGGCGCTGAGCTGGGGGCGGCTGGGCGCGAACACCGCGTGGAGGCCGCGGTCGCGTCGCTCCTGCGTCAGTGCAAGTGACCGGGATCGCACTCACGCGTCGCTGTATGCCTGCGTCTCTCGCTCTCTCTGGTGAGGGCTCGCAGCACCTCACTTGCCGCTTCGCTCGATCTATTCTTTCTCTTTTTGTGCTGCACTGGGCCGCACCACCACCGCATTGGTGACCGCACACCGCTCCCCACCGAGGCTTCCGCGGCTGCTGCACGTGCGTGGACGGCATTCGCGCAGCTCAGCTGCCGGCATTGGCTAGATTGACCGTCTTTTTTGCACAGCCACCCGTTGCAGCGGCGCGACACGGCTGGACCAGTTTTGTGTGCCCTTTGGCTGCTTAGCCCGCTGCGATAGGCGTGCCGGCCGGCAGCTGTCGTCTCCCCTGTCACCGGAGTGCTCGCTCTCCCCGCACCACATGCATTGCGAGCCGCCAGAGCTCTACACGATCATCTTAGTCTTTTGGCACACTTGCTGCGCCCTTTCGGCGCACGCTGCATGTTGCCCATCCGAGCCTGGGCATCTTGGAGATT

### 11. MULTIDRUG RESISTANCE PROTEIN A

>BPK035/0 clone 1|LinJ23_V3.0290|500 bp UPS + CDS + 500 bp DWS|multidrug resistance protein A

CTCCGCGCCGCATGCGAAGCCTGTTGTCCGCGTAGTTGCGTACGTGTGTATCTGCGCGCGTTGACTCGTGTCCTCTTCGCACCTCCTTCTGCTGGTTTGCCGCTCTCACCCGTAAGCGTGAATCAGTCTTCACGAGGCGCTTAGAGTTTCTTTTCTTTCGTGGAGGTGGTGGTCGCCAAGTGTGTGAGCTGATCGGTGCGCGAGGCGTGCGTGTGCCGAGTGCATCCTGTCCCCGACCCCCGCCTCTGGCTCCTCCGGTGCTACGCCTGAGTCTTCCCTGGCCCAGCCAGCACGGTGCCCGTCGTTGAAAGAGTGTGCCAAGACACACCTCGCCCCCTCGGTTTGTGCGCGGTCTGTCACACTGGTGCTGTTCGACCGCTGTTGTGCGGCTCGTAGGACAGGGTTGTGCGAACTACGCCTCCCTCGTTCTCTTCGCATAGGAAAGACATTGTAGACATCGGTGCATACATTGGGGGGGGGCATCCACAGCGCGCAGGGCAATGGCGACACCAGACTTTGTACGTTTATCACCGACTGACGAGTCCAGCGAGCTCATAATCCATGCTCCCTCGCAGCGGTGCCACACCCGATGTGGCGCAGAAAACGGTCATACCACCAGCATCATTACTGATTTGGAGGCGGTGGACGATGTTGCGCAGGTGCGCTGCCAGCAGCAGGCGCAGCGCGAGTTTGCGGAGCAGCTGGACGAGCTGTGGGGCAGCGAGCCCACCTACACGCCGACGGTAGAGGACCAAGCGAGCTGGCTGCAGCAGCTGCACTACGGATGGATCGGGGACTACATCTACAAGGCTGCGACGGGGAGCATTACCGAGGCGGACCTGCCACCGCCGTCGCGGAGCACACGGACCTACCACACGGGGCGCAAGCTGTCGCGGCAGGCGCATGCCGACATCGACGCAAGCCGGCGGTGGGACGGGTATGTCGGGTGCGAGGTCATGTACGAGGCGGAGGCGGAAACCAGCGGCGCGCTGCGGTGGGTCGGGTATTTGCAGCAGTCGGACTACCCGCGATCGCTGGTAGCTGGGGTGGAGTGGCGTGTGCCGCCGCGGTACCGGCGGCAGGCCACGCCGGGCAGCGCGGCGGGGCTTCACAACGGCGTCGTGCACGGTGAGCGGCTGTTTCGGCCATACGAAGACAACTATCTCTGCTCGTGCGAACCGGTTGAACGGCTTTACCTAAGCTCGACGTGCAGTCTGATGCGTCCCGGGCCGCCGCCGTCGCCGGATCTCCTGTTTACACTGTTCAAGGCGCACTCGTACCACGTGTGGGCGCAGATCCTGCCGAAGCTGCTGGCAGACGTTACCGCGCTGATGCTGCCGGTGTTGTTGGAGTACTTTGTGAAGTATTTGGACGCCGACAACGCGACGTGGGGCTGGGGTCTGGGACTTGTGCTGACACTCTTCCTCACGAACGTGATCCAGAGCTGCGCAGCGCACAAGTACGACCACATCAGCATCCGCAGCGCGGCGCTGTTTGAGACGTCGTCGATGGCGCTGCTGTTCGAGAAGTGCTTCACAGTTTCGCAGCGGTCGCTGCAGCGCCCAGACATGTCCGTGGGTCGCATCATGAACATGGTCGGGAATGACGTTGATAACATCGGTAGTCTCAACTGGTACGTGATGTATTTTTGGAGCGCCCCGCTGCAACTGACACTGTGCATGCTGCTGCTGATAAGGCTGGTCGGGTGGTTCGCGCTGCCCGGCATGGCCGTCTTGTTGGTGACGCTCCCGTTACAAGGTGCCATTTCTAAGCATGTTCAGGAGGTGTCTGAGCGAATGGCGAGCGTGGTGGACCTGCGTATCAAGCGCACGAGCGAGCTGCTCTCCGGTGCCCGAATCGTGAAATTCATGGGGTGGGAACCTGTCTTCCTCGCCCGGATCGAAGACGCGCGCAGTCGCGAGCTGCAGTGTCTGCGCGATGTGCATCTCGCCAATGTCATCTTCATGTTCGTGAACGACGCGACGCCGACGTTGGTCGTCGCTGTAGTCTTTATCTTGTACCATGCGAGTGGGCAGGCGCTGAGACCGGAGATTGTGTTTCCGACGATTGCACTGCTAAACACGATGCGCGTGTCGTTCTTTATGATCCCGCTTATCGTCTCCGCCATCCTGCAGTGTTTGGTGTCCACAAAGCGGGTCACCACTTTTGTGGAGTGCCCTGACACGCGCTCACAGGTGCGGGACATTGCTGGCATCGACGCAGCTGGTGCCGCGGCCATCTTCAAGGATGTGTCCATCCACACCTATCTGCCGGCGAAACTGCCCCAGTGCAAGTCGCGCTTGACGACCATGCAGCGCATTACGCTGTGGTTCCGCCGGCGCGGTGTGCCGGAGGCGGAGTGGTACGATCTGGACGGCCCGGATGCGGGTGCGTCTTCGCCGACGGTGCACCTTCCAACGCTCGACATGGGCAGCGCACAGACTGCCAGTGCGGATGGTGACGGCGCTGCCACTGGCGGCGGGGACGAGGAGGGGGACGTGGAAGAGAGAGTCGCACAGTACTACCAACTTGTGCCGAAGGAGCTGCTGCGGAACGTCAACCTCGTTATCCCTCAGTTCAAGTTGACGATGGTGATTGGCGCGACCGGGAGCGGGAAGTCGACGCTGCTGGGCTCTCTGATGGGCGAGTACGACGTGCAGAGTGGCGAGGTGTGGGCGGAGCGGAGCATCGCGTACGTGCCGCAGCAGGCGTGGATCATGAACGCGACGGTGCGCGACAACATCCTGTTCTTCGACGAGGAGCGCGCCGCAGACTTGCAGGACGTAATCCGTTGCTGCCAGCTGGAGGCGGACCTTGCGCAGCTTGGCGGGGGGCTGGAGACGGAGATCGGGGAAATGGGCGTGAACCTGAGCGGCGGGCAGAAGGCGCGCGTGAGCCTTGCGCGCGCCGTGTACGCGAACCGCGACGTGTACCTGCTGGACGACCCCCTGTCCGCGCTGGACGCGCACGTTGGCCAACGCGTCGTGCAAGACGTCATCCGCGGACGACTGCACGGCAAGACGCGCGTGCTTGCGACGCACCAGATTCATCTGCTGCCGCTCGCGGACTACATTGTGGTGTTGCAGCGCGGCCGCATCGCATTCGCGGGCGATTACGCTGCCTTTGCGGCGACTTCGCTGGAGGAGACGCTGCGCGGTGAGCTAAAGGAGAACAAGGACGCGGAGTCTCGCGGCAGCGATGCGGACGCGGAAGCGGCCAGAGCGGAGACGGCACCTGACATTGCGGAAGCGCATGAGCCGAAAGTTGAGCAGGAGACGAGCCTTGCAGGAGGCGAAGACCCCTTGAGGTCCGATGTCGAGGCCGGTAGGCTGATGACCAGGGAGGAGAAGGCAACCGGTCAGGTACCGTGGTCAACCTACGTGGCGTATCTGAGGTCGTGTGGCGGTCTGGCTGCTTGGGGATTCCTGCTAGCCACCTTTGCGGTGACAGAGAGCGTGACTGCGGCCAACGGCGTGTGGTTGTCGATATGGTCAACAGGCTCGCTTAGGTGTAGCGCGGCCACGTACCTGTACGTCTATCTTTTCATTGTTTTCCTCAACATCTTTAACTCCCCGCTGCGGTGTTTCTTATGCTATTACCTTATGCGAATGGGTAGCCGCAACTTGCATCGCGACCTCCTGGAGTCCATCGGCGTTGCGCGGATGTCCTTCTTCGACACGACGCCCTTGGGACGCGTGCTGAACCGGTTCACAAAGGACATGGGCATCCTTGATAACACGCTGAACGACAGCTACCTTTACCTGCTGCAGTACTTCTTTTCCATGTGCTCCAAGGTTATTATTCTGTCGGCCGCGCAGCCGTTTGTGCTTGTGGCCATTGTGCCGTGTGTGTTCATCTACTACAAGCTGATGCAGATCTACAGCGCGTCGAACCGCGAGACACGCCGCATCAAGAGCATCGCGCACTCGCCCGTGTTCACGCTGCTGGAGGAGTCGCTGCAAGGGCAGCGCACCATCGCGACGTACGGCAAGATGCACCTCGTACTGCAGGAAGCACTGAGGCGGCTCGATGTGGTCTACAGCGCGCTGTACATGCAGAACGTCTCCAACCGCTGGCTCGGTGTCCGCCTCGAGTTTGTGAGCTGTGTTATCACTTTCGTGGTGGCCCTCATTGGTGTGATTGGGAAAATGGAGCGGGCTTCGAGTCAGAGCATCGGTCTCATCTCGCTGTCACTGACGATGGCGATCACGCTGACGGAAACGCTCAATTGGCTGGTGCGGCAGGTTGCGACGGTGGAGGCAAACATGAACAGCGTGGAGCGTGTGATGTATTACACCCACGAAGTGGAGCACGAGTACGTGCCGGAGATGAAGGAGTTAGTGGCACAGCTGGTAGGGAGCGAGTCAGGGACAGCGGCGGAANNNNNNNNNNNNNNNNNNNNNNNNNNNNNNNNNNNNNNNNNNNNNNNNNNNNNNNNNNNNNNNNNNNNNNNNNNNNNNNNNNNNNNNNNNNNNNNNNNNNNNNNNNNNNNNNNNNNNNNNNNNNNNNNNNNNNNNNNNNNNNNNNNNNNNNNNNNNNNNNNNNNNNNNNNNNNNNNNNNNNNNNNNNNNNNNNNNNNNNNNNNNNNNNNNNNNNNNNNNNNNNNNNNNNNNNNNNNNNNNNNNNNNNNNNNNNNNNNNNNNNNNNNNNNNNNNNNNNNNNNNNNNNNNNNNNNNNNNNNNNNNNNNNNNNNNNNNNNNNNNNNNNNNNNNNNNNNNNNNNNNNNNNNNNNNNNNNNNNNNNNNNNNNNNNNNNNNNNNNNNNNNNNNNNNNNNNNNNNNNNNNNNNNNNNNNNNNNNNNNNNNNNNNNNNNNNNNNNNNNNNNNNNNNNNNNNNNNNNNNNNNNNNNNNNNNNNNNNNNNNNNNNNNNNNNNNNNNNNNNNNNNNNNNNNNNNNNNNNNNNNNNNNNNNNNNNNNNNNNNNNNNNNNNNNNNNNNNNNNNNNNNNNNNNNNNNNNNNNNNNNNNNNNNNNNNNNNNNNNNNNNNNNNNNNNNNNNNNNNNNNNNNNNNNNNNNNNNNNNNNNNNNNNNNNNNNNNNNNNNNNNNNNNNNNNNNNNNNNNNNNNNNNNNNNNNNNNNNNNNNNNNNNNNNNNNNNNNNNNNNNNNNNNNNNNNNNNNNNNNNNNNNNNNNNNNNNNNNNNNNNNNNNNNNNNNNNNNNNNNACTGGGCCGCCGTGGTGCCAAAGACTTCGTCGATCTCCTCGGCTGAGTCGCACGAGTCTGTGCAGCAGCCCGTGCGCACCAATGAAAGACTCCGTCGCAACCTGCCGATCTCAAAGCAGAAGCCGCTCGTCGTACGCCACCCCGCATGCACGTGCAGGAGAACGAAGTGCTCCACATGCCGTGTACCAATCTTGCCTTTGTCTTCTCCTATTCACGGCAGCGCGCTTGCATTTGCGCGATGGCGGTGCGTCGTCGGTACCCGCGGACTAGGCGCCACAGCGGCCATATGCTAGTTCATTGAATAGCACCTTACATCCTTACCGTTTCTGAGTGAAATCTGTATGCATGTGGAACACAAAGTCTTGCACGGCATAGCCGGCTAGGGGAGCGCCTCTGAGTCGTGCGTGACAGTATTGGCTGTACGATATCGTTCAAGACGCCACGAGTGCAGCTGCGCCGCCGCGACGGAGTCGCCTGCATGCCGGCTGAGGATGCGCGTGAAGGCGCGCCGATGCTTTCTCGGTGCTACTTTCTCCTTTCTCTCCC

>BPK043/0 clone 2|LinJ23_V3.0290|500 bp UPS + CDS + 500 bp DWS|multidrug resistance protein A

CTCCGCGCCGCATGCGAAGCCTGTTGTCCGCGTAGTTGCGTACGTGTGTATCTGCGCGCGTTGACTCGTGTCCTCTTCGCACCTCCTTCTGCTGGTTTGCCGCTCTCACCCGTAAGCGTGAATCAGTCTTCACGAGGCGCTTAGAGTTTCTTTTCTTTCGTGGAGGTGGTGGTCGCCAAGTGTGTGAGCTGATCGGTGCGCGAGGCGTGCGTGTGCCGAGTGCATCCTGTCCCCGACCCCCGCCTCTGGCTCCTCCGGTGCTACGCCTGAGTCTTCCCTGGCCCAGCCAGCACGGTGCCCGTCGTTGAAAGAGTGTGCCAAGACACACCTCGCCCCCTCGGTTTGTGCGCGGTCTGTCACACTGGTGCTGTTCGACCGCTGTTGTGCGGCTCGTAGGACAGGGTTGTGCGAACTACGCCTCCCTCGTTCTCTTCGCATAGGAAAGACATTGTAGACATCGGTGCATACATTGGGGGGGGGCATCCACAGCGCGCAGGGCAATGGCGACACCAGACTTTGTACGTTTATCACCGACTGACGAGTCCAGCGAGCTCATAATCCATGCTCCCTCGCAGCGGTGCCACACCCGATGTGGCGCAGAAAACGGTCATACCACCAGCATCATTACTGATTTGGAGGCGGTGGACGATGTTGCGCAGGTGCGCTGCCAGCAGCAGGCGCAGCGCGAGTTTGCGGAGCAGCTGGACGAGCTGTGGGGCAGCGAGCCCACCTACACGCCGACGGTAGAGGACCAAGCGAGCTGGCTGCAGCAGCTGCACTACGGATGGATCGGGGACTACATCTACAAGGCTGCGACGGGGAGCATTACCGAGGCGGACCTGCCACCGCCGTCGCGGAGCACACGGACCTACCACACGGGGCGCAAGCTGTCGCGGCAGGCGCATGCCGACATCGACGCAAGCCGGCGGTGGGACGGGTATGTCGGGTGCGAGGTCATGTACGAGGCGGAGGCGGAAACCAGCGGCGCGCTGCGGTGGGTCGGGTATTTGCAGCAGTCGGACTACCCGCGATCGCTGGTAGCTGGGGTGGAGTGGCGTGTGCCGCCGCGGTACCGGCGGCAGGCCACGCCGGGCAGCGCGGCGGGGCTTCACAACGGCGTCGTGCACGGTGAGCGGCTGTTTCGGCCATACGAAGACAACTATCTCTGCTCGTGCGAACCGGTTGAACGGCTTTACCTAAGCTCGACGTGCAGTCTGATGCGTCCCGGGCCGCCGCCGTCGCCGGATCTCCTGTTTACACTGTTCAAGGCGCACTCGTACCACGTGTGGGCGCAGATCCTGCCGAAGCTGCTGGCAGACGTTACCGCGCTGATGCTGCCGGTGTTGTTGGAGTACTTTGTGAAGTATTTGGACGCCGACAACGCGACGTGGGGCTGGGGTCTGGGACTTGTGCTGACACTCTTCCTCACGAACGTGATCCAGAGCTGCGCAGCGCACAAGTACGACCACATCAGCATCCGCAGCGCGGCGCTGTTTGAGACGTCGTCGATGGCGCTGCTGTTCGAGAAGTGCTTCACAGTTTCGCAGCGGTCGCTGCAGCGCCCAGACATGTCCGTGGGTCGCATCATGAACATGGTCGGGAATGACGTTGATAACATCGGTAGTCTCAACTGGTACGTGATGTATTTTTGGAGCGCCCCGCTGCAACTGACACTGTGCATGCTGCTGCTGATAAGGCTGGTCGGGTGGTTCGCGCTGCCCGGCATGGCCGTCTTGTTGGTGACGCTCCCGTTACAAGGTGCCATTTCTAAGCATGTTCAGGAGGTGTCTGAGCGAATGGCGAGCGTGGTGGACCTGCGTATCAAGCGCACGAGCGAGCTGCTCTCCGGTGCCCGAATCGTGAAATTCATGGGGTGGGAACCTGTCTTCCTCGCCCGGATCGAAGACGCGCGCAGTCGCGAGCTGCAGTGTCTGCGCGATGTGCATCTCGCCAATGTCATCTTCATGTTCGTGAACGACGCGACGCCGACGTTGGTCGTCGCTGTAGTCTTTATCTTGTACCATGCGAGTGGGCAGGCGCTGAGACCGGAGATTGTGTTTCCGACGATTGCACTGCTAAACACGATGCGCGTGTCGTTCTTTATGATCCCGCTTATCGTCTCCGCCATCCTGCAGTGTTTGGTGTCCACAAAGCGGGTCACCACTTTTGTGGAGTGCCCTGACACGCGCTCACAGGTGCGGGACATTGCTGGCATCGACGCAGCTGGTGCCGCGGCCATCTTCAAGGATGTGTCCATCCACACCTATCTGCCGGCGAAACTGCCCCAGTGCAAGTCGCGCTTGACGACCATGCAGCGCATTACGCTGTGGTTCCGCCGGCGCGGTGTGCCGGAGGCGGAGTGGTACGATCTGGACGGCCCGGATGCGGGTGCGTCTTCGCCGACGGTGCACCTTCCAACGCTCGACATGGGCAGCGCACAGACTGCCAGTGCGGATGGTGACGGCGCTGCCACTGGCGGCGGGGACGAGGAGGGGGACGTGGAAGAGAGAGTCGCACAGTACTACCAACTTGTGCCGAAGGAGCTGCTGCGGAACGTCAACCTCGTTATCCCTCAGTTCAAGTTGACGATGGTGATTGGCGCGACCGGGAGCGGGAAGTCGACGCTGCTGGGCTCTCTGATGGGCGAGTACGACGTGCAGAGTGGCGAGGTGTGGGCGGAGCGGAGCATCGCGTACGTGCCGCAGCAGGCGTGGATCATGAACGCGACGGTGCGCGACAACATCCTGTTCTTCGACGAGGAGCGCGCCGCAGACTTGCAGGACGTAATCCGTTGCTGCCAGCTGGAGGCGGACCTTGCGCAGCTTGGCGGGGGGCTGGAGACGGAGATCGGGGAAATGGGCGTGAACCTGAGCGGCGGGCAGAAGGCGCGCGTGAGCCTTGCGCGCGCCGTGTACGCGAACCGCGACGTGTACCTGCTGGACGACCCCCTGTCCGCGCTGGACGCGCACGTTGGCCAACGCGTCGTGCAAGACGTCATCCGCGGACGACTGCACGGCAAGACGCGCGTGCTTGCGACGCACCAGATTCATCTGCTGCCGCTCGCGGACTACATTGTGGTGTTGCAGCGCGGCCGCATCGCATTCGCGGGCGATTACGCTGCCTTTGCGGCGACTTCGCTGGAGGAGACGCTGCGCGGTGAGCTAAAGGAGAACAAGGACGCGGAGTCTCGCGGCAGCGATGCGGACGCGGAAGCGGCCAGAGCGGAGACGGCACCTGACATTGCGGAAGCGCATGAGCCGAAAGTTGAGCAGGAGACGAGCCTTGCAGGAGGCGAAGACCCCTTGAGGTCCGATGTCGAGGCCGGTAGGCTGATGACCAGGGAGGAGAAGGCAACCGGTCAGGTACCGTGGTCAACCTACGTGGCGTATCTGAGGTCGTGTGGCGGTCTGGCTGCTTGGGGATTCCTGCTAGCCACCTTTGCGGTGACAGAGAGCGTGACTGCGGCCAACGGCGTGTGGTTGTCGATATGGTCAACAGGCTCGCTTAGGTGTAGCGCGGCCACGTACCTGTACGTCTATCTTTTCATTGTTTTCCTCAACATCTTTAACTCCCCGCTGCGGTGTTTCTTATGCTATTACCTTATGCGAATGGGTAGCCGCAACTTGCATCGCGACCTCCTGGAGTCCATCGGCGTTGCGCGGATGTCCTTCTTCGACACGACGCCCTTGGGACGCGTGCTGAACCGGTTCACAAAGGACATGGGCATCCTTGATAACACGCTGAACGACAGCTACCTTTACCTGCTGCAGTACTTCTTTTCCATGTGCTCCAAGGTTATTATTCTGTCGGCCGCGCAGCCGTTTGTGCTTGTGGCCATTGTGCCGTGTGTGTTCATCTACTACAAGCTGATGCAGATCTACAGCGCGTCGAACCGCGAGACACGCCGCATCAAGAGCATCGCGCACTCGCCCGTGTTCACGCTGCTGGAGGAGTCGCTGCAAGGGCAGCGCACCATCGCGACGTACGGCAAGATGCACCTCGTACTGCAGGAAGCACTGAGGCGGCTCGATGTGGTCTACAGCGCGCTGTACATGCAGAACGTCTCCAACCGCTGGCTCGGTGTCCGCCTCGAGTTTGTGAGCTGTGTTATCACTTTCGTGGTGGCCCTCATTGGTGTGATTGGGAAAATGGAGCGGGCTTCGAGTCAGAGCATCGGTCTCATCTCGCTGTCACTGACGATGGCGATCACGCTGACGGAAACGCTCAATTGGCTGGTGCGGCAGGTTGCGACGGTGGAGGCAAACATGAACAGCGTGGAGCGTGTGATGTATTACACCCACGAAGTGGAGCACGAGTACGTGCCGGAGATGAAGGAGTTAGTGGCACAGCTGGTAGGGAGCGAGTCAGGGACAGCGGCGGAANNNNNNNNNNNNNNNNNNNNNNNNNNNNNNNNNNNNNNNNNNNNNNNNNNNNNNNNNNNNNNNNNNNNNNNNNNNNNNNNNNNNNNNNNNNNNNNNNNNNNNNNNNNNNNNNNNNNNNNNNNNNNNNNNNNNNNNNNNNNNNNNNNNNNNNNNNNNNNNNNNNNNNNNNNNNNNNNNNNNNNNNNNNNNNNNNNNNNNNNNNNNNNNNNNNNNNNNNNNNNNNNNNNNNNNNNNNNNNNNNNNNNNNNNNNNNNNNNNNNNNNNNNNNNNNNNNNNNNNNNNNNNNNNNNNNNNNNNNNNNNNNNNNNNNNNNNNNNNNNNNNNNNNNNNNNNNNNNNNNNNNNNNNNNNNNNNNNNNNNNNNNNNNNNNNNNNNNNNNNNNNNNNNNNNNNNNNNNNNNNNNNNNNNNNNNNNNNNNNNNNNNNNNNNNNNNNNNNNNNNNNNNNNNNNNNNNNNNNNNNNNNNNNNNNNNNNNNNNNNNNNNNNNNNNNNNNNNNNNNNNNNNNNNNNNNNNNNNNNNNNNNNNNNNNNNNNNNNNNNNNNNNNNNNNNNNNNNNNNNNNNNNNNNNNNNNNNNNNNNNNNNNNNNNNNNNNNNNNNNNNNNNNNNNNNNNNNNNNNNNNNNNNNNNNNNNNNNNNNNNNNNNNNNNNNNNNNNNNNNNNNNNNNNNNNNNNNNNNNNNNNNNNNNNNNNNNNNNNNNNNNNNNNNNNNNNNNNNNNNNNNNNNNNNNNNNNNNNNNNNNNNNNNNNNNNNNNNNNNNNNNNNNNNNNNNNNNNNNNNACTGGGCCGCCGTGGTGCCAAAGACTTCGTCGATCTCCTCGGCTGAGTCGCACGAGTCTGTGCAGCAGCCCGTGCGCACCAATGAAAGACTCCGTCGCAACCTGCCGATCTCAAAGCAGAAGCCGCTCGTCGTACGCCACCCCGCATGCACGTGCAGGAGAACGAAGTGCTCCACATGCCGTGTACCAATCTTGCCTTTGTCTTCTCCTATTCACGGCAGCGCGCTTGCATTTGCGCGATGGCGGTGCGTCGTCGGTACCCGCGGACTAGGCGCCACAGCGGCCATATGCTAGTTCATTGAATAGCACCTTACATCCTTACCGTTTCTGAGTGAAATCTGTATGCATGTGGAACACAAAGTCTTGCACGGCATAGCCGGCTAGGGGAGCGCCTCTGAGTCGTGCGTGACAGTATTGGCTGTACGATATCGTTCAAGACGCCACGAGTGCAGCTGCGCCGCCGCGACGGAGTCGCCTGCATGCCGGCTGAGGATGCGCGTGAAGGCGCGCCGATGCTTTCTCGGTGCTACTTTCTCCTTTCTCTCCC

>BPK085/0 clone 8|LinJ23_V3.0290|500 bp UPS + CDS + 500 bp DWS|multidrug resistance protein A

CTCCGCGCCGCATGCGAAGCCTGTTGTCCGCGTAGTTGCGTACGTGTGTATCTGCGCGCGTTGACTCGTGTCCTCTTCGCACCTCCTTCTGCTGGTTTGCCGCTCTCACCCGTAAGCGTGAATCAGTCTTCACGAGGCGCTTAGAGTTTCTTTTCTTTCGTGGAGGTGGTGGTCGCCAAGTGTGTGAGCTGATCGGTGCGCGAGGCGTGCGTGTGCCGAGTGCATCCTGTCCCCGACCCCCGCCTCTGGCTCCTCCGGTGCTACGCCTGAGTCTTCCCTGGCCCAGCCAGCACGGTGCCCGTCGTTGAAAGAGTGTGCCAAGACACACCTCGCCCCCTCGGTTTGTGCGCGGTCTGTCACACTGGTGCTGTTCGACCGCTGTTGTGCGGCTCGTAGGACAGGGTTGTGCGAACTACGCCTCCCTCGTTCTCTTCGCATAGGAAAGACATTGTAGACATCGGTGCATACATTGGGGGGGGGCATCCACAGCGCGCAGGGCAATGGCGACACCAGACTTTGTACGTTTATCACCGACTGACGAGTCCAGCGAGCTCATAATCCATGCTCCCTCGCAGCGGTGCCACACCCGATGTGGCGCAGAAAACGGTCATACCACCAGCATCATTACTGATTTGGAGGCGGTGGACGATGTTGCGCAGGTGCGCTGCCAGCAGCAGGCGCAGCGCGAGTTTGCGGAGCAGCTGGACGAGCTGTGGGGCAGCGAGCCCACCTACACGCCGACGGTAGAGGACCAAGCGAGCTGGCTGCAGCAGCTGCACTACGGATGGATCGGGGACTACATCTACAAGGCTGCGACGGGGAGCATTACCGAGGCGGACCTGCCACCGCCGTCGCGGAGCACACGGACCTACCACACGGGGCGCAAGCTGTCGCGGCAGGCGCATGCCGACATCGACGCAAGCCGGCGGTGGGACGGGTATGTCGGGTGCGAGGTCATGTACGAGGCGGAGGCGGAAACCAGCGGCGCGCTGCGGTGGGTCGGGTATTTGCAGCAGTCGGACTACCCGCGATCGCTGGTAGCTGGGGTGGAGTGGCGTGTGCCGCCGCGGTACCGGCGGCAGGCCACGCCGGGCAGCGCGGCGGGGCTTCACAACGGCGTCGTGCACGGTGAGCGGCTGTTTCGGCCATACGAAGACAACTATCTCTGCTCGTGCGAACCGGTTGAACGGCTTTACCTAAGCTCGACGTGCAGTCTGATGCGTCCCGGGCCGCCGCCGTCGCCGGATCTCCTGTTTACACTGTTCAAGGCGCACTCGTACCACGTGTGGGCGCAGATCCTGCCGAAGCTGCTGGCAGACGTTACCGCGCTGATGCTGCCGGTGTTGTTGGAGTACTTTGTGAAGTATTTGGACGCCGACAACGCGACGTGGGGCTGGGGTCTGGGACTTGTGCTGACACTCTTCCTCACGAACGTGATCCAGAGCTGCGCAGCGCACAAGTACGACCACATCAGCATCCGCAGCGCGGCGCTGTTTGAGACGTCGTCGATGGCGCTGCTGTTCGAGAAGTGCTTCACAGTTTCGCAGCGGTCGCTGCAGCGCCCAGACATGTCCGTGGGTCGCATCATGAACATGGTCGGGAATGACGTTGATAACATCGGTAGTCTCAACTGGTACGTGATGTATTTTTGGAGCGCCCCGCTGCAACTGACACTGTGCATGCTGCTGCTGATAAGGCTGGTCGGGTGGTTCGCGCTGCCCGGCATGGCCGTCTTGTTGGTGACGCTCCCGTTACAAGGTGCCATTTCTAAGCATGTTCAGGAGGTGTCTGAGCGAATGGCGAGCGTGGTGGACCTGCGTATCAAGCGCACGAGCGAGCTGCTCTCCGGTGCCCGAATCGTGAAATTCATGGGGTGGGAACCTGTCTTCCTCGCCCGGATCGAAGACGCGCGCAGTCGCGAGCTGCAGTGTCTGCGCGATGTGCATCTCGCCAATGTCATCTTCATGTTCGTGAACGACGCGACGCCGACGTTGGTCGTCGCTGTAGTCTTTATCTTGTACCATGCGAGTGGGCAGGCGCTGAGACCGGAGATTGTGTTTCCGACGATTGCACTGCTAAACACGATGCGCGTGTCGTTCTTTATGATCCCGCTTATCGTCTCCGCCATCCTGCAGTGTTTGGTGTCCACAAAGCGGGTCACCACTTTTGTGGAGTGCCCTGACACGCGCTCACAGGTGCGGGACATTGCTGGCATCGACGCAGCTGGTGCCGCGGCCATCTTCAAGGATGTGTCCATCCACACCTATCTGCCGGCGAAACTGCCCCAGTGCAAGTCGCGCTTGACGACCATGCAGCGCATTACGCTGTGGTTCCGCCGGCGCGGTGTGCCGGAGGCGGAGTGGTACGATCTGGACGGCCCGGATGCGGGTGCGTCTTCGCCGACGGTGCACCTTCCAACGCTCGACATGGGCAGCGCACAGACTGCCAGTGCGGATGGTGACGGCGCTGCCACTGGCGGCGGGGACGAGGAGGGGGACGTGGAAGAGAGAGTCGCACAGTACTACCAACTTGTGCCGAAGGAGCTGCTGCGGAACGTCAACCTCGTTATCCCTCAGTTCAAGTTGACGATGGTGATTGGCGCGACCGGGAGCGGGAAGTCGACGCTGCTGGGCTCTCTGATGGGCGAGTACGACGTGCAGAGTGGCGAGGTGTGGGCGGAGCGGAGCATCGCGTACGTGCCGCAGCAGGCGTGGATCATGAACGCGACGGTGCGCGACAACATCCTGTTCTTCGACGAGGAGCGCGCCGCAGACTTGCAGGACGTAATCCGTTGCTGCCAGCTGGAGGCGGACCTTGCGCAGCTTGGCGGGGGGCTGGAGACGGAGATCGGGGAAATGGGCGTGAACCTGAGCGGCGGGCAGAAGGCGCGCGTGAGCCTTGCGCGCGCCGTGTACGCGAACCGCGACGTGTACCTGCTGGACGACCCCCTGTCCGCGCTGGACGCGCACGTTGGCCAACGCGTCGTGCAAGACGTCATCCGCGGACGACTGCACGGCAAGACGCGCGTGCTTGCGACGCACCAGATTCATCTGCTGCCGCTCGCGGACTACATTGTGGTGTTGCAGCGCGGCCGCATCGCATTCGCGGGCGATTACGCTGCCTTTGCGGCGACTTCGCTGGAGGAGACGCTGCGCGGTGAGCTAAAGGAGAACAAGGACGCGGAGTCTCGCGGCAGCGATGCGGACGCGGAAGCGGCCAGAGCGGAGACGGCACCTGACATTGCGGAAGCGCATGAGCCGAAAGTTGAGCAGGAGACGAGCCTTGCAGGAGGCGAAGACCCCTTGAGGTCCGATGTCGAGGCCGGTAGGCTGATGACCAGGGAGGAGAAGGCAACCGGTCAGGTACCGTGGTCAACCTACGTGGCGTATCTGAGGTCGTGTGGCGGTCTGGCTGCTTGGGGATTCCTGCTAGCCACCTTTGCGGTGACAGAGAGCGTGACTGCGGCCAACGGCGTGTGGTTGTCGATATGGTCAACAGGCTCGCTTAGGTGTAGCGCGGCCACGTACCTGTACGTCTATCTTTTCATTGTTTTCCTCAACATCTTTAACTCCCCGCTGCGGTGTTTCTTATGCTATTACCTTATGCGAATGGGTAGCCGCAACTTGCATCGCGACCTCCTGGAGTCCATCGGCGTTGCGCGGATGTCCTTCTTCGACACGACGCCCTTGGGACGCGTGCTGAACCGGTTCACAAAGGACATGGGCATCCTTGATAACACGCTGAACGACAGCTACCTTTACCTGCTGCAGTACTTCTTTTCCATGTGCTCCAAGGTTATTATTCTGTCGGCCGCGCAGCCGTTTGTGCTTGTGGCCATTGTGCCGTGTGTGTTCATCTACTACAAGCTGATGCAGATCTACAGCGCGTCGAACCGCGAGACACGCCGCATCAAGAGCATCGCGCACTCGCCCGTGTTCACGCTGCTGGAGGAGTCGCTGCAAGGGCAGCGCACCATCGCGACGTACGGCAAGATGCACCTCGTACTGCAGGAAGCACTGAGGCGGCTCGATGTGGTCTACAGCGCGCTGTACATGCAGAACGTCTCCAACCGCTGGCTCGGTGTCCGCCTCGAGTTTGTGAGCTGTGTTATCACTTTCGTGGTGGCCCTCATTGGTGTGATTGGGAAAATGGAGCGGGCTTCGAGTCAGAGCATCGGTCTCATCTCGCTGTCACTGACGATGGCGATCACGCTGACGGAAACGCTCAATTGGCTGGTGCGGCAGGTTGCGACGGTGGAGGCAAACATGAACAGCGTGGAGCGTGTGATGTATTACACCCACGAAGTGGAGCACGAGTACGTGCCGGAGATGAAGGAGTTAGTGGCACAGCTGGTAGGGAGCGAGTCAGGGACAGCGGCGGAANNNNNNNNNNNNNNNNNNNNNNNNNNNNNNNNNNNNNNNNNNNNNNNNNNNNNNNNNNNNNNNNNNNNNNNNNNNNNNNNNNNNNNNNNNNNNNNNNNNNNNNNNNNNNNNNNNNNNNNNNNNNNNNNNNNNNNNNNNNNNNNNNNNNNNNNNNNNNNNNNNNNNNNNNNNNNNNNNNNNNNNNNNNNNNNNNNNNNNNNNNNNNNNNNNNNNNNNNNNNNNNNNNNNNNNNNNNNNNNNNNNNNNNNNNNNNNNNNNNNNNNNNNNNNNNNNNNNNNNNNNNNNNNNNNNNNNNNNNNNNNNNNNNNNNNNNNNNNNNNNNNNNNNNNNNNNNNNNNNNNNNNNNNNNNNNNNNNNNNNNNNNNNNNNNNNNNNNNNNNNNNNNNNNNNNNNNNNNNNNNNNNNNNNNNNNNNNNNNNNNNNNNNNNNNNNNNNNNNNNNNNNNNNNNNNNNNNNNNNNNNNNNNNNNNNNNNNNNNNNNNNNNNNNNNNNNNNNNNNNNNNNNNNNNNNNNNNNNNNNNNNNNNNNNNNNNNNNNNNNNNNNNNNNNNNNNNNNNNNNNNNNNNNNNNNNNNNNNNNNNNNNNNNNNNNNNNNNNNNNNNNNNNNNNNNNNNNNNNNNNNNNNNNNNNNNNNNNNNNNNNNNNNNNNNNNNNNNNNNNNNNNNNNNNNNNNNNNNNNNNNNNNNNNNNNNNNNNNNNNNNNNNNNNNNNNNNNNNNNNNNNNNNNNNNNNNNNNNNNNNNNNNNNNNNNNNNNNNNNNNNNNNNNNNNNNNNNNNNNNACTGGGCCGCCGTGGTGCCAAAGACTTCGTCGATCTCCTCGGCTGAGTCGCACGAGTCTGTGCAGCAGCCCGTGCGCACCAATGAAAGACTCCGTCGCAACCTGCCGATCTCAAAGCAGAAGCCGCTCGTCGTACGCCACCCCGCATGCACGTGCAGGAGAACGAAGTGCTCCACATGCCGTGTACCAATCTTGCCTTTGTCTTCTCCTATTCACGGCAGCGCGCTTGCATTTGCGCGATGGCGGTGCGTCGTCGGTACCCGCGGACTAGGCGCCACAGCGGCCATATGCTAGTTCATTGAATAGCACCTTACATCCTTACCGTTTCTGAGTGAAATCTGTATGCATGTGGAACACAAAGTCTTGCACGGCATAGCCGGCTAGGGGAGCGCCTCTGAGTCGTGCGTGACAGTATTGGCTGTACGATATCGTTCAAGACGCCACGAGTGCAGCTGCGCCGCCGCGACGGAGTCGCCTGCATGCCGGCTGAGGATGCGCGTGAAGGCGCGCCGATGCTTTCTCGGTGCTACTTTCTCCTTTCTCTCCC

>BPK087/0 clone 11|LinJ23_V3.0290|500 bp UPS + CDS + 500 bp DWS|multidrug resistance protein A

CTCCGCGCCGCATGCGAAGCCTGTTGTCCGCGTAGTTGCGTACGTGTGTATCTGCGCGCGTTGACTCGTGTCCTCTTCGCACCTCCTTCTGCTGGTTTGCCGCTCTCACCCGTAAGCGTGAATCAGTCTTCACGAGGCGCTTAGAGTTTCTTTTCTTTCGTGGAGGTGGTGGTCGCCAAGTGTGTGAGCTGATCGGTGCGCGAGGCGTGCGTGTGCCGAGTGCATCCTGTCCCCGACCCCCGCCTCTGGCTCCTCCGGTGCTACGCCTGAGTCTTCCCTGGCCCAGCCAGCACGGTGCCCGTCGTTGAAAGAGTGTGCCAAGACACACCTCGCCCCCTCGGTTTGTGCGCGGTCTGTCACACTGGTGCTGTTCGACCGCTGTTGTGCGGCTCGTAGGACAGGGTTGTGCGAACTACGCCTCCCTCGTTCTCTTCGCATAGGAAAGACATTGTAGACATCGGTGCATACATTGGGGGGGGGCATCCACAGCGCGCAGGGCAATGGCGACACCAGACTTTGTACGTTTATCACCGACTGACGAGTCCAGCGAGCTCATAATCCATGCTCCCTCGCAGCGGTGCCACACCCGATGTGGCGCAGAAAACGGTCATACCACCAGCATCATTACTGATTTGGAGGCGGTGGACGATGTTGCGCAGGTGCGCTGCCAGCAGCAGGCGCAGCGCGAGTTTGCGGAGCAGCTGGACGAGCTGTGGGGCAGCGAGCCCACCTACACGCCGACGGTAGAGGACCAAGCGAGCTGGCTGCAGCAGCTGCACTACGGATGGATCGGGGACTACATCTACAAGGCTGCGACGGGGAGCATTACCGAGGCGGACCTGCCACCGCCGTCGCGGAGCACACGGACCTACCACACGGGGCGCAAGCTGTCGCGGCAGGCGCATGCCGACATCGACGCAAGCCGGCGGTGGGACGGGTATGTCGGGTGCGAGGTCATGTACGAGGCGGAGGCGGAAACCAGCGGCGCGCTGCGGTGGGTCGGGTATTTGCAGCAGTCGGACTACCCGCGATCGCTGGTAGCTGGGGTGGAGTGGCGTGTGCCGCCGCGGTACCGGCGGCAGGCCACGCCGGGCAGCGCGGCGGGGCTTCACAACGGCGTCGTGCACGGTGAGCGGCTGTTTCGGCCATACGAAGACAACTATCTCTGCTCGTGCGAACCGGTTGAACGGCTTTACCTAAGCTCGACGTGCAGTCTGATGCGTCCCGGGCCGCCGCCGTCGCCGGATCTCCTGTTTACACTGTTCAAGGCGCACTCGTACCACGTGTGGGCGCAGATCCTGCCGAAGCTGCTGGCAGACGTTACCGCGCTGATGCTGCCGGTGTTGTTGGAGTACTTTGTGAAGTATTTGGACGCCGACAACGCGACGTGGGGCTGGGGTCTGGGACTTGTGCTGACACTCTTCCTCACGAACGTGATCCAGAGCTGCGCAGCGCACAAGTACGACCACATCAGCATCCGCAGCGCGGCGCTGTTTGAGACGTCGTCGATGGCGCTGCTGTTCGAGAAGTGCTTCACAGTTTCGCAGCGGTCGCTGCAGCGCCCAGACATGTCCGTGGGTCGCATCATGAACATGGTCGGGAATGACGTTGATAACATCGGTAGTCTCAACTGGTACGTGATGTATTTTTGGAGCGCCCCGCTGCAACTGACACTGTGCATGCTGCTGCTGATAAGGCTGGTCGGGTGGTTCGCGCTGCCCGGCATGGCCGTCTTGTTGGTGACGCTCCCGTTACAAGGTGCCATTTCTAAGCATGTTCAGGAGGTGTCTGAGCGAATGGCGAGCGTGGTGGACCTGCGTATCAAGCGCACGAGCGAGCTGCTCTCCGGTGCCCGAATCGTGAAATTCATGGGGTGGGAACCTGTCTTCCTCGCCCGGATCGAAGACGCGCGCAGTCGCGAGCTGCAGTGTCTGCGCGATGTGCATCTCGCCAATGTCATCTTCATGTTCGTGAACGACGCGACGCCGACGTTGGTCGTCGCTGTAGTCTTTATCTTGTACCATGCGAGTGGGCAGGCGCTGAGACCGGAGATTGTGTTTCCGACGATTGCACTGCTAAACACGATGCGCGTGTCGTTCTTTATGATCCCGCTTATCGTCTCCGCCATCCTGCAGTGTTTGGTGTCCACAAAGCGGGTCACCACTTTTGTGGAGTGCCCTGACACGCGCTCACAGGTGCGGGACATTGCTGGCATCGACGCAGCTGGTGCCGCGGCCATCTTCAAGGATGTGTCCATCCACACCTATCTGCCGGCGAAACTGCCCCAGTGCAAGTCGCGCTTGACGACCATGCAGCGCATTACGCTGTGGTTCCGCCGGCGCGGTGTGCCGGAGGCGGAGTGGTACGATCTGGACGGCCCGGATGCGGGTGCGTCTTCGCCGACGGTGCACCTTCCAACGCTCGACATGGGCAGCGCACAGACTGCCAGTGCGGATGGTGACGGCGCTGCCACTGGCGGCGGGGACGAGGAGGGGGACGTGGAAGAGAGAGTCGCACAGTACTACCAACTTGTGCCGAAGGAGCTGCTGCGGAACGTCAACCTCGTTATCCCTCAGTTCAAGTTGACGATGGTGATTGGCGCGACCGGGAGCGGGAAGTCGACGCTGCTGGGCTCTCTGATGGGCGAGTACGACGTGCAGAGTGGCGAGGTGTGGGCGGAGCGGAGCATCGCGTACGTGCCGCAGCAGGCGTGGATCATGAACGCGACGGTGCGCGACAACATCCTGTTCTTCGACGAGGAGCGCGCCGCAGACTTGCAGGACGTAATCCGTTGCTGCCAGCTGGAGGCGGACCTTGCGCAGCTTGGCGGGGGGCTGGAGACGGAGATCGGGGAAATGGGCGTGAACCTGAGCGGCGGGCAGAAGGCGCGCGTGAGCCTTGCGCGCGCCGTGTACGCGAACCGCGACGTGTACCTGCTGGACGACCCCCTGTCCGCGCTGGACGCGCACGTTGGCCAACGCGTCGTGCAAGACGTCATCCGCGGACGACTGCACGGCAAGACGCGCGTGCTTGCGACGCACCAGATTCATCTGCTGCCGCTCGCGGACTACATTGTGGTGTTGCAGCGCGGCCGCATCGCATTCGCGGGCGATTACGCTGCCTTTGCGGCGACTTCGCTGGAGGAGACGCTGCGCGGTGAGCTAAAGGAGAACAAGGACGCGGAGTCTCGCGGCAGCGATGCGGACGCGGAAGCGGCCAGAGCGGAGACGGCACCTGACATTGCGGAAGCGCATGAGCCGAAAGTTGAGCAGGAGACGAGCCTTGCAGGAGGCGAAGACCCCTTGAGGTCCGATGTCGAGGCCGGTAGGCTGATGACCAGGGAGGAGAAGGCAACCGGTCAGGTACCGTGGTCAACCTACGTGGCGTATCTGAGGTCGTGTGGCGGTCTGGCTGCTTGGGGATTCCTGCTAGCCACCTTTGCGGTGACAGAGAGCGTGACTGCGGCCAACGGCGTGTGGTTGTCGATATGGTCAACAGGCTCGCTTAGGTGTAGCGCGGCCACGTACCTGTACGTCTATCTTTTCATTGTTTTCCTCAACATCTTTAACTCCCCGCTGCGGTGTTTCTTATGCTATTACCTTATGCGAATGGGTAGCCGCAACTTGCATCGCGACCTCCTGGAGTCCATCGGCGTTGCGCGGATGTCCTTCTTCGACACGACGCCCTTGGGACGCGTGCTGAACCGGTTCACAAAGGACATGGGCATCCTTGATAACACGCTGAACGACAGCTACCTTTACCTGCTGCAGTACTTCTTTTCCATGTGCTCCAAGGTTATTATTCTGTCGGCCGCGCAGCCGTTTGTGCTTGTGGCCATTGTGCCGTGTGTGTTCATCTACTACAAGCTGATGCAGATCTACAGCGCGTCGAACCGCGAGACACGCCGCATCAAGAGCATCGCGCACTCGCCCGTGTTCACGCTGCTGGAGGAGTCGCTGCAAGGGCAGCGCACCATCGCGACGTACGGCAAGATGCACCTCGTACTGCAGGAAGCACTGAGGCGGCTCGATGTGGTCTACAGCGCGCTGTACATGCAGAACGTCTCCAACCGCTGGCTCGGTGTCCGCCTCGAGTTTGTGAGCTGTGTTATCACTTTCGTGGTGGCCCTCATTGGTGTGATTGGGAAAATGGAGCGGGCTTCGAGTCAGAGCATCGGTCTCATCTCGCTGTCACTGACGATGGCGATCACGCTGACGGAAACGCTCAATTGGCTGGTGCGGCAGGTTGCGACGGTGGAGGCAAACATGAACAGCGTGGAGCGTGTGATGTATTACACCCACGAAGTGGAGCACGAGTACGTGCCGGAGATGAAGGAGTTAGTGGCACAGCTGGTAGGGAGCGAGTCAGGGACAGCGGCGGAANNNNNNNNNNNNNNNNNNNNNNNNNNNNNNNNNNNNNNNNNNNNNNNNNNNNNNNNNNNNNNNNNNNNNNNNNNNNNNNNNNNNNNNNNNNNNNNNNNNNNNNNNNNNNNNNNNNNNNNNNNNNNNNNNNNNNNNNNNNNNNNNNNNNNNNNNNNNNNNNNNNNNNNNNNNNNNNNNNNNNNNNNNNNNNNNNNNNNNNNNNNNNNNNNNNNNNNNNNNNNNNNNNNNNNNNNNNNNNNNNNNNNNNNNNNNNNNNNNNNNNNNNNNNNNNNNNNNNNNNNNNNNNNNNNNNNNNNNNNNNNNNNNNNNNNNNNNNNNNNNNNNNNNNNNNNNNNNNNNNNNNNNNNNNNNNNNNNNNNNNNNNNNNNNNNNNNNNNNNNNNNNNNNNNNNNNNNNNNNNNNNNNNNNNNNNNNNNNNNNNNNNNNNNNNNNNNNNNNNNNNNNNNNNNNNNNNNNNNNNNNNNNNNNNNNNNNNNNNNNNNNNNNNNNNNNNNNNNNNNNNNNNNNNNNNNNNNNNNNNNNNNNNNNNNNNNNNNNNNNNNNNNNNNNNNNNNNNNNNNNNNNNNNNNNNNNNNNNNNNNNNNNNNNNNNNNNNNNNNNNNNNNNNNNNNNNNNNNNNNNNNNNNNNNNNNNNNNNNNNNNNNNNNNNNNNNNNNNNNNNNNNNNNNNNNNNNNNNNNNNNNNNNNNNNNNNNNNNNNNNNNNNNNNNNNNNNNNNNNNNNNNNNNNNNNNNNNNNNNNNNNNNNNNNNNNNNNNNNNNNNNNNNNNNNNNNNNNNNNNACTGGGCCGCCGTGGTGCCAAAGACTTCGTCGATCTCCTCGGCTGAGTCGCACGAGTCTGTGCAGCAGCCCGTGCGCACCAATGAAAGACTCCGTCGCAACCTGCCGATCTCAAAGCAGAAGCCGCTCGTCGTACGCCACCCCGCATGCACGTGCAGGAGAACGAAGTGCTCCACATGCCGTGTACCAATCTTGCCTTTGTCTTCTCCTATTCACGGCAGCGCGCTTGCATTTGCGCGATGGCGGTGCGTCGTCGGTACCCGCGGACTAGGCGCCACAGCGGCCATATGCTAGTTCATTGAATAGCACCTTACATCCTTACCGTTTCTGAGTGAAATCTGTATGCATGTGGAACACAAAGTCTTGCACGGCATAGCCGGCTAGGGGAGCGCCTCTGAGTCGTGCGTGACAGTATTGGCTGTACGATATCGTTCAAGACGCCACGAGTGCAGCTGCGCCGCCGCGACGGAGTCGCCTGCATGCCGGCTGAGGATGCGCGTGAAGGCGCGCCGATGCTTTCTCGGTGCTACTTTCTCCTTTCTCTCCC

>BPK178/0 clone 3|LinJ23_V3.0290|500 bp UPS + CDS + 500 bp DWS|multidrug resistance protein A

CTCCGCGCCGCATGCGAAGCCTGTTGTCCGCGTAGTTGCGTACGTGTGTATCTGCGCGCGTTGACTCGTGTCCTCTTCGCACCTCCTTCTGCTGGTTTGCCGCTCTCACCCGTAAGCGTGAATCAGTCTTCACGAGGCGCTTAGAGTTTCTTTTCTTTCGTGGAGGTGGTGGTCGCCAAGTGTGTGAGCTGATCGGTGCGCGAGGCGTGCGTGTGCCGAGTGCATCCTGTCCCCGACCCCCGCCTCTGGCTCCTCCGGTGCTACGCCTGAGTCTTCCCTGGCCCAGCCAGCACGGTGCCCGTCGTTGAAAGAGTGTGCCAAGACACACCTCGCCCCCTCGGTTTGTGCGCGGTCTGTCACACTGGTGCTGTTCGACCGCTGTTGTGCGGCTCGTAGGACAGGGTTGTGCGAACTACGCCTCCCTCGTTCTCTTCGCATAGGAAAGACATTGTAGACATCGGTGCATACATTGGGGGGGGGCATCCACAGCGCGCAGGGCAATGGCGACACCAGACTTTGTACGTTTATCACCGACTGACGAGTCCAGCGAGCTCATAATCCATGCTCCCTCGCAGCGGTGCCACACCCGATGTGGCGCAGAAAACGGTCATACCACCAGCATCATTACTGATTTGGAGGCGGTGGACGATGTTGCGCAGGTGCGCTGCCAGCAGCAGGCGCAGCGCGAGTTTGCGGAGCAGCTGGACGAGCTGTGGGGCAGCGAGCCCACCTACACGCCGACGGTAGAGGACCAAGCGAGCTGGCTGCAGCAGCTGCACTACGGATGGATCGGGGACTACATCTACAAGGCTGCGACGGGGAGCATTACCGAGGCGGACCTGCCACCGCCGTCGCGGAGCACACGGACCTACCACACGGGGCGCAAGCTGTCGCGGCAGGCGCATGCCGACATCGACGCAAGCCGGCGGTGGGACGGGTATGTCGGGTGCGAGGTCATGTACGAGGCGGAGGCGGAAACCAGCGGCGCGCTGCGGTGGGTCGGGTATTTGCAGCAGTCGGACTACCCGCGATCGCTGGTAGCTGGGGTGGAGTGGCGTGTGCCGCCGCGGTACCGGCGGCAGGCCACGCCGGGCAGCGCGGCGGGGCTTCACAACGGCGTCGTGCACGGTGAGCGGCTGTTTCGGCCATACGAAGACAACTATCTCTGCTCGTGCGAACCGGTTGAACGGCTTTACCTAAGCTCGACGTGCAGTCTGATGCGTCCCGGGCCGCCGCCGTCGCCGGATCTCCTGTTTACACTGTTCAAGGCGCACTCGTACCACGTGTGGGCGCAGATCCTGCCGAAGCTGCTGGCAGACGTTACCGCGCTGATGCTGCCGGTGTTGTTGGAGTACTTTGTGAAGTATTTGGACGCCGACAACGCGACGTGGGGCTGGGGTCTGGGACTTGTGCTGACACTCTTCCTCACGAACGTGATCCAGAGCTGCGCAGCGCACAAGTACGACCACATCAGCATCCGCAGCGCGGCGCTGTTTGAGACGTCGTCGATGGCGCTGCTGTTCGAGAAGTGCTTCACAGTTTCGCAGCGGTCGCTGCAGCGCCCAGACATGTCCGTGGGTCGCATCATGAACATGGTCGGGAATGACGTTGATAACATCGGTAGTCTCAACTGGTACGTGATGTATTTTTGGAGCGCCCCGCTGCAACTGACACTGTGCATGCTGCTGCTGATAAGGCTGGTCGGGTGGTTCGCGCTGCCCGGCATGGCCGTCTTGTTGGTGACGCTCCCGTTACAAGGTGCCATTTCTAAGCATGTTCAGGAGGTGTCTGAGCGAATGGCGAGCGTGGTGGACCTGCGTATCAAGCGCACGAGCGAGCTGCTCTCCGGTGCCCGAATCGTGAAATTCATGGGGTGGGAACCTGTCTTCCTCGCCCGGATCGAAGACGCGCGCAGTCGCGAGCTGCAGTGTCTGCGCGATGTGCATCTCGCCAATGTCATCTTCATGTTCGTGAACGACGCGACGCCGACGTTGGTCGTCGCTGTAGTCTTTATCTTGTACCATGCGAGTGGGCAGGCGCTGAGACCGGAGATTGTGTTTCCGACGATTGCACTGCTAAACACGATGCGCGTGTCGTTCTTTATGATCCCGCTTATCGTCTCCGCCATCCTGCAGTGTTTGGTGTCCACAAAGCGGGTCACCACTTTTGTGGAGTGCCCTGACACGCGCTCACAGGTGCGGGACATTGCTGGCATCGACGCAGCTGGTGCCGCGGCCATCTTCAAGGATGTGTCCATCCACACCTATCTGCCGGCGAAACTGCCCCAGTGCAAGTCGCGCTTGACGACCATGCAGCGCATTACGCTGTGGTTCCGCCGGCGCGGTGTGCCGGAGGCGGAGTGGTACGATCTGGACGGCCCGGATGCGGGTGCGTCTTCGCCGACGGTGCACCTTCCAACGCTCGACATGGGCAGCGCACAGACTGCCAGTGCGGATGGTGACGGCGCTGCCACTGGCGGCGGGGACGAGGAGGGGGACGTGGAAGAGAGAGTCGCACAGTACTACCAACTTGTGCCGAAGGAGCTGCTGCGGAACGTCAACCTCGTTATCCCTCAGTTCAAGTTGACGATGGTGATTGGCGCGACCGGGAGCGGGAAGTCGACGCTGCTGGGCTCTCTGATGGGCGAGTACGACGTGCAGAGTGGCGAGGTGTGGGCGGAGCGGAGCATCGCGTACGTGCCGCAGCAGGCGTGGATCATGAACGCGACGGTGCGCGACAACATCCTGTTCTTCGACGAGGAGCGCGCCGCAGACTTGCAGGACGTAATCCGTTGCTGCCAGCTGGAGGCGGACCTTGCGCAGCTTGGCGGGGGGCTGGAGACGGAGATCGGGGAAATGGGCGTGAACCTGAGCGGCGGGCAGAAGGCGCGCGTGAGCCTTGCGCGCGCCGTGTACGCGAACCGCGACGTGTACCTGCTGGACGACCCCCTGTCCGCGCTGGACGCGCACGTTGGCCAACGCGTCGTGCAAGACGTCATCCGCGGACGACTGCACGGCAAGACGCGCGTGCTTGCGACGCACCAGATTCATCTGCTGCCGCTCGCGGACTACATTGTGGTGTTGCAGCGCGGCCGCATCGCATTCGCGGGCGATTACGCTGCCTTTGCGGCGACTTCGCTGGAGGAGACGCTGCGCGGTGAGCTAAAGGAGAACAAGGACGCGGAGTCTCGCGGCAGCGATGCGGACGCGGAAGCGGCCAGAGCGGAGACGGCACCTGACATTGCGGAAGCGCATGAGCCGAAAGTTGAGCAGGAGACGAGCCTTGCAGGAGGCGAAGACCCCTTGAGGTCCGATGTCGAGGCCGGTAGGCTGATGACCAGGGAGGAGAAGGCAACCGGTCAGGTACCGTGGTCAACCTACGTGGCGTATCTGAGGTCGTGTGGCGGTCTGGCTGCTTGGGGATTCCTGCTAGCCACCTTTGCGGTGACAGAGAGCGTGACTGCGGCCAACGGCGTGTGGTTGTCGATATGGTCAACAGGCTCGCTTAGGTGTAGCGCGGCCACGTACCTGTACGTCTATCTTTTCATTGTTTTCCTCAACATCTTTAACTCCCCGCTGCGGTGTTTCTTATGCTATTACCTTATGCGAATGGGTAGCCGCAACTTGCATCGCGACCTCCTGGAGTCCATCGGCGTTGCGCGGATGTCCTTCTTCGACACGACGCCCTTGGGACGCGTGCTGAACCGGTTCACAAAGGACATGGGCATCCTTGATAACACGCTGAACGACAGCTACCTTTACCTGCTGCAGTACTTCTTTTCCATGTGCTCCAAGGTTATTATTCTGTCGGCCGCGCAGCCGTTTGTGCTTGTGGCCATTGTGCCGTGTGTGTTCATCTACTACAAGCTGATGCAGATCTACAGCGCGTCGAACCGCGAGACACGCCGCATCAAGAGCATCGCGCACTCGCCCGTGTTCACGCTGCTGGAGGAGTCGCTGCAAGGGCAGCGCACCATCGCGACGTACGGCAAGATGCACCTCGTACTGCAGGAAGCACTGAGGCGGCTCGATGTGGTCTACAGCGCGCTGTACATGCAGAACGTCTCCAACCGCTGGCTCGGTGTCCGCCTCGAGTTTGTGAGCTGTGTTATCACTTTCGTGGTGGCCCTCATTGGTGTGATTGGGAAAATGGAGCGGGCTTCGAGTCAGAGCATCGGTCTCATCTCGCTGTCACTGACGATGGCGATCACGCTGACGGAAACGCTCAATTGGCTGGTGCGGCAGGTTGCGACGGTGGAGGCAAACATGAACAGCGTGGAGCGTGTGATGTATTACACCCACGAAGTGGAGCACGAGTACGTGCCGGAGATGAAGGAGTTAGTGGCACAGCTGGTAGGGAGCGAGTCAGGGACAGCGGCGGAANNNNNNNNNNNNNNNNNNNNNNNNNNNNNNNNNNNNNNNNNNNNNNNNNNNNNNNNNNNNNNNNNNNNNNNNNNNNNNNNNNNNNNNNNNNNNNNNNNNNNNNNNNNNNNNNNNNNNNNNNNNNNNNNNNNNNNNNNNNNNNNNNNNNNNNNNNNNNNNNNNNNNNNNNNNNNNNNNNNNNNNNNNNNNNNNNNNNNNNNNNNNNNNNNNNNNNNNNNNNNNNNNNNNNNNNNNNNNNNNNNNNNNNNNNNNNNNNNNNNNNNNNNNNNNNNNNNNNNNNNNNNNNNNNNNNNNNNNNNNNNNNNNNNNNNNNNNNNNNNNNNNNNNNNNNNNNNNNNNNNNNNNNNNNNNNNNNNNNNNNNNNNNNNNNNNNNNNNNNNNNNNNNNNNNNNNNNNNNNNNNNNNNNNNNNNNNNNNNNNNNNNNNNNNNNNNNNNNNNNNNNNNNNNNNNNNNNNNNNNNNNNNNNNNNNNNNNNNNNNNNNNNNNNNNNNNNNNNNNNNNNNNNNNNNNNNNNNNNNNNNNNNNNNNNNNNNNNNNNNNNNNNNNNNNNNNNNNNNNNNNNNNNNNNNNNNNNNNNNNNNNNNNNNNNNNNNNNNNNNNNNNNNNNNNNNNNNNNNNNNNNNNNNNNNNNNNNNNNNNNNNNNNNNNNNNNNNNNNNNNNNNNNNNNNNNNNNNNNNNNNNNNNNNNNNNNNNNNNNNNNNNNNNNNNNNNNNNNNNNNNNNNNNNNNNNNNNNNNNNNNNNNNNNNNNNNNNNNNNNNNNNNNNNNNNNNNNNNNNNACTGGGCCGCCGTGGTGCCAAAGACTTCGTCGATCTCCTCGGCTGAGTCGCACGAGTCTGTGCAGCAGCCCGTGCGCACCAATGAAAGACTCCGTCGCAACCTGCCGATCTCAAAGCAGAAGCCGCTCGTCGTACGCCACCCCGCATGCACGTGCAGGAGAACGAAGTGCTCCACATGCCGTGTACCAATCTTGCCTTTGTCTTCTCCTATTCACGGCAGCGCGCTTGCATTTGCGCGATGGCGGTGCGTCGTCGGTACCCGCGGACTAGGCGCCACAGCGGCCATATGCTAGTTCATTGAATAGCACCTTACATCCTTACCGTTTCTGAGTGAAATCTGTATGCATGTGGAACACAAAGTCTTGCACGGCATAGCCGGCTAGGGGAGCGCCTCTGAGTCGTGCGTGACAGTATTGGCTGTACGATATCGTTCAAGACGCCACGAGTGCAGCTGCGCCGCCGCGACGGAGTCGCCTGCATGCCGGCTGAGGATGCGCGTGAAGGCGCGCCGATGCTTTCTCGGTGCTACTTTCTCCTTTCTCTCCC

>BPK190/0 clone 3|LinJ23_V3.0290|500 bp UPS + CDS + 500 bp DWS|multidrug resistance protein A

CTCCGCGCCGCATGCGAAGCCTGTTGTCCGCGTAGTTGCGTACGTGTGTATCTGCGCGCGTTGACTCGTGTCCTCTTCGCACCTCCTTCTGCTGGTTTGCCGCTCTCACCCGTAAGCGTGAATCAGTCTTCACGAGGCGCTTAGAGTTTCTTTTCTTTCGTGGAGGTGGTGGTCGCCAAGTGTGTGAGCTGATCGGTGCGCGAGGCGTGCGTGTGCCGAGTGCATCCTGTCCCCGACCCCCGCCTCTGGCTCCTCCGGTGCTACGCCTGAGTCTTCCCTGGCCCAGCCAGCACGGTGCCCGTCGTTGAAAGAGTGTGCCAAGACACACCTCGCCCCCTCGGTTTGTGCGCGGTCTGTCACACTGGTGCTGTTCGACCGCTGTTGTGCGGCTCGTAGGACAGGGTTGTGCGAACTACGCCTCCCTCGTTCTCTTCGCATAGGAAAGACATTGTAGACATCGGTGCATACATTGGGGGGGGGCATCCACAGCGCGCAGGGCAATGGCGACACCAGACTTTGTACGTTTATCACCGACTGACGAGTCCAGCGAGCTCATAATCCATGCTCCCTCGCAGCGGTGCCACACCCGATGTGGCGCAGAAAACGGTCATACCACCAGCATCATTACTGATTTGGAGGCGGTGGACGATGTTGCGCAGGTGCGCTGCCAGCAGCAGGCGCAGCGCGAGTTTGCGGAGCAGCTGGACGAGCTGTGGGGCAGCGAGCCCACCTACACGCCGACGGTAGAGGACCAAGCGAGCTGGCTGCAGCAGCTGCACTACGGATGGATCGGGGACTACATCTACAAGGCTGCGACGGGGAGCATTACCGAGGCGGACCTGCCACCGCCGTCGCGGAGCACACGGACCTACCACACGGGGCGCAAGCTGTCGCGGCAGGCGCATGCCGACATCGACGCAAGCCGGCGGTGGGACGGGTATGTCGGGTGCGAGGTCATGTACGAGGCGGAGGCGGAAACCAGCGGCGCGCTGCGGTGGGTCGGGTATTTGCAGCAGTCGGACTACCCGCGATCGCTGGTAGCTGGGGTGGAGTGGCGTGTGCCGCCGCGGTACCGGCGGCAGGCCACGCCGGGCAGCGCGGCGGGGCTTCACAACGGCGTCGTGCACGGTGAGCGGCTGTTTCGGCCATACGAAGACAACTATCTCTGCTCGTGCGAACCGGTTGAACGGCTTTACCTAAGCTCGACGTGCAGTCTGATGCGTCCCGGGCCGCCGCCGTCGCCGGATCTCCTGTTTACACTGTTCAAGGCGCACTCGTACCACGTGTGGGCGCAGATCCTGCCGAAGCTGCTGGCAGACGTTACCGCGCTGATGCTGCCGGTGTTGTTGGAGTACTTTGTGAAGTATTTGGACGCCGACAACGCGACGTGGGGCTGGGGTCTGGGACTTGTGCTGACACTCTTCCTCACGAACGTGATCCAGAGCTGCGCAGCGCACAAGTACGACCACATCAGCATCCGCAGCGCGGCGCTGTTTGAGACGTCGTCGATGGCGCTGCTGTTCGAGAAGTGCTTCACAGTTTCGCAGCGGTCGCTGCAGCGCCCAGACATGTCCGTGGGTCGCATCATGAACATGGTCGGGAATGACGTTGATAACATCGGTAGTCTCAACTGGTACGTGATGTATTTTTGGAGCGCCCCGCTGCAACTGACACTGTGCATGCTGCTGCTGATAAGGCTGGTCGGGTGGTTCGCGCTGCCCGGCATGGCCGTCTTGTTGGTGACGCTCCCGTTACAAGGTGCCATTTCTAAGCATGTTCAGGAGGTGTCTGAGCGAATGGCGAGCGTGGTGGACCTGCGTATCAAGCGCACGAGCGAGCTGCTCTCCGGTGCCCGAATCGTGAAATTCATGGGGTGGGAACCTGTCTTCCTCGCCCGGATCGAAGACGCGCGCAGTCGCGAGCTGCAGTGTCTGCGCGATGTGCATCTCGCCAATGTCATCTTCATGTTCGTGAACGACGCGACGCCGACGTTGGTCGTCGCTGTAGTCTTTATCTTGTACCATGCGAGTGGGCAGGCGCTGAGACCGGAGATTGTGTTTCCGACGATTGCACTGCTAAACACGATGCGCGTGTCGTTCTTTATGATCCCGCTTATCGTCTCCGCCATCCTGCAGTGTTTGGTGTCCACAAAGCGGGTCACCACTTTTGTGGAGTGCCCTGACACGCGCTCACAGGTGCGGGACATTGCTGGCATCGACGCAGCTGGTGCCGCGGCCATCTTCAAGGATGTGTCCATCCACACCTATCTGCCGGCGAAACTGCCCCAGTGCAAGTCGCGCTTGACGACCATGCAGCGCATTACGCTGTGGTTCCGCCGGCGCGGTGTGCCGGAGGCGGAGTGGTACGATCTGGACGGCCCGGATGCGGGTGCGTCTTCGCCGACGGTGCACCTTCCAACGCTCGACATGGGCAGCGCACAGACTGCCAGTGCGGATGGTGACGGCGCTGCCACTGGCGGCGGGGACGAGGAGGGGGACGTGGAAGAGAGAGTCGCACAGTACTACCAACTTGTGCCGAAGGAGCTGCTGCGGAACGTCAACCTCGTTATCCCTCAGTTCAAGTTGACGATGGTGATTGGCGCGACCGGGAGCGGGAAGTCGACGCTGCTGGGCTCTCTGATGGGCGAGTACGACGTGCAGAGTGGCGAGGTGTGGGCGGAGCGGAGCATCGCGTACGTGCCGCAGCAGGCGTGGATCATGAACGCGACGGTGCGCGACAACATCCTGTTCTTCGACGAGGAGCGCGCCGCAGACTTGCAGGACGTAATCCGTTGCTGCCAGCTGGAGGCGGACCTTGCGCAGCTTGGCGGGGGGCTGGAGACGGAGATCGGGGAAATGGGCGTGAACCTGAGCGGCGGGCAGAAGGCGCGCGTGAGCCTTGCGCGCGCCGTGTACGCGAACCGCGACGTGTACCTGCTGGACGACCCCCTGTCCGCGCTGGACGCGCACGTTGGCCAACGCGTCGTGCAAGACGTCATCCGCGGACGACTGCACGGCAAGACGCGCGTGCTTGCGACGCACCAGATTCATCTGCTGCCGCTCGCGGACTACATTGTGGTGTTGCAGCGCGGCCGCATCGCATTCGCGGGCGATTACGCTGCCTTTGCGGCGACTTCGCTGGAGGAGACGCTGCGCGGTGAGCTAAAGGAGAACAAGGACGCGGAGTCTCGCGGCAGCGATGCGGACGCGGAAGCGGCCAGAGCGGAGACGGCACCTGACATTGCGGAAGCGCATGAGCCGAAAGTTGAGCAGGAGACGAGCCTTGCAGGAGGCGAAGACCCCTTGAGGTCCGATGTCGAGGCCGGTAGGCTGATGACCAGGGAGGAGAAGGCAACCGGTCAGGTACCGTGGTCAACCTACGTGGCGTATCTGAGGTCGTGTGGCGGTCTGGCTGCTTGGGGATTCCTGCTAGCCACCTTTGCGGTGACAGAGAGCGTGACTGCGGCCAACGGCGTGTGGTTGTCGATATGGTCAACAGGCTCGCTTAGGTGTAGCGCGGCCACGTACCTGTACGTCTATCTTTTCATTGTTTTCCTCAACATCTTTAACTCCCCGCTGCGGTGTTTCTTATGCTATTACCTTATGCGAATGGGTAGCCGCAACTTGCATCGCGACCTCCTGGAGTCCATCGGCGTTGCGCGGATGTCCTTCTTCGACACGACGCCCTTGGGACGCGTGCTGAACCGGTTCACAAAGGACATGGGCATCCTTGATAACACGCTGAACGACAGCTACCTTTACCTGCTGCAGTACTTCTTTTCCATGTGCTCCAAGGTTATTATTCTGTCGGCCGCGCAGCCGTTTGTGCTTGTGGCCATTGTGCCGTGTGTGTTCATCTACTACAAGCTGATGCAGATCTACAGCGCGTCGAACCGCGAGACACGCCGCATCAAGAGCATCGCGCACTCGCCCGTGTTCACGCTGCTGGAGGAGTCGCTGCAAGGGCAGCGCACCATCGCGACGTACGGCAAGATGCACCTCGTACTGCAGGAAGCACTGAGGCGGCTCGATGTGGTCTACAGCGCGCTGTACATGCAGAACGTCTCCAACCGCTGGCTCGGTGTCCGCCTCGAGTTTGTGAGCTGTGTTATCACTTTCGTGGTGGCCCTCATTGGTGTGATTGGGAAAATGGAGCGGGCTTCGAGTCAGAGCATCGGTCTCATCTCGCTGTCACTGACGATGGCGATCACGCTGACGGAAACGCTCAATTGGCTGGTGCGGCAGGTTGCGACGGTGGAGGCAAACATGAACAGCGTGGAGCGTGTGATGTATTACACCCACGAAGTGGAGCACGAGTACGTGCCGGAGATGAAGGAGTTAGTGGCACAGCTGGTAGGGAGCGAGTCAGGGACAGCGGCGGAANNNNNNNNNNNNNNNNNNNNNNNNNNNNNNNNNNNNNNNNNNNNNNNNNNNNNNNNNNNNNNNNNNNNNNNNNNNNNNNNNNNNNNNNNNNNNNNNNNNNNNNNNNNNNNNNNNNNNNNNNNNNNNNNNNNNNNNNNNNNNNNNNNNNNNNNNNNNNNNNNNNNNNNNNNNNNNNNNNNNNNNNNNNNNNNNNNNNNNNNNNNNNNNNNNNNNNNNNNNNNNNNNNNNNNNNNNNNNNNNNNNNNNNNNNNNNNNNNNNNNNNNNNNNNNNNNNNNNNNNNNNNNNNNNNNNNNNNNNNNNNNNNNNNNNNNNNNNNNNNNNNNNNNNNNNNNNNNNNNNNNNNNNNNNNNNNNNNNNNNNNNNNNNNNNNNNNNNNNNNNNNNNNNNNNNNNNNNNNNNNNNNNNNNNNNNNNNNNNNNNNNNNNNNNNNNNNNNNNNNNNNNNNNNNNNNNNNNNNNNNNNNNNNNNNNNNNNNNNNNNNNNNNNNNNNNNNNNNNNNNNNNNNNNNNNNNNNNNNNNNNNNNNNNNNNNNNNNNNNNNNNNNNNNNNNNNNNNNNNNNNNNNNNNNNNNNNNNNNNNNNNNNNNNNNNNNNNNNNNNNNNNNNNNNNNNNNNNNNNNNNNNNNNNNNNNNNNNNNNNNNNNNNNNNNNNNNNNNNNNNNNNNNNNNNNNNNNNNNNNNNNNNNNNNNNNNNNNNNNNNNNNNNNNNNNNNNNNNNNNNNNNNNNNNNNNNNNNNNNNNNNNNNNNNNNNNNNNNNNNNNNNNNNNNNNNNNNNNNNNACTGGGCCGCCGTGGTGCCAAAGACTTCGTCGATCTCCTCGGCTGAGTCGCACGAGTCTGTGCAGCAGCCCGTGCGCACCAATGAAAGACTCCGTCGCAACCTGCCGATCTCAAAGCAGAAGCCGCTCGTCGTACGCCACCCCGCATGCACGTGCAGGAGAACGAAGTGCTCCACATGCCGTGTACCAATCTTGCCTTTGTCTTCTCCTATTCACGGCAGCGCGCTTGCATTTGCGCGATGGCGGTGCGTCGTCGGTACCCGCGGACTAGGCGCCACAGCGGCCATATGCTAGTTCATTGAATAGCACCTTACATCCTTACCGTTTCTGAGTGAAATCTGTATGCATGTGGAACACAAAGTCTTGCACGGCATAGCCGGCTAGGGGAGCGCCTCTGAGTCGTGCGTGACAGTATTGGCTGTACGATATCGTTCAAGACGCCACGAGTGCAGCTGCGCCGCCGCGACGGAGTCGCCTGCATGCCGGCTGAGGATGCGCGTGAAGGCGCGCCGATGCTTTCTCGGTGCTACTTTCTCCTTTCTCTCCC

>BPK206/0 clone 10|LinJ23_V3.0290|500 bp UPS + CDS + 500 bp DWS|multidrug resistance protein A

CTCCGCGCCGCATGCGAAGCCTGTTGTCCGCGTAGTTGCGTACGTGTGTATCTGCGCGCGTTGACTCGTGTCCTCTTCGCACCTCCTTCTGCTGGTTTGCCGCTCTCACCCGTAAGCGTGAATCAGTCTTCACGAGGCGCTTAGAGTTTCTTTTCTTTCGTGGAGGTGGTGGTCGCCAAGTGTGTGAGCTGATCGGTGCGCGAGGCGTGCGTGTGCCGAGTGCATCCTGTCCCCGACCCCCGCCTCTGGCTCCTCCGGTGCTACGCCTGAGTCTTCCCTGGCCCAGCCAGCACGGTGCCCGTCGTTGAAAGAGTGTGCCAAGACACACCTCGCCCCCTCGGTTTGTGCGCGGTCTGTCACACTGGTGCTGTTCGACCGCTGTTGTGCGGCTCGTAGGACAGGGTTGTGCGAACTACGCCTCCCTCGTTCTCTTCGCATAGGAAAGACATTGTAGACATCGGTGCATACATTGGGGGGGGGCATCCACAGCGCGCAGGGCAATGGCGACACCAGACTTTGTACGTTTATCACCGACTGACGAGTCCAGCGAGCTCATAATCCATGCTCCCTCGCAGCGGTGCCACACCCGATGTGGCGCAGAAAACGGTCATACCACCAGCATCATTACTGATTTGGAGGCGGTGGACGATGTTGCGCAGGTGCGCTGCCAGCAGCAGGCGCAGCGCGAGTTTGCGGAGCAGCTGGACGAGCTGTGGGGCAGCGAGCCCACCTACACGCCGACGGTAGAGGACCAAGCGAGCTGGCTGCAGCAGCTGCACTACGGATGGATCGGGGACTACATCTACAAGGCTGCGACGGGGAGCATTACCGAGGCGGACCTGCCACCGCCGTCGCGGAGCACACGGACCTACCACACGGGGCGCAAGCTGTCGCGGCAGGCGCATGCCGACATCGACGCAAGCCGGCGGTGGGACGGGTATGTCGGGTGCGAGGTCATGTACGAGGCGGAGGCGGAAACCAGCGGCGCGCTGCGGTGGGTCGGGTATTTGCAGCAGTCGGACTACCCGCGATCGCTGGTAGCTGGGGTGGAGTGGCGTGTGCCGCCGCGGTACCGGCGGCAGGCCACGCCGGGCAGCGCGGCGGGGCTTCACAACGGCGTCGTGCACGGTGAGCGGCTGTTTCGGCCATACGAAGACAACTATCTCTGCTCGTGCGAACCGGTTGAACGGCTTTACCTAAGCTCGACGTGCAGTCTGATGCGTCCCGGGCCGCCGCCGTCGCCGGATCTCCTGTTTACACTGTTCAAGGCGCACTCGTACCACGTGTGGGCGCAGATCCTGCCGAAGCTGCTGGCAGACGTTACCGCGCTGATGCTGCCGGTGTTGTTGGAGTACTTTGTGAAGTATTTGGACGCCGACAACGCGACGTGGGGCTGGGGTCTGGGACTTGTGCTGACACTCTTCCTCACGAACGTGATCCAGAGCTGCGCAGCGCACAAGTACGACCACATCAGCATCCGCAGCGCGGCGCTGTTTGAGACGTCGTCGATGGCGCTGCTGTTCGAGAAGTGCTTCACAGTTTCGCAGCGGTCGCTGCAGCGCCCAGACATGTCCGTGGGTCGCATCATGAACATGGTCGGGAATGACGTTGATAACATCGGTAGTCTCAACTGGTACGTGATGTATTTTTGGAGCGCCCCGCTGCAACTGACACTGTGCATGCTGCTGCTGATAAGGCTGGTCGGGTGGTTCGCGCTGCCCGGCATGGCCGTCTTGTTGGTGACGCTCCCGTTACAAGGTGCCATTTCTAAGCATGTTCAGGAGGTGTCTGAGCGAATGGCGAGCGTGGTGGACCTGCGTATCAAGCGCACGAGCGAGCTGCTCTCCGGTGCCCGAATCGTGAAATTCATGGGGTGGGAACCTGTCTTCCTCGCCCGGATCGAAGACGCGCGCAGTCGCGAGCTGCAGTGTCTGCGCGATGTGCATCTCGCCAATGTCATCTTCATGTTCGTGAACGACGCGACGCCGACGTTGGTCGTCGCTGTAGTCTTTATCTTGTACCATGCGAGTGGGCAGGCGCTGAGACCGGAGATTGTGTTTCCGACGATTGCACTGCTAAACACGATGCGCGTGTCGTTCTTTATGATCCCGCTTATCGTCTCCGCCATCCTGCAGTGTTTGGTGTCCACAAAGCGGGTCACCACTTTTGTGGAGTGCCCTGACACGCGCTCACAGGTGCGGGACATTGCTGGCATCGACGCAGCTGGTGCCGCGGCCATCTTCAAGGATGTGTCCATCCACACCTATCTGCCGGCGAAACTGCCCCAGTGCAAGTCGCGCTTGACGACCATGCAGCGCATTACGCTGTGGTTCCGCCGGCGCGGTGTGCCGGAGGCGGAGTGGTACGATCTGGACGGCCCGGATGCGGGTGCGTCTTCGCCGACGGTGCACCTTCCAACGCTCGACATGGGCAGCGCACAGACTGCCAGTGCGGATGGTGACGGCGCTGCCACTGGCGGCGGGGACGAGGAGGGGGACGTGGAAGAGAGAGTCGCACAGTACTACCAACTTGTGCCGAAGGAGCTGCTGCGGAACGTCAACCTCGTTATCCCTCAGTTCAAGTTGACGATGGTGATTGGCGCGACCGGGAGCGGGAAGTCGACGCTGCTGGGCTCTCTGATGGGCGAGTACGACGTGCAGAGTGGCGAGGTGTGGGCGGAGCGGAGCATCGCGTACGTGCCGCAGCAGGCGTGGATCATGAACGCGACGGTGCGCGACAACATCCTGTTCTTCGACGAGGAGCGCGCCGCAGACTTGCAGGACGTAATCCGTTGCTGCCAGCTGGAGGCGGACCTTGCGCAGCTTGGCGGGGGGCTGGAGACGGAGATCGGGGAAATGGGCGTGAACCTGAGCGGCGGGCAGAAGGCGCGCGTGAGCCTTGCGCGCGCCGTGTACGCGAACCGCGACGTGTACCTGCTGGACGACCCCCTGTCCGCGCTGGACGCGCACGTTGGCCAACGCGTCGTGCAAGACGTCATCCGCGGACGACTGCACGGCAAGACGCGCGTGCTTGCGACGCACCAGATTCATCTGCTGCCGCTCGCGGACTACATTGTGGTGTTGCAGCGCGGCCGCATCGCATTCGCGGGCGATTACGCTGCCTTTGCGGCGACTTCGCTGGAGGAGACGCTGCGCGGTGAGCTAAAGGAGAACAAGGACGCGGAGTCTCGCGGCAGCGATGCGGACGCGGAAGCGGCCAGAGCGGAGACGGCACCTGACATTGCGGAAGCGCATGAGCCGAAAGTTGAGCAGGAGACGAGCCTTGCAGGAGGCGAAGACCCCTTGAGGTCCGATGTCGAGGCCGGTAGGCTGATGACCAGGGAGGAGAAGGCAACCGGTCAGGTACCGTGGTCAACCTACGTGGCGTATCTGAGGTCGTGTGGCGGTCTGGCTGCTTGGGGATTCCTGCTAGCCACCTTTGCGGTGACAGAGAGCGTGACTGCGGCCAACGGCGTGTGGTTGTCGATATGGTCAACAGGCTCGCTTAGGTGTAGCGCGGCCACGTACCTGTACGTCTATCTTTTCATTGTTTTCCTCAACATCTTTAACTCCCCGCTGCGGTGTTTCTTATGCTATTACCTTATGCGAATGGGTAGCCGCAACTTGCATCGCGACCTCCTGGAGTCCATCGGCGTTGCGCGGATGTCCTTCTTCGACACGACGCCCTTGGGACGCGTGCTGAACCGGTTCACAAAGGACATGGGCATCCTTGATAACACGCTGAACGACAGCTACCTTTACCTGCTGCAGTACTTCTTTTCCATGTGCTCCAAGGTTATTATTCTGTCGGCCGCGCAGCCGTTTGTGCTTGTGGCCATTGTGCCGTGTGTGTTCATCTACTACAAGCTGATGCAGATCTACAGCGCGTCGAACCGCGAGACACGCCGCATCAAGAGCATCGCGCACTCGCCCGTGTTCACGCTGCTGGAGGAGTCGCTGCAAGGGCAGCGCACCATCGCGACGTACGGCAAGATGCACCTCGTACTGCAGGAAGCACTGAGGCGGCTCGATGTGGTCTACAGCGCGCTGTACATGCAGAACGTCTCCAACCGCTGGCTCGGTGTCCGCCTCGAGTTTGTGAGCTGTGTTATCACTTTCGTGGTGGCCCTCATTGGTGTGATTGGGAAAATGGAGCGGGCTTCGAGTCAGAGCATCGGTCTCATCTCGCTGTCACTGACGATGGCGATCACGCTGACGGAAACGCTCAATTGGCTGGTGCGGCAGGTTGCGACGGTGGAGGCAAACATGAACAGCGTGGAGCGTGTGATGTATTACACCCACGAAGTGGAGCACGAGTACGTGCCGGAGATGAAGGAGTTAGTGGCACAGCTGGTAGGGAGCGAGTCAGGGACAGCGGCGGAANNNNNNNNNNNNNNNNNNNNNNNNNNNNNNNNNNNNNNNNNNNNNNNNNNNNNNNNNNNNNNNNNNNNNNNNNNNNNNNNNNNNNNNNNNNNNNNNNNNNNNNNNNNNNNNNNNNNNNNNNNNNNNNNNNNNNNNNNNNNNNNNNNNNNNNNNNNNNNNNNNNNNNNNNNNNNNNNNNNNNNNNNNNNNNNNNNNNNNNNNNNNNNNNNNNNNNNNNNNNNNNNNNNNNNNNNNNNNNNNNNNNNNNNNNNNNNNNNNNNNNNNNNNNNNNNNNNNNNNNNNNNNNNNNNNNNNNNNNNNNNNNNNNNNNNNNNNNNNNNNNNNNNNNNNNNNNNNNNNNNNNNNNNNNNNNNNNNNNNNNNNNNNNNNNNNNNNNNNNNNNNNNNNNNNNNNNNNNNNNNNNNNNNNNNNNNNNNNNNNNNNNNNNNNNNNNNNNNNNNNNNNNNNNNNNNNNNNNNNNNNNNNNNNNNNNNNNNNNNNNNNNNNNNNNNNNNNNNNNNNNNNNNNNNNNNNNNNNNNNNNNNNNNNNNNNNNNNNNNNNNNNNNNNNNNNNNNNNNNNNNNNNNNNNNNNNNNNNNNNNNNNNNNNNNNNNNNNNNNNNNNNNNNNNNNNNNNNNNNNNNNNNNNNNNNNNNNNNNNNNNNNNNNNNNNNNNNNNNNNNNNNNNNNNNNNNNNNNNNNNNNNNNNNNNNNNNNNNNNNNNNNNNNNNNNNNNNNNNNNNNNNNNNNNNNNNNNNNNNNNNNNNNNNNNNNNNNNNNNNNNNNNNNNNNNNNNNNNNNNACTGGGCCGCCGTGGTGCCAAAGACTTCGTCGATCTCCTCGGCTGAGTCGCACGAGTCTGTGCAGCAGCCCGTGCGCACCAATGAAAGACTCCGTCGCAACCTGCCGATCTCAAAGCAGAAGCCGCTCGTCGTACGCCACCCCGCATGCACGTGCAGGAGAACGAAGTGCTCCACATGCCGTGTACCAATCTTGCCTTTGTCTTCTCCTATTCACGGCAGCGCGCTTGCATTTGCGCGATGGCGGTGCGTCGTCGGTACCCGCGGACTAGGCGCCACAGCGGCCATATGCTAGTTCATTGAATAGCACCTTACATCCTTACCGTTTCTGAGTGAAATCTGTATGCATGTGGAACACAAAGTCTTGCACGGCATAGCCGGCTAGGGGAGCGCCTCTGAGTCGTGCGTGACAGTATTGGCTGTACGATATCGTTCAAGACGCCACGAGTGCAGCTGCGCCGCCGCGACGGAGTCGCCTGCATGCCGGCTGAGGATGCGCGTGAAGGCGCGCCGATGCTTTCTCGGTGCTACTTTCTCCTTTCTCTCCC

>BPK275/0 clone 18|LinJ23_V3.0290|500 bp UPS + CDS + 500 bp DWS|multidrug resistance protein A

CTCCGCGCCGCATGCGAAGCCTGTTGTCCGCGTAGTTGCGTACGTGTGTATCTGCGCGCGTTGACTCGTGTCCTCTTCGCACCTCCTTCTGCTGGTTTGCCGCTCTCACCCGTAAGCGTGAATCAGTCTTCACGAGGCGCTTAGAGTTTCTTTTCTTTCGTGGAGGTGGTGGTCGCCAAGTGTGTGAGCTGATCGGTGCGCGAGGCGTGCGTGTGCCGAGTGCATCCTGTCCCCGACCCCCGCCTCTGGCTCCTCCGGTGCTACGCCTGAGTCTTCCCTGGCCCAGCCAGCACGGTGCCCGTCGTTGAAAGAGTGTGCCAAGACACACCTCGCCCCCTCGGTTTGTGCGCGGTCTGTCACACTGGTGCTGTTCGACCGCTGTTGTGCGGCTCGTAGGACAGGGTTGTGCGAACTACGCCTCCCTCGTTCTCTTCGCATAGGAAAGACATTGTAGACATCGGTGCATACATTGGGGGGGGGCATCCACAGCGCGCAGGGCAATGGCGACACCAGACTTTGTACGTTTATCACCGACTGACGAGTCCAGCGAGCTCATAATCCATGCTCCCTCGCAGCGGTGCCACACCCGATGTGGCGCAGAAAACGGTCATACCACCAGCATCATTACTGATTTGGAGGCGGTGGACGATGTTGCGCAGGTGCGCTGCCAGCAGCAGGCGCAGCGCGAGTTTGCGGAGCAGCTGGACGAGCTGTGGGGCAGCGAGCCCACCTACACGCCGACGGTAGAGGACCAAGCGAGCTGGCTGCAGCAGCTGCACTACGGATGGATCGGGGACTACATCTACAAGGCTGCGACGGGGAGCATTACCGAGGCGGACCTGCCACCGCCGTCGCGGAGCACACGGACCTACCACACGGGGCGCAAGCTGTCGCGGCAGGCGCATGCCGACATCGACGCAAGCCGGCGGTGGGACGGGTATGTCGGGTGCGAGGTCATGTACGAGGCGGAGGCGGAAACCAGCGGCGCGCTGCGGTGGGTCGGGTATTTGCAGCAGTCGGACTACCCGCGATCGCTGGTAGCTGGGGTGGAGTGGCGTGTGCCGCCGCGGTACCGGCGGCAGGCCACGCCGGGCAGCGCGGCGGGGCTTCACAACGGCGTCGTGCACGGTGAGCGGCTGTTTCGGCCATACGAAGACAACTATCTCTGCTCGTGCGAACCGGTTGAACGGCTTTACCTAAGCTCGACGTGCAGTCTGATGCGTCCCGGGCCGCCGCCGTCGCCGGATCTCCTGTTTACACTGTTCAAGGCGCACTCGTACCACGTGTGGGCGCAGATCCTGCCGAAGCTGCTGGCAGACGTTACCGCGCTGATGCTGCCGGTGTTGTTGGAGTACTTTGTGAAGTATTTGGACGCCGACAACGCGACGTGGGGCTGGGGTCTGGGACTTGTGCTGACACTCTTCCTCACGAACGTGATCCAGAGCTGCGCAGCGCACAAGTACGACCACATCAGCATCCGCAGCGCGGCGCTGTTTGAGACGTCGTCGATGGCGCTGCTGTTCGAGAAGTGCTTCACAGTTTCGCAGCGGTCGCTGCAGCGCCCAGACATGTCCGTGGGTCGCATCATGAACATGGTCGGGAATGACGTTGATAACATCGGTAGTCTCAACTGGTACGTGATGTATTTTTGGAGCGCCCCGCTGCAACTGACACTGTGCATGCTGCTGCTGATAAGGCTGGTCGGGTGGTTCGCGCTGCCCGGCATGGCCGTCTTGTTGGTGACGCTCCCGTTACAAGGTGCCATTTCTAAGCATGTTCAGGAGGTGTCTGAGCGAATGGCGAGCGTGGTGGACCTGCGTATCAAGCGCACGAGCGAGCTGCTCTCCGGTGCCCGAATCGTGAAATTCATGGGGTGGGAACCTGTCTTCCTCGCCCGGATCGAAGACGCGCGCAGTCGCGAGCTGCAGTGTCTGCGCGATGTGCATCTCGCCAATGTCATCTTCATGTTCGTGAACGACGCGACGCCGACGTTGGTCGTCGCTGTAGTCTTTATCTTGTACCATGCGAGTGGGCAGGCGCTGAGACCGGAGATTGTGTTTCCGACGATTGCACTGCTAAACACGATGCGCGTGTCGTTCTTTATGATCCCGCTTATCGTCTCCGCCATCCTGCAGTGTTTGGTGTCCACAAAGCGGGTCACCACTTTTGTGGAGTGCCCTGACACGCGCTCACAGGTGCGGGACATTGCTGGCATCGACGCAGCTGGTGCCGCGGCCATCTTCAAGGATGTGTCCATCCACACCTATCTGCCGGCGAAACTGCCCCAGTGCAAGTCGCGCTTGACGACCATGCAGCGCATTACGCTGTGGTTCCGCCGGCGCGGTGTGCCGGAGGCGGAGTGGTACGATCTGGACGGCCCGGATGCGGGTGCGTCTTCGCCGACGGTGCACCTTCCAACGCTCGACATGGGCAGCGCACAGACTGCCAGTGCGGATGGTGACGGCGCTGCCACTGGCGGCGGGGACGAGGAGGGGGACGTGGAAGAGAGAGTCGCACAGTACTACCAACTTGTGCCGAAGGAGCTGCTGCGGAACGTCAACCTCGTTATCCCTCAGTTCAAGTTGACGATGGTGATTGGCGCGACCGGGAGCGGGAAGTCGACGCTGCTGGGCTCTCTGATGGGCGAGTACGACGTGCAGAGTGGCGAGGTGTGGGCGGAGCGGAGCATCGCGTACGTGCCGCAGCAGGCGTGGATCATGAACGCGACGGTGCGCGACAACATCCTGTTCTTCGACGAGGAGCGCGCCGCAGACTTGCAGGACGTAATCCGTTGCTGCCAGCTGGAGGCGGACCTTGCGCAGCTTGGCGGGGGGCTGGAGACGGAGATCGGGGAAATGGGCGTGAACCTGAGCGGCGGGCAGAAGGCGCGCGTGAGCCTTGCGCGCGCCGTGTACGCGAACCGCGACGTGTACCTGCTGGACGACCCCCTGTCCGCGCTGGACGCGCACGTTGGCCAACGCGTCGTGCAAGACGTCATCCGCGGACGACTGCACGGCAAGACGCGCGTGCTTGCGACGCACCAGATTCATCTGCTGCCGCTCGCGGACTACATTGTGGTGTTGCAGCGCGGCCGCATCGCATTCGCGGGCGATTACGCTGCCTTTGCGGCGACTTCGCTGGAGGAGACGCTGCGCGGTGAGCTAAAGGAGAACAAGGACGCGGAGTCTCGCGGCAGCGATGCGGACGCGGAAGCGGCCAGAGCGGAGACGGCACCTGACATTGCGGAAGCGCATGAGCCGAAAGTTGAGCAGGAGACGAGCCTTGCAGGAGGCGAAGACCCCTTGAGGTCCGATGTCGAGGCCGGTAGGCTGATGACCAGGGAGGAGAAGGCAACCGGTCAGGTACCGTGGTCAACCTACGTGGCGTATCTGAGGTCGTGTGGCGGTCTGGCTGCTTGGGGATTCCTGCTAGCCACCTTTGCGGTGACAGAGAGCGTGACTGCGGCCAACGGCGTGTGGTTGTCGATATGGTCAACAGGCTCGCTTAGGTGTAGCGCGGCCACGTACCTGTACGTCTATCTTTTCATTGTTTTCCTCAACATCTTTAACTCCCCGCTGCGGTGTTTCTTATGCTATTACCTTATGCGAATGGGTAGCCGCAACTTGCATCGCGACCTCCTGGAGTCCATCGGCGTTGCGCGGATGTCCTTCTTCGACACGACGCCCTTGGGACGCGTGCTGAACCGGTTCACAAAGGACATGGGCATCCTTGATAACACGCTGAACGACAGCTACCTTTACCTGCTGCAGTACTTCTTTTCCATGTGCTCCAAGGTTATTATTCTGTCGGCCGCGCAGCCGTTTGTGCTTGTGGCCATTGTGCCGTGTGTGTTCATCTACTACAAGCTGATGCAGATCTACAGCGCGTCGAACCGCGAGACACGCCGCATCAAGAGCATCGCGCACTCGCCCGTGTTCACGCTGCTGGAGGAGTCGCTGCAAGGGCAGCGCACCATCGCGACGTACGGCAAGATGCACCTCGTACTGCAGGAAGCACTGAGGCGGCTCGATGTGGTCTACAGCGCGCTGTACATGCAGAACGTCTCCAACCGCTGGCTCGGTGTCCGCCTCGAGTTTGTGAGCTGTGTTATCACTTTCGTGGTGGCCCTCATTGGTGTGATTGGGAAAATGGAGCGGGCTTCGAGTCAGAGCATCGGTCTCATCTCGCTGTCACTGACGATGGCGATCACGCTGACGGAAACGCTCAATTGGCTGGTGCGGCAGGTTGCGACGGTGGAGGCAAACATGAACAGCGTGGAGCGTGTGATGTATTACACCCACGAAGTGGAGCACGAGTACGTGCCGGAGATGAAGGAGTTAGTGGCACAGCTGGTAGGGAGCGAGTCAGGGACAGCGGCGGAANNNNNNNNNNNNNNNNNNNNNNNNNNNNNNNNNNNNNNNNNNNNNNNNNNNNNNNNNNNNNNNNNNNNNNNNNNNNNNNNNNNNNNNNNNNNNNNNNNNNNNNNNNNNNNNNNNNNNNNNNNNNNNNNNNNNNNNNNNNNNNNNNNNNNNNNNNNNNNNNNNNNNNNNNNNNNNNNNNNNNNNNNNNNNNNNNNNNNNNNNNNNNNNNNNNNNNNNNNNNNNNNNNNNNNNNNNNNNNNNNNNNNNNNNNNNNNNNNNNNNNNNNNNNNNNNNNNNNNNNNNNNNNNNNNNNNNNNNNNNNNNNNNNNNNNNNNNNNNNNNNNNNNNNNNNNNNNNNNNNNNNNNNNNNNNNNNNNNNNNNNNNNNNNNNNNNNNNNNNNNNNNNNNNNNNNNNNNNNNNNNNNNNNNNNNNNNNNNNNNNNNNNNNNNNNNNNNNNNNNNNNNNNNNNNNNNNNNNNNNNNNNNNNNNNNNNNNNNNNNNNNNNNNNNNNNNNNNNNNNNNNNNNNNNNNNNNNNNNNNNNNNNNNNNNNNNNNNNNNNNNNNNNNNNNNNNNNNNNNNNNNNNNNNNNNNNNNNNNNNNNNNNNNNNNNNNNNNNNNNNNNNNNNNNNNNNNNNNNNNNNNNNNNNNNNNNNNNNNNNNNNNNNNNNNNNNNNNNNNNNNNNNNNNNNNNNNNNNNNNNNNNNNNNNNNNNNNNNNNNNNNNNNNNNNNNNNNNNNNNNNNNNNNNNNNNNNNNNNNNNNNNNNNNNNNNNNNNNNNNNNNNNNNNNNNNNNNNNNNNACTGGGCCGCCGTGGTGCCAAAGACTTCGTCGATCTCCTCGGCTGAGTCGCACGAGTCTGTGCAGCAGCCCGTGCGCACCAATGAAAGACTCCGTCGCAACCTGCCGATCTCAAAGCAGAAGCCGCTCGTCGTACGCCACCCCGCATGCACGTGCAGGAGAACGAAGTGCTCCACATGCCGTGTACCAATCTTGCCTTTGTCTTCTCCTATTCACGGCAGCGCGCTTGCATTTGCGCGATGGCGGTGCGTCGTCGGTACCCGCGGACTAGGCGCCACAGCGGCCATATGCTAGTTCATTGAATAGCACCTTACATCCTTACCGTTTCTGAGTGAAATCTGTATGCATGTGGAACACAAAGTCTTGCACGGCATAGCCGGCTAGGGGAGCGCCTCTGAGTCGTGCGTGACAGTATTGGCTGTACGATATCGTTCAAGACGCCACGAGTGCAGCTGCGCCGCCGCGACGGAGTCGCCTGCATGCCGGCTGAGGATGCGCGTGAAGGCGCGCCGATGCTTTCTCGGTGCTACTTTCTCCTTTCTCTCCC

>BPK282/0 clone 4|LinJ23_V3.0290|500 bp UPS + CDS + 500 bp DWS|multidrug resistance protein A

CTCCGCGCCGCATGCGAAGCCTGTTGTCCGCGTAGTTGCGTACGTGTGTATCTGCGCGCGTTGACTCGTGTCCTCTTCGCACCTCCTTCTGCTGGTTTGCCGCTCTCACCCGTAAGCGTGAATCAGTCTTCACGAGGCGCTTAGAGTTTCTTTTCTTTCGTGGAGGTGGTGGTCGCCAAGTGTGTGAGCTGATCGGTGCGCGAGGCGTGCGTGTGCCGAGTGCATCCTGTCCCCGACCCCCGCCTCTGGCTCCTCCGGTGCTACGCCTGAGTCTTCCCTGGCCCAGCCAGCACGGTGCCCGTCGTTGAAAGAGTGTGCCAAGACACACCTCGCCCCCTCGGTTTGTGCGCGGTCTGTCACACTGGTGCTGTTCGACCGCTGTTGTGCGGCTCGTAGGACAGGGTTGTGCGAACTACGCCTCCCTCGTTCTCTTCGCATAGGAAAGACATTGTAGACATCGGTGCATACATTGGGGGGGGGCATCCACAGCGCGCAGGGCAATGGCGACACCAGACTTTGTACGTTTATCACCGACTGACGAGTCCAGCGAGCTCATAATCCATGCTCCCTCGCAGCGGTGCCACACCCGATGTGGCGCAGAAAACGGTCATACCACCAGCATCATTACTGATTTGGAGGCGGTGGACGATGTTGCGCAGGTGCGCTGCCAGCAGCAGGCGCAGCGCGAGTTTGCGGAGCAGCTGGACGAGCTGTGGGGCAGCGAGCCCACCTACACGCCGACGGTAGAGGACCAAGCGAGCTGGCTGCAGCAGCTGCACTACGGATGGATCGGGGACTACATCTACAAGGCTGCGACGGGGAGCATTACCGAGGCGGACCTGCCACCGCCGTCGCGGAGCACACGGACCTACCACACGGGGCGCAAGCTGTCGCGGCAGGCGCATGCCGACATCGACGCAAGCCGGCGGTGGGACGGGTATGTCGGGTGCGAGGTCATGTACGAGGCGGAGGCGGAAACCAGCGGCGCGCTGCGGTGGGTCGGGTATTTGCAGCAGTCGGACTACCCGCGATCGCTGGTAGCTGGGGTGGAGTGGCGTGTGCCGCCGCGGTACCGGCGGCAGGCCACGCCGGGCAGCGCGGCGGGGCTTCACAACGGCGTCGTGCACGGTGAGCGGCTGTTTCGGCCATACGAAGACAACTATCTCTGCTCGTGCGAACCGGTTGAACGGCTTTACCTAAGCTCGACGTGCAGTCTGATGCGTCCCGGGCCGCCGCCGTCGCCGGATCTCCTGTTTACACTGTTCAAGGCGCACTCGTACCACGTGTGGGCGCAGATCCTGCCGAAGCTGCTGGCAGACGTTACCGCGCTGATGCTGCCGGTGTTGTTGGAGTACTTTGTGAAGTATTTGGACGCCGACAACGCGACGTGGGGCTGGGGTCTGGGACTTGTGCTGACACTCTTCCTCACGAACGTGATCCAGAGCTGCGCAGCGCACAAGTACGACCACATCAGCATCCGCAGCGCGGCGCTGTTTGAGACGTCGTCGATGGCGCTGCTGTTCGAGAAGTGCTTCACAGTTTCGCAGCGGTCGCTGCAGCGCCCAGACATGTCCGTGGGTCGCATCATGAACATGGTCGGGAATGACGTTGATAACATCGGTAGTCTCAACTGGTACGTGATGTATTTTTGGAGCGCCCCGCTGCAACTGACACTGTGCATGCTGCTGCTGATAAGGCTGGTCGGGTGGTTCGCGCTGCCCGGCATGGCCGTCTTGTTGGTGACGCTCCCGTTACAAGGTGCCATTTCTAAGCATGTTCAGGAGGTGTCTGAGCGAATGGCGAGCGTGGTGGACCTGCGTATCAAGCGCACGAGCGAGCTGCTCTCCGGTGCCCGAATCGTGAAATTCATGGGGTGGGAACCTGTCTTCCTCGCCCGGATCGAAGACGCGCGCAGTCGCGAGCTGCAGTGTCTGCGCGATGTGCATCTCGCCAATGTCATCTTCATGTTCGTGAACGACGCGACGCCGACGTTGGTCGTCGCTGTAGTCTTTATCTTGTACCATGCGAGTGGGCAGGCGCTGAGACCGGAGATTGTGTTTCCGACGATTGCACTGCTAAACACGATGCGCGTGTCGTTCTTTATGATCCCGCTTATCGTCTCCGCCATCCTGCAGTGTTTGGTGTCCACAAAGCGGGTCACCACTTTTGTGGAGTGCCCTGACACGCGCTCACAGGTGCGGGACATTGCTGGCATCGACGCAGCTGGTGCCGCGGCCATCTTCAAGGATGTGTCCATCCACACCTATCTGCCGGCGAAACTGCCCCAGTGCAAGTCGCGCTTGACGACCATGCAGCGCATTACGCTGTGGTTCCGCCGGCGCGGTGTGCCGGAGGCGGAGTGGTACGATCTGGACGGCCCGGATGCGGGTGCGTCTTCGCCGACGGTGCACCTTCCAACGCTCGACATGGGCAGCGCACAGACTGCCAGTGCGGATGGTGACGGCGCTGCCACTGGCGGCGGGGACGAGGAGGGGGACGTGGAAGAGAGAGTCGCACAGTACTACCAACTTGTGCCGAAGGAGCTGCTGCGGAACGTCAACCTCGTTATCCCTCAGTTCAAGTTGACGATGGTGATTGGCGCGACCGGGAGCGGGAAGTCGACGCTGCTGGGCTCTCTGATGGGCGAGTACGACGTGCAGAGTGGCGAGGTGTGGGCGGAGCGGAGCATCGCGTACGTGCCGCAGCAGGCGTGGATCATGAACGCGACGGTGCGCGACAACATCCTGTTCTTCGACGAGGAGCGCGCCGCAGACTTGCAGGACGTAATCCGTTGCTGCCAGCTGGAGGCGGACCTTGCGCAGCTTGGCGGGGGGCTGGAGACGGAGATCGGGGAAATGGGCGTGAACCTGAGCGGCGGGCAGAAGGCGCGCGTGAGCCTTGCGCGCGCCGTGTACGCGAACCGCGACGTGTACCTGCTGGACGACCCCCTGTCCGCGCTGGACGCGCACGTTGGCCAACGCGTCGTGCAAGACGTCATCCGCGGACGACTGCACGGCAAGACGCGCGTGCTTGCGACGCACCAGATTCATCTGCTGCCGCTCGCGGACTACATTGTGGTGTTGCAGCGCGGCCGCATCGCATTCGCGGGCGATTACGCTGCCTTTGCGGCGACTTCGCTGGAGGAGACGCTGCGCGGTGAGCTAAAGGAGAACAAGGACGCGGAGTCTCGCGGCAGCGATGCGGACGCGGAAGCGGCCAGAGCGGAGACGGCACCTGACATTGCGGAAGCGCATGAGCCGAAAGTTGAGCAGGAGACGAGCCTTGCAGGAGGCGAAGACCCCTTGAGGTCCGATGTCGAGGCCGGTAGGCTGATGACCAGGGAGGAGAAGGCAACCGGTCAGGTACCGTGGTCAACCTACGTGGCGTATCTGAGGTCGTGTGGCGGTCTGGCTGCTTGGGGATTCCTGCTAGCCACCTTTGCGGTGACAGAGAGCGTGACTGCGGCCAACGGCGTGTGGTTGTCGATATGGTCAACAGGCTCGCTTAGGTGTAGCGCGGCCACGTACCTGTACGTCTATCTTTTCATTGTTTTCCTCAACATCTTTAACTCCCCGCTGCGGTGTTTCTTATGCTATTACCTTATGCGAATGGGTAGCCGCAACTTGCATCGCGACCTCCTGGAGTCCATCGGCGTTGCGCGGATGTCCTTCTTCGACACGACGCCCTTGGGACGCGTGCTGAACCGGTTCACAAAGGACATGGGCATCCTTGATAACACGCTGAACGACAGCTACCTTTACCTGCTGCAGTACTTCTTTTCCATGTGCTCCAAGGTTATTATTCTGTCGGCCGCGCAGCCGTTTGTGCTTGTGGCCATTGTGCCGTGTGTGTTCATCTACTACAAGCTGATGCAGATCTACAGCGCGTCGAACCGCGAGACACGCCGCATCAAGAGCATCGCGCACTCGCCCGTGTTCACGCTGCTGGAGGAGTCGCTGCAAGGGCAGCGCACCATCGCGACGTACGGCAAGATGCACCTCGTACTGCAGGAAGCACTGAGGCGGCTCGATGTGGTCTACAGCGCGCTGTACATGCAGAACGTCTCCAACCGCTGGCTCGGTGTCCGCCTCGAGTTTGTGAGCTGTGTTATCACTTTCGTGGTGGCCCTCATTGGTGTGATTGGGAAAATGGAGCGGGCTTCGAGTCAGAGCATCGGTCTCATCTCGCTGTCACTGACGATGGCGATCACGCTGACGGAAACGCTCAATTGGCTGGTGCGGCAGGTTGCGACGGTGGAGGCAAACATGAACAGCGTGGAGCGTGTGATGTATTACACCCACGAAGTGGAGCACGAGTACGTGCCGGAGATGAAGGAGTTAGTGGCACAGCTGGTAGGGAGCGAGTCAGGGACAGCGGCGGAANNNNNNNNNNNNNNNNNNNNNNNNNNNNNNNNNNNNNNNNNNNNNNNNNNNNNNNNNNNNNNNNNNNNNNNNNNNNNNNNNNNNNNNNNNNNNNNNNNNNNNNNNNNNNNNNNNNNNNNNNNNNNNNNNNNNNNNNNNNNNNNNNNNNNNNNNNNNNNNNNNNNNNNNNNNNNNNNNNNNNNNNNNNNNNNNNNNNNNNNNNNNNNNNNNNNNNNNNNNNNNNNNNNNNNNNNNNNNNNNNNNNNNNNNNNNNNNNNNNNNNNNNNNNNNNNNNNNNNNNNNNNNNNNNNNNNNNNNNNNNNNNNNNNNNNNNNNNNNNNNNNNNNNNNNNNNNNNNNNNNNNNNNNNNNNNNNNNNNNNNNNNNNNNNNNNNNNNNNNNNNNNNNNNNNNNNNNNNNNNNNNNNNNNNNNNNNNNNNNNNNNNNNNNNNNNNNNNNNNNNNNNNNNNNNNNNNNNNNNNNNNNNNNNNNNNNNNNNNNNNNNNNNNNNNNNNNNNNNNNNNNNNNNNNNNNNNNNNNNNNNNNNNNNNNNNNNNNNNNNNNNNNNNNNNNNNNNNNNNNNNNNNNNNNNNNNNNNNNNNNNNNNNNNNNNNNNNNNNNNNNNNNNNNNNNNNNNNNNNNNNNNNNNNNNNNNNNNNNNNNNNNNNNNNNNNNNNNNNNNNNNNNNNNNNNNNNNNNNNNNNNNNNNNNNNNNNNNNNNNNNNNNNNNNNNNNNNNNNNNNNNNNNNNNNNNNNNNNNNNNNNNNNNNNNNNNNNNNNNNNNNNNNNNNNNNNNNNNNNNACTGGGCCGCCGTGGTGCCAAAGACTTCGTCGATCTCCTCGGCTGAGTCGCACGAGTCTGTGCAGCAGCCCGTGCGCACCAATGAAAGACTCCGTCGCAACCTGCCGATCTCAAAGCAGAAGCCGCTCGTCGTACGCCACCCCGCATGCACGTGCAGGAGAACGAAGTGCTCCACATGCCGTGTACCAATCTTGCCTTTGTCTTCTCCTATTCACGGCAGCGCGCTTGCATTTGCGCGATGGCGGTGCGTCGTCGGTACCCGCGGACTAGGCGCCACAGCGGCCATATGCTAGTTCATTGAATAGCACCTTACATCCTTACCGTTTCTGAGTGAAATCTGTATGCATGTGGAACACAAAGTCTTGCACGGCATAGCCGGCTAGGGGAGCGCCTCTGAGTCGTGCGTGACAGTATTGGCTGTACGATATCGTTCAAGACGCCACGAGTGCAGCTGCGCCGCCGCGACGGAGTCGCCTGCATGCCGGCTGAGGATGCGCGTGAAGGCGCGCCGATGCTTTCTCGGTGCTACTTTCTCCTTTCTCTCCC

>BPK294/0 clone 1|LinJ23_V3.0290|500 bp UPS + CDS + 500 bp DWS|multidrug resistance protein A

CTCCGCGCCGCATGCGAAGCCTGTTGTCCGCGTAGTTGCGTACGTGTGTATCTGCGCGCGTTGACTCGTGTCCTCTTCGCACCTCCTTCTGCTGGTTTGCCGCTCTCACCCGTAAGCGTGAATCAGTCTTCACGAGGCGCTTAGAGTTTCTTTTCTTTCGTGGAGGTGGTGGTCGCCAAGTGTGTGAGCTGATCGGTGCGCGAGGCGTGCGTGTGCCGAGTGCATCCTGTCCCCGACCCCCGCCTCTGGCTCCTCCGGTGCTACGCCTGAGTCTTCCCTGGCCCAGCCAGCACGGTGCCCGTCGTTGAAAGAGTGTGCCAAGACACACCTCGCCCCCTCGGTTTGTGCGCGGTCTGTCACACTGGTGCTGTTCGACCGCTGTTGTGCGGCTCGTAGGACAGGGTTGTGCGAACTACGCCTCCCTCGTTCTCTTCGCATAGGAAAGACATTGTAGACATCGGTGCATACATTGGGGGGGGGCATCCACAGCGCGCAGGGCAATGGCGACACCAGACTTTGTACGTTTATCACCGACTGACGAGTCCAGCGAGCTCATAATCCATGCTCCCTCGCAGCGGTGCCACACCCGATGTGGCGCAGAAAACGGTCATACCACCAGCATCATTACTGATTTGGAGGCGGTGGACGATGTTGCGCAGGTGCGCTGCCAGCAGCAGGCGCAGCGCGAGTTTGCGGAGCAGCTGGACGAGCTGTGGGGCAGCGAGCCCACCTACACGCCGACGGTAGAGGACCAAGCGAGCTGGCTGCAGCAGCTGCACTACGGATGGATCGGGGACTACATCTACAAGGCTGCGACGGGGAGCATTACCGAGGCGGACCTGCCACCGCCGTCGCGGAGCACACGGACCTACCACACGGGGCGCAAGCTGTCGCGGCAGGCGCATGCCGACATCGACGCAAGCCGGCGGTGGGACGGGTATGTCGGGTGCGAGGTCATGTACGAGGCGGAGGCGGAAACCAGCGGCGCGCTGCGGTGGGTCGGGTATTTGCAGCAGTCGGACTACCCGCGATCGCTGGTAGCTGGGGTGGAGTGGCGTGTGCCGCCGCGGTACCGGCGGCAGGCCACGCCGGGCAGCGCGGCGGGGCTTCACAACGGCGTCGTGCACGGTGAGCGGCTGTTTCGGCCATACGAAGACAACTATCTCTGCTCGTGCGAACCGGTTGAACGGCTTTACCTAAGCTCGACGTGCAGTCTGATGCGTCCCGGGCCGCCGCCGTCGCCGGATCTCCTGTTTACACTGTTCAAGGCGCACTCGTACCACGTGTGGGCGCAGATCCTGCCGAAGCTGCTGGCAGACGTTACCGCGCTGATGCTGCCGGTGTTGTTGGAGTACTTTGTGAAGTATTTGGACGCCGACAACGCGACGTGGGGCTGGGGTCTGGGACTTGTGCTGACACTCTTCCTCACGAACGTGATCCAGAGCTGCGCAGCGCACAAGTACGACCACATCAGCATCCGCAGCGCGGCGCTGTTTGAGACGTCGTCGATGGCGCTGCTGTTCGAGAAGTGCTTCACAGTTTCGCAGCGGTCGCTGCAGCGCCCAGACATGTCCGTGGGTCGCATCATGAACATGGTCGGGAATGACGTTGATAACATCGGTAGTCTCAACTGGTACGTGATGTATTTTTGGAGCGCCCCGCTGCAACTGACACTGTGCATGCTGCTGCTGATAAGGCTGGTCGGGTGGTTCGCGCTGCCCGGCATGGCCGTCTTGTTGGTGACGCTCCCGTTACAAGGTGCCATTTCTAAGCATGTTCAGGAGGTGTCTGAGCGAATGGCGAGCGTGGTGGACCTGCGTATCAAGCGCACGAGCGAGCTGCTCTCCGGTGCCCGAATCGTGAAATTCATGGGGTGGGAACCTGTCTTCCTCGCCCGGATCGAAGACGCGCGCAGTCGCGAGCTGCAGTGTCTGCGCGATGTGCATCTCGCCAATGTCATCTTCATGTTCGTGAACGACGCGACGCCGACGTTGGTCGTCGCTGTAGTCTTTATCTTGTACCATGCGAGTGGGCAGGCGCTGAGACCGGAGATTGTGTTTCCGACGATTGCACTGCTAAACACGATGCGCGTGTCGTTCTTTATGATCCCGCTTATCGTCTCCGCCATCCTGCAGTGTTTGGTGTCCACAAAGCGGGTCACCACTTTTGTGGAGTGCCCTGACACGCGCTCACAGGTGCGGGACATTGCTGGCATCGACGCAGCTGGTGCCGCGGCCATCTTCAAGGATGTGTCCATCCACACCTATCTGCCGGCGAAACTGCCCCAGTGCAAGTCGCGCTTGACGACCATGCAGCGCATTACGCTGTGGTTCCGCCGGCGCGGTGTGCCGGAGGCGGAGTGGTACGATCTGGACGGCCCGGATGCGGGTGCGTCTTCGCCGACGGTGCACCTTCCAACGCTCGACATGGGCAGCGCACAGACTGCCAGTGCGGATGGTGACGGCGCTGCCACTGGCGGCGGGGACGAGGAGGGGGACGTGGAAGAGAGAGTCGCACAGTACTACCAACTTGTGCCGAAGGAGCTGCTGCGGAACGTCAACCTCGTTATCCCTCAGTTCAAGTTGACGATGGTGATTGGCGCGACCGGGAGCGGGAAGTCGACGCTGCTGGGCTCTCTGATGGGCGAGTACGACGTGCAGAGTGGCGAGGTGTGGGCGGAGCGGAGCATCGCGTACGTGCCGCAGCAGGCGTGGATCATGAACGCGACGGTGCGCGACAACATCCTGTTCTTCGACGAGGAGCGCGCCGCAGACTTGCAGGACGTAATCCGTTGCTGCCAGCTGGAGGCGGACCTTGCGCAGCTTGGCGGGGGGCTGGAGACGGAGATCGGGGAAATGGGCGTGAACCTGAGCGGCGGGCAGAAGGCGCGCGTGAGCCTTGCGCGCGCCGTGTACGCGAACCGCGACGTGTACCTGCTGGACGACCCCCTGTCCGCGCTGGACGCGCACGTTGGCCAACGCGTCGTGCAAGACGTCATCCGCGGACGACTGCACGGCAAGACGCGCGTGCTTGCGACGCACCAGATTCATCTGCTGCCGCTCGCGGACTACATTGTGGTGTTGCAGCGCGGCCGCATCGCATTCGCGGGCGATTACGCTGCCTTTGCGGCGACTTCGCTGGAGGAGACGCTGCGCGGTGAGCTAAAGGAGAACAAGGACGCGGAGTCTCGCGGCAGCGATGCGGACGCGGAAGCGGCCAGAGCGGAGACGGCACCTGACATTGCGGAAGCGCATGAGCCGAAAGTTGAGCAGGAGACGAGCCTTGCAGGAGGCGAAGACCCCTTGAGGTCCGATGTCGAGGCCGGTAGGCTGATGACCAGGGAGGAGAAGGCAACCGGTCAGGTACCGTGGTCAACCTACGTGGCGTATCTGAGGTCGTGTGGCGGTCTGGCTGCTTGGGGATTCCTGCTAGCCACCTTTGCGGTGACAGAGAGCGTGACTGCGGCCAACGGCGTGTGGTTGTCGATATGGTCAACAGGCTCGCTTAGGTGTAGCGCGGCCACGTACCTGTACGTCTATCTTTTCATTGTTTTCCTCAACATCTTTAACTCCCCGCTGCGGTGTTTCTTATGCTATTACCTTATGCGAATGGGTAGCCGCAACTTGCATCGCGACCTCCTGGAGTCCATCGGCGTTGCGCGGATGTCCTTCTTCGACACGACGCCCTTGGGACGCGTGCTGAACCGGTTCACAAAGGACATGGGCATCCTTGATAACACGCTGAACGACAGCTACCTTTACCTGCTGCAGTACTTCTTTTCCATGTGCTCCAAGGTTATTATTCTGTCGGCCGCGCAGCCGTTTGTGCTTGTGGCCATTGTGCCGTGTGTGTTCATCTACTACAAGCTGATGCAGATCTACAGCGCGTCGAACCGCGAGACACGCCGCATCAAGAGCATCGCGCACTCGCCCGTGTTCACGCTGCTGGAGGAGTCGCTGCAAGGGCAGCGCACCATCGCGACGTACGGCAAGATGCACCTCGTACTGCAGGAAGCACTGAGGCGGCTCGATGTGGTCTACAGCGCGCTGTACATGCAGAACGTCTCCAACCGCTGGCTCGGTGTCCGCCTCGAGTTTGTGAGCTGTGTTATCACTTTCGTGGTGGCCCTCATTGGTGTGATTGGGAAAATGGAGCGGGCTTCGAGTCAGAGCATCGGTCTCATCTCGCTGTCACTGACGATGGCGATCACGCTGACGGAAACGCTCAATTGGCTGGTGCGGCAGGTTGCGACGGTGGAGGCAAACATGAACAGCGTGGAGCGTGTGATGTATTACACCCACGAAGTGGAGCACGAGTACGTGCCGGAGATGAAGGAGTTAGTGGCACAGCTGGTAGGGAGCGAGTCAGGGACAGCGGCGGAANNNNNNNNNNNNNNNNNNNNNNNNNNNNNNNNNNNNNNNNNNNNNNNNNNNNNNNNNNNNNNNNNNNNNNNNNNNNNNNNNNNNNNNNNNNNNNNNNNNNNNNNNNNNNNNNNNNNNNNNNNNNNNNNNNNNNNNNNNNNNNNNNNNNNNNNNNNNNNNNNNNNNNNNNNNNNNNNNNNNNNNNNNNNNNNNNNNNNNNNNNNNNNNNNNNNNNNNNNNNNNNNNNNNNNNNNNNNNNNNNNNNNNNNNNNNNNNNNNNNNNNNNNNNNNNNNNNNNNNNNNNNNNNNNNNNNNNNNNNNNNNNNNNNNNNNNNNNNNNNNNNNNNNNNNNNNNNNNNNNNNNNNNNNNNNNNNNNNNNNNNNNNNNNNNNNNNNNNNNNNNNNNNNNNNNNNNNNNNNNNNNNNNNNNNNNNNNNNNNNNNNNNNNNNNNNNNNNNNNNNNNNNNNNNNNNNNNNNNNNNNNNNNNNNNNNNNNNNNNNNNNNNNNNNNNNNNNNNNNNNNNNNNNNNNNNNNNNNNNNNNNNNNNNNNNNNNNNNNNNNNNNNNNNNNNNNNNNNNNNNNNNNNNNNNNNNNNNNNNNNNNNNNNNNNNNNNNNNNNNNNNNNNNNNNNNNNNNNNNNNNNNNNNNNNNNNNNNNNNNNNNNNNNNNNNNNNNNNNNNNNNNNNNNNNNNNNNNNNNNNNNNNNNNNNNNNNNNNNNNNNNNNNNNNNNNNNNNNNNNNNNNNNNNNNNNNNNNNNNNNNNNNNNNNNNNNNNNNNNNNNNNNNNNNNNNNNNNNNNNACTGGGCCGCCGTGGTGCCAAAGACTTCGTCGATCTCCTCGGCTGAGTCGCACGAGTCTGTGCAGCAGCCCGTGCGCACCAATGAAAGACTCCGTCGCAACCTGCCGATCTCAAAGCAGAAGCCGCTCGTCGTACGCCACCCCGCATGCACGTGCAGGAGAACGAAGTGCTCCACATGCCGTGTACCAATCTTGCCTTTGTCTTCTCCTATTCACGGCAGCGCGCTTGCATTTGCGCGATGGCGGTGCGTCGTCGGTACCCGCGGACTAGGCGCCACAGCGGCCATATGCTAGTTCATTGAATAGCACCTTACATCCTTACCGTTTCTGAGTGAAATCTGTATGCATGTGGAACACAAAGTCTTGCACGGCATAGCCGGCTAGGGGAGCGCCTCTGAGTCGTGCGTGACAGTATTGGCTGTACGATATCGTTCAAGACGCCACGAGTGCAGCTGCGCCGCCGCGACGGAGTCGCCTGCATGCCGGCTGAGGATGCGCGTGAAGGCGCGCCGATGCTTTCTCGGTGCTACTTTCTCCTTTCTCTCCC

>BPK298/0 clone 8|LinJ23_V3.0290|500 bp UPS + CDS + 500 bp DWS|multidrug resistance protein A

CTCCGCGCCGCATGCGAAGCCTGTTGTCCGCGTAGTTGCGTACGTGTGTATCTGCGCGCGTTGACTCGTGTCCTCTTCGCACCTCCTTCTGCTGGTTTGCCGCTCTCACCCGTAAGCGTGAATCAGTCTTCACGAGGCGCTTAGAGTTTCTTTTCTTTCGTGGAGGTGGTGGTCGCCAAGTGTGTGAGCTGATCGGTGCGCGAGGCGTGCGTGTGCCGAGTGCATCCTGTCCCCGACCCCCGCCTCTGGCTCCTCCGGTGCTACGCCTGAGTCTTCCCTGGCCCAGCCAGCACGGTGCCCGTCGTTGAAAGAGTGTGCCAAGACACACCTCGCCCCCTCGGTTTGTGCGCGGTCTGTCACACTGGTGCTGTTCGACCGCTGTTGTGCGGCTCGTAGGACAGGGTTGTGCGAACTACGCCTCCCTCGTTCTCTTCGCATAGGAAAGACATTGTAGACATCGGTGCATACATTGGGGGGGGGCATCCACAGCGCGCAGGGCAATGGCGACACCAGACTTTGTACGTTTATCACCGACTGACGAGTCCAGCGAGCTCATAATCCATGCTCCCTCGCAGCGGTGCCACACCCGATGTGGCGCAGAAAACGGTCATACCACCAGCATCATTACTGATTTGGAGGCGGTGGACGATGTTGCGCAGGTGCGCTGCCAGCAGCAGGCGCAGCGCGAGTTTGCGGAGCAGCTGGACGAGCTGTGGGGCAGCGAGCCCACCTACACGCCGACGGTAGAGGACCAAGCGAGCTGGCTGCAGCAGCTGCACTACGGATGGATCGGGGACTACATCTACAAGGCTGCGACGGGGAGCATTACCGAGGCGGACCTGCCACCGCCGTCGCGGAGCACACGGACCTACCACACGGGGCGCAAGCTGTCGCGGCAGGCGCATGCCGACATCGACGCAAGCCGGCGGTGGGACGGGTATGTCGGGTGCGAGGTCATGTACGAGGCGGAGGCGGAAACCAGCGGCGCGCTGCGGTGGGTCGGGTATTTGCAGCAGTCGGACTACCCGCGATCGCTGGTAGCTGGGGTGGAGTGGCGTGTGCCGCCGCGGTACCGGCGGCAGGCCACGCCGGGCAGCGCGGCGGGGCTTCACAACGGCGTCGTGCACGGTGAGCGGCTGTTTCGGCCATACGAAGACAACTATCTCTGCTCGTGCGAACCGGTTGAACGGCTTTACCTAAGCTCGACGTGCAGTCTGATGCGTCCCGGGCCGCCGCCGTCGCCGGATCTCCTGTTTACACTGTTCAAGGCGCACTCGTACCACGTGTGGGCGCAGATCCTGCCGAAGCTGCTGGCAGACGTTACCGCGCTGATGCTGCCGGTGTTGTTGGAGTACTTTGTGAAGTATTTGGACGCCGACAACGCGACGTGGGGCTGGGGTCTGGGACTTGTGCTGACACTCTTCCTCACGAACGTGATCCAGAGCTGCGCAGCGCACAAGTACGACCACATCAGCATCCGCAGCGCGGCGCTGTTTGAGACGTCGTCGATGGCGCTGCTGTTCGAGAAGTGCTTCACAGTTTCGCAGCGGTCGCTGCAGCGCCCAGACATGTCCGTGGGTCGCATCATGAACATGGTCGGGAATGACGTTGATAACATCGGTAGTCTCAACTGGTACGTGATGTATTTTTGGAGCGCCCCGCTGCAACTGACACTGTGCATGCTGCTGCTGATAAGGCTGGTCGGGTGGTTCGCGCTGCCCGGCATGGCCGTCTTGTTGGTGACGCTCCCGTTACAAGGTGCCATTTCTAAGCATGTTCAGGAGGTGTCTGAGCGAATGGCGAGCGTGGTGGACCTGCGTATCAAGCGCACGAGCGAGCTGCTCTCCGGTGCCCGAATCGTGAAATTCATGGGGTGGGAACCTGTCTTCCTCGCCCGGATCGAAGACGCGCGCAGTCGCGAGCTGCAGTGTCTGCGCGATGTGCATCTCGCCAATGTCATCTTCATGTTCGTGAACGACGCGACGCCGACGTTGGTCGTCGCTGTAGTCTTTATCTTGTACCATGCGAGTGGGCAGGCGCTGAGACCGGAGATTGTGTTTCCGACGATTGCACTGCTAAACACGATGCGCGTGTCGTTCTTTATGATCCCGCTTATCGTCTCCGCCATCCTGCAGTGTTTGGTGTCCACAAAGCGGGTCACCACTTTTGTGGAGTGCCCTGACACGCGCTCACAGGTGCGGGACATTGCTGGCATCGACGCAGCTGGTGCCGCGGCCATCTTCAAGGATGTGTCCATCCACACCTATCTGCCGGCGAAACTGCCCCAGTGCAAGTCGCGCTTGACGACCATGCAGCGCATTACGCTGTGGTTCCGCCGGCGCGGTGTGCCGGAGGCGGAGTGGTACGATCTGGACGGCCCGGATGCGGGTGCGTCTTCGCCGACGGTGCACCTTCCAACGCTCGACATGGGCAGCGCACAGACTGCCAGTGCGGATGGTGACGGCGCTGCCACTGGCGGCGGGGACGAGGAGGGGGACGTGGAAGAGAGAGTCGCACAGTACTACCAACTTGTGCCGAAGGAGCTGCTGCGGAACGTCAACCTCGTTATCCCTCAGTTCAAGTTGACGATGGTGATTGGCGCGACCGGGAGCGGGAAGTCGACGCTGCTGGGCTCTCTGATGGGCGAGTACGACGTGCAGAGTGGCGAGGTGTGGGCGGAGCGGAGCATCGCGTACGTGCCGCAGCAGGCGTGGATCATGAACGCGACGGTGCGCGACAACATCCTGTTCTTCGACGAGGAGCGCGCCGCAGACTTGCAGGACGTAATCCGTTGCTGCCAGCTGGAGGCGGACCTTGCGCAGCTTGGCGGGGGGCTGGAGACGGAGATCGGGGAAATGGGCGTGAACCTGAGCGGCGGGCAGAAGGCGCGCGTGAGCCTTGCGCGCGCCGTGTACGCGAACCGCGACGTGTACCTGCTGGACGACCCCCTGTCCGCGCTGGACGCGCACGTTGGCCAACGCGTCGTGCAAGACGTCATCCGCGGACGACTGCACGGCAAGACGCGCGTGCTTGCGACGCACCAGATTCATCTGCTGCCGCTCGCGGACTACATTGTGGTGTTGCAGCGCGGCCGCATCGCATTCGCGGGCGATTACGCTGCCTTTGCGGCGACTTCGCTGGAGGAGACGCTGCGCGGTGAGCTAAAGGAGAACAAGGACGCGGAGTCTCGCGGCAGCGATGCGGACGCGGAAGCGGCCAGAGCGGAGACGGCACCTGACATTGCGGAAGCGCATGAGCCGAAAGTTGAGCAGGAGACGAGCCTTGCAGGAGGCGAAGACCCCTTGAGGTCCGATGTCGAGGCCGGTAGGCTGATGACCAGGGAGGAGAAGGCAACCGGTCAGGTACCGTGGTCAACCTACGTGGCGTATCTGAGGTCGTGTGGCGGTCTGGCTGCTTGGGGATTCCTGCTAGCCACCTTTGCGGTGACAGAGAGCGTGACTGCGGCCAACGGCGTGTGGTTGTCGATATGGTCAACAGGCTCGCTTAGGTGTAGCGCGGCCACGTACCTGTACGTCTATCTTTTCATTGTTTTCCTCAACATCTTTAACTCCCCGCTGCGGTGTTTCTTATGCTATTACCTTATGCGAATGGGTAGCCGCAACTTGCATCGCGACCTCCTGGAGTCCATCGGCGTTGCGCGGATGTCCTTCTTCGACACGACGCCCTTGGGACGCGTGCTGAACCGGTTCACAAAGGACATGGGCATCCTTGATAACACGCTGAACGACAGCTACCTTTACCTGCTGCAGTACTTCTTTTCCATGTGCTCCAAGGTTATTATTCTGTCGGCCGCGCAGCCGTTTGTGCTTGTGGCCATTGTGCCGTGTGTGTTCATCTACTACAAGCTGATGCAGATCTACAGCGCGTCGAACCGCGAGACACGCCGCATCAAGAGCATCGCGCACTCGCCCGTGTTCACGCTGCTGGAGGAGTCGCTGCAAGGGCAGCGCACCATCGCGACGTACGGCAAGATGCACCTCGTACTGCAGGAAGCACTGAGGCGGCTCGATGTGGTCTACAGCGCGCTGTACATGCAGAACGTCTCCAACCGCTGGCTCGGTGTCCGCCTCGAGTTTGTGAGCTGTGTTATCACTTTCGTGGTGGCCCTCATTGGTGTGATTGGGAAAATGGAGCGGGCTTCGAGTCAGAGCATCGGTCTCATCTCGCTGTCACTGACGATGGCGATCACGCTGACGGAAACGCTCAATTGGCTGGTGCGGCAGGTTGCGACGGTGGAGGCAAACATGAACAGCGTGGAGCGTGTGATGTATTACACCCACGAAGTGGAGCACGAGTACGTGCCGGAGATGAAGGAGTTAGTGGCACAGCTGGTAGGGAGCGAGTCAGGGACAGCGGCGGAANNNNNNNNNNNNNNNNNNNNNNNNNNNNNNNNNNNNNNNNNNNNNNNNNNNNNNNNNNNNNNNNNNNNNNNNNNNNNNNNNNNNNNNNNNNNNNNNNNNNNNNNNNNNNNNNNNNNNNNNNNNNNNNNNNNNNNNNNNNNNNNNNNNNNNNNNNNNNNNNNNNNNNNNNNNNNNNNNNNNNNNNNNNNNNNNNNNNNNNNNNNNNNNNNNNNNNNNNNNNNNNNNNNNNNNNNNNNNNNNNNNNNNNNNNNNNNNNNNNNNNNNNNNNNNNNNNNNNNNNNNNNNNNNNNNNNNNNNNNNNNNNNNNNNNNNNNNNNNNNNNNNNNNNNNNNNNNNNNNNNNNNNNNNNNNNNNNNNNNNNNNNNNNNNNNNNNNNNNNNNNNNNNNNNNNNNNNNNNNNNNNNNNNNNNNNNNNNNNNNNNNNNNNNNNNNNNNNNNNNNNNNNNNNNNNNNNNNNNNNNNNNNNNNNNNNNNNNNNNNNNNNNNNNNNNNNNNNNNNNNNNNNNNNNNNNNNNNNNNNNNNNNNNNNNNNNNNNNNNNNNNNNNNNNNNNNNNNNNNNNNNNNNNNNNNNNNNNNNNNNNNNNNNNNNNNNNNNNNNNNNNNNNNNNNNNNNNNNNNNNNNNNNNNNNNNNNNNNNNNNNNNNNNNNNNNNNNNNNNNNNNNNNNNNNNNNNNNNNNNNNNNNNNNNNNNNNNNNNNNNNNNNNNNNNNNNNNNNNNNNNNNNNNNNNNNNNNNNNNNNNNNNNNNNNNNNNNNNNNNNNNNNNNNNNNNNNNNNNNNACTGGGCCGCCGTGGTGCCAAAGACTTCGTCGATCTCCTCGGCTGAGTCGCACGAGTCTGTGCAGCAGCCCGTGCGCACCAATGAAAGACTCCGTCGCAACCTGCCGATCTCAAAGCAGAAGCCGCTCGTCGTACGCCACCCCGCATGCACGTGCAGGAGAACGAAGTGCTCCACATGCCGTGTACCAATCTTGCCTTTGTCTTCTCCTATTCACGGCAGCGCGCTTGCATTTGCGCGATGGCGGTGCGTCGTCGGTACCCGCGGACTAGGCGCCACAGCGGCCATATGCTAGTTCATTGAATAGCACCTTACATCCTTACCGTTTCTGAGTGAAATCTGTATGCATGTGGAACACAAAGTCTTGCACGGCATAGCCGGCTAGGGGAGCGCCTCTGAGTCGTGCGTGACAGTATTGGCTGTACGATATCGTTCAAGACGCCACGAGTGCAGCTGCGCCGCCGCGACGGAGTCGCCTGCATGCCGGCTGAGGATGCGCGTGAAGGCGCGCCGATGCTTTCTCGGTGCTACTTTCTCCTTTCTCTCCC
