# Supplementary material for: Molecular Mechanisms of Drug Resistance in Natural Leishmania Populations Vary with Genetic Background
Source: PLoS Negl Trop Dis. 2012 Feb 28;6(2):e1514. doi: 10.1371/journal.pntd.0001514 (PMC3289598; doi:10.1371/journal.pntd.0001514)
Supplement: Dataset S3 — Relative levels of the proteins trypanothione reductase (TR) and mercaptopyruvate sulfurtransferase of 12 L. (L.) donovani clones with variable SSG susceptibility during 8 consecutive days of in vitro promastigote growth. The given relative protein expression levels (± SEM) are the average of 2 independent samples prepared from parallel cultures which were analysed on the same Western Blot, the listed values correspond to the normalised digitally integrated intensities of the protein bands. (NA = result not available). (DOC) [file pntd.0001514.s003.doc]

## Dataset S3.

#### Trypanothione reductase (TR)

| POPULATION A | | | | | | | | | | | | | | | | | | | |
| --- | --- | --- | --- | --- | --- | --- | --- | --- | --- | --- | --- | --- | --- | --- | --- | --- | --- | --- | --- |
| Time  point | | SSG-sensitive strains | | | | | | | | | SSG-resistant strains | | | | | | | | |
|  |  | BPK0206/0 clone 10 | | | BPK206/0 clone 14 | | | BPK206/0 clone 20 | | | BPK190/0 clone 3 | | | BPK190/0 clone 11 | | | BPK190/0 clone 19 | | |
| log phase | day 1 | NA | | | NA | | | NA | | | NA | | | NA | | | NA | | |
|  | day 2 | NA | | | NA | | | NA | | | NA | | | NA | | | NA | | |
|  | day 3 | NA | | | NA | | | NA | | | NA | | | NA | | | NA | | |
|  | day 4 | 1370 | ± | 17 | 645 | ± | 7 | 1838 | ± | 68 | 2938 | ± | 204 | 810 | ± | 15 | 592 | ± | 146 |
| stationary phase | day 5 | NA | | | NA | | | NA | | | NA | | | NA | | | NA | | |
|  | day 6 | 1460 | ± | 13 | 796 | ± | 1 | 1606 | ± | 93 | 2733 | ± | 152 | 274 | ± | 4 | 814 | ± | 372 |
|  | day 7 | 466 | ± | 112 | 1001 | ± | 101 | 1895 | ± | 210 | 2124 | ± | 45 | 588 | ± | 0 | 972 | ± | 0 |
|  | day 8 | 987 | ± | 1 | 467 | ± | 121 | 1781 | ± | 474 | 538 | ± | 167 | 914 | ± | 0 | 1322 | ± | 79 |

| POPULATION B | | | | | | | | | | | | | | | | | | | |
| --- | --- | --- | --- | --- | --- | --- | --- | --- | --- | --- | --- | --- | --- | --- | --- | --- | --- | --- | --- |
| Time  point | | SSG-sensitive strains | | | | | | SSG-resistant strains | | | | | | | | | | | |
|  |  | BPK282/0 clone 4 | | | BPK282/0 clone 9 | | | BPK275/0 clone 12 | | | BPK275/0 clone 15 | | | BPK275/0 clone 17 | | | BPK275/0 clone 18 | | |
| log phase | day 1 | NA | | | NA | | | NA | | | NA | | | NA | | | NA | | |
|  | day 2 | NA | | | NA | | | NA | | | NA | | | NA | | | NA | | |
|  | day 3 | NA | | | NA | | | NA | | | NA | | | NA | | | NA | | |
|  | day 4 | 1817 | ± | 0 | 1287 | ± | 55 | 440 | ± | 61 | 98 | ± | 0 | 62 | ± | 9 | 572 | ± | 102 |
| stationary phase | day 5 | NA | | | NA | | | NA | | | NA | | | NA | | | NA | | |
|  | day 6 | 1809 | ± | 0 | 1441 | ± | 167 | 302 | ± | 31 | 413 | ± | 103 | 491 | ± | 68 | 984 | ± | 0 |
|  | day 7 | 1626 | ± | 2 | 556 | ± | 197 | 308 | ± | 261 | 28 | ± | 6 | 628 | ± | 0 | 480 | ± | 194 |
|  | day 8 | 1861 | ± | 90 | 961 | ± | 70 | 227 | ± | 0 | 28 | ± | 27 | 217 | ± | 85 | 611 | ± | 98 |

#### Mercapto-pyruvate sulfurtransferase (MST)

| POPULATION A | | | | | | | | | | | | | | | | |
| --- | --- | --- | --- | --- | --- | --- | --- | --- | --- | --- | --- | --- | --- | --- | --- | --- |
| Time  point | | SSG-sensitive strains | | | | | | | | | SSG-resistant strains | | | | | |
|  |  | BPK0206/0 clone 10 | | | BPK206/0 clone 14 | | | BPK206/0 clone 20 | | | BPK190/0 clone 11 | | | BPK190/0 clone 19 | | |
| log phase | day 1 | NA | | | NA | | | NA | | | NA | | | NA | | |
|  | day 2 | NA | | | NA | | | NA | | | NA | | | NA | | |
|  | day 3 | NA | | | NA | | | NA | | | NA | | | NA | | |
|  | day 4 | 12.4 | ± | 5.3 | 9.4 | ± | 0.1 | 18.4 | ± | 1.1 | 5.9 | ± | 0.4 | 8.5 | ± | 0.0 |
| stationary phase | day 5 | NA | | | NA | | | NA | | | NA | | | NA | | |
|  | day 6 | 7.9 | ± | 1.2 | 6.6 | ± | 0.5 | 13.2 | ± | 0.6 | 1.1 | ± | 0.0 | 10.3 | ± | 0.3 |
|  | day 7 | 4.2 | ± | 0.0 | 6.3 | ± | 0.3 | 13.3 | ± | 0.3 | 4.3 | ± | 0.2 | 8.7 | ± | 0.4 |
|  | day 8 | 6.0 | ± | 0.2 | 2.6 | ± | 0.9 | 11.3 | ± | 0.0 | 2.0 | ± | 0.3 | 7.7 | ± | 0.5 |

| POPULATION B | | | | | | | | | | | | | | | | | | | |  |
| --- | --- | --- | --- | --- | --- | --- | --- | --- | --- | --- | --- | --- | --- | --- | --- | --- | --- | --- | --- | --- |
| Time  point | | SSG-sensitive strains | | | | | | SSG-resistant strains | | | | | | | | | | | |  |
|  |  | BPK282/0 clone 4 | | | BPK282/0 clone 9 | | | BPK275/0 clone 12 | | | BPK275/0 clone 15 | | | BPK275/0 clone 17 | | | BPK275/0 clone 18 | | | |
| log phase | day 1 | NA | | | NA | | | NA | | | NA | | | NA | | | NA | | | |
|  | day 2 | NA | | | NA | | | NA | | | NA | | | NA | | | NA | | | |
|  | day 3 | NA | | | NA | | | NA | | | NA | | | NA | | | NA | | | |
|  | day 4 | 6.9 | ± | 0.3 | 3.9 | ± | 0.1 | 8.1 | ± | 0.8 | 4.7 | ± | 0.1 | 4.8 | ± | 1.7 | 4.5 | ± | 0.1 | |
| stationary phase | day 5 | NA | | | NA | | | NA | | | NA | | | NA | | | NA | | | |
|  | day 6 | 7.1 | ± | 0.5 | 6.6 | ± | 0.3 | 14.1 | ± | 0.1 | 1.6 | ± | 0.1 | 6.4 | ± | 0.9 | 5.7 | ± | 0.5 | |
|  | day 7 | 6.6 | ± | 0.1 | 7.6 | ± | 2.9 | 4.5 | ± | 1.4 | 1.3 | ± | 0.1 | 5.1 | ± | 0.5 | 4.9 | ± | 1.6 | |
|  | day 8 | 2.1 | ± | 0.7 | 11.1 | ± | 1.2 | 5.1 | ± | 0.3 | 1.1 | ± | 0.1 | 6.3 | ± | 5.2 | 1.4 | ± | 0.3 | |

#### 
