# Supplementary material for: Molecular Mechanisms of Drug Resistance in Natural Leishmania Populations Vary with Genetic Background
Source: PLoS Negl Trop Dis. 2012 Feb 28;6(2):e1514. doi: 10.1371/journal.pntd.0001514 (PMC3289598; doi:10.1371/journal.pntd.0001514)
Supplement: Dataset S4 — Thiol levels of 12 L. (L.) donovani clones with variable SSG susceptibility during 8 consecutive days of in vitro promastigote growth. The thiol quantities (± SEM) are the average of 3 independent samples prepared from parallel cultures which were analysed in 1 HPLC experiment, the listed values are expressed in nmol/108 parasites. (NA = result not available). (DOC) [file pntd.0001514.s004.doc]

## Dataset S4.

#### Trypanothione

| POPULATION A | | | | | | | | | | | | | | | | | | | | | |
| --- | --- | --- | --- | --- | --- | --- | --- | --- | --- | --- | --- | --- | --- | --- | --- | --- | --- | --- | --- | --- | --- |
| Time  point | | SSG-sensitive strains | | | | | | | | | | | SSG-resistant strains | | | | | | | | |
|  |  | BPK0206/0 clone 10 | | | | | BPK206/0 clone 14 | | | BPK206/0 clone 20 | | | BPK190/0 clone 3 | | | BPK190/0 clone 11 | | | BPK087/0 clone 11 | | |
| log phase | day 1 | NA | | | | | NA | | | NA | | | NA | | | NA | | | NA | | |
|  | day 2 | NA | | | | | NA | | | NA | | | NA | | | NA | | | NA | | |
|  | day 3 | 6.5 | ± | | | 0.4 | NA | | | NA | | | 8.5 | ± | 0.2 | NA | | | 5.2 | ± | 0.6 |
|  | day 4 | NA | | | | | NA | | | NA | | | NA | | | NA | | | NA | | |
| stationary phase | day 5 | 2.2 | ± | | | 1.3 | 1.8 | ± | 0.2 | 1.4 | ± | 0.2 | 2.2 | ± | 1.0 | 2.0 | ± | 0.6 | 1.3 | ± | 0.1 |
|  | day 6 | NA | | | | | NA | | | NA | | | NA | | | NA | | | NA | | |
|  | day 7 | 0.0 | | ± | 0.0 | | 2.6 | ± | 0.3 | 1.7 | ± | 0.0 | 0.7 | ± | 0.7 | 4.3 | ± | 0.3 | 1.2 | ± | 0.1 |
|  | day 8 | 3.2 | | ± | 0.4 | | 0.6 | ± | 0.2 | 0.6 | ± | 0.1 | 3.3 | ± | 0.3 | 0.9 | ± | 0.0 | 3.0 | ± | 0.7 |

| POPULATION B | | | | | | | | | | | | | | | | | | | | | |
| --- | --- | --- | --- | --- | --- | --- | --- | --- | --- | --- | --- | --- | --- | --- | --- | --- | --- | --- | --- | --- | --- |
| Time  point | | SSG-sensitive strains | | | | | | | | SSG-resistant strains | | | | | | | | | | | |
|  |  | BPK0282/0 clone 4 | | | | | BPK282/0 clone 9 | | | BPK275/0 clone 12 | | | BPK275/0 clone 18 | | | BPK085/0 clone 3 | | | BPK085/0 clone 8 | | |
| log phase | day 1 | NA | | | | | NA | | | NA | | | NA | | | NA | | | NA | | |
|  | day 2 | NA | | | | | NA | | | NA | | | NA | | | NA | | | NA | | |
|  | day 3 | 4.0 | ± | | 0.3 | | 4.4 | ± | 0.2 | 3.7 | ± | 0.0 | 3.3 | ± | 0.1 | 6.6 | ± | 0. 6 | 6.8 | ± | 0.6 |
|  | day 4 | NA | | | | | NA | | | NA | | | NA | | | NA | | | NA | | |
| stationary phase | day 5 | 9.6 | ± | | 0.1 | | 9.0 | ± | 0.5 | 0.0 | ± | 0.0 | 1.2 | ± | 0.1 | 15.5 | ± | 0.0 | 11.7 | ± | 0.2 |
|  | day 6 | NA | | | | | NA | | | NA | | | NA | | | NA | | | NA | | |
|  | day 7 | 0.2 | | ± | | 0.2 | 0.3 | ± | 0.0 | 0.1 | ± | 0.1 | 0.3 | ± | 0.0 | 1.3 | ± | 0.5 | 0.7 | ± | 0.2 |
|  | day 8 | 0.3 | | ± | | 0.0 | 4.3 | ± | 0.7 | 0.4 | ± | 0.0 | 2.1 | ± | 0.3 | 4.1 | ± | 0.3 | 5.9 | ± | 0.1 |

#### Glutathione

| POPULATION A | | | | | | | | | | | | | | | | | | | | | |
| --- | --- | --- | --- | --- | --- | --- | --- | --- | --- | --- | --- | --- | --- | --- | --- | --- | --- | --- | --- | --- | --- |
| Time  point | | SSG-sensitive strains | | | | | | | | | | | SSG-resistant strains | | | | | | | | |
|  |  | BPK0206/0 clone 10 | | | | | BPK206/0 clone 14 | | | BPK206/0 clone 20 | | | BPK190/0 clone 3 | | | BPK190/0 clone 11 | | | BPK087/0 clone 11 | | |
| log phase | day 1 | NA | | | | | NA | | | NA | | | NA | | | NA | | | NA | | |
|  | day 2 | NA | | | | | NA | | | NA | | | NA | | | NA | | | NA | | |
|  | day 3 | 7.2 | ± | | | 0.4 | NA | | | NA | | | 8.9 | ± | 0.6 | NA | | | 15.9 | ± | 0.4 |
|  | day 4 | NA | | | | | NA | | | NA | | | NA | | | NA | | | NA | | |
| stationary phase | day 5 | 6.32 | ± | | | 1.8 | 9.6 | ± | 0.5 | 5.7 | ± | 1.0 | 14.2 | ± | 1.5 | 6.9 | ± | 0.9 | 8.8 | ± | 0.9 |
|  | day 6 | NA | | | | | NA | | | NA | | | NA | | | NA | | | NA | | |
|  | day 7 | 7.8 | | ± | 0.5 | | 5.5 | ± | 0.5 | 9.5 | ± | 1.0 | 9.3 | ± | 1.0 | 4.7 | ± | 1.3 | 6.3 | ± | 0.2 |
|  | day 8 | 4.0 | | ± | 0.2 | | 5.4 | ± | 0.2 | 5.7 | ± | 0.1 | 3.1 | ± | 0.2 | 5.0 | ± | 0.0 | 1.5 | ± | 0.4 |

| POPULATION B | | | | | | | | | | | | | | | | | | | |
| --- | --- | --- | --- | --- | --- | --- | --- | --- | --- | --- | --- | --- | --- | --- | --- | --- | --- | --- | --- |
| Time  point | | SSG-sensitive strains | | | | | | SSG-resistant strains | | | | | | | | | | | |
|  |  | BPK0282/0 clone 4 | | | BPK282/0 clone 9 | | | BPK275/0 clone 12 | | | BPK275/0 clone 18 | | | BPK085/0 clone 3 | | | BPK085/0 clone 8 | | |
| log phase | day 1 | NA | | | NA | | | NA | | | NA | | | NA | | | NA | | |
|  | day 2 | NA | | | NA | | | NA | | | NA | | | NA | | | NA | | |
|  | day 3 | 8.8 | ± | 0.3 | 7.6 | ± | 0.2 | 4.4 | ± | 0.4 | 3.7 | ± | 0.1 | 18.2 | ± | 1.2 | 18.6 | ± | 2.2 |
|  | day 4 | NA | | | NA | | | NA | | | NA | | | NA | | | NA | | |
| stationary phase | day 5 | 16.3 | ± | 2.2 | 8.4 | ± | 0.8 | 14.1 | ± | 0.6 | 12.4 | ± | 1.1 | 9.3 | ± | 1.0 | 4.3 | ± | 0.3 |
|  | day 6 | NA | | | NA | | | NA | | | NA | | | NA | | | NA | | |
|  | day 7 | 11.7 | ± | 0.5 | 14.5 | ± | 0.7 | 19.7 | ± | 0.4 | 12.8 | ± | 0.1 | 20.1 | ± | 0.2 | 17.6 | ± | 1.4 |
|  | day 8 | 5.1 | ± | 0.2 | 7.9 | ± | 0.5 | 9.6 | ± | 0.1 | 7.9 | ± | 0.6 | 6.8 | ± | 0.5 | 5.6 | ± | 0.1 |

#### Cysteine

| POPULATION A | | | | | | | | | | | | | | | | | | | | | |
| --- | --- | --- | --- | --- | --- | --- | --- | --- | --- | --- | --- | --- | --- | --- | --- | --- | --- | --- | --- | --- | --- |
| Time  point | | SSG-sensitive strains | | | | | | | | | | | SSG-resistant strains | | | | | | | | |
|  |  | BPK0206/0 clone 10 | | | | | BPK206/0 clone 14 | | | BPK206/0 clone 20 | | | BPK190/0 clone 3 | | | BPK190/0 clone 11 | | | BPK087/0 clone 11 | | |
| log phase | day 1 | NA | | | | | NA | | | NA | | | NA | | | NA | | | NA | | |
|  | day 2 | NA | | | | | NA | | | NA | | | NA | | | NA | | | NA | | |
|  | day 3 | 18.8 | ± | | | 0.6 | NA | | | NA | | | 18.2 | ± | 0.4 | NA | | | 18.3 | ± | 0.3 |
|  | day 4 | NA | | | | | NA | | | NA | | | NA | | | NA | | | NA | | |
| stationary phase | day 5 | 8.5 | ± | | | 1.2 | 8.7 | ± | 0.7 | 5.9 | ± | 2.0 | 10.9 | ± | 1.7 | 6.8 | ± | 2.0 | 11.0 | ± | 1.7 |
|  | day 6 | NA | | | | | NA | | | NA | | | NA | | | NA | | | NA | | |
|  | day 7 | 4.7 | | ± | 0.4 | | 14.5 | ± | 0.8 | 6.6 | ± | 0.3 | 6.5 | ± | 0.7 | 9.8 | ± | 9.89 | 11.8 | ± | 1.0 |
|  | day 8 | 17.8 | | ± | 0.2 | | 15.2 | ± | 0.5 | 4.8 | ± | 0.1 | 17.4 | ± | 0.5 | 10.1 | ± | 0.0 | 7.2 | ± | 1.8 |

| POPULATION B | | | | | | | | | | | | | | | | | | | |
| --- | --- | --- | --- | --- | --- | --- | --- | --- | --- | --- | --- | --- | --- | --- | --- | --- | --- | --- | --- |
| Time  point | | SSG-sensitive strains | | | | | | SSG-resistant strains | | | | | | | | | | | |
|  |  | BPK0282/0 clone 4 | | | BPK282/0 clone 9 | | | BPK275/0 clone 12 | | | BPK275/0 clone 18 | | | BPK085/0 clone 3 | | | BPK085/0 clone 8 | | |
| log phase | day 1 | NA | | | NA | | | NA | | | NA | | | NA | | | NA | | |
|  | day 2 | NA | | | NA | | | NA | | | NA | | | NA | | | NA | | |
|  | day 3 | 19.9 | ± | 0.9 | 20.4 | ± | 0.5 | 18.0 | ± | 0.4 | 19.1 | ± | 0.5 | 18.4 | ± | 1.3 | 19.1 | ± | 0.7 |
|  | day 4 | NA | | | NA | | | NA | | | NA | | | NA | | | NA | | |
| stationary phase | day 5 | 7.0 | ± | 0.8 | 6.7 | ± | 1.6 | 6.2 | ± | 0.3 | 5.9 | ± | 0.5 | 9.1 | ± | 0.3 | 5.5 | ± | 0.6 |
|  | day 6 | NA | | | NA | | | NA | | | NA | | | NA | | | NA | | |
|  | day 7 | 5.2 | ± | 0.7 | 8.6 | ± | 1.3 | 6.1 | ± | 0.2 | 4.2 | ± | 0.2 | 15.2 | ± | 1.2 | 9.1 | ± | 0.7 |
|  | day 8 | 5.2 | ± | 0.7 | 7.4 | ± | 1.2 | 4.3 | ± | 0.5 | 3.8 | ± | 0.5 | 19.6 | ± | 0.2 | 19.3 | ± | 0.9 |
