# Supplementary material for: Molecular Mechanisms of Drug Resistance in Natural Leishmania Populations Vary with Genetic Background
Source: PLoS Negl Trop Dis. 2012 Feb 28;6(2):e1514. doi: 10.1371/journal.pntd.0001514 (PMC3289598; doi:10.1371/journal.pntd.0001514)
Supplement: Dataset S5 — In vitro susceptibility to H202, SNAP and SbIII of 12 L. (L.) donovani clones with variable SSG susceptibility during 8 consecutive days of in vitro promastigote growth. The given IC50 values [±95 CI] were determined by sigmoidal regression analysis based on the % parasite viability following 48 hrs exposure to 6 different concentrations of the tested compound. (NA = result not available). (DOC) [file pntd.0001514.s005.doc]

## Dataset S5.

#### in vitro H_2_0_2_ susceptibility

| POPULATION A | | | | | | | | | | | | | |
| --- | --- | --- | --- | --- | --- | --- | --- | --- | --- | --- | --- | --- | --- |
| Time  point | | SSG-sensitive strains | | | | | | SSG-resistant strains | | | | | |
|  |  | BPK0206/0 clone 10 | | BPK206/0 clone 14 | | BPK206/0 clone 20 | | BPK190/0 clone 3 | | BPK190/0 clone 11 | | BPK190/0 clone 19 | |
| log phase | day 1 | NA | | NA | | NA | | NA | | NA | | NA | |
|  | day 2 | NA | | NA | | NA | | NA | | NA | | NA | |
|  | day 3 | NA | | NA | | NA | | NA | | NA | | NA | |
|  | day 4 | NA | | NA | | NA | | NA | | NA | | NA | |
| stationary phase | day 5 | 362.6 | [353.3, 372.1] | 398.7 | [389.5, 408.1] | 400.7 | [391.3, 410.2] | 344.8 | [341.4, 348.2] | 362.2 | [354.9, 369.6] | 284.2 | [280.0, 288.5] |
|  | day 6 | 364.8 | [359.1, 370.5] | 441.1 | [433.9, 448.5] | 312.3 | [305.0, 319.7] | 432.8 | [422.4, 443.3] | 352.9 | [345.5, 360.5] | 197.1 | [194.5, 199.6] |
|  | day 7 | 557.0 | [538.0, 576.6] | 516.1 | [508.3, 524.1] | 410.9 | [402.3, 419.6] | 502.2 | [490.7, 513.8] | 527.9 | [508.6, 547.9] | 537.7 | [515.0, 561.5] |
|  | day 8 | NA | | NA | | NA | | NA | | NA | | NA | |

| POPULATION B | | | | | | | | | | | | | |
| --- | --- | --- | --- | --- | --- | --- | --- | --- | --- | --- | --- | --- | --- |
| Time  point | | SSG-sensitive strains | | | | SSG-resistant strains | | | | | | | |
|  |  | BPK0282/0 clone 4 | | BPK282/0 clone 9 | | BPK275/0 clone 12 | | BPK275/0 clone 15 | | BPK275/0 clone 17 | | BPK275/0 clone 18 | |
| log phase | day 1 | NA | | NA | | NA | | NA | | NA | | NA | |
|  | day 2 | NA | | NA | | NA | | NA | | NA | | NA | |
|  | day 3 | NA | | NA | | NA | | NA | | NA | |  | |
|  | day 4 | NA | | NA | | NA | | NA | | NA | | NA | |
| stationary phase | day 5 | 340.2 | [335.6, 344.8] | 431.2 | [412.8, 450.4] | 219.2 | [215.0, 223.4] | 244.8 | [240.7, 248.8] | 307.7 | [304.0, 311.3] | 289.4 | [285.9, 292.9] |
|  | day 6 | 582.3 | [549.4, 617.2] | 528.6 | [505.2, 553.1] | 335.8 | [333.3, 338.3] | 321.2 | [315.2, 327.4] | 317.3 | [303.0, 332.3] | 306.9 | [297.4, 316.6] |
|  | day 7 | 510.3 | [455.4, 571.7] | 509.8 | [451.9, 575.1] | 309.7 | [303.2, 316.3] | 331.7 | [322.7, 341.0] | 337.3 | [330.2, 344.5] | 394.9 | [385.4, 404.7] |
|  | day 8 | NA | | NA | | NA | | NA | | NA | | NA | |

#### in vitro SNAP susceptibility

| POPULATION A | | | | | | | | | | | | | |
| --- | --- | --- | --- | --- | --- | --- | --- | --- | --- | --- | --- | --- | --- |
| Time  point | | SSG-sensitive strains | | | | | | SSG-resistant strains | | | | | |
|  |  | BPK0206/0 clone 10 | | BPK206/0 clone 14 | | BPK206/0 clone 20 | | BPK190/0 clone 3 | | BPK190/0 clone 11 | | BPK190/0 clone 19 | |
| log phase | day 1 | NA | | NA | | NA | | NA | | NA | | NA | |
|  | day 2 | NA | | NA | | NA | | NA | | NA | | NA | |
|  | day 3 | NA | | NA | | NA | | NA | | NA | | NA | |
|  | day 4 | NA | | NA | | NA | | NA | | NA | | NA | |
| stationary phase | day 5 | 13.9 | [13.1, 14.7] | 31.2 | [30.0, 32.4] | 19.9 | [19.0, 20.9] | 17.5 | [16.5, 18.7] | 14.6 | [13.6, 15.7] | 31.0 | [30.2, 31.8] |
|  | day 6 | NA | | 41.4 | [35.1, 48.8] | 33.1 | [31.1, 35.2] | 21.9 | [20.7, 23.2] | 24.4 | [23.3, 25.5] | 29.1 | [28.0, 30.3] |
|  | day 7 | 45.3 | [43.4, 47.2] | 48.7 | [37.4, 63.5] | 55.0 | [49.7, 60.7] | 44.0 | [41.3, 47.0] | 33.3 | [31.3, 35.3] | 27.4 | [25.2, 29.7] |
|  | day 8 | NA | | NA | | NA | | NA | | NA | | NA | |

| POPULATION B | | | | | | | | | | | | | |
| --- | --- | --- | --- | --- | --- | --- | --- | --- | --- | --- | --- | --- | --- |
| Time  point | | SSG-sensitive strains | | | | SSG-resistant strains | | | | | | | |
|  |  | BPK0282/0 clone 4 | | BPK282/0 clone 9 | | BPK275/0 clone 12 | | BPK275/0 clone 15 | | BPK275/0 clone 17 | | BPK275/0 clone 18 | |
| log phase | day 1 | NA | | NA | | NA | | NA | | NA | | NA | |
|  | day 2 | NA | | NA | | NA | | NA | | NA | | NA | |
|  | day 3 | NA | | NA | | NA | | NA | | NA | |  | |
|  | day 4 | NA | | NA | | NA | | NA | | NA | | NA | |
| stationary phase | day 5 | 30.2 | [29.4, 31.0] | 22.9 | [22.5, 23.4] | 13.4 | [12.6, 14.3] | 12.8 | [12.1, 13.5] | 10.7 | [10.3, 11.0] | 8.9 | [8.71, 9.12] |
|  | day 6 | 19.9 | [18.3, 21.8] | 33.2 | [31.4, 35.1] | 14.8 | [14.1, 15.6] | 14.8 | [13.0, 16.8] | 13.1 | [12.4, 13.8] | 18.4 | [17.0, 20.0] |
|  | day 7 | 58.2 | [50.3, 67.3] | 45.1 | [40.7, 49.9] | 36.8 | [35.1, 38.6] | 38.7 | [36.7, 40.7] | 47.1 | [41.2, 53.9] | NA | |
|  | day 8 | NA | | NA | | NA | | NA | | NA | | NA | |

#### in vitro SbIII susceptibility

| POPULATION A | | | | | | | | | | | | | |
| --- | --- | --- | --- | --- | --- | --- | --- | --- | --- | --- | --- | --- | --- |
| Time  point | | SSG-sensitive strains | | | | | | SSG-resistant strains | | | | | |
|  |  | BPK0206/0 clone 10 | | BPK206/0 clone 14 | | BPK206/0 clone 20 | | BPK190/0 clone 3 | | BPK190/0 clone 11 | | BPK190/0 clone 19 | |
| log phase | day 1 | NA | | NA | | NA | | NA | | NA | | NA | |
|  | day 2 | NA | | NA | | NA | | NA | | NA | | NA | |
|  | day 3 | NA | | NA | | NA | | NA | | NA | | NA | |
|  | day 4 | NA | | NA | | NA | | NA | | NA | | NA | |
| stationary phase | day 5 | 125.0 | [120.8, 129.3] | 36.4 | [34.1, 38.8] | 25.5 | [24.0, 27.1] | 51.4 | [47.4, 55.6] | 39.4 | [35.9, 43.2] | 29.5 | [28.3, 30.7] |
|  | day 6 | 58.8 | [50.8, 68.01] | 49.7 | [46.7, 53.0] | 107.2 | [99.8, 115.1] | 70.9 | [63.5, 79.1] | 45.4 | [34.7, 59.6] | 36.7 | [33.6, 39.9] |
|  | day 7 | 182.6 | [165.1, 201.9] | 104.2 | [75.2, 144.3] | 118.2 | [103.0, 135.8] | 133.8 | [120.5, 148.6] | 119.4 | [107.6, 132.6] | 57.2 | [51.5, 63.6] |
|  | day 8 | NA | | NA | | NA | | NA | | NA | | NA | |

| POPULATION B | | | | | | | | | | | | | |
| --- | --- | --- | --- | --- | --- | --- | --- | --- | --- | --- | --- | --- | --- |
| Time  point | | SSG-sensitive strains | | | | SSG-resistant strains | | | | | | | |
|  |  | BPK0282/0 clone 4 | | BPK282/0 clone 9 | | BPK275/0 clone 12 | | BPK275/0 clone 15 | | BPK275/0 clone 17 | | BPK275/0 clone 18 | |
| log phase | day 1 | NA | | NA | | NA | | NA | | NA | | NA | |
|  | day 2 | NA | | NA | | NA | | NA | | NA | | NA | |
|  | day 3 | NA | | NA | | NA | | NA | | NA | |  | |
|  | day 4 | NA | | NA | | NA | | NA | | NA | | NA | |
| stationary phase | day 5 | 266.8 | [252.6, 281.8] | 370.0 | [343.2, 398.8] | 127.9 | [122.0, 134.2] | 119.4 | [111.7, 127.6] | 181.4 | [167.5, 196.5] | 127.1 | [118.5, 136.4] |
|  | day 6 | 114.8 | [106.3, 124.0] | 115.8 | [109.0, 123.0] | 83.6 | [78.5, 89.0] | 81.5 | [73.1, 90.8] | 72.1 | [69.0, 75.4] | 61.9 | [53.3, 72.0] |
|  | day 7 | 2.0 | [1.7, 2.4] | 2.1 | [1.7, 2.5] | 120.7 | [109.1, 133.6] | 67.5 | [63.0, 72.4] | 69.4 | [65.3, 73.8] | 77.3 | [72.1, 82.8] |
|  | day 8 | NA | | NA | | NA | | NA | | NA | | NA | |
